# Characterization of the dual role of *Plasmodium falciparum* DNA methyltransferase in regulating transcription and translation

**DOI:** 10.1093/nar/gkad248

**Published:** 2023-04-07

**Authors:** Amuza B Lucky, Chengqi Wang, Xiaolian Li, Anongruk Chim-Ong, Swamy R Adapa, Eoin P Quinlivan, Rays Jiang, Liwang Cui, Jun Miao

**Affiliations:** Department of Internal Medicine, Morsani College of Medicine, University of South Florida, 3720 Spectrum Blvd, Tampa, FL, USA; Center for Global Health and Infectious Diseases Research, College of Public Health, University of South Florida, 3720 Spectrum Blvd, Tampa, FL, USA; Department of Internal Medicine, Morsani College of Medicine, University of South Florida, 3720 Spectrum Blvd, Tampa, FL, USA; Department of Internal Medicine, Morsani College of Medicine, University of South Florida, 3720 Spectrum Blvd, Tampa, FL, USA; Center for Global Health and Infectious Diseases Research, College of Public Health, University of South Florida, 3720 Spectrum Blvd, Tampa, FL, USA; Department of Pathology, Immunology and Laboratory Medicine, University of Florida, Gainesville, FL, USA; Center for Global Health and Infectious Diseases Research, College of Public Health, University of South Florida, 3720 Spectrum Blvd, Tampa, FL, USA; Department of Internal Medicine, Morsani College of Medicine, University of South Florida, 3720 Spectrum Blvd, Tampa, FL, USA; Center for Global Health and Infectious Diseases Research, College of Public Health, University of South Florida, 3720 Spectrum Blvd, Tampa, FL, USA; Department of Internal Medicine, Morsani College of Medicine, University of South Florida, 3720 Spectrum Blvd, Tampa, FL, USA; Center for Global Health and Infectious Diseases Research, College of Public Health, University of South Florida, 3720 Spectrum Blvd, Tampa, FL, USA

## Abstract

DNA modifications are critical in fine-tuning the biological processes in model organisms. However, the presence of cytosine methylation (5mC) and the function of the putative DNA methyltransferase, PfDNMT2, in the human malaria pathogen, *Plasmodium falciparum*, remain controversial. Here, we revisited the 5mC in the parasite genome and the function of *PfDNMT2*. Low levels of genomic 5mC (0.1–0.2%) during asexual development were identified using a sensitive mass spectrometry procedure. Native PfDNMT2 displayed substantial DNA methylation activities, and disruption or overexpression of PfDNMT2 resulted in reduced or elevated genomic 5mC levels, respectively. *PfDNMT2* disruption led to an increased proliferation phenotype, with the parasites having an extended schizont stage and producing a higher number of progenies. Consistent with PfDNMT2’s interaction with an AP2 domain-containing transcription factor, transcriptomic analyses revealed that *PfDNMT2* disruption led to a drastic alteration in the expression of many genes, some of which provided the molecular basis of enhanced proliferation after *PfDNMT2* disruption. Furthermore, levels of tRNA^Asp^ and its methylation rate at position C38, and the translation of a reporter containing an aspartate repeat were significantly reduced after *PfDNMT2* disruption, while the levels of tRNA^Asp^ and its C38 methylation were restored after complementation of *PfDNMT2*. Our study sheds new light on the dual function of PfDNMT2 during *P. falciparum* asexual development.

## INTRODUCTION

DNA methylation plays a critical role in numerous biological functions via an inheritable epigenetic regulatory mechanism through regulation of transcription, chromatin structure, and chromosome stability by genomic imprinting, X-chromosome inactivation, and suppression of repetitive elements (1–4). DNA methylation is conserved in most major eukaryotic organisms, including plants, animals, and fungi, with a few exceptions in certain model organisms such as the budding yeast *Saccharomyces cerevisiae* and the nematode worm *Caenorhabditis elegans* (5,6). In general, DNA methylation occurs ‘globally’ in vertebrates, with CG sites being heavily methylated genome-wide except for those in CpG islands, whereas invertebrates, plants and fungi have a ‘mosaic’ methylation landscape, characterized by sporadically methylated and unmethylated areas (3,5). Methylation at the CG sites is the major type, mostly symmetrical on both DNA strands, whereas methylation in non-CG contexts (CHG and CHH, where H can be any nucleotide but G) mainly occurs in plants and certain human developmental stages such as embryonic stem cells, germ cells and differentiated neuronal cells (3,7–12).

DNA methylation at cytosine is catalyzed by DNA methyltransferases (DNMTs), and it is a widely accepted paradigm that methylation is introduced *de novo* by the DNMT3 DNA methyltransferase. DNMT1 maintains methylation patterns by methylating hemimethylated CG, although accumulated evidence indicates functional overlaps of both DNMTs (4,10). DNMT2 was the third DNMT based on its conserved motifs among DNMTs (13). However, its function is still equivocal, and data from previous studies disputed whether DNMT2 is a primary DNMT or redundant (13). It localizes in the nucleus of *Entamoeba histolytica* (14), distributes in both the cytoplasm and nucleus of the *Drosophila* embryo (15), and shuttles from the nucleus to cytoplasm during *Dictyostelium discoideum* development from S-phase to mitosis (16), suggesting it has a dual function. In agreement with its nuclear and cytoplasmic localization, the recombinant DNMT2 proteins from *E. histolytica*, *Homo sapiens*, and *Spodoptera frugiperda* have low-level cytosine methylation activities on DNA, but relatively high-level RNA methylation activities on tRNA^ASP^ (14,17–19). However, other studies showed that DNMT2s from humans and *D. melanogaster* had no detectable *in vitro* DNA methylation activities (19,20). Loss of DNMT2 had only subtle phenotypic effects in *Mus musculus*, *D. melanogaster*, *S. pombe* and *D. discoideum* (13,21,22). In sharp contrast, DNMT2 is essential for *E. histolytica* and *Danio rerio* (23,24). In the genomes of ‘DNMT2 only’ organisms, such as *D. melanogaster, D. discoideum*, *S. pombe* and *E. histolytica*, which harbor one DNMT2 ortholog as the only known DNMT, DNA is methylated at a low-level without an obvious methylation pattern except that CG methylation is not prevalent in these organisms (13,25–30).

The notorious malaria parasite *Plasmodium falciparum*, responsible for about half a million human deaths annually (31), is another ‘DNMT2 only’ organism with a single PfDNMT2 (PF3D7_0727300) gene identified (32,33). *P. falciparum* undergoes a 48-h developmental cycle in human red blood cells (RBCs) through morphologically different stages (ring, trophozoite, and schizont) (34). This intraerythrocytic development cycle (IDC) is tightly controlled by multiple regulatory mechanisms, including the selection and activation of gene-specific transcription factors and epigenetic mechanisms such as histone modifications (34–42). DNA methylation in *P. falciparum* remains obscure. Early studies failed to identify methylated cytosine in *P. falciparum*, whereas partial methylation in the *dihydrofolate reductase-thymidylate synthase* (*dhfr-ts*) coding region was detected (43,44). Two attempts to determine the 5-methyl-2’-deoxycytidine (5mC) levels in the *P. falciparum* genome using the DNA bisulfite sequencing approach led to controversial results, with one study detecting a relatively high level (0.36–1.3%) but the other an extremely low level (0.01–0.05%) (32,45). Recent studies reported that two truncated PfDNMT2 proteins expressed in bacteria showed weak or no DNA methylation activity (32,46), whereas the latter protein also harbored apparent methylation activity on C38 of the tRNA^ASP^. Conditional knockout of PfDNMT2 resulted in no noticeable growth phenotype, but methylation of the tRNA^ASP^ C38 was significantly reduced, with a concomitant reduction in the expression of Asp (D)-rich proteins (33). These alterations were considered the reasons why PfDNMT knockout (KO) parasites became more sensitive to stress and prone to gametocyte production.

These controversial studies have prompted us to perform a detailed functional analysis of PfDNMT2 in *P. falciparum*. Here, using an accurate mass spectrometry method, we determined the presence of low levels of 5mC in the parasite genomic DNA during the IDC. We conclusively demonstrated the substantial DNA methylation activity of PfDNMT2 using an *in vitro* enzyme assay. Interestingly, the *PfDNMT2*-disrupted parasite had a more rapid growth phenotype partially due to enhanced merozoite production and invasion efficiency, which were further verified by genetic complementation and overexpression of *PfDNMT2*. Transcriptomic analysis revealed considerable alterations in gene expression that were consistent with the growth phenotype. In addition, the disruptant line showed substantially reduced methylation of the tRNA^ASP^ C38 and the level of tRNA^ASP^, correlating with reduced synthesis of a poly-D-containing reporter. This study provides solid evidence of the dual role of PfDNMT2 in DNA and tRNA methylation in *P. falciparum*, which influences gene expression and protein translation.

## MATERIALS AND METHODS

### Parasites culture, leucocyte depletion, and isolation of DNA

The *P. falciparum* 3D7 strain and transgenic lines were cultured according to a standard method with 0.5% AlbuMAX II added to the buffered medium (RPMI-1640 supplemented with 25 mM HEPES, 50 mg/l hypoxanthine, 40 mg/ml gentamycin sulfate and 25 mM sodium bicarbonate) and O^+^ human RBCs. Cultivation was performed at 37°C in a gas mixture of 5% CO_2_, 3% O_2_ and 92% N_2_ (47). To minimize DNA contamination, parasites used for DNA methylation analysis were cultured in leucocyte (white blood cell, WBC)-depleted blood by removing the ‘buffy coat’ (48) followed by using Plasmodipur filters (EuroProxima) for filtering WBCs (49) and keeping blood at 4°C for 1 week for the WBC decay (50). The number of WBCs in the blood was measured by flow cytometry after nuclear DNA was stained by Hoechst 33342. Parasite development was synchronized twice with 5% sorbitol treatment, and genomic DNA was purified at the ring, trophozoite and schizont stages according to established methods (51,52).

### Mass spectrometry-based quantification of DNA methylation in *P. falciparum*

Global 5mC levels of the parasite genome at the ring, trophozoite and schizont stages were quantitively analyzed by liquid chromatography coupled with tandem mass spectrometry (LC–MS/MS) (53,54). Briefly, [*U*-^15^N]-labeled nucleoside internal standard ([^15^N_3_]-5C, [^15^N_3_]-5mC) was added to 1 μg DNA/sample, which had been digested completely to nucleosides for 14–16 h following our simplified one-step protocol (54) before the mixture was analyzed by LC–MS/MS. Methyldeoxycytidine and deoxycytidine concentrations were calculated using the analyte/internal standard peak area ratios and standard curves. The percentage of methyldeoxycytidine was calculated by dividing methyldeoxycytidine concentration by total deoxycytidine concentration (methyldeoxycytidine plus deoxycytidine). The same approach was also used to measure the 5-hydroxymethyl-cytidine (5hmC) by using [D3]-5hmC as an internal standard.

### Tagging, disruption, and episomal expression of PfDNMT2

To tag PfDNMT2 with PTP or GFP, a C-terminus *PfDNMT2* fragment [nucleotides (nt) 948–2118] was amplified using primers Int-F and Int-R (Supplementary Table S1) from *P. falciparum* genomic DNA and cloned into modified pBluescript SK to fuse with the PTP or GFP and pDT 3' UTR as described earlier (55,56). The PTP tag consists of two protein A- and one C-binding motifs for protein purification using a tandem affinity purification (TAP) procedure (57–59). This cassette was then subcloned into pHD22Y at the *Bam*HI and *Not*I sites to produce pHD22Y/DNMT2-PTP/GFP (56). To disrupt *PfDNMT2* by single cross-over recombination, a *PfDNMT2* fragment [nt 2–1089] was amplified using primers KO-F and KO-R (Supplementary Table S1) and cloned into pHD22Y at the *Spe*I and *Not*I sites to produce the plasmid pHD22Y/ΔDNMT2. For episomal expression of PfDNMT2, the entire length of *PfDNMT2* was amplified using primers Exp-F and Exp-R (stop codon was omitted) (Supplementary Table S1) and cloned into modified pBluescript SK between HSP86 5' and pDT 3' UTRs to produce pBlue/DNMT. The GFP sequence was cloned at the end of PfDNMT in pBlue/DNMT to form pBlue/DNMT-GFP. The dihydroorotate dehydrogenase (DHODH) drug cassette was released from pUF1-Cas9 (60) and subcloned at *Spe*I and *Xba*I into pBlue/DNMT-GFP to produce pBlue/DNMT2-Exp.

Parasite transfection was done by an RBC loading method (61). Briefly, 100 μg of plasmid was introduced into fresh RBCs by electroporation. Purified schizonts were used to infect the loaded RBCs and selected with 2.5 nM of WR99210 or 1.5 μM of DSM1 for approximately four weeks with weekly replenishment of fresh RBCs until resistant parasites appeared. Resistant parasites were subjected to three cycles of on-off drug selection (62), and single clones of parasites with stable integration of the constructs were obtained by limiting dilution (63). Correct integration of the plasmids into the parasite genome was screened by integration-specific PCR, consisting of a forward primer upstream of the homologous region and a reverse primer located in the PTP or GFP tag (Supplementary Table S1). Correct integration resulting in *PfDNMT2* domain deletion was further confirmed by Southern blot with a specific probe located in the homologous region, which was generated by DIG-labeling PCR (Supplementary Table S1).

### Expression of PfDNMT2 in *P. falciparum*

Real-time quantitative PCR was performed to monitor the relative transcription of *PfDNMT2* to the endogenous *seryl-tRNA synthetase* gene (*stRNA*, PF3D7_0717700) as the control. RNA harvested at an 8 h interval was reverse transcribed using the SuperScript III RNase H reverse transcriptase (Invitrogen). cDNA was PCR amplified with the following primer pairs: *PfDNMT2* Fw/Rv and *stRNA* Fw/Rv (Supplementary Table S1). PCR was done in quadruplicates in 96-well MicroAmp plates in 20 μl (Applied Biosystems), 900 nM of each forward and reverse primer and 2 ng of template. PCR (45 cycles of 95°C for 15 sec and 56°C for 1 min) was performed in an ABI sequence detector 7300 (Applied Biosystems). The detection threshold was set above the mean baseline value for the first 6–15 cycles. The standard deviation of the quotient was calculated according to User Bulletin 2 (Applied Biosystems, http://www.appliedbiosystems.com). Results were visualized as percentages of *PfDNMT2* mRNA relative to the *stRNA* mRNA.

To assess the PfDNMT2 protein expression during the IDC, synchronized parasite cultures were lysed with 0.06% saponin, and the parasite pellet was washed thrice with PBS. Proteins were extracted by incubating parasite pellets with 2% SDS. For subcellular fractionation, parasites were under a freeze-thaw process three times. After centrifugation at 10 000g for 5 min, the supernatants were obtained as the cytoplasmic fractions, the pellets were suspended in 2% SDS, and the supernatants were used as the nuclear fractions after the same centrifugation procedure. Western blot analysis was performed using transgenic parasites with PTP-tagged PfDNMT2 and the anti-Protein C antibodies (1:1000, GenScript). Cytoplasmic and nuclear fractions of rings, trophozoites, and schizonts were equally loaded in SDS-PAGE for detecting PTP-tagged PfDNMT2. To monitor the GFP signal in live PfDNMT2::GFP parasites, images were captured using an epifluorescence microscope (Nikon Eclipse Ni, USA; 100×, 1.4 oil immersion lens) after the nuclei were stained by DAPI at 0.5 μg/ml (Invitrogen).

### TAP purification and MS

TAP was performed using the PTP-tagged PfDNMT2 parasite line according to the published method (55,57,59). Briefly, 10^9^ parasites were lysed in 5 volumes of the hypotonic buffer (10 mM HEPES, pH 7.9, 1.5 mM MgCl_2_, 10 mM KCl, 0.5 mM DTT, 0.5 mM EDTA) at 4°C for 10 min followed by centrifugation for 20 min at 500 × g. The supernatant was harvested as the cytoplasmic fraction. Five volumes of the PA150 buffer (150 mM KCl, 20 mM Tris–HCl, pH 7.7, 3 mM MgCl_2_, 0.5 mM DTT, and 0.1% Tween 20) containing a protease inhibitor cocktail (Roche) were added to the pellets and mixed thoroughly. The lysate was centrifuged for 10 min at 16 000 × g, and the supernatant was harvested as the nuclear fraction. The supernatants were incubated with 100 μl (settled volume) of IgG agarose beads (GE Healthcare) at 4°C for 2 h. The beads were washed twice with PA150 and equilibrated twice with the TEV buffer (150 mM KCl, 20 mM Tris–HCl, pH 7.7, 3 mM MgCl_2_, 0.5 mM EDTA, 1 mM DTT, and 0.1% Tween 20). The beads were incubated with 2 ml of TEV buffer containing 150 U of TEV protease and rotated overnight at 4°C to release the PfDNMT2 and its associated proteins from IgG beads. The supernatant was collected, and the beads were rinsed with another 4 ml of the PC-150 buffer (150 mM KCl, 20 mM Tris–HCl, pH 7.7, 3 mM MgCl_2_, 1 mM CaCl_2_, 0.1% Tween 20). These eluted proteins either proceeded directly to protein identification by LC–MS/MS or were subjected to further purification. For the second step of TAP, 7.5 μl of 1 M CaCl_2_ was added to chelate the EDTA from the TEV buffer, and the combined supernatant was incubated with anti-protein C beads for 2 h at 4°C. The beads were washed four times with PC150 and eluted with the buffer containing 10 nM EGTA/5 mM EDTA.

The proteins from the first elution step were concentrated by Amicon Ultra centrifugal filters (Millipore Sigma) and separated briefly in a 10% Bis–Tris SDS-PAGE gel for 10 min. Proteins in the gel were excised, and in-gel digestion was done as described (64). The digests were analyzed by nanoLC–MS/MS using a Waters NanoAcquity HPLC system interfaced to a Q Exactive Hybrid Quadrupole-Orbitrap Mass Spectrometer (Thermo Scientific). Peptides were loaded on a trapping column and eluted over a 75 μm analytical column at 350 nL/min. MS and MS/MS were performed at 70 000 FWHM and 17 500 FWHM resolutions. The 15 most abundant ions were selected for MS/MS. Parasite proteins were identified by searching the UniProt *P. falciparum* protein database (v01/2014). Data were filtered at 1% protein and 0.2% peptide false discovery rates (FDR) and at least two unique peptides per protein. Mascot DAT files were parsed into the Scaffold software for validation and filtering to create a non-redundant list per sample. Protein data were analyzed by significance Analysis of INTeractome (SAINT) using a probability threshold above 93% and an FDR below 1% (65). The proteomics data were deposited into the ProteomeXchange Consortium via the PRIDE (66) partner repository with the dataset identifier PXD032860 and 10.6019/PXD032860.

### DNA methylation activities of PfDNMT2

The DNMT activity of nuclear extracts and the TAP eluate were measured using the EpiQuik™ DNA Methyltransferase Activity Ultra Kit (Fluorometric) (EpigenTek) following the manufacturer's instructions. Assays were performed in triplicate on 10 μg of nuclear protein extracts from ∼2 × 10^7^ infected RBCs harvested from ∼5% parasitemia culture (∼4 × 10^8^ RBCs from WBC-depleted blood) or ∼200 ng of TAP eluate purified from ∼2.5 × 10^8^ PfDAMT2::PTP parasite-infected RBCs with 100 ng of purified bacterial C5-methyltransferase as the positive control. Nuclear extract from ∼4 × 10^8^ of uninfected RBCs from WBC-depleted blood was used as uninfected RBC control. SG1-1027, a DNMT inhibitor (67), at 10 and 100 μM was used to inhibit DNMT activities in the nuclear extracts. A buffer-only blank was used for background subtraction. DNMT activity was expressed in relative fluorescence units per hour and per mg of proteins (RFU/h/mg).

### *In vitro* drug susceptibility assay

The *in vitro* drug susceptibility assay was performed as previously described (68,69). Briefly, synchronous ring parasites were grown in the presence of different concentrations of SG1-1027 (Cayman chemical company) ranging from 0 to 1500 nM in 96-well plates at 2% hematocrit, 0.5% starting parasitemia and 200 μl of culture media. IC_50_ was determined after 72 h of parasite growth. 20 μl of 10x SYBR green lysis buffer (20 mM Tris–HCl [pH 7.5], 5 mM EDTA, 0.08% Triton X-100, 0.008% saponin in phosphate-buffered saline [PBS; 137 mM NaCl, 2.7 mM KCl, 10 mM Na_2_HPO_4_, 1.8 mM KH_2_PO_4_], 0.2 μl SYBR green I) was added and incubated at room temperature for 30 min in the dark before measuring fluorescence on a TECAN spark multimode plate reader using a 485/535 nm filter. Analysis of the obtained counts plotted against the logarithm of SG1-1027 was performed with GraphPad Prism software 8.0 by fitting a curve with nonlinear regression (sigmoidal dose-response/variable slope equation) to generate IC_50_ values.

### Growth phenotype analysis

Growth phenotypes of the *PfDNMT2* disruptant (*ΔPfDNMT2)*, complementation (episomal expression of *PfDNMT2* under HSP86 promoter in *ΔPfDNMT2*), and overexpression (episomal expression of *PfDNMT2* under HSP86 promoter in 3D7) lines during the IDC were compared with the wild-type (WT) as described (55,59). To measure the cell cycle progression, highly synchronous rings obtained by incubating schizonts with RBCs for 3 h were initiated at 2% parasitemia. Parasite development through the IDC was monitored using Giemsa-stained smears every 2 h over 50 h. Cycle time was determined as the duration between the peak ring parasitemias of two consecutive cycles. After two consecutive rounds of synchronization by sorbitol, synchronous cultures were initiated with 0.5% rings, and parasitemia was monitored daily for seven days without replenishing RBCs to measure parasite proliferation. The number of merozoites produced per schizont was determined from mature segmenters. To delineate individual merozoites, highly segmented schizonts were stained by 4',6-diamidino-2-phenylindole (DAPI). The smears were read under a fluorescence microscope. Three independent biological replications were done for each parasite line.

Merozoite invasion assay was performed as described (70,71). To isolate merozoites for RBC invasion, schizonts were pelleted and resuspended in a culture medium supplemented with 10 μM of the protease inhibitor trans-epoxysuccinyl-l-leucylamido (4-guanidino) butane (E64, Sigma) to prevent rupture. The cultures were incubated for 4 h in standard culture conditions with monitoring for segmentation by Giemsa staining. Merozoites were isolated by mechanically passing schizonts (25% hematocrit) twice through a filter unit with a 1.2 μm pore size FP 30/1.2 CA-S (Whatman) pre-equilibrated with medium and attached to a 3 ml syringe. The filtrate containing free merozoites was divided to mix with pre-warmed (37°C) uninfected RBCs, and the number of merozoites contained therein was counted. The same numbers of purified merozoites from the WT, *ΔPfDNMT2*, complementation, and overexpression lines were mixed with fresh RBCs, and 24 h later, the parasitemia of the culture was determined. The invasion rate was calculated as % RBCs invaded × [(RBCs per μl)/ (merozoites per μl)].

### Parasite transcriptome analysis

RNA-seq was performed to compare the transcriptomes during the IDC among the WT, *ΔPfDNMT2*, complementation, and overexpression lines by the established methods (59). Three replicates of total RNA from parasites at the ring, trophozoite, and schizont stages were harvested with the ZYMO RNA purification kit and used to generate sequencing libraries using the KAPA Stranded mRNA Seq kit for the Illumina sequencing platform according to the manufacturer's protocol (KAPA biosystems). Pooled libraries were sequenced on the Illumina NextSeq 550 instrument using 150 bp paired-end sequencing and dual indexing. Reads from Illumina sequencing were mapped to the *P. falciparum* genome sequence (Genedb v3.1) using HISAT2. The expression levels and differential expression were calculated by FeatureCounts and DESeq2, respectively. The differential expression analysis was performed using a *P*-adjustment value of < 0.01 as the cut-off. RNA-seq data were also normalized by transcripts per million (TPM) to validate the DEseq2 results. Differentially expressed genes between transgenic parasites and WT were selected according to the following criteria: 1) the absolute fold change of TPM higher than 1.5 in biological replicates, and 2) *P*-adjustment from the DEseq lower than 0.1. RNA-Seq data were submitted to NCBI GEO (72) (GSE199368).

### Bisulfite sequencing of tRNA^ASP^ and stability of tRNA^ASP^

Total RNA was isolated from the WT, *ΔPfDNMT2*, complementation, and overexpression lines in the trophozoite stage. The RNA bisulfite conversion was performed using EpiNext 5-mC RNA Bisulfite-Seq Easy Kit (Epigentek) according to the manufacturer's instructions with minor modifications (73). Briefly, 200 ng RNA was treated with a conversion solution, and samples were incubated in a thermal cycler using the following program: 65°C for 5 min, 60°C for 90 min and 4°C on hold. The bisulfite-converted RNA was column cleaned. For reverse transcription, bisulfite-converted total RNA was used as a template for cDNA synthesis with a stem-loop primer (55Pf-AspStem-loop) (Supplementary Table S1) (46). The reverse transcription reaction mixtures were heated at 25°C for 5 min, 50°C for 60 min in a thermal cycler, followed by RNA digestion at 37°C for 10 min according to the manufacturer's instructions. cDNA was purified by MQ binding beads. cDNA coding for Aspartic acid tRNA was enriched by PCR using GoTaq qPCR master mix and Bryt Green dye (Promega) with a deaminated specific forward primer (30Pf-Asp-deaminated-F) and a stem-loop specific reverse primer (30Universal stem-loop R) (Supplementary Table S1). The amplicons were cleaned using the NucleoSpin Gel and PCR clean-up kit (Macherey-Nagel) and quantified using the Qubit® 4.0 Fluorometer and Invitrogen™ Qubit® Quantitation Kit (Thermo Fisher Scientific). The PCR products were cloned using pGEM®-T Easy Vector Systems (Promega) and transformed into JM109 Competent Cells (Promega). The positive clones were confirmed by blue-white screening on X-gal LB plates. At least 50 positive clones per parasite line were selected, and the plasmids were prepared and sequenced using the universal M13 forward primer. The sequenced clones were converted to fasta files, and the presence of methyl cytosines was analyzed using BISMA methylation analysis software (74).

To analyze tRNA^ASP^ level in the *ΔPfDNMT2*, complementation, and overexpression parasite lines compared to the wildtype 3D7 parasite line, total RNA harvested from 30 h trophozoites were reverse transcribed using the SuperScript III RNase H reverse transcriptase (Invitrogen). cDNA were PCR amplified with the following primer pairs: tRNAasp F/R and stRNA Fw/Rv (Supplementary Table S1) in triplicates following a 45-cycle program of 95°C for 15 sec and 42°C for 30 sec) performed on a QuantStudio 3 platform (Applied Biosystems). Results were visualized as fold change of tRNA^ASP^ mRNA relative to the *stRNA* mRNA.

### Microscopic, flow cytometric, and transcriptional analysis of GFP reporter expression

To determine the translational efficiency of Asp-rich proteins, we designed a GFP reporter, where a 6 × Asp tag was introduced by infusion cloning into the *Bam*HI site at the N-terminal of GFP in the modified pBluescript SK with HSP86 5' and pDT 3' UTRs described earlier. The primers used were D6F and D6R (Supplementary Table S1). The 6 × D-GFP expression cassette was then subcloned into pCC4 (55,75) at the *Not*I and *Spe*I sites to produce pCC4/6D-GFP. The DHODH drug cassette was subcloned from pUF1-Cas9 via *Afl*II and *Eco*RI into pCC4/6D-GFP to produce pBlue/6D-GFP. Parasite transfection was done in the WT 3D7 and the *ΔPfDNMT2* parasites. Drug selection was made with 1.5 μM of DSM1 to obtain the transgenic parasites. The synchronized parasites at 36 h trophozoite were stained with 10 μM Hoechst 33342 for 10 min at 37°C and then were observed under an epifluorescence microscope to assess GFP expression (Nikon Eclipse). Totally 100000 cells per sample from the two transgenic lines with 6D-GFP expressed episomally were assayed using the Beckman Coulter MoFlo Astrios flow cytometer. The relative GFP fluorescence intensities in 36 h trophozoites were measured with a Violet laser (395 nm), and bandpass filter of 450/50 nm for detecting Hoechst labeled nucleus, and a blue laser (488 nm) and bandpass filter of 530/30 nm for measuring GFP fluorescence in the cells. The flow cytometry data were analyzed with the FlowJo software. Real-time quantitative PCR with a pair of primers (*6 × D-*GFP-F and *6 × D-*GFP-R) (Supplementary Table S1) was performed to assess the *6 × D-GFP* transcripts levels in the WT and disruptant line at 30 h post-invasion (hpi) relative to mRNA of the endogenous control *stRNA*.

### Statistics

Parasite proliferation rates were compared by two-way ANOVA. DNA methylation levels, DNA methyltransferase activities, cycle lengths, merozoite number in mature schizonts, merozoite invasion rates, and mRNA levels of the reporter and tRNA^Asp^ levels were analyzed by paired *t*-test. Methylation levels at Cytosine 38 of tRNA were compared by paired Wilcoxon test. Spearman correlation was used to compare the transcriptomes among the parasite lines with *PfDNMT2* manipulation and the published transcriptome data of WT parasites.

## RESULTS

### The *P. Falciparum* genome during IDC contains low-level 5mC

**Figure 1.**
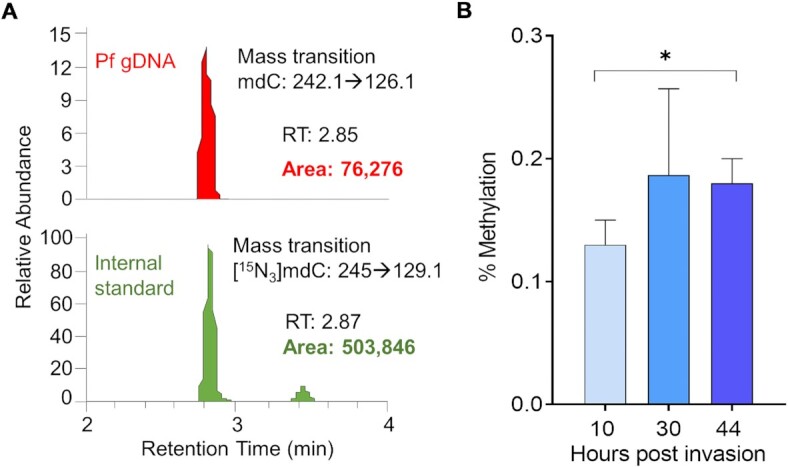
Methylation of cytosine in the *P. falciparum* genome during IDC. (**A**) The presentative diagrams show 5mC LC–MS/MS captured specific spectra using the selected reaction monitoring mode for *P. falciparum* gDNA (red peak) at the trophozoite stage. The mass transition of 5mC was monitored and was shown as a single peak. Internal standards (green) were included in all nucleosides and measured to ensure each analyte's identity. The internal standards have similar retention times (RTs) as the sample deoxynucleotides. The size of the area under the peak indicates the actual mass of methyldeoxycytidine. (**B**) Quantification of 5mC in *P. falciparum*. gDNA prepared from synchronous *P. falciparum* at the ring (10 hpi), trophozoite (30 hpi), and schizont (44 hpi) was digested, and absolute quantification of methyldeoxycytidine was performed on triplicate samples by LC–MS/MS. Methyldeoxycytidine and deoxycytidine concentrations are calculated using the analyte/internal standard peak area ratios and standard curves. The percentage of methyldeoxycytidine is calculated by dividing methyldeoxycytidine concentration by total deoxycytidine concentration (methyldeoxycytidine plus deoxycytidine). *P < 0.05 (paired *t*-test).

To accurately measure the methyldeoxycytidine in the *P. falciparum* genome, we cultured the parasites in WBC-free human blood to eliminate contamination from human DNA. Flow-cytometry only detected ∼5–10 WBCs in one million RBCs after WBCs depletion (Supplementary Figure S1), which only accounts for 0.001–0.002% 5mC of parasite genomic DNA harvested from parasite culture at 5% parasitemia given that the human genome harbors ∼4% 5mC (1). We performed complete digestion of the purified genomic DNA using our improved DNA digestion method (54) followed by LC–MS/MS analysis coupled with a stable-isotopic [*U*-^15^N]-labeled internal standards (53) to obtain an absolute measure of 5mC. Internal standards allowed the retention times of the analytes to be compared within the same run for accurate identification of the analytes. Using three replicates of genomic DNA prepared from highly synchronous *P. falciparum* during the IDC, we quantified that 5mC accounted for 0.13%, 0.19% and 0.18% of total cytosines in the ring, trophozoite, and schizont stages, respectively (Figure 1 and Supplementary Figure S2). The 5mC levels determined in this study are several-fold lower than those (1.16–1.31%) reported by Ponts *et al.* (32) except for the schizont stage (0.36%). Hammam et al. detected very low levels (0.01–0.02%) of 5mC but relatively high levels (0.2–0.4%) of a 5hmC-like molecule in the *P. falciparum* genome (45). However, our analysis with an internal standard of [D3]-labeled 5hmC showed that 5hmC was below the limit of detection and there was no trace of a 5hmC-like peak in the DNA samples from parasites at different stages compared to the positive control of the human genome (Supplementary Figure S2).

### PfDNMT2 is expressed throughout the IDC and localized in the cytoplasm and nucleus

**Figure 2.**
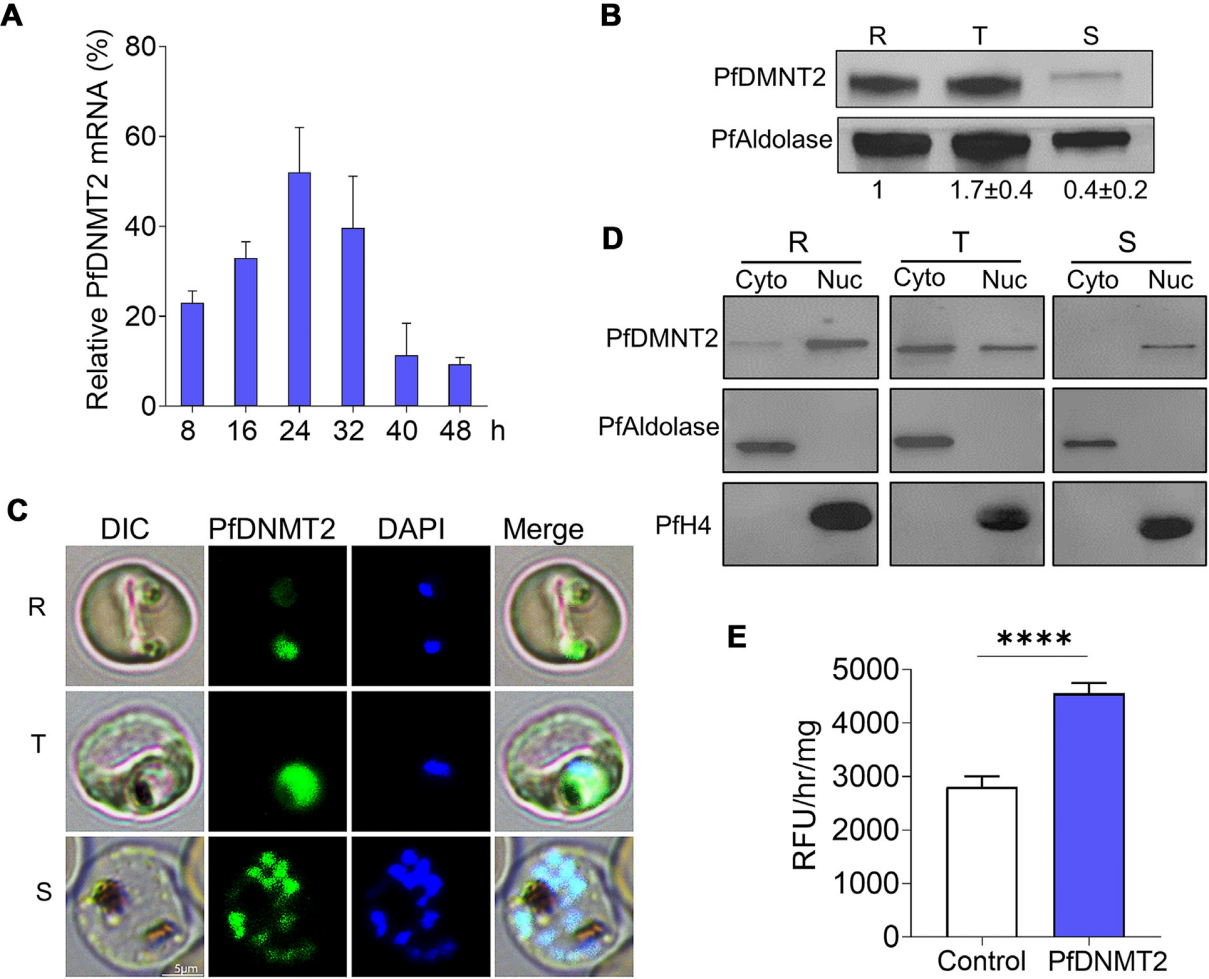
PfDNMT2 expression, localization, and enzymatic activity. (**A**) PfDNMT2 mRNA isolated at the six intraerythrocytic development time points was reverse transcribed (RT) and quantitatively analyzed by qPCR with seryl-tRNA synthetase as a reference. Three biological replicates of RT-qPCR were conducted. (**B**) Western blot showing PfDNMT2-PTP expression levels in the ring (R), trophozoite (T), and schizont stage parasites probed by anti-protein C antibodies (upper panel). PfAldolase was used as a loading control (lower panel). The blots were performed in three biological replicates, and the band intensities were determined using a densitometer. The numbers indicate the PfDNMT2 levels normalized by PfAldolase when the ratio of PfDNMT2/PfAldolase at the ring stage was set as 1. (**C**) Localization of PfDNMT2-GFP at R, T and S stages detected by live imaging, parasite nuclei were stained by DAPI. (**D**) PfDNMT2-PTP expression in the cytoplasm (Cyto) and nuclear (Nuc) fractions at R, T and S stages. Equal amounts of proteins (∼20 μg) were separated by 4–20% SDS PAGE and probed with anti-protein C antibodies (upper panel). Cytoplasmic and nuclear markers were monitored by anti-PfAldolase (middle panel) and anti-PfHistone 4 (lower panel) antibodies, respectively. (**E**) PfDNMT2 purified from PTP-tagged parasites showed high DNA methylation activity by EpiQuik DNMT activity/inhibitor Assay Ultra kit. Assays were performed in biological triplicate with the positive control (50 ng of purified bacterial C5-methyltransferase). DNMT2 activity was expressed in relative fluorescence units per hour and per mg of proteins (RFU/hr/mg). ****P <0.001 (paired *t*-test).

A time-course study by RT-qPCR revealed that *PfDNMT2* was expressed throughout the IDC, with the peak mRNA level detected in the trophozoite stage (Figure 2A), consistent with the expression pattern identified in several transcriptomic studies (36,76,77). To study protein expression and localization, we tagged the endogenous *PfDNMT2* in the 3D7 strain with the GFP or PTP tag (Supplementary Figure S3A) and verified the correct integration of the plasmid into the parasite genome by diagnostic PCR (Supplementary Figure S3B, C). The genetically modified parasites with C-terminal tagging of PfDNMT2 did not show any noticeable change in growth. Western blot analysis of the PTP-tagged parasites with the anti-protein C antibody detected a single protein band at ∼100 kDa, which agreed with the predicted size of the PfDNMT2-PTP fusion protein (Supplementary Figure S3D). The PfDNMT2-PTP protein was detected throughout the IDC, with its relative abundance showing a similar trend as the *PfDNMT2* transcript (Figure 2B). Live imaging of the GFP-tagged PfDNMT2 showed that PfDNMT2-GFP colocalized extensively with the nuclear staining in all the stages of the IDC, while it also had substantial localization in the cytoplasm in the trophozoite stage (Figure 2C). Consistently, cell fractionation of the PfDNMT2::PTP parasites followed by western blots confirmed this predominantly nuclear localization pattern of PfDNMT2-GFP in rings and schizonts, whereas PfDNMT2-PTP was detected in both the nuclear and cytoplasmic fractions in trophozoites (Figure 2D).

### PfDNMT2 has robust DNA methyltransferase activity

Previous studies showed that DNMT2 orthologs possessed weak or no DNA methyltransferase activities (14,17–20). The DNMT activity of the recombinant truncated PfDNMT2 expressed in bacteria was either weak (32) or undetectable (46) from *in vitro* assays. We speculated that the poor enzyme activities of the recombinant PfDNMT2 might be due to improper folding, or PfDNMT2 may need to be in its native protein complex for enzyme activity. To address this problem, we purified ∼200 ng of PfDNMT2-PTP from the nuclear extracts of ∼2.5 × 10^8^ synchronized trophozoites under native conditions using the TAP procedure (57–59). We measured the DNMT activity using 200 ng of the TAP eluate with a DNA methyltransferase activity kit. Surprisingly, the TAP eluate showed higher DNMT activity (∼4600 RFU/mg) than the positive control (∼2860 RFU/mg), 100 ng of purified bacterial C5-methyltransferase M.SssI (Figure 2E). M.SssI contains all ten conserved motifs of C5-DNMTs and has been used as a positive control because it has the same specificity as the mammalian DNMTs (78,79). When 10 μg of the crude nuclear extracts from ∼2 × 10^7^ parasites was used in the enzyme assays, the DNMT activity was relatively low at ∼610 RFU/mg, consistent with a previous study (32). Western blotting showed that the abundance of PfDNMT2 in 200 ng TAP eluate was substantially higher than that from 10 μg of the crude nuclear extracts, suggesting that a higher abundance of PfDNMT2 in TAP eluate contributed to its higher DNMT activities (Supplementary Figure S3E).

### PfDNMT2 associates with distinct proteins in the nucleus and cytoplasm of parasites

We next wanted to determine the proteins associated with PfDNMT2. To capture proteins that may be transiently or weakly associated with PfDNMT2, we performed IPs with only the IgG Sepharose column using lysates from the nuclear and cytoplasmic fractions of trophozoites. Bound proteins were released from the column by TEV proteinase digestion and subjected to protein identification by LC–MS/MS. SAINT identified 16 proteins from the nuclear fraction in all three biological replicates, including an AP2 transcription factor (PF3D7_1239200), the SPT16 subunit of the putative FACT complex, a protein kinase, and two RNA-binding proteins (Table 1, Supplementary Table S2). In the cytoplasmic fraction, SAINT identified 13 proteins, including the eukaryotic translation initiation factor eIF2A, an RNA binding protein, a Zinc finger protein, two aspartic proteases (plasmepsin I and III), a ubiquitin-like protein, and two exported proteins (Table 1, Supplementary Table S2). Only three identified proteins were shared in both fractions, indicating that PfDNMT2 associates with distinct protein partners to potentially regulate transcription in the nucleus and protein translation and degradation in the cytoplasm.

**Table 1.** Identification of PfDNMT2-associated proteins. IPs by IgG beads followed by TEV protease digestion identified proteins interacting with PfDNMT2. Proteins were pulled down from the nuclear and cytoplasmic extracts of the PfDNMT2-PTP parasite line at the trophozoite stage and identified by LC–MS/MS. ^#^: SC: spectra count. *: R1, R2, and R3 denote three replicates of the experiment. Protein names in bold are AP2 TF or RNA binding proteins

Gene ID	Protein name	SC^#^ PfDNMT2-PTP R1/R2/R3*	SC Control IPs R1/R2/R3	Average probability R1/R2/R3	FDR
**Nucleus**					
PF3D7_0727300	DNA (cytosine-5)-methyltransferase	94|48|49	0|0|0	1.00	0
PF3D7_0323800	Conserved Plasmodium protein	12|10|7	0|1|0	0.99	0.0032
PF3D7_1022000	**RNA-binding protein, putative**	26|12|15	3|2|3	0.98	0.0071
PF3D7_1015600	Heat shock protein 60	117|73|74	14|25|11	0.98	0.0094
PF3D7_0716300	Conserved protein	6|5|5	0|0|0	0.98	0.0114
PF3D7_1426000	60S ribosomal protein L21	6|5|6	0|0|0	0.98	0.0129
PF3D7_1239200	**AP2 domain transcription factor, putative**	78|23|25	11|7|3	0.98	0.0142
PF3D7_0517400	FACT complex subunit SPT16, putative	13|7|4	0|0|0	0.98	0.0154
PF3D7_0307700	Conserved Plasmodium protein	21|6|9	1|2|0	0.96	0.0179
PF3D7_0926100	Protein kinase, putative	30|9|8	2|3|0	0.96	0.0206
PF3D7_0817300	Conserved Plasmodium protein	20|7|13	1|3|0	0.95	0.0231
PF3D7_0510100	Conserved protein	48|18|18	6|11|2	0.94	0.0284
PF3D7_1237500	Conserved Plasmodium protein	7|4|4	0|0|0	0.94	0.0284
PF3D7_1419400	Conserved Plasmodium membrane protein	27|9|9	2|2|1	0.94	0.0324
PF3D7_1419700	Conserved Plasmodium protein	6|4|5	0|1|0	0.94	0.0305
PF3D7_0927600	**RNA-binding protein, putative**	6|5|6	1|0|0	0.93	0.0344
**Cytoplasm**					
PF3D7_0727300	DNA (cytosine-5)-methyltransferase	82|70	0|0	1.00	0
PF3D7_0510100	Conserved protein	43|37	18|19	1.00	0
PF3D7_1036900	Conserved Plasmodium protein	22|25	12|6	1.00	0
PF3D7_0906600	Zinc finger protein, putative	32|26	15|13	1.00	0.0008
PF3D7_1022000	**RNA-binding protein, putative**	17|18	6|2	1.00	0.0008
PF3D7_1419400	Conserved Plasmodium membrane protein	38|26	22|14	1.00	0.0013
PF3D7_1132000	ubiquitin-like protein, putative	22|11	4|3	1.00	0.0018
PF3D7_1019000	Eukaryotic translation initiation factor subunit eIF2A	27|18	13|7	1.00	0.0024
PF3D7_0730900	EMP1-trafficking protein	9|11	1|0	0.99	0.003
PF3D7_1407900	Plasmepsin I	23|21	8|9	0.99	0.0041
PF3D7_1408100	Plasmepsin III	24|20	10|9	0.99	0.0052
PF3D7_0301600	Plasmodium exported protein (hyp1)	15|15	5|5	0.97	0.007
PF3D7_1008900	Adenylate kinase	9|6	0|2	0.97	0.0087

^#^: SC: spectra count. *: R1, R2, and R3 denote three replicates of the experiment. Protein names in bold are AP2 TF or RNA binding proteins.

### *PfDNMT2* disruption enhances parasite proliferation

**Figure 3.**
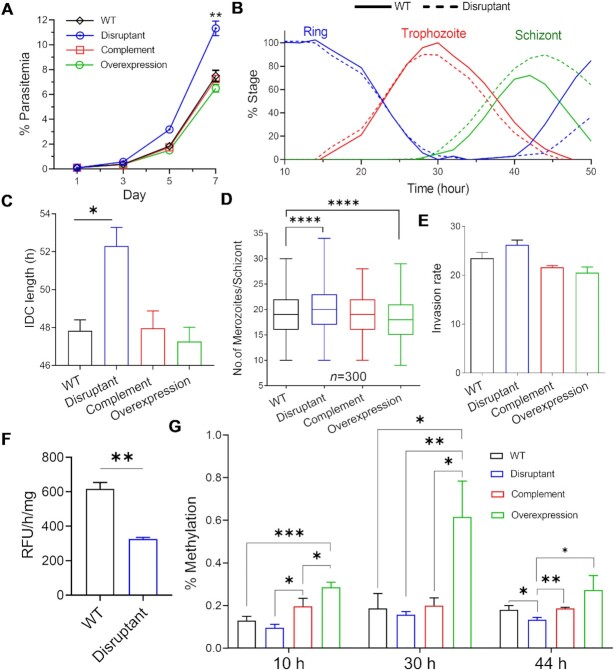
Manipulation of *PfDNMT2* causes alteration in growth and DNA methylation. (**A**) *In vitro* growth curves of the *PfDNMT2* disruptant, complementation, and overexpression lines compared to the WT. Means ± SDs are shown from six replicates of independent experiments conducted by two persons. All parasites started at 0.1% ring-stage parasitemia, and parasitemia was monitored every day using Giemsa-stained smears. ** indicates *P <*0.01 (ANOVA) on day 7. (**B**) A growth course monitoring highly synchronous rings throughout the IDC. Blood smears were collected every 2 h, and the percentage of each stage was counted (*y*-axis). PfDNMT2 disruption showed an extended schizont stage. (**C**) The duration of the IDC. *, *P* < 0.05 (*t*-test paired). (**D**) A box-whisker plot showing the number of merozoites produced per schizont in different parasite lines. The plot shows the median (line), 25th and 75th percentile (box), and the minimum and maximum values (whiskers) obtained from counting at least 300 schizonts. ****, *P* < 0.0001 (paired *t*-test). (**E**) The merozoite invasion rates determined by the invasion assay. Equal numbers of invasive merozoites isolated from segmented schizonts were mixed with fresh RBCs, and 24 h later, the invasion rates of WT and disruptant lines were calculated as %RBCs invaded × [(RBCs per μl)/(merozoites per μl)]. (**F**) Enzyme activity assays performed in triplicate with 10 μg of nuclear protein extracts. *PfDNMT2* disruption caused a ∼50% decrease in DNA methylation activity from the nuclear extract compared to WT. DNA methylation activity was measured by EpiQuik DNMT activity/inhibitor Assay kit. RFU/hr/mg denotes units of fluorescence per hour per mg of proteins. **P < 0.01 (paired *t*-test). (**G**) Absolute quantification of gDNA methyldeoxycytidine conducted by LC–MS/MS on triplicate samples from four parasite lines. PfDNMT2 overexpression increased methylation at all IDC stages. Means ± SDs are shown. Nonsignificant tests are not indicated. *, ** and *** denote *P* < 0.05, 0.01 and 0.001, respectively (paired *t*-test).

To investigate the function of PfDNMT2, we sought to disrupt this gene by single cross-over recombination (Supplementary Figure S4A, B). PfDNMT2 is dispensable for the IDC (80), and its disruption was confirmed by the Southern blot (Supplementary Figure S4C). Compared to the WT parasite, the *ΔPfDNMT2* parasite displayed an increased proliferation rate (Figure 3A). Starting from 0.5% ring-stage parasitemia, the *ΔPfDNMT2* parasite reached 11.3% on day 7 as compared to 7.5% for the WT parasite (*P <* 0.01). To understand this growth phenotype, we examined the parasite development progression through different IDC stages. We found that the schizont stage of the *ΔPfDNMT2* parasite was ∼3 h longer than the WT (Figure 3B), significantly extending the duration of the IDC to ∼51 h compared to ∼48 h in the WT (Figure 3C, *P* < 0.05, paired *t*-test). We then determined if the increased proliferation rate of the *ΔPfDNMT2* parasite resulted from enhanced efficiency of parasite multiplication or merozoite invasion. We found that the *ΔPfDNMT2* mature schizonts contained significantly more merozoites than the WT parasite (Figure 3D, *P* < 0.0001, paired *t*-test). Merozoites isolated from the *ΔPfDNMT2* parasite also showed a higher RBC invasion rate (Figure 3E). However, this higher rate did not reach statistical significance (*P* > 0.05, paired *t*-test). These data collectively indicate that the higher proliferation rate of the *ΔPfDNMT2* parasite was mainly due to the higher number of merozoites produced during the longer schizont development and partially due to higher invasion capacity.

To corroborate that the phenotypic change of the *ΔPfDNMT2* was caused by *PfDNMT2* gene disruption, we performed genetic complementation and overexpression studies by episomally expressing PfDNMT2-GFP in the *ΔPfDNMT2* and 3D7 parasites, respectively (Supplementary Figure S5A). The episomally expressed PfDNMT2-GFP showed a similar localization pattern as observed for the endogenously tagged PfDNMT2-GFP (Supplementary Figure S5B). Episomal expression of PfDNMT2 in the *ΔPfDNMT2* parasite fully restored the parasite growth phenotypes, including the proliferation rate, the IDC progression, merozoite production, and merozoite invasion efficiency (Figure 3A, C–E, Supplementary Figure S5C). Moreover, overexpression of PfDNMT2 in 3D7 further reversed the growth phenotype compared to the WT 3D7, showing a significantly smaller number of merozoites per mature schizont (Figure 3D, *P* < 0.0001, paired *t*-test), as well as a slightly lower proliferation rate, shorter IDC length, and lower merozoite invasion efficiency (Figure 3A, C, E, Supplementary Figure S5D, *P* > 0.05, paired *t*-test). Taken together, these PfDNMT2 genetic manipulation studies demonstrated that PfDNMT2 is involved in parasite development, proliferation, and invasion.

### Alteration of DNA methylation upon *PfDNMT2* manipulation

To further corroborate the PfDNMT2’s DNA methylase activity, we first measured the DNA methylation activity in 10 μg of nuclear extract from ∼2 × 10^7^ of *ΔPfDNMT2* parasites, which exhibited a ∼50% reduction compared to the WT parasite (Figure 3F, Supplementary Figure S6A). The nuclear extracts from the same numbers of WBC-depleted RBCs for harvesting ∼2 × 10^7^ of *ΔPfDNMT2* parasites harbored only ∼3% of DNMT activity in nuclear extracts of WT parasites, indicating that the DNMT activity in the nuclear extract of the *ΔPfDNMT2* parasites is not from contaminating WBC from the asexual stage blood culture (Supplementary Figure S6A). This finding is in line with the similar levels of reduction in DNA methylase activity observed in the *Drosophila* embryos and *D. discoideum* after *DNMT2* gene deletion, suggesting that there is probably another DNMT in these ‘DNMT2 only’ organisms (81,82). The DNMT activity in *ΔPfDNMT2* parasites was substantially inhibited by SGI-1027, a DNMT inhibitor (67), corroborating that *P. falciparum* harbors another DNMT (Supplementary Figure S6B).

We next performed absolute measurement of methyldeoxycytidine by LC–MS using parasite genomic DNA isolated from the *ΔPfDNMT2*, complementation, and overexpression parasite lines. Compared to the WT, the 5mC levels in the *ΔPfDNMT2* parasite decreased by ∼25.6% (0.13% versus 0.09%), 16% (0.19% versus 0.15%) and 26% (0.18% versus 0.13%) in the ring, trophozoite and schizont stages, respectively (Figure 3G). Notably, the level of 5mC reduction in the schizont stage was similar to the 30% reduction reported earlier in two conditional *PfDNMT2* knockout clones (33). The smaller scale of reduction in 5mC (16–26%) compared to the reduction in DNMT activity (50%) in *ΔPfDNMT2* parasites could be a compensation effect from an unknown DNMT. Upon *PfDNMT2* complementation, DNA methylation levels reached ∼146%, 100% and 103% of the WT values in the ring, trophozoite, and schizont stages, respectively. Compared to the WT, overexpression of PfDNMT2 in 3D7 further increased the methylation levels by ∼115%, ∼221% and ∼51% in the ring, trophozoite, and schizont stages, respectively (Figure 3G). These results reaffirmed that *PfDNMT2* disruption substantially reduced DNA methylation in *P. falciparum*, which was restored by complementation and increased by overexpression of PfDNMT2. Additionally, *in vivo* drug susceptibility assays showed that *ΔPfDNMT2* parasites became more sensitive to SGI-1027 (IC_50_: 38.1 ± 0.96 nM), in contrast, *PfDNMT2* complementation and overexpression parasite lines became more resistant to SGI-1027 (IC_50_: 54.10 ± 0.32 and 55.81 ± 0.86 nM) compared to the WT (IC_50_: 49.9 ± 0.93 nM) (Supplementary Figure S6C, D). Collectively, these results further demonstrated that PfDNMT2 is a bona fide DNMT.

### *PfDNMT2* disruption causes specific transcriptional changes

**Figure 4.**
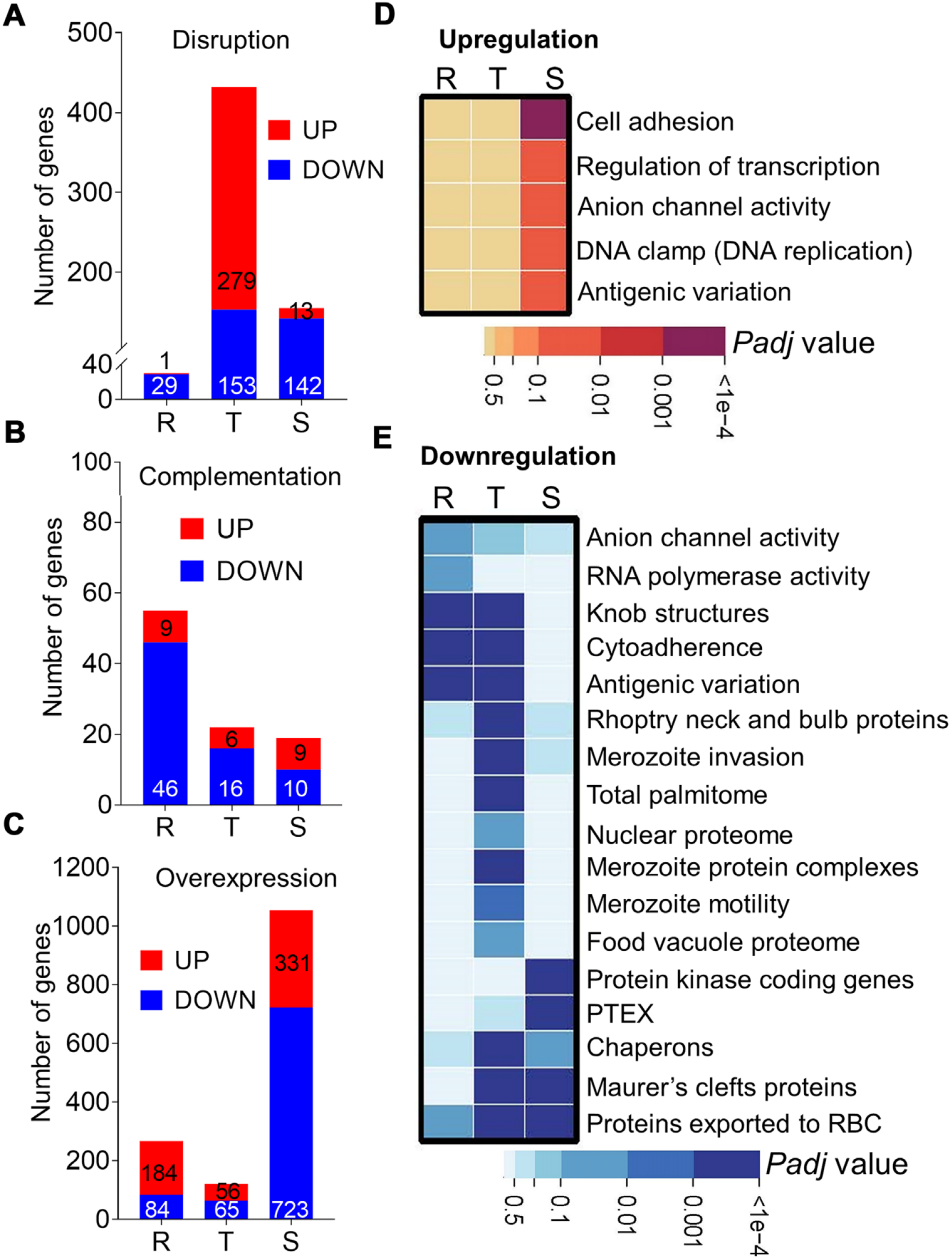
Transcriptomic changes after genetic manipulation of *PfDNMT2*. (A-C) The number of genes with altered expression at the ring (R), trophozoite (T) and schizont (S) stages in the *PfDNMT2* disruption (**A**), complementation (**B**) and overexpression (**C**) lines compared to the WT 3D7. The up-and-down-regulated genes are labeled in red and blue, respectively. Cut-offs are FDR 0.1 and fold change 1.5. (D, E) GO enrichment analysis of the upregulated (**D**) and downregulated (**E**) genes in Δ*PfDNMT2* compared to 3D7 at the ring (R), trophozoite (T) and schizont (S) stages. The color bars show the scales of adjusted *P* values.

To assess the effect of altered DNA methylation on gene expression, we compared the transcriptomes of the WT and *PfDNMT2* manipulation parasite lines (disruption, complementation, and overexpression) by RNA-seq. RNA was prepared from tightly synchronized parasites at 10, 30, and 40 hpi in three biological replicates except for *ΔPfDNMT2* at the schizont stage, which was prepared at 43 hpi to compensate for the delay in development compared to the WT (Figure 3B, C). This sampling scheme well matched the time-series transcriptome data of WT parasites, as shown with the high Spearman correlations (Supplementary Figure S7) (83). Using FDR of 0.1 and fold change of 1.5 as the significance threshold, we identified that *PfDNMT2* disruption resulted in the downregulation of 29, 153 and 142 genes and up-regulation of 1, 279 and 13 genes in the ring, trophozoite, and schizont stages, respectively (Figure 4A–C, Supplementary Figure S8, Supplementary Table S3). Gene ontology (GO) enrichment analysis revealed that the processes such as invasion and protein export were downregulated in the trophozoite stage, whereas DNA replication was upregulated at the schizont stage, explaining the ∼3 h extension of the schizont stage and more progeny (merozoite) produced in the *ΔPfDNMT2* schizont (Figure 4D, E). Upon complementation of *ΔPfDNMT2*, transcription was restored to the WT levels with only 1% (55), 0.4% (22) and 0.36% (19) of genes differentially expressed in the ring, trophozoite, and schizont stages, respectively (Figure 4A-C, Supplementary Figure S8, Supplementary Table S3). Conversely, overexpression of *PfDNMT2* in 3D7 led to 5% (268), 2.3% (121) and 20.3% (1054) of genes differentially expressed in the ring, trophozoite, and schizont stages, respectively, indicating that *PfDNMT2* overexpression caused more profound disturbance of gene expression throughout the IDC, especially in the schizont stage (Figure 4A–C, Supplementary Figure S8, Supplementary Table S3). GO enrichment analysis showed that invasion-related transcripts were downregulated in schizonts of the PfDNMT2 overexpression line, consistent with its reduced invasion phenotype (Supplementary Figure S9, Figure 3E).

### *PfDNMT2* disruption reduces tRNA^ASP^ C38 methylation

**Figure 5.**
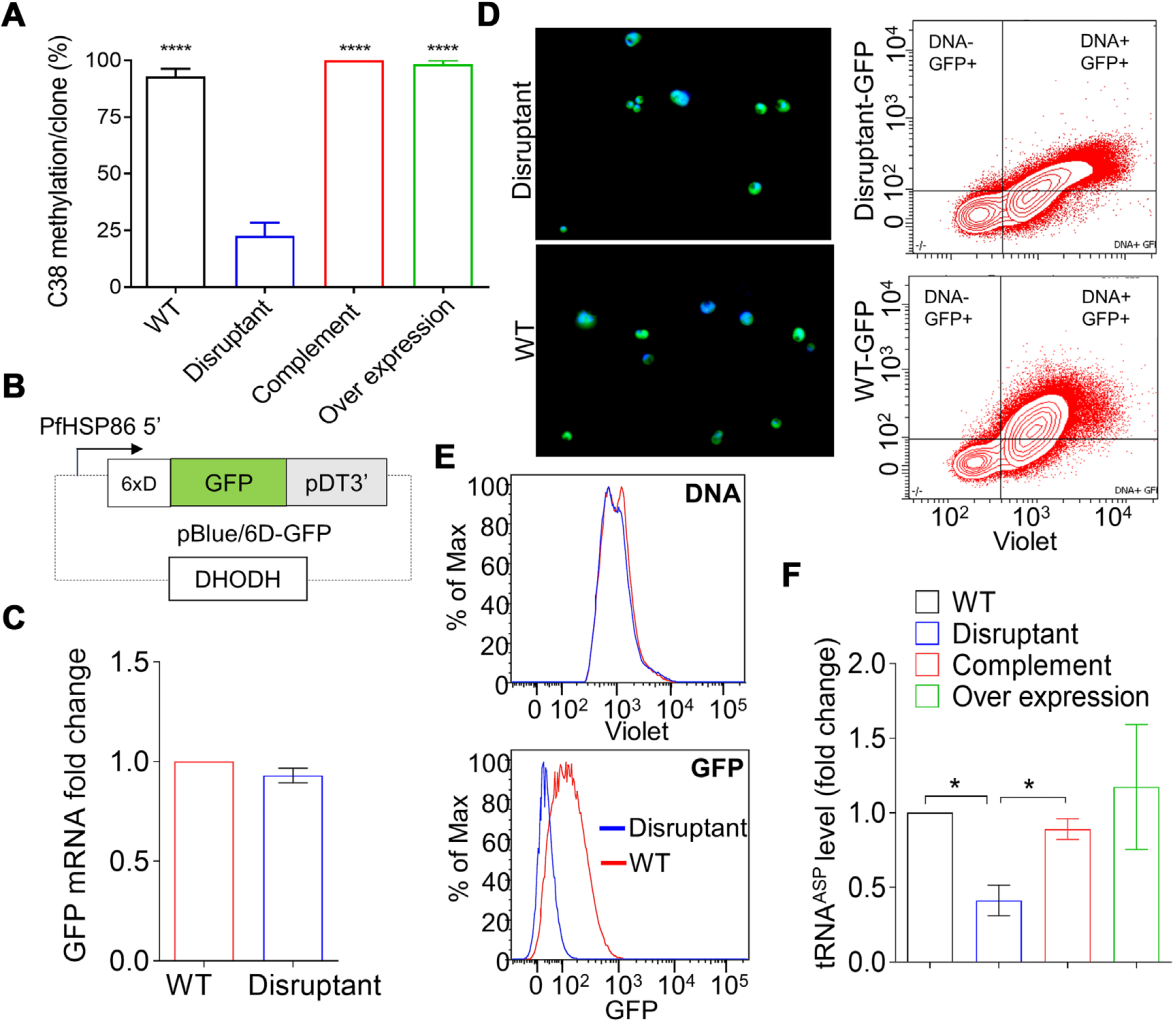
Decreased tRNA^ASP^ methylation reduced poly-aspartate protein expression. (**A**) The bar graph shows the percentage of methylated tRNA^ASP^ gene at C38 identified using RNA bisulfite amplicon sequencing from multiple clones (WT *n*= 54, complement *n*= 57, overexpression *n*= 56, disruptant *n*= 51). **** indicates a significant difference from the disruptant (*P* < 0.0001, paired Wilcoxon test). (**B**) A schematic diagram of the reporter construct showing GFP with N-terminal insertion of 6 × D. DHODH; DSM1 selectable marker. **(C)** RT-PCR analysis of the reporter mRNA. mRNA isolated from 6D-GFP expressing trophozoites of transgenic 3D7 (named WT here) and disruptant lines was quantitatively analyzed by RT-PCR with seryl-tRNA synthetase gene as a reference. Data from three biological replicates represent the fold change of 6 × D -GFP mRNA relative to the constitutively expressed seryl-tRNA synthetase gene. *P =*0.12 (unpaired two-tailed *t* test). (**D**) Analysis of GFP expression of parasites with episomal expressing of the 6 × D-GFP by live imaging (left panels) and flow cytometry (right panels). Infected red blood cells (iRBC) were stained with a DNA-specific dye Hoechst 33258 to distinguish iRBCs from uninfected RBCs. GFP and Hoechst fluorescence intensities of synchronous trophozoite stage in WT 3D7 and disruptant lines were determined by flow cytometry. The dot plots in the right panels show the fluorescence intensities of the Hoechst staining (DNA content; x-axis) and GFP (y-axis). (**E**) Histograms showing the DNA contents of equal numbers of parasites (left panel) and GFP fluorescent intensity (right panel) of transgenic 3D7 (WT) (red) and disruptant (in blue) parasite lines with the episomal expression of the reporter construct. (**F**) RT-qPCR analysis of tRNA^ASP^ level. Total RNA isolated from trophozoites of WT, Δ*PfDNMT2*, *PfDNMT2* complementation and overexpression lines was used for quantitative analyses with seryl-tRNA synthetase gene as a reference. Data from three biological replicates represent the fold change of tRNA^ASP^ relative to the constitutively expressed seryl-tRNA synthetase gene. *P < 0.05 (unpaired two-tailed *t*-test).

Previous studies reported that the recombinant truncated PfDNMT2 had *in vitro* tRNA methylation activity (46), and conditional knockout of *PfDNMT2* resulted in a reduction of methylation at C38 of tRNA^ASP^ (33). To confirm that PfDNMT2 is the specific tRNA methyltransferase for C38 of tRNA^ASP^, we determined the methylation status of tRNA^ASP^ in the WT, *ΔPfDNMT2*, complementation, and overexpression lines by RNA bisulfite sequencing (73) in trophozoite stage when PfDNMT2 had peak expression and cytoplasmic location. We performed deamination-sensitive PCR to enrich bisulfite-converted tRNA^ASP^ from these parasite lines followed by cloning and sequencing the PCR products (Supplementary Figure S10). Overall, *PfDNMT2* disruption significantly decreased C38 methylation, whereas complementation restored C38 methylation to the WT level (Figure 5A). PfDNMT2 overexpression maintained a methylation level similar to the WT. The tRNA^ASP^ gene has seven sites in the CpG context, and C38 showed 96.3% methylation in the WT (Supplementary Figure S11). *PfDNMT2* disruption resulted in a 72% reduction in C38 methylation. Other sites not known as PfDNMT2 substrates did not show significant alterations in methylation upon *PfDNMT2* manipulation (84,85). These results confirmed the direct role of PfDNMT2 in C38 methylation.

### Loss of tRNA^ASP^ methylation decreases tRNA^ASP^ and poly-aspartate protein expression

tRNA cytosine methylation has been previously highlighted in protein synthesis (13,84–86), notably in proteomes of organisms with substantial proportions of Asp residues (46). A recent study showed that disruption of *PfDNMT2* resulted in the decrease of Asp-rich proteins in the parasite (33). To confirm that *PfDNMT2* affects the translational efficiency of Asp-rich proteins, we examined whether reduced tRNA^ASP^ C38 methylation conferred a defect in the synthesis of Asp-rich proteins using a GFP reporter gene with six aspartate residues inserted in its N-terminus, consistent with the reduction of poly-Asp-tagged reporter proteins after DNMT2 KO in murine embryonic fibroblast cells (84) (Figure 5B). The GFP reporter construct was episomally expressed in the WT and *ΔPfDNMT2* parasites and analyzed by fluorescent microscopy and flow cytometry. Although the GFP transcript levels in the two parasite lines were similar (Figure 5C), quantitative flow cytometry detected a significant decrease in relative GFP fluorescent intensity in the *ΔPfDNMT2* parasite compared to the WT control (Figure 5D, E). These data provide direct evidence that loss of tRNA^ASP^ methylation reduces the translation efficiency of Asp-rich proteins.

Earlier studies indicated that C38 methylation of tRNA^ASP^ by DNMT2 protected the cleavage of tRNA^ASP^ in *Drosophila* (87) and double KO of DNMT2 and Nsun2, which generates 5mC at other tRNA positions, caused the reduction of tRNA levels in mice (86). To investigate whether disruption of *PfDNMT2* led to any change in tRNA^ASP^ abundance, we measured the level of tRNA^ASP^ in *ΔPfDNMT2*, *PfDNMT2* complementation, and overexpression parasite lines compared to the WT control. As shown in Figure 5F, disruption of *PfDNMT2* resulted in a significant reduction in tRNA^ASP^ level whereas complementation and overexpression of PfDNMT2 restored and escalated the tRNA level in the parasite, respectively, indicating that PfDNMT2 regulates tRNA^ASP^ stability probably by protecting it from cleavage via methylation.

## DISCUSSION

In *P. falciparum*, the biological functions of DNMT2, the most widely conserved gene of the DNMT family in eukaryotes, are presently contentious with tRNA and potentially DNA methyltransferase activities. We have comprehensively interrogated the PfDNMT2 enzyme activities on genomic DNA and tRNA throughout the IDC in a panel of transgenic parasites through a series of complementary experiments, including accurate MS, *in vitro* DNMT activity, subcellular localization, interactome, genetic manipulation, tRNA methylation and levels, and reporter gene translation. We provide evidence that PfDNMT2 possesses both DNA and tRNA methyltransferase activities and plays important roles in the transcriptional regulation of specific biological pathways such as DNA replication and invasion in the nucleus, and protein translation in the cytoplasm.

An earlier study detected relatively high levels (1.16–1.31%) of 5mC in the *P. falciparum* genome (32), whereas a follow-up study identified extremely low levels of 5mC (0.01–0.02%) in the parasite genome (45), leading to the speculation that the relatively high 5mC contents reported earlier were probably due to the method used without internal control or human DNA contamination during parasite culture. To solve this discrepancy, we developed a protocol to incorporate steps to eliminate potential contamination of human DNA from WBCs in the blood, improve DNA digestion, and precisely identify the target analytes by including stable isotope-labeled internal standards in the same LC–MS/MS runs. Including internal standards in the same MS/MS runs improves the accuracy of the quantitative assay, especially when there are significant concentration differences and variations between the analytes. We found that 0.13–0.19% of the total cytosines in the parasite genome were methylated during the IDC (Figure 1) and these 5mC levels are similar to the levels in other ‘DNMT2 only’ organisms (27,82). The study that identified 5mC at extremely low levels also detected relatively high levels of 5mC-like and 5hmC-like peaks at longer retention times compared to 5hmC and 5mC peaks in the parasite genome (45). In contrast, our analyses did not reveal any trace of 5mC-like and 5hmC-like peaks (Figure 1 and Supplementary Figure S2). Previous studies showed that more than one peak at different retention times indicated nucleoside dimers, salt adduct byproducts, or oligomers existed in the incompletely digested DNA (88,89). Therefore, we speculate that the appearance of 5hmC-like and 5mC-like peaks could be derived from the incomplete digestion of genomic DNA or salt adduct byproducts.

Our complementary approaches conclusively attest that PfDNMT2 is the methylase catalyzing the cytosine methylation in the parasite genome. First, the native PfDNMT2 enzyme (complex) purified from the parasite using a TAP procedure displayed high-level DNA methylase activity, rivaling a bacterial DNA methylase included as the positive control. This result is in stark contrast to the insignificant DNA methylase activity of recombinant truncated PfDNMT2 expressed in bacteria, which might have improperly folded, missed essential DNA methylase motifs (32), or lacked the endogenous complex (90). Previous reports showed that DNA methylation activities of DNMT2 from malaria parasites (32,46) and other ‘DNMT2 only’ organisms (14,17–20) were not detectable or at low levels, which led to suspicion that DNMT2 may not have real DNA methylation activity. However, by using TAP, we enriched PfDNMT2 at a higher concentration from parasite nuclei and defined for the first time that PfDNMT2 has strong DNA methylation activity like the positive control. Second, *PfDNMT2* disruption led to a significant decrease in the DNA methylase activity of the nuclear extract and a reduction in the global DNA methylation level in parasite genomic DNA compared to the WT. Third, *PfDNMT2* complementation restored the 5mC levels, while *PfDNMT2* overexpression further elevated parasite genomic DNA 5mC level up to 221% of that in the WT parasite (Figure 3). The shift in IC_50_ values after *PfDNMT2* manipulation further confirms that PfDNMT2 is an authentic DNMT. Finally, the DNMT activity of PfDNMT2 is also consistent with its predominant nuclear localization in the ring and schizont stages (Figure 2). These data demonstrated the presence of relatively low levels of 5mC modification in the *P. falciparum* genome and incriminated PfDNMT2 as the DNA methylase. On the other hand, *PfDNMT2* disruption resulted in the reductions of DNA methylation activities and 5mC levels by only 50% and 16–26%, respectively, and SGI-1027 significantly reduced the DNA methylation activity in the *ΔPfDNMT2* nuclei, suggesting that there might be an unidentified DNMT in the parasites like other ‘DNMT2 only’ organisms (81,82).

Does the low-level 5mC in the *P. falciparum* genome have any functional significance? Our transcriptome analysis detected extensive transcription perturbance from *PfDNMT2* disruption, with the most significant effect on the trophozoite stage coincidental with peak PfDNMT2 expression (Figure 4). While the transcriptomic changes resulting from *PfDNMT2* disruption were fully rescued by complementation, *PfDNMT2* overexpression led to a more substantial disturbance of the transcriptome. The upregulated genes upon *PfDNMT2* disruption are enriched in transcription regulation and DNA replication, which may be responsible for the extended schizont stage and enhanced merozoite production, underlying the increased proliferation of the *ΔPfDNMT2*. The association of PfDNMT2 with the AP2-TF (PF3D7_1239200) indicates that PfDNMT2 is involved in transcriptional regulation. These data, together with the predominant nuclear localization of PfDNMT2 in ring and schizont stages, demonstrate PfDNMT2’s involvement in the transcription regulation of specific cellular pathways. Recent studies showed that DNMT2 has a novel function in RNA-mediated epigenetic heredity in mice (91–93) and whether PfDNMT2 participates in this mechanism needs more investigation.

We observed a marked dual localization pattern of PfDNMT2 in the nucleus and cytoplasm, particularly in trophozoites, when protein synthesis is most active (94,95). Although we could not speculate on the cytoplasmic functions of PfDNMT2 based only on its interactome, its action on epitranscriptomic tRNA methylation will probably influence protein translation. Similar to the observation from a recent study (33), *PfDNMT2* disruption led to a ∼72% reduction in tRNA^ASP^ methylation at position C38, which was restored to the WT levels by genetic complementation. Using a reporter construct incorporating poly-d, we provided a biological implication that the deficit in tRNA^ASP^ methylation is directly linked to the reduced translation of the poly-d-containing reporter protein. C38 methylation of tRNA^Asp^ was fully restored after PfDNMT2 complementation, which further confirmed that PfDNMT2 was responsible for this methylation. tRNA^Asp^ level in *ΔPfDNMT2* was significantly reduced whereas complementation and overexpression of PfDNMT2 restored and escalated the tRNA^Asp^ levels, respectively, suggesting that PfDNMT2 regulates tRNA^ASP^ stability probably by protecting it from cleavage via methylation. Further characterization of RNA binding proteins identified from PfDNMT2 interactome opens a window to study the underlying mechanisms.

In summary, we provide solid evidence of the presence of low-level 5mC in the *P. falciparum* genome during its asexual development in the RBCs. Through a series of experiments, we conclusively showed that PfDNMT2 was responsible for depositing methyl groups on genomic DNA and C38 of tRNA^ASP^, which have functional significance in transcriptional and translational regulation, respectively. The regulation of general biological processes such as DNA replication and parasite-specific biological pathways such as invasion indicates that *P. falciparum*, a DNMT2-only organism, has evolved to designate DNMT2 novel regulatory functions.

## DATA AVAILABILITY

The proteomics data were deposited into the ProteomeXchange Consortium via the PRIDE partner repository with the dataset identifier PXD032860 and 10.6019/PXD032860. RNA-Seq data were submitted to NCBI GEO (GSE199368).

## Supplementary Material

gkad248_Supplemental_Files
