# Supplementary material for: Characterization of the dual role of Plasmodium falciparum DNA methyltransferase in regulating transcription and translation
Source: Nucleic Acids Res. 2023 Apr 7;51(8):3918–33. doi: 10.1093/nar/gkad248 (PMC10164579; doi:10.1093/nar/gkad248)
Supplement: gkad248_Supplemental_Files [file gkad248_supplemental_files.zip › Supplementary Figures.pdf]

Figure S1

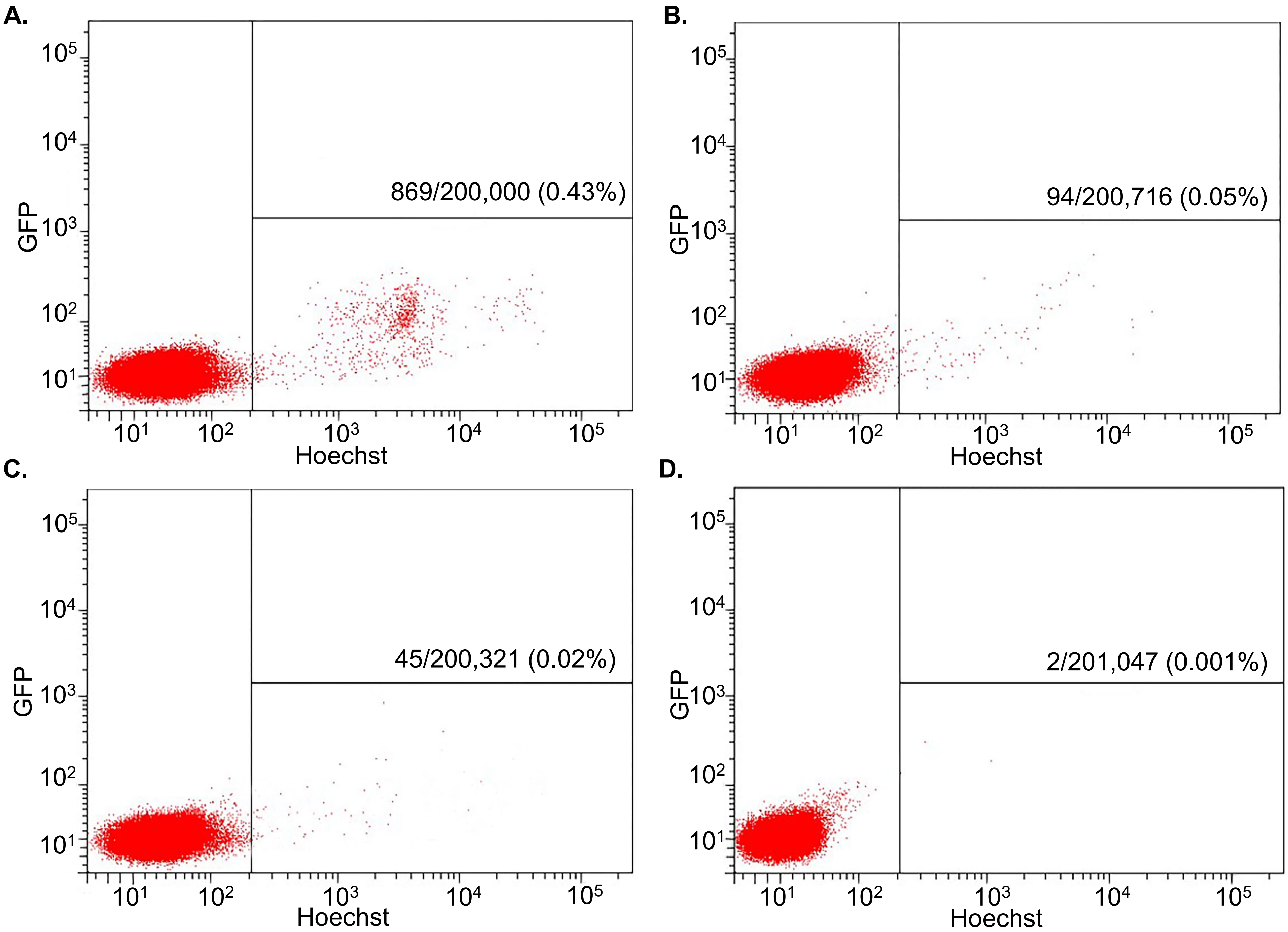

Figure S1. Depletion of WBCs in blood. WBCs were stained by Hoechst 33342 and detected by flow-cytometry. (**A**). About 0.4-0.5% of WBCs in the blood before depletion. (**B**). About 0.05% WBCs left in the blood after the "buffy coat" was removed, (**C**). About 0.02% WBCs in the blood after one week of storage in 4 ^{o}C. (**D**). Only about 0.001% WBCs left in the blood after using Plasmodipur filters. Based on the size of the human (6.27 gigabases) and *P. falciparum* (22.9 megabases) genome and the percentage of 5mC (~4%) in the human genome. We calculated that the contamination from WBC DNA will only count ~2-5% of parasite genomic DNA harvested from the culture at 5% parasitemia and 5mC from WBC DNA will only reach 0.001-0.002% in the harvested DNA from the parasite culture at 5% parasitemia.

Figure S2.

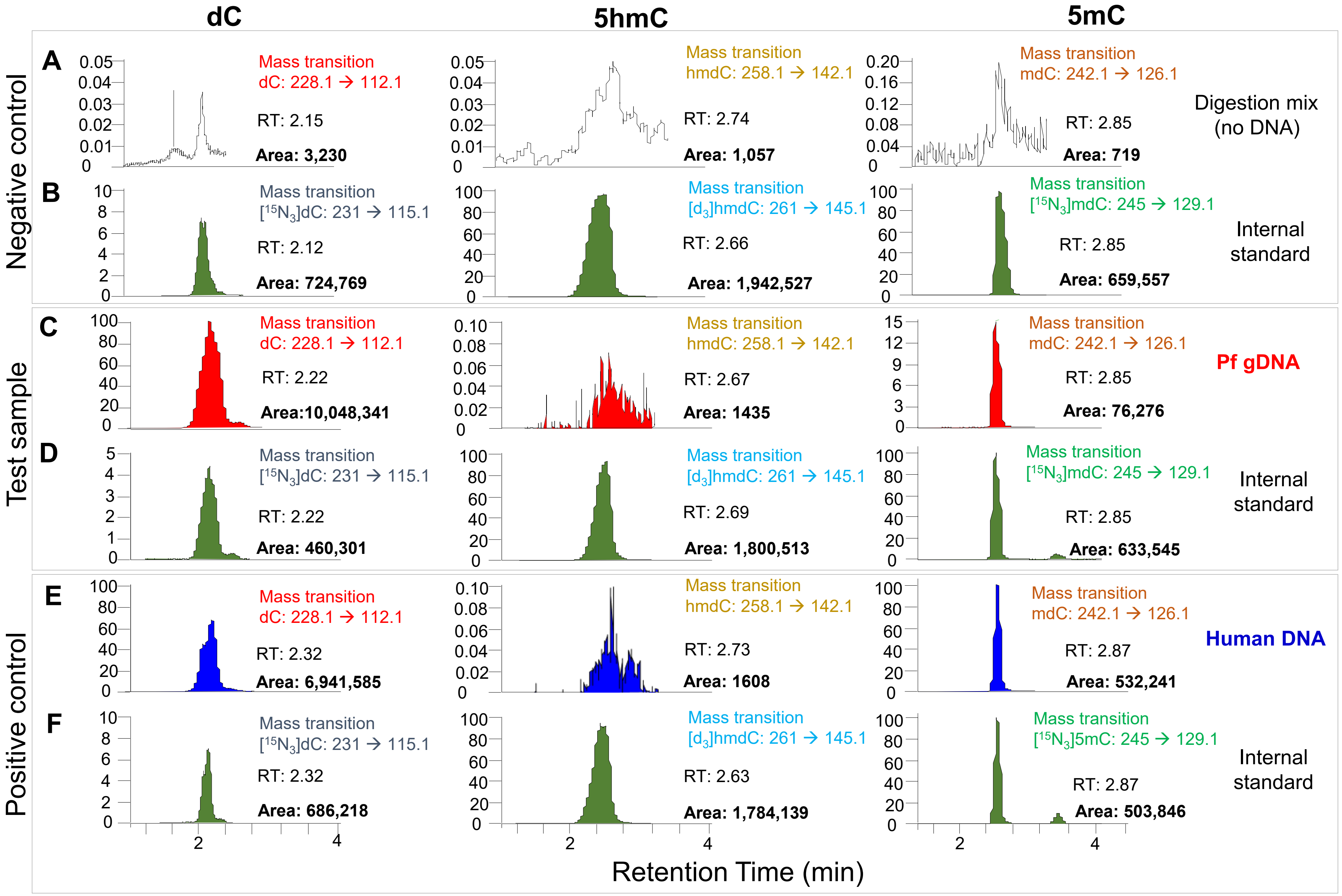

Figure S2. Detection of DNA methylation by mass spectrometry. (**A-F**). LC-MS/MS captured spectra using the selected reaction monitoring mode for *P. falciparum* gDNA (**C**, red) from WT parasites at the trophozoite stage. The x- and y-axis denote the retention time and the relative abundance, respectively. The digestion mix containing buffers and enzymes served as a negative control (**A**). Human DNA (**E**, blue) was used as a 5mC positive control. Internal standards (**B**, **D**, and **F**, green) for all nucleosides were measured to confirm the analytes' identities. The internal standards have similar retention times (RTs) as the sample deoxynucleotides, differing by mass shown as the size of the area under each peak. The mass transition 228.1 → 112.1, 242.1 → 126.1, and 258.1→ 142.1 were monitored for detection of dC, 5mC, and 5hmC, respectively while the mass transition 231 → 115.1, 245 → 129.1, and 261→ 145.1 were monitored for the detection of [$^{15}N_3$]dC, [$^{15}N_3$]mdC, and [d_3]hmC, respectively.

Figure S3.

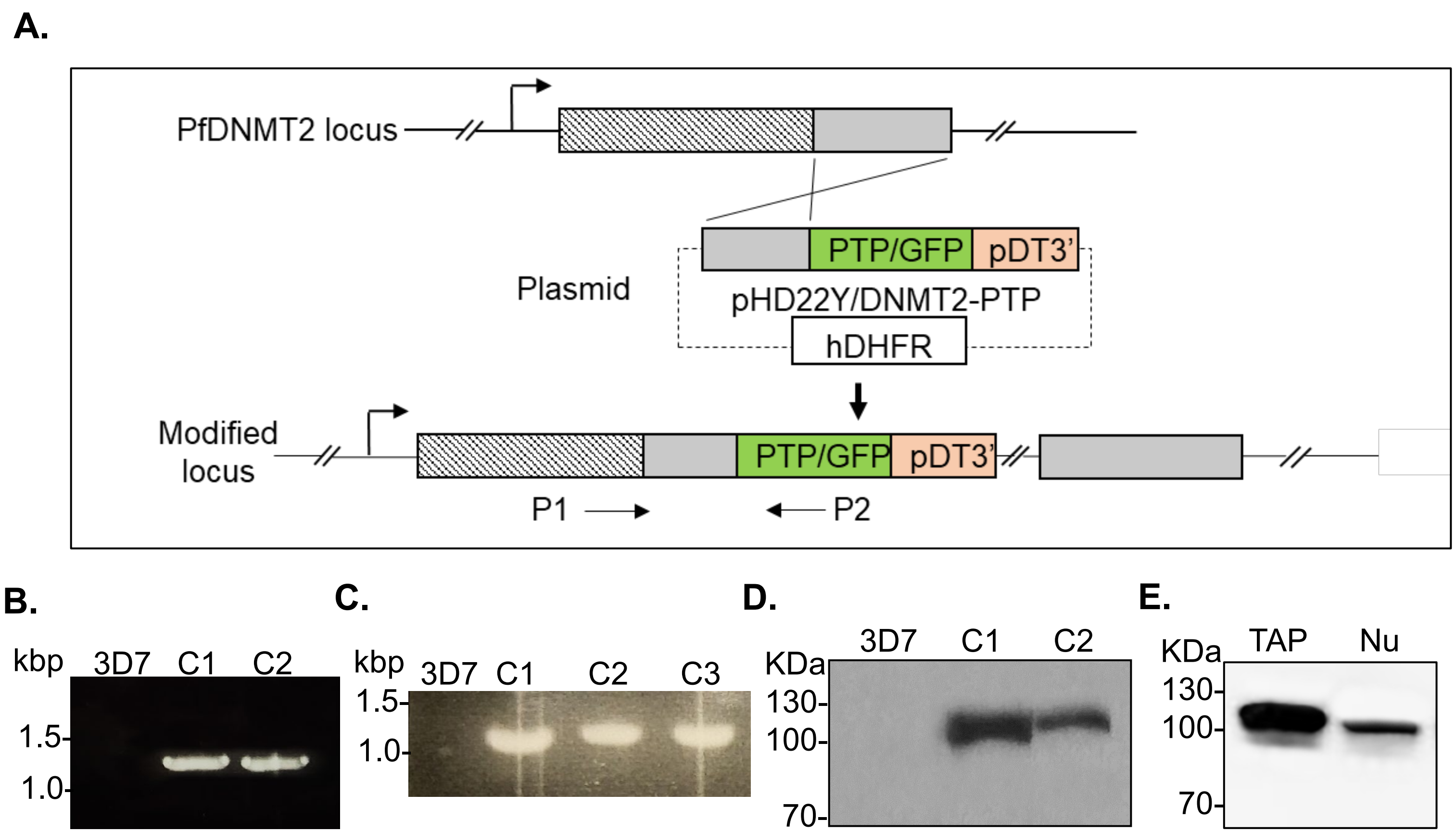

Figure S3. Generation of chimeric PfDNMT2 in *P. falciparum*. (**A**) A schematic diagram showing PfDNMT2 C-terminal tagging with PTP or GFP by a single cross-over recombination strategy. hDHRF, human DHFR drug selection cassette; P1 and P2 indicate primer 1 and 2 binding upstream of the homologous arm and in PTP/GFP for the diagnostic PCR shown in **B** and **C**. (**B**) Diagnostic PCR with primers P1 × P2 confirming successful PTP tagging in 2 clones (C1 and C2). (**C**) Diagnostic PCR with primers P1 × P2 confirming the successful GFP tagging in three clones (C1-C3). (**D**) Western blotting shows the expression of PTP-tagged PfDNMT2 (~102 kDa) in clones C1 and C2 using an anti-protein C antibody. (**E**) Western blotting shows the expression of PTP-tagged PfDNMT2 in 0.2 μg of TAP eluate and 10 μg of nuclear extracts (Nu).

Figure S4.

A.

```
                               ____I______
P.fal          -----------MHKIKVLELYCGIGGLHYSLLQAFNNFVHANKITEKKCDTYKDGIHNHM  49
A.thaliana     MAEQELQRINEKKPWQVLEFYSGIGGMRYSLMASGIV-----------------------  37
S.pombe        --------MLSTKRLRVLELYSGIGGMHYALNLANIP-----------------------  29
D.Melanogast   ------------MVFRVLELFSGIGGMHYAFNYAQLD-----------------------  25
B.mori         --------MEEKMEHRILELYSGIGGMHCAWNESTIK-----------------------  29
D.rerio        --------MENTERLRVFELYSGIGGMHYALKESLVP-----------------------  29
H.sapiens      -----------MEPLRVLELYSGVGGMHHALRESCIP-----------------------  26
M.musculus     -----------MEPLRVLELYSGIGGMHHALRESHIP-----------------------  26
                              :::*::.*:**:: :   :

                                         _______II______
P.fal          SNNKSIEIHKYHDCTLTCLNDLFCFISVDLNPVANQTYFHNFKDSTILLTQTRDVHKFFK  109
A.thaliana     ---------------------SEVVEAFEINDSANDVYQHNFKHR-PY------------  63
S.pombe        ---------------------ADIVCAIDINPQANEIYNLNHGKL-AK------------  55
D.Melanogast   ---------------------GQIVAALDVNTVANAVYAHNYGSNLVK------------  52
B.Mori         ---------------------GKVVAAIDINTVANDVYKYNFPETLLF------------  56
D.Rerio        ---------------------AEVVAAVDVNTTANLIYKHNFPTTQLL------------  56
H.sapiens      ---------------------AQVVAAIDVNTVANEVYKYNFPHTQLL------------  53
M.musculus     ---------------------AHVVAAIDVNTVANEVYKHNFPHTHLL------------  53
                                       . :.::*  **  *  *.

                                                                  _III
P.fal          WKCDSMDKQSNGSTHNEHEKTQKKKNKKKNNDDDEKNNIFNINKNYIIQTDINNIMPEFL  169
A.thaliana     ------------------------------------------------QGNIQSLTAADL  75
S.pombe        ------------------------------------------------HMDISTLTAKDF  67
D.Melanogast   ------------------------------------------------TRNIQSLSVKEV  64
B.Mori         ------------------------------------------------TKNIQSLTPIEI  68
D.Rerio        ------------------------------------------------PKTIEGMTLQDF  68
H.sapiens      ------------------------------------------------AKTIEGITLEEF  65
M.musculus     ------------------------------------------------SKTIEGISLEDF  65
                                                                  *. :    .

                         ___IV___
P.fal          NNHHFNILLISNPCQPYTRQNQKFKEINLDLLFCKNNEYKQNVNNNISNDNNSFYSNHNG  229
A.thaliana     DKYNADAWLLSPPCQPYTRQGLQK------------------------------------  99
S.pombe        DAFDCKLWTMSPSCQPFTRIGNRK------------------------------------  91
D.Melanogast   TKLQANMLLMSPPCQPHTRQGLQR------------------------------------  88
B.Mori         EKYKIDTVLMSPPCQPFTRNGKNL------------------------------------  92
D.Rerio        DRLNFDMILMSPPCQPFTRIGLQG------------------------------------  92
H.sapiens      DRLSFDMILMSPPCQPFTRIGRQG------------------------------------  89
M.musculus     DKLSFNMILMSPPCQPFTRIGLQG------------------------------------  89
                    .   :*  ***.** . .

                                                V                ____VI___
P.fal          DENHQFNVDNINIDELNNYIYNDKDERTKSFIHICTLLTKVDFKNLPEYIFIENVKNFEL  289
A.thaliana     ---------------------HSGDARAFSFLRILELIPHT--TKPPQMLFVENVVGFET  136
S.pombe        ---------------------DILDPRSQAFLNILNVLPHV--NNLPEYILIENVQGFEE  128
D.Melanogast   ---------------------DTEDKRSDALTHLCGLIPEC--QEL-EYILMENVKGFES  124
B.Mori         ---------------------DENDPRTNSFLYFIDILDKL--NTL-QYILMENVKGFEC  128
D.Rerio        ---------------------DVADPRTKSFLYILDLLPRL--SKRPRFILLENVKGFES  129
H.sapiens      ---------------------DMTDSRTNSFLHILDILPRL--QKLPKYILLENVKGFEV  126
M.musculus     ---------------------DMTDPRTTSFLYILDILPRL--QKLPKYILLENVKGFEV  126
                                    .  * *: ::  :  :: .       . :::*** .**

                              ____VII___    __VIII_
P.fal          SSSFIYFLYCI-KNNYSFQTYLLSPLQFGIPNERLRFYCICKKKNYDFKHANNLSGINYI  348
A.thaliana     SDTHMEMIGTLTKLDYVTQEFILSPLQFGVPYSRPRYFCLAKRKPLPFKSQHSNNKLLWS  196
S.pombe        SKAAEECRKVLRNCGYNLIEGILSPNQFNIPNSRSRWYGLARLNF-----KGEWSIDDVF  183
D.Melanogast   SQARNQFIESLERSGFHWREFILTPTQFNVPNTRYRYYCIARKGA-DFPFAG-GKIWEEM  182
B.Mori         STVRNLFVEKLTYCGFVYQEFMLSPVSVGVPNSRLRYYCIAKRNN-TWNFKRKDELITCL  187
D.Rerio        SAARDALLQTLRECDYSFQEFLISPTSLGIPNSRLRYFLIAKRAPETFSFPVSTEIIEGF  189
H.sapiens      SSTRDLLIQTIENCGFQYQEFILSPTSLGIPNSRLRYFLIAKLQSEPLPFQAPGQVLMEF  186
M.musculus     SSTRGLLIQTIEACGFQYQEFLLSPSSLGIPNSRLRYFLIAKLQSEPFPFQAPGQILMEF  186
               *         :   .:     :::* ...:*  * *:: :.:            .

P.fal          KDKNLNLYTNSLIPKNYLH--KNNIHEQKNNQGD--NYNNISCE---NVIFYTPNLITYL  401
A.thaliana     PDPLYG----RDDQVEFGKC--------------------QAEEGLDKLLEFCKPVEKFL  232
S.pombe        QFSE------VA------------------------------------------------  189
D.Melanogast   PGAI------AQNQ----------------------------------------------  190
B.Mori         PKTF------AK------------------------------------------------  193
D.Rerio        PMSE------STDGLSVPYD----------------HPTSSASEREKTIM---------F  218
H.sapiens      PKIE------SVHPQKYAMDVENKIQEKNVEPNISFD-GSIQCSGKDAIL---------F  230
M.musculus     PKIV------TVEPQKYAVVEESQPRVQRTGPRICAESSSTQSSGKDTIL---------F  231

P.fal          DVNNNFNIT------------NNIWNHINIYNNYLDNYQVQNQVLQKNASYCFDIININK  449
A.thaliana     ELAAHVDGEPSSVDDSENGSKDCCGQEGDSVPDSVHQYLVPVSLIERWG-----------  281
S.pombe        --------------QKE-GEVKRIRDYLEI-ERDWSSYMVLESVLNKWG-----------  222
D.Melanogast   ------------------GLSQIAEIVE--ENVSPDFLVPDDVLTKRV-----------   218
B.Mori         --------------------PHCLKDIIEN--NVPDDYL-VPDKMLRKA-----------  219
D.Rerio        KLETEEDLERKRKQNSQ-ESVRRLLDFLQEEEEDMEPYLLPPKTLLRYA-----------  266
H.sapiens      KLETAEEIHRKNQQDSD-LSVKMLKDFLED-DTDVNQYLLPPKSLLRYA-----------  277
M.musculus     KLETVEERDRKHQQDSD-LSVQMLKDFLED-G-DTDEYLLPPKLLLRYA-----------  277
                                        :  :        :    . : :

                                                 ____________TRD___________
P.fal          KTTTCCHVANYYHHHHQKKKENVNNISPDEPTKHMNAKGNYAMCFTSNYGRYIKGSGSIL  509
A.thaliana     ---------------------N--------AMDIVYPDSKRCCCFTKSYYRYVKGTGSLL  312
S.pombe        ---------------------H--------QFDIVKPDSSSCCCFTRGYTHLVQGAGSIL  253
D.Melanogast   ---------------------L--------VMDIIHPAQSRSMCFTKGYTHYTEGTGSAY  249
B.Mori         ---------------------N--------IFDICYADSNRSCCFTKAYTHYVEGTGSVF  250
D.Rerio        ---------------------L--------VMDIVQPSSRRSVCFTKGYGHYVEGTGSVL  297
H.sapiens      ---------------------L--------LLDIVQPTCRRSVCFTKGYGSYIEGTGSVL  308
M.musculus     ---------------------L--------LLDIVKPTSRRSMCFTKGYGSYIEGTGSVL  308
                                               .        . ***  *    :*:**
P.fal          YYNRKENSNCAEQKTKNKETNVLTKENNEYVHTSNYTCNSISNSDNDTYETRRKKNCMKK  569
A.thaliana     ATVQPKI----------------------------------------------KGKESCL  326
S.pombe        QMSDHENTHEQF--------------------------------------------ERNR  269
D.Melanogast   TPLSEDESHRIFE-------------------LVKE--IDTSNQDASKSEKILQQRLDLL  288
B.Mori         TETSYDIVQKYLK-------------------LANY--FEVGS----------DEFLQTL  279
D.Rerio        QSCMDVDLETVF---------------------KNL--DQLSE----------EDKLKQL  324
H.sapiens      QTAEDVQVENIY---------------------KSL--TNLSQ----------EEQITKL  335
M.musculus     QAAEDAQIENIY---------------------KSL--PDLPP----------EEKIAKL  335

                   _______IX_______
P.fal          YEQNVRYFTPTEISRLMGFKMKTNNKNINQNEKGKNTYGNVFWNIDHINHTCAYFSNVHY  629
A.thaliana     KEQRLRYFTPREVANFHSFPE---------------------------------------  347
S.pombe        MALQLRYFTAREVARLMGFPE---------------------------------------  290
D.Melanogast   HQVRLRYFTPREVARLMSFPE---------------------------------------  309
B.Mori         KKLKLRFFTSKEILQLMSFPS---------------------------------------  300
D.Rerio        LRLKLRYFTPREISRLMGFPD---------------------------------------  345
H.sapiens      LILKLRYFTPKEIANLLGFPP---------------------------------------  356
M.musculus     SMLKLRYFTPKEIANLQGFPP---------------------------------------  356
                  .:*:**  *: .: .*

                                                                 _______X__
P.fal          CDVQNKNACLLTYQNVTNLNNNHTYQNCHQKNCLCHEFVFPNF-LTDRQKYKLIGNSVNV  688
A.thaliana     ------------------------------------DFEFPKH-ISLRQRYAMLGNSLSV  370
S.pombe        ------------------------------------SLEWSKSNVTEKCMYRLLGNSINV  314
D.Melanogast   ------------------------------------NFEFPPE-TTNRQKYRLLGNSINV  332
B.Mori         ------------------------------------EYSFPKT-VTRKQCYRLLGNSVNV  323
D.Rerio        ------------------------------------HFTFPKH-ISFKQQYRVLGNSLNV  368
H.sapiens      ------------------------------------EFGFPEK-ITVKQRYRLLGNSLNV  379
M.musculus     ------------------------------------EFGFPEK-TTVKQRYRLLGNSLNV  379
                                                      :     : :  * ::***:.*

               ______
P.fal          IVISYIFHVHNIFEHIHI--------------------     706
A.thaliana     AVVAPLLRYLF--DS-----------------------     383
S.pombe        KVVSYLISLLL--EPLNF--------------------     330
D.Melanogast   KVVGELIKLLT--IK-----------------------     345
B.Mori         KVISELLQILF--DE-----------------------     336
D.Rerio        HVVSHLIRLML--SK-----------------------     381
H.sapiens      HVVAKLIKILY--E------------------------     391
M.Musculus     HVVAKLLTVLC--EGFGNASESCHKMPLILDSNSKILS     415
                *:. ::
```

Figure S4. (Cont.)

B.

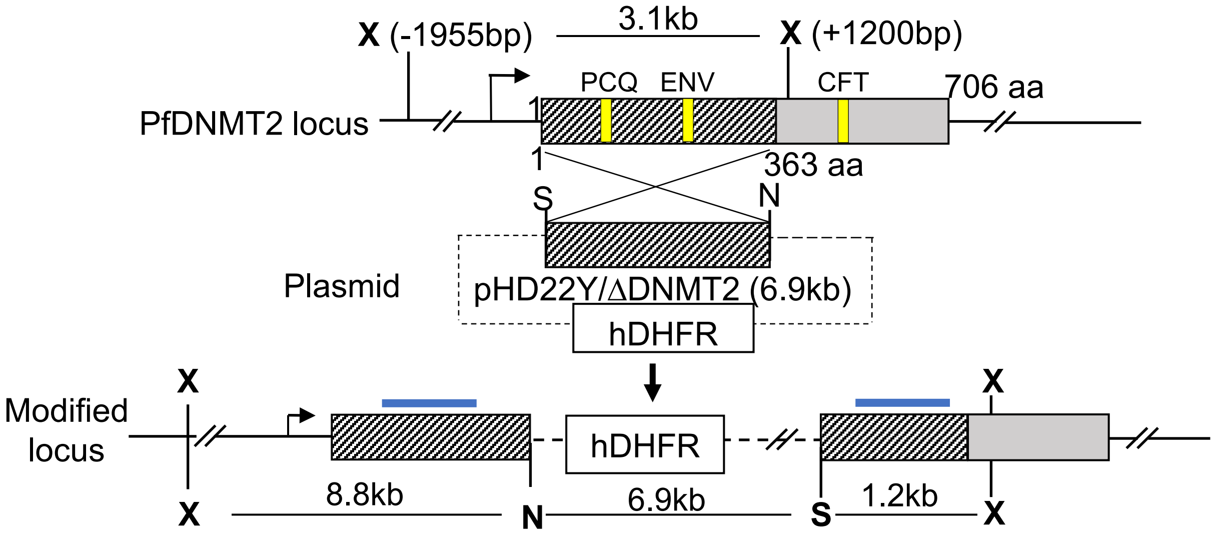

C.

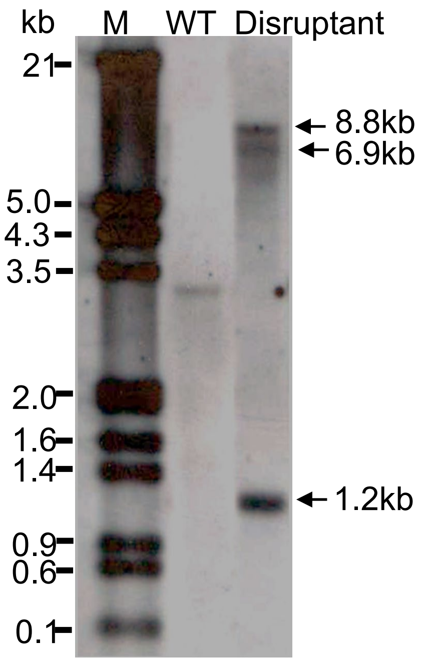

Figure S4. DNMT2 sequence alignment and PfDNMT2 disruption. (**A**) Alignment *PfDNMT2* with *DNMT2* sequences from six species showing conserved motifs. Motifs shaded in yellow (PCQ in domain IV and ENV in domain VI) are essential for enzyme binding and DNA methylation activity. The motif colored in green in the target recognition domain (TRD) determines DNA target specificity and tRNA specificity. DNMT2 sequences were obtained from GenBank for *A. thaliana* (AED93453), *S. pombe* (CAA57824), *D. melanogaster* (AAF53163), *Bombyx mori* (BAD67190), *D. rerio* (BAD95483), *H. sapiens* (AAC39764), and *M. musculus* (AAC53529). (**B**) A schematic diagram shows the disruption of *PfDNMT2* by single cross-over homologous recombination. Two motifs essential for enzyme binding and DNA methylation activity (PCQ and ENV) and one motif for DNA/tRNA specificity (CFT) are highlighted in yellow. The blue line indicated the location of the probe. Restriction enzyme sites are marked as X for *Xho*I, S for *Spe*I, and N for *Not*I. **(C)** Southern blot confirmed disruption of *PfDNMT2* locus. M, DNA marker.

Figure S5.

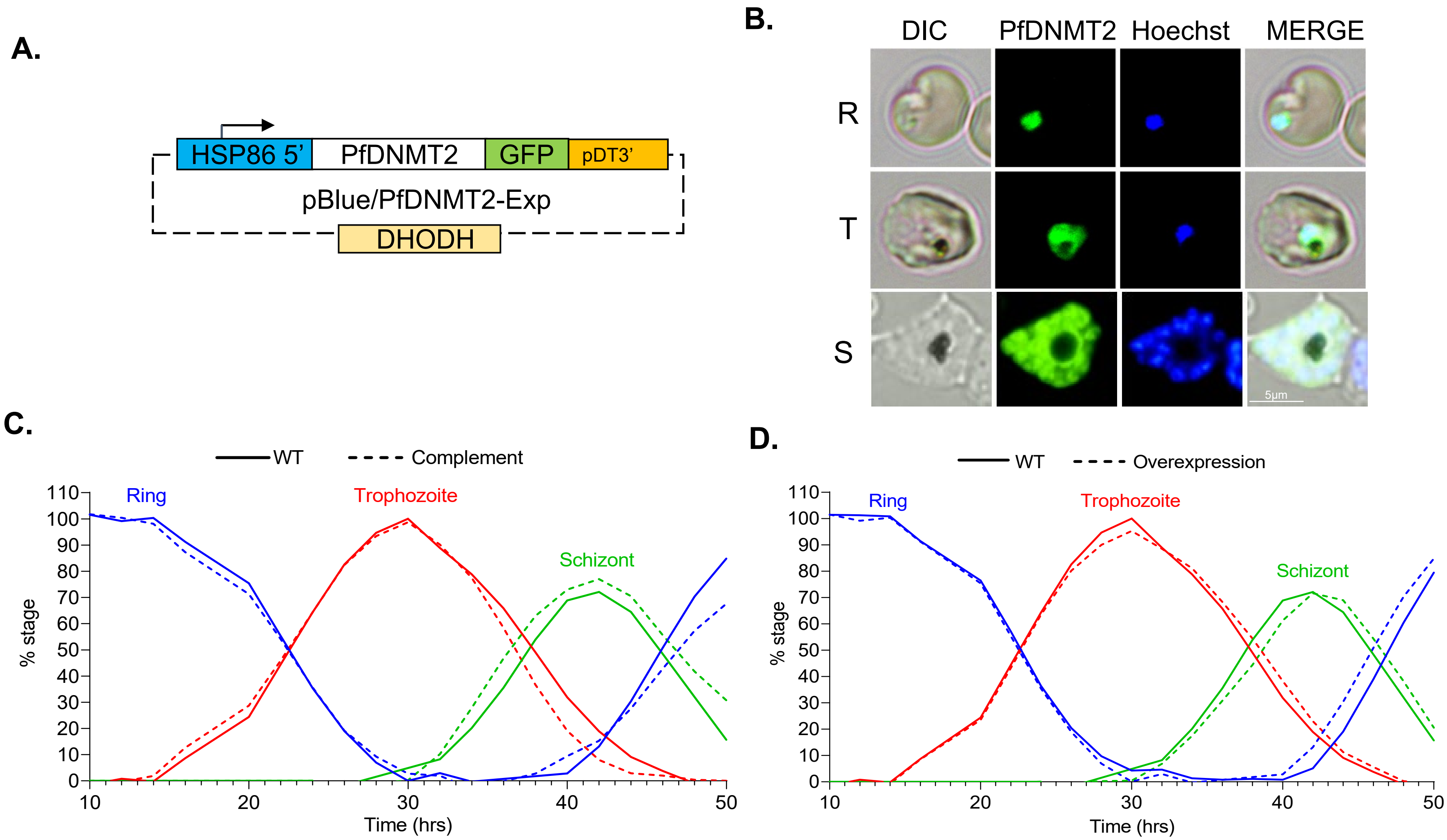

Figure S5. Complementation and overexpression of *PfDNMT2*. (**A**) A schematic diagram showing the PfDNMT2-GFP expression construct. (**B**) Episomal PfDNMT2-GFP expression in the ring (R), trophozoite (T), and schizont (S) stages was detected by live imaging. Scale bar = 5 μm. Parasite nuclei were stained by Hoechst. (**C, D**) Growth course monitoring of highly synchronous rings throughout the IDC for the complementation **(C)** and overexpression **(D)** parasite lines (dashed lines) compared to the WT (solid line). Blood smears were collected every 2 h, and the percentage of each stage was counted (*y*-axis) and shown by blue (ring), red (trophozoite), and green (schizont) lines.

Figure S6

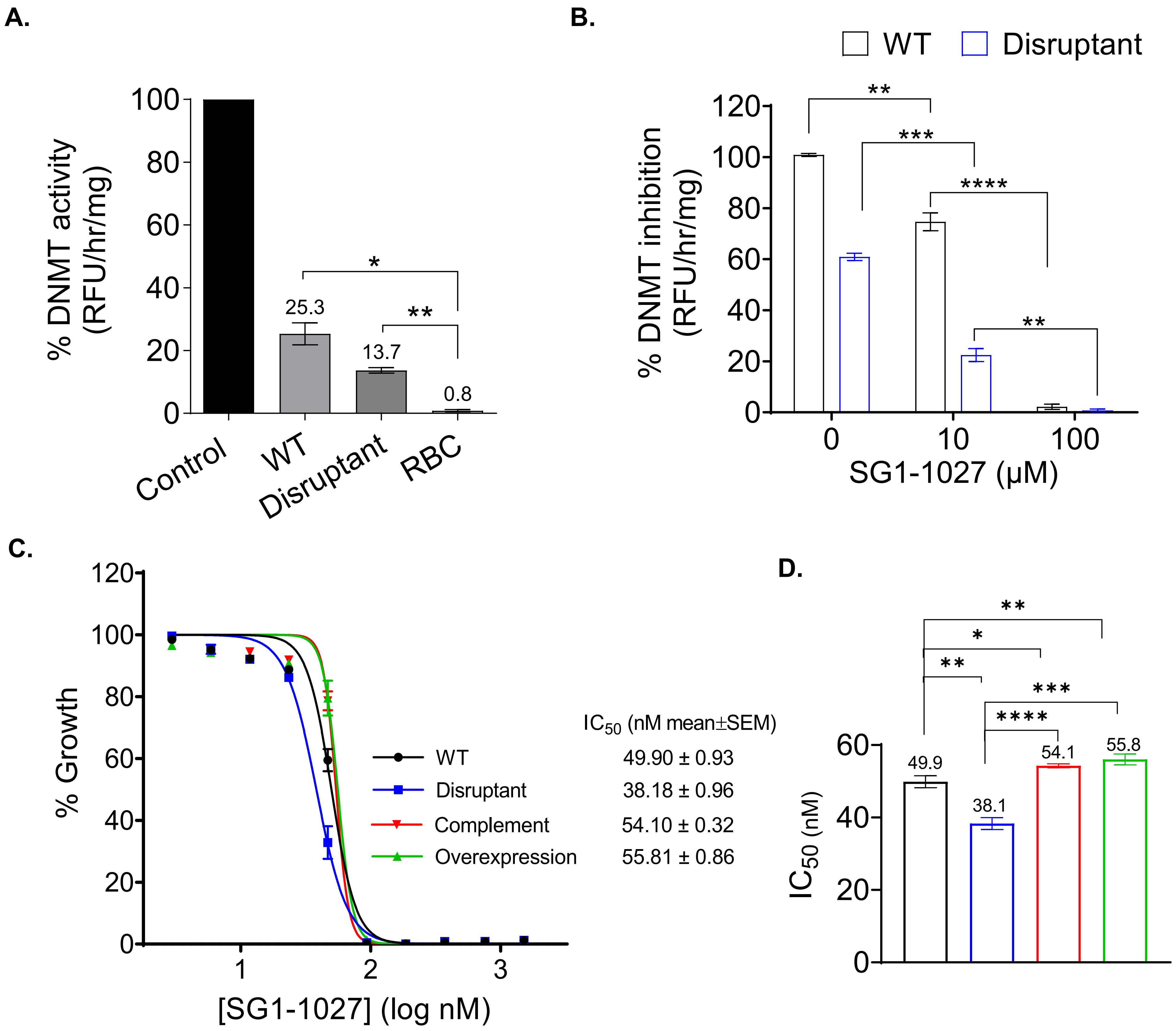

Figure S6. Alterations of DNMT activities and parasite sensitivities after manipulation of *PfDNMT2* or using a DNMT inhibitor. (**A**). DNMT activity assays of parasite nuclear extracts were performed in triplicate. 50 ng of bacterial DNMT was used as a positive control. ~10 μg of extracts from ~2 x 10^7 parasites harvested from blood culture at 5% parasitemia (~4 x 10^8 RBCs) were tested for DNMT activity. The extracts from same number of uninfected RBCs were analyzed to check potential contamination from WBCs in the blood. *PfDNMT2* disruption caused a ~50% decrease in DNA methylation activity from the nuclear extract compared to WT. Numbers atop bars show percentage activity to DNMT control. RFU/hr/mg denotes units of fluorescence per hour per mg of proteins. * and ** denote $P < 0.05$ and 0.01, respectively (paired *t*-test). (**B**). DNMT activity assays were performed in presence of a DNMT inhibitor, SG1-1027 at 0, 10 and 100 μM. DNMT activity from WT without inhibitor was setup as 100%. **, $P < 0.01$; ***, $P < 0.001$, ****, $P < 0.0001$ (paired *t*-test). (**C**) SG1-1027 dose response curves and IC_{50} values of PfDNMT2 disruptant, complement and overexpression lines. WT served as a control. Data points are the mean ± standard error of mean from at least three independent triplicate measurements. (**D**) Statistical differences in IC_{50} values (shown above bars) from C. Comparison of the results was done using an unpaired t-test and the *P* values are indicated. *, $P < 0.05$; **, $P < 0.01$; ***, $P < 0.001$, ****, $P < 0.0001$.

Figure S7.

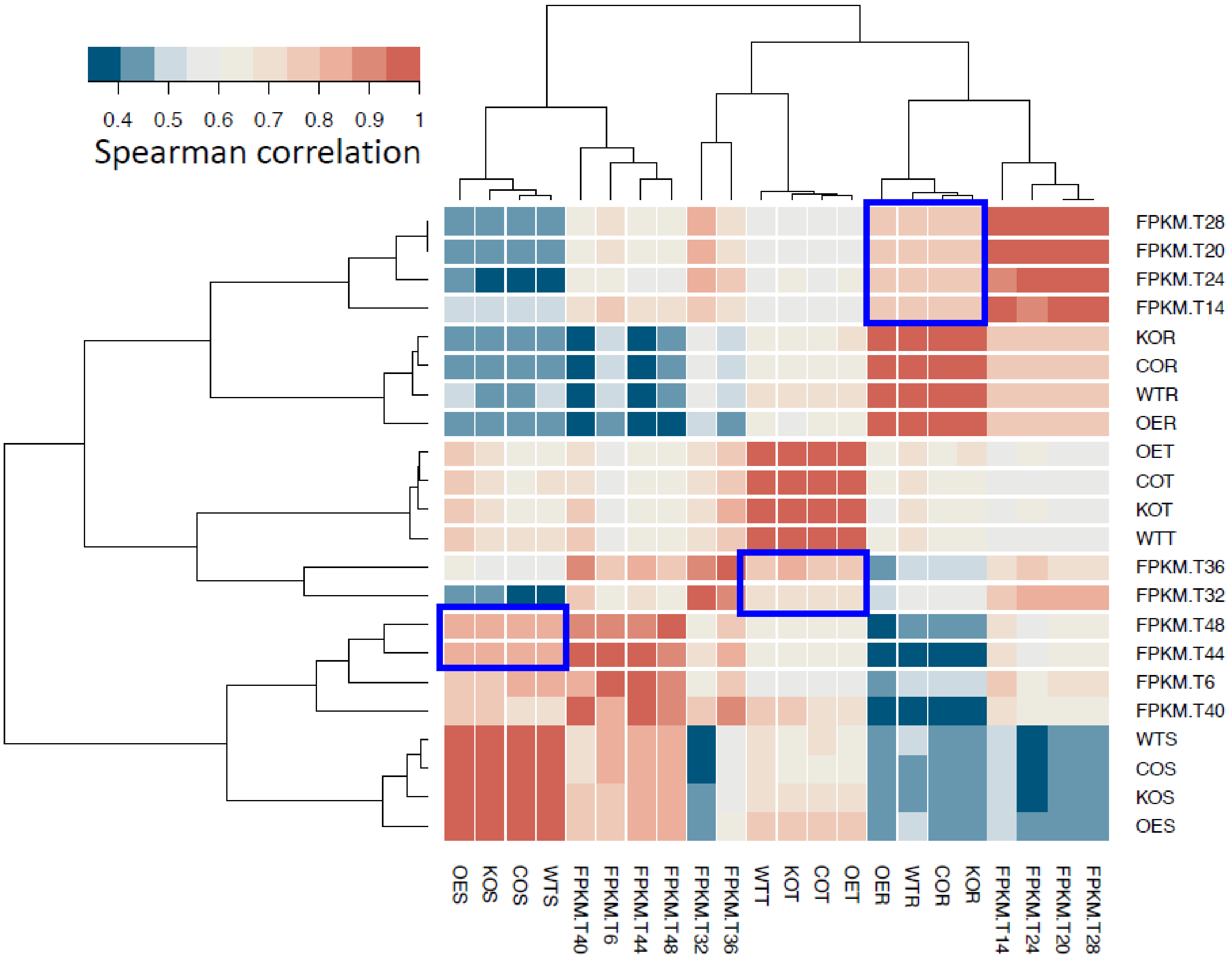

Figure S7. The correlations between the *PfDNMT2* transcriptomes and the IDC time course. A heat map represents the correlation between the published asexual stage transcriptomes at 10 time-points (T) of IDC (from Broadbent et al. 2015) and the transcriptomes of 4 parasite lines (3D7, WT; *PfDNMT2* disruption, KO; complementation, CO; and overexpression, OE) in this study at 3-time points in the ring (R, 10 h), trophozoite (T, 30 h), and schizont (S, 40 h) stages. The blue boxes show that the transcriptomes of the *PfDNMT2* WT, KO, CO, and OE parasites were highly correlated with the published parasite transcriptomes at T14, T36, and T44, respectively.

Figure S8.

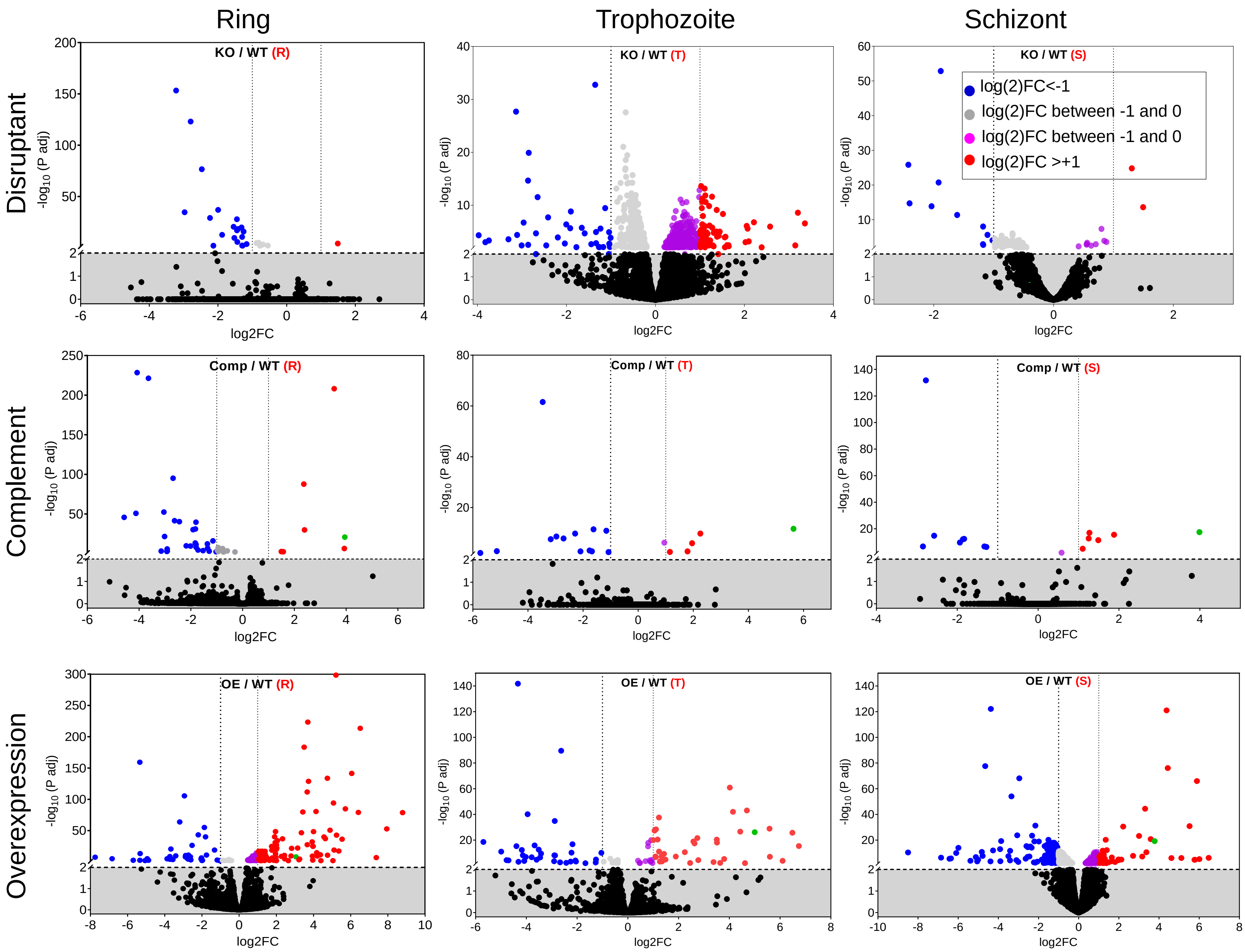

Figure S8. Identification of differentially expressed genes after genetic manipulations of *PfDNMT2*. Volcano plots show altered gene expression at the ring (R), trophozoite (T), and schizont (S) stages in the disruptant (KO), complementation (Comp), and overexpression (OE) compared to the WT 3D7. The x-axis indicates log2 Fold change (FC) of the transcript level in transgenic parasites compared to WT 3D7, while the y-axis indicates -log10 of the adjusted *P* values. The green dots denote PfDNMT2 (PF3D7_0727300). Transcripts are colored based on fold change values (transgenic lines/WT).

Figure S9

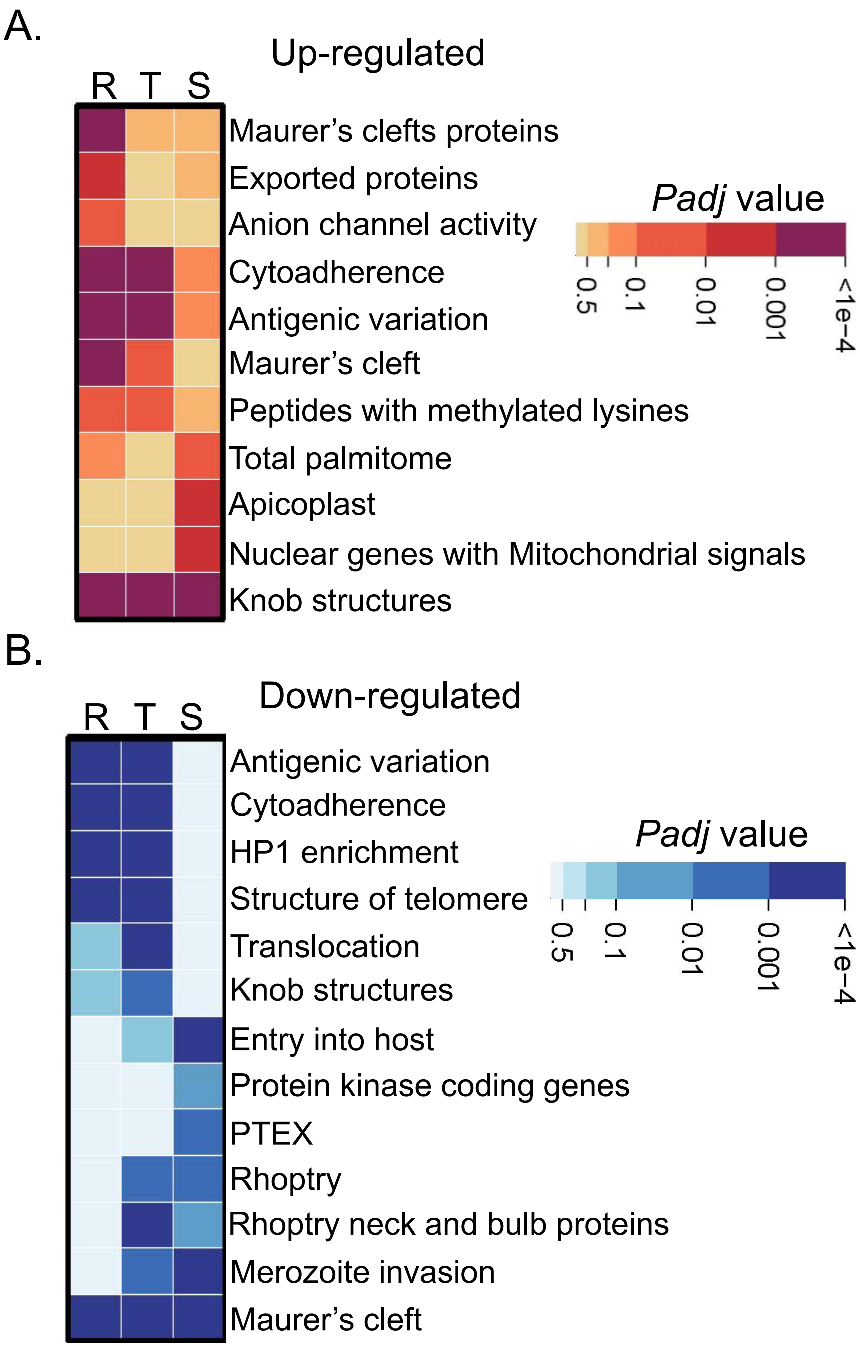

Figure S9. GO enrichment analysis upon overexpression of *PfDNMT2*. Heatmaps show the enriched GO terms of up-regulated (**A**) and down-regulated (**B**) genes in the *PfDNMT2* overexpression line compared to 3D7-WT at the ring (R), trophozoite (T), and schizont (S) stages. The color bars show the scales of adjusted P values.

Figure S10

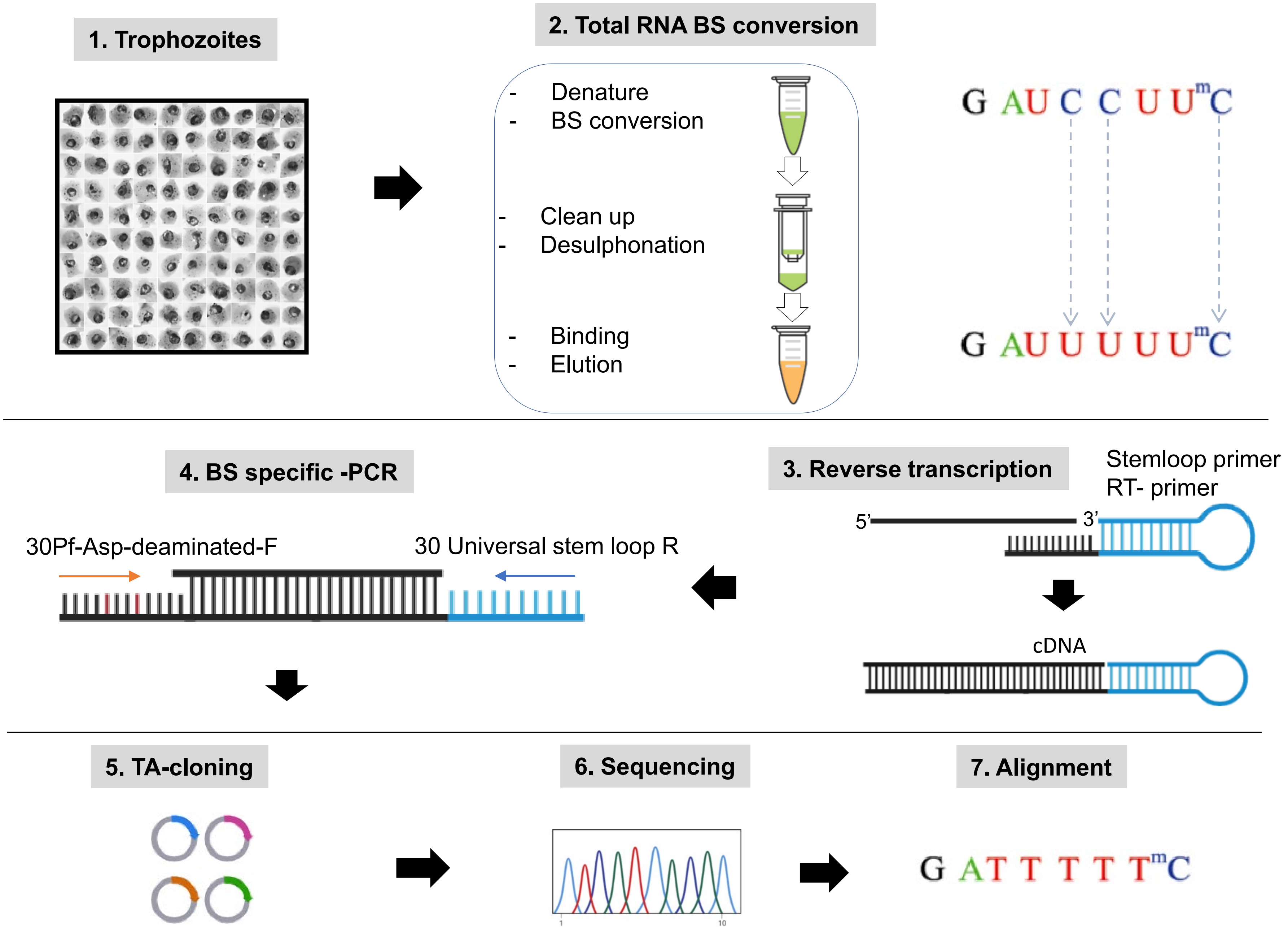

Figure S10. RNA bisulfite amplicon sequencing. The workflow of RNA bisulfite amplicon sequencing procedure adopted for *P. falciparum* tRNA. BS; bisulfite. See details in the Materials and Methods section.

Figure 11.

A.

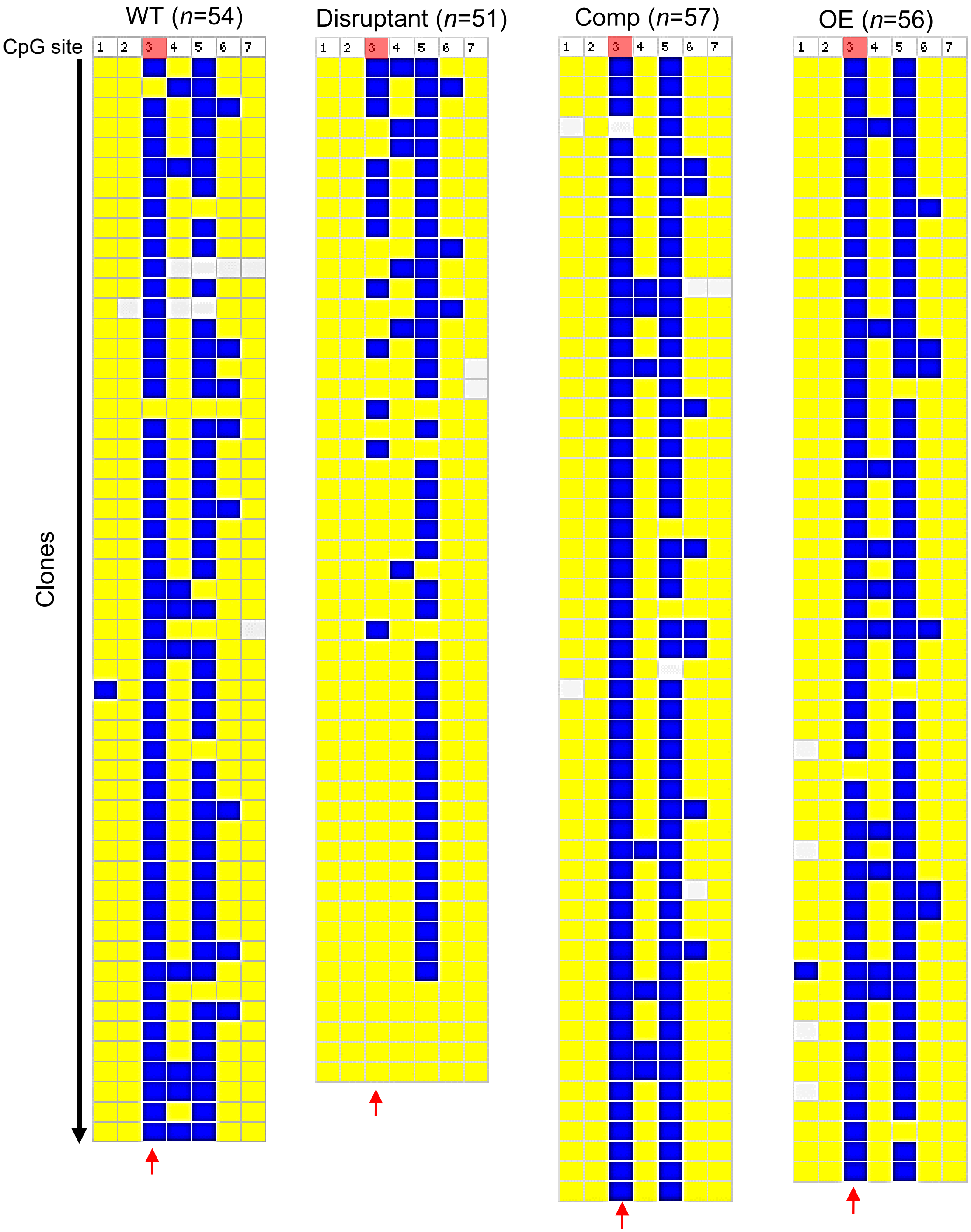

B.

CpG site	1	2	3	4	5	6	7
CpG position	3	29	38	40	49	62	68
Methylation [%] in WT	1.9	0.0	96.3	17.3	88.5	15.1	0.0
Methylation [%] in Disruptant	0.0	0.0	23.5	11.8	82.4	5.9	0.0
Methylation [%] in Comp	0.0	0.0	100	12.3	96.4	14.5	0.0
Methylation [%] in OE	1.9	0.0	98.2	17.9	94.6	10.7	0.0

Figure S11. tRNA^{ASP} methylation determined by RNA bisulfite amplicon sequencing. (**A**) Plots show the cytosine methylation status in the tRNA^{ASP} from the wild-type (WT), *PfDNMT2* disruption (disruptant), complementation (Comp), and overexpression (OE) parasite lines. BISMA software was used to generate these plots. The numbers on top show all the seven CpG sites in the tRNA^{ASP} and each row represents results from each clone. Blue – methylated cytosines; Yellow – unmethylated cytosines; Gray – Unknown status (due to the vague sequence results). The cytosine at position 38 is marked by the red arrows. *n* represents the number of clones sequenced. (**B**) A table summarizing the results from A. The data for cytosine at position 38 are shadowed in red.
