# Supplementary material for: Characterization of the dual role of Plasmodium falciparum DNA methyltransferase in regulating transcription and translation
Source: Nucleic Acids Res. 2023 Apr 7;51(8):3918–33. doi: 10.1093/nar/gkad248 (PMC10164579; doi:10.1093/nar/gkad248)
Supplement: gkad248_Supplemental_Files [file gkad248_supplemental_files.zip › Supplementary Tables.pdf]

Table S1. Primer information

Name	Sequence (5'-3')	Purpose
Int-F	GAAGATCTCGGTATACCCAATGAACGACTTCG, *Bgl* II underlined	amplification of C-terminus PfDNMT2
Int-R	GACCTAGGTATGTGTATATGCTCAAATATATTGTGGACG -3', *Avr* II underlined	amplification of C-terminus PfDNMT3
KO-F	GGACTAGTGCACAAAATAAAGGTACTGG-3', *Spe* I underlined	PfDNMT2 gene disruption
KO-R	ATAAGAATGCGGCCGCCTTGGGTATTAAAGAATTTGTG-3', *Not* I underlined	PfDNMT2 gene disruption
Exp-F	GAAGATCTATGCACAAAATAAAGGTACTGG-3', *Bgl* II underlined	PfDNMT2 gene expression
Exp-R	GACCTAGGTATGTGTATATGCTCAAATATATTGTGG-3', *Avr* II underlined	PfDNMT2 gene expression
Int-diag-F	CAGACATATTTACTTTCCCCTTTAC	Integration diagnostic PCR
GFP-R	CAACTCCAGTGAAAAGTTCTTCTCC	Integration diagnostic PCR
PTP-R	CGAGGATCGACCTGATCTTCC	Integration diagnostic PCR
DIG-F	GCGGAATAGGAGGATTACAC	Southern blot
DIG-R	CCGAATTGTAAAGGGGAAAG	Southern blot
PfDNMT2 Fw	CAACCACTTGTTGTCATGTGGCAAA	RT qPCR
PfDNMT2 Rv	TGCGTTCATATGCTTGGTTGGTTCA	RT qPCR
stRNA Fw	AAGTAGCAGGTCATCGTGGTT	RT qPCR
stRNA Rv	TTCGGCACATTCTTCCATAA	RT qPCR
55Pf-AspStem-loop	CTCAACTGGTGTCGTGGAGTCGGCAATTCAGTTGAGTGGTAACTCCAAAA CCAAA	reverse transcription, bisulfite-converted total RNA
30Pf-Asp-deaminated-F	TTTGAGATAGTATAGTGGTAAGTATTTTTG	Aspartic acid tRNA enrichment
30Universal stem-loop R	CTGAATTGCCGACTCCACGACACCAGTTGA	Aspartic acid tRNA enrichment
D6F	GCCCAAGCTTGGATCTAATGGACGACGACGACGATGACG	6× aspartate tagging
D6R	TACTCCTAGGGGATCCGTCATCGTCGTCGTCGTCCATT	6× aspartate tagging

6×D-GFP F	CACTGGAGTTGTCCCAATTCTTGT	RT qPCR
6×D-GFP R	CTCGCAAAGCATTGAAGACCATACG	RT qPCR
tRNAasp-F	TCCGAGATAGTATAGTGG	RT qPCR
tRNAasp-R	CTCAACTGGTGTCGTGGAGTCGGCAATTCAGTTGAGTGGTAACTCCGAGACC	RT qPCR

Table S2A. Proteomic data from IPs of PfDNMT2::PTP cytoplasmic fraction.

R: replicate, P: probability

Gene ID	Protein description	IP R1	IP R2	Control IP R1	Control IP R2	P	FDR
PF3D7_0727300	DNA_(cytosine-5)-methyltransferase	82	70	0	0	1	0
PF3D7_0510100	conserved_protein,_unknown_function	43	37	18	19	1	0
PF3D7_1036900	conserved_Plasmodium_protein,_unknown_function	22	25	12	6	1	0
PF3D7_0906600	zinc_finger_protein,_putative	32	26	15	13	0.998	0.0008
PF3D7_1022000	RNA-binding_protein,_putative	17	18	6	2	0.998	0.0008
PF3D7_1419400	conserved_Plasmodium_membrane_protein,_unknown_function	38	26	22	14	0.996	0.0013
PF3D7_1132000	ubiquitin-like_protein,_putative	22	11	4	3	0.9955	0.0018
PF3D7_1019000	eukaryotic_translation_initiation_factor_subunit_eIF2A,_putative	27	18	13	7	0.993	0.0024
PF3D7_0730900	EMP1-trafficking_protein	9	11	1	0	0.9925	0.003
PF3D7_1407900	plasmepsin_I	23	21	8	9	0.986	0.0041
PF3D7_1408100	plasmepsin_III	24	20	10	9	0.984	0.0052
PF3D7_0301600	Plasmodium_exported_protein_(hyp1),_unknown_function	15	15	5	5	0.973	0.007
PF3D7_1008900	adenylate_kinase	9	6	0	2	0.971	0.0087
PF3D7_1463300	DNA_polymerase_alpha_subunit_B,_putative	7	3	1	0	0.9085	0.0146
PF3D7_0313000	conserved_Plasmodium_protein,_unknown_function	15	11	6	6	0.8855	0.0213
PF3D7_1017300	golgi_re-assembly_stacking_protein_1,_golgi_re-assembly_stacking_protein_2	3	9	0	0	0.8615	0.0286
PF3D7_0702500	Plasmodium_exported_protein,_unknown_function	9	6	2	2	0.859	0.0352
PF3D7_1035700	duffy_binding-like_merozoite_surface_protein	9	6	3	3	0.8475	0.0417
PF3D7_1116300	peptidyl-prolyl_cis-trans_isomerase	6	4	2	1	0.8175	0.0491
PF3D7_1474900	trailer_hitch_homolog,_putative	4	3	1	0	0.8115	0.0561
PF3D7_1343100	conserved_Plasmodium_protein,_unknown_function	4	2	0	0	0.808	0.0626
PF3D7_1317200	AP2_domain_transcription_factor_AP2-G3,_putative	4	4	1	0	0.801	0.0688
PF3D7_1119400	ubiquitin-protein_ligase,_putative	5	5	1	2	0.787	0.075
PF3D7_0522100	conserved_Plasmodium_protein,_unknown_function	5	3	2	0	0.78	0.0811
PF3D7_0215100	RING_zinc_finger_protein,_putative	4	3	0	1	0.7755	0.0868
PF3D7_1231800	asparagine-rich_protein,_putative	20	16	13	10	0.7675	0.0924
PF3D7_1111900	Josephin_domain-containing_protein,_putative	6	4	2	1	0.739	0.0987
PF3D7_0212100	conserved_Plasmodium_protein,_unknown_function	4	3	0	0	0.734	0.1046
PF3D7_0924400	conserved_Plasmodium_protein,_unknown_function	6	4	1	3	0.719	0.1107
PF3D7_1238700	BTB/POZ_domain-containing_protein,_putative	4	5	1	2	0.717	0.1165
PF3D7_1426200	protein_arginine_N-methyltransferase_1	4	2	0	1	0.713	0.122

PF3D7_1002100	EMP1-trafficking_protein	4	2	0	0	0.7055	0.1274
PF3D7_1366900	conserved_protein,_unknown_function	5	5	2	2	0.704	0.1325
PF3D7_1304500	small_heat_shock_protein,_putative	3	3	0	1	0.694	0.1376
PF3D7_0323800	conserved_Plasmodium_protein,_unknown_function	8	2	0	0	0.679	0.1428
PF3D7_1312700	conserved_Plasmodium_protein,_unknown_function	3	2	0	0	0.671	0.148
PF3D7_1318300	conserved_Plasmodium_protein,_unknown_function	5	4	2	2	0.648	0.1535
PF3D7_1239200	AP2_domain_transcription_factor,_putative	25	11	14	10	0.611	0.1597
PF3D7_1445700	conserved_Plasmodium_protein,_unknown_function	2	3	0	1	0.607	0.1657
PF3D7_0314700	RING_finger_protein_RNF1	24	14	13	12	0.5975	0.1716
PF3D7_1420600	pantothenate_kinase_1,_putative	4	3	0	3	0.5945	0.1773
PF3D7_1134600	zinc_finger_protein,_putative	3	4	1	0	0.594	0.1827
PF3D7_0702400	small_exported_membrane_protein_1	7	3	1	4	0.585	0.1882
PF3D7_0811300	CCR4-associated_factor_1	14	10	8	9	0.583	0.1934
PF3D7_0933900	conserved_Plasmodium_protein,_unknown_function	4	3	1	1	0.579	0.1984
PF3D7_1417900	ATP_synthase-associated_protein,_putative	2	2	0	0	0.556	0.2037
PF3D7_0704400	phosphoinositide-binding_protein,_putative	10	3	3	3	0.55	0.209
PF3D7_1235300	CCR4-NOT_transcription_complex_subunit_4,_putative	6	3	3	1	0.544	0.2141
PF3D7_1233200	conserved_Plasmodium_protein,_unknown_function	3	3	1	0	0.5365	0.2192
PF3D7_0513300	purine_nucleoside_phosphorylase	4	1	0	1	0.535	0.2241
PF3D7_0905800	conserved_Plasmodium_protein,_unknown_function	3	2	0	0	0.532	0.2289
PF3D7_1415400	conserved_Plasmodium_protein,_unknown_function	3	2	0	0	0.53	0.2336
PF3D7_1325200	lactate_dehydrogenase,_putative	4	3	1	2	0.524	0.2381
PF3D7_1249500	protein_CutA,_putative	3	1	0	0	0.4885	0.2432
PF3D7_1329100	myosin_F,_putative	13	11	9	10	0.4725	0.2484
PF3D7_0422300	alpha_tubulin_2	4	1	2	0	0.457	0.2536
PF3D7_0414000	structural_maintenance_of_chromosomes_protein_3,_putative	4	2	1	2	0.4565	0.2587
PF3D7_0315400	conserved_Plasmodium_protein,_unknown_function	2	1	0	0	0.4505	0.2637
PF3D7_1033400	haloacid_dehalogenase-like_hydrolase	2	3	1	1	0.44	0.2687
PF3D7_1134000	heat_shock_protein_70	22	23	16	19	0.4375	0.2736
PF3D7_1435700	ataxin-2_like_protein,_putative	4	2	1	2	0.424	0.2786
PF3D7_1236100	clustered-asparagine-rich_protein	17	11	12	7	0.4025	0.2837
PF3D7_0619500	acyl-CoA_synthetase	6	7	2	6	0.3965	0.2888
PF3D7_0809200	asparagine-rich_antigen_Pfa55-14	6	1	3	1	0.389	0.2939
PF3D7_0623100	nuclear_polyadenylated_RNA-binding_protein_NAB2,_putative	8	2	4	3	0.3875	0.2988
PF3D7_0503200	conserved_Plasmodium_protein,_unknown_function	3	1	1	0	0.387	0.3035

PF3D7_0602600	SAC3_domain-containing_protein,_putative	1	4	1	1	0.378	0.3083
PF3D7_1331500	conserved_Plasmodium_protein,_unknown_function	4	0	0	0	0.3755	0.3129
PF3D7_0413700	lysine_decarboxylase-like_protein,_putative	3	1	2	0	0.367	0.3176
PF3D7_1451200	conserved_Plasmodium_protein,_unknown_function	4	0	1	1	0.3545	0.3222
PF3D7_1206500	Tat_binding_protein_1(TBP-1)-interacting_protein,_putative	2	1	0	1	0.348	0.3269
PF3D7_1468700	eukaryotic_initiation_factor_4A	45	45	35	28	0.3445	0.3315
PF3D7_0317500	kinesin-5	3	1	1	1	0.342	0.3359
PF3D7_1434100	queuine_tRNA-ribosyltransferase,_putative	3	3	1	2	0.3365	0.3404
PF3D7_1446200	M17_leucyl_aminopeptidase	11	10	6	9	0.322	0.3449
PF3D7_1248700	conserved_Plasmodium_protein,_unknown_function	4	4	3	2	0.319	0.3493
PF3D7_1428100	WW_domain-binding_protein_11,_putative	2	1	0	0	0.3175	0.3536
PF3D7_0817300	conserved_Plasmodium_protein,_unknown_function	17	9	13	9	0.313	0.3579
PF3D7_0832200	Plasmodium_exported_protein_(PHISTa-like),_unknown_function	3	0	0	0	0.311	0.3621
PF3D7_1205800	high_mobility_group_protein_B3,_putative	4	0	2	1	0.3105	0.3662
PF3D7_0624600	SNF2_helicase,_putative	3	2	0	2	0.302	0.3743
PF3D7_1238100	calcyclin_binding_protein,_putative	4	1	1	2	0.302	0.3743
PF3D7_1110400	RNA-binding_protein,_putative	2	1	1	0	0.3005	0.3782
PF3D7_0804900	GTPase-activating_protein,_putative	9	10	7	5	0.2935	0.3821
PF3D7_1120000	conserved_protein,_unknown_function	3	0	0	1	0.2855	0.386
PF3D7_1107300	polyadenylate-binding_protein-interacting_protein_1,_putative	19	13	19	11	0.279	0.3899
PF3D7_0604500	conserved_Plasmodium_protein,_unknown_function	41	29	32	36	0.2785	0.3937
PF3D7_1023900	chromodomain-helicase-DNA-binding_protein_1_homolog,_putative	9	9	6	8	0.27	0.3975
PF3D7_0406200	sexual_stage-specific_protein_precursor	0	2	1	0	0.2545	0.4014
PF3D7_1359600	conserved_Plasmodium_protein,_unknown_function	49	41	43	39	0.2485	0.4053
PF3D7_1421500	mitochondrial_import_inner_membrane_translocase_subunit_TIM8,_putative	3	0	0	0	0.247	0.4091
PF3D7_0317800	26S_proteasome_non-ATPase_regulatory_subunit_9,_putative	20	18	12	16	0.2465	0.4129
PF3D7_0108300	conserved_Plasmodium_protein,_unknown_function	32	27	26	30	0.2425	0.4166
PF3D7_0301700	Plasmodium_exported_protein,_unknown_function	2	2	1	1	0.229	0.4204
PF3D7_0723300	conserved_protein,_unknown_function	2	0	0	1	0.221	0.4241
PF3D7_0526200	ADP-ribosylation_factor_GTPase-activating_protein,_putative	2	0	0	0	0.2085	0.428
PF3D7_1437900	HSP40,_subfamily_A	35	30	26	29	0.2075	0.4317
PF3D7_0726600	conserved_Plasmodium_protein,_unknown_function	2	0	0	0	0.206	0.4354

PF3D7_0106500	conserved_Plasmodium_protein,_unknown_function	2	0	0	1	0.205	0.4391
PF3D7_0420300	AP2_domain_transcription_factor,_putative	10	5	9	3	0.204	0.4426
PF3D7_0907200	GTPase-activating_protein,_putative	4	2	2	2	0.202	0.4461
PF3D7_0903500	nucleoporin_NUP138,_putative	2	0	1	0	0.2005	0.4496
PF3D7_0608700	T-complex_protein_1_subunit_zeta	16	19	13	16	0.198	0.453
PF3D7_0602000	zinc_finger_protein,_putative	2	0	0	1	0.1925	0.4564
PF3D7_1308700	conserved_Plasmodium_protein,_unknown_function	2	0	0	0	0.1915	0.4598
PF3D7_1140800	conserved_Plasmodium_protein,_unknown_function	9	4	5	5	0.1845	0.4631
PF3D7_1016300	GBP130_protein	6	3	4	4	0.184	0.4664
PF3D7_1306900	U1_small_nuclear_ribonucleoprotein_A,_putative	2	2	1	2	0.182	0.4697
PF3D7_1357000	elongation_factor_1-alpha	68	55	40	42	0.179	0.4729
PF3D7_1459600	conserved_Plasmodium_protein,_unknown_function	4	4	5	2	0.1765	0.4761
PF3D7_1330300	DnaJ_protein,_putative	1	2	1	1	0.1745	0.4793
PF3D7_1466400	AP2_domain_transcription_factor_AP2-EXP	4	6	5	4	0.172	0.4824
PF3D7_0220000	liver_stage_antigen_3	2	1	0	0	0.171	0.4854
PF3D7_0111800	eukaryotic_translation_initiation_factor_4E,_putative	3	4	3	3	0.1705	0.4885
PF3D7_0914900	BSD-domain_protein,_putative	21	18	17	20	0.169	0.4914
PF3D7_1244600	ADP-ribosylation_factor_GTPase-activating_protein	10	8	8	6	0.1655	0.4944
PF3D7_1229300	conserved_Plasmodium_protein,_unknown_function	10	11	11	8	0.1645	0.4973
PF3D7_0323700	U4/U6.U5_tri-snRNP-associated_protein_1,_putative	4	2	1	4	0.1635	0.5002
PF3D7_0922500	phosphoglycerate_kinase	7	8	7	4	0.1615	0.5058
PF3D7_0111000	kinesin-8,_putative	2	0	0	1	0.1615	0.5058
PF3D7_1035400	merozoite_surface_protein_3	1	2	1	1	0.16	0.5086
PF3D7_0102900	aspartate--tRNA_ligase	3	4	2	4	0.1595	0.5113
PF3D7_1132200	T-complex_protein_1_subunit_alpha	19	11	13	12	0.158	0.514
PF3D7_1146600	oocyst_rupture_protein_1,_putative	12	7	10	8	0.1565	0.5166
PF3D7_1410200	CTP_synthase	4	3	2	4	0.155	0.5219
PF3D7_0810800	hydroxymethyldihydropterin_pyrophosphokinase-dihydropteroate_synthase	2	3	2	2	0.155	0.5219
PF3D7_0922100	ubiquitin-like_protein,_putative	44	25	32	31	0.144	0.5271
PF3D7_1342900	AP2_domain_transcription_factor,_putative	4	0	0	0	0.144	0.5271
PF3D7_1122500	conserved_Plasmodium_protein,_unknown_function	2	0	1	0	0.143	0.5296
PF3D7_1007700	AP2_domain_transcription_factor_AP2-I	12	11	12	11	0.1425	0.5322
PF3D7_0104200	StAR-related_lipid_transfer_protein	11	13	10	8	0.1335	0.5347
PF3D7_1357800	T-complex_protein_1_subunit_delta	21	20	18	17	0.1315	0.5372
PF3D7_1010100	PI31_domain-containing_protein,_putative	2	3	1	3	0.13	0.5397
PF3D7_0424600	Plasmodium_exported_protein_(PHISTb),_unknown_function	3	3	3	2	0.13	0.5422

PF3D7_0610200	RNA-binding_protein_25,_putative	7	3	6	5	0.129	0.5446
PF3D7_0213100	protein_SIS1	7	6	5	5	0.1285	0.547
PF3D7_0920700	CRAL/TRIO_domain-containing_protein,_putative	3	3	2	4	0.1265	0.5494
PF3D7_1004300	E3_ubiquitin-protein_ligase,_putative	20	20	16	20	0.126	0.5518
PF3D7_1035300	glutamate-rich_protein_GLURP	2	1	2	0	0.125	0.5541
PF3D7_1139100	RNA-binding_protein,_putative	6	3	5	3	0.1215	0.5564
PF3D7_0803700	tubulin_gamma_chain	2	2	3	1	0.1195	0.5587
PF3D7_0417200	bifunctional_dihydrofolate_reductase-thymidylate_synthase	2	4	3	3	0.119	0.561
PF3D7_1305000	MCL1_domain-containing_protein,_putative	3	1	2	2	0.1185	0.5632
PF3D7_1218900	WD_repeat-containing_protein,_putative	2	3	3	2	0.117	0.5655
PF3D7_1123500	golgi_protein_2	4	1	4	1	0.1145	0.5677
PF3D7_1247500	serine/threonine_protein_kinase,_putative	9	3	7	6	0.114	0.572
PF3D7_1126400	mediator_of_RNA_polymerase_II_transcription_subunit_21,_putative	1	3	1	2	0.114	0.572
PF3D7_0105700	asparagine-rich_antigen_Pfa35-2	4	4	5	3	0.1125	0.5741
PF3D7_1408000	plasmepsin_II	30	27	20	25	0.1105	0.5783
PF3D7_1414100	zinc_finger_protein,_putative	2	0	1	1	0.1105	0.5783
PF3D7_1203500	threonylcarbamoyl-AMP_synthase,_putative	2	0	1	1	0.1095	0.5804
PF3D7_0929000	transcription_initiation_factor_TFIID_subunit_7,_putative	2	2	3	1	0.109	0.5824
PF3D7_1353100	Plasmodium_exported_protein,_unknown_function	12	6	6	8	0.108	0.5845
PF3D7_0607000	translation_initiation_factor_IF-2,_putative	3	4	4	3	0.1065	0.5865
PF3D7_1027300	peroxiredoxin	13	11	11	11	0.1045	0.5885
PF3D7_1355500	serine/threonine_protein_phosphatase_5	3	0	2	1	0.102	0.5904
PF3D7_0705500	inositol-phosphate_phosphatase,_putative	4	1	3	1	0.101	0.5924
PF3D7_0625400	conserved_Plasmodium_protein,_unknown_function	3	2	2	3	0.1005	0.5944
PF3D7_1206800	conserved_Plasmodium_protein,_unknown_function	23	17	20	22	0.099	0.5963
PF3D7_0806500	DnaJ_protein,_putative	1	2	2	1	0.0985	0.6001
PF3D7_0914400	conserved_protein,_unknown_function	2	1	2	1	0.0985	0.6001
PF3D7_1006700	conserved_Plasmodium_protein,_unknown_function	12	8	9	9	0.098	0.6019
PF3D7_0517300	serine/arginine-rich_splicing_factor_1	3	2	3	2	0.097	0.6038
PF3D7_1111200	conserved_protein,_unknown_function	2	2	3	1	0.0965	0.6056
PF3D7_1142600	60S_ribosomal_protein_L35ae,_putative	1	3	2	1	0.096	0.6074
PF3D7_1252300	conserved_Plasmodium_protein,_unknown_function	3	2	2	1	0.0955	0.6092
PF3D7_0202000	knob-associated_histidine-rich_protein	4	1	2	4	0.094	0.611
PF3D7_0515000	pre-mRNA-splicing_factor_CWC2,_putative	14	20	13	16	0.094	0.6127
PF3D7_1245100	kinesin-13,_putative	19	15	18	18	0.093	0.6179
PF3D7_0618500	malate_dehydrogenase	2	0	2	1	0.093	0.6179
PF3D7_1244200	RNA_polymerase_II_transcription_factor_B_subunit_2,_putative	2	0	2	0	0.093	0.6179

PF3D7_0416800	small_GTP-binding_protein_sar1	15	12	10	9	0.092	0.6213
PF3D7_0905900	coatomer_subunit_beta,_putative	4	3	1	6	0.092	0.6213
PF3D7_0202400	translation-enhancing_factor	4	2	4	2	0.0915	0.6229
PF3D7_1144000	40S_ribosomal_protein_S21	3	3	2	2	0.0915	0.6245
PF3D7_1119900	protein_transport_protein_SEC16,_putative	20	13	20	18	0.089	0.6262
PF3D7_0935900	ring-exported_protein_1	5	1	5	3	0.089	0.6278
PF3D7_1211400	heat_shock_protein_DNAJ_homologue_Pfj4	10	15	10	7	0.0885	0.6294
PF3D7_1205500	zinc_finger_protein,_putative	8	7	8	8	0.0875	0.631
PF3D7_1453800	glucose-6-phosphate_dehydrogenase-6-phosphogluconolactonase	5	3	5	4	0.086	0.6325
PF3D7_1124400	U6_snRNA-associated_Sm-like_protein_LSm1,_putative	4	4	3	3	0.085	0.6356
PF3D7_0206800	merozoite_surface_protein_2	3	2	4	1	0.085	0.6356
PF3D7_1107000	U6_snRNA-associated_Sm-like_protein_LSm4,_putative	2	0	1	1	0.0835	0.6372
PF3D7_0617200	conserved_protein,_unknown_function	5	1	3	4	0.083	0.6387
PF3D7_0214400	conserved_protein,_unknown_function	2	0	3	0	0.0775	0.6402
PF3D7_0422700	eukaryotic_initiation_factor_4A-III,_putative	2	4	3	4	0.077	0.6432
PF3D7_1026000	conserved_Plasmodium_protein,_unknown_function	2	0	2	1	0.077	0.6432
PF3D7_0302800	RNA-binding_protein,_putative	2	0	2	1	0.075	0.6447
PF3D7_1203200	signal_recognition_particle_subunit_SRP14	2	0	0	2	0.0745	0.6462
PF3D7_1473500	conserved_Plasmodium_protein,_unknown_function	4	1	4	3	0.0735	0.6477
PF3D7_0727900	conserved_Plasmodium_protein,_unknown_function	12	2	11	12	0.073	0.6492
PF3D7_0207400	serine_repeat_antigen_7	4	0	3	3	0.072	0.6506
PF3D7_1434300	Hsp70/Hsp90_organizing_protein	4	1	4	3	0.071	0.6521
PF3D7_0606000	conserved_Plasmodium_protein,_unknown_function	4	4	3	6	0.0705	0.6535
PF3D7_0524300	conserved_Plasmodium_protein,_unknown_function	6	6	6	6	0.07	0.6549
PF3D7_1361000	protein_arginine_N-methyltransferase_5,_putative	1	2	2	2	0.066	0.6563
PF3D7_1414800	small_nuclear_ribonucleoprotein-associated_protein_B,_putative	5	3	3	4	0.062	0.6578
PF3D7_1014600	transcriptional_coactivator_ADA2	2	3	4	3	0.0615	0.6606
PF3D7_0912800	tRNA_(adenine(58)-N(1))-methyltransferase_non-catalytic_subunit_TRM6,_putative	3	0	2	3	0.0615	0.6606
PF3D7_0621800	nascent_polypeptide-associated_complex_subunit_alpha,_putative	5	5	4	6	0.06	0.662
PF3D7_0819900	U6_snRNA-associated_Sm-like_protein_LSm3,_putative	3	2	2	2	0.058	0.6634
PF3D7_0420000	zinc_finger_protein,_putative	4	1	4	3	0.0565	0.6661
PF3D7_1325100	phosphoribosylpyrophosphate_synthetase	2	0	2	2	0.0565	0.6661
PF3D7_0813400	conserved_protein,_unknown_function	5	6	6	7	0.056	0.6702

PF3D7_0813100	conserved_Plasmodium_protein,_unknown_function	5	1	4	5	0.056	0.6702
PF3D7_0418700	RNA-binding_protein_NOB1,_putative	2	1	2	2	0.056	0.6702
PF3D7_0723800	conserved_Plasmodium_protein,_unknown_function	3	2	4	3	0.0545	0.6715
PF3D7_1021900	PHAX_domain-containing_protein,_putative	22	12	19	24	0.0535	0.6728
PF3D7_1468400	zinc_finger_protein,_putative	2	1	2	2	0.053	0.6741
PF3D7_0706500	conserved_Plasmodium_protein,_unknown_function	35	27	36	30	0.0525	0.6793
PF3D7_1129100	parasitophorous_vacuolar_protein_1	26	16	20	15	0.0525	0.6793
PF3D7_1471100	exported_protein_2	3	1	1	4	0.0525	0.6793
PF3D7_1328500	alpha/beta-hydrolase,_putative	3	1	4	1	0.0525	0.6793
PF3D7_0303000	N-ethylmaleimide-sensitive_fusion_protein	0	3	3	3	0.0515	0.6805
PF3D7_1331700	glutamine--tRNA_ligase,_putative	10	7	8	13	0.051	0.6818
PF3D7_1224300	polyadenylate-binding_protein_1,_putative	65	54	60	64	0.049	0.6843
PF3D7_1425900	conserved_Plasmodium_protein,_unknown_function	8	9	10	7	0.049	0.6843
PF3D7_1015600	heat_shock_protein_60	13	12	13	13	0.048	0.6855
PF3D7_0629400	polyadenylate-binding_protein_3,_putative	15	14	11	19	0.047	0.6867
PF3D7_0714000	histone_H2B_variant	2	2	1	3	0.0465	0.6879
PF3D7_1302000	EMP1-trafficking_protein	2	2	2	3	0.046	0.6891
PF3D7_1237500	conserved_Plasmodium_protein,_unknown_function	3	0	3	3	0.0455	0.6903
PF3D7_0402100	Plasmodium_exported_protein_(PHISTb),_unknown_function	3	3	5	3	0.045	0.6927
PF3D7_0615400	ribonuclease,_putative	3	0	3	1	0.045	0.6927
PF3D7_1230900	serine/threonine_protein_kinase_RIO1,_putative	2	0	3	1	0.044	0.6939
PF3D7_0106700	small_ribosomal_subunit_assembling_AARP2_protein	2	0	2	3	0.0435	0.695
PF3D7_1015900	enolase	8	7	5	10	0.043	0.6962
PF3D7_1242800	rab_specific_GDP_dissociation_inhibitor	1	2	2	4	0.043	0.6973
PF3D7_1035900	probable_protein,_unknown_function	2	0	3	1	0.0425	0.6985
PF3D7_1343700	kelch_protein_K13	6	3	5	7	0.042	0.7007
PF3D7_1420400	glycine--tRNA_ligase	1	2	2	3	0.042	0.7007
PF3D7_1228600	merozoite_surface_protein_9	22	14	21	21	0.042	0.7018
PF3D7_1453000	conserved_Plasmodium_protein,_unknown_function	10	6	8	13	0.04	0.7029
PF3D7_0807800	26S_proteasome_regulatory_subunit_RPN10,_putative	38	29	36	32	0.038	0.7073
PF3D7_0929200	RNA-binding_protein,_putative	21	15	15	15	0.038	0.7073
PF3D7_0309500	asparagine_synthetase_[glutamine-hydrolyzing],_putative	3	2	4	4	0.038	0.7073
PF3D7_0906500	arginase	2	1	3	2	0.038	0.7073
PF3D7_1035500	merozoite_surface_protein_6	4	2	5	3	0.0375	0.7084
PF3D7_0923900	polyadenylate-binding_protein_2,_putative	3	4	5	3	0.037	0.7094

PF3D7_1360400	conserved_Plasmodium_protein,_unknown_function	3	2	4	3	0.036	0.7105
PF3D7_1312900	eukaryotic_translation_initation_factor_4_gamma	3	1	3	3	0.0355	0.7115
PF3D7_0817500	histidine_triad_nucleotide-binding_protein_1	2	0	2	2	0.034	0.7126
PF3D7_1344300	zinc_finger_protein,_putative	2	0	3	2	0.0335	0.7136
PF3D7_1442300	tRNA_import_protein_tRIP	1	3	2	5	0.0325	0.7157
PF3D7_0110600	phosphatidylinositol-4-phosphate_5-kinase	3	1	4	3	0.0325	0.7157
PF3D7_0626800	pyruvate_kinase	16	15	14	21	0.032	0.7178
PF3D7_1227800	elongator_complex_protein_3,_putative	2	1	4	1	0.032	0.7178
PF3D7_0925900	parasitophorous_vacuolar_protein_5,_putative	14	12	12	10	0.0315	0.7188
PF3D7_1447800	calponin_homology_domain-containing_protein,_putative	7	4	8	5	0.0305	0.7198
PF3D7_0922200	S-adenosylmethionine_synthetase	32	34	30	36	0.0305	0.7218
PF3D7_1417200	NOT_family_protein,_putative	34	24	36	38	0.0305	0.7218
PF3D7_0621900	signal_recognition_particle_subunit_SRP68,_putative	9	4	9	11	0.0295	0.7228
PF3D7_1316600	choline-phosphate_cytidylyltransferase	1	2	2	4	0.029	0.7237
PF3D7_1460700	60S_ribosomal_protein_L27	7	5	5	6	0.029	0.7266
PF3D7_1340600	RNA_lariat_debranching_enzyme,_putative	2	2	3	5	0.029	0.7266
PF3D7_1241900	tetratricopeptide_repeat_protein,_putative	2	1	3	4	0.029	0.7266
PF3D7_0926100	protein_kinase,_putative	13	8	12	13	0.0285	0.7285
PF3D7_0110500	bromodomain_protein,_putative	3	0	4	3	0.0285	0.7285
PF3D7_1336800	nuclear_movement_protein,_putative	9	4	10	5	0.0275	0.7295
PF3D7_0828500	translation_initiation_factor_eIF-2B_subunit_alpha,_putative	4	3	3	6	0.0275	0.7304
PF3D7_1410600	eukaryotic_translation_initiation_factor_2_subunit_gamma,_putative	10	11	8	18	0.027	0.7313
PF3D7_0831700	heat_shock_protein_70	18	9	14	18	0.027	0.7341
PF3D7_0618700	trafficking_protein_particle_complex_subunit_6A,_putative	4	3	5	4	0.027	0.7341
PF3D7_1460500	conserved_Plasmodium_protein,_unknown_function	2	0	4	1	0.027	0.7341
PF3D7_0308200	T-complex_protein_1_subunit_eta	14	14	17	16	0.0265	0.7368
PF3D7_1241700	replication_factor_C_subunit_4,_putative	2	0	3	2	0.0265	0.7368
PF3D7_1323200	V-type_proton_ATPase_subunit_G,_putative	2	0	2	2	0.0265	0.7368
PF3D7_1324900	L-lactate_dehydrogenase	6	2	5	6	0.026	0.7377
PF3D7_0507800	conserved_protein,_unknown_function	17	6	19	20	0.0255	0.7394
PF3D7_1140100	V-type_proton_ATPase_subunit_F,_putative	3	1	2	4	0.0255	0.7394
PF3D7_1109900	60S_ribosomal_protein_L36	2	2	3	2	0.0255	0.7403
PF3D7_1412600	deoxyhypusine_synthase	2	1	5	2	0.025	0.7411
PF3D7_1402300	26S_proteasome_regulatory_subunit_RPN6	15	17	20	20	0.024	0.7437

PF3D7_1006800	single-strand_telomeric_DNA-binding_protein_GBP2,_putative	13	10	10	10	0.024	0.7437
PF3D7_0320800	ATP-dependent_RNA_helicase_DDX6	3	1	4	4	0.024	0.7437
PF3D7_1028400	nucleolar_preribosomal_assembly_protein,_putative	3	1	3	5	0.0235	0.7445
PF3D7_1407800	plasmepsin_IV	24	17	18	20	0.023	0.7454
PF3D7_1031600	conserved_Plasmodium_protein,_unknown_function	10	5	12	12	0.0225	0.7462
PF3D7_0810600	ATP-dependent_RNA_helicase_DBP1,_putative	8	8	11	11	0.022	0.7479
PF3D7_0903200	ras-related_protein_RAB7	3	2	4	3	0.022	0.7479
PF3D7_1213800	proline--tRNA_ligase	9	9	10	12	0.0215	0.7503
PF3D7_1032100	mRNA-decapping_enzyme_subunit_1,_putative	8	7	8	12	0.0215	0.7503
PF3D7_0719000	conserved_protein,_unknown_function	9	5	5	7	0.0215	0.7503
PF3D7_1309100	60S_ribosomal_protein_L24,_putative	4	4	6	4	0.021	0.7511
PF3D7_1201000	Plasmodium_exported_protein_(PHISTb),_unknown_function	3	3	7	3	0.0205	0.7519
PF3D7_1115400	cysteine_proteinase_falcipain_3	3	2	3	5	0.02	0.7543
PF3D7_0508800	single-stranded_DNA-binding_protein	2	0	4	2	0.02	0.7543
PF3D7_1446600	centrin-2	2	0	3	2	0.02	0.7543
PF3D7_1130400	26S_protease_regulatory_subunit_6A,_putative	26	26	28	25	0.02	0.7551
PF3D7_0931800	proteasome_subunit_beta_type-6,_putative	4	4	5	5	0.0195	0.7559
PF3D7_0607700	conserved_Plasmodium_protein,_unknown_function	2	1	5	2	0.0195	0.7566
PF3D7_0922600	glutamine_synthetase,_putative	3	4	7	5	0.019	0.7582
PF3D7_1468800	splicing_factor_U2AF_large_subunit,_putative	4	2	5	7	0.019	0.7582
PF3D7_1462800	glyceraldehyde-3-phosphate_dehydrogenase	13	12	11	15	0.0185	0.7612
PF3D7_1450100	signal_recognition_particle_subunit_SRP54	10	9	12	11	0.0185	0.7612
PF3D7_1465200	mediator_of_RNA_polymerase_II_transcription_subunit_4,_putative	3	3	5	4	0.0185	0.7612
PF3D7_0512000	prefoldin_subunit_6,_putative	0	2	1	3	0.0185	0.7612
PF3D7_0627800	acetyl-CoA_synthetase,_putative	15	16	16	23	0.018	0.7627
PF3D7_0710600	60S_ribosomal_protein_L34	2	1	2	3	0.018	0.7627
PF3D7_0823200	RNA-binding_protein,_putative	6	9	9	10	0.0175	0.7656
PF3D7_1136400	signal_recognition_particle_subunit_SRP72,_putative	3	6	8	8	0.0175	0.7656
PF3D7_1329500	conserved_protein,_unknown_function	2	5	8	6	0.0175	0.7656
PF3D7_1115700	cysteine_proteinase_falcipain_2a	4	3	6	6	0.0175	0.7656
PF3D7_0525100	acyl-CoA_synthetase	5	5	10	6	0.017	0.7677
PF3D7_0315100	eukaryotic_translation_initiation_factor_4E	1	2	4	2	0.017	0.7677
PF3D7_1034900	methionine--tRNA_ligase	2	1	4	3	0.017	0.7677
PF3D7_1404900	conserved_Plasmodium_protein,_unknown_function	3	4	5	6	0.0165	0.7684
PF3D7_1438600	conserved_protein,_unknown_function	6	7	9	8	0.016	0.7705

PF3D7_0322000	peptidyl-prolyl_cis-trans_isomerase	2	2	3	3	0.016	0.7705
PF3D7_1346500	conserved_Plasmodium_protein,_unknown_function	2	0	5	2	0.016	0.7705
PF3D7_0319100	E3_ubiquitin-protein_ligase_RBX1,_putative	2	2	2	4	0.0155	0.7712
PF3D7_1360900	RNA-binding_protein,_putative	27	20	24	28	0.015	0.7739
PF3D7_1011700	DNA_repair_protein_RAD23,_putative	8	7	11	9	0.015	0.7739
PF3D7_1426100	transcription_factor_BTF3,_putative	3	2	3	4	0.015	0.7739
PF3D7_1436300	translocon_component_PTEX150	1	2	6	3	0.015	0.7739
PF3D7_1004000	60S_ribosomal_protein_L13,_putative	8	6	8	8	0.0145	0.7759
PF3D7_0821700	60S_ribosomal_protein_L22,_putative	3	4	3	6	0.0145	0.7759
PF3D7_1130100	60S_ribosomal_protein_L38	3	3	3	4	0.0145	0.7759
PF3D7_0501200	parasite-infected_erythrocyte_surface_protein	5	4	6	7	0.0145	0.7778
PF3D7_1217900	PPPDE_peptidase_domain-containing_protein,_putative	4	2	6	6	0.0145	0.7778
PF3D7_1126900	small_nuclear_ribonucleoprotein_F,_putative	3	1	3	3	0.0145	0.7778
PF3D7_1139300	AP2_domain_transcription_factor,_putative	18	13	23	20	0.014	0.7791
PF3D7_1466800	conserved_Plasmodium_protein,_unknown_function	7	4	8	9	0.014	0.7791
PF3D7_1424400	60S_ribosomal_protein_L7-3,_putative	12	11	12	12	0.0135	0.7817
PF3D7_1020900	ADP-ribosylation_factor	8	7	8	7	0.0135	0.7817
PF3D7_0707700	E3_ubiquitin-protein_ligase,_putative	2	3	6	5	0.0135	0.7817
PF3D7_1451000	conserved_Plasmodium_protein,_unknown_function	2	2	3	6	0.0135	0.7817
PF3D7_0320900	histone_H2A.Z	0	2	2	3	0.0135	0.7823
PF3D7_1229500	T-complex_protein_1_subunit_gamma	13	17	17	20	0.013	0.7829
PF3D7_1113400	ubiquitin_domain-containing_protein_DSK2,_putative	70	55	49	49	0.013	0.7841
PF3D7_0611800	conserved_Plasmodium_protein,_unknown_function	4	3	8	5	0.013	0.7841
PF3D7_1307700	TOM1-like_protein,_putative	3	1	5	5	0.0125	0.7854
PF3D7_1125500	small_nuclear_ribonucleoprotein_Sm_D1,_putative	2	1	4	3	0.0125	0.7854
PF3D7_0422500	pre-mRNA-splicing_helicase_BRR2,_putative	3	2	6	5	0.012	0.7872
PF3D7_1340300	nucleolar_complex_protein_2,_putative	2	1	4	4	0.012	0.7872
PF3D7_1461300	40S_ribosomal_protein_S28e,_putative	2	1	2	3	0.012	0.7872
PF3D7_0608800	ornithine_aminotransferase	3	4	6	5	0.012	0.7878
PF3D7_0909800	small_nuclear_ribonucleoprotein_Sm_D3,_putative	1	2	3	3	0.0115	0.789
PF3D7_0614400	pre-mRNA-splicing_factor_CWF7,_putative	2	0	4	2	0.0115	0.789
PF3D7_0527500	Hsc70-interacting_protein	39	24	42	44	0.011	0.7901
PF3D7_1213700	DNA-directed_RNA_polymerases_I,_II,_and_III_subunit_RPABC3,_putative	2	1	2	4	0.011	0.7901
PF3D7_1453700	HSP90_co-chaperone_p23	27	23	27	29	0.011	0.7924
PF3D7_0320300	T-complex_protein_1_subunit_epsilon	12	10	13	16	0.011	0.7924

PF3D7_1432700	protein-L-isoaspartate(D-aspartate)_O-methyltransferase,_putative	7	8	8	9	0.011	0.7924
PF3D7_1128100	prefoldin_subunit_5,_putative	4	6	9	5	0.011	0.7924
PF3D7_0813300	conserved_protein,_unknown_function	7	3	6	8	0.0105	0.7969
PF3D7_1129800	conserved_Plasmodium_protein,_unknown_function	3	3	3	6	0.0105	0.7969
PF3D7_0614500	60S_ribosomal_protein_L19	3	3	5	4	0.0105	0.7969
PF3D7_1443000	serine/threonine_protein_kinase	6	2	8	10	0.0105	0.7969
PF3D7_0932300	M18_aspartyl_aminopeptidase	3	1	5	5	0.0105	0.7969
PF3D7_1008100	zinc_finger_protein,_putative	3	1	7	2	0.0105	0.7969
PF3D7_1224900	splicing_factor_3B_subunit_6,_putative	4	0	3	5	0.0105	0.7969
PF3D7_0403600	conserved_Plasmodium_protein,_unknown_function	2	0	5	5	0.0105	0.7969
PF3D7_0305200	conserved_Plasmodium_protein,_unknown_function	13	4	19	16	0.01	0.7974
PF3D7_1104000	phenylalanine--tRNA_ligase_beta_subunit	3	2	3	9	0.01	0.798
PF3D7_1108600	endoplasmic_reticulum-resident_calcium_binding_protein	16	5	19	10	0.0095	0.8017
PF3D7_0312800	60S_ribosomal_protein_L26,_putative	5	3	5	5	0.0095	0.8017
PF3D7_1316500	pre-mRNA-processing_factor_40,_putative	2	2	7	5	0.0095	0.8017
PF3D7_1136600	conserved_Plasmodium_protein,_unknown_function	2	1	5	5	0.0095	0.8017
PF3D7_1231100	ras-related_protein_Rab-2	2	1	3	4	0.0095	0.8017
PF3D7_0823300	histone_acetyltransferase_GCN5	2	0	4	3	0.0095	0.8017
PF3D7_1456500	conserved_Plasmodium_protein,_unknown_function	2	0	3	3	0.0095	0.8017
PF3D7_1358800	40S_ribosomal_protein_S15	8	8	7	10	0.009	0.8033
PF3D7_1414300	60S_ribosomal_protein_L10,_putative	4	5	8	6	0.009	0.8033
PF3D7_1473200	DnaJ_protein,_putative	3	2	7	4	0.009	0.8033
PF3D7_0802000	glutamate_dehydrogenase,_putative	11	14	17	20	0.0085	0.8058
PF3D7_0110400	DNA-directed_RNA_polymerase_II_subunit_RPB9,_putative	6	7	8	10	0.0085	0.8058
PF3D7_1010600	eukaryotic_translation_initiation_factor_2_subunit_beta	5	3	5	8	0.0085	0.8058
PF3D7_1003700	MKT1_domain-containing_protein,_putative	4	2	6	8	0.0085	0.8058
PF3D7_0903900	60S_ribosomal_protein_L32	2	2	3	5	0.0085	0.8058
PF3D7_0407800	conserved_Plasmodium_protein,_unknown_function	13	9	20	13	0.008	0.8068
PF3D7_1349600	conserved_Plasmodium_protein,_unknown_function	3	1	4	6	0.008	0.8068
PF3D7_0317600	40S_ribosomal_protein_S11,_putative	7	10	13	8	0.0075	0.8088
PF3D7_0813900	40S_ribosomal_protein_S16,_putative	3	5	6	5	0.0075	0.8088
PF3D7_1438900	thioredoxin_peroxidase_1	5	1	6	8	0.0075	0.8088
PF3D7_1345900	kinetochore_protein_SPC25,_putative	4	1	5	6	0.0075	0.8088
PF3D7_1008700	tubulin_beta_chain	42	33	43	49	0.007	0.8117
PF3D7_1306400	26S_protease_regulatory_subunit_10B,_putative	17	18	20	19	0.007	0.8117
PF3D7_0207700	serine_repeat_antigen_4	7	6	13	10	0.007	0.8117

PF3D7_1326400	translation_initiation_factor_eIF-2B_subunit_gamma,_putative	6	6	8	9	0.007	0.8117
PF3D7_1010700	dolichyl-phosphate-mannose--protein_mannosyltransferase,_putative	4	1	4	5	0.007	0.8117
PF3D7_1344900	conserved_Plasmodium_protein,_unknown_function	3	1	7	2	0.007	0.8117
PF3D7_1365900	ubiquitin-60S_ribosomal_protein_L40	7	6	8	5	0.0065	0.814
PF3D7_0519400	40S_ribosomal_protein_S24	4	5	8	3	0.0065	0.814
PF3D7_1361100	protein_transport_protein_Sec24A	8	2	11	12	0.0065	0.814
PF3D7_1002400	transformer-2_protein_homolog_beta,_putative	3	2	5	5	0.0065	0.814
PF3D7_0623600	transcription_or_splicing_factor-like_protein,_putative	2	1	3	7	0.0065	0.814
PF3D7_1456700	conserved_Plasmodium_protein,_unknown_function	7	9	9	15	0.006	0.815
PF3D7_0306100	conserved_Plasmodium_protein,_unknown_function	6	3	8	10	0.006	0.815
PF3D7_1117100	ubiquitin_carboxyl-terminal_hydrolase_UCH54	17	12	18	20	0.006	0.8154
PF3D7_1126000	threonine--tRNA_ligase	4	3	9	9	0.0055	0.8181
PF3D7_0404600	conserved_Plasmodium_membrane_protein,_unknown_function	6	2	7	13	0.0055	0.8181
PF3D7_1107400	DNA_repair_protein_RAD51	4	2	6	6	0.0055	0.8181
PF3D7_0802800	serine/threonine_protein_phosphatase_2B_catalytic_subunit_A	5	1	10	7	0.0055	0.8181
PF3D7_0805700	serine/threonine_protein_kinase,_FIKK_family	3	0	5	6	0.0055	0.8181
PF3D7_1136300	tudor_staphylococcal_nuclease	2	0	5	4	0.0055	0.8181
PF3D7_0703500	erythrocyte_membrane-associated_antigen	6	3	10	6	0.0055	0.8199
PF3D7_1358500	zinc_finger_protein,_putative	3	2	8	6	0.0055	0.8199
PF3D7_0512600	ras-related_protein_Rab-1B	2	2	5	3	0.0055	0.8199
PF3D7_1351400	60S_ribosomal_protein_L17,_putative	3	1	3	5	0.0055	0.8199
PF3D7_1353800	proteasome_subunit_alpha_type-4,_putative	5	8	10	9	0.005	0.8216
PF3D7_1308300	40S_ribosomal_protein_S27	6	3	5	5	0.005	0.8216
PF3D7_0102500	erythrocyte_binding_antigen-181	4	1	7	8	0.005	0.8216
PF3D7_0310300	phosphoglycerate_mutase,_putative	3	1	10	6	0.005	0.8216
PF3D7_0818900	heat_shock_protein_70	227	183	170	189	0.005	0.8225
PF3D7_0601900	conserved_Plasmodium_protein,_unknown_function	2	3	4	5	0.005	0.8225
PF3D7_1302800	40S_ribosomal_protein_S7,_putative	7	9	11	13	0.0045	0.8246
PF3D7_1142500	60S_ribosomal_protein_L28	6	7	8	7	0.0045	0.8246
PF3D7_1468900	zinc_finger_protein,_putative	3	5	9	8	0.0045	0.8246
PF3D7_0520300	U6_snRNA-associated_Sm-like_protein_LSm2,_putative	4	4	5	6	0.0045	0.8246
PF3D7_0627500	protein_DJ-1	3	1	4	7	0.0045	0.8246
PF3D7_1412500	actin_II	10	13	15	15	0.0045	0.825
PF3D7_0915400	ATP-dependent_6-phosphofructokinase	23	23	33	34	0.004	0.8314
PF3D7_0903700	alpha_tubulin_1	20	23	32	29	0.004	0.8314
PF3D7_0507600	protein_CAF40,_putative	16	12	22	24	0.004	0.8314
PF3D7_0517000	60S_ribosomal_protein_L12,_putative	13	9	13	18	0.004	0.8314

PF3D7_1409800	CUGBP_Elav-like_family_member_2,_putative	8	7	13	13	0.004	0.8314
PF3D7_0306900	40S_ribosomal_protein_S23,_putative	5	6	8	7	0.004	0.8314
PF3D7_1118200	heat_shock_protein_90,_putative	8	4	12	14	0.004	0.8314
PF3D7_1467600	conserved_Plasmodium_protein,_unknown_function	10	3	15	15	0.004	0.8314
PF3D7_0511300	MORN_repeat_protein,_putative	5	3	12	7	0.004	0.8314
PF3D7_0934500	V-type_proton_ATPase_subunit_E,_putative	3	2	5	8	0.004	0.8314
PF3D7_0217800	40S_ribosomal_protein_S26	3	2	5	4	0.004	0.8314
PF3D7_1473700	nucleoporin_NUP116/NSP116,_putative	4	1	6	9	0.004	0.8314
PF3D7_0629800	cullin-like_protein,_putative	3	1	8	7	0.004	0.8314
PF3D7_1317800	40S_ribosomal_protein_S19	2	1	3	6	0.004	0.8314
PF3D7_0801000	Plasmodium_exported_protein_(PHISTc),_unknown_function	3	0	4	6	0.004	0.8314
PF3D7_1033200	early_transcribed_membrane_protein_10.2	2	0	4	6	0.004	0.8314
PF3D7_1331800	60S_ribosomal_protein_L23,_putative	8	9	10	13	0.0035	0.8337
PF3D7_1103100	60S_acidic_ribosomal_protein_P1,_putative	10	7	11	13	0.0035	0.8337
PF3D7_0108000	proteasome_subunit_beta_type-3,_putative	4	4	7	7	0.0035	0.8337
PF3D7_0613500	AP-3_complex_subunit_beta,_putative	2	1	6	4	0.0035	0.8337
PF3D7_1004500	conserved_Plasmodium_protein,_unknown_function	3	0	5	4	0.0035	0.8337
PF3D7_1445200	ATP-dependent_RNA_helicase_MAK5,_putative	2	0	6	4	0.0035	0.8337
PF3D7_0826700	receptor_for_activated_c_kinase	15	11	18	16	0.003	0.8352
PF3D7_1441400	FACT_complex_subunit_SSRP1,_putative	3	3	8	8	0.003	0.8352
PF3D7_0218500	small_nuclear_ribonucleoprotein_Sm_D2,_putative	3	2	4	5	0.003	0.8352
PF3D7_1250300	vacuolar_protein_sorting-associated_protein_26,_putative	2	1	6	4	0.003	0.8352
PF3D7_0306800	T-complex_protein_1_subunit_beta	19	17	20	26	0.003	0.837
PF3D7_0518300	proteasome_subunit_beta_type-1,_putative	10	4	11	11	0.003	0.837
PF3D7_0913200	elongation_factor_1-beta	2	1	4	6	0.003	0.837
PF3D7_1346300	DNA/RNA-binding_protein_Alba_2	3	0	5	7	0.003	0.837
PF3D7_0602200	MYND-type_zinc_finger_protein,_putative	2	0	5	4	0.003	0.837
PF3D7_1424100	60S_ribosomal_protein_L5,_putative	14	14	20	19	0.0025	0.8412
PF3D7_1368100	26S_proteasome_regulatory_subunit_RPN11,_putative	12	10	15	15	0.0025	0.8412
PF3D7_1027800	60S_ribosomal_protein_L3	17	9	15	22	0.0025	0.8412
PF3D7_1129200	26S_proteasome_regulatory_subunit_RPN7,_putative	11	7	13	18	0.0025	0.8412
PF3D7_0312300	26S_proteasome_regulatory_subunit_RPN12,_putative	7	7	10	14	0.0025	0.8412
PF3D7_1209200	U6_snRNA-associated_Sm-like_protein_LSm7,_putative	6	6	8	6	0.0025	0.8412

PF3D7_0520200	mediator_of_RNA_polymerase_II_transcription_subunit_17,_putative	4	3	7	11	0.0025	0.8412
PF3D7_0410600	conserved_protein,_unknown_function	0	3	6	5	0.0025	0.8412
PF3D7_1235500	mRNA_methyltransferase,_putative	2	2	5	10	0.0025	0.8412
PF3D7_0511800	inositol-3-phosphate_synthase	0	2	3	7	0.0025	0.8412
PF3D7_1201500	GPN-loop_GTPase,_putative	3	1	8	7	0.0025	0.8412
PF3D7_0822300	small_nuclear_ribonucleoprotein_G,_putative	2	1	3	4	0.0025	0.8412
PF3D7_0728000	eukaryotic_translation_initiation_factor_2_subunit_alpha	4	3	6	7	0.0025	0.8416
PF3D7_1311500	26S_protease_regulatory_subunit_7,_putative	12	14	20	19	0.002	0.8459
PF3D7_0307200	60S_ribosomal_protein_L7,_putative	9	11	12	18	0.002	0.8459
PF3D7_1338200	60S_ribosomal_protein_L6,_putative	12	9	12	14	0.002	0.8459
PF3D7_1006200	DNA/RNA-binding_protein_Alba_3	9	7	10	13	0.002	0.8459
PF3D7_1431700	60S_ribosomal_protein_L14,_putative	8	5	11	11	0.002	0.8459
PF3D7_1426000	60S_ribosomal_protein_L21	5	5	5	9	0.002	0.8459
PF3D7_0822100	mediator_of_RNA_polymerase_II_transcription_subunit_7,_putative	5	4	10	8	0.002	0.8459
PF3D7_1225200	conserved_Plasmodium_protein,_unknown_function	3	4	13	7	0.002	0.8459
PF3D7_1205600	tetratricopeptide_repeat_protein,_putative	8	3	16	11	0.002	0.8459
PF3D7_0811500	histone-arginine_methyltransferase_CARM1,_putative	4	2	9	9	0.002	0.8459
PF3D7_0405100	protein_transport_protein_Sec24B,_putative	2	2	7	8	0.002	0.8459
PF3D7_0706000	importin-7,_putative	3	0	9	13	0.002	0.8459
PF3D7_0808100	AP-3_complex_subunit_delta,_putative	2	0	7	7	0.002	0.8459
PF3D7_1213900	conserved_Plasmodium_protein,_unknown_function	10	12	19	18	0.0015	0.8509
PF3D7_0516200	40S_ribosomal_protein_S11	10	12	15	14	0.0015	0.8509
PF3D7_1414000	26S_proteasome_regulatory_subunit_RPN13,_putative	7	9	11	10	0.0015	0.8509
PF3D7_0415900	60S_ribosomal_protein_L15,_putative	7	8	10	12	0.0015	0.8509
PF3D7_1030500	26S_proteasome_regulatory_subunit_RPN9,_putative	6	7	9	16	0.0015	0.8509
PF3D7_1347500	DNA/RNA-binding_protein_Alba_4	7	6	12	15	0.0015	0.8509
PF3D7_0516900	60S_ribosomal_protein_L2	6	6	11	11	0.0015	0.8509
PF3D7_1369400	conserved_Plasmodium_protein,_unknown_function	7	5	12	15	0.0015	0.8509
PF3D7_1242700	40S_ribosomal_protein_S17,_putative	7	5	7	11	0.0015	0.8509
PF3D7_1330800	RNA-binding_protein,_putative	3	3	9	8	0.0015	0.8509
PF3D7_1433400	zinc_finger_protein,_putative	0	2	2	2	0.0015	0.8509
PF3D7_1033100	S-adenosylmethionine_decarboxylase/ornithine_decarboxylase	3	1	7	9	0.0015	0.8509
PF3D7_0822900	conserved_Plasmodium_protein,_unknown_function	2	1	12	5	0.0015	0.8509
PF3D7_0213900	RTR1_domain-containing_protein,_putative	4	0	8	9	0.0015	0.8509

PF3D7_1143300	DNA-directed_RNA_polymerases_I_and_III_subunit_RPAC1,_putative	3	0	5	7	0.0015	0.8509
PF3D7_0516800	AP2_domain_transcription_factor_AP2-O2,_putative	2	0	5	7	0.0015	0.8509
PF3D7_0916700	RNA-binding_protein_musashi,_putative	28	28	34	46	0.001	0.8593
PF3D7_0708800	heat_shock_protein_110	31	26	40	38	0.001	0.8593
PF3D7_1248900	26S_protease_regulatory_subunit_8,_putative	16	16	22	22	0.001	0.8593
PF3D7_0507100	60S_ribosomal_protein_L4	13	11	24	21	0.001	0.8593
PF3D7_1408600	40S_ribosomal_protein_S8e,_putative	12	11	20	19	0.001	0.8593
PF3D7_0608500	proteasome_subunit_alpha_type-2,_putative	11	11	15	19	0.001	0.8593
PF3D7_0214000	T-complex_protein_1_subunit_theta	10	8	15	20	0.001	0.8593
PF3D7_1302100	gamete_antigen_27/25	8	6	12	10	0.001	0.8593
PF3D7_0618300	60S_ribosomal_protein_L27a,_putative	4	5	8	8	0.001	0.8593
PF3D7_1216300	signal_recognition_particle_subunit_SRP19	4	5	6	8	0.001	0.8593
PF3D7_1107800	AP2_domain_transcription_factor,_putative	10	4	20	14	0.001	0.8593
PF3D7_0406100	V-type_proton_ATPase_subunit_B	5	4	9	9	0.001	0.8593
PF3D7_1421200	40S_ribosomal_protein_S25	3	4	9	8	0.001	0.8593
PF3D7_1105100	histone_H2B	1	4	7	4	0.001	0.8593
PF3D7_0627700	transportin	7	3	15	14	0.001	0.8593
PF3D7_1412100	mini-chromosome_maintenance_complex-binding_protein,_putative	5	3	15	16	0.001	0.8593
PF3D7_1323400	60S_ribosomal_protein_L23	4	3	8	9	0.001	0.8593
PF3D7_1225700	VAC14_domain-containing_protein,_putative	5	2	11	13	0.001	0.8593
PF3D7_0716200	PDCD2_domain-containing_protein,_putative	3	2	9	9	0.001	0.8593
PF3D7_1458500	spindle_assembly_abnormal_protein_4,_putative	5	1	11	12	0.001	0.8593
PF3D7_0803800	proteasome_subunit_beta_type-4	4	1	9	11	0.001	0.8593
PF3D7_1133400	apical_membrane_antigen_1	4	1	8	7	0.001	0.8593
PF3D7_1359400	CUGBP_Elav-like_family_member_1	3	1	6	11	0.001	0.8593
PF3D7_1216900	DNA-binding_chaperone,_putative	2	1	8	9	0.001	0.8593
PF3D7_1367000	suppressor_of_kinetochore_protein_1,_putative	2	1	6	5	0.001	0.8593
PF3D7_1426800	conserved_Plasmodium_protein,_unknown_function	4	0	14	9	0.001	0.8593
PF3D7_1149000	antigen_332,_DBL-like_protein	3	0	19	17	0.001	0.8593
PF3D7_1364800	DNA-directed_RNA_polymerases_I,_II,_and_III_subunit_RPABC1,_putative	2	0	6	9	0.001	0.8593
PF3D7_1134200	conserved_Plasmodium_protein,_unknown_function	2	0	9	8	0.001	0.8593
PF3D7_0205900	26S_proteasome_regulatory_subunit_RPN1,_putative	29	30	41	43	0.0005	0.8694
PF3D7_1130200	60S_ribosomal_protein_P0	26	19	37	31	0.0005	0.8694

PF3D7_0413600	26S_protease_regulatory_subunit_6B,_putative	16	15	22	24	0.0005	0.8694
PF3D7_0721600	40S_ribosomal_protein_S5,_putative	17	13	29	25	0.0005	0.8694
PF3D7_0322900	40S_ribosomal_protein_S3A,_putative	14	13	25	21	0.0005	0.8694
PF3D7_1008400	26S_protease_regulatory_subunit_4,_putative	16	11	26	18	0.0005	0.8694
PF3D7_1311900	V-type_proton_ATPase_catalytic_subunit_A	15	11	24	24	0.0005	0.8694
PF3D7_1230700	protein_transport_protein_SEC13	10	11	16	22	0.0005	0.8694
PF3D7_1026800	40S_ribosomal_protein_S2	11	10	17	26	0.0005	0.8694
PF3D7_0812400	karyopherin_alpha	14	9	27	26	0.0005	0.8694
PF3D7_1353900	proteasome_subunit_alpha_type-7,_putative	8	9	10	13	0.0005	0.8694
PF3D7_1342000	40S_ribosomal_protein_S6	13	8	20	16	0.0005	0.8694
PF3D7_1105400	40S_ribosomal_protein_S4,_putative	10	8	17	17	0.0005	0.8694
PF3D7_0507700	nuclear_protein_localization_protein_4,_putative	9	6	12	18	0.0005	0.8694
PF3D7_1234800	splicing_factor_3B_subunit_3,_putative	8	6	15	21	0.0005	0.8694
PF3D7_1123900	13_kDa_ribonucleoprotein-associated_protein,_putative	5	6	13	11	0.0005	0.8694
PF3D7_0215700	DNA-directed_RNA_polymerase_II_subunit_RPB2,_putative	7	5	21	21	0.0005	0.8694
PF3D7_1323100	60S_ribosomal_protein_L6,_putative	5	5	9	9	0.0005	0.8694
PF3D7_1423700	conserved_Plasmodium_protein,_unknown_function	5	4	11	12	0.0005	0.8694
PF3D7_1223100	cAMP-dependent_protein_kinase_regulatory_subunit	4	4	9	10	0.0005	0.8694
PF3D7_0807500	proteasome_subunit_alpha_type-6,_putative	6	3	13	10	0.0005	0.8694
PF3D7_0309600	60S_acidic_ribosomal_protein_P2	6	3	10	8	0.0005	0.8694
PF3D7_1105000	histone_H4	4	3	6	7	0.0005	0.8694
PF3D7_1213200	mediator_of_RNA_polymerase_II_transcription_subunit_18,_putative	3	3	8	7	0.0005	0.8694
PF3D7_0620500	cleavage_stimulation_factor_subunit_1,_putative	0	3	7	10	0.0005	0.8694
PF3D7_1208900	protein_phosphatase_PPM11,_putative	7	2	16	17	0.0005	0.8694
PF3D7_1343000	phosphoethanolamine_N-methyltransferase	6	2	10	13	0.0005	0.8694
PF3D7_0811400	conserved_protein,_unknown_function	5	2	15	14	0.0005	0.8694
PF3D7_0309300	N2227-like_protein,_putative	3	2	11	14	0.0005	0.8694
PF3D7_0310500	ATP-dependent_RNA_helicase_DHX57,_putative	3	2	8	13	0.0005	0.8694
PF3D7_0520900	adenosylhomocysteinase	3	2	10	12	0.0005	0.8694
PF3D7_1247400	peptidyl-prolyl_cis-trans_isomerase_FKBP35	3	2	10	10	0.0005	0.8694
PF3D7_1103700	casein_kinase_II_beta_chain	2	2	6	8	0.0005	0.8694
PF3D7_1145100	coatomer_subunit_gamma,_putative	4	1	15	11	0.0005	0.8694
PF3D7_0801800	mannose-6-phosphate_isomerase,_putative	4	1	10	11	0.0005	0.8694

PF3D7_1120100	phosphoglycerate_mutase,_putative	3	1	7	8	0.0005	0.8694
PF3D7_1418000	ubiquitin_fusion_degradation_protein_1,_putative	3	1	6	7	0.0005	0.8694
PF3D7_1304900	DNA-directed_RNA_polymerase_II_subunit_RPB11,_putative	3	1	6	5	0.0005	0.8694
PF3D7_1414400	serine/threonine_protein_phosphatase_PP1	2	1	6	8	0.0005	0.8694
PF3D7_0209100	patatin-like_phospholipase,_putative	3	0	12	12	0.0005	0.8694
PF3D7_0930300	merozoite_surface_protein_1	103	88	211	200	0	0.9701
PF3D7_1246200	actin_I	82	73	77	89	0	0.9701
PF3D7_0826100	HECT-like_E3_ubiquitin_ligase,_putative	67	50	134	140	0	0.9701
PF3D7_0708400	heat_shock_protein_90	38	36	58	63	0	0.9701
PF3D7_1451100	elongation_factor_2	38	32	62	73	0	0.9701
PF3D7_1466300	26S_proteasome_regulatory_subunit_RPN2,_putative	29	31	56	55	0	0.9701
PF3D7_0524000	karyopherin_beta	34	29	55	66	0	0.9701
PF3D7_0619400	cell_division_cycle_protein_48_homologue,_putative	38	28	76	78	0	0.9701
PF3D7_1308200	carbamoyl_phosphate_synthetase	34	26	58	67	0	0.9701
PF3D7_0818200	14-3-3_protein	29	25	53	52	0	0.9701
PF3D7_1138500	protein_phosphatase_PPM2	23	19	59	67	0	0.9701
PF3D7_1338100	26S_proteasome_regulatory_subunit_RPN3,_putative	18	17	24	28	0	0.9701
PF3D7_1233600	asparagine_and_aspartate_rich_protein_1	20	16	75	84	0	0.9701
PF3D7_0917900	heat_shock_protein_70	20	16	40	42	0	0.9701
PF3D7_0422400	40S_ribosomal_protein_S19	17	16	29	26	0	0.9701
PF3D7_1011800	PRE-binding_protein	17	15	26	23	0	0.9701
PF3D7_1103800	CCR4-NOT_transcription_complex_subunit_1,_putative	12	15	38	41	0	0.9701
PF3D7_0500800	mature_parasite-infected_erythrocyte_surface_antigen	19	14	49	40	0	0.9701
PF3D7_0910100	exportin-7,_putative	12	13	25	28	0	0.9701
PF3D7_1335100	merozoite_surface_protein_7	18	12	25	27	0	0.9701
PF3D7_0214100	protein_transport_protein_SEC31	16	12	27	28	0	0.9701
PF3D7_1441200	60S_ribosomal_protein_L1,_putative	11	12	22	22	0	0.9701
PF3D7_0815200	importin_subunit_beta,_putative	10	12	32	29	0	0.9701
PF3D7_0207600	serine_repeat_antigen_5	19	11	25	27	0	0.9701
PF3D7_0707200	conserved_Plasmodium_protein,_unknown_function	16	11	35	29	0	0.9701
PF3D7_1017900	26S_proteasome_regulatory_subunit_p55,_putative	13	11	20	26	0	0.9701
PF3D7_0912900	26S_proteasome_regulatory_subunit_RPN8,_putative	13	11	21	25	0	0.9701
PF3D7_0307100	40S_ribosomal_protein_S12,_putative	13	10	24	17	0	0.9701
PF3D7_1126200	40S_ribosomal_protein_S18,_putative	7	10	18	13	0	0.9701
PF3D7_1447000	40S_ribosomal_protein_S5	13	9	21	18	0	0.9701
PF3D7_1465900	40S_ribosomal_protein_S3	11	9	23	17	0	0.9701
PF3D7_1341200	60S_ribosomal_protein_L18,_putative	11	9	18	16	0	0.9701

PF3D7_1203700	nucleosome_assembly_protein	8	9	20	26	0	0.9701
PF3D7_0814200	DNA/RNA-binding_protein_Alba_1	11	8	15	22	0	0.9701
PF3D7_1106000	RuvB-like_helicase_2	10	8	24	23	0	0.9701
PF3D7_0814000	60S_ribosomal_protein_L13-2,_putative	9	8	14	15	0	0.9701
PF3D7_0606600	conserved_Plasmodium_protein,_unknown_function	17	7	29	31	0	0.9701
PF3D7_1468100	kelch_domain-containing_protein,_putative	9	7	61	67	0	0.9701
PF3D7_1362200	RuvB-like_helicase_3	8	7	26	25	0	0.9701
PF3D7_0727400	proteasome_subunit_alpha_type-5,_putative	8	7	15	20	0	0.9701
PF3D7_0822600	protein_transport_protein_SEC23	8	6	19	22	0	0.9701
PF3D7_0317000	proteasome_subunit_alpha_type-3,_putative	7	6	13	14	0	0.9701
PF3D7_1454400	aminopeptidase_P	6	6	17	16	0	0.9701
PF3D7_0520000	40S_ribosomal_protein_S9,_putative	6	6	10	12	0	0.9701
PF3D7_1366300	conserved_Plasmodium_protein,_unknown_function	10	5	44	41	0	0.9701
PF3D7_1355100	DNA_replication_licensing_factor_MCM6	7	5	20	25	0	0.9701
PF3D7_0705400	DNA_replication_licensing_factor_MCM7	5	5	20	28	0	0.9701
PF3D7_0919000	nucleosome_assembly_protein	5	5	13	22	0	0.9701
PF3D7_0826500	ubiquitin_conjugation_factor_E4_B,_putative	5	5	22	18	0	0.9701
PF3D7_1144900	ras-related_protein_Rab-6	5	5	12	14	0	0.9701
PF3D7_1210600	conserved_Plasmodium_protein,_unknown_function	4	5	18	15	0	0.9701
PF3D7_1426700	phosphoenolpyruvate_carboxylase	9	4	19	21	0	0.9701
PF3D7_1417800	DNA_replication_licensing_factor_MCM2	8	4	24	30	0	0.9701
PF3D7_0316800	40S_ribosomal_protein_S15A,_putative	8	4	13	13	0	0.9701
PF3D7_1118500	nucleolar_protein_56,_putative	6	4	14	16	0	0.9701
PF3D7_0714200	conserved_Plasmodium_protein,_unknown_function	6	4	17	15	0	0.9701
PF3D7_1470900	proteasome_subunit_beta_type-2,_putative	5	4	11	15	0	0.9701
PF3D7_1317100	DNA_replication_licensing_factor_MCM4	4	4	45	37	0	0.9701
PF3D7_1474800	proteasome_subunit_alpha_type-1,_putative	4	4	9	15	0	0.9701
PF3D7_1003500	40S_ribosomal_protein_S20e,_putative	4	4	9	9	0	0.9701
PF3D7_0719700	40S_ribosomal_protein_S10,_putative	4	4	12	7	0	0.9701
PF3D7_0503400	actin-depolymerizing_factor_1	3	4	6	7	0	0.9701
PF3D7_1222300	endoplasmin,_putative	2	4	21	27	0	0.9701
PF3D7_1121100	conserved_protein,_unknown_function	9	3	37	33	0	0.9701
PF3D7_0405400	pre-mRNA-processing-splicing_factor_8,_putative	8	3	37	35	0	0.9701
PF3D7_0726500	ubiquitin_carboxyl-terminal_hydrolase,_putative	7	3	41	46	0	0.9701
PF3D7_1108700	heat_shock_protein_J2	7	3	17	17	0	0.9701
PF3D7_0719600	60S_ribosomal_protein_L11a,_putative	6	3	10	15	0	0.9701
PF3D7_1437200	ribonucleoside-diphosphate_reductase_large_subunit,_putative	6	3	14	14	0	0.9701

PF3D7_0611400	SWIB/MDM2_domain-containing_protein	5	3	15	21	0	0.9701
PF3D7_0704600	E3_ubiquitin-protein_ligase	5	3	21	17	0	0.9701
PF3D7_1145400	dynamin-like_protein	4	3	17	24	0	0.9701
PF3D7_0917000	merozoite_organizing_protein	4	3	14	17	0	0.9701
PF3D7_1230800	pre-mRNA-splicing_regulator,_putative	4	3	14	16	0	0.9701
PF3D7_0605100	RNA-binding_protein,_putative	4	3	18	14	0	0.9701
PF3D7_0511500	RNA_pseudouridylate_synthase,_putative	4	3	20	13	0	0.9701
PF3D7_1341300	60S_ribosomal_protein_L18-2,_putative	4	3	9	11	0	0.9701
PF3D7_0501600	rhoptry-associated_protein_2	4	3	8	10	0	0.9701
PF3D7_1012400	hypoxanthine-guanine_phosphoribosyltransferase	4	3	16	8	0	0.9701
PF3D7_1019400	60S_ribosomal_protein_L30e,_putative	4	3	7	8	0	0.9701
PF3D7_0209800	ATP-dependent_RNA_helicase_UAP56	3	3	15	19	0	0.9701
PF3D7_1003800	U5_small_nuclear_ribonucleoprotein_component,_putative	3	3	20	16	0	0.9701
PF3D7_1410400	rhoptry-associated_protein_1	9	2	16	20	0	0.9701
PF3D7_0302100	serine/threonine_protein_kinase	7	2	14	16	0	0.9701
PF3D7_0109200	cleavage_and_polyadenylation_specificity_factor_subunit_5,_putative	4	2	13	9	0	0.9701
PF3D7_1335700	conserved_oligomeric_Golgi_complex_subunit_3,_putative	3	2	15	19	0	0.9701
PF3D7_0527000	DNA_replication_licensing_factor_MCM3,_putative	3	2	14	19	0	0.9701
PF3D7_0302900	exportin-1,_putative	3	2	15	16	0	0.9701
PF3D7_1108400	casein_kinase_2,_alpha_subunit	2	2	17	18	0	0.9701
PF3D7_1338300	elongation_factor_1-gamma,_putative	2	2	6	12	0	0.9701
PF3D7_0716000	RNA-binding_protein,_putative	2	2	11	10	0	0.9701
PF3D7_0202600	nucleic_acid_binding_protein,_putative	9	1	25	24	0	0.9701
PF3D7_0815800	vacuolar_protein_sorting-associated_protein_9,_putative	6	1	17	17	0	0.9701
PF3D7_1218200	conserved_Plasmodium_protein,_unknown_function	3	1	27	25	0	0.9701
PF3D7_1012700	NLI_interacting_factor-like_phosphatase,_putative	3	1	28	19	0	0.9701
PF3D7_0905400	high_molecular_weight_rhoptry_protein_3	3	1	14	18	0	0.9701
PF3D7_1462100	conserved_Plasmodium_protein,_unknown_function	3	1	12	14	0	0.9701
PF3D7_0829000	conserved_Plasmodium_membrane_protein,_unknown_function	3	1	11	14	0	0.9701
PF3D7_1116700	dipeptidyl_aminopeptidase_1	3	1	9	12	0	0.9701
PF3D7_0621200	pyridoxine_biosynthesis_protein_PDX1	3	1	7	9	0	0.9701
PF3D7_1110500	vacuolar_protein_sorting-associated_protein_35,_putative	2	1	10	16	0	0.9701
PF3D7_1457300	MA3_domain-containing_protein,_putative	2	1	10	13	0	0.9701
PF3D7_1451900	ribosome_biogenesis_protein_TSR1,_putative	2	1	8	10	0	0.9701
PF3D7_1427900	leucine-rich_repeat_protein	2	1	6	7	0	0.9701
PF3D7_1466900	conserved_Plasmodium_protein,_unknown_function	1	1	27	31	0	0.9701

PF3D7_0102200	ring-infected_erythrocyte_surface_antigen	1	1	23	17	0	0.9701
PF3D7_0913600	conserved_Plasmodium_protein,_unknown_function	1	1	10	14	0	0.9701
PF3D7_1103600	actin-like_protein,_putative	1	1	6	14	0	0.9701
PF3D7_1130500	conserved_oligomeric_Golgi_complex_subunit_2,_putative	1	1	15	13	0	0.9701
PF3D7_0819000	conserved_Plasmodium_protein,_unknown_function	1	1	6	10	0	0.9701
PF3D7_0308900	splicing_factor_3B_subunit_1,_putative	1	1	7	9	0	0.9701
PF3D7_1011400	proteasome_subunit_beta_type-5	1	1	6	9	0	0.9701
PF3D7_1142100	conserved_Plasmodium_protein,_unknown_function	1	1	13	8	0	0.9701
PF3D7_1327400	conserved_Plasmodium_protein,_unknown_function	1	1	8	8	0	0.9701
PF3D7_0716800	eukaryotic_translation_initiation_factor_3_subunit_I,_putative	1	1	8	7	0	0.9701
PF3D7_0316500	kinetochore_protein_NUF2,_putative	1	1	7	7	0	0.9701
PF3D7_1134800	coatomer_subunit_delta	1	1	2	7	0	0.9701
PF3D7_0303200	HAD_superfamily_protein,_putative	1	1	9	6	0	0.9701
PF3D7_1417500	H/ACA_ribonucleoprotein_complex_subunit_4,_putative	1	1	6	6	0	0.9701
PF3D7_0923000	DNA-directed_RNA_polymerase_II_subunit_RPB3,_putative	1	1	5	6	0	0.9701
PF3D7_1459000	ATP-dependent_RNA_helicase_DBP5	1	1	5	6	0	0.9701
PF3D7_0212300	peptide_chain_release_factor_subunit_1,_putative	1	1	4	6	0	0.9701
PF3D7_0310700	trafficking_protein_particle_complex_subunit_4,_putative	1	1	4	6	0	0.9701
PF3D7_1408700	conserved_protein,_unknown_function	1	1	6	5	0	0.9701
PF3D7_0303100	CLP1_P-loop_domain-containing_protein,_putative	1	1	4	5	0	0.9701
PF3D7_1444800	fructose-bisphosphate_aldolase	1	1	6	4	0	0.9701
PF3D7_0625600	poly(A)_polymerase_PAP,_putative	1	1	4	4	0	0.9701
PF3D7_0904100	AP-4_complex_subunit_epsilon,_putative	1	1	2	4	0	0.9701
PF3D7_1229400	macrophage_migration_inhibitory_factor	1	1	2	4	0	0.9701
PF3D7_0815600	eukaryotic_translation_initiation_factor_3_subunit_G,_putative	1	1	6	3	0	0.9701
PF3D7_1117700	GTP-binding_nuclear_protein_RAN/TC4	1	1	5	3	0	0.9701
PF3D7_1250600	translation_initiation_factor_eIF-2B_subunit_beta,_putative	1	1	4	3	0	0.9701
PF3D7_1469800	conserved_Plasmodium_protein,_unknown_function	1	1	4	3	0	0.9701
PF3D7_1034000	Sec1_family_protein,_putative	1	1	3	3	0	0.9701
PF3D7_1363600	conserved_Plasmodium_protein,_unknown_function	1	1	3	3	0	0.9701
PF3D7_1451700	calcineurin_subunit_B	1	1	3	3	0	0.9701
PF3D7_1349200	glutamate--tRNA_ligase,_putative	1	1	2	3	0	0.9701
PF3D7_0107800	double-strand_break_repair_protein_MRE11	1	1	1	3	0	0.9701

PF3D7_1251500	ATP-dependent_RNA_helicase_DRS1,_putative	1	1	1	3	0	0.9701
PF3D7_0418500	trafficking_protein_particle_complex_subunit_3,_putative	1	1	3	2	0	0.9701
PF3D7_0721100	conserved_protein,_unknown_function	1	1	3	2	0	0.9701
PF3D7_1017100	rhoptry_neck_protein_12	1	1	3	2	0	0.9701
PF3D7_1029600	adenosine_deaminase	1	1	3	2	0	0.9701
PF3D7_0527600	conserved_Plasmodium_protein,_unknown_function	1	1	2	2	0	0.9701
PF3D7_0927600	RNA-binding_protein,_putative	1	1	2	2	0	0.9701
PF3D7_1416900	prefoldin_subunit_2,_putative	1	1	2	2	0	0.9701
PF3D7_0308000	DNA_polymerase_delta_small_subunit,_putative	1	1	1	2	0	0.9701
PF3D7_0309800	YTH_domain-containing_protein,_putative	1	1	1	2	0	0.9701
PF3D7_0525000	zinc_finger_protein,_putative	1	1	1	2	0	0.9701
PF3D7_0610400	histone_H3	1	1	1	2	0	0.9701
PF3D7_0617800	histone_H2A	1	1	1	2	0	0.9701
PF3D7_0723600	proteasome_assembly_chaperone_4,_putative	1	1	1	2	0	0.9701
PF3D7_1324700	SNARE_protein,_putative	1	1	3	1	0	0.9701
PF3D7_1441100	conserved_Plasmodium_protein,_unknown_function	1	1	3	1	0	0.9701
PF3D7_1202800	DNA/RNA-binding_protein,_putative	1	1	2	1	0	0.9701
PF3D7_0311100	pre-mRNA_splicing_factor,_putative	1	1	1	1	0	0.9701
PF3D7_0502000	vacuolar_protein_sorting-associated_protein_11,_putative	1	1	1	1	0	0.9701
PF3D7_1124900	60S_ribosomal_protein_L35,_putative	1	1	1	1	0	0.9701
PF3D7_1028700	merozoite_TRAP-like_protein	1	1	0	1	0	0.9701
PF3D7_1427500	DNA_mismatch_repair_protein_MSH2,_putative	1	1	0	1	0	0.9701
PF3D7_0708100	DNA-directed_RNA_polymerases_I,_II,_and_III_subunit_RPABC5,_putative	1	1	2	0	0	0.9701
PF3D7_0219700	Plasmodium_exported_protein_(PHISTc),_unknown_function	1	1	1	0	0	0.9701
PF3D7_0803100	U3_small_nucleolar_RNA-associated_protein_14,_putative	1	1	1	0	0	0.9701
PF3D7_0813000	conserved_protein,_unknown_function	1	1	1	0	0	0.9701
PF3D7_0924100	conserved_Plasmodium_protein,_unknown_function	1	1	1	0	0	0.9701
PF3D7_1227100	DNA_helicase_60	1	1	1	0	0	0.9701
PF3D7_1334800	MSP7-like_protein	1	1	1	0	0	0.9701
PF3D7_1446500	nucleoporin_NUP313,_putative	1	1	1	0	0	0.9701
PF3D7_0213700	conserved_protein,_unknown_function	1	1	0	0	0	0.9701
PF3D7_1031500	DEAD/DEAH_box_helicase,_putative	1	1	0	0	0	0.9701
PF3D7_1117900	conserved_Plasmodium_protein,_unknown_function	1	1	0	0	0	0.9701
PF3D7_1125800	kelch_domain-containing_protein,_putative	1	1	0	0	0	0.9701
PF3D7_1135400	conserved_Plasmodium_protein,_unknown_function	1	1	0	0	0	0.9701

PF3D7_1326100	WD_repeat-containing_protein,_putative	1	1	0	0	0	0.9701
PF3D7_1407300	pre-mRNA-splicing_factor_38B,_putative	1	1	0	0	0	0.9701
PF3D7_1416200	metacaspase-3,_putative	1	1	0	0	0	0.9701
PF3D7_1428900	conserved_Plasmodium_protein,_unknown_function	1	1	0	0	0	0.9701
PF3D7_1448100	conserved_Plasmodium_protein,_unknown_function	1	1	0	0	0	0.9701
PF3D7_0827900	protein_disulfide_isomerase	0	1	8	11	0	0.9701
PF3D7_1002700	conserved_Plasmodium_protein,_unknown_function	0	1	13	8	0	0.9701
PF3D7_0517700	eukaryotic_translation_initiation_factor_3_subunit_B,_putative	0	1	7	8	0	0.9701
PF3D7_1438500	cleavage_and_polyadenylation_specificity_factor_subunit_3,_putative	0	1	7	6	0	0.9701
PF3D7_1328100	proteasome_subunit_beta_type-7,_putative	0	1	4	6	0	0.9701
PF3D7_1224000	GTP_cyclohydrolase_1	0	1	3	6	0	0.9701
PF3D7_0525800	inner_membrane_complex_protein_1g,_putative	0	1	6	5	0	0.9701
PF3D7_1423300	serine/threonine_protein_phosphatase_7	0	1	4	5	0	0.9701
PF3D7_1463200	replication_factor_C_subunit_3,_putative	0	1	1	4	0	0.9701
PF3D7_1445900	ATP-dependent_RNA_helicase_DDX5,_putative	0	1	4	3	0	0.9701
PF3D7_0317200	cdc2-related_protein_kinase_4	0	1	3	3	0	0.9701
PF3D7_0718500	prefoldin_subunit_3,_putative	0	1	3	3	0	0.9701
PF3D7_1127900	conserved_Plasmodium_protein,_unknown_function	0	1	3	3	0	0.9701
PF3D7_1430300	acid_phosphatase,_putative	0	1	3	3	0	0.9701
PF3D7_1472000	pre-mRNA-splicing_factor_ISY1,_putative	0	1	1	3	0	0.9701
PF3D7_0412200	conserved_Plasmodium_protein,_unknown_function	0	1	4	2	0	0.9701
PF3D7_0218000	replication_factor_C_subunit_2,_putative	0	1	3	2	0	0.9701
PF3D7_0319600	elongation_factor_1-delta,_putative	0	1	2	2	0	0.9701
PF3D7_1219000	formin_2	0	1	2	2	0	0.9701
PF3D7_0804800	peptidyl-prolyl_cis-trans_isomerase	0	1	1	2	0	0.9701
PF3D7_0917600	pre-mRNA-splicing_factor_ATP-dependent_RNA_helicase_PRP43,_putative	0	1	1	2	0	0.9701
PF3D7_0503800	60S_ribosomal_protein_L31	0	1	1	1	0	0.9701
PF3D7_0603100	RNA-binding_protein,_putative	0	1	1	1	0	0.9701
PF3D7_0628200	eukaryotic_translation_initiation_factor_2-alpha_kinase	0	1	1	1	0	0.9701
PF3D7_1016200	Rab3_GTPase-activating_protein_non-catalytic_subunit,_putative	0	1	1	1	0	0.9701
PF3D7_1230000	TBC_domain-containing_protein,_putative	0	1	1	1	0	0.9701
PF3D7_1248200	pre-mRNA-splicing_factor_RBM22,_putative	0	1	1	1	0	0.9701
PF3D7_0926700	glutamine-dependent_NAD(+)_synthetase,_putative	0	1	0	1	0	0.9701
PF3D7_1315300	conserved_protein,_unknown_function	0	1	0	1	0	0.9701

PF3D7_1308400	conserved_Plasmodium_protein,_unknown_function	0	1	1	0	0	0.9701
PF3D7_1327300	conserved_Plasmodium_protein,_unknown_function	0	1	1	0	0	0.9701
PF3D7_0201800	knob_associated_heat_shock_protein_40	0	1	0	0	0	0.9701
PF3D7_0605600	nucleoside_diphosphate_kinase,_putative	0	1	0	0	0	0.9701
PF3D7_0912500	SAP_domain-containing_protein,_putative	0	1	0	0	0	0.9701
PF3D7_1120700	conserved_Plasmodium_protein,_unknown_function	0	1	0	0	0	0.9701
PF3D7_1311200	alternative_splicing_regulator,_putative	0	1	0	0	0	0.9701
PF3D7_1328000	conserved_Plasmodium_protein,_unknown_function	0	1	0	0	0	0.9701
PF3D7_1420700	surface_protein_P113	6	0	25	25	0	0.9701
PF3D7_1472200	histone_deacetylase,_putative	5	0	16	19	0	0.9701
PF3D7_1429800	coatomer_subunit_beta,_putative	5	0	14	12	0	0.9701
PF3D7_0615800	conserved_oligomeric_Golgi_complex_subunit_4,_putative	4	0	10	9	0	0.9701
PF3D7_0630600	conserved_protein,_unknown_function	3	0	7	17	0	0.9701
PF3D7_1118300	insulinase,_putative	3	0	14	11	0	0.9701
PF3D7_1363100	conserved_Plasmodium_protein,_unknown_function	2	0	11	13	0	0.9701
PF3D7_0918300	eukaryotic_translation_initiation_factor_3_subunit_F,_putative	2	0	8	8	0	0.9701
PF3D7_0318200	DNA-directed_RNA_polymerase_II_subunit_RPB1	1	0	28	27	0	0.9701
PF3D7_0505200	actin-like_protein,_putative	1	0	16	20	0	0.9701
PF3D7_1218500	dynamin-like_protein,_putative	1	0	12	20	0	0.9701
PF3D7_0505000	MMS19-like_protein,_putative	1	0	10	18	0	0.9701
PF3D7_1403100	condensin_complex_subunit_1,_putative	1	0	14	17	0	0.9701
PF3D7_1310800	tetratricopeptide_repeat_protein,_putative	1	0	16	16	0	0.9701
PF3D7_0211800	asparagine--tRNA_ligase	1	0	9	16	0	0.9701
PF3D7_0317300	conserved_Plasmodium_protein,_unknown_function	1	0	9	13	0	0.9701
PF3D7_0308600	pre-mRNA-processing_factor_19,_putative	1	0	8	12	0	0.9701
PF3D7_1008800	nucleolar_protein_5,_putative	1	0	11	11	0	0.9701
PF3D7_0903600	conserved_protein,_unknown_function,_unspecified_product	1	0	11	10	0	0.9701
PF3D7_0210200	conserved_Plasmodium_protein,_unknown_function	1	0	9	10	0	0.9701
PF3D7_0321500	peptidase,_putative	1	0	6	10	0	0.9701
PF3D7_0409600	replication_protein_A1,_large_subunit	1	0	11	9	0	0.9701
PF3D7_0918000	glideosome-associated_protein_50	1	0	10	9	0	0.9701
PF3D7_1347700	ethanolamine-phosphate_cytidylyltransferase	1	0	10	9	0	0.9701
PF3D7_0405700	lysine_decarboxylase,_putative	1	0	6	9	0	0.9701
PF3D7_1020700	N-acetyltransferase,_GNAT_family,_putative	1	0	5	9	0	0.9701

PF3D7_1344100	TLD_domain-containing_protein,_putative	1	0	8	7	0	0.9701
PF3D7_1309500	H/ACA_ribonucleoprotein_complex_subunit_1,_putative	1	0	7	7	0	0.9701
PF3D7_0613800	AP2_domain_transcription_factor,_putative	1	0	7	6	0	0.9701
PF3D7_0821600	polyribonucleotide_5'-hydroxyl-kinase_Clp1,_putative	1	0	7	6	0	0.9701
PF3D7_1004400	RNA-binding_protein,_putative	1	0	7	6	0	0.9701
PF3D7_0924700	splicing_factor_3A_subunit_3,_putative	1	0	6	6	0	0.9701
PF3D7_1406700	vacuolar_protein_sorting-associated_protein_29	1	0	5	6	0	0.9701
PF3D7_0608900	conserved_Plasmodium_protein,_unknown_function	1	0	4	6	0	0.9701
PF3D7_0719900	conserved_Plasmodium_membrane_protein,_unknown_function	1	0	9	5	0	0.9701
PF3D7_0704200	tRNA_m5C-methyltransferase,_putative	1	0	7	5	0	0.9701
PF3D7_0907700	proteasome_activator_28_subunit_beta,_putative	1	0	6	5	0	0.9701
PF3D7_0410800	conserved_Plasmodium_protein,_unknown_function	1	0	5	5	0	0.9701
PF3D7_1343900	U4/U6_small_nuclear_ribonucleoprotein_PRP4,_putative	1	0	5	5	0	0.9701
PF3D7_1356400	phosphatase_2A_regulatory_subunit-related_protein,_putative	1	0	5	5	0	0.9701
PF3D7_0807000	YEATS_domain-containing_protein,_putative	1	0	3	5	0	0.9701
PF3D7_0315800	zinc_finger_protein,_putative	1	0	6	4	0	0.9701
PF3D7_0822800	U5_small_nuclear_ribonucleoprotein_40_kDa_protein,_putative	1	0	5	4	0	0.9701
PF3D7_0711000	AAA_family_ATPase,_CDC48_subfamily	1	0	4	4	0	0.9701
PF3D7_1209400	cytosolic_iron-sulfur_protein_assembly_protein_1,_putative	1	0	4	4	0	0.9701
PF3D7_0310600	eukaryotic_translation_initiation_factor_3_subunit_K,_putative,_unspecified_product	1	0	3	4	0	0.9701
PF3D7_1236300	conserved_protein,_unknown_function	1	0	3	4	0	0.9701
PF3D7_0503300	serine/arginine-rich_splicing_factor_12	1	0	2	4	0	0.9701
PF3D7_0509800	phosphatidylinositol_4-kinase	1	0	2	4	0	0.9701
PF3D7_0704000	conserved_Plasmodium_membrane_protein,_unknown_function	1	0	2	4	0	0.9701
PF3D7_0912100	zinc_finger_protein,_putative	1	0	2	4	0	0.9701
PF3D7_1226600	proliferating_cell_nuclear_antigen_2	1	0	2	4	0	0.9701
PF3D7_1317300	conserved_Plasmodium_protein,_unknown_function	1	0	2	4	0	0.9701
PF3D7_1354300	large_subunit_rRNA_methyltransferase,_putative	1	0	2	4	0	0.9701
PF3D7_0624000	hexokinase	1	0	1	4	0	0.9701
PF3D7_1301600	erythrocyte_binding_antigen-140	1	0	1	4	0	0.9701
PF3D7_1355800	splicing_factor_3B_subunit_5,_putative	1	0	5	3	0	0.9701
PF3D7_1474000	conserved_Plasmodium_protein,_unknown_function	1	0	5	3	0	0.9701

PF3D7_0729300	60S_ribosomal_export_protein_NMD3,_putative	1	0	4	3	0	0.9701
PF3D7_1405100	GTPase-activating_protein,_putative	1	0	4	3	0	0.9701
PF3D7_0303300	DNA-directed_RNA_polymerases_I,_II,_and_III_subunit_RPABC2,_putative	1	0	3	3	0	0.9701
PF3D7_0913300	conserved_protein,_unknown_function	1	0	3	3	0	0.9701
PF3D7_1104200	chromatin_remodeling_protein	1	0	3	3	0	0.9701
PF3D7_0808400	coatomer_subunit_epsilon,_putative	1	0	2	3	0	0.9701
PF3D7_1331200	conserved_Plasmodium_protein,_unknown_function	1	0	2	3	0	0.9701
PF3D7_1419700	conserved_Plasmodium_protein,_unknown_function	1	0	2	3	0	0.9701
PF3D7_1420000	splicing_factor_3B_subunit_4,_putative	1	0	2	3	0	0.9701
PF3D7_0630500	ribosome_biogenesis_protein_YTM1,_putative	1	0	1	3	0	0.9701
PF3D7_0707600	mediator_of_RNA_polymerase_II_transcription_subunit_10,_putative	1	0	1	3	0	0.9701
PF3D7_0719300	actin-related_protein,_putative	1	0	5	2	0	0.9701
PF3D7_1205900	conserved_protein,_unknown_function	1	0	4	2	0	0.9701
PF3D7_0306200	activator_of_Hsp90_ATPase	1	0	3	2	0	0.9701
PF3D7_0414900	armadillo-domain_containing_rhoptry_protein	1	0	3	2	0	0.9701
PF3D7_0607600	spindle_assembly_abnormal_protein_6,_putative	1	0	3	2	0	0.9701
PF3D7_0618200	conserved_protein,_unknown_function	1	0	3	2	0	0.9701
PF3D7_1303400	LisH_domain-containing_protein,_putative	1	0	3	2	0	0.9701
PF3D7_1332800	eukaryotic_translation_initiation_factor_6,_putative	1	0	3	2	0	0.9701
PF3D7_0824600	Fe-S_cluster_assembly_protein_DRE2,_putative	1	0	2	2	0	0.9701
PF3D7_0916900	zinc_finger_protein,_putative	1	0	2	2	0	0.9701
PF3D7_1419300	glutathione_S-transferase	1	0	2	2	0	0.9701
PF3D7_0210100	60S_ribosomal_protein_L37ae,_putative	1	0	1	2	0	0.9701
PF3D7_0508000	6-cysteine_protein	1	0	1	2	0	0.9701
PF3D7_1217700	conserved_Plasmodium_protein,_unknown_function	1	0	1	2	0	0.9701
PF3D7_1313000	ubiquitin-like_protein_nedd8_homologue,_putative	1	0	1	2	0	0.9701
PF3D7_1320600	ras-related_protein_Rab-11A	1	0	1	2	0	0.9701
PF3D7_1360700	E3_SUMO-protein_ligase_PIAS,_putative	1	0	1	2	0	0.9701
PF3D7_0727800	cation_transporting_ATPase,_putative	1	0	0	2	0	0.9701
PF3D7_1127100	deoxyuridine_5'-triphosphate_nucleotidohydrolase	1	0	0	2	0	0.9701
PF3D7_1134700	DNA-directed_RNA_polymerase_I_subunit_RPA2,_putative	1	0	0	2	0	0.9701
PF3D7_1228900	conserved_Plasmodium_protein,_unknown_function	1	0	0	2	0	0.9701
PF3D7_1204300	eukaryotic_translation_initiation_factor_5A	1	0	4	1	0	0.9701

PF3D7_1206600	DNA-directed_RNA_polymerase_III_subunit_RPC2,_putative	1	0	3	1	0	0.9701
PF3D7_1442400	conserved_Plasmodium_protein,_unknown_function	1	0	3	1	0	0.9701
PF3D7_0723900	RNA-binding_protein,_putative	1	0	2	1	0	0.9701
PF3D7_0812700	U1_small_nuclear_ribonucleoprotein_C,_putative	1	0	2	1	0	0.9701
PF3D7_0831300	Plasmodium_exported_protein,_unknown_function	1	0	2	1	0	0.9701
PF3D7_1104700	DNA-directed_RNA_polymerase_III_subunit_RPC8,_putative	1	0	2	1	0	0.9701
PF3D7_1130700	structural_maintenance_of_chromosomes_protein_1,_putative	1	0	2	1	0	0.9701
PF3D7_0402000	Plasmodium_exported_protein_(PHISTa),_unknown_function	1	0	1	1	0	0.9701
PF3D7_0406400	cytosolic_glyoxalase_II	1	0	1	1	0	0.9701
PF3D7_0706400	60S_ribosomal_protein_L37	1	0	1	1	0	0.9701
PF3D7_0907300	prefoldin-like_protein,_putative	1	0	1	1	0	0.9701
PF3D7_0911200	conserved_Plasmodium_protein,_unknown_function	1	0	1	1	0	0.9701
PF3D7_0921000	ubiquitin-conjugating_enzyme_E2,_putative	1	0	1	1	0	0.9701
PF3D7_0923400	conserved_Plasmodium_protein,_unknown_function	1	0	1	1	0	0.9701
PF3D7_1030000	transcription_elongation_factor_SPT4,_putative	1	0	1	1	0	0.9701
PF3D7_1127600	CRAL/TRIO_domain-containing_protein,_putative	1	0	1	1	0	0.9701
PF3D7_1132800	aquaglyceroporin	1	0	1	1	0	0.9701
PF3D7_1332000	syntaxin,_Qa-SNARE_family	1	0	1	1	0	0.9701
PF3D7_1341900	V-type_proton_ATPase_subunit_D,_putative	1	0	1	1	0	0.9701
PF3D7_1440100	conserved_Plasmodium_protein,_unknown_function	1	0	1	1	0	0.9701
PF3D7_1466200	early_gametocyte_enriched_phosphoprotein_EGXP	1	0	1	1	0	0.9701
PF3D7_0104300	ubiquitin_carboxyl-terminal_hydrolase_1,_putative	1	0	0	1	0	0.9701
PF3D7_0423100	AP-4_complex_subunit_sigma,_putative	1	0	0	1	0	0.9701
PF3D7_1327000	conserved_Plasmodium_protein,_unknown_function	1	0	0	1	0	0.9701
PF3D7_1443800	pre-mRNA-splicing_factor_CWC24,_putative	1	0	0	1	0	0.9701
PF3D7_0411100	mediator_of_RNA_polymerase_II_transcription_subunit_8,_putative	1	0	3	0	0	0.9701
PF3D7_1440700	AP-3_complex_subunit_mu,_putative	1	0	3	0	0	0.9701
PF3D7_0927200	zinc_finger_protein,_putative	1	0	2	0	0	0.9701
PF3D7_1421000	DIX_domain-containing_protein,_putative	1	0	2	0	0	0.9701
PF3D7_0501100	heat_shock_protein_40,_type_II	1	0	1	0	0	0.9701
PF3D7_0504800	conserved_Plasmodium_protein,_unknown_function	1	0	1	0	0	0.9701

PF3D7_0624200	conserved_Plasmodium_protein,_unknown_function	1	0	1	0	0	0.9701
PF3D7_0709700	prodrug_activation_and_resistance_esterase	1	0	1	0	0	0.9701
PF3D7_0731500	erythrocyte_binding_antigen-175	1	0	1	0	0	0.9701
PF3D7_0933300	conserved_Plasmodium_protein,_unknown_function	1	0	1	0	0	0.9701
PF3D7_1027100	U3_small_nucleolar_ribonucleoprotein_protein_MPP10,_putative	1	0	1	0	0	0.9701
PF3D7_1320900	RNA-binding_protein,_putative	1	0	1	0	0	0.9701
PF3D7_1337900	conserved_Plasmodium_protein,_unknown_function	1	0	1	0	0	0.9701
PF3D7_1368900	conserved_protein,_unknown_function	1	0	1	0	0	0.9701
PF3D7_0222100	Pfmc-2TM_Maurer's_cleft_two_transmembrane_protein	1	0	0	0	0	0.9701
PF3D7_0311300	phosphatidylinositol_3-_and_4-kinase,_putative	1	0	0	0	0	0.9701
PF3D7_0316300	inorganic_pyrophosphatase,_inorganic_pyrophosphatase,_putative	1	0	0	0	0	0.9701
PF3D7_0425100	Plasmodium_exported_protein_(hyp6),_unknown_function	1	0	0	0	0	0.9701
PF3D7_0525400	7-helix-1_protein	1	0	0	0	0	0.9701
PF3D7_0532100	early_transcribed_membrane_protein_5	1	0	0	0	0	0.9701
PF3D7_0616000	pyridoxal_kinase	1	0	0	0	0	0.9701
PF3D7_0822200	phosphorylated_CTD_interacting_factor_1_WW_domain-containing_protein,_putative	1	0	0	0	0	0.9701
PF3D7_0830400	CRA_domain-containing_protein,_putative	1	0	0	0	0	0.9701
PF3D7_0831400	Plasmodium_exported_protein,_unknown_function	1	0	0	0	0	0.9701
PF3D7_0920800	inosine-5'-monophosphate_dehydrogenase	1	0	0	0	0	0.9701
PF3D7_0922700	pre-mRNA-splicing_factor_18,_putative	1	0	0	0	0	0.9701
PF3D7_0935600	gametocytogenesis-implicated_protein	1	0	0	0	0	0.9701
PF3D7_1327800	ribose-phosphate_pyrophosphokinase,_putative	1	0	0	0	0	0.9701
PF3D7_1334400	MSP7-like_protein	1	0	0	0	0	0.9701
PF3D7_1338900	serine/threonine_protein_kinase,_putativ e	1	0	0	0	0	0.9701
PF3D7_1358600	zinc_finger_protein,_putative	1	0	0	0	0	0.9701
PF3D7_1364200	nucleoporin_NUP205,_putative	1	0	0	0	0	0.9701
PF3D7_1402400	zinc_finger_protein,_putative	1	0	0	0	0	0.9701
PF3D7_1461600	splicing_factor_3B_subunit_2,_putative	1	0	0	0	0	0.9701
PF3D7_1473900	conserved_Plasmodium_protein,_unknown_function	1	0	0	0	0	0.9701

Table S2B. Proteomic data from IPs of PfDNMT2::PTP nuclear fraction.

R: replicate, P: probability

Gene ID	Protein description	IP R1	IP R2	IP R3	Control IP R1	Control IP R2	Control IP R3	P	FDR
PF3D7_0727300	DNA_(cytosine-5)-methyltransferase	94	48	49	0	0	0	1	0
PF3D7_0323800	conserved_Plasmodium_protein,_unknown_function	12	10	7	0	1	0	0.994	0.003
PF3D7_1022000	RNA-binding_protein,_putative	26	12	15	3	2	3	0.985	0.007
PF3D7_1015600	heat_shock_protein_60	117	73	74	14	25	11	0.984	0.009
PF3D7_0716300	conserved_protein,_unknown_function	6	5	5	0	0	0	0.981	0.011
PF3D7_1426000	60S_ribosomal_protein_L21	6	5	6	0	0	0	0.98	0.013
PF3D7_1239200	AP2_domain_transcription_factor,_putative	78	23	25	11	7	3	0.978	0.014
PF3D7_0517400	FACT_complex_subunit_SPT16,_putative	13	7	4	0	0	0	0.976	0.015
PF3D7_0307700	conserved_Plasmodium_protein,_unknown_function	21	6	9	1	2	0	0.962	0.018
PF3D7_0926100	protein_kinase,_putative	30	9	8	2	3	0	0.956	0.021
PF3D7_0817300	conserved_Plasmodium_protein,_unknown_function	20	7	13	1	3	0	0.952	0.023
PF3D7_0510100	conserved_protein,_unknown_function	48	18	18	6	11	2	0.942	0.028
PF3D7_1237500	conserved_Plasmodium_protein,_unknown_function	7	4	4	0	0	0	0.942	0.028
PF3D7_1419400	conserved_Plasmodium_membrane_protein,_unknown_function	27	9	9	2	2	1	0.941	0.032
PF3D7_1419700	conserved_Plasmodium_protein,_unknown_function	6	4	5	0	1	0	0.942	0.031
PF3D7_0927600	RNA-binding_protein,_putative	6	5	6	1	0	0	0.935	0.034
PF3D7_1456500	conserved_Plasmodium_protein,_unknown_function	21	7	9	1	4	0	0.925	0.037
PF3D7_0314700	RING_finger_protein_RNF1	17	5	4	1	1	0	0.919	0.041
PF3D7_1103100	60S_acidic_ribosomal_protein_P1,_putative	10	8	6	1	2	0	0.918	0.043
PF3D7_0812700	U1_small_nuclear_ribonucleoprotein_C,_putative	4	3	4	0	0	0	0.92	0.039
PF3D7_0212100	conserved_Plasmodium_protein,_unknown_function	9	8	5	0	1	1	0.912	0.046
PF3D7_1244600	ADP-ribosylation_factor_GTPase-activating_protein	11	9	7	1	2	1	0.909	0.048
PF3D7_1017300	golgi_re-assembly_stacking_protein_1,_golgi_re-assembly_stacking_protein_2	6	5	2	0	0	0	0.906	0.05
PF3D7_1031600	conserved_Plasmodium_protein,_unknown_function	7	3	4	0	1	0	0.904	0.053
PF3D7_1459600	conserved_Plasmodium_protein,_unknown_function	11	12	11	1	2	2	0.905	0.052
PF3D7_1358800	40S_ribosomal_protein_S15	13	7	7	2	2	0	0.904	0.055
PF3D7_0730900	EMP1-trafficking_protein	5	5	5	0	0	0	0.903	0.057

PF3D7_0301600	Plasmodium_exported_protein_(hyp1),_unknown_function	17	16	22	5	1	3	0.888	0.059
PF3D7_1139300	AP2_domain_transcription_factor,_putative	44	19	15	6	9	1	0.88	0.063
PF3D7_1215000	thioredoxin_peroxidase_2	3	4	3	0	0	0	0.881	0.061
PF3D7_1351400	60S_ribosomal_protein_L17,_putative	3	3	3	0	0	0	0.874	0.065
PF3D7_1474900	trailer_hitch_homolog,_putative	5	5	4	0	1	0	0.851	0.073
PF3D7_0503300	serine/arginine-rich_splicing_factor_12	3	4	3	0	0	0	0.846	0.075
PF3D7_0935600	gametocytogenesis-implicated_protein	3	3	7	0	0	0	0.871	0.067
PF3D7_1236100	clustered-asparagine-rich_protein	29	12	12	5	5	1	0.87	0.069
PF3D7_1342000	40S_ribosomal_protein_S6	19	10	11	4	5	0	0.854	0.071
PF3D7_0623100	nuclear_polyadenylated_RNA-binding_protein_NAB2,_putative	10	9	10	2	3	0	0.842	0.08
PF3D7_1134000	heat_shock_protein_70	93	66	73	32	39	18	0.846	0.077
PF3D7_1215300	10_kDa_chaperonin	3	3	2	0	0	0	0.839	0.082
PF3D7_0315800	zinc_finger_protein,_putative	4	4	3	0	1	0	0.817	0.087
PF3D7_1346300	DNA/RNA-binding_protein_Alba_2	3	2	2	0	0	0	0.802	0.091
PF3D7_0529400	conserved_Plasmodium_protein,_unknown_function	14	5	4	0	4	0	0.802	0.094
PF3D7_1027800	60S_ribosomal_protein_L3	18	13	12	6	6	0	0.795	0.103
PF3D7_0422400	40S_ribosomal_protein_S19	32	13	15	6	12	0	0.823	0.084
PF3D7_1242700	40S_ribosomal_protein_S17,_putative	7	5	3	1	1	0	0.812	0.089
PF3D7_0624600	SNF2_helicase,_putative	17	7	12	2	5	1	0.801	0.098
PF3D7_1205800	high_mobility_group_protein_B3,_putative	21	10	8	4	2	2	0.801	0.098
PF3D7_1006700	conserved_Plasmodium_protein,_unknown_function	10	3	7	1	2	0	0.795	0.101
PF3D7_1312600	2-oxoisovalerate_dehydrogenase_subunit_alpha,_mitochondrial,_putative	3	2	3	0	0	0	0.792	0.105
PF3D7_0402100	Plasmodium_exported_protein_(PHISTb),_unknown_function	5	6	6	1	1	0	0.785	0.107
PF3D7_1424400	60S_ribosomal_protein_L7-3,_putative	15	9	10	4	4	1	0.781	0.109
PF3D7_1209400	cytosolic_iron-sulfur_protein_assembly_protein_1,_putative	3	2	4	0	0	0	0.777	0.112
PF3D7_1431700	60S_ribosomal_protein_L14,_putative	9	3	8	1	2	0	0.767	0.116
PF3D7_0104300	ubiquitin_carboxyl-terminal_hydrolase_1,_putative	6	3	3	0	0	1	0.767	0.118
PF3D7_1130100	60S_ribosomal_protein_L38	6	4	3	1	1	0	0.766	0.12
PF3D7_1320500	SNARE_protein,_putative	7	2	1	0	0	0	0.752	0.128
PF3D7_0813000	conserved_protein,_unknown_function	19	4	4	1	0	2	0.769	0.114
PF3D7_1304500	small_heat_shock_protein,_putative	5	1	3	0	0	0	0.765	0.122

PF3D7_1404900	conserved_Plasmodium_protein,_unknown_function	37	22	26	13	15	7	0.755	0.124
PF3D7_1104100	syntaxin,_Qa-SNARE_family	6	2	2	1	0	0	0.755	0.126
PF3D7_1028400	nucleolar_preribosomal_assembly_protein,_putative	2	2	3	0	0	0	0.748	0.13
PF3D7_1308300	40S_ribosomal_protein_S27	9	3	4	1	2	0	0.745	0.133
PF3D7_1315500	conserved_protein,_unknown_function	2	2	2	0	0	0	0.739	0.136
PF3D7_0924400	conserved_Plasmodium_protein,_unknown_function	12	3	4	2	1	0	0.736	0.138
PF3D7_0519400	40S_ribosomal_protein_S24	8	6	5	1	3	0	0.741	0.135
PF3D7_1366900	conserved_protein,_unknown_function	5	6	8	0	3	0	0.733	0.142
PF3D7_1334800	MSP7-like_protein	3	1	3	0	0	0	0.734	0.14
PF3D7_1467600	conserved_Plasmodium_protein,_unknown_function	7	2	3	0	1	0	0.723	0.148
PF3D7_1331800	60S_ribosomal_protein_L23,_putative	12	5	7	3	3	0	0.732	0.144
PF3D7_1402400	zinc_finger_protein,_putative	4	1	2	0	0	0	0.709	0.157
PF3D7_0811300	CCR4-associated_factor_1	19	9	12	5	4	3	0.725	0.146
PF3D7_0610200	RNA-binding_protein_25,_putative	12	5	4	1	3	0	0.716	0.153
PF3D7_0604500	conserved_Plasmodium_protein,_unknown_function	49	27	35	15	20	5	0.713	0.155
PF3D7_1116300	peptidyl-prolyl_cis-trans_isomerase	12	5	4	1	4	0	0.707	0.159
PF3D7_1142100	conserved_Plasmodium_protein,_unknown_function	4	2	5	1	0	0	0.704	0.164
PF3D7_1338200	60S_ribosomal_protein_L6,_putative	11	7	7	1	6	0	0.719	0.15
PF3D7_0922100	ubiquitin-like_protein,_putative	42	25	27	13	15	7	0.701	0.167
PF3D7_0307200	60S_ribosomal_protein_L7,_putative	16	7	11	5	5	0	0.698	0.172
PF3D7_0905800	conserved_Plasmodium_protein,_unknown_function	9	2	8	0	3	0	0.694	0.175
PF3D7_1107800	AP2_domain_transcription_factor,_putative	38	12	6	1	8	0	0.686	0.18
PF3D7_1117300	conserved_protein,_unknown_function	3	2	3	0	1	0	0.718	0.152
PF3D7_0528200	eukaryotic_translation_initiation_factor_3_subunit_E,_putative	4	5	4	1	1	0	0.706	0.162
PF3D7_0602600	SAC3_domain-containing_protein,_putative	8	4	5	2	2	0	0.702	0.166
PF3D7_0516800	AP2_domain_transcription_factor_AP2-O2,_putative	18	2	3	0	0	0	0.699	0.169
PF3D7_1441400	FACT_complex_subunit_SSRP1,_putative	8	6	6	1	4	0	0.699	0.171
PF3D7_1327300	conserved_Plasmodium_protein,_unknown_function	5	2	2	0	0	0	0.695	0.174
PF3D7_0821700	60S_ribosomal_protein_L22,_putative	7	4	5	3	1	0	0.681	0.181
PF3D7_1445700	conserved_Plasmodium_protein,_unknown_function	3	2	1	0	0	0	0.68	0.183

PF3D7_1201000	Plasmodium_exported_protein_(PHISTb),_unknown_function	4	2	2	0	1	0	0.707	0.161
PF3D7_0201800	knob_associated_heat_shock_protein_40	2	2	2	0	0	0	0.691	0.177
PF3D7_1243600	translation_initiation_factor_SUI1,_putative	5	1	1	0	0	0	0.689	0.178
PF3D7_1309500	H/ACA_ribonucleoprotein_complex_subunit_1,_putative	2	1	2	0	0	0	0.68	0.184
PF3D7_1007900	eukaryotic_translation_initiation_factor_3_subunit_D,_putative	2	2	2	0	0	0	0.679	0.186
PF3D7_1016400	serine/threonine_protein_kinase,_FIKK_family	5	4	2	0	2	0	0.675	0.187
PF3D7_1011800	PRE-binding_protein	24	10	18	7	8	1	0.671	0.189
PF3D7_0723900	RNA-binding_protein,_putative	4	2	3	1	0	0	0.669	0.19
PF3D7_0903900	60S_ribosomal_protein_L32	3	2	2	0	1	0	0.659	0.2
PF3D7_1441200	60S_ribosomal_protein_L1,_putative	12	13	12	6	5	0	0.654	0.203
PF3D7_0702500	Plasmodium_exported_protein,_unknown_function	6	4	8	1	1	2	0.652	0.204
PF3D7_1423700	conserved_Plasmodium_protein,_unknown_function	17	12	13	6	8	1	0.666	0.195
PF3D7_0110400	DNA-directed_RNA_polymerase_II_subunit_RPB9,_putative	5	3	5	0	3	0	0.662	0.199
PF3D7_0813100	conserved_Plasmodium_protein,_unknown_function	4	3	1	0	1	0	0.656	0.202
PF3D7_0507100	60S_ribosomal_protein_L4	11	7	9	3	5	0	0.65	0.207
PF3D7_1230400	ATP-dependent_protease_subunit_ClpQ	1	3	3	0	0	0	0.645	0.21
PF3D7_0312800	60S_ribosomal_protein_L26,_putative	6	2	3	1	1	0	0.669	0.192
PF3D7_1116700	dipeptidyl_aminopeptidase_1	12	6	6	3	4	0	0.667	0.193
PF3D7_1026000	conserved_Plasmodium_protein,_unknown_function	3	2	2	0	1	0	0.665	0.196
PF3D7_0826100	HECT-like_E3_ubiquitin_ligase,_putative	87	39	43	28	34	12	0.664	0.197
PF3D7_0931800	proteasome_subunit_beta_type-6,_putative	3	2	2	0	1	0	0.651	0.206
PF3D7_1460700	60S_ribosomal_protein_L27	5	5	4	1	1	1	0.646	0.208
PF3D7_0611800	conserved_Plasmodium_protein,_unknown_function	10	1	3	0	1	0	0.635	0.216
PF3D7_1113400	ubiquitin_domain-containing_protein_DSK2,_putative	80	29	23	20	31	4	0.613	0.235
PF3D7_1130700	structural_maintenance_of_chromosomes_protein_1,_putative	7	2	3	0	2	0	0.645	0.211
PF3D7_0728000	eukaryotic_translation_initiation_factor_2_subunit_alpha	5	3	2	0	2	0	0.643	0.212
PF3D7_1335000	MSP7-like_protein	5	1	1	0	0	0	0.636	0.215

PF3D7_1440100	conserved_Plasmodium_protein,_unknown_function	9	6	4	1	2	2	0.631	0.217
PF3D7_0423800	cysteine-rich_protective_antigen	2	3	3	0	1	0	0.642	0.214
PF3D7_1134100	protein_disulfide_isomerase	7	20	23	4	5	0	0.627	0.219
PF3D7_1124700	GrpE_protein_homolog,_mitochondrial,_putative	13	10	13	5	5	2	0.625	0.221
PF3D7_0213100	protein_SIS1	27	11	10	4	12	1	0.613	0.236
PF3D7_0916700	RNA-binding_protein_musashi,_putative	39	30	26	16	17	8	0.627	0.22
PF3D7_0503200	conserved_Plasmodium_protein,_unknown_function	6	1	2	0	0	1	0.625	0.223
PF3D7_0414000	structural_maintenance_of_chromosomes_protein_3,_putative	8	3	4	1	3	0	0.623	0.225
PF3D7_0815600	eukaryotic_translation_initiation_factor_3_subunit_G,_putative	4	2	5	1	1	0	0.622	0.228
PF3D7_1434800	mitochondrial_acidic_protein_MAM33,_putative	3	4	1	0	1	0	0.618	0.23
PF3D7_0727100	conserved_protein,_unknown_function	4	4	4	0	3	0	0.617	0.231
PF3D7_1111200	conserved_protein,_unknown_function	4	2	1	0	1	0	0.615	0.234
PF3D7_1008900	adenylate_kinase	19	10	8	7	6	0	0.612	0.238
PF3D7_0315400	conserved_Plasmodium_protein,_unknown_function	5	3	4	0	2	1	0.623	0.226
PF3D7_0527500	Hsc70-interacting_protein	55	24	30	21	26	4	0.619	0.229
PF3D7_0520300	U6_snRNA-associated_Sm-like_protein_LSm2,_putative	9	6	5	3	3	0	0.617	0.233
PF3D7_0516200	40S_ribosomal_protein_S11	20	11	13	6	7	7	0.611	0.239
PF3D7_1309100	60S_ribosomal_protein_L24,_putative	7	5	5	2	3	0	0.624	0.224
PF3D7_1130200	60S_ribosomal_protein_P0	29	20	24	12	10	5	0.612	0.237
PF3D7_0323700	U4/U6.U5_tri-snRNP-associated_protein_1,_putative	12	5	4	3	2	1	0.605	0.241
PF3D7_0933900	conserved_Plasmodium_protein,_unknown_function	3	3	4	1	1	0	0.599	0.243
PF3D7_0220000	liver_stage_antigen_3	3	4	2	0	0	0	0.598	0.244
PF3D7_1323400	60S_ribosomal_protein_L23	5	3	4	1	2	0	0.595	0.246
PF3D7_1105000	histone_H4	17	10	8	4	8	3	0.601	0.242
PF3D7_1422700	conserved_Plasmodium_protein,_unknown_function	1	5	5	0	0	0	0.596	0.245
PF3D7_0307100	40S_ribosomal_protein_S12,_putative	15	11	10	4	9	1	0.586	0.25
PF3D7_0526200	ADP-ribosylation_factor_GTPase-activating_protein,_putative	4	1	1	0	0	0	0.574	0.256
PF3D7_0716800	eukaryotic_translation_initiation_factor_3_subunit_I,_putative	4	4	5	1	2	0	0.587	0.248

PF3D7_1359600	conserved_Plasmodium_protein,_unknown_function	67	15	14	11	8	2	0.587	0.249
PF3D7_1234800	splicing_factor_3B_subunit_3,_putative	12	7	5	0	9	0	0.578	0.252
PF3D7_1330300	DnaJ_protein,_putative	7	5	5	1	3	1	0.574	0.258
PF3D7_1316500	pre-mRNA-processing_factor_40,_putative	14	4	1	0	3	0	0.583	0.251
PF3D7_1209800	ATP_synthase_mitochondrial_F1_complex_assembly_factor_1,_putative	1	2	1	0	0	0	0.574	0.258
PF3D7_0317600	40S_ribosomal_protein_S11,_putative	10	4	6	2	5	0	0.566	0.261
PF3D7_1231800	asparagine-rich_protein,_putative	14	7	8	3	6	1	0.576	0.255
PF3D7_1364800	DNA-directed_RNA_polymerases_I,_II,_and_III_subunit_RPABC1,_putative	5	3	3	2	1	0	0.57	0.259
PF3D7_1368900	conserved_protein,_unknown_function	7	3	2	2	0	1	0.567	0.26
PF3D7_0517700	eukaryotic_translation_initiation_factor_3_subunit_B,_putative	3	2	5	1	1	0	0.564	0.264
PF3D7_0313000	conserved_Plasmodium_protein,_unknown_function	13	6	8	3	7	0	0.56	0.266
PF3D7_1438600	conserved_protein,_unknown_function	10	6	7	4	4	0	0.56	0.267
PF3D7_0723800	conserved_Plasmodium_protein,_unknown_function	16	5	11	3	8	0	0.556	0.27
PF3D7_0316700	protein_YOP1,_putative	1	3	3	0	1	0	0.549	0.276
PF3D7_0919000	nucleosome_assembly_protein	11	6	6	2	6	0	0.576	0.253
PF3D7_1006800	single-strand_telomeric_DNA-binding_protein_GBP2,_putative	7	5	4	2	3	0	0.565	0.263
PF3D7_0222100	Pfmc-2TM_Maurer's_cleft_two_transmembrane_protein	1	2	1	0	0	0	0.562	0.265
PF3D7_0710600	60S_ribosomal_protein_L34	3	2	2	0	2	0	0.559	0.268
PF3D7_0912500	SAP_domain-containing_protein,_putative	6	2	3	1	2	0	0.552	0.271
PF3D7_1229300	conserved_Plasmodium_protein,_unknown_function	18	9	17	6	7	2	0.551	0.274
PF3D7_0719700	40S_ribosomal_protein_S10,_putative	11	6	7	2	5	1	0.55	0.275
PF3D7_1345700	isocitrate_dehydrogenase_[NADP],_mitochondrial	1	4	7	1	1	0	0.548	0.278
PF3D7_0719600	60S_ribosomal_protein_L11a,_putative	7	6	5	3	3	0	0.546	0.28
PF3D7_0906600	zinc_finger_protein,_putative	26	10	11	3	12	3	0.544	0.281
PF3D7_1235500	mRNA_methyltransferase,_putative	8	4	4	1	5	0	0.535	0.291

PF3D7_0707700	E3_ubiquitin-protein_ligase,_putative	9	7	3	3	3	0	0.535	0.291
PF3D7_1228600	merozoite_surface_protein_9	25	18	16	8	15	2	0.533	0.292
PF3D7_0815200	importin_subunit_beta,_putative	10	5	8	7	2	0	0.532	0.294
PF3D7_1468800	splicing_factor_U2AF_large_subunit,_putative	9	8	8	4	5	0	0.525	0.299
PF3D7_0614500	60S_ribosomal_protein_L19	2	3	6	0	2	0	0.556	0.27
PF3D7_0821600	polyribonucleotide_5'-hydroxyl-kinase_Clp1,_putative	3	2	1	0	1	0	0.552	0.272
PF3D7_1125500	small_nuclear_ribonucleoprotein_Sm_D1,_putative	2	4	4	1	1	0	0.548	0.277
PF3D7_1366300	conserved_Plasmodium_protein,_unknown_function	17	16	14	7	10	0	0.537	0.287
PF3D7_1453700	HSP90_co-chaperone_p23	29	17	17	14	15	0	0.533	0.293
PF3D7_0420300	AP2_domain_transcription_factor,_putative	23	8	7	3	6	3	0.529	0.297
PF3D7_1326100	WD_repeat-containing_protein,_putative	2	1	2	1	0	0	0.527	0.298
PF3D7_1004000	60S_ribosomal_protein_L13,_putative	9	4	2	1	4	0	0.523	0.301
PF3D7_0721600	40S_ribosomal_protein_S5,_putative	19	17	12	8	11	3	0.519	0.305
PF3D7_1333000	20_kDa_chaperonin	4	3	1	0	2	0	0.547	0.279
PF3D7_0202400	translation-enhancing_factor	4	1	2	0	0	0	0.544	0.282
PF3D7_1027300	peroxiredoxin	13	10	11	6	7	0	0.537	0.285
PF3D7_0813300	conserved_protein,_unknown_function	18	4	5	3	4	0	0.536	0.288
PF3D7_0413700	lysine_decarboxylase-like_protein,_putative	3	5	2	1	1	0	0.531	0.295
PF3D7_1202800	DNA/RNA-binding_protein,_putative	2	2	1	1	0	0	0.519	0.307
PF3D7_0831700	heat_shock_protein_70	16	15	13	6	8	3	0.518	0.308
PF3D7_0805700	serine/threonine_protein_kinase,_FIKK_family	4	2	4	0	3	0	0.516	0.309
PF3D7_1439800	vesicle-associated_membrane_protein,_putative	1	1	2	0	0	0	0.543	0.283
PF3D7_0704400	phosphoinositide-binding_protein,_putative	9	0	3	0	2	0	0.538	0.284
PF3D7_1241900	tetratricopeptide_repeat_protein,_putative	4	0	3	0	1	0	0.537	0.287
PF3D7_1128100	prefoldin_subunit_5,_putative	12	3	2	1	3	0	0.536	0.289
PF3D7_1010700	dolichyl-phosphate-mannose--protein_mannosyltransferase,_putative	32	14	14	8	9	15	0.529	0.297
PF3D7_1115700	cysteine_proteinase_falcipain_2a	6	4	4	1	4	0	0.525	0.3
PF3D7_1420400	glycine--tRNA_ligase	1	5	4	1	1	0	0.52	0.303
PF3D7_0525000	zinc_finger_protein,_putative	2	1	2	0	0	0	0.514	0.31
PF3D7_1427900	leucine-rich_repeat_protein	1	1	2	0	0	0	0.514	0.311
PF3D7_1477700	Plasmodium_exported_protein_(PHISTa),_unknown_function	4	0	2	0	0	0	0.502	0.315

PF3D7_1035500	merozoite_surface_protein_6	9	4	5	3	3	0	0.527	0.298
PF3D7_1115600	peptidyl-prolyl_cis-trans_isomerase	3	4	1	0	2	0	0.521	0.302
PF3D7_1341300	60S_ribosomal_protein_L18-2,_putative	6	3	6	1	4	0	0.52	0.304
PF3D7_0822100	mediator_of_RNA_polymerase_II_transcription_subunit_7,_putative	6	2	0	1	0	0	0.518	0.308
PF3D7_1002400	transformer-2_protein_homolog_beta,_putative	4	2	1	0	2	0	0.513	0.312
PF3D7_0215500	conserved_Plasmodium_protein,_unknown_function	7	1	1	0	1	0	0.513	0.313
PF3D7_1343900	U4/U6_small_nuclear_ribonucleoprotein_PRP4,_putative	0	5	4	0	3	0	0.486	0.323
PF3D7_1341200	60S_ribosomal_protein_L18,_putative	16	10	9	6	5	2	0.519	0.307
PF3D7_1010600	eukaryotic_translation_initiation_factor_2_subunit_beta	10	3	3	2	3	0	0.507	0.314
PF3D7_1340600	RNA_lariat_debranching_enzyme,_putative	0	3	5	0	2	0	0.498	0.316
PF3D7_1330600	elongation_factor_Tu,_putative	0	3	2	0	0	1	0.492	0.32
PF3D7_1414300	60S_ribosomal_protein_L10,_putative	4	5	4	2	1	1	0.489	0.322
PF3D7_1004400	RNA-binding_protein,_putative	1	2	3	0	0	0	0.501	0.315
PF3D7_0507800	conserved_protein,_unknown_function	13	8	6	6	5	0	0.483	0.323
PF3D7_1142500	60S_ribosomal_protein_L28	14	6	6	5	4	1	0.476	0.324
PF3D7_1327800	ribose-phosphate_pyrophosphokinase,_putative	12	8	9	4	6	1	0.498	0.317
PF3D7_1465900	40S_ribosomal_protein_S3	15	6	10	4	5	3	0.495	0.318
PF3D7_0417200	bifunctional_dihydrofolate_reductase-thymidylate_synthase	5	3	3	1	3	0	0.493	0.319
PF3D7_1304100	DNA_ligase_I	5	9	7	3	2	1	0.491	0.321
PF3D7_0406200	sexual_stage-specific_protein_precursor	7	1	4	2	2	0	0.474	0.326
PF3D7_1248700	conserved_Plasmodium_protein,_unknown_function	16	4	4	3	3	1	0.471	0.327
PF3D7_0302100	serine/threonine_protein_kinase	3	3	1	0	2	0	0.466	0.331
PF3D7_1023900	chromodomain-helicase-DNA-binding_protein_1_homolog,_putative	40	16	13	10	13	7	0.464	0.333
PF3D7_1417500	H/ACA_ribonucleoprotein_complex_subunit_4,_putative	3	3	3	2	1	0	0.464	0.333
PF3D7_1224900	splicing_factor_3B_subunit_6,_putative	1	3	1	0	0	1	0.461	0.334
PF3D7_1143000	alpha/beta_hydrolase,_putative	0	2	2	0	0	0	0.469	0.328
PF3D7_0723400	conserved_Plasmodium_protein,_unknown_function	1	3	3	0	0	0	0.469	0.329

PF3D7_0815800	vacuolar_protein_sorting-associated_protein_9,_putative	9	6	4	1	7	0	0.467	0.33
PF3D7_0322900	40S_ribosomal_protein_S3A,_putative	23	11	11	5	10	3	0.46	0.335
PF3D7_1304000	condensin_complex_subunit_2,_putative	4	1	2	1	1	0	0.458	0.337
PF3D7_1426100	transcription_factor_BTF3,_putative	5	4	5	3	2	0	0.453	0.34
PF3D7_1209200	U6_snRNA-associated_Sm-like_protein_LSm7,_putative	12	6	6	3	7	0	0.474	0.326
PF3D7_1140800	conserved_Plasmodium_protein,_unknown_function	19	4	5	4	4	1	0.458	0.336
PF3D7_0508800	single-stranded_DNA-binding_protein	6	3	4	1	4	0	0.454	0.339
PF3D7_0606800	VFT_protein	3	3	5	1	3	0	0.442	0.345
PF3D7_1357800	T-complex_protein_1_subunit_delta	16	15	13	5	8	6	0.463	0.333
PF3D7_1369600	conserved_Plasmodium_protein,_unknown_function	2	6	6	0	5	0	0.457	0.338
PF3D7_1422800	actin-related_protein,_putative	1	1	2	0	0	0	0.451	0.341
PF3D7_0816600	chaperone_protein_ClpB1	13	24	20	10	11	5	0.442	0.345
PF3D7_0614400	pre-mRNA-splicing_factor_CWF7,_putative	2	1	1	0	1	0	0.44	0.346
PF3D7_0512600	ras-related_protein_Rab-1B	4	5	7	3	3	0	0.439	0.347
PF3D7_1302000	EMP1-trafficking_protein	1	3	1	1	0	0	0.438	0.351
PF3D7_0303300	DNA-directed_RNA_polymerases_I,_II,_and_III_subunit_RPABC2,_putative	1	2	1	0	1	0	0.423	0.369
PF3D7_1359400	CUGBP_Elav-like_family_member_1	1	6	5	2	2	0	0.453	0.341
PF3D7_1417200	NOT_family_protein,_putative	48	28	22	21	25	2	0.448	0.342
PF3D7_1026800	40S_ribosomal_protein_S2	16	11	12	6	9	1	0.447	0.343
PF3D7_1212000	glutathione_peroxidase-like_thioredoxin_peroxidase	7	5	5	3	4	0	0.438	0.352
PF3D7_0618200	conserved_protein,_unknown_function	3	0	1	0	0	0	0.437	0.354
PF3D7_1030000	transcription_elongation_factor_SPT4,_putative	1	2	1	0	1	0	0.426	0.363
PF3D7_1104200	chromatin_remodeling_protein	2	4	3	1	1	1	0.418	0.372
PF3D7_1325200	lactate_dehydrogenase,_putative	2	2	0	0	0	0	0.417	0.373
PF3D7_0912800	tRNA_(adenine(58)-N(1))-methyltransferase_non-catalytic_subunit_TRM6,_putative	1	1	2	0	1	0	0.412	0.378
PF3D7_1305100	protein_AMR3	0	2	2	0	1	0	0.439	0.348
PF3D7_0500800	mature_parasite-infected_erythrocyte_surface_antigen	51	33	37	16	38	4	0.438	0.351

PF3D7_1010100	PI31_domain-containing_protein,_putative	7	3	6	2	2	2	0.438	0.351
PF3D7_0914400	conserved_protein,_unknown_function	6	1	1	1	1	0	0.438	0.351
PF3D7_1441600	acid_cluster_protein_33_homologue,_putative	7	1	1	1	1	0	0.438	0.353
PF3D7_0933600	mitochondrial-processing_peptidase_subunit_beta,_putative	1	3	3	1	1	0	0.434	0.356
PF3D7_1317800	40S_ribosomal_protein_S19	3	1	2	1	1	0	0.432	0.357
PF3D7_0822300	small_nuclear_ribonucleoprotein_G,_putative	2	2	2	1	1	0	0.429	0.358
PF3D7_1409800	CUGBP_Elav-like_family_member_2,_putative	4	8	5	2	4	0	0.428	0.359
PF3D7_0814000	60S_ribosomal_protein_L13-2,_putative	9	6	7	5	5	0	0.427	0.361
PF3D7_1424100	60S_ribosomal_protein_L5,_putative	21	14	10	7	11	1	0.426	0.363
PF3D7_0410600	conserved_protein,_unknown_function	3	2	0	1	0	0	0.426	0.366
PF3D7_1323100	60S_ribosomal_protein_L6,_putative	6	4	5	2	3	1	0.425	0.367
PF3D7_1227100	DNA_helicase_60	18	7	6	2	10	1	0.423	0.369
PF3D7_1031500	DEAD/DEAH_box_helicase,_putative	3	0	3	0	0	0	0.419	0.371
PF3D7_1342400	casein_kinase_II_beta_chain	3	2	2	0	3	0	0.415	0.374
PF3D7_1111900	Josephin_domain-containing_protein,_putative	11	4	1	3	3	0	0.408	0.38
PF3D7_1115400	cysteine_proteinase_falcipain_3	2	2	2	1	1	0	0.436	0.354
PF3D7_0903200	ras-related_protein_RAB7	8	6	7	4	5	0	0.436	0.355
PF3D7_1310600	ras-related_protein_Rab-5B	2	1	1	1	0	0	0.428	0.359
PF3D7_0418700	RNA-binding_protein_NOB1,_putative	2	1	1	1	0	0	0.426	0.364
PF3D7_1303200	SNARE_protein,_putative	5	1	1	1	1	0	0.426	0.365
PF3D7_0618000	conserved_Plasmodium_membrane_protein,_unknown_function	4	3	1	1	1	1	0.423	0.367
PF3D7_0629400	polyadenylate-binding_protein_3,_putative	15	4	2	3	4	0	0.42	0.37
PF3D7_1230800	pre-mRNA-splicing_regulator,_putative	8	7	6	2	8	0	0.42	0.371
PF3D7_1036900	conserved_Plasmodium_protein,_unknown_function	39	27	27	12	17	13	0.415	0.374
PF3D7_1225900	conserved_Plasmodium_protein,_unknown_function	3	4	4	2	2	0	0.408	0.382
PF3D7_1466800	conserved_Plasmodium_protein,_unknown_function	9	4	4	1	6	1	0.402	0.389
PF3D7_1417900	ATP_synthase-associated_protein,_putative	2	2	2	0	2	0	0.428	0.36
PF3D7_1110400	RNA-binding_protein,_putative	3	2	1	0	0	0	0.426	0.364
PF3D7_1103700	casein_kinase_II_beta_chain	7	3	2	1	4	0	0.414	0.375

PF3D7_0819900	U6_snRNA-associated_Sm-like_protein_LSm3,_putative	5	1	2	1	2	0	0.409	0.38
PF3D7_1408600	40S_ribosomal_protein_S8e,_putative	22	12	11	7	8	8	0.407	0.383
PF3D7_1334400	MSP7-like_protein	3	1	0	0	0	0	0.406	0.383
PF3D7_1225700	VAC14_domain-containing_protein,_putative	2	1	3	0	0	0	0.404	0.385
PF3D7_1421500	mitochondrial_import_inner_membrane_translocase_subunit_TIM8,_putative	0	1	3	0	0	0	0.403	0.386
PF3D7_1446500	nucleoporin_NUP313,_putative	1	2	6	0	0	0	0.403	0.388
PF3D7_1238100	calcyclin_binding_protein,_putative	9	2	2	1	4	0	0.403	0.388
PF3D7_0205400	PCI_domain-containing_protein,_putative	3	1	2	0	3	0	0.403	0.388
PF3D7_1002900	conserved_Plasmodium_protein,_unknown_function	0	1	2	0	0	0	0.399	0.391
PF3D7_1018500	pre-mRNA-splicing_factor_RDS3,_putative	0	1	2	0	0	0	0.396	0.393
PF3D7_0935900	ring-exported_protein_1	3	1	6	2	1	0	0.391	0.397
PF3D7_1344900	conserved_Plasmodium_protein,_unknown_function	2	1	1	0	1	0	0.413	0.376
PF3D7_0217100	ATP_synthase_subunit_alpha,_mitochondrial	3	4	5	1	1	3	0.413	0.377
PF3D7_0217800	40S_ribosomal_protein_S26	5	2	3	2	1	1	0.408	0.382
PF3D7_1427500	DNA_mismatch_repair_protein_MSH2,_putative	3	3	6	1	1	2	0.404	0.384
PF3D7_1224300	polyadenylate-binding_protein_1,_putative	79	70	57	44	47	21	0.4	0.39
PF3D7_0813400	conserved_protein,_unknown_function	12	8	8	6	6	0	0.393	0.394
PF3D7_0806500	DnaJ_protein,_putative	1	2	1	0	1	0	0.392	0.395
PF3D7_0104200	StAR-related_lipid_transfer_protein	20	7	11	9	10	0	0.385	0.4
PF3D7_0524300	conserved_Plasmodium_protein,_unknown_function	9	2	1	2	2	0	0.376	0.407
PF3D7_1108600	endoplasmic_reticulum-resident_calcium_binding_protein	32	26	28	9	18	16	0.411	0.379
PF3D7_1346200	nuclear_import_protein_MOG1,_putative	2	0	1	0	0	0	0.403	0.386
PF3D7_0907200	GTPase-activating_protein,_putative	2	2	1	0	0	0	0.397	0.392
PF3D7_1308200	carbamoyl_phosphate_synthetase	11	6	11	7	5	0	0.394	0.393
PF3D7_0606700	coatomer_alpha_subunit,_putative	8	3	6	0	8	0	0.391	0.398
PF3D7_0304900	conserved_protein,_unknown_function	2	1	0	0	0	0	0.386	0.4
PF3D7_1461600	splicing_factor_3B_subunit_2,_putative	0	1	3	0	0	0	0.368	0.41
PF3D7_0202000	knob-associated_histidine-rich_protein	8	2	3	3	2	0	0.412	0.377

PF3D7_1470700	conserved_Plasmodium_protein,_unknown_function	2	3	3	1	1	1	0.392	0.395
PF3D7_0713700	conserved_Plasmodium_protein,_unknown_function	0	2	1	0	0	0	0.385	0.401
PF3D7_1134600	zinc_finger_protein,_putative	6	1	1	0	0	0	0.384	0.402
PF3D7_0301700	Plasmodium_exported_protein,_unknown_function	1	2	2	1	1	0	0.381	0.404
PF3D7_1427300	conserved_Plasmodium_protein,_unknown_function	7	1	2	0	4	0	0.378	0.405
PF3D7_0621800	nascent_polypeptide-associated_complex_subunit_alpha,_putative	5	4	3	2	2	1	0.377	0.406
PF3D7_0620500	cleavage_stimulation_factor_subunit_1,_putative	5	0	1	0	0	0	0.372	0.408
PF3D7_0501200	parasite-infected_erythrocyte_surface_protein	14	11	13	10	8	1	0.364	0.414
PF3D7_1226600	proliferating_cell_nuclear_antigen_2	0	2	3	1	1	0	0.364	0.414
PF3D7_0807900	tyrosine--tRNA_ligase	4	6	6	3	4	0	0.36	0.418
PF3D7_1331700	glutamine--tRNA_ligase,_putative	6	8	3	3	3	1	0.358	0.422
PF3D7_1358700	YOP1-like_protein,_putative	0	1	2	0	0	0	0.398	0.391
PF3D7_1468100	kelch_domain-containing_protein,_putative	71	28	31	21	32	11	0.391	0.396
PF3D7_0520000	40S_ribosomal_protein_S9,_putative	8	3	7	2	3	3	0.386	0.399
PF3D7_1208900	protein_phosphatase_PPM11,_putative	14	4	3	1	8	0	0.384	0.402
PF3D7_1468900	zinc_finger_protein,_putative	14	2	3	1	7	0	0.383	0.403
PF3D7_0317500	kinesin-5	8	1	1	0	0	3	0.379	0.404
PF3D7_0721100	conserved_protein,_unknown_function	11	4	6	4	6	0	0.376	0.406
PF3D7_1120000	conserved_protein,_unknown_function	1	3	1	0	0	0	0.368	0.41
PF3D7_0303100	CLP1_P-loop_domain-containing_protein,_putative	10	0	1	0	1	0	0.363	0.415
PF3D7_0913200	elongation_factor_1-beta	1	2	1	0	2	0	0.359	0.419
PF3D7_0308200	T-complex_protein_1_subunit_eta	8	7	7	3	6	1	0.358	0.421
PF3D7_1133400	apical_membrane_antigen_1	4	5	5	3	2	1	0.374	0.408
PF3D7_0621900	signal_recognition_particle_subunit_SRP68,_putative	0	3	8	3	2	0	0.368	0.409
PF3D7_1306900	U1_small_nuclear_ribonucleoprotein_A,_putative	4	3	2	2	2	0	0.365	0.412
PF3D7_1016300	GBP130_protein	9	5	7	3	5	2	0.365	0.413
PF3D7_0209000	6-cysteine_protein_P230	3	2	0	0	1	0	0.359	0.418
PF3D7_0317800	26S_proteasome_non-ATPase_regulatory_subunit_9,_putative	19	10	8	8	9	0	0.358	0.421
PF3D7_1403900	serine/threonine_protein_phosphatase_CPPED1,_putative	4	1	2	2	0	1	0.357	0.423

PF3D7_0311100	pre-mRNA_splicing_factor,_putative	2	0	2	0	0	0	0.357	0.423
PF3D7_1014600	transcriptional_coactivator_ADA2	23	8	8	4	12	3	0.357	0.424
PF3D7_0608500	proteasome_subunit_alpha_type-2,_putative	5	5	7	4	3	1	0.352	0.427
PF3D7_0508000	6-cysteine_protein	3	7	4	1	4	1	0.348	0.429
PF3D7_0418200	eukaryotic_translation_initiation_factor_3_subunit_M,_putative	2	2	3	2	1	0	0.346	0.431
PF3D7_1460500	conserved_Plasmodium_protein,_unknown_function	16	1	1	0	1	0	0.389	0.398
PF3D7_1124400	U6_snRNA-associated_Sm-like_protein_LSm1,_putative	6	1	1	1	2	0	0.367	0.411
PF3D7_0827900	protein_disulfide_isomerase	23	30	16	12	15	3	0.362	0.416
PF3D7_1213900	conserved_Plasmodium_protein,_unknown_function	19	1	2	1	4	0	0.362	0.416
PF3D7_1228800	WD_repeat-containing_protein,_putative	6	2	1	2	2	0	0.361	0.417
PF3D7_0532100	early_transcribed_membrane_protein_5	0	3	3	2	1	0	0.359	0.42
PF3D7_0511800	inositol-3-phosphate_synthase	3	0	1	0	1	0	0.356	0.424
PF3D7_1347500	DNA/RNA-binding_protein_Alba_4	12	8	7	6	7	0	0.355	0.425
PF3D7_0714200	conserved_Plasmodium_protein,_unknown_function	13	5	4	3	6	2	0.353	0.426
PF3D7_0308000	DNA_polymerase_delta_small_subunit,_putative	2	1	1	0	2	0	0.353	0.426
PF3D7_1335100	merozoite_surface_protein_7	33	21	22	19	16	11	0.353	0.427
PF3D7_1119300	splicing_factor_U2AF_small_subunit,_putative	0	2	1	0	1	0	0.347	0.431
PF3D7_1250300	vacuolar_protein_sorting-associated_protein_26,_putative	7	5	3	2	6	0	0.345	0.432
PF3D7_1425900	conserved_Plasmodium_protein,_unknown_function	15	8	8	6	7	3	0.341	0.435
PF3D7_1033200	early_transcribed_membrane_protein_10.2	5	4	5	3	3	0	0.34	0.436
PF3D7_1149200	ring-infected_erythrocyte_surface_antigen	4	2	1	1	2	0	0.338	0.438
PF3D7_0729500	mRNA_(N6-adenosine)-methyltransferase,_putative	3	1	0	0	1	0	0.335	0.439
PF3D7_1119900	protein_transport_protein_SEC16,_putative	32	5	7	8	7	3	0.328	0.446
PF3D7_0917600	pre-mRNA-splicing_factor_ATP-dependent_RNA_helicase_PRP43,_putative	3	1	3	2	1	0	0.363	0.415
PF3D7_0102500	erythrocyte_binding_antigen-181	2	0	3	0	1	0	0.347	0.429
PF3D7_1107000	U6_snRNA-associated_Sm-like_protein_LSm4,_putative	2	1	2	0	1	1	0.344	0.432

PF3D7_1035700	duffy_binding-like_merozoite_surface_protein	15	6	8	6	3	5	0.338	0.437
PF3D7_0915400	ATP-dependent_6-phosphofructokinase	56	25	36	26	30	9	0.334	0.439
PF3D7_1410600	eukaryotic_translation_initiation_factor_2_subunit_gamma,_putative	14	4	4	6	4	0	0.327	0.447
PF3D7_1473700	nucleoporin_NUP116/NSP116,_putative	34	8	9	8	14	1	0.35	0.428
PF3D7_0109200	cleavage_and_polyadenylation_specificity_factor_subunit_5,_putative	10	5	4	4	4	1	0.347	0.43
PF3D7_0730400	IMP1-like_protein,_putative	5	1	3	2	2	0	0.343	0.433
PF3D7_0922200	S-adenosylmethionine_synthetase	47	18	17	19	25	7	0.342	0.434
PF3D7_0310900	conserved_Plasmodium_protein,_unknown_function	5	1	1	1	1	1	0.34	0.435
PF3D7_0517000	60S_ribosomal_protein_L12,_putative	11	10	8	5	6	2	0.333	0.44
PF3D7_1352500	thioredoxin-related_protein,_putative	5	1	1	2	1	0	0.333	0.442
PF3D7_0306100	conserved_Plasmodium_protein,_unknown_function	11	3	2	2	4	1	0.331	0.443
PF3D7_0110500	bromodomain_protein,_putative	16	0	0	0	2	1	0.329	0.445
PF3D7_0317000	proteasome_subunit_alpha_type-3,_putative	3	3	2	1	3	0	0.324	0.45
PF3D7_0422500	pre-mRNA-splicing_helicase_BRR2,_putative	2	3	7	2	2	1	0.323	0.45
PF3D7_1343300	CDT1-like_protein,_putative	4	2	4	2	3	0	0.323	0.451
PF3D7_1353900	proteasome_subunit_alpha_type-7,_putative	12	6	5	6	5	1	0.32	0.452
PF3D7_1328500	alpha/beta-hydrolase,_putative	1	1	2	0	0	0	0.315	0.455
PF3D7_1006200	DNA/RNA-binding_protein_Alba_3	13	7	8	6	4	7	0.313	0.458
PF3D7_0606600	conserved_Plasmodium_protein,_unknown_function	10	7	8	3	11	0	0.312	0.46
PF3D7_0309600	60S_acidic_ribosomal_protein_P2	5	4	7	3	2	2	0.305	0.468
PF3D7_0102900	aspartate--tRNA_ligase	6	0	1	1	1	0	0.342	0.434
PF3D7_0617800	histone_H2A	6	3	2	1	1	3	0.333	0.44
PF3D7_1447800	calponin_homology_domain-containing_protein,_putative	18	0	0	1	1	0	0.333	0.442
PF3D7_1245100	kinesin-13,_putative	25	3	3	6	2	3	0.33	0.443
PF3D7_0523100	mitochondrial-processing_peptidase_subunit_alpha,_putative	0	1	4	2	0	0	0.329	0.444
PF3D7_0415900	60S_ribosomal_protein_L15,_putative	11	5	7	4	7	0	0.327	0.447
PF3D7_0727900	conserved_Plasmodium_protein,_unknown_function	16	0	0	1	1	1	0.327	0.447

PF3D7_0707800	RAP_protein,_putative	0	2	2	0	0	0	0.324	0.449
PF3D7_0924100	conserved_Plasmodium_protein,_unknown_function	4	1	1	0	1	2	0.32	0.452
PF3D7_1438900	thioredoxin_peroxidase_1	24	6	5	9	6	2	0.318	0.453
PF3D7_0407800	conserved_Plasmodium_protein,_unknown_function	18	1	1	3	5	0	0.317	0.453
PF3D7_1012700	NLI_interacting_factor-like_phosphatase,_putative	7	3	4	3	4	0	0.312	0.46
PF3D7_0627800	acetyl-CoA_synthetase,_putative	35	17	22	16	17	9	0.308	0.465
PF3D7_1443900	heat_shock_protein_90,_putative	3	2	3	2	2	0	0.299	0.475
PF3D7_0310600	eukaryotic_translation_initiation_factor_3_subunit_K,_putative,_unspecified_product	0	2	1	0	1	0	0.339	0.437
PF3D7_0821400	conserved_Plasmodium_protein,_unknown_function	3	1	1	1	1	0	0.332	0.442
PF3D7_1360400	conserved_Plasmodium_protein,_unknown_function	3	0	2	1	1	0	0.328	0.445
PF3D7_0724600	protein_kinase,_putative	9	0	0	0	1	0	0.325	0.448
PF3D7_1317200	AP2_domain_transcription_factor_AP2-G3,_putative	5	2	1	0	0	0	0.324	0.449
PF3D7_1116200	pyridoxine_biosynthesis_protein_PDX2	0	4	3	4	1	0	0.317	0.454
PF3D7_1142600	60S_ribosomal_protein_L35ae,_putative	2	1	0	0	1	0	0.316	0.454
PF3D7_0303700	lipoamide_acyltransferase_component_of_branched-chain_alpha-keto_acid_dehydrogenase_complex	2	1	0	0	1	0	0.314	0.457
PF3D7_0801800	mannose-6-phosphate_isomerase,_putative	2	1	1	1	1	0	0.313	0.458
PF3D7_0808400	coatomer_subunit_epsilon,_putative	3	2	4	2	2	0	0.312	0.459
PF3D7_1361000	protein_arginine_N-methyltransferase_5,_putative	7	2	1	2	3	0	0.311	0.461
PF3D7_0731600	acyl-CoA_synthetase	1	2	3	1	2	0	0.311	0.461
PF3D7_1472000	pre-mRNA-splicing_factor_ISY1,_putative	2	1	0	0	1	0	0.31	0.462
PF3D7_1453000	conserved_Plasmodium_protein,_unknown_function	4	0	1	2	0	0	0.309	0.463
PF3D7_0611400	SWIB/MDM2_domain-containing_protein	14	2	2	3	4	1	0.309	0.463
PF3D7_1458500	spindle_assembly_abnormal_protein_4,_putative	12	0	0	0	3	0	0.308	0.464
PF3D7_0308600	pre-mRNA-processing_factor_19,_putative	1	4	3	1	4	0	0.306	0.466
PF3D7_1252300	conserved_Plasmodium_protein,_unknown_function	0	2	2	1	1	0	0.305	0.467
PF3D7_1436300	translocon_component_PTEX150	2	1	1	1	1	0	0.305	0.468

PF3D7_1312900	eukaryotic_translation_initation_factor_4_gamma	7	3	3	3	4	0	0.303	0.469
PF3D7_1443000	serine/threonine_protein_kinase	10	0	0	1	2	0	0.302	0.472
PF3D7_1003800	U5_small_nuclear_ribonucleoprotein_component,_putative	8	2	9	4	6	0	0.302	0.472
PF3D7_1108700	heat_shock_protein_J2	47	28	31	23	27	15	0.3	0.475
PF3D7_0420600	conserved_Plasmodium_protein,_unknown_function	6	0	0	0	1	0	0.299	0.476
PF3D7_0618300	60S_ribosomal_protein_L27a,_putative	8	5	5	2	4	2	0.314	0.456
PF3D7_0705400	DNA_replication_licensing_factor_MCM7	5	4	5	3	4	0	0.314	0.457
PF3D7_1109900	60S_ribosomal_protein_L36	3	2	2	1	2	0	0.31	0.462
PF3D7_1121100	conserved_protein,_unknown_function	13	4	1	0	10	0	0.308	0.465
PF3D7_1435700	ataxin-2_like_protein,_putative	4	0	1	0	0	0	0.307	0.466
PF3D7_0632500	erythrocyte_membrane_protein_1,_PfEMP1	38	0	0	0	0	0	0.306	0.467
PF3D7_0608800	ornithine_aminotransferase	14	7	5	5	5	3	0.303	0.469
PF3D7_1242800	rab_specific_GDP_dissociation_inhibitor	2	1	1	1	1	0	0.303	0.47
PF3D7_1126200	40S_ribosomal_protein_S18,_putative	13	10	9	6	5	4	0.302	0.473
PF3D7_1233600	asparagine_and_aspartate_rich_protein_1	17	3	4	5	7	0	0.301	0.473
PF3D7_1443100	conserved_Plasmodium_protein,_unknown_function	2	1	1	1	1	0	0.301	0.474
PF3D7_1205500	zinc_finger_protein,_putative	9	0	0	0	2	0	0.3	0.475
PF3D7_0410800	conserved_Plasmodium_protein,_unknown_function	13	2	1	4	4	0	0.293	0.478
PF3D7_1037100	pyruvate_kinase_2	13	12	13	6	8	4	0.293	0.478
PF3D7_1410200	CTP_synthase	2	1	1	1	1	0	0.289	0.48
PF3D7_1311800	M1-family_alanyl_aminopeptidase	1	1	3	0	3	0	0.289	0.481
PF3D7_0817500	histidine_triad_nucleotide-binding_protein_1	1	2	0	0	0	1	0.286	0.484
PF3D7_0726300	DNA_mismatch_repair_protein_PMS1,_putative	2	1	0	0	1	0	0.28	0.489
PF3D7_0822900	conserved_Plasmodium_protein,_unknown_function	8	0	0	1	0	0	0.314	0.456
PF3D7_0924700	splicing_factor_3A_subunit_3,_putative	4	5	5	2	6	0	0.302	0.471
PF3D7_0707200	conserved_Plasmodium_protein,_unknown_function	4	2	1	2	2	0	0.298	0.476
PF3D7_1210600	conserved_Plasmodium_protein,_unknown_function	8	3	2	2	4	1	0.298	0.477
PF3D7_0532300	Plasmodium_exported_protein_(PHISTb),_unknown_function	4	5	6	4	4	0	0.296	0.477
PF3D7_1301600	erythrocyte_binding_antigen-140	3	2	1	1	1	1	0.291	0.479
PF3D7_0802000	glutamate_dehydrogenase,_putative	12	6	4	5	7	0	0.289	0.481

PF3D7_1019000	eukaryotic_translation_initiation_factor_subunit_eIF2A,_putative	42	21	26	14	16	19	0.283	0.485
PF3D7_1361100	protein_transport_protein_Sec24A	18	6	6	7	7	2	0.282	0.486
PF3D7_1105100	histone_H2B	13	4	7	5	6	4	0.28	0.489
PF3D7_1454000	RNA-binding_protein,_putative	2	0	1	0	0	0	0.28	0.489
PF3D7_0905900	coatomer_subunit_beta,_putative	7	2	3	1	6	0	0.302	0.471
PF3D7_1224000	GTP_cyclohydrolase_1	3	0	1	0	0	2	0.29	0.479
PF3D7_1452000	rhoptry_neck_protein_2	2	1	1	1	1	0	0.29	0.48
PF3D7_0401800	Plasmodium_exported_protein_(PHISTb),_unknown_function	2	3	2	1	3	0	0.288	0.482
PF3D7_0522100	conserved_Plasmodium_protein,_unknown_function	10	0	0	0	0	0	0.287	0.482
PF3D7_1103600	actin-like_protein,_putative	4	0	1	1	1	0	0.287	0.483
PF3D7_1149000	antigen_332,_DBL-like_protein	4	1	1	0	1	0	0.286	0.483
PF3D7_0731500	erythrocyte_binding_antigen-175	2	0	1	0	1	0	0.286	0.484
PF3D7_0727200	cysteine_desulfurase,_putative	6	5	3	4	2	1	0.283	0.486
PF3D7_0108000	proteasome_subunit_beta_type-3,_putative	2	3	2	1	3	0	0.281	0.487
PF3D7_1003700	MKT1_domain-containing_protein,_putative	6	1	0	0	0	0	0.279	0.49
PF3D7_1461400	MORN_repeat_protein,_putative	2	1	2	0	3	0	0.278	0.49
PF3D7_0213700	conserved_protein,_unknown_function	2	1	1	1	0	1	0.273	0.492
PF3D7_1351700	inner_membrane_complex_protein_1f,_putative	4	0	1	1	1	0	0.272	0.493
PF3D7_1008100	zinc_finger_protein,_putative	66	23	17	12	32	13	0.265	0.496
PF3D7_0513600	deoxyribodipyrimidine_photo-lyase,_putative	0	1	3	0	0	0	0.284	0.485
PF3D7_0616200	kinetochore_protein_NDC80,_putative	2	1	0	0	1	0	0.281	0.487
PF3D7_0617200	conserved_protein,_unknown_function	12	8	7	6	8	0	0.277	0.491
PF3D7_1138500	protein_phosphatase_PPM2	24	7	10	10	13	0	0.274	0.491
PF3D7_1105400	40S_ribosomal_protein_S4,_putative	13	8	10	7	8	0	0.272	0.493
PF3D7_1007700	AP2_domain_transcription_factor_AP2-I	41	14	16	15	20	5	0.27	0.494
PF3D7_0320800	ATP-dependent_RNA_helicase_DDX6	9	4	4	2	3	4	0.268	0.495
PF3D7_0917000	merozoite_organizing_protein	15	1	2	5	4	0	0.265	0.496
PF3D7_1146600	oocyst_rupture_protein_1,_putative	18	8	7	5	10	3	0.264	0.497
PF3D7_1019400	60S_ribosomal_protein_L30e,_putative	5	4	5	3	3	1	0.262	0.498
PF3D7_1439000	copper_transporter	0	3	0	0	0	0	0.255	0.502
PF3D7_1362200	RuvB-like_helicase_3	20	9	8	9	11	2	0.28	0.488

PF3D7_1335700	conserved_oligomeric_Golgi_complex_subunit_3,_putative	2	0	1	1	0	0	0.272	0.493
PF3D7_0113300	Plasmodium_exported_protein_(hyp1),_unknown_function	2	0	1	0	0	0	0.264	0.497
PF3D7_1360800	falcilysin	5	4	7	4	6	0	0.261	0.498
PF3D7_0204700	hexose_transporter	3	2	2	1	2	1	0.259	0.499
PF3D7_1460100	FYVE_and_coiled-coil_domain-containing_protein	4	0	0	0	0	0	0.258	0.5
PF3D7_1140400	conserved_Plasmodium_protein,_unknown_function	3	0	0	0	0	0	0.255	0.501
PF3D7_0306800	T-complex_protein_1_subunit_beta	20	10	10	10	11	3	0.255	0.502
PF3D7_0608700	T-complex_protein_1_subunit_zeta	19	10	9	6	8	10	0.251	0.504
PF3D7_0322000	peptidyl-prolyl_cis-trans_isomerase	5	1	1	2	2	0	0.248	0.507
PF3D7_0606000	conserved_Plasmodium_protein,_unknown_function	5	0	0	1	1	0	0.243	0.511
PF3D7_0424600	Plasmodium_exported_protein_(PHISTb),_unknown_function	8	4	4	2	5	2	0.24	0.512
PF3D7_1447000	40S_ribosomal_protein_S5	21	11	14	6	12	5	0.239	0.514
PF3D7_0404300	conserved_Plasmodium_protein,_unknown_function	4	1	1	2	2	0	0.239	0.514
PF3D7_1345900	kinetochore_protein_SPC25,_putative	5	0	0	0	1	0	0.269	0.494
PF3D7_0708400	heat_shock_protein_90	65	34	33	31	34	12	0.266	0.495
PF3D7_1032700	conserved_Plasmodium_protein,_unknown_function	2	1	0	0	0	0	0.26	0.499
PF3D7_0807800	26S_proteasome_regulatory_subunit_RPN10,_putative	45	22	20	25	23	7	0.258	0.5
PF3D7_0922700	pre-mRNA-splicing_factor_18,_putative	4	2	0	1	2	0	0.258	0.501
PF3D7_0404900	6-cysteine_protein_P41	10	5	6	3	7	1	0.252	0.503
PF3D7_0310400	parasite-infected_erythrocyte_surface_protein	7	7	5	3	9	0	0.252	0.503
PF3D7_0505200	actin-like_protein,_putative	19	2	2	3	12	0	0.25	0.505
PF3D7_1128000	conserved_Plasmodium_protein,_unknown_function	16	1	3	3	8	1	0.249	0.506
PF3D7_1338300	elongation_factor_1-gamma,_putative	5	3	1	2	3	0	0.245	0.508
PF3D7_0107500	Niemann-Pick_type_C1-related_protein	0	2	1	0	2	0	0.243	0.51
PF3D7_1420700	surface_protein_P113	25	19	12	10	17	3	0.239	0.514
PF3D7_1343000	phosphoethanolamine_N-methyltransferase	14	5	4	7	7	0	0.221	0.524
PF3D7_0812400	karyopherin_alpha	26	9	10	14	14	0	0.25	0.504
PF3D7_1428100	WW_domain-binding_protein_11,_putative	2	1	0	0	0	0	0.249	0.506
PF3D7_1037300	ADP/ATP_transporter_on_adenylate_translocase	5	1	5	2	2	2	0.248	0.508

PF3D7_1033100	S-adenosylmethionine_decarboxylase/ornithine_decarboxylase	2	1	1	0	0	0	0.244	0.509
PF3D7_1352700	intron-binding_protein_aquarius,_putative	0	2	1	0	2	0	0.243	0.51
PF3D7_0507600	protein_CAF40,_putative	26	20	15	14	17	3	0.243	0.511
PF3D7_0627700	transportin	8	3	3	3	7	0	0.236	0.515
PF3D7_1474500	splicing_factor_3A_subunit_1,_putative	0	3	0	0	0	0	0.232	0.516
PF3D7_0714000	histone_H2B_variant	6	1	1	2	3	0	0.231	0.518
PF3D7_0506900	rhomboid_protease_ROM4	1	0	2	0	0	0	0.23	0.519
PF3D7_1016200	Rab3_GTPase-activating_protein_non-catalytic_subunit,_putative	2	0	1	0	2	0	0.229	0.52
PF3D7_0615400	ribonuclease,_putative	5	0	1	1	1	1	0.25	0.505
PF3D7_1326400	translation_initiation_factor_eIF-2B_subunit_gamma,_putative	12	0	0	2	3	1	0.249	0.507
PF3D7_0911900	falstatin	16	9	10	4	11	5	0.244	0.509
PF3D7_1140100	V-type_proton_ATPase_subunit_F,_putative	5	1	1	1	2	1	0.242	0.512
PF3D7_1434300	Hsp70/Hsp90_organizing_protein	5	0	3	2	3	0	0.238	0.514
PF3D7_1126000	threonine--tRNA_ligase	1	2	0	1	0	0	0.237	0.515
PF3D7_1426700	phosphoenolpyruvate_carboxylase	4	1	2	0	5	0	0.234	0.516
PF3D7_1032100	mRNA-decapping_enzyme_subunit_1,_putative	16	9	10	9	9	1	0.231	0.517
PF3D7_1466400	AP2_domain_transcription_factor_AP2-EXP	18	3	6	6	3	7	0.231	0.519
PF3D7_1227800	elongator_complex_protein_3,_putative	5	0	0	1	1	0	0.226	0.52
PF3D7_1412100	mini-chromosome_maintenance_complex-binding_protein,_putative	1	1	3	3	1	0	0.224	0.521
PF3D7_1465200	mediator_of_RNA_polymerase_II_transcription_subunit_4,_putative	4	0	3	4	0	1	0.224	0.521
PF3D7_0711500	regulator_of_chromosome_condensation,_putative	2	1	0	0	0	0	0.224	0.522
PF3D7_1004300	E3_ubiquitin-protein_ligase,_putative	25	8	8	11	7	7	0.219	0.525
PF3D7_0932300	M18_aspartyl_aminopeptidase	3	0	0	0	1	0	0.217	0.527
PF3D7_0614300	major_facilitator_superfamily-related_transporter,_putative	0	1	3	1	2	0	0.231	0.519
PF3D7_1343100	conserved_Plasmodium_protein,_unknown_function	3	0	0	0	0	0	0.23	0.519
PF3D7_1460600	inner_membrane_complex_sub-compartment_protein_3	3	0	1	1	1	0	0.222	0.522

PF3D7_0317200	cdc2-related_protein_kinase_4	2	0	1	0	1	1	0.222	0.523
PF3D7_1033700	bromodomain_protein_1	2	2	2	1	2	0	0.221	0.524
PF3D7_0532400	lysine-rich_membrane-associated_PHISTb_protein	4	0	0	0	0	0	0.219	0.525
PF3D7_0923900	polyadenylate-binding_protein_2,_putative	4	6	2	5	3	0	0.214	0.528
PF3D7_0601900	conserved_Plasmodium_protein,_unknown_function	1	2	0	2	0	0	0.213	0.529
PF3D7_1472900	dihydroorotase,_putative	3	3	6	0	9	0	0.206	0.534
PF3D7_0922600	glutamine_synthetase,_putative	6	0	2	1	5	0	0.201	0.537
PF3D7_0320900	histone_H2A.Z	4	2	1	1	1	2	0.221	0.523
PF3D7_1426800	conserved_Plasmodium_protein,_unknown_function	5	0	0	0	2	0	0.218	0.526
PF3D7_1321700	splicing_factor_1	1	0	2	0	2	0	0.215	0.527
PF3D7_1320800	dihydrolipoyllysine-residue_succinyltransferase_component_of_2-oxoglutarate_dehydrogenase_complex	3	2	4	0	6	0	0.214	0.528
PF3D7_0422700	eukaryotic_initiation_factor_4A-III,_putative	6	1	3	2	3	1	0.211	0.53
PF3D7_0309500	asparagine_synthetase_[glutamine-hydrolyzing],_putative	3	1	0	0	3	0	0.21	0.53
PF3D7_0405400	pre-mRNA-processing-splicing_factor_8,_putative	14	8	5	5	13	1	0.21	0.531
PF3D7_1023400	HORMA_domain_protein,_putative	2	0	0	0	0	0	0.206	0.534
PF3D7_0720200	conserved_protein,_unknown_function	2	0	0	0	0	0	0.205	0.535
PF3D7_0316500	kinetochore_protein_NUF2,_putative	5	1	0	1	4	0	0.204	0.536
PF3D7_0524600	50S_ribosomal_protein_L12,_apicoplast,_putative	6	3	3	1	4	2	0.203	0.536
PF3D7_0804900	GTPase-activating_protein,_putative	19	8	9	7	8	9	0.198	0.539
PF3D7_1225200	conserved_Plasmodium_protein,_unknown_function	9	2	2	5	5	0	0.198	0.54
PF3D7_1407800	plasmepsin_IV	33	18	22	15	12	13	0.197	0.542
PF3D7_1230000	TBC_domain-containing_protein,_putative	8	0	0	0	0	0	0.196	0.543
PF3D7_0804800	peptidyl-prolyl_cis-trans_isomerase	2	3	2	2	2	0	0.232	0.517
PF3D7_1212700	eukaryotic_translation_initiation_factor_3_subunit_A,_putative	2	0	1	1	1	0	0.217	0.526
PF3D7_1203200	signal_recognition_particle_subunit_SRP14	0	0	2	0	0	0	0.212	0.529
PF3D7_1206800	conserved_Plasmodium_protein,_unknown_function	19	7	6	10	10	0	0.211	0.53
PF3D7_0525800	inner_membrane_complex_protein_1g,_putative	7	0	0	1	3	0	0.209	0.531
PF3D7_0308300	conserved_Plasmodium_protein,_unknown_function	3	0	0	0	1	0	0.208	0.532

PF3D7_1414800	small_nuclear_ribonucleoprotein-associated_protein_B,_putative	4	4	8	4	5	2	0.207	0.533
PF3D7_0617900	histone_H3_variant	0	0	2	0	0	0	0.207	0.533
PF3D7_1246400	myosin_A_tail_domain_interacting_protein	2	0	0	0	0	0	0.202	0.537
PF3D7_1144900	ras-related_protein_Rab-6	12	8	7	5	9	0	0.199	0.538
PF3D7_1122500	conserved_Plasmodium_protein,_unknown_function	4	0	1	0	0	0	0.198	0.539
PF3D7_0110600	phosphatidylinositol-4-phosphate_5-kinase	3	0	0	1	0	0	0.197	0.541
PF3D7_0218500	small_nuclear_ribonucleoprotein_Sm_D2,_putative	7	4	4	4	4	0	0.197	0.541
PF3D7_1004500	conserved_Plasmodium_protein,_unknown_function	2	0	1	1	1	0	0.19	0.546
PF3D7_1442300	tRNA_import_protein_tRIP	3	2	0	3	1	0	0.187	0.547
PF3D7_1475600	bromodomain_protein,_putative	4	0	0	0	1	1	0.208	0.532
PF3D7_1443400	WD_repeat-containing_protein	2	0	1	0	0	0	0.205	0.535
PF3D7_0616300	conserved_Plasmodium_protein,_unknown_function	2	1	0	1	1	0	0.201	0.538
PF3D7_1472600	protein_disulfide-isomerase	0	0	2	0	0	0	0.197	0.541
PF3D7_1421200	40S_ribosomal_protein_S25	9	5	3	6	4	0	0.196	0.542
PF3D7_0810600	ATP-dependent_RNA_helicase_DBP1,_putative	11	6	6	4	8	1	0.196	0.543
PF3D7_1403100	condensin_complex_subunit_1,_putative	5	0	1	0	0	0	0.193	0.543
PF3D7_0207000	merozoite_surface_protein_4	2	0	0	0	0	0	0.193	0.544
PF3D7_0403600	conserved_Plasmodium_protein,_unknown_function	5	0	2	3	2	0	0.192	0.545
PF3D7_1117700	GTP-binding_nuclear_protein_RAN/TC4	2	1	0	1	1	0	0.191	0.545
PF3D7_1432700	protein-L-isoaspartate(D-aspartate)_O-methyltransferase,_putative	15	5	3	7	6	2	0.186	0.548
PF3D7_0323400	Rh5_interacting_protein	1	2	0	0	0	0	0.184	0.549
PF3D7_0820700	2-oxoglutarate_dehydrogenase_E1_component	2	1	1	0	3	0	0.18	0.552
PF3D7_1450100	signal_recognition_particle_subunit_SRP54	18	9	6	8	11	4	0.179	0.553
PF3D7_1414100	zinc_finger_protein,_putative	3	0	0	0	0	0	0.175	0.556
PF3D7_0726500	ubiquitin_carboxyl-terminal_hydrolase,_putative	8	0	0	0	6	0	0.2	0.538
PF3D7_0516900	60S_ribosomal_protein_L2	11	6	7	4	8	2	0.19	0.546
PF3D7_0320300	T-complex_protein_1_subunit_epsilon	9	5	4	5	6	0	0.186	0.547
PF3D7_1367000	suppressor_of_kinetochore_protein_1,_putative	4	0	0	1	1	0	0.185	0.548

PF3D7_0630600	conserved_protein,_unknown_function	3	1	4	1	6	0	0.184	0.548
PF3D7_1229500	T-complex_protein_1_subunit_gamma	16	7	8	12	5	4	0.183	0.55
PF3D7_0511500	RNA_pseudouridylate_synthase,_putative	5	3	1	1	3	0	0.182	0.551
PF3D7_1473500	conserved_Plasmodium_protein,_unknown_function	6	0	1	3	1	1	0.181	0.551
PF3D7_0214300	conserved_Plasmodium_protein,_unknown_function	7	0	0	0	0	0	0.18	0.552
PF3D7_0519800	EELM2_domain-containing_protein,_putative	3	0	0	0	0	0	0.178	0.553
PF3D7_1302100	gamete_antigen_27/25	18	8	11	8	13	3	0.177	0.554
PF3D7_1473200	DnaJ_protein,_putative	2	1	2	1	2	0	0.177	0.554
PF3D7_0813900	40S_ribosomal_protein_S16,_putative	8	3	3	3	3	3	0.174	0.557
PF3D7_1203700	nucleosome_assembly_protein	14	6	5	8	6	3	0.172	0.557
PF3D7_1035400	merozoite_surface_protein_3	2	0	0	0	1	0	0.167	0.56
PF3D7_0402000	Plasmodium_exported_protein_(PHISTa),_unknown_function	1	1	2	2	1	0	0.167	0.56
PF3D7_0219600	replication_factor_C_subunit_1,_putative	9	2	2	2	3	5	0.164	0.562
PF3D7_1127900	conserved_Plasmodium_protein,_unknown_function	2	0	0	0	0	0	0.156	0.566
PF3D7_1115800	conserved_Plasmodium_protein,_unknown_function	4	2	3	3	2	0	0.193	0.544
PF3D7_1307700	TOM1-like_protein,_putative	3	1	0	2	1	0	0.183	0.55
PF3D7_1011700	DNA_repair_protein_RAD23,_putative	6	3	4	3	5	0	0.177	0.553
PF3D7_1021900	PHAX_domain-containing_protein,_putative	29	14	21	16	14	8	0.176	0.555
PF3D7_1015900	enolase	13	10	8	4	9	6	0.175	0.555
PF3D7_0613500	AP-3_complex_subunit_beta,_putative	4	0	0	1	1	0	0.172	0.557
PF3D7_1317100	DNA_replication_licensing_factor_MCM4	2	5	7	4	9	0	0.17	0.558
PF3D7_0414900	armadillo-domain_containing_rhoptry_protein	3	0	0	0	2	0	0.167	0.56
PF3D7_1003500	40S_ribosomal_protein_S20e,_putative	9	3	4	4	3	4	0.166	0.561
PF3D7_1433300	chromatin_assembly_factor_1_P55_subunit,_putative	2	0	0	0	0	0	0.164	0.562
PF3D7_1406700	vacuolar_protein_sorting-associated_protein_29	5	3	3	4	3	0	0.16	0.563
PF3D7_1418000	ubiquitin_fusion_degradation_protein_1,_putative	7	6	3	4	5	1	0.153	0.568
PF3D7_1468700	eukaryotic_initiation_factor_4A	54	21	21	22	27	22	0.15	0.571
PF3D7_1134800	coatomer_subunit_delta	6	5	3	4	4	0	0.169	0.558
PF3D7_1477300	Plasmodium_exported_protein_(PHIST),_unknown_function	6	1	1	1	4	1	0.168	0.559

PF3D7_1302800	40S_ribosomal_protein_S7,_putative	14	7	7	8	5	3	0.164	0.562
PF3D7_1236300	conserved_protein,_unknown_function	2	0	0	1	0	0	0.161	0.562
PF3D7_1408200	AP2_domain_transcription_factor_AP2-G2,_putative	5	0	0	0	0	0	0.161	0.563
PF3D7_0106800	ras-related_protein_Rab-5C	3	0	1	1	2	0	0.157	0.564
PF3D7_1205600	tetratricopeptide_repeat_protein,_putative	14	6	8	8	11	0	0.156	0.565
PF3D7_0811500	histone-arginine_methyltransferase_CARM1,_putative	3	0	0	0	2	0	0.155	0.566
PF3D7_0704600	E3_ubiquitin-protein_ligase	9	0	0	3	5	0	0.154	0.567
PF3D7_0927200	zinc_finger_protein,_putative	5	0	0	0	0	0	0.154	0.567
PF3D7_1238700	BTB/POZ_domain-containing_protein,_putative	2	0	0	0	1	0	0.153	0.569
PF3D7_0321500	peptidase,_putative	2	0	0	1	0	0	0.145	0.574
PF3D7_0730800	Plasmodium_exported_protein,_unknown_function,_unspecified_product	2	0	0	0	0	0	0.184	0.549
PF3D7_1247500	serine/threonine_protein_kinase,_putative	4	0	0	0	0	0	0.175	0.556
PF3D7_0515000	pre-mRNA-splicing_factor_CWC2,_putative	24	11	10	13	10	8	0.157	0.565
PF3D7_1206200	eukaryotic_translation_initiation_factor_3_subunit_C,_putative	2	2	2	1	2	0	0.155	0.566
PF3D7_0629700	SET_domain_protein,_putative	4	0	0	0	0	1	0.153	0.568
PF3D7_1237700	conserved_protein,_unknown_function	6	3	3	2	4	2	0.152	0.57
PF3D7_1020900	ADP-ribosylation_factor	16	6	6	9	9	3	0.151	0.57
PF3D7_1034000	Sec1_family_protein,_putative	2	1	1	0	4	1	0.149	0.571
PF3D7_1439500	oocyst_rupture_protein_2,_putative	3	0	1	1	3	0	0.146	0.572
PF3D7_1229400	macrophage_migration_inhibitory_factor	3	0	0	1	1	0	0.145	0.574
PF3D7_1461800	conserved_Plasmodium_protein,_unknown_function	6	0	0	0	0	0	0.143	0.575
PF3D7_1401100	DnaJ_protein,_putative	2	0	0	0	0	0	0.14	0.576
PF3D7_1462100	conserved_Plasmodium_protein,_unknown_function	2	0	0	1	0	0	0.136	0.578
PF3D7_1213800	proline--tRNA_ligase	17	8	12	10	9	4	0.159	0.564
PF3D7_0807300	ras-related_protein_Rab-18	2	2	1	2	1	0	0.153	0.569
PF3D7_0920700	CRAL/TRIO_domain-containing_protein,_putative	4	0	1	2	3	0	0.15	0.571
PF3D7_0603100	RNA-binding_protein,_putative	2	0	0	0	0	0	0.149	0.572
PF3D7_1365900	ubiquitin-60S_ribosomal_protein_L40	7	4	4	3	4	2	0.145	0.574
PF3D7_0803200	filament_assembling_protein,_putative	2	0	0	0	1	0	0.144	0.574
PF3D7_1438400	metacaspase-2	5	0	0	0	0	0	0.142	0.575

PF3D7_1213200	mediator_of_RNA_polymerase_II_transcription_subunit_18,_putative	3	1	1	1	4	0	0.137	0.577
PF3D7_1106000	RuvB-like_helicase_2	18	7	6	12	9	3	0.137	0.577
PF3D7_0409600	replication_protein_A1,_large_subunit	3	0	0	0	0	0	0.136	0.578
PF3D7_1028700	merozoite_TRAP-like_protein	2	0	0	0	0	0	0.134	0.579
PF3D7_1144000	40S_ribosomal_protein_S21	5	2	4	4	3	0	0.131	0.58
PF3D7_1353100	Plasmodium_exported_protein,_unknown_function	6	6	6	4	4	1	0.13	0.58
PF3D7_0501300	skeleton-binding_protein_1	0	2	1	2	1	0	0.127	0.58
PF3D7_1416100	protein_SEY1,_putative	0	0	2	0	0	0	0.145	0.574
PF3D7_0934500	V-type_proton_ATPase_subunit_E,_putative	4	1	2	2	5	0	0.14	0.576
PF3D7_1330800	RNA-binding_protein,_putative	7	4	4	4	6	0	0.138	0.577
PF3D7_1329600	conserved_Plasmodium_protein,_unknown_function	4	0	0	1	2	0	0.132	0.579
PF3D7_1230700	protein_transport_protein_SEC13	28	9	11	15	15	8	0.126	0.581
PF3D7_0604100	AP2_domain_transcription_factor	2	0	0	0	1	0	0.125	0.582
PF3D7_0211800	asparagine--tRNA_ligase	0	1	2	1	4	0	0.125	0.583
PF3D7_0716200	PDCD2_domain-containing_protein,_putative	4	1	1	2	5	0	0.123	0.583
PF3D7_1238800	acyl-CoA_synthetase	4	0	0	1	3	0	0.12	0.585
PF3D7_0420000	zinc_finger_protein,_putative	4	0	2	0	0	0	0.125	0.581
PF3D7_0822600	protein_transport_protein_SEC23	19	7	9	10	15	0	0.125	0.583
PF3D7_0113200	Plasmodium_exported_protein,_unknown_function	2	0	0	0	0	0	0.12	0.584
PF3D7_0909800	small_nuclear_ribonucleoprotein_Sm_D3,_putative	2	0	2	4	1	0	0.12	0.585
PF3D7_1213700	DNA-directed_RNA_polymerases_I,_II,_and_III_subunit_RPABC3,_putative	2	1	1	1	2	0	0.116	0.586
PF3D7_0323600	conserved_Plasmodium_protein,_unknown_function	2	0	0	0	0	0	0.113	0.588
PF3D7_0803700	tubulin_gamma_chain	1	2	0	0	0	3	0.109	0.589
PF3D7_0309300	N2227-like_protein,_putative	6	0	1	5	3	0	0.108	0.59
PF3D7_0415000	arsenical_pump-driving_ATPase,_putative	0	3	2	3	2	0	0.106	0.591
PF3D7_1222700	glideosome-associated_protein_45	3	0	2	2	2	0	0.121	0.584
PF3D7_1342600	myosin_A	2	0	1	0	3	0	0.12	0.584
PF3D7_1331500	conserved_Plasmodium_protein,_unknown_function	2	0	0	0	0	0	0.119	0.586
PF3D7_0311300	phosphatidylinositol_3-_and_4-kinase,_putative	2	1	2	1	1	1	0.115	0.587
PF3D7_0823300	histone_acetyltransferase_GCN5	20	8	6	6	11	9	0.113	0.587

PF3D7_0623600	transcription_or_splicing_factor-like_protein,_putative	2	0	2	1	2	0	0.113	0.588
PF3D7_1117900	conserved_Plasmodium_protein,_unknown_function	3	0	0	0	0	0	0.11	0.588
PF3D7_1145100	coatomer_subunit_gamma,_putative	20	7	8	11	11	5	0.102	0.594
PF3D7_0621200	pyridoxine_biosynthesis_protein_PDX1	3	2	1	2	6	0	0.102	0.594
PF3D7_0613800	AP2_domain_transcription_factor,_putative	4	0	0	2	1	0	0.095	0.599
PF3D7_0605100	RNA-binding_protein,_putative	8	9	7	6	8	0	0.109	0.589
PF3D7_0209100	patatin-like_phospholipase,_putative	3	2	3	3	7	0	0.107	0.59
PF3D7_1003600	inner_membrane_complex_protein_1c,_putative	2	0	0	1	1	0	0.104	0.592
PF3D7_1420000	splicing_factor_3B_subunit_4,_putative	1	3	3	2	4	0	0.103	0.592
PF3D7_1407300	pre-mRNA-splicing_factor_38B,_putative	2	0	0	0	0	0	0.103	0.593
PF3D7_0925900	parasitophorous_vacuolar_protein_5,_putative	36	11	15	13	20	10	0.101	0.595
PF3D7_0214000	T-complex_protein_1_subunit_theta	5	4	6	4	6	0	0.1	0.596
PF3D7_1136400	signal_recognition_particle_subunit_SRP72,_putative	3	2	2	3	2	1	0.098	0.596
PF3D7_1209300	telomere_repeat-binding_zinc_finger_protein	3	0	0	0	0	0	0.097	0.597
PF3D7_0517300	serine/arginine-rich_splicing_factor_1	4	2	3	2	4	0	0.094	0.599
PF3D7_1407100	rRNA_2'-O-methyltransferase_fibrillarin,_putative	2	2	2	2	3	0	0.093	0.6
PF3D7_1464500	conserved_Plasmodium_membrane_protein,_unknown_function	4	1	0	0	0	0	0.09	0.602
PF3D7_1123500	golgi_protein_2	4	4	3	2	3	4	0.09	0.602
PF3D7_1354300	large_subunit_rRNA_methyltransferase,_putative	2	0	0	0	0	0	0.084	0.606
PF3D7_1110500	vacuolar_protein_sorting-associated_protein_35,_putative	3	4	5	5	5	0	0.105	0.591
PF3D7_1320600	ras-related_protein_Rab-11A	3	3	4	3	3	0	0.104	0.592
PF3D7_1305900	conserved_Plasmodium_protein,_unknown_function	2	0	0	0	1	1	0.103	0.593
PF3D7_1239600	hydroxyethylthiazole_kinase	2	3	1	2	3	0	0.101	0.595
PF3D7_1133800	RNA_(uracil-5-)methyltransferase,_putative	1	2	1	0	2	2	0.098	0.596
PF3D7_1014900	conserved_Plasmodium_protein,_unknown_function	2	0	0	0	2	0	0.097	0.597
PF3D7_0917900	heat_shock_protein_70	133	84	94	55	61	52	0.095	0.598
PF3D7_1337500	conserved_Plasmodium_protein,_unknown_function	4	1	0	0	0	0	0.095	0.599

PF3D7_1461300	40S_ribosomal_protein_S28e,_putative	2	1	2	1	1	1	0.093	0.599
PF3D7_0219700	Plasmodium_exported_protein_(PHISTc),_unknown_function	2	1	0	2	0	1	0.092	0.6
PF3D7_0405100	protein_transport_protein_Sec24B,_putative	5	0	0	0	6	0	0.091	0.601
PF3D7_1221000	histone-lysine_N-methyltransferase,_H3_lysine-4_specific	3	0	0	1	3	0	0.091	0.601
PF3D7_1474800	proteasome_subunit_alpha_type-1,_putative	3	3	2	3	3	0	0.088	0.603
PF3D7_1368100	26S_proteasome_regulatory_subunit_RPN11,_putative	13	3	3	7	7	4	0.085	0.604
PF3D7_1445900	ATP-dependent_RNA_helicase_DDX5,_putative	4	3	4	2	5	1	0.085	0.605
PF3D7_0602000	zinc_finger_protein,_putative	3	0	0	0	0	0	0.085	0.605
PF3D7_1444800	fructose-bisphosphate_aldolase	2	2	0	2	5	0	0.084	0.606
PF3D7_0818900	heat_shock_protein_70	275	143	##	109	119	75	0.084	0.606
PF3D7_1417800	DNA_replication_licensing_factor_MCM2	11	4	5	6	10	0	0.084	0.607
PF3D7_0518500	ATP-dependent_RNA_helicase_DDX23,_putative	2	1	0	1	3	0	0.082	0.608
PF3D7_1359000	conserved_Plasmodium_protein,_unknown_function	3	0	0	2	1	0	0.08	0.609
PF3D7_0215700	DNA-directed_RNA_polymerase_II_subunit_RPB2,_putative	2	2	2	0	2	2	0.089	0.603
PF3D7_1220900	heterochromatin_protein_1	2	1	0	1	1	1	0.089	0.603
PF3D7_1135400	conserved_Plasmodium_protein,_unknown_function	8	6	7	4	5	3	0.085	0.604
PF3D7_0607600	spindle_assembly_abnormal_protein_6,_putative	2	0	0	0	0	0	0.083	0.607
PF3D7_1442400	conserved_Plasmodium_protein,_unknown_function	2	1	0	0	0	0	0.082	0.608
PF3D7_1118500	nucleolar_protein_56,_putative	7	8	7	7	7	1	0.079	0.61
PF3D7_0719000	conserved_protein,_unknown_function	9	2	5	5	5	3	0.078	0.61
PF3D7_1134200	conserved_Plasmodium_protein,_unknown_function	2	0	1	0	0	0	0.074	0.611
PF3D7_0207400	serine_repeat_antigen_7	2	1	2	2	3	0	0.063	0.618
PF3D7_0729100	conserved_Plasmodium_protein,_unknown_function	3	0	1	0	0	0	0.081	0.609
PF3D7_0929200	RNA-binding_protein,_putative	38	18	13	19	23	13	0.08	0.609
PF3D7_1329100	myosin_F,_putative	20	5	3	9	9	9	0.075	0.611
PF3D7_0716000	RNA-binding_protein,_putative	3	2	2	1	8	0	0.073	0.612
PF3D7_1002700	conserved_Plasmodium_protein,_unknown_function	2	1	1	2	2	1	0.071	0.612
PF3D7_0909600	conserved_protein,_unknown_function	4	5	6	6	4	2	0.069	0.613
PF3D7_1038000	antigen_UB05	2	1	1	1	1	2	0.068	0.613

PF3D7_1470900	proteasome_subunit_beta_type-2,_putative	3	1	1	2	2	1	0.068	0.614
PF3D7_1211900	non-SERCA-type_Ca2+_-transporting_P-ATPase	11	11	12	7	15	1	0.066	0.616
PF3D7_0520900	adenosylhomocysteinase	4	8	5	6	8	0	0.064	0.617
PF3D7_0610400	histone_H3	1	1	2	1	1	3	0.063	0.618
PF3D7_0818200	14-3-3_protein	64	28	30	25	32	18	0.062	0.619
PF3D7_1454400	aminopeptidase_P	11	10	7	8	13	0	0.061	0.621
PF3D7_1364100	6-cysteine_protein	13	16	19	9	19	6	0.061	0.621
PF3D7_1235300	CCR4-NOT_transcription_complex_subunit_4,_putative	2	1	2	1	2	1	0.059	0.623
PF3D7_1008800	nucleolar_protein_5,_putative	1	4	4	4	4	2	0.056	0.624
PF3D7_0907700	proteasome_activator_28_subunit_beta,_putative	1	2	0	3	2	0	0.056	0.625
PF3D7_0811400	conserved_protein,_unknown_function	4	5	2	4	7	0	0.055	0.625
PF3D7_0624000	hexokinase	2	2	4	3	5	0	0.07	0.612
PF3D7_1218500	dynamin-like_protein,_putative	2	6	7	5	9	0	0.068	0.614
PF3D7_0905400	high_molecular_weight_rhoptry_protein_3	11	8	5	9	6	2	0.067	0.615
PF3D7_0711000	AAA_family_ATPase,_CDC48_subfamily	2	1	2	2	2	0	0.067	0.615
PF3D7_1349600	conserved_Plasmodium_protein,_unknown_function	7	0	2	4	6	0	0.066	0.616
PF3D7_1021600	deoxyribose-phosphate_aldolase,_putative	0	5	2	4	4	0	0.064	0.617
PF3D7_1211400	heat_shock_protein_DNAJ_homologue_Pfj4	16	6	5	8	5	10	0.063	0.619
PF3D7_1416200	metacaspase-3,_putative	2	1	0	0	0	0	0.063	0.619
PF3D7_1355100	DNA_replication_licensing_factor_MCM6	2	2	3	4	4	0	0.062	0.62
PF3D7_0922500	phosphoglycerate_kinase	6	2	5	4	5	2	0.062	0.62
PF3D7_1232100	60_kDa_chaperonin	11	14	13	8	16	2	0.061	0.621
PF3D7_1317300	conserved_Plasmodium_protein,_unknown_function	3	1	1	2	3	1	0.061	0.622
PF3D7_1347200	nucleoside_transporter_1	2	1	3	2	3	2	0.06	0.622
PF3D7_1247400	peptidyl-prolyl_cis-trans_isomerase_FKBP35	6	2	4	4	5	0	0.057	0.624
PF3D7_1414400	serine/threonine_protein_phosphatase_PP1	2	2	1	2	3	2	0.057	0.624
PF3D7_0207700	serine_repeat_antigen_4	4	12	6	7	13	0	0.055	0.626
PF3D7_1453800	glucose-6-phosphate_dehydrogenase-6-phosphogluconolactonase	2	2	2	3	6	0	0.055	0.626
PF3D7_1464700	ATP_synthase_(C/AC39)_subunit,_putative	1	1	2	2	2	2	0.054	0.627
PF3D7_0501100	heat_shock_protein_40,_type_II	2	5	1	1	2	7	0.054	0.627
PF3D7_0612100	eukaryotic_translation_initiation_factor_3_subunit_L,_putative	2	0	2	3	1	2	0.053	0.629

PF3D7_1343700	kelch_protein_K13	4	1	2	1	4	4	0.051	0.631
PF3D7_1358500	zinc_finger_protein,_putative	3	1	0	3	1	2	0.05	0.632
PF3D7_1330400	ER_lumen_protein_retaining_receptor_1,_putative,_unspecified_product	2	0	0	1	1	1	0.05	0.633
PF3D7_0507700	nuclear_protein_localization_protein_4,_putative	14	11	10	11	10	2	0.065	0.616
PF3D7_0501500	rhoptry-associated_protein_3	5	8	6	4	8	3	0.057	0.624
PF3D7_0612700	6-cysteine_protein_P12	6	8	8	7	10	0	0.054	0.627
PF3D7_0607000	translation_initiation_factor_IF-2,_putative	4	2	1	4	4	0	0.054	0.628
PF3D7_0909900	helicase_SKI2W,_putative	2	0	0	0	0	0	0.053	0.628
PF3D7_0807500	proteasome_subunit_alpha_type-6,_putative	3	1	1	2	3	1	0.053	0.629
PF3D7_0907400	ATP-dependent_protease_ATPase_subunit_ClpY	11	10	11	8	3	13	0.052	0.629
PF3D7_1231100	ras-related_protein_Rab-2	2	2	2	3	3	1	0.052	0.63
PF3D7_1237200	conserved_Plasmodium_protein,_unknown_function	3	2	1	2	3	0	0.052	0.63
PF3D7_1420600	pantothenate_kinase_1,_putative	8	2	5	3	5	4	0.051	0.631
PF3D7_0616800	malate:quinone_oxidoreductase	4	5	6	4	4	5	0.05	0.632
PF3D7_0527000	DNA_replication_licensing_factor_MCM3,_putative	3	7	5	7	9	1	0.05	0.632
PF3D7_1233200	conserved_Plasmodium_protein,_unknown_function	4	0	0	0	0	0	0.05	0.633
PF3D7_1324900	L-lactate_dehydrogenase	8	4	4	6	6	0	0.049	0.634
PF3D7_0727400	proteasome_subunit_alpha_type-5,_putative	5	3	1	3	5	1	0.048	0.634
PF3D7_0207600	serine_repeat_antigen_5	25	24	15	15	25	3	0.046	0.636
PF3D7_0914700	major_facilitator_superfamily-related_transporter,_putative	6	7	6	6	7	4	0.043	0.637
PF3D7_1451700	calcineurin_subunit_B	2	0	0	2	1	0	0.043	0.637
PF3D7_1369400	conserved_Plasmodium_protein,_unknown_function	3	0	0	0	0	0	0.039	0.643
PF3D7_0918000	glideosome-associated_protein_50	4	6	4	7	6	2	0.038	0.644
PF3D7_1116800	heat_shock_protein_101	6	6	11	5	5	10	0.048	0.634
PF3D7_0801000	Plasmodium_exported_protein_(PHISTc),_unknown_function	1	1	2	2	2	2	0.048	0.635
PF3D7_0929400	high_molecular_weight_rhoptry_protein_2	14	8	13	9	17	1	0.047	0.635
PF3D7_0906500	arginase	2	0	0	1	2	0	0.046	0.636
PF3D7_1123900	13_kDa_ribonucleoprotein-associated_protein,_putative	10	9	10	7	6	4	0.043	0.637
PF3D7_0404600	conserved_Plasmodium_membrane_protein,_unknown_function	4	0	0	0	0	0	0.043	0.638
PF3D7_0212300	peptide_chain_release_factor_subunit_1,_putative	4	6	4	4	8	1	0.043	0.638

PF3D7_1456800	V-type_H(+)-translocating_pyrophosphatase,_putative	14	16	9	8	12	9	0.042	0.639
PF3D7_1108400	casein_kinase_2,_alpha_subunit	7	6	6	6	5	6	0.042	0.639
PF3D7_0706000	importin-7,_putative	3	1	1	3	5	0	0.042	0.64
PF3D7_1105800	conserved_Apicomplexan_protein,_unknown_function	3	1	1	3	4	0	0.042	0.64
PF3D7_0501600	rhoptry-associated_protein_2	10	15	12	7	9	8	0.041	0.641
PF3D7_1466900	conserved_Plasmodium_protein,_unknown_function	0	1	2	4	2	0	0.041	0.641
PF3D7_0528100	AP-1_complex_subunit_beta,_putative	2	0	2	3	8	0	0.041	0.641
PF3D7_0618700	trafficking_protein_particle_complex_subunit_6A,_putative	5	2	2	4	4	2	0.041	0.641
PF3D7_0308900	splicing_factor_3B_subunit_1,_putative	0	0	3	2	4	0	0.04	0.642
PF3D7_0703500	erythrocyte_membrane-associated_antigen	16	11	10	11	16	2	0.039	0.642
PF3D7_1107300	polyadenylate-binding_protein-interacting_protein_1,_putative	23	23	22	19	32	3	0.038	0.643
PF3D7_1344800	aspartate_carbamoyltransferase	0	3	4	3	5	2	0.038	0.644
PF3D7_0315100	eukaryotic_translation_initiation_factor_4E	5	2	4	5	5	1	0.038	0.644
PF3D7_1132800	aquaglyceroporin	4	4	3	3	3	5	0.037	0.645
PF3D7_1414000	26S_proteasome_regulatory_subunit_RPN13,_putative	8	4	5	5	7	1	0.037	0.645
PF3D7_1107400	DNA_repair_protein_RAD51	5	3	2	4	8	0	0.035	0.647
PF3D7_1223100	cAMP-dependent_protein_kinase_regulatory_subunit	8	5	4	7	8	1	0.034	0.647
PF3D7_1118200	heat_shock_protein_90,_putative	46	43	42	28	31	14	0.032	0.649
PF3D7_1120100	phosphoglycerate_mutase,_putative	6	4	6	6	5	3	0.032	0.649
PF3D7_0802800	serine/threonine_protein_phosphatase_2B_catalytic_subunit_A	0	1	4	6	3	3	0.029	0.656
PF3D7_0108300	conserved_Plasmodium_protein,_unknown_function	42	26	24	24	22	17	0.039	0.643
PF3D7_1463200	replication_factor_C_subunit_3,_putative	5	5	4	1	1	9	0.036	0.645
PF3D7_0730500	conserved_Plasmodium_protein,_unknown_function	3	0	0	0	0	0	0.036	0.646
PF3D7_1437200	ribonucleoside-diphosphate_reductase_large_subunit,_putative	8	3	3	6	8	0	0.036	0.647
PF3D7_1332000	syntaxin,_Qa-SNARE_family	2	0	0	2	2	0	0.036	0.647
PF3D7_1216900	DNA-binding_chaperone,_putative	5	2	1	5	6	1	0.034	0.648

PF3D7_0810800	hydroxymethyldihydropterin_pyrophosphokinase-dihydropteroate_synthase	2	0	0	1	2	0	0.033	0.648
PF3D7_0525100	acyl-CoA_synthetase	20	8	6	11	12	7	0.032	0.649
PF3D7_0406100	V-type_proton_ATPase_subunit_B	6	2	2	4	5	4	0.031	0.65
PF3D7_1117100	ubiquitin_carboxyl-terminal_hydrolase_UCH54	15	10	11	11	14	2	0.031	0.651
PF3D7_0918300	eukaryotic_translation_initiation_factor_3_subunit_F,_putative	3	1	1	3	2	3	0.031	0.651
PF3D7_1342900	AP2_domain_transcription_factor,_putative	2	0	1	0	0	0	0.031	0.651
PF3D7_0303000	N-ethylmaleimide-sensitive_fusion_protein	3	2	5	4	4	8	0.03	0.652
PF3D7_1136500	casein_kinase_1	2	4	5	6	7	1	0.03	0.652
PF3D7_0105700	asparagine-rich_antigen_Pfa35-2	2	1	0	0	0	0	0.03	0.653
PF3D7_1012400	hypoxanthine-guanine_phosphoribosyltransferase	1	2	2	3	6	0	0.03	0.654
PF3D7_0305200	conserved_Plasmodium_protein,_unknown_function	0	2	0	3	3	0	0.03	0.654
PF3D7_0102200	ring-infected_erythrocyte_surface_antigen	6	2	4	7	10	2	0.029	0.654
PF3D7_1360900	RNA-binding_protein,_putative	11	15	17	10	16	2	0.029	0.654
PF3D7_0715200	conserved_Plasmodium_protein,_unknown_function	2	0	0	0	0	0	0.029	0.655
PF3D7_0820000	Snf2-related_CBP_activator,_putative	2	0	0	0	0	0	0.029	0.656
PF3D7_1132200	T-complex_protein_1_subunit_alpha	9	6	7	5	9	7	0.028	0.656
PF3D7_1104000	phenylalanine--tRNA_ligase_beta_subunit	1	2	4	4	7	1	0.028	0.656
PF3D7_1103800	CCR4-NOT_transcription_complex_subunit_1,_putative	12	12	9	14	18	0	0.028	0.657
PF3D7_1126900	small_nuclear_ribonucleoprotein_F,_putative	2	4	2	2	3	2	0.028	0.657
PF3D7_0814200	DNA/RNA-binding_protein_Alba_1	12	8	10	4	7	7	0.027	0.657
PF3D7_0706500	conserved_Plasmodium_protein,_unknown_function	31	22	24	18	27	19	0.027	0.659
PF3D7_0302900	exportin-1,_putative	3	1	1	6	5	1	0.027	0.659
PF3D7_0303200	HAD_superfamily_protein,_putative	3	1	0	5	4	0	0.027	0.659
PF3D7_0217500	calcium-dependent_protein_kinase_1	9	1	3	7	9	1	0.025	0.659
PF3D7_1008700	tubulin_beta_chain	45	33	21	34	16	23	0.024	0.66

PF3D7_1442900	protein_transport_protein_SEC7,_putative	5	2	3	3	6	0	0.024	0.66
PF3D7_0702400	small_exported_membrane_protein_1	4	3	4	4	3	2	0.023	0.661
PF3D7_0316800	40S_ribosomal_protein_S15A,_putative	4	5	9	3	5	8	0.023	0.661
PF3D7_0910100	exportin-7,_putative	18	5	13	15	16	4	0.022	0.663
PF3D7_0708800	heat_shock_protein_110	26	31	29	23	33	1	0.031	0.65
PF3D7_0709700	prodrug_activation_and_resistance_esterase	7	2	3	4	5	7	0.03	0.652
PF3D7_1457300	MA3_domain-containing_protein,_putative	4	0	1	4	4	0	0.027	0.659
PF3D7_0306900	40S_ribosomal_protein_S23,_putative	8	5	5	5	5	3	0.024	0.66
PF3D7_0310500	ATP-dependent_RNA_helicase_DHX57,_putative	3	2	1	3	5	0	0.023	0.661
PF3D7_0625400	conserved_Plasmodium_protein,_unknown_function	17	14	14	7	7	19	0.022	0.663
PF3D7_1336800	nuclear_movement_protein,_putative	10	2	3	7	4	8	0.022	0.663
PF3D7_1030500	26S_proteasome_regulatory_subunit_RPN9,_putative	7	3	2	6	4	8	0.022	0.663
PF3D7_0503400	actin-depolymerizing_factor_1	6	8	3	7	8	0	0.022	0.663
PF3D7_0826500	ubiquitin_conjugation_factor_E4_B,_putative	10	6	7	8	15	0	0.022	0.664
PF3D7_0826700	receptor_for_activated_c_kinase	20	12	14	10	8	13	0.021	0.664
PF3D7_0214100	protein_transport_protein_SEC31	28	15	10	17	19	9	0.021	0.664
PF3D7_0623500	superoxide_dismutase_[Fe]	3	2	3	7	9	0	0.02	0.665
PF3D7_1222300	endoplasmin,_putative	52	39	33	26	38	16	0.018	0.666
PF3D7_0626800	pyruvate_kinase	13	23	22	21	16	9	0.018	0.666
PF3D7_1132000	ubiquitin-like_protein,_putative	7	5	6	8	3	19	0.017	0.666
PF3D7_1410400	rhoptry-associated_protein_1	26	27	26	19	24	12	0.016	0.667
PF3D7_1311500	26S_protease_regulatory_subunit_7,_putative	13	5	6	9	8	7	0.016	0.667
PF3D7_0704100	conserved_Plasmodium_membrane_protein,_unknown_function	2	0	0	0	0	0	0.015	0.668
PF3D7_1456700	conserved_Plasmodium_protein,_unknown_function	3	3	1	5	11	1	0.015	0.669
PF3D7_0619400	cell_division_cycle_protein_48_homologue,_putative	60	31	39	35	44	15	0.014	0.67
PF3D7_0209800	ATP-dependent_RNA_helicase_UAP56	9	4	3	8	7	3	0.019	0.665
PF3D7_1446200	M17_leucyl_aminopeptidase	9	1	2	8	8	4	0.017	0.667
PF3D7_1429800	coatomer_subunit_beta,_putative	10	3	7	13	14	0	0.015	0.668
PF3D7_1145400	dynamin-like_protein	19	5	5	14	16	4	0.015	0.668
PF3D7_1451100	elongation_factor_2	59	33	33	37	45	11	0.014	0.669

PF3D7_1451200	conserved_Plasmodium_protein,_unknown_function	2	0	0	0	1	2	0.014	0.67
PF3D7_0903700	alpha_tubulin_1	25	20	14	19	15	11	0.013	0.67
PF3D7_1025300	conserved_protein,_unknown_function	16	17	23	9	12	40	0.013	0.671
PF3D7_0206800	merozoite_surface_protein_2	6	6	8	8	9	3	0.013	0.671
PF3D7_1471100	exported_protein_2	11	9	9	8	11	4	0.012	0.672
PF3D7_1252100	rhoptry_neck_protein_3	3	0	3	1	6	2	0.012	0.672
PF3D7_0524000	karyopherin_beta	45	19	21	30	36	5	0.012	0.672
PF3D7_1329500	conserved_protein,_unknown_function	13	3	4	13	15	11	0.012	0.672
PF3D7_1325100	phosphoribosylpyrophosphate_synthetase	31	23	20	19	21	26	0.011	0.673
PF3D7_1119400	ubiquitin-protein_ligase,_putative	4	0	0	5	1	11	0.01	0.673
PF3D7_1407900	plasmepsin_I	26	12	15	16	13	17	0.009	0.674
PF3D7_1130400	26S_protease_regulatory_subunit_6A,_putative	26	12	14	15	12	15	0.009	0.674
PF3D7_1311900	V-type_proton_ATPase_catalytic_subunit_A	14	6	4	10	10	10	0.009	0.675
PF3D7_1306400	26S_protease_regulatory_subunit_10B,_putative	15	3	4	10	6	11	0.009	0.675
PF3D7_0819600	conserved_protein,_unknown_function	18	9	12	16	19	0	0.008	0.676
PF3D7_0416800	small_GTP-binding_protein_sar1	22	10	8	12	9	13	0.008	0.676
PF3D7_0823200	RNA-binding_protein,_putative	12	7	5	13	7	4	0.008	0.676
PF3D7_1216300	signal_recognition_particle_subunit_SRP19	6	6	4	6	6	7	0.008	0.676
PF3D7_0627500	protein_DJ-1	4	7	5	9	10	6	0.007	0.676
PF3D7_1111100	replication_factor_C_subunit_5,_putative	4	1	1	8	3	11	0.007	0.677
PF3D7_0914900	BSD-domain_protein,_putative	21	5	5	14	10	17	0.007	0.677
PF3D7_1017900	26S_proteasome_regulatory_subunit_p55,_putative	16	5	4	13	9	8	0.006	0.677
PF3D7_1248900	26S_protease_regulatory_subunit_8,_putative	22	9	11	16	11	17	0.006	0.678
PF3D7_1008400	26S_protease_regulatory_subunit_4,_putative	19	12	12	13	12	16	0.005	0.678
PF3D7_1129200	26S_proteasome_regulatory_subunit_RPN7,_putative	11	4	5	12	7	11	0.005	0.679
PF3D7_0312300	26S_proteasome_regulatory_subunit_RPN12,_putative	4	5	1	10	5	5	0.005	0.679
PF3D7_1408100	plasmepsin_III	33	14	15	18	17	15	0.004	0.679
PF3D7_0629200	DnaJ_protein,_putative	15	5	8	7	0	25	0.002	0.682
PF3D7_1305300	translational_activator_GCN1,_putative	2	0	0	2	3	0	0.004	0.679
PF3D7_1412500	actin_II	22	7	10	13	7	20	0.003	0.68
PF3D7_1408000	plasmepsin_II	34	18	17	26	20	24	0.003	0.681
PF3D7_0912900	26S_proteasome_regulatory_subunit_RPN8,_putative	11	2	6	15	7	13	0.003	0.681

PF3D7_1402300	26S_proteasome_regulatory_subunit_RPN6	17	7	7	18	7	19	0.003	0.681
PF3D7_0205900	26S_proteasome_regulatory_subunit_RPN1,_putative	29	14	16	34	31	33	0.002	0.682
PF3D7_0218000	replication_factor_C_subunit_2,_putative	4	0	3	7	3	15	0.002	0.682
PF3D7_1241700	replication_factor_C_subunit_4,_putative	5	2	2	2	3	14	0.001	0.682
PF3D7_1338100	26S_proteasome_regulatory_subunit_RPN3,_putative	12	7	8	17	13	15	0.001	0.683
PF3D7_1462800	glyceraldehyde-3-phosphate_dehydrogenase	31	22	20	24	21	40	7E-04	0.683
PF3D7_0413600	26S_protease_regulatory_subunit_6B,_putative	18	5	4	20	11	21	3E-04	0.683
PF3D7_0930300	merozoite_surface_protein_1	281	185	##	168	168	125	0	0.885
PF3D7_1357000	elongation_factor_1-alpha	127	68	83	70	95	55	0	0.885
PF3D7_1246200	actin_I	112	50	46	100	45	101	0	0.885
PF3D7_1437900	HSP40,_subfamily_A	63	30	28	48	26	63	0	0.885
PF3D7_1129100	parasitophorous_vacuolar_protein_1	41	26	28	41	10	78	0	0.885
PF3D7_1466300	26S_proteasome_regulatory_subunit_RPN2,_putative	27	15	15	37	29	27	0	0.885
PF3D7_1110200	pre-mRNA-processing_factor_6,_putative	1	1	1	0	0	1	0	0.885
PF3D7_0415400	coatomer_subunit_zeta,_putative	1	1	1	1	2	0	0	0.885
PF3D7_0824400	nucleoside_transporter_2	1	1	1	1	2	0	0	0.885
PF3D7_1217900	PPPDE_peptidase_domain-containing_protein,_putative	1	1	1	1	2	0	0	0.885
PF3D7_1033600	pre-mRNA-splicing_factor_CEF1,_putative	1	1	1	0	2	0	0	0.885
PF3D7_1129800	conserved_Plasmodium_protein,_unknown_function	1	1	1	2	1	0	0	0.885
PF3D7_0319100	E3_ubiquitin-protein_ligase_RBX1,_putative	1	1	1	1	1	0	0	0.885
PF3D7_0822800	U5_small_nuclear_ribonucleoprotein_40_kDa_protein,_putative	1	1	1	1	1	0	0	0.885
PF3D7_0936800	Plasmodium_exported_protein_(PHISTc),_unknown_function	1	1	1	1	1	0	0	0.885
PF3D7_0925700	histone_deacetylase_1	1	1	1	0	1	0	0	0.885
PF3D7_1355700	NLI_interacting_factor-like_phosphatase,_putative	1	1	1	0	1	0	0	0.885
PF3D7_0703300	conserved_Plasmodium_protein,_unknown_function	1	1	1	1	0	0	0	0.885
PF3D7_0911100	conserved_protein,_unknown_function	1	1	1	1	0	0	0	0.885
PF3D7_0923000	DNA-directed_RNA_polymerase_II_subunit_RPB3,_putative	1	1	1	1	0	0	0	0.885
PF3D7_0108500	conserved_Plasmodium_protein,_unknown_function	1	1	1	0	0	0	0	0.885

PF3D7_0708500	heat_shock_protein_86_family_protein	1	1	1	0	0	0	0	0.885
PF3D7_0731100	EMP1-trafficking_protein	1	1	1	0	0	0	0	0.885
PF3D7_0831300	Plasmodium_exported_protein,_unknown_function	1	1	1	0	0	0	0	0.885
PF3D7_0831400	Plasmodium_exported_protein,_unknown_function	1	1	1	0	0	0	0	0.885
PF3D7_1016900	early_transcribed_membrane_protein_10.3	1	1	1	0	0	0	0	0.885
PF3D7_1025100	glutamine--fructose-6-phosphate_aminotransferase_[isomerizing],_putative	1	1	1	0	0	0	0	0.885
PF3D7_1138000	conserved_Plasmodium_protein,_unknown_function	1	1	1	0	0	0	0	0.885
PF3D7_1305000	MCL1_domain-containing_protein,_putative	1	1	1	0	0	0	0	0.885
PF3D7_1309300	U4/U6_small_nuclear_ribonucleoprotein_PRP3,_putative	1	1	1	0	0	0	0	0.885
PF3D7_1324400	PRELI_domain-containing_protein,_putative	1	1	1	0	0	0	0	0.885
PF3D7_1353400	Ran-binding_protein,_putative	1	1	1	0	0	0	0	0.885
PF3D7_0316600	formate-nitrite_transporter	0	1	1	1	4	3	0	0.885
PF3D7_0936600	gametocyte_exported_protein_5	0	1	1	0	0	3	0	0.885
PF3D7_0309800	YTH_domain-containing_protein,_putative	0	1	1	1	0	1	0	0.885
PF3D7_0527600	conserved_Plasmodium_protein,_unknown_function	0	1	1	1	0	1	0	0.885
PF3D7_1034900	methionine--tRNA_ligase	0	1	1	0	2	0	0	0.885
PF3D7_1118300	insulinase,_putative	0	1	1	0	2	0	0	0.885
PF3D7_0606100	RNA-binding_protein,_putative	0	1	1	0	1	0	0	0.885
PF3D7_0620400	merozoite_surface_protein_10	0	1	1	0	1	0	0	0.885
PF3D7_1231600	pre-mRNA-splicing_factor_ATP-dependent_RNA_helicase_PRP2,_putative	0	1	1	0	1	0	0	0.885
PF3D7_1251200	coronin	0	1	1	0	1	0	0	0.885
PF3D7_1323200	V-type_proton_ATPase_subunit_G,_putative	0	1	1	0	1	0	0	0.885
PF3D7_0105800	cyclin-dependent_kinases_regulatory_subunit,_putative	0	1	1	0	0	0	0	0.885
PF3D7_0111800	eukaryotic_translation_initiation_factor_4E,_putative	0	1	1	0	0	0	0	0.885
PF3D7_0318200	DNA-directed_RNA_polymerase_II_subunit_RPB1	0	1	1	0	0	0	0	0.885
PF3D7_0505500	DNA_mismatch_repair_protein_MSH6,_putative	0	1	1	0	0	0	0	0.885
PF3D7_0521700	ATP-dependent_RNA_helicase_DDX1,_putative	0	1	1	0	0	0	0	0.885

PF3D7_0616000	pyridoxal_kinase	0	1	1	0	0	0	0	0.885
PF3D7_0913700	conserved_Plasmodium_protein,_unknown_function	0	1	1	0	0	0	0	0.885
PF3D7_0918200	50S_ribosomal_protein_L3,_apicoplast,_putative	0	1	1	0	0	0	0	0.885
PF3D7_1248500	bax_inhibitor_1,_putative	0	1	1	0	0	0	0	0.885
PF3D7_1434100	queuine_tRNA-ribosyltransferase,_putative	0	1	1	0	0	0	0	0.885
PF3D7_1447200	conserved_protein,_unknown_function	0	1	1	0	0	0	0	0.885
PF3D7_1415300	RNA-binding_protein_Nova-1,_putative	1	0	1	1	0	6	0	0.885
PF3D7_1312700	conserved_Plasmodium_protein,_unknown_function	1	0	1	1	0	1	0	0.885
PF3D7_1335400	reticulocyte_binding_protein_2_homologue_a	1	0	1	0	3	0	0	0.885
PF3D7_0903500	nucleoporin_NUP138,_putative	1	0	1	0	2	0	0	0.885
PF3D7_1035900	probable_protein,_unknown_function	1	0	1	0	2	0	0	0.885
PF3D7_0212400	conserved_Plasmodium_membrane_protein,_unknown_function	1	0	1	1	1	0	0	0.885
PF3D7_1206500	Tat_binding_protein_1(TBP-1)-interacting_protein,_putative	1	0	1	1	1	0	0	0.885
PF3D7_0619500	acyl-CoA_synthetase	1	0	1	0	1	0	0	0.885
PF3D7_1121600	exported_protein_1	1	0	1	0	1	0	0	0.885
PF3D7_1127800	TFIIS_central_domain-containing_protein,_putative	1	0	1	0	1	0	0	0.885
PF3D7_1139100	RNA-binding_protein,_putative	1	0	1	0	1	0	0	0.885
PF3D7_1251500	ATP-dependent_RNA_helicase_DRS1,_putative	1	0	1	0	1	0	0	0.885
PF3D7_0201900	erythrocyte_membrane_protein_3	1	0	1	0	0	0	0	0.885
PF3D7_1143400	translation_initiation_factor_eIF-1A,_putative	1	0	1	0	0	0	0	0.885
PF3D7_1249500	protein_CutA,_putative	1	0	1	0	0	0	0	0.885
PF3D7_1324700	SNARE_protein,_putative	1	0	1	0	0	0	0	0.885
PF3D7_1446700	conserved_Plasmodium_protein,_unknown_function	1	0	1	0	0	0	0	0.885
PF3D7_1451900	ribosome_biogenesis_protein_TSR1,_putative	1	0	1	0	0	0	0	0.885
PF3D7_0718500	prefoldin_subunit_3,_putative	0	0	1	1	1	1	0	0.885
PF3D7_0317300	conserved_Plasmodium_protein,_unknown_function	0	0	1	2	7	0	0	0.885
PF3D7_1455500	AP-1_complex_subunit_gamma,_putative	0	0	1	4	5	0	0	0.885
PF3D7_1466100	protein_phosphatase_containing_kelch-like_domains	0	0	1	2	4	0	0	0.885
PF3D7_1361900	proliferating_cell_nuclear_antigen_1	0	0	1	2	2	0	0	0.885

PF3D7_0406500	NYN_domain-containing_protein,_putative	0	0	1	1	2	0	0	0.885
PF3D7_0505000	MMS19-like_protein,_putative	0	0	1	1	2	0	0	0.885
PF3D7_0610900	transcription_elongation_factor_SPT5,_putative	0	0	1	1	2	0	0	0.885
PF3D7_1253400	acyl-CoA_synthetase	0	0	1	0	2	0	0	0.885
PF3D7_1029600	adenosine_deaminase	0	0	1	3	1	0	0	0.885
PF3D7_1224500	histone_chaperone_ASF1,_putative	0	0	1	2	1	0	0	0.885
PF3D7_0302500	cytoadherence_linked_asexual_protein_3.1	0	0	1	1	1	0	0	0.885
PF3D7_0509000	alpha-soluble_NSF_attachment_protein,_putative	0	0	1	1	1	0	0	0.885
PF3D7_0904600	ubiquitin_specific_protease,_putative	0	0	1	1	1	0	0	0.885
PF3D7_0314000	HSP20-like_chaperone,_putative	0	0	1	0	1	0	0	0.885
PF3D7_0423100	AP-4_complex_subunit_sigma,_putative	0	0	1	0	1	0	0	0.885
PF3D7_1022700	phospholipid_scramblase,_putative	0	0	1	0	1	0	0	0.885
PF3D7_0106700	small_ribosomal_subunit_assembling_AARP2_protein	0	0	1	1	0	0	0	0.885
PF3D7_1136600	conserved_Plasmodium_protein,_unknown_function	0	0	1	1	0	0	0	0.885
PF3D7_1357700	U3_small_nucleolar_RNA-associated_protein_21,_putative	0	0	1	1	0	0	0	0.885
PF3D7_0312400	glycogen_synthase_kinase_3	0	0	1	0	0	0	0	0.885
PF3D7_0511300	MORN_repeat_protein,_putative	0	0	1	0	0	0	0	0.885
PF3D7_0602100	ATP-dependent_RNA_helicase,_putative	0	0	1	0	0	0	0	0.885
PF3D7_0703900	conserved_Plasmodium_membrane_protein,_unknown_function	0	0	1	0	0	0	0	0.885
PF3D7_0706400	60S_ribosomal_protein_L37	0	0	1	0	0	0	0	0.885
PF3D7_0714500	transcription_elongation_factor_s-II,_putative	0	0	1	0	0	0	0	0.885
PF3D7_0912400	alkaline_phosphatase,_putative	0	0	1	0	0	0	0	0.885
PF3D7_0930400	zinc_finger_protein,_putative	0	0	1	0	0	0	0	0.885
PF3D7_0931400	PUB_domain-containing_protein,_putative	0	0	1	0	0	0	0	0.885
PF3D7_1017000	DNA_polymerase_delta_catalytic_subunit	0	0	1	0	0	0	0	0.885
PF3D7_1104700	DNA-directed_RNA_polymerase_III_subunit_RPC8,_putative	0	0	1	0	0	0	0	0.885

PF3D7_1112500	RNA_transcription,_translation_and_transport_factor_protein,_putative	0	0	1	0	0	0	0	0.885
PF3D7_1332800	eukaryotic_translation_initiation_factor_6,_putative	0	0	1	0	0	0	0	0.885
PF3D7_1409200	conserved_Plasmodium_protein,_unknown_function	0	0	1	0	0	0	0	0.885
PF3D7_1412400	conserved_Plasmodium_protein,_unknown_function	0	0	1	0	0	0	0	0.885
PF3D7_1458700	exonuclease_V,_mitochondrial,_putative	0	0	1	0	0	0	0	0.885
PF3D7_1466700	60S_ribosome_subunit_biogenesis_protein_NIP7,_putative	0	0	1	0	0	0	0	0.885
PF3D7_1478000	Plasmodium_exported_protein_(PHISTa),_unknown_function	0	0	1	0	0	0	0	0.885
PF3D7_0727800	cation_transporting_ATPase,_putative	1	1	0	0	1	2	0	0.885
PF3D7_0933300	conserved_Plasmodium_protein,_unknown_function	1	1	0	0	0	1	0	0.885
PF3D7_0911200	conserved_Plasmodium_protein,_unknown_function	1	1	0	1	3	0	0	0.885
PF3D7_1249300	protein_phosphatase_PPM4,_putative	1	1	0	0	2	0	0	0.885
PF3D7_1464600	serine/threonine_protein_phosphatase_UIS2,_putative	1	1	0	0	2	0	0	0.885
PF3D7_0807000	YEATS_domain-containing_protein,_putative	1	1	0	1	1	0	0	0.885
PF3D7_0904100	AP-4_complex_subunit_epsilon,_putative	1	1	0	1	1	0	0	0.885
PF3D7_1244200	RNA_polymerase_II_transcription_factor_B_subunit_2,_putative	1	1	0	1	1	0	0	0.885
PF3D7_0807600	conserved_Plasmodium_protein,_unknown_function	1	1	0	0	1	0	0	0.885
PF3D7_0827500	ribosomal_protein_L21,_apicoplast,_putative	1	1	0	0	1	0	0	0.885
PF3D7_1125800	kelch_domain-containing_protein,_putative	1	1	0	0	1	0	0	0.885
PF3D7_0628200	eukaryotic_translation_initiation_factor_2-alpha_kinase	1	1	0	2	0	0	0	0.885
PF3D7_0319600	elongation_factor_1-delta,_putative	1	1	0	1	0	0	0	0.885
PF3D7_0526500	conserved_Plasmodium_protein,_unknown_function	1	1	0	1	0	0	0	0.885
PF3D7_0921000	ubiquitin-conjugating_enzyme_E2,_putative	1	1	0	1	0	0	0	0.885
PF3D7_1127000	protein_phosphatase,_putative	1	1	0	1	0	0	0	0.885
PF3D7_0828800	GPI-anchored_micronemal_antigen	1	1	0	0	0	0	0	0.885
PF3D7_1318300	conserved_Plasmodium_protein,_unknown_function	1	1	0	0	0	0	0	0.885
PF3D7_0829200	prohibitin_1,_putative	0	1	0	0	0	1	0	0.885

PF3D7_0104100	conserved_Plasmodium_membrane_protein,_unknown_function	0	1	0	0	5	0	0	0.885
PF3D7_1353800	proteasome_subunit_alpha_type-4,_putative	0	1	0	3	4	0	0	0.885
PF3D7_0109800	phenylalanine--tRNA_ligase_alpha_subunit	0	1	0	1	4	0	0	0.885
PF3D7_0516700	ubiquitin_carboxyl-terminal_hydrolase_2,_putative	0	1	0	0	4	0	0	0.885
PF3D7_1459000	ATP-dependent_RNA_helicase_DBP5	0	1	0	1	3	0	0	0.885
PF3D7_0802200	1-cys_peroxiredoxin	0	1	0	0	3	0	0	0.885
PF3D7_0210100	60S_ribosomal_protein_L37ae,_putative	0	1	0	0	2	0	0	0.885
PF3D7_0310000	50S_ribosomal_protein_L9,_apicoplast,_putative	0	1	0	0	2	0	0	0.885
PF3D7_0820500	protein_transport_protein_YIF1,_putative	0	1	0	0	2	0	0	0.885
PF3D7_1438700	DNA_primase_small_subunit	0	1	0	0	2	0	0	0.885
PF3D7_0502000	vacuolar_protein_sorting-associated_protein_11,_putative	0	1	0	1	1	0	0	0.885
PF3D7_0518200	SWIB/MDM2_domain-containing_protein	0	1	0	1	1	0	0	0.885
PF3D7_0110700	chromatin_assembly_factor_1_protein_WD40_domain,_putative	0	1	0	0	1	0	0	0.885
PF3D7_0308500	activator_of_Hsp90_ATPase,_putative	0	1	0	0	1	0	0	0.885
PF3D7_1143300	DNA-directed_RNA_polymerases_I_and_III_subunit_RPAC1,_putative	0	1	0	0	1	0	0	0.885
PF3D7_1433400	zinc_finger_protein,_putative	0	1	0	0	1	0	0	0.885
PF3D7_0512000	prefoldin_subunit_6,_putative	0	1	0	1	0	0	0	0.885
PF3D7_0520200	mediator_of_RNA_polymerase_II_transcription_subunit_17,_putative	0	1	0	1	0	0	0	0.885
PF3D7_1332300	trafficking_protein_particle_complex_subunit_2,_putative	0	1	0	1	0	0	0	0.885
PF3D7_1355800	splicing_factor_3B_subunit_5,_putative	0	1	0	1	0	0	0	0.885
PF3D7_0109500	N-acetyltransferase,_GNAT_family,_putative	0	1	0	0	0	0	0	0.885
PF3D7_0313800	conserved_Plasmodium_protein,_unknown_function	0	1	0	0	0	0	0	0.885
PF3D7_0403700	pre-mRNA-splicing_factor_CLF1,_putative	0	1	0	0	0	0	0	0.885
PF3D7_0419800	60S_ribosomal_protein_L7ae/L30e,_putative	0	1	0	0	0	0	0	0.885

PF3D7_0504800	conserved_Plasmodium_protein,_unknown_function	0	1	0	0	0	0	0	0.885
PF3D7_0728900	RNA-binding_protein,_putative	0	1	0	0	0	0	0	0.885
PF3D7_1001600	exported_lipase_2	0	1	0	0	0	0	0	0.885
PF3D7_1027400	DNA-directed_RNA_polymerase_II_subunit_RPB7,_putative	0	1	0	0	0	0	0	0.885
PF3D7_1116100	serine_esterase,_putative	0	1	0	0	0	0	0	0.885
PF3D7_1204300	eukaryotic_translation_initiation_factor_5A	0	1	0	0	0	0	0	0.885
PF3D7_1320900	RNA-binding_protein,_putative	0	1	0	0	0	0	0	0.885
PF3D7_1325000	U6_snRNA-associated_Sm-like_protein_LSm6,_putative	0	1	0	0	0	0	0	0.885
PF3D7_1344300	zinc_finger_protein,_putative	0	1	0	0	0	0	0	0.885
PF3D7_1350100	lysine--tRNA_ligase	0	1	0	0	0	0	0	0.885
PF3D7_1419300	glutathione_S-transferase	0	1	0	0	0	0	0	0.885
PF3D7_1450500	conserved_Plasmodium_protein,_unknown_function	0	1	0	0	0	0	0	0.885
PF3D7_1451500	pre-mRNA-splicing_factor_CWF18,_putative	0	1	0	0	0	0	0	0.885
PF3D7_1469800	conserved_Plasmodium_protein,_unknown_function	0	1	0	0	0	0	0	0.885
PF3D7_API01800	apicoplast_ribosomal_protein_L16	0	1	0	0	0	0	0	0.885
PF3D7_1315300	conserved_protein,_unknown_function	1	0	0	1	0	7	0	0.885
PF3D7_1458400	aminodeoxychorismate_lyase	1	0	0	2	0	2	0	0.885
PF3D7_0935800	cytoadherence_linked_asexual_protein_9	1	0	0	1	4	1	0	0.885
PF3D7_0609700	conserved_Plasmodium_protein,_unknown_function	1	0	0	0	2	1	0	0.885
PF3D7_0829000	conserved_Plasmodium_membrane_protein,_unknown_function	1	0	0	0	2	1	0	0.885
PF3D7_1021800	schizont_egress_antigen-1	1	0	0	0	2	1	0	0.885
PF3D7_1206400	rhodanese_like_protein,_putative	1	0	0	0	0	1	0	0.885
PF3D7_1358100	Sas10_domain-containing_protein,_putative	1	0	0	0	0	1	0	0.885
PF3D7_1025000	EF-hand_calcium-binding_domain-containing_protein,_putative	1	0	0	0	6	0	0	0.885
PF3D7_1347700	ethanolamine-phosphate_cytidylyltransferase	1	0	0	1	4	0	0	0.885
PF3D7_1346400	VPS13_domain-containing_protein,_putative	1	0	0	2	3	0	0	0.885
PF3D7_0307500	spindle_and_kinetochore-associated_protein_2,_putative	1	0	0	1	3	0	0	0.885

PF3D7_0808100	AP-3_complex_subunit_delta,_putative	1	0	0	1	2	0	0	0.885
PF3D7_0302800	RNA-binding_protein,_putative	1	0	0	0	2	0	0	0.885
PF3D7_0705500	inositol-phosphate_phosphatase,_putative	1	0	0	0	2	0	0	0.885
PF3D7_1409300	DNA_damage-inducible_protein_1,_putative	1	0	0	0	2	0	0	0.885
PF3D7_1426200	protein_arginine_N-methyltransferase_1	1	0	0	0	2	0	0	0.885
PF3D7_0730200	AP-4_complex_subunit_beta,_putative	1	0	0	2	1	0	0	0.885
PF3D7_1218200	conserved_Plasmodium_protein,_unknown_function	1	0	0	2	1	0	0	0.885
PF3D7_0304200	EH_domain-containing_protein	1	0	0	1	1	0	0	0.885
PF3D7_0629800	cullin-like_protein,_putative	1	0	0	1	1	0	0	0.885
PF3D7_0717500	calcium-dependent_protein_kinase_4	1	0	0	1	1	0	0	0.885
PF3D7_0824600	Fe-S_cluster_assembly_protein_DRE2,_putative	1	0	0	1	1	0	0	0.885
PF3D7_0903400	ATP-dependent_RNA_helicase_DDX60,_putative	1	0	0	1	1	0	0	0.885
PF3D7_1124300	conserved_protein,_unknown_function	1	0	0	1	1	0	0	0.885
PF3D7_1304900	DNA-directed_RNA_polymerase_II_subunit_RPB11,_putative	1	0	0	1	1	0	0	0.885
PF3D7_1432100	voltage-dependent_anion-selective_channel_protein,_putative	1	0	0	1	1	0	0	0.885
PF3D7_0625600	poly(A)_polymerase_PAP,_putative	1	0	0	0	1	0	0	0.885
PF3D7_0627900	ribonuclease_P_protein_subunit_p29,_putative	1	0	0	0	1	0	0	0.885
PF3D7_0920800	inosine-5'-monophosphate_dehydrogenase	1	0	0	0	1	0	0	0.885
PF3D7_1102500	Plasmodium_exported_protein_(PHISTb),_unknown_function	1	0	0	0	1	0	0	0.885
PF3D7_1207000	conserved_Plasmodium_protein,_unknown_function	1	0	0	0	1	0	0	0.885
PF3D7_1316900	conserved_protein,_unknown_function	1	0	0	0	1	0	0	0.885
PF3D7_1331200	conserved_Plasmodium_protein,_unknown_function	1	0	0	0	1	0	0	0.885
PF3D7_1367100	U1_small_nuclear_ribonucleoprotein_70_kDa_homolog,_putative	1	0	0	0	1	0	0	0.885
PF3D7_1410700	conserved_protein,_unknown_function	1	0	0	0	1	0	0	0.885

PF3D7_1438500	cleavage_and_polyadenylation_specificity_factor_subunit_3,_putative	1	0	0	0	1	0	0	0.885
PF3D7_0204800	3'-5'_exonuclease,_putative	1	0	0	1	0	0	0	0.885
PF3D7_0418500	trafficking_protein_particle_complex_subunit_3,_putative	1	0	0	1	0	0	0	0.885
PF3D7_0602200	MYND-type_zinc_finger_protein,_putative	1	0	0	1	0	0	0	0.885
PF3D7_0723600	proteasome_assembly_chaperone_4,_putative	1	0	0	1	0	0	0	0.885
PF3D7_1033400	haloacid_dehalogenase-like_hydrolase	1	0	0	1	0	0	0	0.885
PF3D7_0106500	conserved_Plasmodium_protein,_unknown_function	1	0	0	0	0	0	0	0.885
PF3D7_0107800	double-strand_break_repair_protein_MRE11	1	0	0	0	0	0	0	0.885
PF3D7_0114000	exported_protein_family_1	1	0	0	0	0	0	0	0.885
PF3D7_0202600	nucleic_acid_binding_protein,_putative	1	0	0	0	0	0	0	0.885
PF3D7_0203600	conserved_Plasmodium_protein,_unknown_function	1	0	0	0	0	0	0	0.885
PF3D7_0215100	RING_zinc_finger_protein,_putative	1	0	0	0	0	0	0	0.885
PF3D7_0216100	conserved_Plasmodium_protein,_unknown_function	1	0	0	0	0	0	0	0.885
PF3D7_0218700	pre-mRNA-processing_protein_45,_putative	1	0	0	0	0	0	0	0.885
PF3D7_0305500	conserved_Plasmodium_protein,_unknown_function	1	0	0	0	0	0	0	0.885
PF3D7_0422300	alpha_tubulin_2	1	0	0	0	0	0	0	0.885
PF3D7_0513300	purine_nucleoside_phosphorylase	1	0	0	0	0	0	0	0.885
PF3D7_0514900	conserved_Plasmodium_protein,_unknown_function	1	0	0	0	0	0	0	0.885
PF3D7_0519600	zinc_finger_protein,_putative	1	0	0	0	0	0	0	0.885
PF3D7_0606500	polypyrimidine_tract-binding_protein,_putative	1	0	0	0	0	0	0	0.885
PF3D7_0610100	pre-mRNA-splicing_factor_SLU7,_putative	1	0	0	0	0	0	0	0.885
PF3D7_0623900	ribonuclease_H2_subunit_A,_putative	1	0	0	0	0	0	0	0.885
PF3D7_0707600	mediator_of_RNA_polymerase_II_transcription_subunit_10,_putative	1	0	0	0	0	0	0	0.885
PF3D7_0709600	ribonucleases_P/MRP_protein_subunit_POP1,_putative	1	0	0	0	0	0	0	0.885
PF3D7_0720700	phosphoinositide-binding_protein,_putative	1	0	0	0	0	0	0	0.885
PF3D7_0725000	exonuclease_I,_putative	1	0	0	0	0	0	0	0.885

PF3D7_0803100	U3_small_nucleolar_RNA-associated_protein_14,_putative	1	0	0	0	0	0	0	0.885
PF3D7_0809200	asparagine-rich_antigen_Pfa55-14	1	0	0	0	0	0	0	0.885
PF3D7_0817900	high_mobility_group_protein_B2	1	0	0	0	0	0	0	0.885
PF3D7_0825000	conserved_Plasmodium_protein,_unknown_function	1	0	0	0	0	0	0	0.885
PF3D7_0826900	conserved_Plasmodium_protein,_unknown_function	1	0	0	0	0	0	0	0.885
PF3D7_0905100	nucleoporin_NUP221,_putative	1	0	0	0	0	0	0	0.885
PF3D7_0928300	conserved_Plasmodium_protein,_unknown_function	1	0	0	0	0	0	0	0.885
PF3D7_1019600	conserved_Plasmodium_protein,_unknown_function	1	0	0	0	0	0	0	0.885
PF3D7_1025900	conserved_protein,_unknown_function	1	0	0	0	0	0	0	0.885
PF3D7_1031300	conserved_Plasmodium_protein,_unknown_function	1	0	0	0	0	0	0	0.885
PF3D7_1126400	mediator_of_RNA_polymerase_II_transcription_subunit_21,_putative	1	0	0	0	0	0	0	0.885
PF3D7_1127600	CRAL/TRIO_domain-containing_protein,_putative	1	0	0	0	0	0	0	0.885
PF3D7_1133200	conserved_Plasmodium_protein,_unknown_function	1	0	0	0	0	0	0	0.885
PF3D7_1146000	ribosome_assembly_protein_4,_putative	1	0	0	0	0	0	0	0.885
PF3D7_1227600	conserved_Plasmodium_protein,_unknown_function	1	0	0	0	0	0	0	0.885
PF3D7_1230900	serine/threonine_protein_kinase_RIO1,_putative	1	0	0	0	0	0	0	0.885
PF3D7_1252600	esterase,_putative	1	0	0	0	0	0	0	0.885
PF3D7_1311200	alternative_splicing_regulator,_putative	1	0	0	0	0	0	0	0.885
PF3D7_1328000	conserved_Plasmodium_protein,_unknown_function	1	0	0	0	0	0	0	0.885
PF3D7_1334500	MSP7-like_protein	1	0	0	0	0	0	0	0.885
PF3D7_1344100	TLD_domain-containing_protein,_putative	1	0	0	0	0	0	0	0.885
PF3D7_1345600	inner_membrane_complex_protein	1	0	0	0	0	0	0	0.885
PF3D7_1346500	conserved_Plasmodium_protein,_unknown_function	1	0	0	0	0	0	0	0.885
PF3D7_1353600	ER_lumen_protein_retaining_receptor	1	0	0	0	0	0	0	0.885
PF3D7_1356400	phosphatase_2A_regulatory_subunit-related_protein,_putative	1	0	0	0	0	0	0	0.885
PF3D7_1364200	nucleoporin_NUP205,_putative	1	0	0	0	0	0	0	0.885
PF3D7_1364400	conserved_Plasmodium_protein,_unknown_function	1	0	0	0	0	0	0	0.885

PF3D7_1415400	conserved_Plasmodium_protein,_unknown_function	1	0	0	0	0	0	0	0.885
PF3D7_1416900	prefoldin_subunit_2,_putative	1	0	0	0	0	0	0	0.885
PF3D7_1419600	conserved_Plasmodium_protein,_unknown_function	1	0	0	0	0	0	0	0.885
PF3D7_1432900	SF-assemblin,_putative	1	0	0	0	0	0	0	0.885
PF3D7_1441100	conserved_Plasmodium_protein,_unknown_function	1	0	0	0	0	0	0	0.885
PF3D7_1449500	AP2_domain_transcription_factor_AP2-O5,_putative	1	0	0	0	0	0	0	0.885
PF3D7_1451000	conserved_Plasmodium_protein,_unknown_function	1	0	0	0	0	0	0	0.885
PF3D7_1457500	vacuolar_protein_sorting-associated_protein_4	1	0	0	0	0	0	0	0.885
PF3D7_1463300	DNA_polymerase_alpha_subunit_B,_putative	1	0	0	0	0	0	0	0.885
PF3D7_1466200	early_gametocyte_enriched_phosphoprotein_EGXP	1	0	0	0	0	0	0	0.885
PF3D7_1467900	rab_GTPase_activator,_putative	1	0	0	0	0	0	0	0.885
PF3D7_1468400	zinc_finger_protein,_putative	1	0	0	0	0	0	0	0.885
PF3D7_1469200	shewanella-like_protein_phosphatase_1,_putative	1	0	0	0	0	0	0	0.885
PF3D7_1473100	GTPase-activating_protein,_putative	1	0	0	0	0	0	0	0.885

Table S3A. Differential expression between PfDNMT2 disruptant and wildtype parasite at the ring stage

R: replicate, WT: 3D7 wild type, KO: PfDNMT2 disruptant, Adj.Pval: adjusted P value

columns G-L is EdgeR log2 (counts per million + count)

Change pattern	Gene ID	log2 Fold Change	Adj.Pval	WT R1	WT R2	WT R3	KO R1	KO R2	KO R3	Product Description	Name or Symbol
Up	PF3D7_0425500	1.480657	7.58E-05	6	5	5.55	6.9	7	7.2	stevor	N/A
Down	PF3D7_0302300	-2.22014	2.65E-29	8.5	8.8	8.55	6.2	6	6.7	erythrocyte membrane protein 1 (PfEMP1), pseudogene	N/A
Down	PF3D7_0302500	-2.11286	9.80E-03	5.2	5.4	5.25	3.2	4	3.8	cytoadherence linked asexual protein 3.1	CLAG3.1
Down	PF3D7_0412400	-1.44528	1.13E-28	11	11	10.8	9.2	9	9.4	erythrocyte membrane protein 1, PfEMP1	VAR
Down	PF3D7_0412700	-1.40929	5.62E-19	9.1	9.5	9.23	7.7	8	8	erythrocyte membrane protein 1, PfEMP1	VAR
Down	PF3D7_0420900	-1.44426	1.76E-06	7.1	7.4	7.08	5.6	6	5.8	erythrocyte membrane protein 1, PfEMP1	VAR
Down	PF3D7_0421100	-1.16305	2.29E-04	7	7.4	7.12	6	6	6	erythrocyte membrane protein 1, PfEMP1	VAR
Down	PF3D7_0421300	-1.53558	5.30E-21	9.3	9.7	9.39	7.9	8	8.1	erythrocyte membrane protein 1, PfEMP1	VAR
Down	PF3D7_0425200	-1.29559	5.49E-03	6.6	6.6	6.52	5	6	5.1	Plasmodium exported protein (hyp15), unknown function	N/A
Down	PF3D7_0532800	-1.86674	7.09E-02	4.9	5.2	5.39	3.3	4	3.9	stevor, pseudogene	N/A
Down	PF3D7_0532900	-2.18183	8.32E-03	5.1	5.4	5.09	3.5	4	3.5	rifin	RIF
Down	PF3D7_0600200	-1.52485	2.21E-10	7.8	8.5	8.24	6.6	7	6.6	erythrocyte membrane protein 1, PfEMP1	VAR
Down	PF3D7_0632500	-1.25219	1.76E-16	9.6	9.9	9.62	8.3	9	8.6	erythrocyte membrane protein 1, PfEMP1	VAR
Down	PF3D7_0632800	-1.29476	6.92E-03	6.2	6.8	6.57	5	5	5.4	erythrocyte membrane protein 1, PfEMP1	VAR
Down	PF3D7_0711700	-3.21258	########	12	13	12.5	9.2	9	9.5	erythrocyte membrane protein 1, PfEMP1	VAR
Down	PF3D7_0712600	-1.44256	1.08E-17	9.4	9.9	9.35	8	8	8.3	erythrocyte membrane protein 1, PfEMP1	VAR
Down	PF3D7_0712800	-2.96055	4.45E-35	8.7	8.9	8.13	5.8	6	5.8	erythrocyte membrane protein 1, PfEMP1	VAR
Down	PF3D7_0712900	-0.68333	6.47E-04	9.6	9.9	9.44	8.9	9	9.2	erythrocyte membrane protein 1, PfEMP1	VAR
Down	PF3D7_0713300	-0.87665	2.18E-05	8.2	8.4	8.27	7.4	7	7.4	erythrocyte membrane protein 1 (PfEMP1), pseudogene	VAR
Down	PF3D7_0809100	-0.80775	1.18E-05	9.1	9.4	9.02	8.4	8	8.5	erythrocyte membrane protein 1, PfEMP1	VAR
Down	PF3D7_1150400	-0.85317	7.51E-02	7.1	7.5	7.08	6.1	6	6.7	erythrocyte membrane protein 1, PfEMP1	VAR

Down	PF3D7_1219300	-1.85461	4.82E-13	7.5	8	7.66	5.9	6	6.1	erythrocyte membrane protein 1, PfEMP1	VAR
Down	PF3D7_1219400	-1.99351	2.54E-37	9.4	9.7	9.62	7.4	8	7.7	erythrocyte membrane protein 1 (PfEMP1), pseudogene	VAR
Down	PF3D7_1240300	-0.76167	1.07E-02	7.9	8.3	8.04	7.3	7	7.6	erythrocyte membrane protein 1, PfEMP1	VAR
Down	PF3D7_1240400	-2.46608	1.34E-77	11	12	11.3	8.8	9	9.1	erythrocyte membrane protein 1, PfEMP1	VAR
Down	PF3D7_1240500	-3.167	5.56E-02	4	4.4	4.86	2.3	2	3	Plasmodium RNA of unknown function RUF6	N/A
Down	PF3D7_1240600	-2.79027	########	13	14	13.3	11	10	11	erythrocyte membrane protein 1, PfEMP1	VAR
Down	PF3D7_1253000	-1.30046	2.59E-11	9.2	9.4	8.99	8.1	8	7.5	gametocyte erythrocyte cytosolic protein	GECO
Down	PF3D7_1477000	-1.31316	1.22E-20	11	11	11.4	9.8	10	9.7	Plasmodium exported protein (hyp17), unknown function, pseudogene	N/A
Down	PF3D7_1477300	-1.92074	4.08E-02	5.4	5	5.55	3.9	3	4.2	Plasmodium exported protein (PHIST), unknown function	Pfg14-744

Table S3B. Differential expression between PfDNMT2 disruptant and wildtype parasite at the trophozoite stage

R: replicate, WT: 3D7 wild type, KO: PfDNMT2 disruptant, Adj.Pval: adjusted P value

columns G-L is EdgeR log2 (counts per million + count)

Change pattern	Gene ID	log2 Fold Change	Adj.Pval	WT R1	WT R2	WT R3	KO R1	KO R2	KO R3	Product Description	Name or Symbol
Up	PF3D7_0725700	4.44352	3.84E-02	2	2	2	3	3	3		N/A
Up	PF3D7_1242100	3.31156	1.87E-07	3	3.6	2.3	5.17	5.81	5.62	conserved Plasmodium protein, unknown function	N/A
Up	PF3D7_0221100	3.17102	8.20E-02	2.32	2	2.3	2.58	3.81	3.32	Plasmodium exported protein, unknown function, pseudogene	N/A
Up	PF3D7_1107600	3.17094	7.34E-02	2.32	2	2.3	3.32	3	3.59	conserved Plasmodium protein, unknown function	N/A
Up	PF3D7_0926200	3.13207	1.13E-09	3.46	3.8	3	5.55	6.41	6	conserved Plasmodium protein, unknown function	N/A
Up	PF3D7_1130310	3.10508	3.32E-03	2.59	2.6	2.3	4.25	4.32	4	small nucleolar RNA snoR14a	
Up	PF3D7_0512900	2.577	4.76E-07	3.91	3.5	3.7	5.17	6.23	5.96	AKAP-like protein	AKAL
Up	PF3D7_API01600	2.32275	1.58E-02	2.59	2.8	2.8	4.25	4	4.09	apicoplast ribosomal protein S19	RPS19
Up	PF3D7_1462500	2.23347	1.26E-02	2.32	3.2	3	4.25	4.46	4.17	conserved Plasmodium protein, unknown function	N/A
Up	PF3D7_1346200	2.18846	2.52E-02	2.59	2.8	3	4.52	3.46	4.25	nuclear import protein MOG1, putative	N/A
Up	PF3D7_1039200	2.15605	5.36E-04	3.91	2.8	3.6	5.59	5.09	4.81	Plasmodium exported protein, unknown function, pseudogene	N/A
Up	PF3D7_0107700	2.13544	3.64E-07	3.7	4.8	4.2	5.98	6.41	6.05	dolichyl-diphosphooligosaccharide--protein glycosyltransferase subunit OST3/OST6, putative	N/A
Up	PF3D7_1315000	2.09614	4.76E-07	4.25	3.9	4.5	5.7	6.21	6.21	conserved protein, unknown function	N/A
Up	PF3D7_0934200	2.06516	2.82E-06	3.81	3.9	4.8	5.7	6.21	6.13	conserved protein, unknown function	N/A
Up	PF3D7_0916900	2.01487	1.48E-03	5.81	7.1	5.5	7.89	8.48	8.39	zinc finger protein, putative	N/A
Up	PF3D7_0209900	1.96766	2.55E-02	3	2.8	3	4.25	4.32	4	non-coding RNA	N/A
Up	PF3D7_API03000	1.90767	7.75E-02	2.81	2.6	2.8	4.09	3.7	3.59	probable protein, unknown function	ORF78
Up	PF3D7_1039100	1.75575	2.30E-02	3.46	2.8	3.3	4.46	4.64	4.25	DnaJ protein, putative, pseudogene	N/A

Up	PF3D7_1444600	1.7557	2.56E-02	2.81	3.6	3.2	4.17	4.76	4.39	conserved Plasmodium protein, unknown function	N/A
Up	PF3D7_0620300	1.73778	2.42E-03	4.59	3.9	3.2	4.95	5.81	5.49	conserved Plasmodium protein, unknown function	N/A
Up	PF3D7_1133000	1.653	6.03E-02	3.59	2.8	2.8	3.91	4.25	4.46	conserved Plasmodium protein, unknown function	N/A
Up	PF3D7_0626200	1.58556	9.77E-03	3.46	4.6	3.3	5.21	5.49	4.86	conserved Plasmodium protein, unknown function	N/A
Up	PF3D7_0511700	1.57112	4.85E-03	3.59	4.3	3.7	4.95	5.39	5.21	EKC/KEOPS complex subunit CGI121	CGI121
Up	PF3D7_1310000	1.56113	1.34E-04	4.59	4.9	4.2	5.67	6.05	6.11	ATP synthase subunit O, mitochondrial, putative	OSCP
Up	PF3D7_1452100	1.53129	1.11E-02	3.59	3.9	3.6	5	4.86	4.86	leucine-rich repeat protein	LRR3
Up	PF3D7_1446000	1.52803	8.48E-02	3.81	2.6	3.2	4.81	3.7	4.32	U5 spliceosomal RNA	N/A
Up	PF3D7_1210300	1.52241	1.67E-04	4.52	4.9	4.4	5.73	5.88	6.29	conserved Plasmodium protein, unknown function	N/A
Up	PF3D7_1135000	1.48803	3.44E-08	5.67	6.4	5.5	7.18	7.44	7.37	PQ-loop repeat-containing protein	N/A
Up	PF3D7_1313200	1.42572	1.28E-05	5.17	5.1	5.1	6.19	6.41	6.66	methionyl-tRNA formyltransferase, putative	MTFMT
Up	PF3D7_0815700	1.42409	4.33E-02	3.7	3.5	3.3	4.25	4.76	4.65	ubiquitin	Ub
Up	PF3D7_0915500	1.42407	4.80E-02	3.7	3.2	3.6	4.46	4.91	4.25	conserved Plasmodium protein, unknown function	N/A
Up	PF3D7_1363900	1.39462	8.02E-05	5.04	6.1	5.8	6.46	7.44	7.04		
Up	PF3D7_1133500	1.39323	9.59E-05	4.64	4.9	5.2	6.04	6.38	6.19	conserved protein, unknown function	N/A
Up	PF3D7_1016200	1.38922	7.52E-10	6.17	6.1	6	7.24	7.54	7.45	Rab3 GTPase-activating protein non-catalytic subunit, putative	N/A
Up	PF3D7_0829700	1.38549	1.30E-02	3.81	4.3	3.8	4.81	5.39	5.17	non-coding RNA	N/A
Up	PF3D7_1303300	1.36962	1.28E-05	5.32	5.4	5	6.32	6.62	6.65	conserved Plasmodium protein, unknown function	N/A
Up	PF3D7_1364700	1.33203	2.39E-02	4	3.8	3.7	4.86	5	4.86	WD repeat-containing protein, putative, pseudogene	N/A
Up	PF3D7_0921100	1.32285	6.38E-03	4	4.2	4.5	5.25	5.49	5.46	conserved Plasmodium protein, unknown function	N/A
Up	PF3D7_1101700	1.27626	3.40E-02	4.39	4.1	3.6	5.61	4.64	4.91	Pfmc-2TM Maurer's cleft two transmembrane protein	MC-2TM
Up	PF3D7_1208200	1.27528	4.60E-03	4.7	4.9	4.2	6.15	5.49	5.53	cysteine repeat modular protein 3	CRMP3

Up	PF3D7_0811800	1.2716	5.07E-05	5.32	6.2	5.3	6.71	6.98	6.83	conserved Plasmodium protein, unknown function	N/A
Up	PF3D7_1370800	1.26747	5.34E-12	6.67	6.8	6.6	7.94	7.97	7.86	non-coding RNA	N/A
Up	PF3D7_1470000	1.2483	4.85E-06	5.59	6.3	5.8	6.91	7.31	7.12	autophagy-related protein 12, putative	ATG12
Up	PF3D7_1413600	1.24617	7.77E-02	3.59	3.5	4	4.52	4.17	5.13	conserved Plasmodium protein, unknown function	N/A
Up	PF3D7_1339800	1.24068	1.31E-02	3.81	4.5	4.5	5.32	5.49	5.25	mitotic-spindle organizing protein 1, putative	MZT1
Up	PF3D7_0526100	1.2294	7.54E-03	4.09	5.2	4.6	5.49	5.81	6.09	conserved Plasmodium membrane protein, unknown function	N/A
Up	PF3D7_0618800	1.2091	1.01E-06	5.78	6.2	6.2	7.08	7.42	7.18	heptatricopeptide repeat-containing protein, putative	N/A
Up	PF3D7_1112800	1.19636	7.13E-02	4.39	3.6	3.3	4.75	4.7	4.91	conserved protein, unknown function	N/A
Up	PF3D7_1217500	1.19491	3.12E-10	7.74	8.3	7.7	8.87	9.36	9.13	conserved Plasmodium protein, unknown function	N/A
Up	PF3D7_1001300	1.19247	1.46E-03	5.36	5.8	4.5	6.52	6.38	6.43	Plasmodium exported protein (PHISTa), unknown function	N/A
Up	PF3D7_1038500	1.19148	1.77E-02	4.32	4.5	4	5.32	5.13	5.39	Plasmodium exported protein, unknown function	N/A
Up	PF3D7_0210700	1.17656	2.52E-02	4.17	5.1	3.8	5.29	5.59	5.56	syntaxin, Qa-SNARE family	SYN17
Up	PF3D7_1216100	1.17324	3.34E-05	5.52	5.6	5.5	6.55	6.73	6.72	conserved protein, unknown function	N/A
Up	PF3D7_0912600	1.16823	6.27E-03	4.59	5	4.4	5.61	5.86	5.67	conserved Plasmodium protein, unknown function	N/A
Up	PF3D7_1148200	1.12648	2.27E-12	8.01	7.8	7.4	8.96	8.87	8.84	non-coding RNA	N/A
Up	PF3D7_0411100	1.12443	3.87E-05	5.7	6.2	5.6	7	6.92	6.86	mediator of RNA polymerase II transcription subunit 8, putative	MED8
Up	PF3D7_1437600	1.12342	9.46E-03	4.52	4.6	4.7	5.81	5.52	5.49	conserved protein, unknown function	N/A
Up	PF3D7_1479000	1.12295	6.90E-02	4.17	3.6	4	4.7	4.7	5.09	acyl-CoA synthetase	ACS1a
Up	PF3D7_0929700	1.12112	1.24E-03	5.21	5.2	4.9	6.09	6.13	6.21	conserved Plasmodium protein, unknown function	N/A
Up	PF3D7_0608310	1.11604	1.13E-10	6.92	7.3	7.1	8.13	8.24	8.24	sorting assembly machinery 50 kDa subunit, putative	SAM50
Up	PF3D7_1335600	1.11071	5.89E-05	5.83	6.1	6	6.66	7.32	7.06	WD repeat-containing protein, putative	N/A

Up	PF3D7_0823700	1.10693	4.07E-04	5.29	5.6	5.5	6.39	6.3	6.69	mitochondrial import receptor subunit TOM7, putative	TOM7
Up	PF3D7_1438600	1.10082	2.26E-06	6.09	6.5	6.1	7.17	7.35	7.39	Golgi to ER traffic protein 4	GET4
Up	PF3D7_1139200	1.09674	4.27E-03	4.91	5.1	4.8	5.95	5.76	6.07	VPS9 domain-containing protein, putative	N/A
Up	PF3D7_1469500	1.09504	6.38E-02	4	4.3	3.7	5.09	4.86	4.81	conserved Plasmodium protein, unknown function	N/A
Up	PF3D7_1022200	1.09369	1.99E-13	7.85	8.3	8.1	9.05	9.26	9.28	conserved Plasmodium membrane protein, unknown function	N/A
Up	PF3D7_0707500	1.09241	2.78E-06	6.44	6.1	6.4	7.23	7.61	7.28	conserved Plasmodium protein, unknown function	N/A
Up	PF3D7_0515100	1.08397	1.13E-06	6.57	6.3	7	7.55	7.84	7.67	rhomboid protease ROM9	ROM9
Up	PF3D7_1122300	1.07253	2.86E-02	4.52	4.5	4.7	4.91	5.83	5.62	conserved Plasmodium protein, unknown function	N/A
Up	PF3D7_0104900	1.06887	4.67E-05	5.73	6.2	5.9	6.88	7.05	7.04	RNase MRP	N/A
Up	PF3D7_1459300	1.06497	1.29E-02	5.36	4.6	4.5	5.64	6.05	5.83	OPA3-like protein, putative	N/A
Up	PF3D7_1142200	1.06131	8.10E-02	4.17	3.9	3.8	5	4.76	4.7	conserved Plasmodium protein, unknown function	N/A
Up	PF3D7_0218800	1.05671	2.31E-08	6.67	7	6.8	7.95	7.87	7.74	ribonuclease P	N/A
Up	PF3D7_1370500	1.05456	1.69E-11	7.7	7.7	7.3	8.68	8.64	8.56	non-coding RNA	N/A
Up	PF3D7_1354100	1.05052	3.03E-02	4.76	4.6	4.2	5.32	5.73	5.21	conserved Plasmodium protein, unknown function	N/A
Up	PF3D7_0630500	1.04859	8.98E-06	6.46	6.8	6.1	7.52	7.53	7.37	ribosome biogenesis protein YTM1, putative	YTM1
Up	PF3D7_1313900	1.04598	2.19E-04	5.76	6.3	5.7	6.69	7.06	7.06	ankyrin-repeat protein, putative	N/A
Up	PF3D7_0109300	1.03792	2.05E-03	5.73	5.4	5	6.39	6.39	6.32	elongation of fatty acids protein, putative	N/A
Up	PF3D7_1007500	1.03692	3.54E-04	5.81	6	5.6	6.49	6.93	6.96	conserved Plasmodium protein, unknown function	N/A
Up	PF3D7_0721500	1.0366	1.89E-05	6.23	6.5	6.1	7.07	7.44	7.26	conserved Plasmodium membrane protein, unknown function	N/A
Up	PF3D7_1409000	1.03559	1.19E-09	7.11	7.3	7	8.07	8.22	8.15	WD repeat-containing protein, putative	N/A
Up	PF3D7_0621000	1.03444	1.66E-10	7.71	8.1	7.6	8.71	8.86	8.89	conserved Plasmodium protein, unknown function	N/A
Up	PF3D7_0809800	1.03398	5.96E-02	4.17	4.2	4.2	4.95	5.32	4.86	conserved Plasmodium protein, unknown function	N/A

Up	PF3D7_1139900	1.02911	1.15E-06	6.78	7.6	7.1	8.34	8.2	8.06	ER membrane protein complex subunit 8, putative	EMC8
Up	PF3D7_0308200	1.02482	1.32E-14	9.16	9.6	9.6	10.4	10.7	10.4	T-complex protein 1 subunit eta	CCT7
Up	PF3D7_0931200	1.02245	2.63E-03	5.32	6.2	6	6.39	7.2	6.88	selenoprotein	Sel2
Up	PF3D7_0301900	1.01172	2.07E-02	4.52	4.9	4.6	5.46	5.76	5.56	conserved protein, unknown function	N/A
Up	PF3D7_0525300	1.01162	2.80E-02	4.59	5.1	4.2	5.49	5.73	5.56	conserved protein, unknown function	N/A
Up	PF3D7_1251400	1.00994	1.00E-02	4.95	5	4.8	5.86	5.67	5.96	conserved Plasmodium protein, unknown function	N/A
Up	PF3D7_1117300	1.00803	5.89E-06	6.36	6.8	6.9	7.51	7.69	7.88	conserved protein, unknown function	N/A
Up	PF3D7_0510400	1.0059	1.43E-03	5.43	6.1	5.4	6.59	6.62	6.63	PDCD2 domain-containing protein, putative	N/A
Up	PF3D7_1456300	1.00071	2.38E-02	4.81	4.9	4.5	5.83	5.67	5.32	conserved Plasmodium protein, unknown function	N/A
Up	PF3D7_0320600	0.99557	1.68E-03	5.13	5.9	5.8	6.51	6.64	6.56	conserved protein, unknown function	N/A
Up	PF3D7_1111000	0.99415	1.60E-05	6.09	6.3	6.3	7.25	7.15	7.21	RNA cytosine C(5)-methyltransferase NSUN2	NSUN2
Up	PF3D7_1428000	0.98952	3.54E-02	4.95	4.6	4.4	5.25	5.86	5.43	conserved Plasmodium membrane protein, unknown function	N/A
Up	PF3D7_0905500	0.98407	1.88E-02	4.7	5.2	5.1	5.43	6.07	6.17	conserved protein, unknown function	N/A
Up	PF3D7_0303600	0.98382	1.30E-02	4.59	5.2	5.2	5.75	5.93	6.02	plasmoredoxin	Plrx
Up	PF3D7_0919600	0.98314	6.70E-05	5.98	6.4	6.1	6.98	7.18	7.19	dolichyl-diphosphooligosaccharide--protein glycosyltransferase subunit WBP1, putative	WBP1
Up	PF3D7_0829800	0.9792	5.11E-13	7.9	8.2	8.2	8.97	9.09	9.11	non-coding RNA	N/A
Up	PF3D7_1222900	0.97613	3.34E-05	6.63	7	6.3	7.67	7.76	7.44	conserved Plasmodium protein, unknown function	N/A
Up	PF3D7_0514800	0.97051	1.59E-11	7.68	8	7.8	8.73	8.85	8.8	inositol polyphosphate multikinase, putative	N/A
Up	PF3D7_1476700	0.96871	3.35E-02	4.52	4.9	4.8	5.59	5.21	5.91	lysophospholipase LPL1	LPL1
Up	PF3D7_0513500	0.96172	3.75E-03	5.49	5.2	5.4	6.11	6.27	6.39	mitochondrial import inner membrane translocase subunit TIM16, putative	PAM16
Up	PF3D7_0935300	0.94728	2.38E-04	5.86	6.5	6.1	7.19	7.02	7.06	phosphatidylinositol N-acetylglucosaminyltransferase subunit P, putative	N/A

Up	PF3D7_1011600	0.94585	7.86E-03	5.52	5	5.4	6.39	6.11	6.02	heptatricopeptide repeat-containing protein, putative	N/A
Up	PF3D7_1220500	0.93435	7.49E-03	5.17	5.6	5.2	6.02	6.27	6.36	ribosome biogenesis protein TSR3, putative	TSR3
Up	PF3D7_0517100	0.92873	9.64E-04	6.04	5.6	6	6.73	6.85	6.69	conserved protein, unknown function	N/A
Up	PF3D7_0425600	0.91941	3.66E-02	4.7	5.1	4.6	5.83	5.73	5.32	rifin, pseudogene	RIF
Up	PF3D7_0822200	0.91503	8.48E-02	4.39	4.8	4	5.04	5.36	5.17	phosphorylated CTD interacting factor 1 WW domain-containing protein, putative	N/A
Up	MAL_MITO_1	0.91346	9.74E-04	6.36	7.4	6.6	7.55	7.71	7.91		
Up	PF3D7_0922400	0.91189	2.66E-07	7.2	7.5	7.2	8.06	8.32	8.19	para-aminobenzoic acid synthetase	pBAS
Up	PF3D7_1360400	0.91066	6.41E-05	6.43	7	6.5	7.49	7.68	7.41	conserved Plasmodium protein, unknown function	N/A
Up	PF3D7_1011300	0.91033	2.65E-04	6.04	6.2	6.6	7.09	7.28	7.26	protein ARV1, putative	ARV1
Up	PF3D7_0407500	0.90523	2.55E-05	6.71	6.6	6.4	7.44	7.53	7.36	mitochondrial carrier protein, putative	N/A
Up	MALMITO_SSUB	0.90402	3.08E-02	4.91	4.6	5.1	5.83	5.52	5.73		
Up	PF3D7_0817000	0.9004	6.02E-03	5.39	5.6	5.3	6.3	6.23	6.32	NEDD8-activating enzyme E1 catalytic subunit, putative	UBA3
Up	PF3D7_0916600	0.89591	1.47E-02	5.04	5.7	5.1	6.23	6.07	6.11	methyltransferase, putative	N/A
Up	PF3D7_1253100	0.89513	1.53E-03	5.91	6.2	5.9	6.71	7.1	6.63	Plasmodium exported protein (PHISTa), unknown function	N/A
Up	PF3D7_1029000	0.88841	7.54E-02	4.52	4.6	4.6	5	5.73	5.25	conserved Plasmodium protein, unknown function, pseudogene	N/A
Up	PF3D7_1351800	0.88672	4.21E-03	5.78	5.4	5.7	6.32	6.63	6.46	conserved Plasmodium protein, unknown function	N/A
Up	PF3D7_1461000	0.88148	1.18E-02	5.43	5.9	5.1	6.15	6.46	6.41	conserved Plasmodium protein, unknown function	N/A
Up	PF3D7_0412200	0.88134	2.51E-02	5.04	4.8	5.3	5.86	5.83	5.88	conserved Plasmodium protein, unknown function	N/A
Up	PF3D7_1349800	0.87906	8.21E-03	5.32	6	5.4	6.54	6.46	6.34	GPN-loop GTPase, putative	N/A
Up	PF3D7_1225900	0.87767	3.17E-06	7.76	8.3	7.6	8.57	8.84	8.9	conserved Plasmodium protein, unknown function	N/A
Up	PF3D7_0425500	0.87529	2.36E-03	5.76	5.7	5.8	6.7	6.51	6.53	stevor	N/A
Up	PF3D7_1316200	0.87378	3.98E-02	5.36	4.8	4.8	5.52	5.81	5.96	ADP-ribosylation factor, putative	N/A
Up	PF3D7_0901900	0.86756	9.43E-02	3.91	5.2	4.8	5.32	5.56	5.62	probable protein, unknown function	N/A

Up	PF3D7_1016000	0.86652	9.50E-03	5.36	6	5.4	6.23	6.48	6.56	conserved Plasmodium protein, unknown function	N/A
Up	PF3D7_0727600	0.8654	1.31E-03	6.02	6	5.8	6.71	6.85	6.74	conserved Plasmodium protein, unknown function	N/A
Up	PF3D7_1324600	0.85736	3.83E-06	7.77	7.8	8.1	8.45	9.03	8.68	conserved Plasmodium protein, unknown function	N/A
Up	PF3D7_0703100	0.85455	4.93E-02	5.29	5.1	4.4	5.73	5.64	5.88	cytosolic iron-sulfur assembly component 2, putative	CIA2
Up	PF3D7_1230500	0.85256	9.53E-03	5.29	5.8	5.5	6.32	6.3	6.39	WD repeat-containing protein, putative	N/A
Up	PF3D7_1347300	0.84782	2.96E-03	5.91	5.9	5.7	6.7	6.48	6.72	conserved Plasmodium membrane protein, unknown function	N/A
Up	PF3D7_1037400	0.84713	3.25E-03	6.3	6.3	5.6	6.82	7.12	6.86	conserved Plasmodium protein, unknown function	N/A
Up	PF3D7_0708300	0.84277	3.54E-04	6.48	6.5	6.2	7.29	7.27	7.06	EKC/KEOPS complex subunit BUD32	BUD32
Up	PF3D7_0419200	0.83921	6.97E-03	5.49	5.9	6.2	6.54	6.56	6.91	protein transport protein GOT1, putative	GOT1
Up	PF3D7_1458800	0.83753	1.67E-05	6.92	6.9	6.8	7.62	7.63	7.82	DNA-directed RNA polymerase III subunit RPC5, putative	N/A
Up	PF3D7_0523700	0.83218	1.50E-04	6.43	6.8	6.5	7.37	7.45	7.43	conserved Plasmodium membrane protein, unknown function	N/A
Up	PF3D7_1462900	0.8262	1.87E-03	6.23	6	6.1	7.01	6.94	6.67	AAA ATPase, putative	N/A
Up	PF3D7_0422600	0.81977	4.04E-02	4.95	4.9	5.3	5.95	5.64	5.83	conserved Plasmodium protein, unknown function	N/A
Up	PF3D7_1022300	0.81957	6.97E-03	6.02	6.2	5.6	7	6.73	6.53	ZIP domain-containing protein, putative	ZIPCO
Up	PF3D7_1354600	0.81905	1.14E-03	6.13	6.3	6.1	7.09	6.91	6.92	60S ribosomal protein L7-2, putative	N/A
Up	PF3D7_0725600	0.81888	3.06E-07	7.54	7.9	7.7	8.59	8.54	8.38	18S ribosomal RNA	N/A
Up	PF3D7_0520700	0.81381	4.57E-06	7.15	7.1	7.1	7.86	8	7.86	CDC73 domain-containing protein, putative	N/A
Up	PF3D7_1209100	0.812	1.34E-02	5.67	6.2	5.5	6.41	6.83	6.53	conserved Plasmodium protein, unknown function	N/A
Up	PF3D7_1369400	0.81166	4.98E-07	8.21	7.9	8	8.61	9.06	8.84	conserved Plasmodium protein, unknown function	N/A
Up	PF3D7_1368500	0.80314	2.21E-02	5.09	5.6	5.7	6.07	6.29	6.29	heptatricopeptide repeat-containing protein, putative	N/A
Up	PF3D7_0531200	0.80247	1.07E-02	5.73	5.5	5.9	6.64	6.3	6.39	ribosomal protein S16, mitochondrial, putative	N/A

Up	PF3D7_1436900	0.79884	3.14E-02	5.64	5.2	5.1	6.29	5.83	6.13	histidine triad protein, putative	N/A
Up	PF3D7_1208500	0.79596	3.17E-04	6.81	6.6	6.5	7.29	7.36	7.56	conserved Plasmodium protein, unknown function	N/A
Up	PF3D7_0723000	0.79544	1.29E-03	6.13	6.3	6.2	6.99	6.91	7.04	tRNAHis guanylyltransferase, putative	N/A
Up	PF3D7_1410000	0.7949	1.87E-06	7.37	7.7	7.4	8.19	8.32	8.32	ER membrane protein complex subunit 2, putative	EMC2
Up	PF3D7_1111700	0.79444	9.75E-04	6.51	6.3	6.3	6.94	7.23	7.28	conserved Plasmodium protein, unknown function	N/A
Up	PF3D7_0105000	0.7935	4.44E-02	5.36	5.4	5.4	5.49	6.39	6.27	non-coding RNA	N/A
Up	PF3D7_1449900	0.79121	6.23E-04	6.27	6.8	6.7	7.41	7.36	7.36	conserved protein, unknown function	N/A
Up	PF3D7_0631400	0.79067	9.32E-02	5.09	5.7	4.4	6.15	5.64	5.83	Pfmc-2TM Maurer's cleft two transmembrane protein	MC-2TM
Up	PF3D7_0629600	0.78926	1.22E-02	5.61	5.9	5.5	6.54	6.38	6.38	ribosomal RNA-processing protein 7, putative	N/A
Up	PF3D7_1117500	0.78666	4.26E-08	8.1	8.3	8.3	8.85	9.17	9.02	tyrosine--tRNA ligase	TyrRSapi
Up	PF3D7_0808400	0.7858	2.71E-04	6.97	6.9	7	7.48	8.01	7.64	coatomer subunit epsilon, putative	SEC28
Up	PF3D7_1122000	0.78521	5.28E-03	6.15	6.7	5.9	7.04	7.11	7.06	conserved protein, unknown function	N/A
Up	PF3D7_0211000	0.7769	8.07E-02	5	5.2	4.5	5.43	5.73	5.73	non-coding RNA	N/A
Up	PF3D7_1308600	0.77552	2.81E-04	6.71	6.6	6.6	7.43	7.28	7.45	conserved Plasmodium protein, unknown function	N/A
Up	PF3D7_1447400	0.77503	3.73E-02	5.29	5.6	5	5.95	5.98	6.13	conserved Plasmodium protein, unknown function	N/A
Up	PF3D7_0416600	0.77035	1.55E-03	6.23	6.3	6.6	7.17	6.97	7.2	prohibitin-like protein PHBL, putative	PHBL
Up	PF3D7_1245200	0.77034	6.97E-05	7.19	7.3	6.9	7.75	7.94	7.9	conserved Plasmodium membrane protein, unknown function	N/A
Up	PF3D7_0631000	0.77028	1.86E-02	5.36	5.6	5.8	6.23	6.32	6.36	tetratricopeptide repeat protein, putative	N/A
Up	PF3D7_0311900	0.76915	4.10E-05	6.99	7	6.9	7.75	7.74	7.67	heptatricopeptide repeat-containing protein, putative	N/A
Up	PF3D7_0525400	0.76548	7.24E-05	7.03	7.2	7	8.02	7.69	7.83	7-helix-1 protein	N/A
Up	PF3D7_0907100	0.76354	4.43E-06	7.91	8.3	7.8	8.68	8.91	8.71	conserved Plasmodium protein, unknown function	N/A
Up	PF3D7_1220200	0.76296	1.09E-02	5.67	6	6.1	6.39	6.8	6.74	conserved Plasmodium protein, unknown function, pseudogene	N/A

Up	PF3D7_0415700	0.75844	2.57E-02	5.55	5.9	5.5	6.04	6.43	6.57	conserved Plasmodium protein, unknown function	N/A
Up	PF3D7_1405200	0.75805	3.16E-02	5.32	5.9	5.7	5.98	6.56	6.57	trafficking protein particle complex subunit 1, putative	BET5
Up	PF3D7_0829900	0.75778	2.61E-07	8.07	8.4	8.4	8.9	9.19	8.97	non-coding RNA	N/A
Up	PF3D7_1002600	0.75602	2.77E-05	7.45	7.4	7.1	8.04	8.14	8.04	conserved Plasmodium protein, unknown function	N/A
Up	PF3D7_0407000	0.75092	1.80E-02	5.93	5.6	5.8	6.19	6.72	6.54	conserved Plasmodium protein, unknown function	N/A
Up	PF3D7_0218100	0.74563	2.44E-02	5.39	5.9	5.8	6.17	6.63	6.48	conserved protein, unknown function	N/A
Up	PF3D7_1317900	0.74471	2.37E-04	6.92	7.2	6.8	7.76	7.71	7.71	nucleolar complex protein 4, putative	NOC4
Up	PF3D7_1453600	0.74352	7.15E-04	6.52	6.9	6.6	7.44	7.44	7.39	RAP protein, putative	N/A
Up	PF3D7_1305500	0.74017	2.84E-02	5.36	5.4	5.7	6.21	6.17	6.19	mitogen-activated protein kinase phosphatase 1, putative	MKP1
Up	PF3D7_1475400	0.7389	3.83E-06	7.46	7.5	7.4	8.24	8.19	8.14	cysteine repeat modular protein 4	CRMP4
Up	PF3D7_1370900	0.73497	3.22E-05	7.68	7.4	7.3	8.26	8.17	8.12	non-coding RNA	N/A
Up	PF3D7_1202500	0.72928	1.52E-03	6.85	6.9	6.5	7.52	7.59	7.21	trimethylguanosine synthase, putative	TGS1
Up	PF3D7_1016600	0.72448	1.53E-03	6.41	6.5	6.6	7.29	7.1	7.23	Plasmodium exported protein (PHISTc), unknown function	N/A
Up	PF3D7_1248000	0.72392	1.32E-02	5.78	6.4	6	6.86	6.74	6.66	tRNA-splicing endonuclease, putative	N/A
Up	PF3D7_1317700	0.72315	3.91E-03	6.49	6.4	6.2	6.86	7.14	7.19	conserved Plasmodium protein, unknown function	N/A
Up	PF3D7_1405500	0.72287	4.15E-04	6.87	7.1	6.7	7.7	7.57	7.59	COBW domain-containing protein 1, putative	CBWD1
Up	PF3D7_1407100	0.72238	1.34E-05	8.13	8.1	8.5	8.88	9.18	8.88	rRNA 2'-O-methyltransferase fibrillarin, putative	NOP1
Up	PF3D7_1032800	0.72223	1.41E-05	7.4	7.6	7.4	8.11	8.14	8.3	leucine-rich repeat protein	LRR1
Up	PF3D7_0506300	0.71304	1.04E-08	8.36	8.4	8.3	9.09	9.13	8.98	conserved protein, unknown function	N/A
Up	PF3D7_1201600	0.71222	7.77E-02	4.95	5.2	5.1	5.67	5.73	5.83	NIMA related kinase 3	NEK3
Up	PF3D7_0816300	0.7104	1.84E-02	6.11	6.1	5.8	6.81	6.38	6.82	HAP2-GCS1 domain-containing protein, putative	N/A
Up	PF3D7_1122100	0.70866	5.76E-05	7.22	7.5	7.6	8.23	8.08	8.1	GPI transamidase component GPI16, putative	GPI16

Up	PF3D7_1475100	0.70579	8.36E-07	7.83	7.9	7.8	8.57	8.58	8.51	conserved Plasmodium protein, unknown function	N/A
Up	PF3D7_1422200	0.70453	2.01E-02	5.67	6	6.1	6.46	6.57	6.83	conserved Plasmodium protein, unknown function	N/A
Up	PF3D7_0526400	0.70382	2.05E-03	6.59	6.5	6.6	7.09	7.28	7.27	conserved Plasmodium protein, unknown function	N/A
Up	PF3D7_1358700	0.70246	3.15E-07	8.5	8.8	8.8	9.4	9.28	9.56	YOP1-like protein, putative	YOP1L
Up	PF3D7_0730100	0.70154	8.04E-04	7.07	6.9	6.7	7.63	7.67	7.45	tRNA pseudouridine synthase D, putative	N/A
Up	PF3D7_1138100	0.70118	8.77E-02	5.29	5.4	4.8	5.83	5.88	5.7	ubiquitin-related modifier 1, putative	URM1
Up	PF3D7_1142000	0.70114	2.03E-02	6.34	6.3	5.6	6.98	6.74	6.7	heptatricopeptide repeat-containing protein, putative	N/A
Up	PF3D7_1127300	0.69735	1.16E-03	6.71	6.8	6.7	7.29	7.56	7.41	tRNA (guanine-N(7)-)-methyltransferase, putative	N/A
Up	PF3D7_0903100	0.69685	4.44E-03	6.36	6.5	6.5	6.95	7.31	7.07	protein RER1, putative	RER1
Up	PF3D7_1329800	0.69611	6.51E-04	6.88	6.8	6.8	7.52	7.47	7.45	conserved Plasmodium protein, unknown function	N/A
Up	PF3D7_0912000	0.6936	9.10E-11	9.81	10	9.9	10.5	10.7	10.6	conserved Plasmodium protein, unknown function	N/A
Up	PF3D7_0930700	0.69334	1.07E-02	6.19	6.4	6	6.71	7.07	6.83	conserved Plasmodium protein, unknown function	N/A
Up	PF3D7_0819300	0.6923	2.56E-02	6.43	6.5	5.6	6.81	6.91	6.96	conserved Plasmodium protein, unknown function	N/A
Up	PF3D7_0525500	0.69209	3.94E-05	7.81	7.8	7.4	8.38	8.43	8.33	WD repeat-containing protein, putative	N/A
Up	PF3D7_0511900	0.68037	6.31E-02	5.43	5.7	5.1	6.11	6	6.09	conserved Plasmodium protein, unknown function	N/A
Up	PF3D7_1119900	0.68	6.36E-09	9.33	9.2	9.4	9.84	10.1	9.92	protein transport protein SEC16, putative	SEC16
Up	PF3D7_0911200	0.67826	3.64E-03	6.54	6.4	6.6	7.13	7.32	7.12	conserved Plasmodium protein, unknown function	N/A
Up	PF3D7_1112400	0.67569	2.26E-04	7.14	7.6	7.5	8.07	8.07	8.12	nucleic acid binding protein, putative	N/A
Up	PF3D7_1436700	0.67521	2.31E-04	7.23	7.3	7.2	8.01	7.77	7.93	conserved protein, unknown function	N/A
Up	PF3D7_0811200	0.67434	6.95E-08	8.86	8.9	8.9	9.39	9.73	9.54	ER membrane protein complex subunit 1, putative	EMC1
Up	PF3D7_1113200	0.67238	7.23E-02	5.36	6.2	5.3	6.17	6.29	6.41	conserved Plasmodium protein, unknown function	N/A

Up	PF3D7_1450300	0.66554	9.74E-04	6.94	7	6.9	7.44	7.67	7.59	NADPH--cytochrome P450 reductase, putative	CPR
Up	PF3D7_1459200	0.66455	1.64E-04	7.7	7.9	8.1	8.32	8.71	8.59	WD repeat-containing protein, putative	N/A
Up	PF3D7_0807400	0.66438	4.13E-02	5.59	5.7	5.6	6.15	6.3	6.31	coenzyme Q-binding protein COQ10 homolog, mitochondrial	COQ10
Up	PF3D7_0905000	0.66328	3.73E-02	5.81	5.9	5.5	6.27	6.43	6.46	ATP synthase-associated protein, putative	ATPTG4
Up	PF3D7_1433900	0.66158	2.69E-03	6.54	6.8	6.8	7.27	7.41	7.39	protein kinase, putative	N/A
Up	PF3D7_0627600	0.6601	4.23E-02	5.88	5.8	5.4	6.32	6.3	6.36	conserved Plasmodium protein, unknown function	N/A
Up	PF3D7_1224200	0.65979	2.74E-02	5.88	6	5.9	6.66	6.32	6.66	BRO1 domain-containing protein, putative	N/A
Up	PF3D7_0821500	0.65954	2.45E-02	6.23	5.8	5.9	6.59	6.62	6.63	ribosomal RNA small subunit methyltransferase NEP1, putative	NEP1
Up	PF3D7_1302900	0.65863	1.68E-03	7.24	7.5	7	8.06	7.78	7.88	conserved protein, unknown function	N/A
Up	PF3D7_0413800	0.65594	3.08E-02	5.7	6	6.1	6.57	6.73	6.41	50S ribosomal protein L10, putative	N/A
Up	PF3D7_1147600	0.65572	1.44E-04	7.48	7.3	7.6	8.12	8.2	7.98	conserved Plasmodium protein, unknown function	N/A
Up	PF3D7_1111300	0.65346	1.28E-02	6.29	6.7	6.5	6.82	7.32	7.16	protein transport protein BOS1, putative	BOS1
Up	PF3D7_1443700	0.65316	4.78E-05	7.61	7.8	7.8	8.25	8.48	8.44	dephospho-CoA kinase	DPCK
Up	PF3D7_0933300	0.65268	3.36E-09	8.95	8.9	9	9.52	9.68	9.63	conserved Plasmodium protein, unknown function	N/A
Up	PF3D7_0531600	0.65249	1.44E-02	6.32	6.5	6.9	7.16	7.45	6.98	18S ribosomal RNA	N/A
Up	PF3D7_1307300	0.65135	1.10E-03	7.33	7.3	6.9	7.73	7.78	7.93	ATP-dependent RNA helicase DBP6, putative	DBP6
Up	PF3D7_1013300	0.65011	2.16E-04	7.43	7.6	7.6	8.04	8.38	8.17	conserved Plasmodium protein, unknown function	N/A
Up	PF3D7_0210800	0.64978	6.13E-02	5.49	5.6	5.5	6.04	6.05	6.25	conserved Plasmodium protein, unknown function	N/A
Up	PF3D7_1444400	0.64977	6.29E-02	5.49	5.6	5.5	6	6.29	6.05	conserved Plasmodium protein, unknown function	N/A
Up	PF3D7_1213200	0.64847	5.23E-03	6.71	7.4	7	7.74	7.69	7.67	mediator of RNA polymerase II transcription subunit 18, putative	MED18

Up	PF3D7_1459500	0.64745	8.19E-02	5.49	5.8	5.1	6.04	6.11	6.09	conserved Plasmodium protein, unknown function	N/A
Up	PF3D7_1017800	0.64742	9.18E-05	7.65	8	7.7	8.39	8.43	8.45	conserved Plasmodium protein, unknown function	N/A
Up	PF3D7_0716900	0.64687	6.93E-05	8.54	9.1	8.6	9.47	9.38	9.4	drug/metabolite transporter DMT2	DMT2
Up	PF3D7_1469400	0.64228	1.89E-02	6.51	6.9	6.3	7.4	6.91	7.25	nucleoside transporter 3, putative	NT3
Up	PF3D7_1011100	0.6414	4.54E-04	7.24	7.2	7.1	7.85	7.78	7.8	conserved Plasmodium protein, unknown function	N/A
Up	PF3D7_0205400	0.6414	4.86E-05	7.77	8.1	7.9	8.5	8.59	8.61	PCI domain-containing protein, putative	N/A
Up	PF3D7_1200900	0.64089	7.21E-05	7.68	7.7	7.6	8.15	8.39	8.29	Plasmodium exported protein (PHISTc), unknown function	N/A
Up	PF3D7_1029200	0.63933	2.79E-02	6.15	6	5.9	6.85	6.49	6.57	WD repeat-containing protein, putative	N/A
Up	PF3D7_1314600	0.63828	6.02E-03	6.67	6.5	6.5	7.17	7.12	7.22	lipoate-protein ligase 1	LipL1
Up	PF3D7_1236300	0.63526	2.38E-03	6.94	6.9	6.9	7.46	7.66	7.39	conserved protein, unknown function	N/A
Up	PF3D7_1455400	0.63483	2.80E-04	7.61	7.9	7.5	8.29	8.27	8.39	hemolysin III	HlyIII
Up	PF3D7_1025300	0.63344	2.98E-06	8.79	9.2	8.8	9.59	9.54	9.6	conserved protein, unknown function	N/A
Up	PF3D7_1458400	0.62828	5.93E-03	7.04	7.1	6.6	7.69	7.53	7.42	aminodeoxychorismate lyase	ADCL
Up	PF3D7_0506000	0.6265	6.29E-02	5.7	5.6	5.6	6	6.32	6.39	conserved Plasmodium protein, unknown function	N/A
Up	PF3D7_1456200	0.62541	1.05E-02	6.7	6.7	6.3	7.21	7.18	7.16	conserved Plasmodium protein, unknown function	N/A
Up	PF3D7_1250800	0.62211	1.04E-08	10.3	10	11	10.9	11.2	11.1	DNA repair protein rhp16, putative	N/A
Up	PF3D7_1325600	0.62144	3.44E-03	6.7	6.8	6.9	7.41	7.35	7.42	mitochondrial fission 1 protein	FIS1
Up	PF3D7_1453000	0.62059	4.15E-06	8.18	8.3	8.3	8.76	8.92	8.91	conserved Plasmodium protein, unknown function	N/A
Up	PF3D7_1433700	0.62002	2.10E-08	8.9	8.8	8.9	9.45	9.46	9.55	conserved Plasmodium protein, unknown function	N/A
Up	PF3D7_0904500	0.61979	3.75E-04	7.38	7.5	7.3	7.99	7.98	8	prefoldin subunit 4, putative	N/A
Up	PF3D7_0721900	0.61905	4.98E-03	6.73	6.8	7	7.56	7.28	7.44	V-type ATPase V0 subunit e, putative	N/A
Up	PF3D7_1436400	0.61868	3.36E-02	6.15	6.4	5.8	6.67	6.86	6.7	conserved Plasmodium protein, unknown function	N/A
Up	PF3D7_1147500	0.61636	1.76E-02	6.41	6.5	6.1	7.03	6.97	6.82	protein farnesyltransferase subunit beta	FTB

Up	PF3D7_1327700	0.61617	5.26E-03	6.92	7.1	6.6	7.49	7.49	7.49	regulator of nonsense transcripts 3B, putative	UPF3B
Up	PF3D7_1474700	0.61602	3.93E-02	5.83	6.2	5.8	6.57	6.48	6.54	protein kinase, putative	N/A
Up	PF3D7_0920000	0.61576	1.39E-02	6.41	6.6	6.4	7.13	6.88	7.16	elongation of fatty acids protein, putative	ELO3
Up	PF3D7_0614200	0.61442	5.04E-04	7.35	7.2	7.3	7.98	7.86	7.91	cytosolic Fe-S cluster assembly factor NAR1, putative	NAR1
Up	PF3D7_1363600	0.60935	6.25E-02	5.46	6.1	6	6.39	6.51	6.39	conserved Plasmodium protein, unknown function	N/A
Up	PF3D7_1014500	0.60828	3.44E-02	6.15	6.2	5.9	6.51	6.74	6.72	conserved Plasmodium protein, unknown function	N/A
Up	PF3D7_1468600	0.60821	5.80E-02	5.73	5.9	5.6	6.23	6.38	6.34	aminophospholipid transporter, putative	N/A
Up	PF3D7_1144300	0.60538	8.88E-11	10.4	11	11	11	11.2	11.1	60S ribosomal protein L41	RPL41
Up	PF3D7_1416300	0.60353	9.78E-05	7.78	8.1	7.9	8.52	8.58	8.51	N-acyl-phosphatidylethanolamine-hydrolyzing phospholipase D, putative	N/A
Up	PF3D7_1366700	0.60161	7.02E-02	5.78	5.9	5.6	6.17	6.54	6.25	conserved Plasmodium protein, unknown function	N/A
Up	PF3D7_0808000	0.6016	6.74E-03	7.13	7	6.7	7.64	7.41	7.58	conserved Plasmodium protein, unknown function	N/A
Up	PF3D7_1328400	0.59979	1.86E-03	7.25	7.4	7	7.85	7.85	7.8	conserved protein, unknown function	N/A
Up	PF3D7_0603100	0.59938	7.44E-05	7.85	7.9	7.9	8.41	8.62	8.44	RNA-binding protein, putative	N/A
Up	PF3D7_0809200	0.59811	3.83E-07	8.72	8.7	8.6	9.22	9.25	9.31	asparagine-rich antigen Pfa55-14	pfa55-14
Up	PF3D7_1127400	0.59582	2.20E-02	6.21	6.6	6.3	6.86	7.06	6.96	conserved Plasmodium protein, unknown function	N/A
Up	PF3D7_1313400	0.59558	3.07E-06	8.35	8.5	8.4	9.04	9.02	9.01	DEAD box helicase, putative	N/A
Up	PF3D7_1371700	0.59469	1.18E-02	6.9	7	6.5	7.26	7.46	7.4	serine/threonine protein kinase, FIKK family	FIKK13
Up	PF3D7_1414700	0.5928	2.63E-03	7.18	7.6	7.2	7.87	8.02	7.89	ubiquitin carboxyl-terminal hydrolase, putative	N/A
Up	PF3D7_0828100	0.59093	1.49E-03	7.25	7.5	7.2	7.92	7.85	7.9	conserved Plasmodium protein, unknown function	N/A
Up	PF3D7_1443200	0.58991	2.99E-02	6.49	6.3	6.1	7.1	6.73	6.85	heptatricopeptide repeat-containing protein, putative	N/A
Up	PF3D7_1015000	0.58836	7.02E-02	6.02	6.2	5.8	6.85	6.23	6.56	FAD synthetase, putative	N/A

Up	PF3D7_0519900	0.58743	3.17E-04	7.78	7.5	7.7	8.28	8.17	8.27	conserved protein, unknown function	N/A
Up	PF3D7_1404600	0.58587	6.95E-02	5.67	5.7	6.2	6.48	6.36	6.44	adenylyl cyclase alpha	ACalpha
Down	PF3D7_1149800	-5.24915	3.85E-03	3.32	3.5	3.6	2	2	2	rifin	RIF
Down	PF3D7_1479900	-5.06509	1.92E-03	4.17	4.1	3.7	2	2.32	2	stevor	N/A
Down	PF3D7_1477100	-4.28467	3.43E-03	4.17	4.3	3.7	2.32	2	2.32	Plasmodium exported protein (hyp6), unknown function	N/A
Down	PF3D7_1478400	-4.22166	3.16E-04	4.64	4.6	4.2	2.58	2	2.32	Plasmodium exported protein, unknown function, pseudogene	N/A
Down	PF3D7_1477200	-4.02909	1.30E-03	4.91	4.2	3.6	2.58	2	2.32	Plasmodium exported protein (hyp15), unknown function	N/A
Down	PF3D7_0711700	-3.32118	1.12E-03	4.32	4.6	4.1	2.58	2.32	2.59	erythrocyte membrane protein 1, PfEMP1	VAR
Down	PF3D7_0617600	-3.27932	1.45E-04	5.09	4.6	4.4	2.81	2.32	2.81	stevor	N/A
Down	PF3D7_0712800	-3.16931	7.30E-02	3.46	3.5	3	2.32	2.32	2	erythrocyte membrane protein 1, PfEMP1	VAR
Down	PF3D7_0114300	-3.14612	1.25E-24	7.4	7.7	7.1	4.7	4.39	4.39	exported protein family 4, pseudogene	EPF4
Down	PF3D7_0100400	-3.12833	9.41E-03	4.09	3.6	4.2	2.32	2.59	2.32	rifin	RIF
Down	PF3D7_0617500	-3.0697	4.98E-03	3.91	4.6	3.8	2.58	2.32	2.59	rifin, pseudogene	RIF
Down	PF3D7_0532800	-3.02482	1.32E-06	5.46	5.7	4.9	3.17	3	3.17	stevor, pseudogene	N/A
Down	PF3D7_1255200	-2.90618	2.02E-02	3.91	3.9	3.6	2.58	2.32	2.32	erythrocyte membrane protein 1, PfEMP1	VAR
Down	PF3D7_0302500	-2.8634	2.98E-15	11.5	11	11	8.03	8.38	8.62	cytoadherence linked asexual protein 3.1	CLAG3.1
Down	PF3D7_1477400	-2.82104	3.10E-18	7.1	7.4	6.8	4.64	4.7	4.17	Plasmodium exported protein (PHIST), unknown function	N/A
Down	PF3D7_1000800	-2.80651	1.48E-02	4	4.1	3.8	2.58	2	2.81	stevor, pseudogene	N/A
Down	PF3D7_1479500	-2.69975	4.10E-02	3.81	3.9	3.2	2.32	2.59	2.32	stevor	N/A
Down	PF3D7_0114200	-2.6738	9.67E-05	5.13	5.1	4.7	2.81	3.32	3	exported protein family 3	EPF3
Down	PF3D7_0532900	-2.61384	5.43E-03	3.81	4.5	4.6	3	2.32	2.81	rifin	RIF
Down	PF3D7_0533000	-2.61128	1.63E-10	6.25	6.1	6.3	3.59	4.25	4	rifin, pseudogene	RIF
Down	PF3D7_0937200	-2.52238	7.80E-02	3	3.5	4	2	2.59	2.59	lysophospholipase, putative	N/A
Down	PF3D7_1219400	-2.4585	3.71E-02	3.59	3.6	4.4	3	2	2.59	erythrocyte membrane protein 1 (PfEMP1), pseudogene	VAR
Down	PF3D7_0929500	-2.43178	6.49E-02	3.46	3.2	4.2	2.58	2	2.81	CH domain-containing protein, putative	N/A

Down	PF3D7_1372500	-2.3869	2.48E-07	5.59	6	5.9	3.7	4	3.7	stevor, pseudogene	N/A
Down	PF3D7_1149900	-2.29042	2.12E-04	4.86	5.5	5	3.17	3.32	3.59	stevor	N/A
Down	PF3D7_1421150	-2.2719	6.73E-02	4	3.2	4	2.58	2	3	C/D small nucleolar RNA	N/A
Down	PF3D7_0632700	-2.16923	3.64E-02	4.46	3.8	3.6	3	2.59	2.59	rifin	RIF
Down	PF3D7_0713100	-2.15964	2.05E-03	4.64	5.3	4.5	2.81	3.32	3.59	Pfmc-2TM Maurer's cleft two transmembrane protein	MC-2TM
Down	PF3D7_1300200	-2.04988	6.57E-02	3.91	3.8	3.6	2.81	2.59	2.59	rifin	RIF
Down	PF3D7_0223400	-2.04972	7.10E-02	3.46	3.8	4	2.81	2.32	2.81	rifin	RIF
Down	PF3D7_0732900	-1.95835	6.14E-02	4.32	3.5	4	2.81	2.32	3.17	rifin	RIF
Down	PF3D7_1240400	-1.92698	1.01E-05	5.55	5.9	5.9	4	4.32	4.09	erythrocyte membrane protein 1, PfEMP1	VAR
Down	PF3D7_0701600	-1.92525	1.46E-06	6.11	6.2	6.2	4.64	4.86	3.7	Pfmc-2TM Maurer's cleft two transmembrane protein	MC-2TM
Down	PF3D7_1040200	-1.91679	8.38E-03	4.39	5.2	4.4	3.32	3	3.59	stevor	N/A
Down	PF3D7_1240600	-1.85712	2.77E-08	6.63	6.2	6.6	4.86	4.96	4.59	erythrocyte membrane protein 1, PfEMP1	VAR
Down	PF3D7_1372600	-1.74942	7.77E-02	3.7	4.3	4	3.32	3	2.32	rifin	RIF
Down	PF3D7_0302300	-1.71566	5.13E-02	4.32	4.6	3.7	3.32	3.32	2.59	erythrocyte membrane protein 1 (PfEMP1), pseudogene	N/A
Down	PF3D7_1477300	-1.66838	4.94E-06	5.93	6.3	6.6	4.95	4.59	4.86	Plasmodium exported protein (PHIST), unknown function	Pfg14-744
Down	PF3D7_1400100	-1.60178	1.49E-02	5.43	4.6	4.7	3.81	3	4.09	erythrocyte membrane protein 1 (PfEMP1), pseudogene	N/A
Down	PF3D7_1477700	-1.58404	7.81E-05	5.98	5.6	6.3	4.64	4.64	4.46	Plasmodium exported protein (PHISTa), unknown function	Pfg14-748
Down	PF3D7_0702100	-1.53963	4.15E-02	5.21	4.3	4.2	3.32	3	4	Plasmodium exported protein (PHISTb), unknown function, pseudogene	N/A
Down	PF3D7_1000100	-1.48191	3.42E-03	5.04	5.5	5.4	3.91	4.17	4.17	erythrocyte membrane protein 1, PfEMP1	VAR
Down	PF3D7_0701900	-1.47544	2.57E-02	6.36	4.6	5.1	4.17	3.46	4.86	Plasmodium exported protein, unknown function	N/A
Down	PF3D7_0929600	-1.47002	8.30E-02	4.81	3.5	5.1	3.17	3	4.09	G2 protein, putative	N/A
Down	PF3D7_0935900	-1.42919	1.75E-02	8.6	7.7	7.1	6.64	6.13	6.74	ring-exported protein 1	REX1
Down	PF3D7_0700800	-1.39168	5.64E-02	4.25	5.2	4.2	3.81	3.32	3.59	Pfmc-2TM Maurer's cleft two transmembrane protein	MC-2TM

Down	PF3D7_1253000	-1.34212	1.24E-31	9.82	9.7	9.9	8.54	8.57	8.33	gametocyte erythrocyte cytosolic protein	GECO
Down	PF3D7_0830500	-1.33668	1.68E-03	7.53	6.5	6	5.46	5.21	5.91	sporozoite and liver stage tryptophan-rich protein, putative	TryThrA
Down	PF3D7_0206800	-1.33551	1.30E-05	11.2	11	11	9.57	9.75	9.99	merozoite surface protein 2	MSP2
Down	PF3D7_1247800	-1.31452	9.44E-03	5.25	5.1	5.7	4.25	4	4.46	dipeptidyl aminopeptidase 2	DPAP2
Down	PF3D7_0712000	-1.29978	1.51E-02	5.13	5	5.5	4.46	3.81	4.09	erythrocyte membrane protein 1, PfEMP1	VAR
Down	PF3D7_1100800	-1.28867	2.06E-02	5	5.2	4.9	3.81	4	4.17	Pfmc-2TM Maurer's cleft two transmembrane protein	MC-2TM
Down	PF3D7_1200600	-1.26685	1.37E-02	5.95	5.7	4.8	4.75	4.25	4.32	erythrocyte membrane protein 1, PfEMP1	VAR2CSA
Down	PF3D7_0108500	-1.23626	4.19E-06	8.2	7.6	7.3	6.63	6.3	6.65	ELM2 domain-containing protein, putative	N/A
Down	PF3D7_0936300	-1.16417	3.67E-02	6.43	5.2	5.2	4.7	4	5.17	ring-exported protein 3	REX3
Down	PF3D7_0114600	-1.16108	1.36E-02	5.36	6	5.3	4.75	4.64	4.25	stevor, pseudogene	N/A
Down	PF3D7_1240300	-1.1356	1.06E-09	7.91	8.2	8.4	6.98	7.11	7.14	erythrocyte membrane protein 1, PfEMP1	VAR
Down	PF3D7_0620400	-1.11829	2.10E-02	5.73	5.1	5.6	4.09	4.59	4.86	merozoite surface protein 10	MSP10
Down	PF3D7_0114000	-1.09436	1.44E-02	5.52	6	5.5	4.75	4.32	4.96	exported protein family 1	EPF1
Down	PF3D7_0707300	-1.04758	1.10E-02	12	11	12	10.4	10.8	11	rhoptry-associated membrane antigen	RAMA
Down	PF3D7_1102700	-1.0406	2.73E-03	7.43	6.8	6.3	6.13	5.56	6.07	early transcribed membrane protein 11.1	ETRAMP1 1.1
Down	PF3D7_0501300	-1.03948	2.05E-05	8.27	7.8	7.4	6.98	6.74	6.85	skeleton-binding protein 1	SBP1
Down	PF3D7_0114100	-1.03879	2.89E-03	6.04	6.5	6.3	5.61	5.21	5.09	Pfmc-2TM Maurer's cleft two transmembrane protein	MC-2TM
Down	PF3D7_0324800	-1.03723	1.09E-02	5.95	5.6	6.5	5.09	4.86	5.39	rifin	RIF
Down	PF3D7_0831800	-1.02786	6.19E-02	6.51	5.6	4.7	4.91	4.7	4.96	histidine-rich protein II	HRP2
Down	PF3D7_0823500	-1.01741	8.69E-02	4.86	4.9	5	3.7	4	4.46	inner membrane complex protein 1i, putative	IMC1i
Down	PF3D7_1012200	-1.00636	2.23E-04	7.91	7.1	7.8	6.32	6.72	6.9	rhoptry associated adhesin	RA
Down	PF3D7_0214900	-0.95909	3.18E-02	9.49	8.6	9.6	7.92	8.56	8.52	rhoptry neck protein 6	RON6
Down	PF3D7_0722200	-0.93183	3.40E-02	10.1	9.2	10	8.48	9.06	9.17	rhoptry-associated leucine zipper-like protein 1	RALP1
Down	PF3D7_1335300	-0.92924	8.41E-03	8.02	7.3	6.6	6.52	6.17	6.81	reticulocyte binding protein 2 homologue b	RH2b

Down	PF3D7_0730800	-0.88075	5.08E-03	7.27	6.8	6.5	5.95	5.83	6.27	Plasmodium exported protein, unknown function	N/A
Down	MAL_RNA_17	-0.87743	4.48E-14	11.1	11	11	10.1	10.4	10.4		
Down	PF3D7_1035900	-0.87458	8.10E-02	8.77	7.7	8.5	7.15	7.3	7.98	merozoites-associated armadillo repeats protein	MAAP
Down	PF3D7_1035400	-0.85676	1.48E-07	10.4	9.7	10	9.17	9.27	9.46	merozoite surface protein 3	MSP3
Down	PF3D7_1027300	-0.85673	1.06E-11	12.9	13	13	11.9	12.2	12.1	peroxiredoxin	nPrx
Down	PF3D7_0423300	-0.84541	6.97E-03	7.18	6.4	7.2	6.07	5.98	6.41	conserved Plasmodium protein, unknown function	N/A
Down	PF3D7_1341800	-0.84537	4.39E-02	6.23	5.7	5.5	5	5	5.21	inner membrane complex protein 1k, putative	IMC1k
Down	PF3D7_1136200	-0.83404	5.21E-03	7.93	7	8.3	6.92	6.99	7.12	conserved Plasmodium protein, unknown function	N/A
Down	PF3D7_1141100	-0.82678	7.27E-02	5.25	5.8	5.5	5	4.76	4.59	conserved Plasmodium protein, unknown function	N/A
Down	PF3D7_1035700	-0.81861	1.96E-02	10.3	9.5	10	9.02	9.37	9.45	duffy binding-like merozoite surface protein	DBLMSP
Down	PF3D7_0501400	-0.81734	6.02E-08	9.62	9.1	9.2	8.53	8.45	8.62	interspersed repeat antigen	FIRA
Down	PF3D7_0905400	-0.8168	1.91E-02	13	12	13	11.8	12.2	12.2	high molecular weight rhoptry protein 3	RhopH3
Down	PF3D7_1452000	-0.81184	2.86E-02	9.56	8.7	9.5	8.18	8.59	8.7	rhoptry neck protein 2	RON2
Down	PF3D7_0405900	-0.80125	2.08E-04	9.17	8.4	9	7.85	8.06	8.35	apical sushi protein	ASP
Down	PF3D7_0207000	-0.79588	5.54E-12	11.3	11	11	10.4	10.5	10.5	merozoite surface protein 4	MSP4
Down	PF3D7_1035200	-0.78758	6.40E-02	13.1	12	13	11.9	12.3	12.4	S-antigen	N/A
Down	PF3D7_1228600	-0.78675	2.52E-02	13.3	13	14	12.3	12.7	12.7	merozoite surface protein 9	MSP9
Down	PF3D7_1133400	-0.78428	4.68E-05	9.63	8.9	9.3	8.39	8.41	8.77	apical membrane antigen 1	AMA1
Down	PF3D7_1449600	-0.78366	4.33E-02	6.36	5.9	6.3	4.86	5.67	5.67	conserved protein, unknown function	N/A
Down	PF3D7_1211800	-0.78334	1.23E-14	11.4	11	12	10.6	10.7	10.7	polyubiquitin	PfpUB
Down	PF3D7_1035100	-0.78317	4.06E-04	8.73	8.2	9.2	7.89	7.99	8.05	probable protein, unknown function	N/A
Down	PF3D7_1036000	-0.77366	2.11E-02	7.37	6.5	6.6	5.91	6.13	6.31	merozoite surface protein 11	MSP11
Down	PF3D7_1013800	-0.77266	7.01E-05	7.79	7.6	7.6	6.94	6.87	6.94	conserved Plasmodium protein, unknown function	N/A
Down	PF3D7_0817600	-0.77255	5.41E-04	9.41	8.6	9	8.19	7.97	8.58	conserved protein, unknown function	N/A

Down	PF3D7_0723300	-0.76508	4.65E-06	8.91	8.6	9.2	8.04	8.22	8.21	conserved protein, unknown function	N/A
Down	PF3D7_0508000	-0.75775	8.86E-05	9.41	9	9.7	8.38	8.87	8.71	6-cysteine protein	P38
Down	PF3D7_1001600	-0.75439	2.22E-08	12.3	12	13	11.3	11.7	11.5	exported lipase 2	XL2
Down	PF3D7_0817700	-0.75283	5.13E-02	9.65	8.8	9.7	8.4	8.81	8.81	rhoptry neck protein 5	RON5
Down	PF3D7_0929400	-0.75255	8.20E-03	12.7	12	13	11.7	11.9	11.9	high molecular weight rhoptry protein 2	RhopH2
Down	PF3D7_0533100	-0.73794	1.06E-04	8.55	8.5	9.1	7.96	8.14	7.87	erythrocyte membrane protein 1 (PfEMP1), pseudogene	VAR1CSA
Down	PF3D7_1105000	-0.73264	5.49E-08	11.9	12	12	11.6	11.3	11.4	histone H4	H4
Down	PF3D7_1140400	-0.72453	3.44E-03	7.61	7.1	7.8	6.69	6.83	6.96	conserved Plasmodium protein, unknown function	N/A
Down	PF3D7_1016300	-0.72316	1.94E-21	15.6	16	16	15	14.9	14.9	GBP130 protein	GBP130
Down	PF3D7_0506900	-0.7171	8.13E-05	10.3	9.7	11	9.33	9.65	9.56	rhomboid protease ROM4	ROM4
Down	PF3D7_1361800	-0.71148	1.71E-02	11.7	11	12	10.7	10.9	10.9	glideosome-associated connector	GAC
Down	PF3D7_1356300	-0.70819	5.32E-09	9.22	9.1	9.2	8.51	8.41	8.46	ubiquitin-conjugating enzyme E2, putative	N/A
Down	PF3D7_1401600	-0.70793	1.57E-02	8.36	7.2	8.3	7.16	7.3	7.58	Plasmodium exported protein (PHISTb), unknown function	N/A
Down	PF3D7_0503000	-0.70483	7.69E-10	9.52	9.5	9.7	8.91	8.86	8.9	50S ribosomal protein L28, apicoplast, putative	N/A
Down	PF3D7_1125800	-0.70461	8.60E-02	7.63	6.7	6.2	6.29	5.86	6.62	kelch domain-containing protein, putative	N/A
Down	PF3D7_0617400	-0.69897	1.29E-02	6.88	6.6	6.7	6	5.93	6.25	erythrocyte membrane protein 1, PfEMP1	VAR
Down	PF3D7_1141800	-0.69352	9.04E-04	8.24	8	8.7	7.66	7.59	7.76	EELM2 domain-containing protein, putative	N/A
Down	PF3D7_1439400	-0.6929	2.28E-09	9.97	9.8	10	9.26	9.32	9.32	cytochrome b-c1 complex subunit Rieske, putative	N/A
Down	PF3D7_0113000	-0.69265	6.99E-09	12.1	12	12	11.4	11.3	11.3	glutamic acid-rich protein GARP	GARP
Down	PF3D7_0107000	-0.68991	2.74E-10	10.6	11	11	9.94	10.1	9.92	centrin-1	CEN1
Down	PF3D7_0604100	-0.68861	3.76E-02	11.2	11	12	10.3	10.6	10.6	AP2 domain transcription factor	SIP2
Down	PF3D7_1116000	-0.68555	9.24E-05	10.1	9.9	11	9.32	9.65	9.69	rhoptry neck protein 4	RON4
Down	PF3D7_1145400	-0.68105	4.82E-08	11.3	11	12	10.5	10.6	10.6	dynamin-like protein	DYN1
Down	PF3D7_1252100	-0.67832	8.12E-02	10.3	9.6	11	9.19	9.55	9.69	rhoptry neck protein 3	RON3

Down	PF3D7_1202900	-0.67717	1.51E-16	11.9	12	12	11.2	11.3	11.2	high mobility group protein B1	HMGB1
Down	PF3D7_1474000	-0.67708	7.44E-02	6	5.8	6.4	5.25	5.52	5.59	conserved Plasmodium protein, unknown function	N/A
Down	PF3D7_0520900	-0.67263	2.43E-17	13.4	13	14	12.7	12.8	12.8	adenosylhomocysteinase	SAHH
Down	PF3D7_0617800	-0.66927	1.77E-26	15.7	16	16	15	15	15	histone H2A	H2A
Down	PF3D7_1035600	-0.66926	3.87E-02	6.91	6.3	7.2	5.95	6.46	6.13	merozoite surface protein	H101
Down	PF3D7_0922200	-0.66798	7.78E-19	14.2	14	14	13.5	13.6	13.7	S-adenosylmethionine synthetase	SAMS
Down	PF3D7_0731100	-0.6648	8.41E-08	9.5	9.3	9.3	8.7	8.64	8.78	EMP1-trafficking protein	PTP2
Down	PF3D7_1301700	-0.66417	6.91E-02	6.04	5.9	6	5.36	5.39	5.32	CX3CL1-binding protein 2	CBP2
Down	PF3D7_0202400	-0.66039	3.00E-15	11.3	11	11	10.7	10.7	10.6	translation-enhancing factor	PTEF
Down	PF3D7_1222700	-0.65726	4.05E-05	9.18	8.8	9.2	8.36	8.27	8.64	glideosome-associated protein 45	GAP45
Down	PF3D7_0528400	-0.65644	1.92E-02	7.2	6.9	7.7	6.38	6.72	6.85	palmitoyltransferase DHHC7	DHHC7
Down	PF3D7_0207600	-0.65548	3.10E-02	15	15	15	14.3	14.5	14.4	serine repeat antigen 5	SERA5
Down	PF3D7_1235200	-0.65474	1.22E-02	8.21	7.6	8.7	7.34	7.76	7.62	V-type K+-independent H+-translocating inorganic pyrophosphatase	VP2
Down	PF3D7_1216500	-0.65384	2.06E-02	6.77	6.5	6.9	5.93	6.19	6.19	male development gene 1	MDV1
Down	PF3D7_0315600	-0.65275	3.24E-02	6.32	6.5	6.8	5.95	6.05	5.78	zinc finger protein, putative	N/A
Down	PF3D7_1437300	-0.64988	3.50E-02	7	6.3	7.1	6.15	6.29	6.27	conserved Plasmodium protein, unknown function	N/A
Down	PF3D7_1423300	-0.64383	3.44E-02	7.75	7	7.3	6.55	6.41	7.13	serine/threonine protein phosphatase 7	PP7
Down	PF3D7_0800100	-0.64293	9.47E-03	7.31	7	7.6	6.83	6.56	6.76	erythrocyte membrane protein 1, PfEMP1	VAR
Down	PF3D7_1353000	-0.63515	2.93E-12	12.2	12	12	11.5	11.6	11.6	tryptophan-rich protein, pseudogene	LysTrpA
Down	PF3D7_0207700	-0.63395	2.75E-02	11.6	11	12	10.8	11.1	11.1	serine repeat antigen 4	SERA4
Down	PF3D7_0500800	-0.63181	2.40E-19	15.8	16	16	15.1	15.3	15.2	mature parasite-infected erythrocyte surface antigen	MESA
Down	PF3D7_1434200	-0.62861	4.98E-14	11.5	11	12	10.9	10.9	10.8	calmodulin	CAM
Down	PF3D7_1141200	-0.62617	1.09E-02	7.15	7	7.1	6.54	6.27	6.66	ATP synthase-associated protein, putative	N/A
Down	PF3D7_1413200	-0.61469	2.53E-02	6.69	7	6.8	6.3	6.05	6.41	conserved Plasmodium protein, unknown function	N/A

Down	PF3D7_0917900	-0.6084	4.83E-11	15.1	15	15	14.5	14.6	14.5	heat shock protein 70	BIP
Down	PF3D7_1107100	-0.60808	1.64E-04	9.42	9.4	9.9	8.9	9	9.04	nucleic acid binding protein, putative	N/A
Down	PF3D7_0220000	-0.60591	1.23E-14	13.3	13	13	12.6	12.8	12.8	liver stage antigen 3	LSA3
Down	PF3D7_0515700	-0.59962	2.41E-06	11.3	11	11	10.5	10.6	10.7	glideosome-associated protein 40, putative	GAP40
Down	PF3D7_1426900	-0.59823	6.02E-05	8.71	8.6	8.9	8.17	8.2	8.11	cytochrome b-c1 complex subunit 6, putative	QCR6
Down	PF3D7_1248900	-0.59315	1.06E-10	11.2	11	11	10.6	10.6	10.6	26S protease regulatory subunit 8, putative	RPT6
Down	PF3D7_1033200	-0.59223	9.30E-06	10.1	9.9	10	9.61	9.56	9.57	early transcribed membrane protein 10.2	ETRAMP10.2
Down	PF3D7_1115800	-0.59106	8.39E-03	7.64	7.3	7.5	6.75	6.88	7.01	conserved Plasmodium protein, unknown function	N/A
Down	PF3D7_1244700	-0.59052	7.92E-04	8.18	8	8	7.42	7.62	7.4	conserved protein, unknown function	N/A
Down	PF3D7_1467600	-0.58938	3.46E-04	8.49	8.3	8.7	8.04	7.86	7.91	conserved Plasmodium protein, unknown function	N/A
Down	PF3D7_1302100	-0.58721	7.60E-05	10.1	9.9	10	9.78	9.5	9.47	gamete antigen 27/25	G27/25

Table S3C. Differential expression between PfDNMT2 disruptant and wildtype parasite at the schizont stage

R: replicate, WT: 3D7 wild type, KO: PfDNMT2 disruptant, Adj.Pval: adjusted P value

columns G-L is EdgeR log2 (counts per million + count)

Change pattern	Gene ID	log2 Fold Change	Adj.Pval	WT R1	WT R2	WT R3	KO R1	KO R2	KO R3	Product Description	Name or Symbol
Up	PF3D7_0425500	1.498405	2.08E-13	6.5	6.8	6.2	7.9	8	8	stevor	UBP1
Up	PF3D7_0302200	1.306673	4.10E-25	12	12	12	14	14	14	cytoadherence linked asexual protein 3.2	N/A
Up	PF3D7_0900100	0.888597	8.69E-05	7.2	7.2	6.8	8.2	8	7.9	erythrocyte membrane protein 1, PfEMP1	N/A
Up	PF3D7_1370800	0.841678	6.75E-04	6.4	6.6	6.6	7.2	7	7.4	non-coding RNA	PTP1
Up	PF3D7_0223500	0.830805	1.23E-02	5.9	5.8	5.9	6.9	6	6.6	erythrocyte membrane protein 1, PfEMP1	GEXP20
Up	PF3D7_1200400	0.814977	3.56E-02	6.2	5.9	5.2	6.3	7	6.5	erythrocyte membrane protein 1, PfEMP1	N/A
Up	PF3D7_1478900	0.799772	5.09E-08	13	13	12	14	13	13	non-coding RNA	N/A
Up	PF3D7_0805000	0.783201	9.35E-02	5.8	5.8	5.4	7	6	6	alpha/beta hydrolase, putative	VAR
Up	PF3D7_1200200	0.704988	4.68E-02	6.1	6.3	5.6	6.7	6	6.9	rifin	FIKK3
Up	PF3D7_1370900	0.693859	1.82E-03	6.9	7.1	6.9	7.6	8	7.7	non-coding RNA	GEXP21
Up	PF3D7_1356900	0.666069	4.21E-02	6.3	6.3	5.9	7	7	6.6	protein kinase 5	N/A
Up	PF3D7_1148200	0.625589	3.74E-03	7.5	7.7	7.3	8.1	8	8.3	non-coding RNA	CLAG3.2
Up	PF3D7_1241700	0.593722	4.18E-02	6.8	7	6.5	7.6	7	7.3	replication factor C subunit 4, putative	CLAG3.1
Down	PF3D7_0425100	-2.56271	2.03E-16	7.1	6.9	7.6	4.5	5	5.3	Plasmodium exported protein (hyp6), unknown function	N/A
Down	PF3D7_0425200	-2.48126	4.62E-25	7.7	7.5	8	5.2	5	5.5	Plasmodium exported protein (hyp15), unknown function	N/A
Down	PF3D7_1240600	-2.12169	4.30E-14	7.2	6.8	7.4	4.9	5	5.3	erythrocyte membrane protein 1, PfEMP1	N/A
Down	PF3D7_1253000	-1.92533	2.70E-20	8.4	8.2	8.9	6.7	7	6.6	gametocyte erythrocyte cytosolic protein	SMN
Down	PF3D7_0302500	-1.88413	3.53E-53	15	15	15	13	14	13	cytoadherence linked asexual protein 3.1	ACS6
Down	PF3D7_1240400	-1.67773	3.48E-12	7.3	7.3	7.4	5.4	6	5.9	erythrocyte membrane protein 1, PfEMP1	N/A
Down	PF3D7_1370300	-1.22798	1.08E-03	7.5	6.5	6.8	5.4	5	6.4	membrane associated histidine-rich protein 1	RH1

Down	PF3D7_1252900	-1.21647	1.57E-03	6.6	6	7.3	5.5	5	5.6	Plasmodium exported protein, unknown function	RH4
Down	PF3D7_0831800	-1.18008	1.60E-08	9.5	8.9	9.4	7.9	8	8	histidine-rich protein II	FIKK4.1
Down	PF3D7_1200600	-1.1594	4.40E-06	7.8	7.7	7.5	6	7	6.7	erythrocyte membrane protein 1, PfEMP1	FIKK4.2
Down	PF3D7_0936300	-1.14809	9.49E-02	8.8	8	9	6.8	8	7.8	ring-exported protein 3	N/A
Down	PF3D7_1148900	-1.11813	5.00E-03	6.4	5.7	6.6	5.4	5	5.2	Plasmodium exported protein, unknown function	N/A
Down	PF3D7_1253300	-1.09251	2.18E-02	6	5.3	6.7	4.9	5	5	Plasmodium exported protein (PHISTa), unknown function, pseudogene	N/A
Down	PF3D7_0830600	-1.05774	8.69E-05	8.7	8.1	9	7.2	8	7.8	Plasmodium exported protein (PHISTc), unknown function	N/A
Down	PF3D7_0702300	-1.0441	5.49E-04	8.2	7.6	8.5	6.6	7	7.5	sporozoite threonine and asparagine-rich protein	HSP40
Down	PF3D7_1002000	-1.01312	5.81E-03	7.1	6.3	7	5.3	6	6	Plasmodium exported protein (hyp2), unknown function	SBP1
Down	PF3D7_1301700	-1.0104	7.55E-03	7	6.1	6.7	5.6	5	6	CX3CL1-binding protein 2	FIRA
Down	PF3D7_1353100	-1.00039	3.50E-03	7.4	6.5	7.6	6	6	6.5	Plasmodium exported protein, unknown function	MSP8
Down	PF3D7_1352900	-0.99413	3.85E-05	9.6	9.1	9.9	8.2	9	8.7	Plasmodium exported protein, unknown function	SOPT
Down	PF3D7_1478000	-0.98918	6.57E-02	9.6	8.9	9.7	7.8	9	8.7	Plasmodium exported protein (PHISTa), unknown function	N/A
Down	PF3D7_1001400	-0.97403	1.57E-04	7.9	7.3	7.8	6.6	7	7	exported lipase 1	N/A
Down	PF3D7_0220700	-0.94189	3.92E-04	7.7	7.2	7.9	6.7	7	6.7	Plasmodium exported protein (hyp9), unknown function	TKL4
Down	PF3D7_0202200	-0.93287	8.69E-05	9.1	8.4	9.1	7.7	8	8.2	EMP1-trafficking protein	N/A
Down	PF3D7_1315000	-0.93102	2.24E-03	6.7	6.6	6.9	5.6	6	5.9	conserved protein, unknown function	N/A
Down	PF3D7_0113300	-0.92774	6.87E-03	6.7	6.3	7.1	5.6	6	6	Plasmodium exported protein (hyp1), unknown function	N/A

Down	PF3D7_1001900	-0.92746	5.06E-04	7.7	7.4	8.1	6.6	7	7	Plasmodium exported protein (hyp16), unknown function	N/A
Down	PF3D7_0926200	-0.92504	6.87E-03	7.7	7.3	8	6	7	6.8	conserved Plasmodium protein, unknown function	N/A
Down	PF3D7_0726100	-0.9227	6.14E-04	8.4	7.9	8.7	7.1	8	7.5	Plasmodium exported protein, unknown function	LPL20
Down	PF3D7_1302000	-0.91341	1.42E-03	7.3	7	7.8	6.5	7	6.5	EMP1-trafficking protein	STARP
Down	PF3D7_1149300	-0.90488	2.80E-03	7.5	7.2	7.8	6.1	7	6.8	serine/threonine protein kinase, FIKK family	N/A
Down	PF3D7_0525300	-0.89462	4.16E-04	7.8	7.5	8.1	7	7	6.7	conserved protein, unknown function	SDA1
Down	PF3D7_0707500	-0.89461	8.69E-05	10	10	11	9.4	9	9.7	conserved Plasmodium protein, unknown function	N/A
Down	PF3D7_1353200	-0.88259	5.00E-03	7.5	6.7	7.4	6.1	6	6.6	membrane associated histidine-rich protein 2	N/A
Down	PF3D7_0219700	-0.88176	7.44E-03	7.6	7	8.3	6.7	7	7	Plasmodium exported protein (PHISTc), unknown function	NMD3
Down	PF3D7_0424500	-0.87709	1.58E-04	9.8	9.2	10	8.7	9	9.1	serine/threonine protein kinase, FIKK family	N/A
Down	PF3D7_0702000	-0.87535	9.76E-04	9.2	8.8	9.6	7.9	8	8.7	Plasmodium exported protein (hyp12), unknown function	PTP4
Down	PF3D7_1404800	-0.87506	4.15E-06	12	11	12	10	10	11	conserved Plasmodium protein, unknown function	N/A
Down	PF3D7_1401100	-0.86509	7.42E-05	11	11	11	9.9	10	10	DnaJ protein, putative	PTP2
Down	PF3D7_0800800	-0.8457	2.48E-02	6.1	6.3	6.5	5.7	6	5.2	Plasmodium exported protein (hyp7), unknown function	N/A
Down	PF3D7_0201700	-0.82907	6.41E-04	8.6	8.1	8.9	7.6	8	8	DnaJ protein, putative	FIKK7.2
Down	PF3D7_0902500	-0.82785	5.06E-04	9.7	9.2	9.9	8.5	9	9.2	serine/threonine protein kinase, FIKK family	N/A
Down	PF3D7_1200800	-0.82399	3.55E-03	7.1	6.8	7.3	6.2	6	6.3	serine/threonine protein kinase, FIKK family	N/A

Down	PF3D7_0502400	-0.81311	1.82E-03	8.6	8.2	9	7.5	8	8.1	merozoite surface protein 8	N/A
Down	PF3D7_1413800	-0.81276	5.38E-05	8.8	8.5	8.9	7.8	8	7.9	diphthamide biosynthesis protein 1, putative	N/A
Down	PF3D7_0902300	-0.81176	1.70E-02	7.3	6.8	7.5	5.9	7	6.4	serine/threonine protein kinase, FIKK family	TryThrA
Down	PF3D7_0315900	-0.81061	3.17E-02	6.7	6	6.6	5.5	6	5.7	conserved Plasmodium protein, unknown function	N/A
Down	PF3D7_1335200	-0.80912	4.88E-02	6.1	6.2	6.8	5.2	6	5.6	reticulocyte binding protein homologue 6, pseudogene	N/A
Down	PF3D7_1407900	-0.80787	2.64E-04	9.7	9.1	9.8	8.6	9	8.9	plasmepsin I	HRP2
Down	PF3D7_1001100	-0.79616	1.32E-03	8.6	8	8.9	7.6	8	7.9	acyl-CoA binding protein, isoform 1, ACBP1	VAR
Down	PF3D7_0831500	-0.79487	1.57E-04	9.8	9.5	10	8.7	9	9.3	Plasmodium exported protein (PHIST), unknown function	FIKK9.1
Down	PF3D7_1001500	-0.78285	2.26E-03	9.7	8.9	9.7	8.3	9	8.9	early transcribed membrane protein 10.1	FIKK9.4
Down	PF3D7_1253100	-0.78109	1.13E-02	6.7	6.7	7	5.9	6	6.1	Plasmodium exported protein (PHISTa), unknown function	FIKK9.6
Down	PF3D7_0730900	-0.77871	1.57E-04	9.1	8.7	9.2	8	8	8.4	EMP1-trafficking protein	N/A
Down	PF3D7_0424900	-0.7735	1.49E-02	8.2	7.6	8.7	7.2	8	7.7	Plasmodium exported protein (PHISTa), unknown function	N/A
Down	PF3D7_1252800	-0.77174	1.59E-04	10	10	11	9.2	10	9.7	Plasmodium exported protein (PHISTb), unknown function	HoMu
Down	PF3D7_0731000	-0.77046	8.22E-04	8.7	8.6	9	7.8	8	8.4	non-coding RNA	N/A
Down	PF3D7_1404700	-0.77017	6.75E-04	8.2	7.8	8.4	7.4	7	7.4	cysteine-rich small secreted protein CSS, putative	LRR8
Down	PF3D7_0902000	-0.77002	5.49E-02	6.1	5.9	6.5	5.2	5	5.7	serine/threonine protein kinase, FIKK family	N/A
Down	PF3D7_0814500	-0.76835	7.87E-03	8.4	7.8	8.6	7.1	8	7.9	conserved protein, unknown function	GIG
Down	PF3D7_1477700	-0.76743	9.06E-02	6	5.6	6.5	5.4	5	5.5	Plasmodium exported protein (PHISTa), unknown function	REX3

Down	PF3D7_1218100	-0.76594	5.03E-02	6.7	5.9	6.7	5.8	6	5.8	conserved Plasmodium protein, unknown function	ACBP1
Down	PF3D7_0301600	-0.76545	1.82E-03	8.1	7.7	8.2	6.9	7	7.4	Plasmodium exported protein (hyp1), unknown function	XL1
Down	PF3D7_1244500	-0.76515	7.95E-02	6.3	6.1	6.5	4.9	6	5.9	PIMMS57 protein	ETRAMP10
Down	PF3D7_1462400	-0.76015	5.37E-04	9.3	8.9	9.6	8.3	9	8.6	conserved Plasmodium protein, unknown function	PfJ23
Down	PF3D7_0301800	-0.75496	8.93E-04	9.3	9.1	9.7	8.4	9	9	Plasmodium exported protein, unknown function	N/A
Down	PF3D7_0935600	-0.74706	1.08E-03	12	11	12	10	11	11	gametocytogenesis-implicated protein	PTP5
Down	PF3D7_1372300	-0.73943	9.76E-02	6.4	5.6	6.8	5.5	6	5.6	Plasmodium exported protein (PHIST), unknown function	N/A
Down	PF3D7_0716100	-0.73023	6.98E-02	6.5	6.1	6.9	5.4	6	6.1	protein SDA1, putative	N/A
Down	PF3D7_1450000	-0.72507	2.57E-04	9.1	8.9	9.4	8.3	8	8.6	serine/threonine protein kinase, putative	N/A
Down	PF3D7_0731200	-0.72248	2.54E-04	8.6	8.5	8.6	7.8	8	8.1	Plasmodium exported protein, unknown function	FIKK10.2
Down	PF3D7_1114200	-0.71627	6.41E-04	9	8.8	9.3	8.4	8	8.5	GTPase-activating protein, putative	ETRAMP11.2
Down	PF3D7_0702200	-0.70526	8.19E-05	10	10	11	9.6	10	10	lysophospholipase LPL20	N/A
Down	PF3D7_1148800	-0.7045	3.30E-02	6.6	6.5	6.8	5.9	6	6.1	Plasmodium exported protein (hyp11), unknown function	HSP101
Down	PF3D7_1329500	-0.69624	6.75E-04	11	11	11	10	10	10	conserved protein, unknown function	N/A
Down	PF3D7_1416500	-0.69556	2.97E-06	10	10	10	9.6	10	9.8	NADP-specific glutamate dehydrogenase	CAP93
Down	PF3D7_1455100	-0.69409	1.18E-03	8.7	8.4	9	8	8	8.1	protein tyrosine phosphatase, putative	N/A
Down	PF3D7_1016700	-0.69361	1.09E-02	8.6	7.9	8.9	7.7	8	8	Plasmodium exported protein (PHISTc), unknown function	N/A
Down	PF3D7_0906600	-0.693	5.37E-04	9.5	9.1	9.4	8.6	8	8.9	zinc finger protein, putative	N/A
Down	PF3D7_1337800	-0.69275	9.08E-07	11	11	11	11	11	11	calcium-dependent protein kinase 5	RESA3

Down	PF3D7_1039000	-0.68375	2.90E-02	7	6.7	7.3	6.2	6	6.5	serine/threonine protein kinase, FIKK family	FIKK11
Down	PF3D7_1116800	-0.68346	1.55E-06	13	13	13	12	13	13	heat shock protein 101	N/A
Down	PF3D7_1367400	-0.68234	4.07E-02	7.1	6.9	6.9	5.9	6	6.7	conserved Plasmodium protein, unknown function	RIF
Down	PF3D7_0716200	-0.68134	1.09E-02	8	7.6	7.9	6.8	7	7.5	PDCD2 domain-containing protein, putative	VAR
Down	PF3D7_1002100	-0.67958	7.73E-04	11	11	12	10	11	11	EMP1-trafficking protein	VAR2CSA
Down	PF3D7_1357700	-0.67066	6.21E-02	6.6	6.3	6.6	5.6	6	6.1	U3 small nucleolar RNA-associated protein 21, putative	FIKK12
Down	PF3D7_0501400	-0.66782	3.93E-03	12	11	12	11	11	11	interspersed repeat antigen	N/A
Down	PF3D7_1477800	-0.66624	5.86E-03	7.9	8.1	8.4	7.4	7	7.8	acyl-CoA binding protein	N/A
Down	PF3D7_0401900	-0.6656	3.82E-04	11	11	11	10	10	10	acyl-CoA synthetase	N/A
Down	PF3D7_0702100	-0.66425	2.80E-03	10	10	11	9.7	9	10	Plasmodium exported protein (PHISTb), unknown function, pseudogene	N/A
Down	PF3D7_1329800	-0.66418	1.16E-02	7.5	7.3	7.5	6.5	7	6.9	conserved Plasmodium protein, unknown function	AP2-G
Down	PF3D7_1460100	-0.66416	7.55E-03	7.7	7.6	8	6.9	7	7	FYVE and coiled-coil domain-containing protein	N/A
Down	PF3D7_0630700	-0.66299	3.02E-02	7	7.2	7.6	6.4	7	6.9	bifunctional methylenetetrahydrofolate dehydrogenase/cyclohydrolase, putative	VAR
Down	PF3D7_1421100	-0.66253	6.87E-03	8	7.7	8.2	7.2	7	7.6	conserved Plasmodium protein, unknown function	VAR
Down	PF3D7_0424700	-0.66232	3.23E-02	8.3	7.3	7.8	7.1	7	7.4	serine/threonine protein kinase, FIKK family	RFC4
Down	PF3D7_0630100	-0.66042	2.38E-02	7.5	7.1	7.6	6.5	7	6.8	alpha/beta hydrolase, putative	PIMMS57
Down	PF3D7_0501300	-0.66013	4.85E-03	10	9.4	10	8.9	9	9.4	skeleton-binding protein 1	N/A
Down	PF3D7_1322100	-0.65793	7.01E-03	8.9	8.4	9.2	8.1	8	8.3	histone-lysine N-methyltransferase SET2	N/A
Down	PF3D7_1149600	-0.65621	1.06E-03	10	10	11	9.6	10	10	DnaJ protein, putative	N/A

Down	PF3D7_0402100	-0.65589	1.59E-04	12	12	12	11	11	12	Plasmodium exported protein (PHISTb), unknown function	GECO
Down	PF3D7_0507300	-0.65474	1.82E-03	10	9.9	10	9.2	10	9.6	subtilisin-like ookinete protein SOPT	N/A
Down	PF3D7_0301200	-0.65422	2.37E-02	7.3	7	7.5	6.4	7	6.8	serine/threonine protein kinase, FIKK family	N/A
Down	PF3D7_0830500	-0.65282	1.82E-03	12	11	12	11	11	11	sporozoite and liver stage tryptophan-rich protein, putative	CBP2
Down	PF3D7_1020700	-0.64976	5.17E-02	7.2	6.7	7.2	6.1	6	6.7	N-acetyltransferase, GNAT family, putative	PTP6
Down	PF3D7_1213900	-0.64768	3.39E-03	8.8	8.4	9	8.1	8	8.2	W2 domain-containing protein, putative	N/A
Down	PF3D7_0219900	-0.64078	1.09E-02	8.3	8.2	8.8	7.7	8	8.1	Plasmodium exported protein, unknown function	DHHC8
Down	PF3D7_0730800	-0.63722	4.82E-03	11	11	11	10	10	11	Plasmodium exported protein, unknown function	SET2
Down	PF3D7_0930200	-0.6358	3.90E-03	11	11	11	10	11	11	leucine-rich repeat protein	N/A
Down	PF3D7_1016800	-0.63548	9.25E-04	11	11	11	10	10	11	Plasmodium exported protein (PHISTc), unknown function	N/A
Down	PF3D7_0701900	-0.63411	3.66E-03	11	11	12	11	11	11	Plasmodium exported protein, unknown function	RH6
Down	PF3D7_1251600	-0.63244	2.87E-02	7.5	7.5	8	6.7	7	7.3	conserved Plasmodium protein, unknown function	CDPK5
Down	PF3D7_1149200	-0.63119	1.33E-03	15	14	15	14	14	14	ring-infected erythrocyte surface antigen	N/A
Down	PF3D7_1446300	-0.63107	7.73E-04	11	10	11	9.8	10	10	conserved Plasmodium membrane protein, unknown function	N/A
Down	PF3D7_1409500	-0.62885	2.86E-02	7.8	7.7	8.1	6.7	7	7.4	conserved Plasmodium protein, unknown function	MAHRP2
Down	PF3D7_1217500	-0.62853	3.79E-03	8.9	8.6	9.2	8.2	8	8.2	conserved Plasmodium protein, unknown function	PK5
Down	PF3D7_0906500	-0.62678	5.34E-03	9.4	9.1	9.8	8.6	9	9.1	arginase	UTP21

Down	PF3D7_1472500	-0.62475	7.95E-02	6.3	6.6	6.6	5.8	6	6	conserved Plasmodium protein, unknown function	N/A
Down	PF3D7_1411100	-0.6238	2.08E-03	10	10	11	9.5	10	10	conserved Plasmodium membrane protein, unknown function	MAHRP1
Down	PF3D7_1411000	-0.6213	3.79E-03	13	12	13	12	12	12	conserved Plasmodium protein, unknown function	N/A
Down	PF3D7_0805300	-0.62111	2.27E-03	12	12	12	11	12	11	zinc finger protein, putative	N/A
Down	PF3D7_0623800	-0.6174	9.76E-04	12	12	13	12	12	12	tyrosine kinase-like protein, putative	N/A
Down	PF3D7_1471100	-0.61676	1.20E-04	11	11	11	11	11	11	exported protein 2	N/A
Down	PF3D7_1201200	-0.61599	2.37E-03	10	10	10	9.3	10	9.9	Plasmodium exported protein (PHISTa-like), unknown function	N/A
Down	PF3D7_0316100	-0.61546	2.08E-03	8.8	8.7	9	8	8	8.3	ribosomal protein L27, mitochondrial, putative	N/A
Down	PF3D7_1321400	-0.61238	5.81E-03	8.5	8.2	8.8	7.9	8	7.8	palmitoyltransferase DHHC8, putative	PMI
Down	PF3D7_1232400	-0.61013	3.93E-02	7.9	7.8	8.3	6.9	8	7.7	CWC16 domain-containing protein, putative	N/A
Down	PF3D7_1427100	-0.61009	6.44E-03	9.9	9.5	10	9	9	9.5	lipase, putative	N/A
Down	PF3D7_0501100	-0.61006	2.18E-03	10	10	10	9.5	10	10	co-chaperone J domain protein JDP	N/A
Down	PF3D7_1133700	-0.60833	1.38E-02	9	8.5	9.3	8.1	8	8.6	FHA domain-containing protein, putative	DPH1
Down	PF3D7_0731100	-0.60644	1.52E-03	11	10	11	9.7	10	9.9	EMP1-trafficking protein	GDH1
Down	PF3D7_0916700	-0.60501	2.64E-04	10	10	10	9.7	10	9.7	RNA-binding protein musashi, putative	N/A
Down	PF3D7_0531600	-0.60483	6.26E-02	7.3	7.1	7	6.9	7	6.1	18S ribosomal RNA	N/A
Down	PF3D7_0931100	-0.60463	3.93E-02	8.8	8.4	9.3	8	8	8.5	nucleolar protein Nop52, putative	N/A
Down	PF3D7_1222600	-0.60073	6.98E-02	7.1	7	7.7	6.5	7	7	AP2 domain transcription factor AP2-G	N/A
Down	PF3D7_0731400	-0.59888	9.47E-03	10	9.7	10	9	10	9.5	serine/threonine protein kinase, FIKK family, pseudogene	PTP1
Down	PF3D7_0104300	-0.59576	1.43E-03	14	14	14	13	13	14	ubiquitin carboxyl-terminal hydrolase 1, putative	FCP
Down	PF3D7_0402300	-0.5927	8.59E-03	14	14	14	13	14	14	reticulocyte binding protein homologue 1	N/A

Down	PF3D7_1143800	-0.59098	6.41E-04	11	11	11	10	10	10	oocyst capsule protein Cap93, putative	EXP2
Down	PF3D7_0323500	-0.58969	1.24E-03	9.8	9.7	10	9	9	9.3	survival motor neuron-like protein	N/A
Down	PF3D7_0424200	-0.58867	2.11E-03	13	12	13	12	12	12	reticulocyte binding protein homologue 4	Pfg14-748
Down	PF3D7_0318600	-0.58785	1.90E-02	9	8.9	9.4	8.1	9	8.7	cleavage and polyadenylation specificity factor, putative	ACBP
Down	PF3D7_0729300	-0.58737	9.36E-02	6.9	6.5	7.3	6.2	6	6.5	60S ribosomal export protein NMD3, putative	GEXP17
Down	PF3D7_1102800	-0.58624	7.16E-03	14	14	15	13	14	14	early transcribed membrane protein 11.2	N/A

Table S3D. Differential expression between PfDNMT2 complementation and wildtype parasite at the ring stage

R: replicate, WT: 3D7 wild type, CO: PfDNMT2 complementation, Adj.Pval: adjusted P value

columns G-L is EdgeR log2 (counts per million + count)

Change pattern	Gene ID	log2 Fold Change	Adj.Pval	WT R1	WT R2	WT R3	CO R1	CO R2	CO R3	Product Description	Name or Symbol
Up	PF3D7_0425500	1.493456	3.28E-03	5.8	4.9	5.4	7.4	6	7	stevor	N/A
Up	PF3D7_0425600	1.576026	5.34E-03	4.6	4.2	4.8	6.2	6	6	rifin, pseudogene	RIF
Up	PF3D7_0713200	2.394687	8.24E-30	5.9	5.9	6.1	8	8	8	exported protein family 1	EPF1
Up	PF3D7_0713300	2.370088	1.44E-87	8	8.2	8.1	10	10	11	erythrocyte membrane protein 1 (PfEMP1), pseudogene	VAR
Up	PF3D7_0727300	3.949777	2.03E-21	8.3	8.6	8.5	13	11	13	DNA (cytosine-5)-methyltransferase	DNMT
Up	PF3D7_0809000	4.895707	7.45E-02	2	2.3	2	3.8	3	4	Plasmodium RNA of unknown function RUF6	N/A
Up	PF3D7_0809100	3.542278	2.60E-211	8.9	9.2	8.8	13	12	13	erythrocyte membrane protein 1, PfEMP1	VAR
Up	PF3D7_1101300	3.927666	7.10E-07	2.6	2.6	3.2	6.1	5	6	rifin	RIF
Up	PF3D7_1372000	0.769843	1.38E-02	6.6	6.6	6.9	7.5	7	7	Plasmodium exported protein (PHISTa), unknown function	N/A
Down	PF3D7_0110700	-1.03052	2.21E-02	7.3	6.8	6.5	5.9	6	6	chromatin assembly factor 1 subunit C, putative	CAF1C
Down	PF3D7_0214300	-1.06285	5.00E-02	6.4	6.2	6.5	5.2	5	6	conserved Plasmodium protein, unknown function	N/A
Down	PF3D7_0302300	-4.12838	8.33E-54	8.3	8.6	8.4	4.2	5	4	erythrocyte membrane protein 1 (PfEMP1), pseudogene	N/A
Down	PF3D7_0302500	-2.92347	6.95E-04	5	5.2	5.1	2.8	3	3	cytoadherence linked asexual protein 3.1	CLAG3.1
Down	PF3D7_0412400	-1.80028	6.76E-41	10	11	11	9.1	9	9	erythrocyte membrane protein 1, PfEMP1	VAR
Down	PF3D7_0412700	-0.74169	9.39E-03	9	9.3	9	8.7	8	9	erythrocyte membrane protein 1, PfEMP1	VAR
Down	PF3D7_0413400	-2.90752	8.58E-07	5.9	5.6	5.7	3	4	3	erythrocyte membrane protein 1 (PfEMP1), exon 1, pseudogene	VAR
Down	PF3D7_0420700	-1.28654	7.45E-04	6.5	7.1	6.9	5.4	6	6	erythrocyte membrane protein 1, PfEMP1	VAR
Down	PF3D7_0420900	-2.00056	2.40E-10	6.9	7.2	6.9	5.2	5	5	erythrocyte membrane protein 1, PfEMP1	VAR
Down	PF3D7_0421100	-1.79249	7.47E-09	6.8	7.2	6.9	5.5	5	5	erythrocyte membrane protein 1, PfEMP1	VAR

Down	PF3D7_0421300	-1.91028	1.26E-31	9.1	9.5	9.2	7.4	8	7	erythrocyte membrane protein 1, PfEMP1	VAR
Down	PF3D7_0422300	-0.76708	3.77E-07	9.1	9.2	9.3	8.6	8	8	alpha tubulin 2	N/A
Down	PF3D7_0425100	-1.4981	6.28E-02	5.4	5.8	5.9	4.3	5	4	Plasmodium exported protein (hyp6), unknown function	N/A
Down	PF3D7_0425200	-1.71333	2.31E-05	6.4	6.4	6.3	5	5	5	Plasmodium exported protein (hyp15), unknown function	N/A
Down	PF3D7_0532800	-2.93339	1.31E-03	4.7	5	5.2	3	3	3	stevor, pseudogene	N/A
Down	PF3D7_0532900	-3.13789	5.35E-04	5	5.2	5	2.3	3	3	rifin	RIF
Down	PF3D7_0533100	-0.97546	8.07E-05	8.1	8.2	7.9	7.4	7	7	erythrocyte membrane protein 1 (PfEMP1), pseudogene	VAR1CSA
Down	PF3D7_0600200	-1.82676	2.06E-14	7.6	8.3	8.1	6.3	6	6	erythrocyte membrane protein 1, PfEMP1	VAR
Down	PF3D7_0617400	-1.80129	1.28E-12	7.4	7.8	7.4	5.9	6	6	erythrocyte membrane protein 1, PfEMP1	VAR
Down	PF3D7_0632500	-1.82121	1.64E-32	9.4	9.8	9.4	8	8	8	erythrocyte membrane protein 1, PfEMP1	VAR
Down	PF3D7_0701600	-1.82591	7.45E-02	6.2	4.6	5.3	4.2	3	4	Pfmc-2TM Maurer's cleft two transmembrane protein	MC-2TM
Down	PF3D7_0711700	-3.6356	3.25E-228	12	12	12	8.8	9	8	erythrocyte membrane protein 1, PfEMP1	VAR
Down	PF3D7_0712000	-1.35911	2.68E-13	8.5	9	8.6	7.5	7	7	erythrocyte membrane protein 1, PfEMP1	VAR
Down	PF3D7_0712300	-1.35242	9.22E-08	7.6	8.1	7.6	6.6	7	6	erythrocyte membrane protein 1, PfEMP1	VAR
Down	PF3D7_0712400	-1.51861	1.62E-04	6.3	6.9	6.5	5	5	5	erythrocyte membrane protein 1, PfEMP1	VAR
Down	PF3D7_0712600	-2.44021	7.10E-42	9.3	9.7	9.2	7.1	7	7	erythrocyte membrane protein 1, PfEMP1	VAR
Down	PF3D7_0712800	-4.57283	2.50E-49	8.5	8.7	7.9	4	4	4	erythrocyte membrane protein 1, PfEMP1	VAR
Down	PF3D7_0712900	-2.62821	1.99E-42	9.4	9.7	9.3	7	7	6	erythrocyte membrane protein 1, PfEMP1	VAR
Down	PF3D7_0808600	-0.75853	6.61E-04	9	9.5	8.9	8.4	9	8	erythrocyte membrane protein 1, PfEMP1	VAR
Down	PF3D7_0808700	-2.16125	1.60E-10	6.9	7.3	7	4.9	6	5	erythrocyte membrane protein 1, PfEMP1	VAR
Down	PF3D7_1026600	-0.72561	5.77E-03	8.1	8.4	8.8	7.6	8	8	conserved Plasmodium protein, unknown function	N/A
Down	PF3D7_1115500	-0.93963	1.87E-03	7.1	7.3	7.2	6.4	6	6	AP2 domain transcription factor, putative	ApiAP2
Down	PF3D7_1150400	-1.01174	4.56E-03	6.9	7.3	6.9	6.2	6	6	erythrocyte membrane protein 1, PfEMP1	VAR
Down	PF3D7_1219300	-2.99248	1.16E-22	7.3	7.8	7.5	4.4	5	5	erythrocyte membrane protein 1, PfEMP1	VAR

Down	PF3D7_1219400	-3.03594	1.17E-52	9.2	9.5	9.4	6.3	7	6	erythrocyte membrane protein 1 (PfEMP1), pseudogene	VAR
Down	PF3D7_1240400	-2.68468	2.60E-98	11	11	11	8.7	9	8	erythrocyte membrane protein 1, PfEMP1	VAR
Down	PF3D7_1240500	-6.41252	6.10E-04	3.8	4.2	4.7	2	2	2	Plasmodium RNA of unknown function RUF6	N/A
Down	PF3D7_1240600	-4.07478	8.48E-233	13	13	13	9.1	9	9	erythrocyte membrane protein 1, PfEMP1	VAR
Down	PF3D7_1253000	-0.93849	1.84E-08	9	9.2	8.8	7.9	8	8	gametocyte erythrocyte cytosolic protein	GECO
Down	PF3D7_1319400	-0.9115	1.28E-02	7.5	6.9	7.1	6.2	6	6	conserved protein, unknown function	N/A
Down	PF3D7_1330600	-2.16342	7.11E-02	6	4.6	4.2	3	3	4	elongation factor Tu, putative	N/A
Down	PF3D7_1334500	-2.15875	9.25E-02	6.4	4.9	3.9	3	4	4	MSP7-like protein	MSRP6
Down	PF3D7_1372300	-0.58983	3.24E-04	9.3	9.6	9.5	9	9	9	Plasmodium exported protein (PHIST), unknown function	N/A
Down	PF3D7_1460400	-0.71154	5.56E-04	8.1	8.3	8.3	7.5	8	8	ubiquitin carboxyl-terminal hydrolase isozyme L3	UCHL3
Down	PF3D7_1477000	-1.14412	5.49E-17	11	11	11	9.8	10	10	Plasmodium exported protein (hyp17), unknown function, pseudogene	N/A
Down	PF3D7_1479700	-5.25043	9.25E-02	3.3	3.6	3.5	2	2	2	rifin	RIF

Table S3E. Differential expression between PfDNMT2 complementation and WT parasite at trophozoite stage

R: replicate, WT: 3D7 wild type, CO: PfDNMT2 complementation, Adj.Pval: adjusted P value

columns G-L is EdgeR log2 (counts per million + count)

Change pattern	Gene ID	log2 Fold Change	Adj.Pval	WT R1	WT R2	WT R3	CO R1	CO R2	CO R3	Product Description	Name or Symbol
Up	PF3D7_0727300	5.636534	1.87E-12	7.3	7.5	7.7	13	11	14	DNA (cytosine-5)-methyltransferase	DNMT
Up	PF3D7_0833500	2.243359	4.33E-10	4.4	4.2	4.9	6	6.8	6.4	erythrocyte membrane protein 1, PfEMP1	VAR
Up	PF3D7_0713100	1.915338	4.40E-06	4.2	4.9	4.1	6	6.3	6.2	Pfmc-2TM Maurer's cleft two transmembrane protein	MC-2TM
Up	PF3D7_0700800	1.697459	5.60E-03	3.9	4.8	3.9	5	5.9	6	Pfmc-2TM Maurer's cleft two transmembrane protein	MC-2TM
Up	PF3D7_0425500	1.136871	4.62E-03	5.3	5.2	5.3	6	6.3	6.5	stevor	N/A
Up	PF3D7_0809100	0.944452	1.37E-06	7.2	7.1	7.3	8	8.3	8.2	erythrocyte membrane protein 1, PfEMP1	VAR
Down	PF3D7_0532900	-5.90276	4.62E-03	3.5	4	4.2	2	2	2	rifin	RIF
Down	PF3D7_0532800	-4.71957	2.64E-04	5	5.2	4.5	3	2.3	2	stevor, pseudogene	N/A
Down	PF3D7_0302500	-3.465012	2.61E-63	11	10	11	7	7.4	7.2	cytoadherence linked asexual protein 3.1	CLAG3.1
Down	PF3D7_0533000	-3.303179	5.10E-09	5.8	5.6	5.8	3	3.2	3.5	rifin, pseudogene	RIF
Down	PF3D7_1401000	-3.056913	3.47E-10	5.9	6.3	5.8	3	3.6	3.6	GBPH protein	GBPH
Down	PF3D7_1400100	-3.026235	1.13E-02	5	4.2	4.2	3	2.8	2.3	erythrocyte membrane protein 1 (PfEMP1), pseudogene	N/A
Down	PF3D7_1240600	-2.693261	6.46E-09	6.1	5.8	6.1	4	3.2	3.9	erythrocyte membrane protein 1, PfEMP1	VAR
Down	PF3D7_1477400	-2.261682	9.08E-11	6.6	6.9	6.3	5	4.4	4.6	Plasmodium exported protein (PHIST), unknown function	N/A
Down	PF3D7_1240400	-2.170464	6.67E-04	5.1	5.4	5.4	3	3.9	3.6	erythrocyte membrane protein 1, PfEMP1	VAR

Down	PF3D7_1100800	-2.024563	9.48E-02	4.5	4.8	4.5	3	3.2	3	Pfmc-2TM Maurer's cleft two transmembrane protein	MC-2TM
Down	PF3D7_0701600	-1.798051	4.05E-04	5.6	5.8	5.8	4	4.5	4.2	Pfmc-2TM Maurer's cleft two transmembrane protein	MC-2TM
Down	PF3D7_0114100	-1.682588	1.14E-03	5.6	6	5.8	4	3.8	4.6	Pfmc-2TM Maurer's cleft two transmembrane protein	MC-2TM
Down	PF3D7_0533100	-1.631017	1.87E-12	8	7.9	8.5	6	6.7	6.8	erythrocyte membrane protein 1 (PfEMP1), pseudogene	VAR1CSA
Down	PF3D7_1372500	-1.483363	5.06E-02	5.1	5.5	5.4	4	4.4	3.9	stevor, pseudogene	N/A
Down	PF3D7_1253000	-1.154382	1.03E-11	9.3	9.2	9.4	8	8.4	7.9	gametocyte erythrocyte cytosolic protein	GECO
Down	PF3D7_0114300	-1.060747	4.62E-03	6.9	7.1	6.6	6	5.7	5.7	exported protein family 4, pseudogene	EPF4

Table S3F. Differential expression between PfDNMT2 complementation and wildtype parasite at schizont stage

R: replicate, WT: 3D7 wild type, CO: PfDNMT2 complementation, Adj.Pval: adjusted P value

columns G-L is EdgeR log2 (counts per million + count)

Change pattern	Gene ID	log2 Fold Change	Adj.Pval	WT R1	WT R2	WT R3	CO R1	CO R2	CO R3	Product Description	Name or Symbol
Up	PF3D7_0727300	3.9929861	3.60E-18	6.78	6.71	6.6	11	9.7	11	DNA (cytosine-5)-methyltransferase	DNMT
Up	PF3D7_0713200	2.5589149	3.67E-02	3.17	2.8	2.3	4.8	4.3	4.2	exported protein family 1	EPF1
Up	PF3D7_0700800	2.3993667	3.67E-02	3.32	2.32	3	4.2	4.5	4.9	Pfmc-2TM Maurer's cleft two transmembrane protein	MC-2TM
Up	PF3D7_0833500	1.923531	4.63E-16	5.78	5.72	5.2	7.4	7.3	7.6	erythrocyte membrane protein 1, PfEMP1	VAR
Up	PF3D7_0425500	1.4920792	1.50E-11	6.2	6.53	6	7.6	7.6	7.9	stevor	N/A
Up	PF3D7_0809100	1.2683416	1.61E-17	7.94	8.14	8	9.3	9.1	9.4	erythrocyte membrane protein 1, PfEMP1	VAR
Up	PF3D7_0302200	1.2532365	1.80E-13	12.1	12.1	12	13	13	14	cytoadherence linked asexual protein 3.2	CLAG3.2
Up	PF3D7_0808900	1.1400779	4.49E-06	6.67	6.37	6.1	7.6	7.3	7.7	rifin	RIF
Up	PF3D7_0808800	1.000934	2.05E-02	5.64	5.55	5.2	6.5	6.2	6.5	rifin	RIF
Down	PF3D7_0711700	-2.8598179	8.50E-08	5.58	5.39	5.9	3.6	3	3.5	erythrocyte membrane protein 1, PfEMP1	VAR
Down	PF3D7_0302500	-2.774724	7.93E-133	15	15	15	12	12	12	cytoadherence linked asexual protein 3.1	CLAG3.1
Down	PF3D7_1240600	-2.6324167	4.19E-16	6.89	6.55	7.1	4	4.8	4.5	erythrocyte membrane protein 1, PfEMP1	VAR
Down	PF3D7_0712000	-2.0191849	3.67E-02	4.17	4.9	5.2	3.3	3.5	3.2	erythrocyte membrane protein 1, PfEMP1	VAR
Down	PF3D7_0425100	-1.9640347	8.21E-11	6.84	6.62	7.3	5	5.4	4.9	Plasmodium exported protein (hyp6), unknown function	N/A
Down	PF3D7_1240400	-1.8661056	2.97E-13	7.04	7.01	7.1	5.3	5.2	5.4	erythrocyte membrane protein 1, PfEMP1	VAR
Down	PF3D7_0425200	-1.8260522	2.97E-13	7.44	7.24	7.7	5.8	6	5.3	Plasmodium exported protein (hyp15), unknown function	N/A

Down	PF3D7_0302300	-1.6025633	9.73E-02	5.04	4.75	5	3.7	3.8	3.5	erythrocyte membrane protein 1 (PfEMP1), pseudogene	N/A
Down	PF3D7_0533100	-1.3378257	1.04E-07	8.12	8.95	8.4	7.1	6.9	7.6	erythrocyte membrane protein 1 (PfEMP1), pseudogene	VAR1CSA
Down	PF3D7_1253000	-1.297656	3.59E-07	8.11	7.94	8.6	6.7	7.4	6.7	gametocyte erythrocyte cytosolic protein	GECO

Table S3G. Differential expression between PfDNMT2 overexpression and wildtype parasite at the ring stage

R: replicate, WT: 3D7 wild type, OE: PfDNMT2 overexpression, Adj.Pval: adjusted P value

columns G-L is EdgeR log2 (counts per million + count)

Change pattern	Gene ID	log2 Fold Change	Adj.Pval	WT R1	WT R2	WT R3	OE R1	OE R2	OE R3	Product Description	Name or Symbol
Up	PF3D7_0800200	10.75574	0.00E+00	3.8	4	4.1	14	14	14	erythrocyte membrane protein 1, PfEMP1	VAR
Up	PF3D7_0800300	10.20571	0.00E+00	5.4	5.81	5.9	16	16	16	erythrocyte membrane protein 1, PfEMP1	VAR
Up	PF3D7_0300100	8.821357	3.34E-74	3.5	4.52	3	12	12	12	erythrocyte membrane protein 1, PfEMP1	VAR
Up	PF3D7_1150400	8.332895	0.00E+00	6.9	7.37	6.9	15	15	15	erythrocyte membrane protein 1, PfEMP1	VAR
Up	PF3D7_1150300	7.979936	1.24E-48	2.3	2.58	3.2	9.6	9.4	9	rifin	RIF
Up	PF3D7_0221400	7.247531	2.20E-07	2	2	2.3	5.9	6.2	5	stevor	N/A
Up	PF3D7_0733000	6.535049	6.27E-203	4.2	5.21	4.7	11	11	11	erythrocyte membrane protein 1, PfEMP1	VAR
Up	PF3D7_0632500	6.487348	0.00E+00	9.5	9.8	9.5	16	16	16	erythrocyte membrane protein 1, PfEMP1	VAR
Up	PF3D7_0632600	6.476793	2.35E-73	3.3	3.58	3.5	9.4	9.4	9	rifin, pseudogene	RIF
Up	PF3D7_0413400	6.23292	0.00E+00	5.9	5.64	5.8	12	12	12	erythrocyte membrane protein 1 (PfEMP1), exon 1, pseudogene	VAR
Up	PF3D7_1000100	6.091386	5.31E-133	3.7	4.52	4.5	10	10	10	erythrocyte membrane protein 1, PfEMP1	VAR
Up	PF3D7_0100300	5.755835	9.46E-80	3.5	4.17	3.8	9.2	9.2	9	erythrocyte membrane protein 1, PfEMP1	VAR
Up	PF3D7_1240200	5.637826	3.83E-16	2	2.81	2.8	6.6	6.7	7	erythrocyte membrane protein 1 (PfEMP1), pseudogene	N/A
Up	PF3D7_0413300	5.604486	1.56E-34	3	3.17	3.2	8.1	7.5	8	rifin	RIF
Up	PF3D7_1300300	5.561382	0.00E+00	7	7.23	6.9	13	12	13	erythrocyte membrane protein 1, PfEMP1	VAR
Up	PF3D7_0412400	5.387153	0.00E+00	10	10.9	11	16	16	16	erythrocyte membrane protein 1, PfEMP1	VAR
Up	PF3D7_0937600	5.253868	9.51E-41	3.3	3.46	3.3	8	7.9	8	erythrocyte membrane protein 1, PfEMP1	VAR
Up	PF3D7_1100200	5.20455	2.08E-292	6.3	6.71	6.3	12	12	12	erythrocyte membrane protein 1, PfEMP1	VAR
Up	PF3D7_0600400	5.123224	1.83E-18	2.3	2.58	3.3	6.8	6.8	7	erythrocyte membrane protein 1, PfEMP1	VAR
Up	PF3D7_0400100	5.028358	4.16E-90	4.9	4.46	3.9	9.2	9.2	9	erythrocyte membrane protein 1, PfEMP1	VAR
Up	PF3D7_0425800	4.979512	0.00E+00	6.7	7.05	6.9	12	12	12	erythrocyte membrane protein 1, PfEMP1	VAR
Up	PF3D7_0402600	4.934185	1.11E-02	2	2	2.3	3.8	4.1	4	stevor	N/A
Up	PF3D7_0115700	4.860138	8.12E-49	4.2	4	3	8.3	8.1	8	erythrocyte membrane protein 1, PfEMP1	VAR

Up	PF3D7_0412600	4.851861	8.15E-11	2.6	2.81	2.3	5.9	6	6	rifin, pseudogene	RIF
Up	PF3D7_0833500	4.754725	3.15E-130	5	5.75	5.2	10	9.9	10	erythrocyte membrane protein 1, PfEMP1	VAR
Up	PF3D7_0100100	4.626151	1.96E-37	6.2	6.04	6.4	11	11	10	erythrocyte membrane protein 1, PfEMP1	VAR
Up	PF3D7_0708600	4.543906	1.11E-38	4	3.32	3.8	7.8	7.6	8	inner membrane complex protein 1d, putative	IMC1d
Up	PF3D7_0412900	4.541099	1.44E-11	2	3.17	2.8	5.9	6.2	6	erythrocyte membrane protein 1, PfEMP1	VAR
Up	PF3D7_1100300	4.344466	7.99E-13	2.3	2.81	3.3	6.1	6.1	6	rifin	RIF
Up	PF3D7_1400300	4.316149	1.25E-02	2.3	2	2.3	4.6	3.9	4	rifin	RIF
Up	PF3D7_0221500	4.272856	9.85E-15	3.3	2.81	2.8	6.2	6.5	6	Pfmc-2TM Maurer's cleft two transmembrane protein, pseudogene	N/A
Up	PF3D7_0425900	4.242662	1.27E-03	2	2.81	2	4.6	4.6	4	rifin	RIF
Up	PF3D7_0412500	4.242077	1.15E-09	2.6	2.81	2.8	5.8	6	5	Plasmodium RNA of unknown function RUF6	N/A
Up	PF3D7_0800100	4.156706	3.07E-78	4.7	5.28	5.1	9.1	9.1	9	erythrocyte membrane protein 1, PfEMP1	VAR
Up	PF3D7_0425700	3.966737	1.15E-09	3.2	3	2.3	6.1	5.6	6	rifin	RIF
Up	PF3D7_0413500	3.963317	6.99E-47	5	4.25	4.1	8.3	8.3	8	phosphoglucomutase-2	PGM2
Up	PF3D7_1480100	3.946566	1.75E-24	3.9	4	3.3	7.1	7.6	7	erythrocyte membrane protein 1 (PfEMP1), pseudogene	N/A
Up	PF3D7_0700100	3.94439	1.31E-24	3.8	3.32	3.8	7.1	7.1	7	erythrocyte membrane protein 1, PfEMP1	VAR
Up	PF3D7_0426000	3.925992	2.28E-31	4.1	3.91	3.8	7.5	7.5	7	erythrocyte membrane protein 1, PfEMP1	VAR
Up	PF3D7_1400200	3.801277	5.90E-02	2	2.32	2.3	3.6	3.6	4	rifin	RIF
Up	PF3D7_1100100	3.728138	1.70E-125	6.4	6.19	6.4	10	10	10	erythrocyte membrane protein 1, PfEMP1	VAR
Up	PF3D7_0324000	3.694556	8.90E-02	2	2	2.6	3.9	3.3	4	exported protein family 1	EPF1
Up	PF3D7_0302300	3.689941	1.10E-222	8.3	8.6	8.4	12	12	12	erythrocyte membrane protein 1 (PfEMP1), pseudogene	N/A
Up	PF3D7_0200100	3.669462	3.23E-27	3.9	4.46	3.9	7.7	7.1	7	erythrocyte membrane protein 1, PfEMP1	VAR
Up	PF3D7_0632800	3.662404	5.00E-110	6	6.63	6.4	10	10	10	erythrocyte membrane protein 1, PfEMP1	VAR
Up	PF3D7_0713300	3.492236	2.99E-181	8	8.23	8.1	12	11	12	erythrocyte membrane protein 1 (PfEMP1), pseudogene	VAR
Up	PF3D7_0324900	3.434112	8.65E-79	5.7	6.11	6.2	9.5	9.2	9	erythrocyte membrane protein 1, PfEMP1	VAR

Up	PF3D7_0713200	3.340386	1.29E-46	5.9	5.93	6.1	9	8.8	10	exported protein family 1	EPF1
Up	PF3D7_1480000	3.267229	3.70E-05	2.6	3	2.8	5.1	5.1	5	rifin	RIF
Up	PF3D7_1255200	3.13675	3.44E-22	4.4	4.46	4.1	7.1	7.1	7	erythrocyte membrane protein 1, PfEMP1	VAR
Up	PF3D7_0400500	3.102256	6.63E-04	2.8	2.81	2.6	4.8	4.4	5	rifin	RIF
Up	PF3D7_0727300	3.051681	8.86E-09	8.3	8.67	8.5	11	12	11	DNA (cytosine-5)-methyltransferase	DNMT
Up	PF3D7_1300100	2.806775	1.05E-21	5.2	4.64	4.4	7.4	7.4	7	erythrocyte membrane protein 1, PfEMP1	VAR
Up	PF3D7_0712300	2.786039	3.12E-10	7.6	8.16	7.6	10	11	10	erythrocyte membrane protein 1, PfEMP1	VAR
Up	PF3D7_0600100	2.657022	9.21E-03	2.6	2.58	3.2	4.5	4.9	4	erythrocyte membrane protein 1 (PfEMP1), pseudogene	VAR
Up	PF3D7_0601400	2.50047	1.17E-05	3.3	3.58	3.6	5.6	5.6	5	erythrocyte membrane protein 1 (PfEMP1), pseudogene	VAR
Up	PF3D7_0713100	2.378115	1.12E-10	4.9	4.09	4.2	6.5	6.7	7	Pfmc-2TM Maurer's cleft two transmembrane protein	MC-2TM
Up	PF3D7_1373500	2.335304	1.49E-37	6.3	6.83	6.4	8.8	8.9	9	erythrocyte membrane protein 1, PfEMP1	VAR
Up	PF3D7_0900100	2.285947	2.07E-09	4.4	4.17	4.5	6.3	6.5	6	erythrocyte membrane protein 1, PfEMP1	VAR
Up	PF3D7_1200500	2.181774	8.56E-02	2.6	2.81	3	4.2	4.2	4	rifin	RIF
Up	PF3D7_1200100	2.052127	5.97E-34	6.8	6.74	6.9	8.9	8.8	9	erythrocyte membrane protein 1, PfEMP1	VAR
Up	PF3D7_0201500	2.022103	3.45E-23	6.7	6.41	7.2	9	8.7	9	Plasmodium exported protein (hyp9), unknown function	N/A
Up	PF3D7_0323900	2.011869	9.92E-03	3.7	3.58	2.8	5	5.1	5	erythrocyte membrane protein 1 (PfEMP1), exon 2, pseudogene	VAR
Up	PF3D7_0102600	2.005041	1.01E-20	6.4	6.74	7.3	9	8.9	9	serine/threonine protein kinase, FIKK family	FIKK1
Up	PF3D7_0401800	1.997605	6.96E-28	7	6.67	7.2	8.9	9.1	9	Plasmodium exported protein (PHISTb), unknown function	PfD80
Up	PF3D7_0413100	1.994159	7.59E-08	4.3	4.7	4.8	6.6	6.3	6	erythrocyte membrane protein 1, PfEMP1	VAR
Up	PF3D7_0937800	1.968093	7.73E-28	6.7	6.79	7	8.9	8.8	9	erythrocyte membrane protein 1, PfEMP1	VAR
Up	PF3D7_0931300	1.960073	1.35E-13	5.3	5.75	6.1	7.8	7.4	8	conserved Plasmodium protein, unknown function	N/A
Up	PF3D7_0400400	1.954964	6.98E-49	8.6	8.99	9	11	11	11	erythrocyte membrane protein 1, PfEMP1	VAR
Up	PF3D7_0600200	1.940366	8.67E-32	7.6	8.31	8.1	10	10	10	erythrocyte membrane protein 1, PfEMP1	VAR

Up	PF3D7_0601300	1.919612	8.51E-02	2.6	3.17	3.6	4.2	4.1	5	exported protein family 1	EPF1
Up	PF3D7_0712000	1.905986	3.93E-41	8.6	9.03	8.6	11	11	11	erythrocyte membrane protein 1, PfEMP1	VAR
Up	PF3D7_1208400	1.872369	9.35E-02	3.3	2.32	4.5	4.8	4	6	amino acid transporter, putative	N/A
Up	PF3D7_0115600	1.868389	2.72E-03	3.5	3.58	4.2	5.4	5.2	5	rifin	RIF
Up	PF3D7_1206300	1.844264	2.76E-04	4	4.09	5.5	6.5	5.9	7	conserved Plasmodium protein, unknown function	N/A
Up	PF3D7_0500100	1.790898	4.06E-07	5	4.95	4.8	6.8	6.5	6	erythrocyte membrane protein 1, PfEMP1	VAR
Up	PF3D7_0532500	1.786956	1.14E-02	3.5	3.81	4.1	4.7	5.1	6	Plasmodium exported protein, unknown function	N/A
Up	PF3D7_0420700	1.766646	1.35E-19	6.5	7.17	7	8.7	8.5	9	erythrocyte membrane protein 1, PfEMP1	VAR
Up	PF3D7_1200600	1.764303	9.17E-07	10	10.4	9.6	12	12	12	erythrocyte membrane protein 1, PfEMP1	VAR2CSA
Up	PF3D7_1478600	1.746526	1.98E-25	9.7	9.96	11	12	12	12	EMP1-trafficking protein	PTP3
Up	PF3D7_0302200	1.698987	3.69E-02	3.5	3.46	3.7	4.6	5.1	5	cytoadherence linked asexual protein 3.2	CLAG3.2
Up	PF3D7_1341500	1.63796	2.97E-03	4.1	4.25	4	5.6	5.5	5	inner membrane complex suture component, putative	ISC1
Up	PF3D7_0302500	1.526477	1.01E-05	5	5.25	5.1	6.7	6.6	6	cytoadherence linked asexual protein 3.1	CLAG3.1
Up	PF3D7_0708100	1.466846	1.63E-08	6	5.93	5.7	7.4	7.2	7	DNA-directed RNA polymerases I, II, and III subunit RPABC5, putative	RPB10
Up	PF3D7_1372000	1.400024	1.58E-12	6.7	6.6	6.9	8.1	8.2	8	Plasmodium exported protein (PHISTa), unknown function	N/A
Up	PF3D7_0220300	1.398367	1.40E-09	6.3	6.07	6.5	7.7	7.7	8	Plasmodium exported protein, unknown function	N/A
Up	PF3D7_0301700	1.39351	1.08E-17	7.9	7.82	8.2	9.5	9.4	9	Plasmodium exported protein, unknown function	N/A
Up	PF3D7_0632700	1.369197	5.16E-03	4.6	4.91	4.4	5.9	6.1	6	rifin	RIF
Up	PF3D7_0707600	1.327291	1.19E-05	5.6	5.73	5.9	6.8	6.8	7	mediator of RNA polymerase II transcription subunit 10, putative	MED10
Up	PF3D7_0822000	1.299353	9.77E-05	5.2	5.52	5.6	6.8	6.7	6	ribosomal protein L4, mitochondrial, putative	N/A
Up	PF3D7_0708300	1.29286	4.53E-06	6.1	5.78	5.8	7.1	7	7	EKC/KEOPS complex subunit BUD32	BUD32

Up	PF3D7_0601700	1.288966	3.55E-04	5	5.61	5.9	6.6	6.7	7	Plasmodium exported protein (PHISTa), unknown function, pseudogene	N/A
Up	PF3D7_0707400	1.242555	1.56E-17	8.4	8.04	8.2	9.5	9.4	9	AAA family ATPase, putative	N/A
Up	PF3D7_1305900	1.236098	2.85E-03	4.9	4.86	5.5	6.3	6.3	6	conserved Plasmodium protein, unknown function	N/A
Up	PF3D7_0532200	1.228598	6.69E-03	4.6	5.46	5	6.4	6.3	6	Plasmodium exported protein (PHISTc), unknown function	N/A
Up	PF3D7_1253000	1.196722	9.58E-15	9	9.23	8.8	10	10	10	gametocyte erythrocyte cytosolic protein	GECO
Up	PF3D7_0503500	1.180067	5.31E-02	4.5	4.52	4.6	5.1	5.9	6	protein kinase, putative	N/A
Up	PF3D7_0220600	1.170418	3.64E-09	7.4	7.3	7.9	8.9	8.7	9	Plasmodium exported protein (hyp9), unknown function	N/A
Up	PF3D7_0532600	1.163862	7.35E-03	5.2	4.86	5.9	6.7	6.3	6	Plasmodium exported protein, unknown function	N/A
Up	PF3D7_0601900	1.148582	1.19E-17	10	10.7	11	12	12	12	conserved Plasmodium protein, unknown function	N/A
Up	PF3D7_0731400	1.141944	8.93E-03	4.8	5.25	5.1	6.1	6.1	6	serine/threonine protein kinase, FIKK family, pseudogene	FIKK7.2
Up	PF3D7_0719500	1.140075	1.83E-02	5	4.81	4.8	5.9	6	6	LEM3/CDC50 family protein, putative	CDC50A
Up	PF3D7_1131200	1.136557	7.26E-02	4.9	4.17	5	5.6	5.3	6	conserved Plasmodium protein, unknown function	N/A
Up	PF3D7_1477600	1.129644	4.49E-04	5.6	5.75	5.7	6.7	6.9	7	surface-associated interspersed protein 14.1 (SURFIN 14.1)	SURF14.1
Up	PF3D7_0220500	1.100229	5.54E-04	6	5.67	6.1	7.2	6.7	7	Plasmodium exported protein (hyp2), unknown function	N/A
Up	PF3D7_0221200	1.093332	1.78E-03	5.8	5.43	6	7	6.5	7	Plasmodium exported protein (hyp15), unknown function	N/A
Up	PF3D7_1035800	1.087066	1.75E-13	9.4	9.64	9.9	11	11	11	probable protein, unknown function	M712
Up	PF3D7_0830900	1.081565	1.30E-06	7.2	7.32	7.9	8.7	8.4	9	Plasmodium exported protein, unknown function	N/A
Up	PF3D7_0506900	1.070372	1.31E-05	6.3	6.79	6.6	7.8	7.7	7	rhomboid protease ROM4	ROM4
Up	PF3D7_0707900	1.065113	2.80E-15	9.2	9.08	9.1	10	10	10	ribosomal protein S8e, putative	N/A
Up	PF3D7_1149400	1.06084	8.55E-04	7	5.91	6.6	7.8	7.5	7	Plasmodium exported protein, unknown function	N/A
Up	PF3D7_0201800	1.040839	2.40E-17	10	10.3	10	12	11	11	knob associated heat shock protein 40	KAHsp40

Up	PF3D7_0707500	1.040631	4.35E-02	5.4	5.09	4.8	5.8	5.9	6	conserved Plasmodium protein, unknown function	N/A
Up	PF3D7_0708000	1.027636	1.93E-02	5.5	4.91	5.4	6.4	6.2	6	cytoskeleton associated protein, putative	N/A
Up	PF3D7_1350900	0.994085	4.08E-03	5.6	5.7	6	6.8	6.7	7	AP2 domain transcription factor AP2-O4, putative	ApiAP2
Up	PF3D7_1041300	0.984459	2.96E-05	6.6	7.1	7.2	8.1	7.8	8	erythrocyte membrane protein 1, PfEMP1	VAR
Up	PF3D7_1201100	0.98435	7.24E-05	6.7	6.52	6.9	7.6	7.9	8	RESA-like protein with PHIST and DnaJ domains	N/A
Up	PF3D7_1301300	0.976781	9.26E-02	4.5	5.13	5.3	5.6	5.8	6	Plasmodium exported protein (PHISTa-like), unknown function, pseudogene	N/A
Up	PF3D7_0201900	0.973421	1.09E-14	14	14	14	15	15	15	erythrocyte membrane protein 3	EMP3
Up	PF3D7_0708400	0.959654	6.63E-13	14	14	14	15	15	15	heat shock protein 90	HSP90
Up	PF3D7_0908000	0.957157	7.95E-02	5	4.91	4.9	5.7	5.7	6	plasma membrane protein 1, putative	PMP1
Up	PF3D7_1401500	0.947745	1.90E-02	5.7	5.52	5.4	6.2	6.5	7	esterase, putative	N/A
Up	PF3D7_1252200	0.945008	3.51E-04	6.6	7.29	7.3	7.9	8.3	8	chitinase	CHT1
Up	PF3D7_1001000	0.928623	2.42E-02	6.9	6.02	7.3	7.4	7.3	8	chondroitin sulfate A ligand	CSA-L
Up	PF3D7_1007000	0.927915	1.17E-02	5.8	5.64	5.8	6.4	6.6	7	transmembrane protein 147, putative	TMEM147
Up	PF3D7_0617400	0.926744	2.80E-06	7.5	7.8	7.4	8.7	8.4	8	erythrocyte membrane protein 1, PfEMP1	VAR
Up	PF3D7_0412700	0.921539	7.93E-11	9	9.33	9.1	10	10	10	erythrocyte membrane protein 1, PfEMP1	VAR
Up	PF3D7_0702500	0.916019	6.16E-15	11	10.9	11	12	12	12	Plasmodium exported protein, unknown function	N/A
Up	PF3D7_0320500	0.911296	8.66E-03	6.2	6.63	7.2	7.3	7.6	8	nicotinamidase, putative	Nico
Up	PF3D7_1301400	0.898807	2.08E-12	12	12.1	12	13	13	13	Plasmodium exported protein (hyp12), unknown function	HYP12
Up	PF3D7_1477500	0.888265	1.09E-10	11	11	11	12	12	12	Plasmodium exported protein (PHISTb), unknown function	N/A
Up	PF3D7_0113200	0.885316	1.62E-03	7.5	6.51	7	8	7.9	8	Plasmodium exported protein, unknown function	N/A
Up	PF3D7_0601200	0.880676	5.57E-02	6.3	5.49	5.4	6.6	6.8	6	Pfmc-2TM Maurer's cleft two transmembrane protein	MC-2TM
Up	PF3D7_1317400	0.874927	1.33E-02	6.1	5.81	6.5	7.2	6.9	7	zinc finger protein, putative	N/A

Up	PF3D7_1476200	0.869257	4.54E-10	9.4	9.65	9.8	11	10	10	Plasmodium exported protein (PHISTb), unknown function	N/A
Up	PF3D7_0719400	0.86092	6.45E-02	6	5.32	5.4	6.2	6.6	6	conserved Plasmodium protein, unknown function	N/A
Up	PF3D7_0817300	0.857669	6.06E-07	9.2	9.09	9.6	10	10	10	conserved Plasmodium protein, unknown function	N/A
Up	PF3D7_0702400	0.854893	9.78E-10	11	11.2	11	12	12	12	small exported membrane protein 1	SEMP1
Up	PF3D7_1219300	0.846331	5.62E-05	7.3	7.81	7.5	8.6	8.3	8	erythrocyte membrane protein 1, PfEMP1	VAR
Up	PF3D7_1246800	0.834262	2.47E-03	6.7	6.32	6.8	7.4	7.6	7	signal recognition particle receptor subunit beta, putative	SRPRB
Up	PF3D7_1252300	0.827996	6.92E-11	11	11	11	12	12	12	conserved Plasmodium protein, unknown function	N/A
Up	PF3D7_0726200	0.826813	2.05E-06	8.3	8.25	8.7	9.1	9.4	9	serine/threonine protein kinase, FIKK family	FIKK7.1
Up	PF3D7_0830800	0.819542	1.51E-03	7.7	7.77	8.5	8.7	8.8	9	surface-associated interspersed protein 8.2 (SURFIN 8.2)	SURF8.2
Up	PF3D7_0911400	0.819117	1.72E-02	6.4	6.36	6.6	6.9	7.1	8	conserved Plasmodium protein, unknown function	N/A
Up	PF3D7_0806100	0.815334	5.48E-02	5.4	5.83	5.9	6.4	6.4	7	conserved Plasmodium protein, unknown function	N/A
Up	PF3D7_0707700	0.802777	1.00E-10	10	10.6	10	11	11	11	E3 ubiquitin-protein ligase, putative	N/A
Up	PF3D7_0113800	0.796789	8.09E-03	6.6	6.86	7.4	7.6	8	8	DBL containing protein, unknown function	N/A
Up	PF3D7_0936200	0.789866	1.18E-03	6.9	6.78	7	7.8	7.5	8	Plasmodium exported protein (hyp11), unknown function	N/A
Up	PF3D7_0807200	0.785179	5.52E-05	7.6	7.94	8.1	8.8	8.6	9	conserved Plasmodium membrane protein, unknown function	N/A
Up	PF3D7_1317200	0.781655	3.51E-04	7.9	7.57	8.2	8.5	8.8	9	AP2 domain transcription factor AP2-FG, putative	ApiAP2
Up	PF3D7_1317300	0.777739	1.83E-04	7.5	7.45	7.4	8.4	8.1	8	RNA-binding protein, putative	N/A
Up	PF3D7_1203000	0.775205	5.06E-02	5.9	5.75	5.8	6.6	6.7	6	origin recognition complex subunit 1	ORC1
Up	PF3D7_0731300	0.773214	3.49E-07	9.8	10.1	10	11	11	11	Plasmodium exported protein (PHISTb), unknown function	PfG174
Up	PF3D7_0201600	0.771349	2.08E-09	10	10.2	10	11	11	11	PHISTb domain-containing RESA-like protein 1	RLP1
Up	PF3D7_0730900	0.770754	2.22E-08	12	12.4	13	13	13	13	EMP1-trafficking protein	PTP4

Up	PF3D7_0708500	0.77029	2.34E-07	9	9.15	9	9.9	9.9	10	heat shock protein 86 family protein	N/A
Up	PF3D7_0418400	0.770208	7.93E-02	5.7	5.7	6.2	6.9	6.4	7	LSM domain-containing protein, putative	N/A
Up	PF3D7_1445500	0.769677	3.89E-03	7.1	6.93	7.2	7.8	7.5	8	conserved Plasmodium protein, unknown function	N/A
Up	PF3D7_1403900	0.737852	2.09E-02	6.6	6.17	6.5	7.3	7.1	7	serine/threonine protein phosphatase CPPED1, putative	CPPED1
Up	PF3D7_0532400	0.736213	4.76E-07	11	11.2	11	12	12	12	lysine-rich membrane-associated PHISTb protein	LyMP
Up	PF3D7_0902300	0.732054	8.72E-10	9.8	10	9.9	11	11	11	serine/threonine protein kinase, FIKK family	FIKK9.4
Up	PF3D7_0221700	0.726448	2.56E-04	7.4	7.57	7.5	8.1	8.2	8	Plasmodium exported protein, unknown function	N/A
Up	PF3D7_0532300	0.715663	2.09E-10	13	13.2	13	14	14	14	Plasmodium exported protein (PHISTb), unknown function	N/A
Up	PF3D7_0832200	0.708862	1.31E-05	8.6	8.37	8.8	9.3	9.2	9	Plasmodium exported protein (PHISTa-like), unknown function	N/A
Up	PF3D7_0219700	0.704767	4.91E-02	6.3	6.25	6.1	7	6.7	7	Plasmodium exported protein (PHISTc), unknown function	GEXP20
Up	PF3D7_1442400	0.688992	2.68E-02	7.1	6.89	7.5	7.5	8	8	protein KIC9	KIC9
Up	PF3D7_1025900	0.687677	4.15E-02	6.3	6.46	6.9	7.3	7.4	7	conserved protein, unknown function	N/A
Up	PF3D7_0501200	0.681309	1.41E-04	9.8	9.17	9.4	10	10	10	parasite-infected erythrocyte surface protein	PIESP2
Up	PF3D7_1240300	0.673505	7.99E-04	7.7	8.19	7.9	8.7	8.7	9	erythrocyte membrane protein 1, PfEMP1	VAR
Up	PF3D7_1002200	0.664533	9.98E-03	8	7.38	7.8	8.4	8.6	8	tryptophan-rich antigen 3	PArt
Up	PF3D7_1301800	0.657581	3.00E-02	6.8	7.11	7.4	7.8	7.9	7	surface-associated interspersed protein 13.1 (SURFIN 13.1), pseudogene	SURF13.1
Up	PF3D7_1467900	0.657166	4.35E-02	6.7	6.34	7	7.3	7.4	7	rab GTPase activator, putative	N/A
Up	PF3D7_1033200	0.656535	4.36E-05	9.1	9.34	9.3	10	10	10	early transcribed membrane protein 10.2	ETRAMP10.2
Up	PF3D7_1026000	0.643693	7.97E-07	9.7	9.8	10	11	10	10	conserved Plasmodium protein, unknown function	N/A
Up	PF3D7_0417800	0.633123	5.22E-02	6.6	6.37	6.7	7.1	7.2	7	cdc2-related protein kinase 1	CRK1
Up	PF3D7_1021700	0.630747	3.05E-02	8.6	7.74	8.7	8.8	9.2	9	VPS13 domain-containing protein, putative	N/A

Up	PF3D7_0420900	0.625847	4.23E-02	6.9	7.27	6.9	7.8	7.8	7	erythrocyte membrane protein 1, PfEMP1	VAR
Up	PF3D7_0501000	0.618677	1.09E-06	11	10.7	11	11	11	11	Plasmodium exported protein, unknown function	N/A
Up	PF3D7_1107800	0.612288	2.90E-04	11	11.1	11	12	12	12	AP2 domain transcription factor, putative	ApiAP2
Up	PF3D7_0936400	0.607264	5.46E-03	7.8	8.04	8.3	8.8	8.5	9	ring-exported protein 4	REX4
Up	PF3D7_0713400	0.605062	4.34E-02	6.7	6.78	6.7	7.3	7.3	7	GPCR-like receptor SR25	SR25
Up	PF3D7_1412500	0.599944	1.59E-03	8.9	9.06	9.2	9.8	9.8	9	actin II	ACT2
Up	PF3D7_0206300	0.598886	5.26E-03	11	10.5	11	11	12	11	pentafunctional AROM polypeptide, putative, pseudogene	AROM
Up	PF3D7_1129900	0.58665	9.97E-02	6.6	6.52	7	7.2	7.2	7	major facilitator superfamily-related transporter, putative	MFR5
Down	PF3D7_0114700	-7.85245	1.90E-08	5.3	5.32	5.9	2	2	2	rifin	RIF
Down	PF3D7_1240800	-6.9515	4.06E-06	4.8	4.52	4.9	2	2	2	Plasmodium RNA of unknown function RUF6	N/A
Down	PF3D7_1041100	-5.86354	1.35E-03	3.8	4.17	3.6	2	2	2	rifin	RIF
Down	PF3D7_1240900	-5.49552	0.00E+00	15	15.1	15	9.3	9.5	9	erythrocyte membrane protein 1, PfEMP1	VAR
Down	PF3D7_0223400	-5.45676	1.51E-03	4.4	4.25	4.2	2	2.3	2	rifin	RIF
Down	PF3D7_0114600	-5.32316	1.19E-02	3.5	3.58	3.5	2	2	2	stevor, pseudogene	N/A
Down	PF3D7_0425400	-5.32262	6.49E-167	10	10.3	10	5.4	5	5	Plasmodium exported protein (PHISTa), unknown function	N/A
Down	PF3D7_1479200	-5.18717	4.55E-03	3.8	4.17	4.2	2	2.3	2	Plasmodium exported protein (PHISTa), unknown function	N/A
Down	PF3D7_1240700	-5.0393	1.54E-15	6.5	6.6	6.4	3.3	2	3	rifin, pseudogene	RIF
Down	PF3D7_0631900	-4.96212	1.59E-03	5.5	3.7	3.9	2	2.3	2	stevor	N/A
Down	PF3D7_0324200	-4.95132	4.19E-02	3.3	3.32	3.2	2	2	2	exported protein family 3	EPF3
Down	PF3D7_0223100	-4.86373	5.59E-02	3.3	3.46	2.8	2	2	2	rifin	RIF
Down	PF3D7_0900600	-4.63511	2.48E-06	5.2	5.28	5.1	2.8	2.3	2	rifin	RIF
Down	PF3D7_0500400	-4.48677	4.47E-02	3.3	3.58	3.8	2	2.3	2	rifin	RIF
Down	PF3D7_0937300	-4.32748	1.27E-02	3.8	4.52	3.9	2	2.6	2	rifin	RIF
Down	PF3D7_1300700	-3.96046	4.76E-06	5	5.09	5.3	2.3	2.6	3	rifin	RIF

Down	PF3D7_0532800	-3.83044	2.00E-05	4.7	5.04	5.2	2.3	2.8	3	stevor, pseudogene	N/A
Down	PF3D7_1478700	-3.7802	1.55E-02	4.8	3.81	3.5	2	2.3	3	Plasmodium exported protein, unknown function, pseudogene	N/A
Down	PF3D7_0400700	-3.74299	4.01E-05	4.8	5.13	4.9	2.3	2.8	3	rifin	RIF
Down	PF3D7_0808800	-3.70701	1.44E-02	4.3	3.58	4.3	2.3	2.3	2	rifin	RIF
Down	PF3D7_0900200	-3.68789	1.12E-09	5.6	5.83	5.9	2.8	3.2	3	rifin	RIF
Down	PF3D7_0324100	-3.6695	7.92E-05	5.1	4.7	4.8	2.3	2.8	3	Pfmc-2TM Maurer's cleft two transmembrane protein	MC-2TM
Down	PF3D7_0424800	-3.62906	6.65E-22	6.9	7.24	7	4.1	3.5	4	Plasmodium exported protein (PHISTb), unknown function	N/A
Down	PF3D7_0632300	-3.5504	3.24E-02	4	4.39	3.3	2	2.6	2	rifin	RIF
Down	PF3D7_1373400	-3.4186	8.73E-11	5.9	5.93	6.2	3	3.5	3	rifin	RIF
Down	PF3D7_0401900	-3.20379	1.21E-66	9.5	9.71	9.2	6.4	6.4	6	acyl-CoA synthetase	ACS6
Down	PF3D7_1306200	-3.05233	9.26E-02	4.7	3.58	2.8	2	2.6	3	conserved protein, unknown function	N/A
Down	PF3D7_0424900	-2.9452	3.18E-108	11	11.1	11	8.1	7.9	8	Plasmodium exported protein (PHISTa), unknown function	N/A
Down	PF3D7_0324300	-2.89369	1.99E-11	6.3	6.27	6.3	3.9	3.5	4	exported protein family 4	EPF4
Down	PF3D7_0223500	-2.89318	4.34E-02	3.7	4.32	4	2.3	2.8	2	erythrocyte membrane protein 1, PfEMP1	VAR
Down	PF3D7_0631500	-2.83582	6.69E-09	6.3	6.07	6	3.8	2.8	4	exported protein family 3	EPF3
Down	PF3D7_0922400	-2.78012	4.76E-02	4.6	4	3.6	2	2.6	3	para-aminobenzoic acid synthetase	pBAS
Down	PF3D7_0101100	-2.74383	1.77E-06	5.9	5.46	5.6	3	3.2	4	exported protein family 4	EPF4
Down	PF3D7_0425100	-2.73653	1.18E-06	5.4	5.83	5.9	3.3	3	4	Plasmodium exported protein (hyp6), unknown function	N/A
Down	PF3D7_0114500	-2.70667	6.42E-12	6.5	6.3	7.2	4.4	4.1	4	Plasmodium exported protein (hyp10), unknown function	N/A
Down	PF3D7_0532900	-2.69028	2.85E-04	5	5.28	5	3	3.6	3	rifin	RIF
Down	PF3D7_1479000	-2.66952	2.96E-05	5.8	5.36	4.9	3.3	3.2	3	acyl-CoA synthetase	ACS1a
Down	PF3D7_1041200	-2.63009	7.12E-02	4.3	4	3.8	3	2.6	2	rifin	RIF
Down	PF3D7_0222300	-2.62829	3.30E-09	6.1	6.11	6.2	3.8	3.6	4	exported protein family 4, pseudogene	EPF4
Down	PF3D7_0808900	-2.53753	2.30E-02	5	4.17	3.8	2.8	2.6	3	rifin	RIF

Down	PF3D7_0533100	-2.53033	6.96E-28	8.1	8.27	7.9	5.8	5.9	5	erythrocyte membrane protein 1 (PfEMP1), pseudogene	VAR1CSA
Down	PF3D7_0102300	-2.48876	2.09E-02	5.6	3.58	4.1	3	3.2	3	Plasmodium exported protein, unknown function	N/A
Down	PF3D7_0613400	-2.43156	7.07E-02	5.6	3.46	3.5	2.3	3.5	3	50S ribosomal protein L18, apicoplast, putative	RPL18
Down	PF3D7_0409500	-2.38103	5.59E-02	5.5	3.46	3.7	3	3.2	3	NAD(P)-binding protein, putative	N/A
Down	PF3D7_1219400	-2.19659	5.74E-45	9.2	9.53	9.5	7.3	7.2	7	erythrocyte membrane protein 1 (PfEMP1), pseudogene	VAR
Down	PF3D7_0832000	-2.08852	5.49E-06	6.5	5.75	6	4.2	4.7	4	stevor	N/A
Down	PF3D7_0425200	-2.05542	1.91E-07	6.4	6.41	6.4	4.2	4.2	5	Plasmodium exported protein (hyp15), unknown function	N/A
Down	PF3D7_1432700	-2.03278	6.03E-02	5.2	4	4	3	3.2	3	protein-L-isoaspartate(D-aspartate) O-methyltransferase, putative	N/A
Down	PF3D7_0509100	-1.99847	4.16E-03	6.8	4.86	5	4.1	4.4	4	structural maintenance of chromosomes protein 4, putative	SMC4
Down	PF3D7_0701600	-1.99596	2.53E-03	6.2	4.58	5.4	3.8	3.9	4	Pfmc-2TM Maurer's cleft two transmembrane protein	MC-2TM
Down	PF3D7_0718100	-1.92495	9.96E-02	5.6	4.09	3.8	3	3.9	3	exported serine/threonine protein kinase	EST
Down	PF3D7_0425600	-1.8887	5.62E-02	4.6	4.17	4.8	3.5	3.3	3	rifin, pseudogene	RIF
Down	PF3D7_0902800	-1.88722	4.88E-02	6.3	4.25	4.1	3.5	3.9	4	serine repeat antigen 9	SERA9
Down	PF3D7_0711700	-1.87559	5.74E-57	12	12.5	12	10	11	10	erythrocyte membrane protein 1, PfEMP1	VAR
Down	PF3D7_1240600	-1.8106	3.23E-41	13	13.5	13	12	12	11	erythrocyte membrane protein 1, PfEMP1	VAR
Down	PF3D7_1228300	-1.78232	5.71E-02	5.9	4.75	3.8	3.8	3.7	3	NIMA related kinase 1	NEK1
Down	PF3D7_1476600	-1.75317	8.82E-12	7.9	8.17	8.6	6.8	6.8	6	Plasmodium exported protein, unknown function	N/A
Down	PF3D7_0613800	-1.74143	2.24E-02	6.6	4.32	5.2	4.2	4.1	4	AP2 domain transcription factor, putative	ApiAP2
Down	PF3D7_0705400	-1.60411	7.77E-02	6.5	4.52	4.5	3.9	4.6	4	DNA replication licensing factor MCM7	MCM7
Down	PF3D7_1366500	-1.59253	3.73E-02	6.5	4.81	4.8	4.1	4.3	4	nucleoside diphosphate kinase	NDK

Down	PF3D7_0934400	-1.55131	3.00E-02	5.9	4.7	5	3.9	4.1	4	AP2 domain transcription factor, putative	ApiAP2
Down	PF3D7_1349600	-1.54265	5.52E-02	5.6	4.86	4.5	3.8	4	4	conserved Plasmodium protein, unknown function	N/A
Down	PF3D7_0918500	-1.48295	1.64E-02	6	5	5.5	4.5	4.4	4	telomerase RNA	TR
Down	PF3D7_0425500	-1.4632	2.11E-02	5.9	4.91	5.4	4.2	4.1	4	stevor	N/A
Down	PF3D7_0316300	-1.43904	8.56E-02	6.3	4.52	4.8	4.2	4.3	4	inorganic pyrophosphatase	PPase
Down	PF3D7_0416300	-1.42682	9.66E-02	5.9	4.46	4.8	4.2	4.1	4	DNA helicase MCM9, putative	MCM9
Down	PF3D7_1239200	-1.42033	1.72E-02	7.3	5.64	5.5	5.2	5	5	AP2 domain transcription factor, putative	ApiAP2
Down	PF3D7_1127100	-1.35583	5.52E-02	7	5.13	5.4	5.2	4.8	5	deoxyuridine 5'-triphosphate nucleotidohydrolase	dUTPase
Down	PF3D7_1428400	-1.34814	7.07E-02	4.9	5.28	5.7	3.5	4.6	4	WD and tetratricopeptide repeats protein 1, putative	WDTC1
Down	PF3D7_1477000	-1.32607	5.64E-20	11	10.9	11	9.7	9.5	10	Plasmodium exported protein (hyp17), unknown function, pseudogene	N/A
Down	PF3D7_1458300	-1.2794	5.59E-02	5.7	4.91	5.5	4.6	4.2	4	alpha/beta hydrolase, putative	N/A
Down	PF3D7_0526600	-1.19513	4.35E-02	7.3	5.78	5.9	5.3	5.8	5	conserved Plasmodium protein, unknown function	N/A
Down	PF3D7_0829800	-1.19417	7.37E-04	7	6.58	6.5	5.6	5.8	5	non-coding RNA	N/A
Down	PF3D7_0829900	-1.05625	7.73E-03	6.8	6.15	6.5	5.7	5.5	5	non-coding RNA	N/A
Down	MAL_RNA_14	-1.04158	6.17E-03	7	6.27	6.6	5.6	5.6	6		
Down	PF3D7_0110700	-0.96554	1.52E-02	7.3	6.82	6.5	6.3	5.9	6	chromatin assembly factor 1 subunit C, putative	CAF1C
Down	PF3D7_1370500	-0.96339	6.84E-02	6.1	5.78	5.8	4.9	4.9	5	non-coding RNA	N/A
Down	PF3D7_1318000	-0.91152	1.97E-03	6.9	7.15	7.1	6.1	6.1	6	conserved protein, unknown function	N/A
Down	PF3D7_0404400	-0.83397	2.63E-02	6.5	6.52	6.8	5.8	5.8	6	6-cysteine protein P36	P36
Down	PF3D7_1307600	-0.8288	2.07E-02	7	6.94	6.6	5.8	6.2	6	DNA-directed RNA polymerase subunit alpha, putative	rpoA
Down	PF3D7_1443900	-0.78676	6.03E-03	8	7.55	7.3	6.9	7	7	heat shock protein 90, putative	HSP90
Down	PF3D7_1343300	-0.7693	2.13E-02	7.8	7.21	7.1	6.6	6.8	6	CDT1-like protein, putative	N/A
Down	PF3D7_1427900	-0.66147	2.10E-03	9.1	8.77	8.6	8.3	8	8	leucine-rich repeat protein	N/A

Down	PF3D7_1457200	-0.62859	4.61E-02	8.6	8.21	8	8.1	7.5	7	thioredoxin 1	TRX1

Table S3H. Differential expression between PfDNMT2 overexpression and wildtype parasite at trophozoite stage

R: replicate, WT: 3D7 wild type, OE: PfDNMT2 overexpression, Adj.Pval: adjusted P value

columns G-L is EdgeR log2 (counts per million + count)

Change pattern	Gene ID	log2 Fold Change	Adj.Pval	WT R1	WT R2	WT R3	OE R1	OE R2	OE R3	Product Description	Name or Symbol
Up	PF3D7_1150400	7.0617	1.27E-13	2.3	2.6	2	7.15	7.5	6.5	erythrocyte membrane protein 1, PfEMP1	VAR
Up	PF3D7_0221400	6.85978	5.82E-22	3.2	2	2	7.66	7.6	7.6	stevor	N/A
Up	PF3D7_0402600	6.07607	2.07E-06	2.3	2.3	2	5.7	5.8	5.4	stevor	N/A
Up	PF3D7_1400200	6.045	1.68E-04	2	2	2.3	5	4.6	4.7	rifin	RIF
Up	PF3D7_0412400	5.60064	1.56E-30	4.1	3.8	3.3	8.78	9.4	8.3	erythrocyte membrane protein 1, PfEMP1	VAR
Up	PF3D7_1150300	5.17885	2.67E-02	2	2	2	3	3.5	3.7	rifin	RIF
Up	PF3D7_0221300	5.105	3.25E-02	2	2	2	3.58	3.2	3.3	Plasmodium exported protein, unknown function, pseudogene	N/A
Up	PF3D7_0727300	4.99781	7.82E-27	7.4	7.7	7.8	12.7	13	12	DNA (cytosine-5)-methyltransferase	DNMT
Up	PF3D7_0800300	4.69971	6.23E-42	3.6	3.3	4.1	7.81	8.1	7.7	erythrocyte membrane protein 1, PfEMP1	VAR
Up	PF3D7_0425900	4.52289	1.07E-02	2.3	2	2.3	3.58	3.8	5	rifin	RIF
Up	PF3D7_0221500	4.41315	2.92E-26	3.3	3.3	3.5	7.27	7.2	6.9	Pfmc-2TM Maurer's cleft two transmembrane protein, pseudogene	N/A
Up	PF3D7_0221100	4.12863	3.08E-02	2.3	2	2.3	3.81	3.9	4.2	Plasmodium exported protein, unknown function, pseudogene	N/A
Up	PF3D7_0708600	4.11762	3.56E-41	4.2	4.2	3.8	7.59	8	7.8	inner membrane complex protein 1d, putative	IMC1d
Up	PF3D7_0632500	4.06622	7.05E-61	5.2	4.7	4.6	8.71	9	8.3	erythrocyte membrane protein 1, PfEMP1	VAR
Up	PF3D7_1300300	3.93789	6.28E-03	2.6	2.3	2	4.32	4.2	4.2	erythrocyte membrane protein 1, PfEMP1	VAR
Up	PF3D7_1200500	3.89009	8.71E-06	2.8	2.3	2.6	5.09	5.2	4.9	rifin	RIF
Up	PF3D7_0632700	3.58874	1.02E-18	4.2	3.5	3.3	6.66	6.5	7.2	rifin	RIF
Up	PF3D7_0425800	3.53534	1.23E-03	2.8	2.3	2.3	4.32	4.6	4.6	erythrocyte membrane protein 1, PfEMP1	VAR
Up	PF3D7_0302300	3.49565	1.72E-20	4	4.3	3.5	7.33	7.3	6.5	erythrocyte membrane protein 1 (PfEMP1), pseudogene	N/A
Up	PF3D7_0413400	2.82134	3.45E-05	3.6	3	2.6	5.36	5.2	5.1	erythrocyte membrane protein 1 (PfEMP1), exon 1, pseudogene	VAR
Up	PF3D7_1200600	2.74277	1.88E-22	5.6	5.3	4.5	8.01	8	7.3	erythrocyte membrane protein 1, PfEMP1	VAR2CSA

Up	PF3D7_1400100	2.66955	1.76E-18	5.1	4.3	4.4	7.3	7.1	6.9	erythrocyte membrane protein 1 (PfEMP1), pseudogene	N/A
Up	PF3D7_0300100	2.60996	2.19E-19	4.7	4.4	4.9	7.2	7.1	7	erythrocyte membrane protein 1, PfEMP1	VAR
Up	PF3D7_0221800	2.49414	6.36E-03	3	2.6	3.2	4.7	4.6	4.6	hypothetical protein	N/A
Up	PF3D7_0800200	2.28892	2.86E-11	4.9	3.7	5	6.67	6.8	6.7	erythrocyte membrane protein 1, PfEMP1	VAR
Up	PF3D7_0115700	2.21235	4.35E-02	3	2.6	3.2	4.46	4.6	4.1	erythrocyte membrane protein 1, PfEMP1	VAR
Up	PF3D7_0221700	1.86062	1.30E-07	4.6	5.2	4.5	6.27	6.7	6.4	Plasmodium exported protein, unknown function	N/A
Up	PF3D7_0320400	1.71284	2.64E-02	3.6	3.9	3.3	4.64	5.1	5.1	oocyst capsule protein Cap380	Cap380
Up	PF3D7_1200400	1.47316	1.66E-05	4.8	5.2	5.2	6.27	6.6	6.4	erythrocyte membrane protein 1, PfEMP1	VAR
Up	PF3D7_0708300	1.42019	6.62E-10	6.1	6.1	5.8	7.4	7.5	7.2	EKC/KEOPS complex subunit BUD32	BUD32
Up	PF3D7_0707500	1.34274	4.72E-08	6.1	5.8	6	7.04	7.4	7.3	conserved Plasmodium protein, unknown function	N/A
Up	PF3D7_1000100	1.3282	5.35E-04	4.7	5.1	5	6.25	6.2	6.1	erythrocyte membrane protein 1, PfEMP1	VAR
Up	PF3D7_1146800	1.30802	3.50E-04	5	5.1	5.1	6.21	6.2	6.4	conserved Plasmodium protein, unknown function	N/A
Up	PF3D7_1141100	1.23672	8.81E-04	4.9	5.4	5.1	6.34	6.4	6.2	conserved Plasmodium protein, unknown function	N/A
Up	PF3D7_0707400	1.2256	4.15E-38	9.6	9.6	9.5	10.7	11	11	AAA family ATPase, putative	N/A
Up	PF3D7_0221200	1.21977	2.21E-11	7.2	7.6	7	8.56	8.5	8.3	Plasmodium exported protein (hyp15), unknown function	N/A
Up	PF3D7_0713100	1.1746	6.06E-02	4.3	5	4.2	5.67	5.8	5.2	Pfmc-2TM Maurer's cleft two transmembrane protein	MC-2TM
Up	PF3D7_0708000	1.17268	6.04E-21	8.7	8.7	8.4	9.62	9.7	9.9	cytoskeleton associated protein, putative	N/A
Up	PF3D7_0707800	1.10601	6.31E-29	9.9	9.9	10	11	11	11	RAP protein, putative	N/A
Up	PF3D7_1148200	1.1039	1.81E-07	7.6	7.4	7.1	8.7	8.6	8.1	non-coding RNA	N/A
Up	PF3D7_0707600	1.09645	5.44E-08	7.1	6.8	6.9	7.94	7.8	8.3	mediator of RNA polymerase II transcription subunit 10, putative	MED10
Up	PF3D7_1011600	1.06036	4.83E-02	5.2	4.6	5	5.95	6.2	5.5	heptatricopeptide repeat-containing protein, putative	N/A
Up	PF3D7_0707700	1.0492	7.03E-28	10	10	10	11.3	11	11	E3 ubiquitin-protein ligase, putative	N/A
Up	PF3D7_0707900	0.99984	1.63E-20	8.7	8.8	8.8	9.76	9.8	9.7	ribosomal protein S8e, putative	N/A
Up	PF3D7_0632800	0.98348	7.65E-03	5.6	5.4	5.9	6.6	6.4	6.7	erythrocyte membrane protein 1, PfEMP1	VAR
Up	PF3D7_1370500	0.94965	2.51E-05	7.3	7.3	6.9	8.29	8.3	7.7	non-coding RNA	N/A

Up	PF3D7_0529200	0.92701	1.36E-02	5.6	5.6	5.6	6.48	6.3	6.6	sugar transporter, putative	N/A
Up	PF3D7_1253000	0.91946	1.44E-20	9.4	9.3	9.5	10.3	10	10	gametocyte erythrocyte cytosolic protein	GECO
Up	PF3D7_1240400	0.91749	9.92E-02	5.2	5.5	5.6	6.46	6.7	5.5	erythrocyte membrane protein 1, PfEMP1	VAR
Up	PF3D7_0708100	0.90866	4.51E-04	6.7	6.7	6.2	7.6	7.3	7.4	DNA-directed RNA polymerases I, II, and III subunit RPABC5, putative	RPB10
Up	PF3D7_1370800	0.88645	1.08E-03	6.3	6.4	6.2	7.3	7.2	6.9	non-coding RNA	N/A
Up	PF3D7_1370900	0.86641	4.51E-05	7.3	7	6.9	8.09	7.9	7.7	non-coding RNA	N/A
Up	PF3D7_0708400	0.80003	2.06E-18	15	15	15	15.6	16	15	heat shock protein 90	HSP90
Up	PF3D7_0708500	0.7968	1.17E-15	9.3	9.4	9.3	10.1	10	10	heat shock protein 86 family protein	N/A
Up	PF3D7_1453600	0.70012	4.18E-02	6.1	6.5	6.2	6.91	7.2	6.9	RAP protein, putative	N/A
Up	PF3D7_1240300	0.69106	2.70E-04	7.5	7.8	8	8.61	8.5	8.3	erythrocyte membrane protein 1, PfEMP1	VAR
Down	PF3D7_0114600	-6.555	1.63E-05	5	5.6	5	2	2	2.3	stevor, pseudogene	N/A
Down	PF3D7_0400700	-6.12548	1.25E-04	4.5	4.9	5.1	2	2.3	2	rifin	RIF
Down	PF3D7_0114300	-5.74806	1.29E-19	7	7.3	6.7	2.32	3	2.6	exported protein family 4, pseudogene	EPF4
Down	PF3D7_0114500	-5.25069	1.88E-02	3.5	3.3	3.6	2	2	2	Plasmodium exported protein (hyp10), unknown function	N/A
Down	PF3D7_1401000	-5.05967	4.82E-12	6	6.4	5.9	3	2	2.6	GBPH protein	GBPH
Down	PF3D7_0532800	-4.8421	3.20E-05	5.1	5.3	4.5	2.32	2	2.6	stevor, pseudogene	N/A
Down	PF3D7_0223500	-4.75548	4.86E-05	5.2	4.6	5	2.32	2	2.6	erythrocyte membrane protein 1, PfEMP1	VAR
Down	PF3D7_0732900	-4.59689	4.70E-02	4	3.2	3.7	2	2.3	2	rifin	RIF
Down	PF3D7_1478700	-4.44931	2.56E-16	6.6	6.8	6	2.81	3.2	3	Plasmodium exported protein, unknown function, pseudogene	N/A
Down	PF3D7_0114200	-4.36998	6.37E-04	4.8	4.7	4.4	2	2	2.8	exported protein family 3	EPF3
Down	PF3D7_1476600	-4.34263	9.20E-145	10	9.9	9.8	5.61	6	5.3	Plasmodium exported protein, unknown function	N/A
Down	PF3D7_0324800	-4.18055	4.27E-09	5.6	5.3	6.1	2.58	3.2	2.3	rifin	RIF
Down	PF3D7_0631900	-4.1163	2.24E-13	6.2	6.5	5.8	2.58	3	3.3	stevor	N/A
Down	PF3D7_0808900	-4.10913	1.80E-04	4.9	4.4	4.9	2.32	2.3	2.6	rifin	RIF
Down	PF3D7_1041200	-4.08813	7.73E-08	5.2	5.3	5.7	2.81	2.6	2.6	rifin	RIF
Down	PF3D7_0222100	-3.95487	5.55E-02	4.2	3.9	3.3	2.32	2	2.3	Pfmc-2TM Maurer's cleft two transmembrane protein	MC-2TM

Down	PF3D7_0302200	-3.93612	3.86E-41	8.4	7.7	8.7	4.7	4.2	4.9	cytoadherence linked asexual protein 3.2	CLAG3.2
Down	PF3D7_0900100	-3.84623	1.35E-07	5.5	5.5	5.2	2.81	2.8	2.6	erythrocyte membrane protein 1, PfEMP1	VAR
Down	PF3D7_1478400	-3.84203	9.64E-03	4.3	4.2	4	2.32	2.3	2.3	Plasmodium exported protein, unknown function, pseudogene	N/A
Down	PF3D7_1373500	-3.65815	7.43E-17	6.6	6.2	6.7	3.46	3.2	3.7	erythrocyte membrane protein 1, PfEMP1	VAR
Down	PF3D7_0400300	-3.65273	1.33E-05	4.9	4.9	5.4	2.81	2.6	2.6	rifin	RIF
Down	PF3D7_0532900	-3.62536	3.05E-02	3.5	4.2	4.3	2	2.6	2.3	rifin	RIF
Down	PF3D7_1477200	-3.62519	3.45E-02	4.6	3.9	3.3	2.32	2.6	2	Plasmodium exported protein (hyp15), unknown function	N/A
Down	PF3D7_0425500	-3.51163	2.47E-07	5.4	5.4	5.4	3.17	2.6	2.8	stevor	N/A
Down	PF3D7_0114100	-3.49475	1.10E-10	5.7	6.1	5.9	3.46	3	3	Pfmc-2TM Maurer's cleft two transmembrane protein	MC-2TM
Down	PF3D7_0324100	-3.48756	1.59E-13	6.3	6.4	6	3.32	3.5	3.3	Pfmc-2TM Maurer's cleft two transmembrane protein	MC-2TM
Down	PF3D7_1101700	-3.27374	9.12E-02	4.1	3.8	3.3	2.32	2.3	2.3	Pfmc-2TM Maurer's cleft two transmembrane protein	MC-2TM
Down	PF3D7_0533100	-2.88275	5.80E-36	8.2	8.1	8.7	5.43	5.9	5.3	erythrocyte membrane protein 1 (PfEMP1), pseudogene	VAR1CSA
Down	PF3D7_0533000	-2.88114	6.08E-09	5.9	5.8	5.9	3	3.6	3.8	rifin, pseudogene	RIF
Down	PF3D7_0733000	-2.86323	5.83E-09	5.7	5.9	5.9	3.7	3.5	3.3	erythrocyte membrane protein 1, PfEMP1	VAR
Down	PF3D7_0401900	-2.82511	3.50E-04	5.5	4.8	4.5	2.58	3	3.3	acyl-CoA synthetase	ACS6
Down	PF3D7_0424900	-2.71111	1.65E-03	5.2	4.7	4.3	3.17	2.6	3	Plasmodium exported protein (PHISTa), unknown function	N/A
Down	PF3D7_1478900	-2.62827	1.91E-89	12	12	12	9.53	9.5	8.9	non-coding RNA	N/A
Down	PF3D7_1200200	-2.4819	3.70E-03	4.5	4.9	4.8	3	3	3	rifin	RIF
Down	PF3D7_0400400	-2.29356	4.54E-04	5.2	5.3	5	3.46	3.8	2.8	erythrocyte membrane protein 1, PfEMP1	VAR
Down	PF3D7_0631400	-2.28381	2.35E-02	4.8	5.4	4.1	3.7	3.3	2	Pfmc-2TM Maurer's cleft two transmembrane protein	MC-2TM
Down	PF3D7_1477400	-2.2311	6.16E-11	6.7	7	6.4	4.91	4.9	4.1	Plasmodium exported protein (PHIST), unknown function	N/A
Down	PF3D7_0500100	-2.1846	1.31E-17	7.5	7.2	7.9	5.52	5.7	5.3	erythrocyte membrane protein 1, PfEMP1	VAR
Down	PF3D7_1352900	-2.17067	3.87E-02	5.2	4.5	3.8	2.58	3.3	3.3	Plasmodium exported protein, unknown function	N/A

Down	PF3D7_0701600	-2.11328	6.90E-05	5.8	5.9	5.9	4	4.7	2.8	Pfmc-2TM Maurer's cleft two transmembrane protein	MC-2TM
Down	PF3D7_0101300	-2.08844	8.82E-02	4	4.9	4.2	3	3.5	2.3	Pfmc-2TM Maurer's cleft two transmembrane protein	MC-2TM
Down	PF3D7_0425600	-2.08171	5.31E-02	4.4	4.7	4.3	3.32	3.3	2.3	rifin, pseudogene	RIF
Down	PF3D7_0832000	-2.03529	4.85E-04	5.9	5.6	4.9	3.7	4.2	3.5	stevor	N/A
Down	PF3D7_0701900	-1.72741	4.74E-02	6	4.2	4.7	4	3.7	3.6	Plasmodium exported protein, unknown function	N/A
Down	PF3D7_0424200	-1.63582	1.22E-02	6.4	5.3	5	4.46	4.5	3.8	reticulocyte binding protein homologue 4	RH4
Down	PF3D7_0424300	-1.48593	9.53E-02	6.6	5.1	4.4	4.25	3.9	4.8	erythrocyte binding antigen-165, pseudogene	EBA165
Down	PF3D7_0935600	-1.32599	5.60E-02	6.9	5.8	5.2	5.46	4.9	4.2	gametocytogenesis-implicated protein	GIG
Down	PF3D7_0402300	-1.25556	3.00E-03	7.6	7.1	6.1	6	5.6	6	reticulocyte binding protein homologue 1	RH1
Down	PF3D7_1476500	-1.25201	1.33E-05	6.8	6.8	6.8	5.75	5.5	5.6	probable protein, unknown function	N/A
Down	PF3D7_1038400	-1.17496	1.76E-02	5.6	5.8	6.2	4.75	5	4.8	gametocyte-specific protein	Pf11-1
Down	PF3D7_1401100	-1.14537	1.28E-02	6.9	6.2	5.9	5.25	5.5	5.2	DnaJ protein, putative	N/A
Down	PF3D7_1240900	-1.04416	7.68E-11	8.6	8.6	8.8	7.86	7.5	7.6	erythrocyte membrane protein 1, PfEMP1	VAR
Down	PF3D7_1240600	-1.03745	6.89E-02	6.3	5.9	6.2	4.95	5.8	4.5	erythrocyte membrane protein 1, PfEMP1	VAR
Down	PF3D7_1035900	-0.9422	6.76E-04	8.4	7.3	8.1	7.04	7.2	6.9	merozoites-associated armadillo repeats protein	MAAP
Down	PF3D7_1351700	-0.93227	1.84E-02	8.2	7	7.1	6.58	6.8	6.6	inner membrane complex protein 1f, putative	IMC1f
Down	PF3D7_1118700	-0.82953	8.47E-02	7.2	6.6	7.9	6.91	6.5	6	myosin light chain B	MLC-B
Down	PF3D7_1143100	-0.76104	6.70E-02	8.1	7.3	6.9	6.83	6.6	6.9	AP2 domain transcription factor AP2-O	AP2-O
Down	PF3D7_0501600	-0.72149	2.60E-02	8.1	7.2	8	6.97	7.1	7.2	rhoptry-associated protein 2	RAP2
Down	PF3D7_0402100	-0.71846	9.37E-02	6.7	6.7	6.3	5.88	6	5.8	Plasmodium exported protein (PHISTb), unknown function	N/A
Down	PF3D7_1476800	-0.70168	8.47E-02	6.8	6.4	6.9	5.95	6	6.2	lysophospholipase, putative	N/A
Down	PF3D7_1476300	-0.69414	3.66E-02	7.9	7.2	7.7	7.15	6.5	7.1	Plasmodium exported protein (PHISTb), unknown function	N/A
Down	PF3D7_1302100	-0.69232	3.36E-06	9.7	9.5	10	9.05	9.1	9.1	gamete antigen 27/25	G27/25
Down	PF3D7_1426300	-0.64307	3.98E-02	7.4	7.1	7.7	6.86	6.7	6.9	dynein intermediate chain, putative	N/A
Down	PF3D7_1452000	-0.61493	1.66E-02	9.2	8.3	9.1	8.37	8.2	8.3	rhoptry neck protein 2	RON2
Down	PF3D7_1478600	-0.60928	4.05E-03	8.5	8.8	8.3	7.99	8.1	7.7	EMP1-trafficking protein	PTP3

Table S3I. Differential expression between PfDNMT2 overexpression and wildtype parasite at the schizont stage

R: replicate, WT: 3D7 wild type, OE: PfDNMT2 overexpression, Adj.Pval: adjusted P value

columns G-L is EdgeR log2 (counts per million + count)

Change pattern	Gene ID	log2 Fold Change	Adj.Pval	WT R1	WT R2	WT R3	OE R1	OE R2	OE R3	Product Description	Name or Symbol
Up	PF3D7_1400200	7.144863	1.93E-07	2	2	2	4.6	5	5.1	rifin	RIF
Up	PF3D7_1150300	6.444383	6.14E-06	2	2	2	4.1	4.3	4.5	rifin	RIF
Up	PF3D7_0412600	6.413873	7.26E-06	2	2	2	4.3	4	4.5	rifin, pseudogene	RIF
Up	PF3D7_0221400	6.067496	1.30E-05	2.3	2	2	4.8	4.7	4.9	stevor	N/A
Up	PF3D7_1150400	5.791965	2.12E-55	3.3	3.5	3.8	8.7	8.2	9.1	erythrocyte membrane protein 1, PfEMP1	VAR
Up	PF3D7_0632600	5.730381	5.82E-05	2	2.3	2	4.5	4.2	4.9	rifin, pseudogene	RIF
Up	PF3D7_0800300	5.560887	2.93E-27	4.2	4.9	3.5	9.5	9.2	9.8	erythrocyte membrane protein 1, PfEMP1	VAR
Up	PF3D7_0221500	5.067764	5.09E-05	2.3	2.3	2	5	4.9	4.1	Pfmc-2TM Maurer's cleft two transmembrane protein, pseudogene	N/A
Up	PF3D7_0412400	4.409354	3.13E-64	4.5	3.8	4.8	8.5	8.4	8.7	erythrocyte membrane protein 1, PfEMP1	VAR
Up	PF3D7_0632500	4.399737	9.74E-110	5.6	5.5	5.5	9.8	9.6	10	erythrocyte membrane protein 1, PfEMP1	VAR
Up	PF3D7_0727300	3.781826	1.67E-19	6.7	6.6	6.5	11	11	9.4	DNA (cytosine-5)-methyltransferase	DNMT
Up	PF3D7_1200500	3.500554	6.41E-18	3.2	3.6	3.8	6.3	6.6	6.6	rifin	RIF
Up	PF3D7_0800200	3.350213	2.45E-42	5.7	5.5	5.3	8.7	8.2	9.2	erythrocyte membrane protein 1, PfEMP1	VAR
Up	PF3D7_0632700	3.253475	8.60E-07	4.3	4.6	2.8	7	7	7.1	rifin	RIF
Up	PF3D7_0425900	3.238632	2.19E-09	3	2.8	3.5	5.5	5.4	5.9	rifin	RIF
Up	PF3D7_1400100	2.919172	2.45E-20	3.9	4.3	4.5	7	6.7	6.8	erythrocyte membrane protein 1 (PfEMP1), pseudogene	N/A
Up	PF3D7_0100300	2.738464	3.95E-02	2.3	2.3	2.3	3.7	3.2	3.3	erythrocyte membrane protein 1, PfEMP1	VAR
Up	PF3D7_0413400	2.721637	1.82E-07	3.2	3.6	3	5.2	5.4	5.5	erythrocyte membrane protein 1 (PfEMP1), exon 1, pseudogene	VAR
Up	PF3D7_1000600	2.238914	1.61E-02	2.8	2.8	2.3	3.5	4.2	3.9	rifin	RIF
Up	PF3D7_0300100	2.207213	2.31E-29	5.9	6.4	5.9	8.2	8.2	8.3	erythrocyte membrane protein 1, PfEMP1	VAR
Up	PF3D7_0221200	2.101406	9.72E-05	3.6	3.7	3	4.8	5.5	4.9	Plasmodium exported protein (hyp15), unknown function	N/A

Up	PF3D7_0425800	2.089028	6.04E-05	3.3	3.6	4.2	5	4.8	6.1	erythrocyte membrane protein 1, PfEMP1	VAR
Up	PF3D7_1300300	2.050359	2.39E-05	3.9	3.6	3.8	5.4	4.7	6	erythrocyte membrane protein 1, PfEMP1	VAR
Up	PF3D7_1146700	1.965056	6.75E-04	4	2.6	3.3	4.7	5.1	4.9	kinesin-X4, putative	N/A
Up	PF3D7_0302300	1.718514	5.45E-07	4.9	4.6	4.8	6.4	5.7	6.8	erythrocyte membrane protein 1 (PfEMP1), pseudogene	N/A
Up	PF3D7_0115700	1.628408	6.60E-04	3.9	3.3	4.4	4.9	5.2	5.6	erythrocyte membrane protein 1, PfEMP1	VAR
Up	PF3D7_1335900	1.566413	5.38E-03	3.9	3.8	2.8	4.8	4.7	4.9	thrombospondin-related anonymous protein	TRAP
Up	PF3D7_0713100	1.542301	7.26E-02	2.8	3	3	4.1	4.1	3.3	Pfmc-2TM Maurer's cleft two transmembrane protein	MC-2TM
Up	PF3D7_0708600	1.45603	7.13E-03	4	3.6	3.5	5.2	4.5	4.7	inner membrane complex protein 1d, putative	IMC1d
Up	PF3D7_0112700	1.450348	2.69E-03	3.6	4.5	4.7	4.6	5.6	6.1	28S ribosomal RNA	N/A
Up	PF3D7_1200400	1.391013	2.09E-06	5.8	5.6	4.8	6.8	6.4	7	erythrocyte membrane protein 1, PfEMP1	VAR
Up	PF3D7_1203600	1.383546	5.56E-03	3.9	4.2	3.6	4.8	4.8	5.4	cytochrome c1 heme lyase, putative	CC1HL
Up	PF3D7_0708500	1.376492	6.05E-12	6.2	6.4	6.4	7.6	7.8	7.6	heat shock protein 86 family protein	N/A
Up	PF3D7_0707800	1.349563	3.07E-20	7.7	7.8	7.6	9.1	9	9	RAP protein, putative	N/A
Up	PF3D7_1334400	1.338591	4.53E-02	3.7	3	3.3	4.2	4.2	4.5	MSP7-like protein	MSRP4
Up	PF3D7_0109600	1.323758	8.08E-02	3.5	3.2	3	3.5	4.5	4.2	cold-shock protein, putative	CoSP
Up	PF3D7_0929700	1.314755	1.77E-02	3.9	3.6	4.2	4.3	4.6	5.6	conserved Plasmodium protein, unknown function	N/A
Up	PF3D7_1111400	1.286498	1.72E-04	5	4.8	4.4	5.9	5.8	6.1	conserved Plasmodium protein, unknown function	N/A
Up	PF3D7_1306700	1.25158	8.86E-03	4.2	3.8	4	4.9	4.9	5.3	conserved Plasmodium protein, unknown function	N/A
Up	PF3D7_1312000	1.249564	8.90E-03	4.2	4.5	3.5	5.2	4.9	5.3	malonyl CoA-acyl carrier protein transacylase	MCAT
Up	PF3D7_1135500	1.244104	9.62E-03	4.2	4.6	3.3	5.3	5.2	5	RNA-binding protein, putative	N/A
Up	PF3D7_0708000	1.238223	1.44E-08	5.9	6	6.2	7.3	7.2	7.2	cytoskeleton associated protein, putative	N/A
Up	PF3D7_0426000	1.223964	8.63E-02	3.3	3.3	3.3	4.1	3.8	4.5	erythrocyte membrane protein 1, PfEMP1	VAR
Up	PF3D7_1148200	1.201654	7.23E-12	7.2	7.3	6.9	8.4	8.3	8.2	non-coding RNA	N/A
Up	PF3D7_1223800	1.187084	1.34E-03	4.7	4.7	4.4	5.5	5.8	5.7	citrate/oxoglutarate carrier protein, putative	YHM2

Up	PF3D7_0405800	1.171667	2.18E-02	4	3.8	4.2	4.4	5.1	5.2	conserved protein, unknown function	N/A
Up	PF3D7_0819900	1.143797	6.21E-02	3	3.5	4.5	4.6	4.9	4.4	U6 snRNA-associated Sm-like protein LSm3, putative	LSM3
Up	PF3D7_0632800	1.137501	6.94E-08	6.5	6.8	6.7	7.5	7.8	8.1	erythrocyte membrane protein 1, PfEMP1	VAR
Up	PF3D7_1417100	1.13022	5.81E-03	5	4.5	4	5.6	5.7	5.2	conserved Plasmodium protein, unknown function	N/A
Up	PF3D7_0712300	1.117246	4.36E-02	3.6	3.8	4	4.6	4.9	4.5	erythrocyte membrane protein 1, PfEMP1	VAR
Up	PF3D7_0708300	1.098486	2.98E-02	4.1	3.7	4.2	5	5	4.6	EKC/KEOPS complex subunit BUD32	BUD32
Up	PF3D7_1370900	1.088527	1.21E-08	6.6	6.7	6.5	7.7	7.6	7.7	non-coding RNA	N/A
Up	PF3D7_1100200	1.070673	5.48E-04	5.5	5.2	4.9	6.1	6.2	6.4	erythrocyte membrane protein 1, PfEMP1	VAR
Up	PF3D7_0301400	1.067234	1.98E-02	4.2	4.3	4	5.2	5	5	Plasmodium exported protein, unknown function	N/A
Up	PF3D7_1101100	1.063958	4.46E-02	4	3.9	3.9	4.9	4.9	4.4	rifin	RIF
Up	PF3D7_1451500	1.058072	9.15E-02	3.7	3.3	3.8	4.6	4.1	4.5	pre-mRNA-splicing factor CWF18, putative	N/A
Up	PF3D7_1430700	1.053938	7.93E-04	5.2	5.3	4.9	5.9	6.3	6.2	NADP-specific glutamate dehydrogenase	GDH2
Up	PF3D7_0603700	1.047928	1.88E-02	4.4	4.4	4	5.2	5	5.2	phenylalanine--tRNA ligase	mFRS
Up	PF3D7_1312400	1.036816	1.93E-03	4.9	5	5	6	5.8	5.8	translation initiation factor IF-2, putative	IF2a
Up	PF3D7_1370800	1.036753	5.37E-06	6	6.2	6.2	7.3	7.1	7	non-coding RNA	N/A
Up	PF3D7_0522700	1.035625	3.07E-02	4.1	3.8	4.5	4.9	5.2	5	iron-sulfur cluster assembly protein SufA	SufA
Up	PF3D7_0810100	1.024776	9.78E-02	4	4.3	2.8	4.9	4.6	4.2	ribosomal protein L33, apicoplast, putative	N/A
Up	PF3D7_0820100	1.022695	5.51E-03	4.6	4.5	5	5.6	5.6	5.7	RNA-binding protein, putative	N/A
Up	PF3D7_1370500	1.010222	4.08E-07	6.7	7	6.8	8	7.9	7.6	non-coding RNA	N/A
Up	PF3D7_0513800	0.992157	2.43E-04	5.8	5.9	5.6	6.5	7	6.5	ras-related protein Rab-1A	RAB1a
Up	PF3D7_0707900	0.987829	1.41E-06	6.5	6.5	6.4	7.4	7.6	7.3	ribosomal protein S8e, putative	N/A
Up	PF3D7_0622400	0.984322	4.42E-02	4.5	4.1	3.9	4.8	4.9	5.2	conserved protein, unknown function	N/A
Up	PF3D7_0931300	0.961655	1.26E-02	4.9	5.1	4.2	5.8	5.8	5.4	conserved Plasmodium protein, unknown function	N/A
Up	PF3D7_1417900	0.960519	1.11E-02	4.9	5	4.5	5.3	5.7	5.8	ATP synthase-associated protein, putative	ATPTG9
Up	PF3D7_1435800	0.95717	8.13E-02	4.1	3.8	3.8	4.8	4.5	4.6	GTPase Era, putative	ERA

Up	PF3D7_0624800	0.955982	4.53E-02	4.4	4.7	3.6	5	5.2	5.1	conserved Plasmodium protein, unknown function	N/A
Up	PF3D7_0526600	0.950297	1.45E-09	8.4	8.5	8.2	9.3	9.2	9.5	conserved Plasmodium protein, unknown function	N/A
Up	PF3D7_1358900	0.949613	1.69E-03	5.5	5	5.5	6.4	6.3	6	GTP-binding protein, putative	N/A
Up	PF3D7_1310900	0.947785	5.45E-02	4.3	3.8	4.2	5	4.9	4.9	ribosomal protein S15, mitochondrial, putative	N/A
Up	PF3D7_0320300	0.944096	8.70E-08	7.3	7.4	7	8.2	8.2	8.1	T-complex protein 1 subunit epsilon	CCT5
Up	PF3D7_1318600	0.927551	2.53E-02	4.9	4.2	4.5	5.3	5.4	5.5	thioredoxin, putative	N/A
Up	PF3D7_0113800	0.925281	4.70E-03	5.2	4.7	5.5	5.9	6.1	6	DBL containing protein, unknown function	N/A
Up	PF3D7_1112400	0.922105	4.41E-02	4.6	4.7	4.7	5.2	6.1	4.7	nucleic acid binding protein, putative	N/A
Up	PF3D7_1200600	0.919743	1.41E-07	7.4	7.3	7.1	8.1	8.1	8.3	erythrocyte membrane protein 1, PfEMP1	VAR2CSA
Up	PF3D7_1141600	0.919327	3.38E-02	4.2	4.7	4.4	5.1	5.4	5.2	dolichol-phosphate mannosyltransferase	DPM1
Up	PF3D7_0315200	0.913316	1.12E-03	5.5	5.8	5.8	6.7	6.7	6.2	circumsporozoite- and TRAP-related protein	CTRP
Up	PF3D7_0918400	0.911533	3.04E-02	4.7	5	4.2	5.7	5.6	5	conserved Plasmodium protein, unknown function	N/A
Up	PF3D7_0608800	0.906832	2.43E-11	8.6	8.7	8.5	9.4	9.5	9.6	ornithine aminotransferase	OAT
Up	PF3D7_1361600	0.904333	1.85E-02	5.4	5.2	4.6	5.5	5.7	6.4	Fe-S assembly protein IscX, putative	YfhJ
Up	PF3D7_1240300	0.898514	1.02E-07	7.2	7.5	7.3	8.2	8.3	8.2	erythrocyte membrane protein 1, PfEMP1	VAR
Up	PF3D7_0520200	0.898235	6.50E-04	5.9	5.7	5.8	6.8	6.8	6.4	mediator of RNA polymerase II transcription subunit 17, putative	MED17
Up	PF3D7_0519300	0.893023	3.97E-03	5.5	5.6	5.3	6.5	6.6	5.8	protoheme IX farnesyltransferase	COX10
Up	PF3D7_0810000	0.890431	5.57E-04	5.6	6.3	5.9	6.7	6.9	6.8	acyl-CoA binding protein, putative	N/A
Up	PF3D7_0708100	0.887426	5.87E-02	4.5	4.1	4.2	5	4.9	5.1	DNA-directed RNA polymerases I, II, and III subunit RPABC5, putative	RPB10
Up	PF3D7_1114500	0.88618	8.68E-02	4.2	4.2	3.8	4.9	4.8	4.6	conserved Plasmodium protein, unknown function	N/A
Up	PF3D7_0821900	0.885045	8.27E-02	4.2	4.6	3.8	4.9	5.4	4.5	WPP motif-containing protein, putative	N/A
Up	PF3D7_0320400	0.884869	1.09E-02	5.4	5.1	4.7	5.7	6	6	oocyst capsule protein Cap380	Cap380
Up	PF3D7_0728200	0.884195	4.47E-03	5.3	5.7	5.1	6	6.2	6.4	actin-like protein, putative	ALP3

Up	PF3D7_1309800	0.882946	9.84E-02	4.4	3.7	4	4.9	4.9	4.5	homeobox domain-containing protein, putative	N/A
Up	PF3D7_0731600	0.875854	1.97E-08	8.7	9	8.6	9.7	9.8	9.5	acyl-CoA synthetase	ACS5
Up	PF3D7_1029800	0.870362	3.89E-05	6.6	6.5	6.5	7.2	7.5	7.5	heptatricopeptide repeat and RAP domain-containing protein, putative	HPR2
Up	PF3D7_1429600	0.867858	1.60E-02	4.9	4.8	5	5.5	5.8	5.8	conserved Plasmodium protein, unknown function	GEXP03
Up	PF3D7_1204700	0.866093	1.88E-02	4.7	4.8	5.2	5.6	5.9	5.5	conserved protein, unknown function	N/A
Up	PF3D7_1463300	0.857094	4.68E-03	5.7	5.8	5.1	6.2	6.6	6.2	DNA polymerase alpha subunit B, putative	N/A
Up	PF3D7_0829800	0.853111	3.36E-10	8.8	8.8	9	9.8	9.7	9.7	non-coding RNA	N/A
Up	PF3D7_0209300	0.847254	3.60E-02	4.6	4.8	4.5	5.2	5.6	5.4	2C-methyl-D-erythritol 2,4-cyclodiphosphate synthase	IspF
Up	PF3D7_0715500	0.846475	7.57E-02	4.1	4.7	4.2	4.9	4.9	5.3	ATP synthase subunit epsilon, mitochondrial, putative	N/A
Up	PF3D7_0204000	0.845958	9.03E-02	4.6	4.2	3.8	4.9	4.9	4.9	condensin-2 complex subunit H2, putative	CAPH2
Up	PF3D7_1144800	0.844504	4.37E-02	4.9	4.7	4.2	5.3	5.2	5.6	conserved Plasmodium protein, unknown function	N/A
Up	PF3D7_1024700	0.843763	3.61E-03	5.6	5.6	5.6	6.5	6.6	6	conserved protein, unknown function	N/A
Up	PF3D7_0616000	0.843626	8.20E-03	5.2	5.3	5.3	5.8	6	6.3	pyridoxal kinase	PDXK
Up	PF3D7_1451400	0.843442	3.77E-03	5.5	5.8	5.3	6.5	6.2	6.3	transcriptional regulatory protein sir2b	Sir2B
Up	PF3D7_1323600	0.838612	1.73E-03	5.7	5.8	5.6	6.5	6.6	6.4	conserved protein, unknown function	N/A
Up	PF3D7_1012400	0.837546	3.70E-06	8.4	8.2	8.2	9.1	9.4	8.7	hypoxanthine-guanine phosphoribosyltransferase	HGPRT
Up	PF3D7_0303900	0.833547	6.95E-02	4.3	4.6	4.2	5.1	4.9	5.2	phosphatidylethanolamine-binding protein, putative	N/A
Up	PF3D7_1243100	0.832813	1.66E-02	5.1	4.9	5.1	5.7	5.9	5.6	zinc finger protein, putative	N/A
Up	PF3D7_1111500	0.832519	5.59E-03	5.3	5.4	5.3	6.2	6	6.2	acylphosphatase, putative	N/A
Up	PF3D7_1124500	0.829714	1.70E-02	5.3	5.1	4.8	5.9	5.9	5.6	pyruvate dehydrogenase E1 component subunit alpha	pdhA
Up	PF3D7_0908300	0.827166	9.88E-04	6.1	6.3	5.8	6.7	7	6.8	conserved protein, unknown function	N/A
Up	PF3D7_0404500	0.825711	4.91E-02	4.9	4.2	4.6	5.3	5.2	5.4	6-cysteine protein P52	P52
Up	PF3D7_0902800	0.824723	7.71E-08	7.8	7.9	7.7	8.6	8.7	8.6	serine repeat antigen 9	SERA9

Up	PF3D7_0602400	0.822486	2.67E-05	6.9	7.2	6.7	7.7	7.9	7.8	elongation factor G	EF-G
Up	PF3D7_0626800	0.821184	8.45E-08	9.8	10	9.7	11	11	10	pyruvate kinase	PyrK
Up	PF3D7_0317900	0.820991	8.00E-02	4.7	3.9	4.4	5	5.1	5.1	conserved protein, unknown function	N/A
Up	PF3D7_0404600	0.820932	5.71E-09	8.3	8.4	8.2	9.1	9.1	9.2	conserved Plasmodium membrane protein, unknown function	N/A
Up	PF3D7_1215200	0.81996	1.08E-02	5.4	5.7	5	5.9	6.3	6.2	peptidyl-prolyl cis-trans isomerase	CYP32
Up	PF3D7_0623300	0.817988	3.59E-03	5.7	5.7	5.5	6.5	6.5	6.2	EGF-like membrane protein, putative	N/A
Up	PF3D7_1129500	0.817225	1.22E-03	6	6.1	5.8	6.9	6.7	6.6	A/G-specific adenine glycosylase, putative	N/A
Up	PF3D7_0627200	0.816644	6.97E-02	4.5	4.6	4.2	5	5	5.4	myosin light chain, putative	N/A
Up	PF3D7_0812220	0.812336	5.00E-02	4.7	4.8	4.5	5.1	5.7	5.3	GTP-binding protein YihA3	YihA3
Up	PF3D7_0521500	0.809131	5.75E-02	4.6	4.7	4.5	5	5.5	5.3	ribosomal large subunit pseudouridylate synthase, putative	N/A
Up	PF3D7_1406000	0.80906	8.69E-02	4.2	4.5	4.2	4.9	4.9	5.1	RNA-binding protein 8A, putative	RBM8A
Up	PF3D7_1427700	0.809011	8.80E-02	4.3	4.2	4.4	5.1	5.1	4.7	conserved Plasmodium protein, unknown function	N/A
Up	PF3D7_1106200	0.809003	1.49E-02	5	5.3	5.2	6	5.7	5.9	conserved Plasmodium protein, unknown function	N/A
Up	PF3D7_1460800	0.806644	2.48E-02	4.9	5.2	4.9	5.5	5.9	5.7	snRNA-activating protein complex subunit 3, putative	SNAPC3
Up	PF3D7_1034200	0.805848	6.56E-02	4.6	5	4.2	5.5	5.5	5	ribosomal protein L27, putative	RPL27
Up	PF3D7_1212600	0.80396	2.40E-02	4.9	4.8	5.1	5.7	5.7	5.6	SND2 domain-containing protein, putative	N/A
Up	PF3D7_1103400	0.803702	6.50E-06	7.5	7.5	7.3	8.4	8.1	8.1	iron-sulfur cluster assembly protein SufD	SufD
Up	PF3D7_0417800	0.799561	4.98E-05	7	7.1	6.8	7.9	7.8	7.6	cdc2-related protein kinase 1	CRK1
Up	PF3D7_0805000	0.799406	1.25E-02	5.4	5.4	5	5.8	6.1	6.1	alpha/beta hydrolase, putative	N/A
Up	PF3D7_1314300	0.797768	9.86E-03	5.4	5.3	5.3	6	6.2	6	MACRO domain-containing protein, putative	N/A
Up	PF3D7_0801100	0.797618	5.08E-02	4.5	4.6	5.2	5.2	5.7	5.6	28S ribosomal RNA	N/A
Up	PF3D7_1248300	0.796873	4.43E-02	4.5	4.7	4.9	5.3	5.4	5.5	conserved protein, unknown function	N/A
Up	PF3D7_0500800	0.79419	4.99E-11	11	11	11	11	12	11	mature parasite-infected erythrocyte surface antigen	MESA

Up	PF3D7_1222100	0.79168	1.50E-02	5.1	5.6	5.1	6	6.1	5.9	conserved Plasmodium protein, unknown function	N/A
Up	PF3D7_0623900	0.791007	1.86E-02	5	5.2	5.1	5.8	5.9	5.8	ribonuclease H2 subunit A, putative	N/A
Up	PF3D7_1303100	0.790948	1.35E-02	5.2	5.2	5.3	5.8	6.1	6	methyltransferase, putative	N/A
Up	PF3D7_0829900	0.787734	2.55E-08	8.6	8.9	8.9	9.7	9.6	9.5	non-coding RNA	N/A
Up	PF3D7_0508300	0.783166	1.67E-03	6.1	6.2	5.7	6.8	6.8	6.8	triose phosphate transporter	TPT
Up	PF3D7_1237000	0.782902	1.40E-04	6.9	6.8	6.6	7.7	7.6	7.4	SUMO-activating enzyme subunit 2	UBA2
Up	PF3D7_1334500	0.781745	1.77E-02	5.3	5.2	5.1	5.7	6.1	5.9	MSP7-like protein	MSRP6
Up	PF3D7_1026300	0.781242	1.00E-02	5.5	5.5	5.3	6.3	6.2	6	conserved Plasmodium protein, unknown function	N/A
Up	PF3D7_0627500	0.780907	1.02E-03	6.3	6.6	6	7.1	7.2	6.9	protein DJ-1	DJ1
Up	PF3D7_1460700	0.779309	3.27E-06	7.4	7.4	7.6	8.1	8.3	8.2	60S ribosomal protein L27	RPL27
Up	PF3D7_1314500	0.779296	2.98E-03	5.9	6.3	5.7	6.9	6.7	6.7	transmembrane emp24 domain-containing protein, putative	N/A
Up	PF3D7_1366500	0.772279	2.26E-03	6	6.4	6.1	7	7.1	6.6	nucleoside diphosphate kinase	NDK
Up	PF3D7_1317300	0.772014	7.60E-04	6.3	6.7	6.2	7.2	7.1	7.2	RNA-binding protein, putative	N/A
Up	PF3D7_1309500	0.77131	8.45E-02	5	4.6	4	5.2	5.3	5.3	H/ACA ribonucleoprotein complex subunit 1, putative	GAR1
Up	PF3D7_1016300	0.767502	3.19E-09	11	11	10	11	11	11	GBP130 protein	GBP130
Up	PF3D7_1204500	0.767361	4.43E-02	4.7	5.1	4.9	5.5	5.9	5.4	conserved protein, unknown function	N/A
Up	PF3D7_1422800	0.765995	4.61E-02	4.9	4.8	4.8	5.6	5.5	5.4	actin-related protein ARP4	ARP4
Up	PF3D7_0728700	0.761609	8.27E-02	4.4	4.6	4.8	5.4	4.9	5.5	alpha/beta hydrolase, putative	N/A
Up	PF3D7_1460300	0.76131	2.29E-04	7	7.1	6.8	7.6	8	7.5	60S ribosomal protein L29, putative	RPL29
Up	PF3D7_1128200	0.759916	5.22E-02	4.9	4.6	5.3	5.5	5.9	5.4	multiprotein-bridging factor 1, putative	MBF1
Up	PF3D7_1337200	0.751024	8.88E-04	6.3	6.8	6.4	7.2	7.3	7.2	1-deoxy-D-xylulose 5-phosphate synthase	DXS
Up	PF3D7_0626900	0.750105	5.93E-02	5	5	4.4	5.7	5.5	5.3	ribosomal protein L46, mitochondrial, putative	N/A
Up	PF3D7_1324900	0.74937	1.59E-08	9.3	9.4	9.2	10	10	10	L-lactate dehydrogenase	LDH
Up	PF3D7_0710400	0.747481	7.19E-02	4.6	4.9	4.5	5.5	5.2	5.2	DNA repair protein RAD14, putative	RAD14
Up	PF3D7_1234700	0.747449	3.14E-02	5.3	5.3	4.8	5.6	6.1	5.9	upregulated in late gametocytes ULG8	ULG8

Up	PF3D7_0708400	0.746012	5.78E-10	12	11	12	12	12	12	heat shock protein 90	HSP90
Up	PF3D7_1467300	0.745889	1.05E-03	6.5	6.4	6.2	7	7	7.2	1-deoxy-D-xylulose 5-phosphate reductoisomerase	DXR
Up	PF3D7_1303000	0.742488	6.23E-02	4.8	4.8	5	5.6	5.8	5.1	ATP synthase-associated protein, putative	ATPTG13
Up	PF3D7_1475300	0.735633	2.98E-02	5.2	5.3	5	6	5.7	5.9	cytochrome c oxidase assembly protein COX11, putative	COX11
Up	PF3D7_0718100	0.732643	5.41E-06	7.9	7.8	7.8	8.5	8.7	8.4	exported serine/threonine protein kinase	EST
Up	PF3D7_1402500	0.731478	2.68E-04	7	7	6.8	7.8	7.8	7.4	ribosomal protein S27a, putative	N/A
Up	PF3D7_1439900	0.730022	3.57E-05	7.7	7.7	7.4	8.3	8.4	8.1	triosephosphate isomerase	TIM
Up	PF3D7_1123700	0.729076	9.10E-02	4.5	4.6	4.7	5.3	5	5.4	ribosomal protein L37, mitochondrial, putative	N/A
Up	PF3D7_0204800	0.728581	4.69E-02	4.9	5	5.2	6	5.6	5.5	3'-5' exonuclease, putative	N/A
Up	PF3D7_1411600	0.724623	5.62E-02	4.9	5.1	4.8	5.9	5.6	5.3	GTP-binding protein Obg1	Obg1
Up	PF3D7_1130700	0.724183	3.52E-06	8	8.1	7.8	8.8	8.7	8.6	structural maintenance of chromosomes protein 1, putative	SMC1
Up	PF3D7_0320000	0.72367	1.28E-03	6.2	6.6	6.5	7.1	7.2	7.2	protein phosphatase inhibitor 2	I2
Up	PF3D7_1438700	0.722015	1.54E-03	6.5	6.9	6.3	7.3	7.4	7.2	DNA primase small subunit	N/A
Up	PF3D7_1454700	0.721006	8.71E-05	7.7	7.8	7.3	8.3	8.4	8.2	6-phosphogluconate dehydrogenase, decarboxylating	6PGD
Up	PF3D7_0603200	0.720482	1.31E-02	5.7	5.7	5.7	6.5	6.5	6	mitochondrial chaperone BCS1, putative	BCS1
Up	PF3D7_1032000	0.719025	1.35E-02	5.8	5.7	6	6.7	6.7	6.1	ribosome maturation factor RimM, putative	RimM
Up	PF3D7_1215900	0.718324	1.07E-02	5.8	5.6	5.6	6.2	6.5	6.4	serpentine receptor 10	SR10
Up	PF3D7_0319500	0.718173	3.61E-03	6	6.4	6.5	7.2	7	6.7	RNA-binding protein, putative	N/A
Up	PF3D7_1356900	0.717857	9.75E-03	5.9	5.9	5.5	6.4	6.4	6.6	protein kinase 5	PK5
Up	PF3D7_1210700	0.716517	1.64E-02	5.6	5.6	5.5	6	6.4	6.3	conserved Plasmodium protein, unknown function	N/A
Up	PF3D7_1351100	0.714593	4.49E-02	5.5	5.1	4.8	5.6	5.8	6	conserved protein, unknown function	N/A
Up	PF3D7_0715600	0.712369	7.07E-05	7.3	7.5	7.2	8	8	7.9	GTP-binding translation elongation factor tu family protein, putative	N/A
Up	PF3D7_1223700	0.711961	4.35E-03	6.2	6.7	6.2	7.1	7.3	6.8	vacuolar iron transporter	VIT

Up	PF3D7_0411300	0.711413	3.61E-02	5.6	5	5.3	5.7	6.2	5.9	conserved Plasmodium protein, unknown function	N/A
Up	PF3D7_0203400	0.711131	2.66E-03	6.2	6.8	6.6	7.3	7.4	7	conserved protein, unknown function	N/A
Up	PF3D7_1104300	0.708111	5.60E-05	7.4	7.6	7.4	8.3	8.1	8.1	conserved Plasmodium protein, unknown function	N/A
Up	PF3D7_1228100	0.707925	7.56E-02	4.9	4.8	4.8	5.2	5.4	5.8	leucine-rich repeat protein	LRR13
Up	PF3D7_1411400	0.707094	1.67E-04	7	7.2	7.1	7.8	7.9	7.7	plastid replication-repair enzyme	PREX
Up	PF3D7_1357100	0.707038	9.66E-07	8.9	8.9	8.6	9.5	9.6	9.4	elongation factor 1-alpha	N/A
Up	PF3D7_0912100	0.706885	4.53E-02	5.3	5.2	4.8	5.7	5.7	5.8	zinc finger protein, putative	N/A
Up	PF3D7_0505900	0.706362	3.76E-02	5.2	5.6	5	5.8	6.2	5.9	mediator of RNA polymerase II transcription subunit 11, putative	MED11
Up	PF3D7_0608100	0.706212	3.99E-05	7.4	7.6	7.6	8.2	8.3	8.1	conserved Plasmodium protein, unknown function	N/A
Up	PF3D7_0727500	0.705174	1.49E-02	5.8	5.9	5.4	6.2	6.5	6.5	mTERF domain-containing protein, putative	N/A
Up	PF3D7_1353800	0.703461	2.09E-04	7	7.1	7.3	7.7	7.8	7.9	proteasome subunit alpha type-4, putative	N/A
Up	PF3D7_1416900	0.70049	9.23E-02	5	4.5	4.6	5.2	5.5	5.4	prefoldin subunit 2, putative	N/A
Up	PF3D7_1439200	0.69937	6.95E-02	5.1	4.5	5.2	5.5	5.7	5.5	conserved Plasmodium protein, unknown function	N/A
Up	PF3D7_1121900	0.695875	1.12E-03	6.7	6.8	6.5	7.2	7.4	7.5	3-phosphoinositide-dependent protein kinase 1	PDK1
Up	PF3D7_1347900	0.695181	9.14E-03	5.7	6.2	5.8	6.7	6.7	6.5	conserved Plasmodium protein, unknown function	N/A
Up	PF3D7_0802200	0.69398	1.41E-03	7.2	7.1	6.7	7.6	7.9	7.5	1-cys peroxiredoxin	1-CysPxn
Up	PF3D7_0716500	0.693027	1.61E-02	5.6	5.9	5.7	6.5	6.1	6.4	conserved Plasmodium protein, unknown function	N/A
Up	PF3D7_0915900	0.69226	3.21E-02	5.5	5	5.5	6	6	6.1	conserved Plasmodium protein, unknown function	N/A
Up	PF3D7_1241700	0.691771	9.34E-03	6.4	6.6	6.1	6.9	7.4	6.8	replication factor C subunit 4, putative	RFC4
Up	PF3D7_1308900	0.691218	1.14E-05	7.7	7.8	7.8	8.5	8.5	8.4	mRNA-decapping enzyme 2, putative	DCP2
Up	PF3D7_0628600	0.690079	1.11E-02	5.8	5.9	5.7	6.3	6.5	6.5	DNA methyltransferase 1-associated protein 1, putative	N/A
Up	PF3D7_1106100	0.687028	1.57E-02	5.8	5.8	5.5	6.3	6.3	6.5	ribosomal protein S15, apicoplast, putative	N/A

Up	PF3D7_0209200	0.68594	3.98E-02	5.5	5.2	5.5	5.6	6.2	6.2	exosome complex component MTR3, putative	MTR3
Up	PF3D7_0110100	0.685722	2.90E-02	5.5	5.5	5.5	6.1	6.4	5.9	selenocysteine-specific elongation factor, putative	SELB
Up	PF3D7_1141400	0.684326	8.53E-04	7.3	7.5	6.8	7.8	7.9	8	phosphatidylinositol N-acetylglucosaminyltransferase subunit H, putative	PIGH
Up	PF3D7_0320800	0.683473	7.61E-04	6.9	6.6	7	7.4	7.5	7.6	ATP-dependent RNA helicase DDX6	DOZI
Up	PF3D7_0728300	0.683393	4.96E-02	5.3	5	5.2	5.9	5.7	5.7	conserved protein, unknown function	N/A
Up	PF3D7_0628800	0.67986	1.23E-02	6.1	6.1	5.9	6.5	7	6.4	glutamyl-tRNA(Gln) amidotransferase subunit B	GATB
Up	PF3D7_1344000	0.67744	7.38E-02	5	4.8	5	5.8	5.5	5.5	aminomethyltransferase, putative	N/A
Up	PF3D7_1245100	0.675404	2.56E-03	6.6	7.2	6.6	7.5	7.5	7.5	kinesin-13, putative	KLP8
Up	PF3D7_1201000	0.673085	9.14E-03	6.7	6.8	6.5	7.3	7.7	6.8	Plasmodium exported protein (PHISTb), unknown function	N/A
Up	PF3D7_0935400	0.672736	2.15E-03	6.7	6.7	6.4	7.2	7.4	7.1	gametocyte development protein 1	GDV1
Up	PF3D7_0207900	0.670606	1.29E-03	7	7.3	6.7	7.5	7.7	7.7	serine repeat antigen 2	SERA2
Up	PF3D7_1311300	0.669445	1.44E-02	6	6.2	5.7	6.4	6.6	6.8	ATP synthase subunit gamma, mitochondrial	N/A
Up	PF3D7_0723700	0.668664	7.33E-03	6.1	6.2	6.3	6.8	7.1	6.7	metallo-hydrolase/oxidoreductase, putative	N/A
Up	PF3D7_1127100	0.668594	1.24E-02	5.9	6.1	5.9	6.5	6.8	6.5	deoxyuridine 5'-triphosphate nucleotidohydrolase	dUTPase
Up	PF3D7_1029600	0.66834	2.35E-06	8.3	8.4	8.3	9	9	9	adenosine deaminase	ADA
Up	PF3D7_1431000	0.667348	3.83E-02	5.3	5.6	5.3	5.8	6.2	6	ribosomal protein L17, mitochondrial, putative	N/A
Up	PF3D7_0422300	0.666057	3.74E-03	6.6	6.6	6.3	7.3	7.2	7	alpha tubulin 2	N/A
Up	PF3D7_1135400	0.665153	2.00E-03	6.7	6.7	6.4	7.2	7.2	7.4	HotDog domain-containing protein, putative	N/A
Up	PF3D7_1138600	0.663305	3.74E-02	5.6	5.4	5.3	6.3	6	5.9	beta-catenin-like protein 1, putative	N/A
Up	PF3D7_0618200	0.662852	7.67E-03	6.1	6.3	6.1	6.7	6.9	6.7	conserved protein, unknown function	N/A
Up	PF3D7_0209400	0.66147	3.78E-02	5.5	5.6	5.2	6.1	6.1	6	ARMT1-like domain-containing protein, putative	N/A
Up	PF3D7_1355900	0.660598	4.35E-02	5.4	5.3	5.5	6.2	6	5.7	RWD domain-containing protein, putative	N/A

Up	PF3D7_1329300	0.66013	4.19E-04	7.9	7.9	7.4	8.4	8.5	8.3	chromatin assembly factor 1 subunit B, putative	CAF1B
Up	PF3D7_0310000	0.659314	2.90E-02	5.7	6	5.3	6.3	6.3	6.3	50S ribosomal protein L9, apicoplast, putative	N/A
Up	PF3D7_1416800	0.658409	4.21E-02	5.5	5.2	5.3	5.9	5.9	6	lysine--tRNA ligase, putative	KRS2
Up	PF3D7_0827900	0.658159	5.20E-06	10	11	10	11	11	11	protein disulfide-isomerase	PDI8
Up	PF3D7_1227100	0.657536	3.86E-03	6.3	6.5	6.5	6.9	7.1	7.1	DNA helicase 60	DH60
Up	PF3D7_0910900	0.656823	2.40E-03	6.7	6.9	6.5	7.2	7.3	7.5	DNA primase large subunit, putative	N/A
Up	PF3D7_1147700	0.656607	1.23E-02	6.4	6.5	6.1	6.6	7	7.3	ATP synthase subunit delta, mitochondrial, putative	N/A
Up	PF3D7_1216500	0.656126	1.50E-02	5.9	6	5.8	6.5	6.7	6.4	male development gene 1	MDV1
Up	PF3D7_1449800	0.655355	8.65E-02	5.2	5.2	4.8	5.5	6	5.5	conserved protein, unknown function	N/A
Up	PF3D7_0204500	0.65449	1.49E-02	5.9	5.7	6	6.5	6.5	6.5	aspartate transaminase	AspAT
Up	PF3D7_1360200	0.654428	1.71E-02	6	6.2	5.8	6.6	6.4	6.8	ER membrane protein complex subunit 3, putative	EMC3
Up	PF3D7_0707400	0.654389	7.21E-04	7.1	7	6.9	7.6	7.6	7.8	AAA family ATPase, putative	N/A
Up	PF3D7_1111100	0.653762	6.23E-04	7.1	7.1	6.9	7.7	7.7	7.6	replication factor C subunit 5, putative	RFC5
Up	PF3D7_0916500	0.651714	1.05E-02	6.3	6.1	5.9	6.6	6.7	6.8	ubiquitin fusion degradation protein 1	UFD1
Up	PF3D7_0728800	0.650841	1.28E-02	6	5.8	6.2	6.6	6.6	6.7	conserved Plasmodium protein, unknown function	N/A
Up	PF3D7_0513300	0.650724	1.46E-04	8	8.2	7.8	8.7	8.7	8.5	purine nucleoside phosphorylase	PNP
Up	PF3D7_0112000	0.650442	4.79E-02	5.3	5.2	5.7	5.8	6.1	6.2	TatD-like deoxyribonuclease	TatD
Up	PF3D7_1141200	0.646731	6.59E-02	5.2	5.2	5	5.7	5.7	5.7	ATP synthase-associated protein, putative	N/A
Up	PF3D7_0107000	0.646501	1.89E-03	7.2	7.7	7.2	8	8.3	7.8	centrin-1	CEN1
Up	PF3D7_0711500	0.64552	5.24E-04	7.6	7.8	7.3	8.3	8.2	8.1	regulator of chromosome condensation, putative	N/A
Up	PF3D7_0501200	0.644674	8.75E-04	7.2	7.3	7.3	8	8	7.6	parasite-infected erythrocyte surface protein	PIESP2
Up	PF3D7_1004800	0.644599	1.10E-02	6	6.1	6.1	6.6	6.6	6.7	ADP,ATP carrier protein 2	AAC2
Up	PF3D7_0906100	0.641873	7.73E-03	6.1	6.5	6.4	6.8	7.1	6.9	vacuolar protein sorting-associated protein 46, putative	VPS46
Up	PF3D7_0505500	0.641743	3.36E-05	8.6	8.8	8.4	9.2	9.3	9.2	DNA mismatch repair protein MSH6, putative	MSH6

Up	PF3D7_1249500	0.640306	1.80E-02	6.1	6.1	5.6	6.5	6.6	6.5	protein CutA, putative	N/A
Up	PF3D7_0413700	0.63976	2.21E-04	7.8	7.9	7.6	8.5	8.4	8.2	lysine decarboxylase-like protein, putative	N/A
Up	PF3D7_0715900	0.63944	1.67E-04	7.8	7.6	7.6	8.3	8.4	8.2	cation diffusion facilitator family protein, putative	CDF
Up	PF3D7_0726600	0.639131	2.90E-02	5.9	5.8	6.2	6.2	6.6	6.9	conserved Plasmodium protein, unknown function	N/A
Up	PF3D7_1462800	0.637473	5.85E-05	11	11	11	11	12	11	glyceraldehyde-3-phosphate dehydrogenase	GAPDH
Up	PF3D7_0918500	0.636326	3.71E-03	7.1	7.4	7.6	7.8	8.3	7.8	telomerase RNA	TR
Up	PF3D7_1343400	0.636308	2.55E-03	7.1	7.1	6.7	7.5	7.5	7.8	DNA repair protein RAD5, putative	RAD5
Up	PF3D7_1342300	0.635773	4.65E-02	5.7	5.6	5.2	6	6	6.2	tetratricopeptide repeat protein, putative	N/A
Up	PF3D7_1324800	0.634787	1.49E-03	7.1	7.3	6.9	7.8	7.8	7.6	dihydrofolate synthase/folylpolyglutamat e synthase	DHFS-FPGS
Up	PF3D7_0517400	0.634724	1.08E-05	8.7	8.8	8.6	9.4	9.3	9.2	FACT complex subunit SPT16, putative	FACT-L
Up	PF3D7_1411700	0.634017	3.29E-02	5.7	5.7	5.5	6.1	6.4	6.2	methyltransferase, putative	N/A
Up	PF3D7_0618300	0.633362	9.45E-04	7.7	7.7	7.4	8.3	8.4	8	60S ribosomal protein L27a, putative	N/A
Up	PF3D7_1038100	0.632	6.93E-02	5.5	5	5.3	6	5.7	5.8	GDP dissociation inhibitor, putative	N/A
Up	PF3D7_1366400	0.630684	1.73E-04	7.7	8	7.8	8.3	8.5	8.5	rhoptry protein RHOP148	RHOP148
Up	PF3D7_0920200	0.630275	4.72E-05	7.9	8.2	8	8.7	8.7	8.6	CS domain protein, putative	N/A
Up	PF3D7_0913700	0.62957	1.16E-04	7.6	7.6	7.7	8.2	8.3	8.2	conserved protein, unknown function	N/A
Up	PF3D7_1019300	0.628501	4.43E-05	8.1	8.3	8	8.8	8.7	8.7	zinc finger protein, putative	N/A
Up	PF3D7_1030600	0.627879	3.52E-02	5.7	5.6	5.6	6.2	6.4	6	tRNA N6-adenosine threonylcarbamoyltransfer ase	KAE1
Up	PF3D7_1323100	0.626614	7.08E-04	7.3	7.6	7.4	8.1	8.2	7.8	60S ribosomal protein L6, putative	N/A
Up	PF3D7_0626100	0.624335	4.32E-02	5.5	5.5	5.5	6	6.2	6.1	oxidoreductase, short-chain dehydrogenase family, putative	N/A
Up	PF3D7_0623000	0.623964	3.93E-03	6.6	6.7	6.5	7.2	7.3	7.1	chorismate synthase	CS
Up	PF3D7_0514900	0.623963	2.53E-02	6.1	5.6	5.9	6.4	6.5	6.5	conserved Plasmodium protein, unknown function	N/A
Up	PF3D7_1360500	0.623737	8.30E-02	5.5	5.3	5.3	5.6	5.7	6.4	guanylyl cyclase beta	GCbeta
Up	PF3D7_1316800	0.622046	7.30E-03	6.8	7	6.3	7.3	7.4	7.3	protein transport protein SEC20, putative	SEC20

Up	PF3D7_0521400	0.622015	4.84E-02	5.7	5.6	5.5	6.5	6.1	5.9	conserved protein, unknown function	N/A
Up	PF3D7_1429100	0.620942	2.75E-04	7.6	7.6	7.4	8.1	8.1	8.2	ribosomal protein L15, apicoplast, putative	N/A
Up	PF3D7_0624100	0.620051	1.66E-02	6	6	6.1	6.5	6.7	6.7	conserved protein, unknown function	N/A
Up	PF3D7_0305100	0.619802	2.18E-05	8.5	8.6	8.3	9.1	9.1	9.2	conserved Plasmodium protein, unknown function	N/A
Up	PF3D7_1205600	0.619696	2.76E-03	7.1	7.3	7.1	7.6	8	7.7	tetratricopeptide repeat protein, putative	N/A
Up	PF3D7_1229400	0.619519	9.27E-04	7.1	7.4	7.2	7.9	7.9	7.8	macrophage migration inhibitory factor	MIF
Up	PF3D7_1363700	0.619053	1.55E-02	6.1	6.5	6.1	6.7	7	6.8	protein AMR1	AMR1
Up	PF3D7_0933600	0.618846	2.11E-03	7.3	7.2	6.9	7.7	7.9	7.6	mitochondrial-processing peptidase subunit beta, putative	QCR1
Up	PF3D7_1144700	0.618823	4.68E-02	5.3	5.6	5.8	6.1	6.1	6.3	apicoplast import protein Tic20, putative	TIC20
Up	PF3D7_1249900	0.618617	5.68E-03	6.6	6.7	6.5	7	7.3	7.3	ribosomal RNA small subunit methyltransferase A2, putative	KsgA2
Up	PF3D7_0316600	0.618274	3.18E-04	8.1	8.1	8.1	8.7	9	8.5	formate-nitrite transporter	FNT
Up	PF3D7_1409900	0.617047	1.20E-04	7.8	7.7	7.7	8.4	8.4	8.3	cytidine diphosphate-diacylglycerol synthase	CDS
Up	PF3D7_0904100	0.616663	3.09E-05	8.1	8.2	8.2	8.7	8.8	8.8	AP-4 complex subunit epsilon, putative	N/A
Up	PF3D7_1356400	0.615685	2.90E-02	6.4	6.5	6	7.1	7.2	6.4	phosphatase 2A regulatory subunit-related protein, putative	N/A
Up	PF3D7_0803700	0.614092	1.44E-02	6.1	6.3	6.1	6.8	6.6	6.9	tubulin gamma chain	g-tub
Up	PF3D7_1239700	0.613893	1.56E-05	8.8	9.1	8.8	9.5	9.6	9.4	ATP-dependent zinc metalloprotease FTSH 1	FTSH1
Up	PF3D7_0608000	0.613865	5.86E-02	5.3	5.8	5.3	6	6.2	6	diphthine methyltransferase, putative	DPH7
Up	PF3D7_1015600	0.61359	1.59E-03	7	7	7	7.5	7.5	7.7	heat shock protein 60	HSP60
Up	PF3D7_1249300	0.613451	5.47E-04	7.7	8	7.6	8.3	8.5	8.2	protein phosphatase PPM4, putative	PPM4
Up	PF3D7_0605200	0.612204	2.78E-02	6	6.2	5.8	6.9	6.5	6.4	acylphosphatase, putative	N/A
Up	PF3D7_1132700	0.611722	4.29E-02	5.5	5.5	5.7	6.1	6.1	6.2	50S ribosomal protein L2, putative	mRPL2
Up	PF3D7_1009400	0.611334	6.14E-02	5.2	5.5	5.5	5.8	5.9	6.1	zinc finger protein, putative	ZNF2
Up	PF3D7_1333000	0.61118	9.25E-02	5.3	5.3	4.8	5.7	5.6	5.8	20 kDa chaperonin	CPN20
Up	PF3D7_1357200	0.609882	3.82E-02	5.9	5.9	5.8	6.7	6.5	6	glutamate--tRNA ligase	GluRS
Up	PF3D7_1203000	0.605771	2.43E-04	8.3	8.3	7.9	8.7	8.9	8.8	origin recognition complex subunit 1	ORC1

Up	PF3D7_1343500	0.60455	2.32E-02	5.9	6.3	5.9	6.6	6.5	6.7	conserved protein, unknown function	N/A
Up	PF3D7_0217900	0.604118	7.71E-04	7.7	7.6	7.3	8.2	8.1	8	thioesterase/thiol ester dehydrase-isomerase, putative	N/A
Up	PF3D7_1475200	0.603934	2.64E-02	5.8	6	5.9	6.4	6.5	6.5	conserved protein, unknown function	N/A
Up	PF3D7_1408600	0.602628	3.63E-05	8.3	8.5	8.4	9	9.1	8.9	40S ribosomal protein S8e, putative	N/A
Up	PF3D7_1358800	0.602377	1.99E-03	7.7	7.6	7.5	8.3	8.3	7.9	40S ribosomal protein S15	RPS15
Up	PF3D7_1123400	0.60182	5.93E-04	7.5	7.6	7.4	8.1	8.2	8	eukaryotic peptide chain release factor GTP-binding subunit, putative	ERF3
Up	PF3D7_1239200	0.601308	2.28E-07	10	10	10	11	11	11	AP2 domain transcription factor, putative	ApiAP2
Up	PF3D7_1007700	0.600971	3.48E-06	9.9	10	9.8	11	11	11	AP2 domain transcription factor AP2-I	AP2-I
Up	PF3D7_0628700	0.600928	9.84E-02	5.1	5.4	5.2	5.4	5.8	6	conserved Plasmodium protein, unknown function	N/A
Up	PF3D7_1327800	0.596883	2.48E-02	6	6.3	5.8	6.5	6.7	6.7	ribose-phosphate pyrophosphokinase, putative	N/A
Up	PF3D7_1441400	0.596819	4.02E-03	7	7.2	6.8	7.6	7.8	7.5	FACT complex subunit SSRP1, putative	FACT-S
Up	PF3D7_0308000	0.596647	4.98E-02	5.5	5.4	6	6.2	6.2	6.3	DNA polymerase delta small subunit, putative	N/A
Up	PF3D7_0826700	0.596027	5.25E-05	8.4	8.3	8.3	8.9	9	8.9	receptor for activated c kinase	RACK1
Up	PF3D7_1240400	0.596017	2.47E-03	6.9	6.9	7	7.5	7.5	7.6	erythrocyte membrane protein 1, PfEMP1	VAR
Up	PF3D7_0810800	0.595051	7.38E-02	5.5	5.3	5.3	6	6.1	5.7	hydroxymethyldihydropterin pyrophosphokinase-dihydropteroate synthase	PPPK-DHPS
Up	PF3D7_1250800	0.593928	2.71E-04	8	8.3	7.9	8.6	8.7	8.6	DNA repair protein rhp16, putative	N/A
Up	PF3D7_0917600	0.593777	8.88E-03	6.6	6.4	6.6	7	7.3	7	pre-mRNA-splicing factor ATP-dependent RNA helicase PRP43, putative	PRP43
Up	PF3D7_1320800	0.593243	1.55E-02	6.1	6.3	6.4	6.7	6.9	6.9	dihydrolipoyllysine-residue succinyltransferase component of 2-oxoglutarate dehydrogenase complex	N/A
Up	PF3D7_1235700	0.590382	5.63E-03	7.1	7.3	6.7	7.6	7.6	7.7	ATP synthase subunit beta, mitochondrial	N/A
Up	PF3D7_1033400	0.589815	6.88E-03	6.8	6.6	6.6	7.3	7.3	7.1	haloacid dehalogenase-like hydrolase	HAD1

Up	PF3D7_1228700	0.58926	1.04E-04	8.3	8.4	8.2	8.8	8.9	8.9	conserved Plasmodium protein, unknown function	N/A
Up	PF3D7_1307200	0.589013	2.40E-02	6.1	6.5	6	6.7	6.8	6.8	DnaJ protein, putative	N/A
Up	PF3D7_1359200	0.588949	2.09E-02	6.2	6.2	6.2	6.5	6.7	7	high mobility group protein B4, putative	HMGB4
Up	PF3D7_1333900	0.588452	1.42E-02	6.5	6.2	6.6	7.2	7	6.9	conserved Plasmodium protein, unknown function	N/A
Up	PF3D7_1451800	0.587031	9.78E-05	9	9.3	8.9	9.6	9.7	9.6	sortilin	SORTLR
Up	PF3D7_1106000	0.586682	1.72E-02	6.3	6.2	6.2	6.7	7	6.7	RuvB-like helicase 2	RUVB2
Up	PF3D7_0516900	0.585118	4.10E-04	8.2	8.4	8.2	8.9	9	8.6	60S ribosomal protein L2	RPL2
Down	PF3D7_0425400	-8.77703	1.95E-11	7.4	6.7	7.7	2.3	2	2	Plasmodium exported protein (PHISTa), unknown function	N/A
Down	PF3D7_1479100	-7.14146	1.99E-07	4.7	4.7	5.2	2	2	2	Plasmodium exported protein, unknown function	N/A
Down	PF3D7_1479200	-6.66339	2.08E-06	4.6	4.6	4.2	2	2	2	Plasmodium exported protein (PHISTa), unknown function	N/A
Down	PF3D7_0400700	-6.64911	9.66E-07	5.1	5.4	5.4	2	2.3	2	rifin	RIF
Down	PF3D7_0808900	-6.22722	5.31E-11	6.6	6.3	6	2.6	2.3	2	rifin	RIF
Down	PF3D7_0424800	-6.09087	9.58E-16	7.6	7.1	8.2	3.3	2.3	2.6	Plasmodium exported protein (PHISTb), unknown function	N/A
Down	PF3D7_0222300	-5.9006	8.01E-05	4.2	3.6	3.8	2	2	2	exported protein family 4, pseudogene	EPF4
Down	PF3D7_0114500	-5.6221	9.17E-05	4.5	4.7	4	2	2.3	2	Plasmodium exported protein (hyp10), unknown function	N/A
Down	PF3D7_0223400	-5.61097	2.50E-04	3.7	3.6	3.8	2	2	2	rifin	RIF
Down	PF3D7_0808800	-5.25889	1.02E-07	5.5	5.4	5.2	2.6	2.3	2	rifin	RIF
Down	PF3D7_0400500	-5.18814	2.37E-05	4.9	4.9	4.7	2.3	2.3	2	rifin	RIF
Down	PF3D7_0631500	-5.17802	1.36E-03	3.5	3.3	3.5	2	2	2	exported protein family 3	EPF3
Down	PF3D7_1041100	-4.94347	3.19E-03	3.3	3.5	3	2	2	2	rifin	RIF
Down	PF3D7_1479000	-4.79217	6.69E-84	9.1	8.9	9.1	5	4.3	4.1	acyl-CoA synthetase	ACS1a
Down	PF3D7_0424900	-4.69475	1.91E-10	7.8	7.2	8.3	2.8	3.5	4.4	Plasmodium exported protein (PHISTa), unknown function	N/A
Down	PF3D7_0223500	-4.6306	3.67E-09	5.5	5.4	5.6	2.3	2.6	2.6	erythrocyte membrane protein 1, PfEMP1	VAR
Down	PF3D7_0324300	-4.5834	4.06E-04	4.4	3.8	4.6	2.3	2.3	2	exported protein family 4	EPF4

Down	PF3D7_1254200	-4.45766	6.67E-04	3.9	4.5	4.2	2	2	2.6	rifin	RIF
Down	PF3D7_1041200	-4.41326	2.74E-13	6.1	6.4	5.6	2.8	3	2.6	rifin	RIF
Down	PF3D7_0401900	-4.36821	8.42E-129	11	10	11	6.5	6	6.4	acyl-CoA synthetase	ACS6
Down	PF3D7_0425100	-4.07743	2.38E-21	6.7	6.5	7.2	4	3	3	Plasmodium exported protein (hyp6), unknown function	N/A
Down	PF3D7_0601100	-4.06413	1.66E-02	3.5	3.6	2.8	2.3	2	2	exported protein family 3	EPF3
Down	PF3D7_0532700	-3.99831	2.74E-04	4.6	4.3	4	2.3	2.6	2	erythrocyte membrane protein 1 (PfEMP1), pseudogene	VAR
Down	PF3D7_0425500	-3.95602	1.97E-14	6.1	6.4	5.8	3	3.2	3	stevor	N/A
Down	PF3D7_0101100	-3.73563	1.27E-03	4.5	3.3	4.2	2.3	2	2.6	exported protein family 4	EPF4
Down	PF3D7_0631900	-3.70156	3.88E-02	3.2	3.3	2.8	2.3	2	2	stevor	N/A
Down	PF3D7_0631600	-3.70122	4.74E-02	2.8	3.8	2.3	2	2.3	2	exported protein family 4	EPF4
Down	PF3D7_0400300	-3.50916	1.90E-08	5.3	5.7	5	2.6	3	3	rifin	RIF
Down	PF3D7_1401000	-3.49012	4.67E-04	4.2	4.4	4.2	2.6	2.6	2	GBPH protein	GBPH
Down	PF3D7_1039100	-3.40275	5.75E-13	5.9	6.1	6	3.5	3	3.3	DnaJ protein, putative, pseudogene	N/A
Down	PF3D7_1373500	-3.34949	1.66E-57	8.5	8.8	8.4	5.2	5.5	5.3	erythrocyte membrane protein 1, PfEMP1	VAR
Down	PF3D7_0732900	-3.32033	6.45E-05	4.6	4.8	4.2	2.6	2	3	rifin	RIF
Down	PF3D7_0301000	-3.2837	2.09E-03	3.8	3.5	4.7	2	2.6	2.6	acyl-CoA synthetase	ACS2
Down	PF3D7_1000500	-3.27127	7.34E-03	3.3	4	3.9	2.6	2.3	2	rifin	RIF
Down	PF3D7_0425200	-3.26254	3.37E-06	7.3	7.1	7.6	4.8	4.7	3	Plasmodium exported protein (hyp15), unknown function	N/A
Down	PF3D7_1300500	-3.21342	2.81E-05	4.9	4.7	4.4	2.8	2.6	2.6	rifin	RIF
Down	PF3D7_0631400	-3.16872	4.53E-02	3.7	2.8	3.3	2.6	2	2	Pfmc-2TM Maurer's cleft two transmembrane protein	MC-2TM
Down	PF3D7_0114100	-3.0573	1.52E-02	3.8	3.7	3.3	2.6	2.3	2	Pfmc-2TM Maurer's cleft two transmembrane protein	MC-2TM
Down	PF3D7_0425600	-2.99828	1.94E-02	3.3	4	3.3	2.3	2	2.6	rifin, pseudogene	RIF
Down	PF3D7_0302200	-2.94061	1.68E-65	12	12	12	8.8	8.6	9.4	cytoadherence linked asexual protein 3.2	CLAG3.2
Down	PF3D7_0900200	-2.93708	2.18E-02	3.6	3.5	3.6	2.3	2	2.6	rifin	RIF
Down	PF3D7_1248800	-2.90549	7.74E-02	3	3	3.5	2.6	2	2	conserved Plasmodium protein, unknown function	N/A

Down	PF3D7_0214500	-2.90543	7.73E-02	3.5	2.6	3.3	2.3	2.3	2	conserved Plasmodium protein, unknown function	N/A
Down	PF3D7_0701400	-2.75341	1.49E-02	3.7	3.6	3.8	2.6	2	2.6	exported protein family 4, pseudogene	EPF4
Down	PF3D7_1373400	-2.72084	6.19E-03	3.9	4	3.8	2.6	2.3	2.6	rifin	RIF
Down	PF3D7_0936700	-2.70633	1.67E-06	5.2	4.9	5.5	3.5	2.6	3.3	lysophospholipase, putative	N/A
Down	PF3D7_1101700	-2.69893	1.85E-02	3.5	3.6	3.9	2.6	2	2.6	Pfmc-2TM Maurer's cleft two transmembrane protein	MC-2TM
Down	PF3D7_1461500	-2.66136	5.29E-02	3	3.2	3.8	2.6	2.3	2	meiotic nuclear division protein 1, putative	MND1
Down	PF3D7_0400600	-2.58347	6.59E-02	3	3	3.8	2.3	2	2.6	erythrocyte membrane protein 1 (PfEMP1), exon 1, pseudogene	N/A
Down	PF3D7_0324100	-2.58307	2.84E-02	3.6	3.7	3.5	2.3	2.8	2	Pfmc-2TM Maurer's cleft two transmembrane protein	MC-2TM
Down	PF3D7_0900100	-2.57382	4.05E-16	6.8	6.8	6.4	4.2	4.4	4.5	erythrocyte membrane protein 1, PfEMP1	VAR
Down	PF3D7_0733000	-2.51763	1.72E-16	6.7	7.1	6.8	4.9	4.8	3.9	erythrocyte membrane protein 1, PfEMP1	VAR
Down	PF3D7_0936500	-2.50067	7.76E-02	3.5	3.5	2.8	2.3	2.6	2	virulence-associated protein 1	VAP1
Down	PF3D7_0114300	-2.50042	8.31E-02	3	3.2	3.6	2	2.8	2	exported protein family 4, pseudogene	EPF4
Down	PF3D7_1200200	-2.49419	1.84E-07	5.8	5.9	5.2	4	2.6	3.9	rifin	RIF
Down	PF3D7_0324800	-2.49403	3.46E-08	5.6	5.9	5.4	3.9	3.3	3.6	rifin	RIF
Down	PF3D7_1240600	-2.45548	5.62E-15	6.8	6.4	7	4.1	4.6	4.7	erythrocyte membrane protein 1, PfEMP1	VAR
Down	PF3D7_0533100	-2.32699	2.06E-25	8	8.8	8.3	6.1	6.3	6.2	erythrocyte membrane protein 1 (PfEMP1), pseudogene	VAR1CSA
Down	PF3D7_1139000	-2.24643	8.72E-02	3.2	2.6	4	2.3	2.3	2.6		
Down	PF3D7_0600600	-2.24616	7.52E-02	3.2	3.5	3.5	2.6	2.6	2	erythrocyte membrane protein 1 (PfEMP1), exon 2	VAR
Down	PF3D7_1107600	-2.24614	8.27E-02	2.8	3.3	3.8	2.6	2.6	2	conserved Plasmodium protein, unknown function	N/A
Down	PF3D7_0832100	-2.23209	1.01E-13	6.7	7	7.3	4.4	4.8	5.5	rifin, pseudogene	RIF
Down	PF3D7_1372200	-2.22375	1.50E-06	6.8	5.4	5.4	4.5	3.7	4.1	histidine-rich protein III	HRPIII
Down	PF3D7_1476600	-2.22067	2.12E-20	7.2	7.5	7.4	5.3	5.3	5.2	Plasmodium exported protein, unknown function	N/A
Down	PF3D7_0101300	-2.20005	5.45E-02	3.5	3.7	3.5	2.3	2	3	Pfmc-2TM Maurer's cleft two transmembrane protein	MC-2TM

Down	PF3D7_1039900	-2.19981	9.77E-03	3	4.5	4.8	2.6	2.6	3.3	exported protein family 4	EPF4
Down	PF3D7_0500100	-2.15284	1.25E-32	8.5	8.8	8.4	6.4	6.5	6.5	erythrocyte membrane protein 1, PfEMP1	VAR
Down	PF3D7_1219400	-2.05714	5.77E-02	3.6	3.2	4	2.6	3	2	erythrocyte membrane protein 1 (PfEMP1), pseudogene	VAR
Down	PF3D7_0800750	-2.03084	1.02E-02	3.9	3.7	4.8	3.2	3	2.6	Plasmodium exported protein, unknown function, pseudogene	N/A
Down	PF3D7_0500600	-1.99812	9.01E-04	4.6	5.2	4.4	2.8	3.5	3.6	stevor, pseudogene	N/A
Down	PF3D7_1478000	-1.95095	1.67E-18	9.2	8.5	9.3	7	6.7	7.5	Plasmodium exported protein (PHISTa), unknown function	GEXP17
Down	PF3D7_1477000	-1.94624	9.85E-03	5.1	4.3	3.6	3.2	2.3	3.6	Plasmodium exported protein (hyp17), unknown function, pseudogene	N/A
Down	PF3D7_1465600	-1.937	8.23E-02	3.2	3	4.2	2.6	2.6	2.6	conserved Plasmodium protein, unknown function	N/A
Down	PF3D7_1302400	-1.81937	5.65E-03	4.3	4.5	4.5	3.3	3.2	3	conserved protein, unknown function	N/A
Down	PF3D7_0800800	-1.78452	8.60E-07	5.7	5.9	6.1	4.5	4.2	4.2	Plasmodium exported protein (hyp7), unknown function	N/A
Down	PF3D7_0711700	-1.76166	3.25E-05	5.5	5.3	5.8	3.7	4	4.4	erythrocyte membrane protein 1, PfEMP1	VAR
Down	PF3D7_0402900	-1.75916	3.67E-03	4.7	4.4	4.7	3.2	3.5	3.3	probable protein, unknown function	N/A
Down	PF3D7_1203800	-1.74521	1.72E-02	4	4.6	4.2	2.8	3.7	2.6	non-coding RNA	N/A
Down	PF3D7_0731000	-1.73259	7.71E-17	8.3	8.3	8.6	7.1	6.4	6.5	non-coding RNA	N/A
Down	PF3D7_0936000	-1.67048	1.19E-08	7.7	6.6	7.8	5.9	5.7	6	ring-exported protein 2	REX2
Down	PF3D7_0629600	-1.66042	4.96E-08	6.5	6.2	6.6	5.1	4.9	4.6	ribosomal RNA-processing protein 7, putative	N/A
Down	PF3D7_1102900	-1.65049	5.36E-03	4.7	4.2	5.1	3.3	3.5	3.6	Plasmodium exported protein (hyp11), unknown function	N/A
Down	PF3D7_0630200	-1.62671	5.86E-02	4	3.5	4.2	3.3	2.3	3	secreted ookinete protein, putative	PSOP6
Down	PF3D7_0112900	-1.62353	2.82E-03	7.3	7	7.4	6.2	5.7	4.6	Plasmodium exported protein, unknown function	N/A
Down	PF3D7_1206400	-1.61147	1.57E-02	4.3	4	4.8	3.5	3	3.3	thiosulfate sulfurtransferase, putative	TUM1
Down	PF3D7_1401100	-1.5989	4.57E-17	11	10	11	9.4	8.9	8.9	DnaJ protein, putative	N/A
Down	PF3D7_0832000	-1.58338	7.64E-02	3.5	3.7	4.2	3	2.6	3	stevor	N/A

Down	PF3D7_1301400	-1.56887	3.02E-03	5.9	4.8	4.5	4.1	4	3.6	Plasmodium exported protein (hyp12), unknown function	HYP12
Down	PF3D7_1352900	-1.55541	2.14E-13	9.3	8.7	9.5	7.8	7.3	7.9	Plasmodium exported protein, unknown function	N/A
Down	PF3D7_1109100	-1.55322	7.52E-02	3.9	3	5.2	2.8	2.6	3.9	conserved Plasmodium protein, unknown function	N/A
Down	PF3D7_0900400	-1.54584	5.01E-04	5.6	5.2	5.3	3.5	4.1	4.5	rifin	RIF
Down	PF3D7_0702000	-1.52926	1.21E-13	8.9	8.4	9.2	7.4	7.1	7.5	Plasmodium exported protein (hyp12), unknown function	N/A
Down	PF3D7_1300600	-1.52614	1.16E-05	6.1	5.9	5.7	4.6	4.5	4.6	rifin	RIF
Down	PF3D7_1367800	-1.51288	6.08E-02	3.6	3.6	4.8	2.8	3.2	3.3	secreted ookinete protein, putative	PSOP2
Down	PF3D7_1301700	-1.50757	1.56E-05	6.7	5.7	6.3	4.7	4.6	5.2	CX3CL1-binding protein 2	CBP2
Down	PF3D7_0702300	-1.50021	3.20E-10	7.8	7.2	8.1	6.2	6.2	6.4	sporozoite threonine and asparagine-rich protein	STARP
Down	PF3D7_0202200	-1.48956	1.08E-12	8.7	8	8.7	7.1	6.8	7.2	EMP1-trafficking protein	PTP1
Down	PF3D7_0414800	-1.48388	2.01E-19	9.6	9.4	9.7	8	7.8	8.4	conserved Plasmodium protein, unknown function	N/A
Down	PF3D7_0830600	-1.48371	2.08E-03	8.3	7.7	8.6	6.6	6.3	7.3	Plasmodium exported protein (PHISTc), unknown function	N/A
Down	PF3D7_1478800	-1.47184	1.56E-06	6.5	6.3	6.6	5.6	4.7	4.9	Plasmodium exported protein, unknown function	N/A
Down	PF3D7_1370300	-1.45672	7.53E-06	7.1	6.2	6.4	5	4.9	5.7	membrane associated histidine-rich protein 1	MAHRP1
Down	PF3D7_0935900	-1.45403	7.71E-04	11	9.9	11	9	8.8	9.5	ring-exported protein 1	REX1
Down	PF3D7_0701900	-1.44049	2.00E-17	11	11	11	9.8	9.4	9.6	Plasmodium exported protein, unknown function	N/A
Down	PF3D7_0936300	-1.42723	3.98E-03	8.4	7.6	8.6	6.7	6.4	7.4	ring-exported protein 3	REX3
Down	PF3D7_1404700	-1.42474	4.48E-11	7.8	7.4	8	6.5	6.2	6.4	cysteine-rich small secreted protein CSS, putative	N/A
Down	PF3D7_0702600	-1.42077	1.55E-02	4.7	4.6	4.8	4.2	3.3	3	chitinase, fragment	N/A
Down	PF3D7_1351800	-1.41814	1.44E-08	7.5	7.1	7.8	6.4	6.1	5.7	conserved Plasmodium protein, unknown function	N/A
Down	PF3D7_0900500	-1.40343	1.65E-06	6.3	6.6	6.2	5.2	4.9	5.1	rifin	RIF
Down	PF3D7_1353200	-1.4007	3.50E-06	7.1	6.3	7	5.5	5	5.9	membrane associated histidine-rich protein 2	MAHRP2

Down	PF3D7_0628400	-1.37038	3.93E-03	5.2	4.9	5	4	3.9	3.9	protease, putative	N/A
Down	PF3D7_1318900	-1.36613	6.56E-03	4.9	4.7	5.2	3.9	3.7	3.9	conserved Plasmodium protein, unknown function	N/A
Down	PF3D7_0219700	-1.36465	8.15E-03	7.3	6.7	7.9	6.2	5.5	6.3	Plasmodium exported protein (PHISTc), unknown function	GEXP20
Down	PF3D7_1207700	-1.36375	1.45E-09	7.4	7.4	7.6	6.5	6	5.9	41-3 protein	N/A
Down	PF3D7_0424500	-1.35978	2.10E-10	9.4	8.9	9.6	8.3	7.7	7.8	serine/threonine protein kinase, FIKK family	FIKK4.1
Down	PF3D7_1149600	-1.35901	3.55E-16	10	9.8	10	8.9	8.5	8.6	DnaJ protein, putative	N/A
Down	PF3D7_1310300	-1.35769	3.16E-09	7.3	7.2	7.7	6.2	6.1	6	zinc finger protein, putative	N/A
Down	PF3D7_1329800	-1.35623	5.38E-08	7.1	6.9	7.1	5.7	5.4	6.1	conserved Plasmodium protein, unknown function	N/A
Down	PF3D7_1321000	-1.35312	2.48E-03	4.7	5.6	6.1	4.5	4.2	4.5	conserved Plasmodium protein, unknown function	N/A
Down	PF3D7_0726100	-1.35227	8.11E-08	8.1	7.5	8.3	6.9	6.1	6.8	Plasmodium exported protein, unknown function	N/A
Down	PF3D7_1001500	-1.34764	1.05E-09	9.3	8.5	9.3	8	7.4	7.8	early transcribed membrane protein 10.1	ETRAMP10
Down	PF3D7_0502400	-1.33376	7.39E-10	8.2	7.8	8.6	7	6.9	6.9	merozoite surface protein 8	MSP8
Down	PF3D7_0424600	-1.33142	2.18E-13	12	11	12	11	10	9.8	Plasmodium exported protein (PHISTb)	N/A
Down	PF3D7_0628100	-1.32655	9.91E-04	15	15	15	13	13	14	HECT domain-containing protein 1, putative	HECT1
Down	PF3D7_1300200	-1.32026	5.29E-02	4.2	4.3	4.2	3.2	3.2	3.6	rifin	RIF
Down	PF3D7_0808700	-1.30959	2.36E-10	7.5	7.5	7.5	6	6.3	6.4	erythrocyte membrane protein 1, PfEMP1	VAR
Down	PF3D7_1372300	-1.30823	7.75E-04	6	5.3	6.4	4.7	4.4	5.2	Plasmodium exported protein (PHIST), unknown function	N/A
Down	PF3D7_1102800	-1.30733	1.95E-14	14	14	14	13	12	12	early transcribed membrane protein 11.2	ETRAMP11.2
Down	PF3D7_0702100	-1.30443	1.35E-11	10	9.8	10	9.1	8.7	8.4	Plasmodium exported protein (PHISTb), unknown function, pseudogene	N/A
Down	PF3D7_0902500	-1.30398	6.05E-12	9.3	8.8	9.5	8.2	7.7	7.9	serine/threonine protein kinase, FIKK family	FIKK9.6
Down	PF3D7_0623800	-1.29877	1.21E-16	12	12	12	11	11	11	tyrosine kinase-like protein, putative	TKL4
Down	PF3D7_1411100	-1.29734	5.19E-16	10	9.8	10	8.9	8.5	8.8	conserved Plasmodium membrane protein, unknown function	N/A
Down	PF3D7_1014000	-1.29634	2.09E-05	6.3	6.1	6.2	5	5	5	GDP-L-fucose synthase	FS

Down	PF3D7_0930200	-1.29367	3.98E-15	11	10	11	9.5	9.1	9.6	leucine-rich repeat protein	LRR8
Down	PF3D7_1121800	-1.29136	6.09E-06	6.4	6.3	6.9	5.2	5.5	5.4	peptidase M16, putative	N/A
Down	PF3D7_1148900	-1.28611	6.67E-04	6	5.3	6.2	4.7	4.3	5.1	Plasmodium exported protein, unknown function	N/A
Down	PF3D7_1021700	-1.28418	1.73E-04	14	14	14	13	12	13	VPS13 domain-containing protein, putative	N/A
Down	PF3D7_0104300	-1.28054	2.03E-18	14	13	14	13	12	12	ubiquitin carboxyl-terminal hydrolase 1, putative	UBP1
Down	PF3D7_1201200	-1.27746	5.31E-13	10	9.6	9.9	8.9	8.4	8.4	Plasmodium exported protein (PHISTa-like), unknown function	N/A
Down	PF3D7_0402300	-1.27497	1.20E-14	14	13	14	12	12	13	reticulocyte binding protein homologue 1	RH1
Down	PF3D7_1016800	-1.26663	8.84E-18	10	10	11	9.5	9.3	9.3	Plasmodium exported protein (PHISTc), unknown function	N/A
Down	PF3D7_0525300	-1.26476	3.26E-08	7.4	7.1	7.8	6.1	6.2	6.4	conserved protein, unknown function	N/A
Down	PF3D7_0402100	-1.2617	1.71E-04	12	12	12	11	10	10	Plasmodium exported protein (PHISTb), unknown function	N/A
Down	PF3D7_1404800	-1.26078	1.34E-11	11	11	11	10	9.5	9.6	conserved Plasmodium protein, unknown function	N/A
Down	PF3D7_0805200	-1.25843	1.71E-02	10	10	11	9.7	9.2	8.2	gamete release protein, putative	GAMER
Down	PF3D7_1002000	-1.25264	4.30E-05	6.8	5.9	6.6	5.2	5.4	5.5	Plasmodium exported protein (hyp2), unknown function	N/A
Down	PF3D7_1149200	-1.25115	5.78E-14	14	14	15	13	13	13	ring-infected erythrocyte surface antigen	RESA3
Down	PF3D7_1411000	-1.24246	6.47E-16	12	12	13	11	11	11	conserved Plasmodium protein, unknown function	N/A
Down	PF3D7_1302200	-1.24072	8.48E-03	4.8	4.9	5.6	4.2	4.1	4.1	protein UIS3	UIS3
Down	PF3D7_0219900	-1.23689	2.62E-08	7.9	7.8	8.4	7.1	6.9	6.4	Plasmodium exported protein, unknown function	N/A
Down	PF3D7_0315900	-1.23609	2.35E-04	6.3	5.6	6.2	5.1	4.7	5	conserved Plasmodium protein, unknown function	N/A
Down	PF3D7_0505300	-1.23343	6.50E-04	5.9	5.5	5.8	4.9	4.7	4.4	UDP-N-acetylglucosamine transporter, putative	N/A
Down	PF3D7_0818300	-1.22659	6.97E-04	6	5.7	6	5.1	4.1	5.1	dynactin subunit 6, putative	N/A
Down	PF3D7_0400400	-1.22552	5.81E-03	5.5	5.2	5	4.6	4.1	3.9	erythrocyte membrane protein 1, PfEMP1	VAR
Down	PF3D7_0814500	-1.2223	1.61E-07	8	7.4	8.2	7	6.4	6.7	conserved protein, unknown function	N/A

Down	PF3D7_1337800	-1.22159	1.10E-18	11	11	11	9.9	9.5	9.7	calcium-dependent protein kinase 5	CDPK5
Down	PF3D7_1335500	-1.22085	1.15E-02	5.3	4.7	5.2	3.8	3.8	4.5	DNA replication complex GINS protein, putative	N/A
Down	PF3D7_1200700	-1.22053	9.63E-10	11	11	12	10	9.8	10	acyl-CoA synthetase	ACS7
Down	PF3D7_1102700	-1.2127	1.21E-13	12	12	12	11	11	10	early transcribed membrane protein 11.1	ETRAMP11.1
Down	PF3D7_0731400	-1.21156	3.92E-13	9.8	9.3	9.6	8.5	8.2	8.5	serine/threonine protein kinase, FIKK family, pseudogene	FIKK7.2
Down	PF3D7_0301800	-1.21144	5.06E-11	8.9	8.7	9.3	8	7.7	7.6	Plasmodium exported protein, unknown function	N/A
Down	PF3D7_0202500	-1.20376	7.02E-04	13	13	13	12	12	11	early transcribed membrane protein 2	ETRAMP2
Down	PF3D7_0113300	-1.20071	2.43E-04	6.4	5.9	6.7	5	4.9	5.7	Plasmodium exported protein (hyp1), unknown function	N/A
Down	PF3D7_1149300	-1.19841	1.13E-06	7.2	6.8	7.4	6.3	5.9	5.9	serine/threonine protein kinase, FIKK family	FIKK11
Down	PF3D7_0935600	-1.19688	1.34E-11	11	11	11	10	9.8	10	gametocytogenesis-implicated protein	GIG
Down	PF3D7_0902300	-1.19111	9.93E-06	6.9	6.4	7.1	5.7	5.5	5.9	serine/threonine protein kinase, FIKK family	FIKK9.4
Down	PF3D7_0507300	-1.1905	4.00E-10	9.7	9.5	10	8.5	8.2	8.9	subtilisin-like ookinete protein SOPT	SOPT
Down	PF3D7_0730800	-1.18494	1.08E-09	11	10	11	9.5	9.1	9.6	Plasmodium exported protein, unknown function	N/A
Down	PF3D7_0731200	-1.18351	8.44E-08	8.2	8.1	8.2	7.5	6.9	6.5	Plasmodium exported protein, unknown function	N/A
Down	PF3D7_0831500	-1.18146	3.92E-11	9.4	9.1	9.6	8.5	8	8.1	Plasmodium exported protein (PHIST), unknown function	N/A
Down	PF3D7_1016700	-1.18069	2.08E-06	8.2	7.6	8.5	7.2	6.5	7.1	Plasmodium exported protein (PHISTc), unknown function	N/A
Down	PF3D7_1446300	-1.1794	4.37E-15	10	10	11	9.2	9	9.1	conserved Plasmodium membrane protein, unknown function	N/A
Down	PF3D7_0424200	-1.17566	6.27E-13	12	12	12	11	11	11	reticulocyte binding protein homologue 4	RH4
Down	PF3D7_1409500	-1.17316	2.88E-07	7.4	7.3	7.7	6.3	6	6.7	conserved Plasmodium protein, unknown function	N/A
Down	PF3D7_0621350	-1.17276	1.99E-02	4.6	4.9	5.2	3.9	4.1	3.9	protein SYS1, putative	SYS1
Down	PF3D7_0625400	-1.17159	7.25E-14	9.5	9.3	9.6	8.5	8.2	8.3	conserved Plasmodium protein, unknown function	N/A
Down	PF3D7_1411800	-1.16837	7.73E-03	5	5.3	5.8	4.7	3.9	4.5	conserved Plasmodium protein, unknown function	N/A

Down	PF3D7_1425500	-1.16837	1.56E-03	5.7	5.7	6.1	4.7	4.3	5.2	conserved Plasmodium protein, unknown function	N/A
Down	PF3D7_0800900	-1.16829	9.25E-02	4.4	3.8	4.5	3.5	3.7	3	Plasmodium exported protein (hyp7), unknown function, pseudogene	N/A
Down	PF3D7_0522400	-1.16155	3.82E-04	13	13	13	12	11	12	conserved Plasmodium protein, unknown function	N/A
Down	PF3D7_1001100	-1.15917	2.65E-07	8.3	7.6	8.5	6.9	6.8	7.3	acyl-CoA binding protein, isoform 1, ACBP1	ACBP1
Down	PF3D7_0702200	-1.15748	1.82E-11	10	9.9	10	9.3	8.9	8.7	lysophospholipase LPL20	LPL20
Down	PF3D7_0318700	-1.15703	1.38E-15	9.5	9.4	9.6	8.5	8.2	8.4	conserved Plasmodium protein, unknown function	N/A
Down	PF3D7_1331400	-1.15698	4.19E-10	9.2	8.8	9	7.6	7.7	8.2	CPW-WPC family protein	N/A
Down	PF3D7_1133700	-1.1554	4.21E-09	8.6	8.1	8.9	7.5	7.4	7.4	FHA domain-containing protein, putative	N/A
Down	PF3D7_0831800	-1.15064	4.40E-08	9.1	8.5	9	7.6	7.4	8.2	histidine-rich protein II	HRP2
Down	PF3D7_1105600	-1.14972	3.82E-09	11	11	11	11	9.9	9.6	translocon component PTEX88	PTEX88
Down	PF3D7_0215300	-1.14536	4.26E-08	8.5	8.3	8.6	7.8	7.2	7	acyl-CoA synthetase	ACS8
Down	PF3D7_0935700	-1.1425	2.55E-08	8.4	8.3	8.8	7.3	7	7.8	Plasmodium exported protein, unknown function	N/A
Down	PF3D7_1436300	-1.14135	7.25E-14	13	13	13	12	12	12	translocon component PTEX150	PTEX150
Down	PF3D7_0424100	-1.13459	1.74E-10	11	11	11	10	9.8	10	reticulocyte binding protein homologue 5	RH5
Down	PF3D7_1252400	-1.13336	3.92E-11	14	14	14	13	12	13	reticulocyte binding protein homologue 3, pseudogene	RH3
Down	PF3D7_0200100	-1.1296	8.78E-02	4.3	4.3	4.2	3.6	3.5	3.3	erythrocyte membrane protein 1, PfEMP1	VAR
Down	PF3D7_0109100	-1.12694	3.20E-10	9.4	9.2	9.3	8.1	7.9	8.6	LCCL domain-containing protein	CCp5
Down	PF3D7_1478900	-1.12494	1.27E-14	12	12	12	11	11	11	non-coding RNA	N/A
Down	PF3D7_0629500	-1.12417	6.48E-13	9.9	9.7	10	8.9	8.7	8.8	amino acid transporter AAT1	AAT1
Down	PF3D7_0318600	-1.12139	1.83E-09	8.7	8.5	9	7.6	7.3	7.8	cleavage and polyadenylation specificity factor, putative	N/A
Down	PF3D7_1200800	-1.11619	2.28E-05	6.7	6.4	6.9	5.7	5.8	5.5	serine/threonine protein kinase, FIKK family	FIKK12
Down	PF3D7_1401400	-1.11414	3.92E-11	11	11	12	11	10	10	early transcribed membrane protein 14.1	ETRAMP14
Down	PF3D7_1218100	-1.11409	2.21E-03	6.3	5.5	6.3	5.2	4.5	5.5	conserved Plasmodium protein, unknown function	N/A

Down	PF3D7_1351600	-1.11361	5.79E-10	8.2	7.9	8.1	7	6.8	7.1	glycerol kinase	GK
Down	PF3D7_1240900	-1.11269	1.03E-16	9.8	9.8	9.7	8.5	8.6	8.8	erythrocyte membrane protein 1, PfEMP1	VAR
Down	PF3D7_0424700	-1.11181	1.41E-05	7.9	7	7.4	6.5	6.2	6.5	serine/threonine protein kinase, FIKK family	FIKK4.2
Down	PF3D7_0830500	-1.10313	1.82E-11	11	11	12	10	10	10	sporozoite and liver stage tryptophan-rich protein, putative	TryThrA
Down	PF3D7_0301300	-1.1026	2.48E-09	8.1	8.1	8.2	7.2	6.7	7.2	epoxide hydrolase 1	EH1
Down	PF3D7_1148800	-1.09974	2.50E-04	6.2	6.1	6.5	5	5.3	5.5	Plasmodium exported protein (hyp11), unknown function	N/A
Down	PF3D7_1477800	-1.09946	2.52E-06	7.5	7.8	8	7	6.8	6.2	acyl-CoA binding protein	ACBP
Down	PF3D7_0301600	-1.09621	3.60E-07	7.7	7.3	7.8	6.5	6.4	6.8	Plasmodium exported protein (hyp1), unknown function	GEXP21
Down	PF3D7_1115800	-1.09503	5.81E-12	11	11	11	10	9.9	9.8	conserved Plasmodium protein, unknown function	N/A
Down	PF3D7_0525900	-1.09458	5.08E-02	4.6	4.5	4.7	3.9	3.8	3.6	NIMA related kinase 2	NEK2
Down	PF3D7_1416500	-1.09327	2.66E-08	9.9	9.8	10	9.3	8.8	8.3	NADP-specific glutamate dehydrogenase	GDH1
Down	PF3D7_0102200	-1.08831	1.95E-11	15	15	16	14	14	14	ring-infected erythrocyte surface antigen	RESA
Down	PF3D7_1328600	-1.08809	2.53E-02	5.2	4.6	5.1	4.2	4	4.1	conserved Plasmodium protein, unknown function	N/A
Down	PF3D7_1343800	-1.08604	7.45E-11	13	13	13	11	11	12	VPS13 domain-containing protein, putative	N/A
Down	PF3D7_1428000	-1.08587	1.62E-03	5.8	5.7	6.2	5.1	4.8	4.9	conserved Plasmodium membrane protein, unknown function	N/A
Down	PF3D7_0824000	-1.08035	2.27E-03	5.9	5.8	5.9	4.7	4.5	5.3	conserved Plasmodium protein, unknown function	N/A
Down	PF3D7_1014100	-1.07513	6.96E-13	13	13	13	12	12	12	merozoite surface protein MSA180	MSA180
Down	PF3D7_1247600	-1.07477	1.72E-02	5.1	4.9	5.5	4.5	4.2	4.1	uroporphyrinogen-III synthase, putative	N/A
Down	PF3D7_1002100	-1.07421	5.78E-10	11	11	11	10	9.7	10	EMP1-trafficking protein	PTP5
Down	PF3D7_0403800	-1.06947	1.77E-13	12	11	12	11	10	11	alpha/beta hydrolase, putative	N/A
Down	PF3D7_1243400	-1.06319	1.78E-08	9.2	9.1	9.6	8.2	8	8.6	conserved Plasmodium protein, unknown function	N/A
Down	PF3D7_0202100	-1.06293	2.39E-09	11	11	12	11	10	9.9	liver stage associated protein 2	LSAP2
Down	PF3D7_1116800	-1.06285	4.17E-15	13	13	13	12	12	12	heat shock protein 101	HSP101

Down	PF3D7_1302700	-1.05951	1.11E-05	7.8	7.4	7.9	7.1	6.7	6.2	ATP-dependent RNA helicase DHR1, putative	N/A
Down	PF3D7_1428100	-1.05494	1.54E-10	8.7	8.5	8.9	7.7	7.7	7.7	WW domain-binding protein 11, putative	WBP11
Down	PF3D7_0207200	-1.05312	4.28E-05	7	6.8	6.9	6.1	6.1	5.4	iron-sulfur assembly protein, putative	ISCA1
Down	PF3D7_0318800	-1.04977	7.29E-03	5.7	5.3	6	4.5	4.5	5.2	triosephosphate isomerase, putative	N/A
Down	PF3D7_0415800	-1.04632	2.84E-09	9.8	9.6	10	8.7	8.4	9	PhIL1-interacting candidate PIC3	N/A
Down	PF3D7_1416400	-1.0435	7.48E-03	5.7	5.2	5.8	4.7	4.7	4.5	conserved protein, unknown function	N/A
Down	PF3D7_1371700	-1.04045	3.78E-12	8.9	8.9	9	8	7.8	8	serine/threonine protein kinase, FIKK family	FIKK13
Down	PF3D7_1216600	-1.0379	2.02E-02	5	5	5.8	4.7	4.5	4.1	cell traversal protein for ookinetes and sporozoites	CelTOS
Down	PF3D7_1413800	-1.03749	6.47E-07	8.4	8.1	8.6	7.2	7	7.7	diphthamide biosynthesis protein 1, putative	DPH1
Down	PF3D7_1149500	-1.03639	6.51E-02	7.4	6.5	7.5	6.2	5.5	6.6	ring-infected erythrocyte surface antigen 2, pseudogene	RESA2
Down	PF3D7_1120400	-1.03483	2.00E-03	6.1	5.7	6.5	5.4	5	5.2	alpha/beta hydrolase fold domain containing protein, putative	N/A
Down	PF3D7_1471000	-1.03285	6.56E-03	5.5	5.5	5.5	4.6	4.5	4.7	RNA 3'-terminal phosphate cyclase-like protein, putative	RCL1
Down	PF3D7_1312500	-1.03209	1.19E-08	8.5	8.2	8.4	7.5	7.4	7.1	conserved Plasmodium protein, unknown function	N/A
Down	PF3D7_0918700	-1.03144	9.16E-11	11	11	11	10	9.7	10	conserved Plasmodium protein, unknown function	N/A
Down	PF3D7_1252900	-1.03018	5.10E-03	6.2	5.6	6.9	5.6	4.9	5.5	Plasmodium exported protein, unknown function	N/A
Down	PF3D7_1315000	-1.02971	6.32E-04	6.3	6.2	6.5	5.7	5.3	5	conserved protein, unknown function	N/A
Down	PF3D7_0407300	-1.02864	2.97E-02	5.1	4.8	5.5	4.2	4.7	3.9	transcription factor, putative	N/A
Down	PF3D7_1455100	-1.02572	1.86E-07	8.3	8	8.6	7.5	7.2	7.3	protein tyrosine phosphatase, putative	PTP1
Down	PF3D7_1332100	-1.02472	4.00E-10	8.9	8.8	9	7.9	7.7	8.1	conserved protein, unknown function	N/A
Down	PF3D7_0501100	-1.02391	1.14E-10	10	9.7	10	9.1	8.7	8.8	co-chaperone J domain protein JDP	HSP40
Down	PF3D7_0220000	-1.02237	6.72E-12	14	14	14	13	13	13	liver stage antigen 3	LSA3
Down	PF3D7_1460100	-1.02184	1.49E-06	7.3	7.2	7.6	6.4	6.3	6.5	FYVE and coiled-coil domain-containing protein	FCP
Down	PF3D7_0620300	-1.02072	2.11E-09	8.8	8.7	9	7.9	7.6	8	conserved Plasmodium protein, unknown function	N/A

Down	PF3D7_1115700	-1.02041	6.35E-08	9.6	9.3	9.6	8.8	8.6	8	cysteine proteinase falcipain 2a	FP2A
Down	PF3D7_1240100	-1.01845	5.13E-08	9.4	9.4	9.5	8.8	8.3	8	early transcribed membrane protein 12	ETRAMP12
Down	PF3D7_1139700	-1.0176	8.79E-07	7.9	7.7	7.9	7	7	6.4	adrenodoxin reductase, putative	N/A
Down	PF3D7_0906600	-1.01415	4.14E-09	9.1	8.7	9	8.1	8	7.7	zinc finger protein, putative	N/A
Down	PF3D7_1472500	-1.01296	1.05E-03	5.9	6.2	6.2	5.3	5.2	5.1	conserved Plasmodium protein, unknown function	N/A
Down	PF3D7_1434500	-1.01291	3.78E-12	9.7	9.6	9.9	8.9	8.6	8.8	dynein-related AAA-type ATPase, putative	N/A
Down	PF3D7_1414200	-1.01268	2.09E-11	10	10	11	9.5	9.2	9.5	conserved Plasmodium protein, unknown function	N/A
Down	PF3D7_1407900	-1.01248	2.97E-07	9.4	8.7	9.4	8.2	8	8.4	plasmepsin I	PMI
Down	PF3D7_1329600	-1.00896	1.50E-08	8.5	8.3	8.8	7.6	7.5	7.4	conserved Plasmodium protein, unknown function	N/A
Down	PF3D7_1421100	-1.00842	1.69E-06	7.6	7.3	7.8	6.7	6.5	6.7	conserved Plasmodium protein, unknown function	N/A
Down	PF3D7_0725300	-1.00525	4.73E-08	8.1	8	8.3	7.2	7	7.3	conserved protein, unknown function	N/A
Down	PF3D7_0501300	-1.00385	8.60E-07	9.6	9	9.8	8.4	8.2	8.8	skeleton-binding protein 1	SBP1
Down	PF3D7_1427100	-1.00255	3.10E-08	9.5	9.1	9.7	8.7	8.3	8.4	lipase, putative	N/A
Down	PF3D7_1423400	-0.99834	4.82E-03	5.8	5.6	6.3	5.1	5.3	4.7	conserved Plasmodium membrane protein, unknown function	N/A
Down	PF3D7_0319700	-0.99675	3.94E-10	11	11	11	9.9	9.7	10	ABC transporter I family member 1, putative	ABCI3
Down	PF3D7_0402200	-0.99613	5.00E-10	13	13	14	12	12	13	surface-associated interspersed protein 4.1 (SURFIN 4.1), pseudogene	SURF4.1
Down	PF3D7_0926200	-0.99548	7.41E-05	7.3	6.9	7.6	6.5	5.9	6.6	conserved Plasmodium protein, unknown function	N/A
Down	PF3D7_1302000	-0.99083	1.95E-04	7	6.6	7.4	6.1	6.2	5.8	EMP1-trafficking protein	PTP6
Down	PF3D7_1420700	-0.9898	1.14E-10	13	12	13	12	12	12	surface protein P113	P113
Down	PF3D7_0905500	-0.98944	2.76E-08	8.8	8.5	8.8	7.9	7.4	7.9	conserved protein, unknown function	N/A
Down	PF3D7_0107500	-0.9876	7.36E-12	12	12	13	12	11	11	Niemann-Pick type C1-related protein	NCR1
Down	PF3D7_1142900	-0.98587	1.60E-05	7.1	7.2	7.5	6.6	6.2	6.2	conserved Plasmodium protein, unknown function	N/A
Down	PF3D7_0716100	-0.98461	2.27E-03	6.1	5.8	6.5	5.2	5.2	5.4	protein SDA1, putative	SDA1

Down	PF3D7_0104900	-0.98324	1.95E-03	6	6	6.2	5.3	5.2	4.9	RNase MRP	N/A
Down	PF3D7_1252200	-0.9831	9.66E-07	7.6	7.4	7.7	6.7	6.7	6.4	chitinase	CHT1
Down	PF3D7_1218500	-0.98286	1.80E-07	9.8	9.5	9.9	9.1	8.6	8.5	dynamin-like protein, putative	DRPC
Down	PF3D7_0805300	-0.98064	4.09E-08	11	11	12	11	10	11	zinc finger protein, putative	N/A
Down	PF3D7_1114200	-0.97953	5.63E-08	8.7	8.4	8.9	7.7	7.5	7.8	GTPase-activating protein, putative	N/A
Down	PF3D7_1119700	-0.97899	5.65E-09	8.6	8.4	8.5	7.6	7.3	7.7	conserved Plasmodium protein, unknown function	N/A
Down	PF3D7_1401500	-0.97829	6.79E-02	4.9	4.5	4.9	4.3	4	3.6	esterase, putative	N/A
Down	PF3D7_0108500	-0.97795	8.02E-09	11	11	11	10	9.8	9.9	ELM2 domain-containing protein, putative	N/A
Down	PF3D7_1036500	-0.9767	2.84E-09	12	12	12	11	11	11	conserved Plasmodium protein, unknown function	N/A
Down	PF3D7_1022600	-0.97594	4.63E-07	8.2	7.9	8.3	7.4	7.1	6.9	kelch protein K10	Kelch10
Down	PF3D7_1404900	-0.9752	1.14E-10	11	11	11	11	10	10	conserved Plasmodium protein, unknown function	N/A
Down	PF3D7_0824100	-0.97471	2.22E-02	5.5	5.6	5.3	4.6	5.1	3.9	3'-5' exonuclease, putative	N/A
Down	PF3D7_1366700	-0.97334	1.54E-03	6.4	5.9	6.6	5.3	5.3	5.6	conserved Plasmodium protein, unknown function	N/A
Down	PF3D7_0630100	-0.9729	6.32E-04	7.1	6.8	7.2	5.9	5.6	6.7	alpha/beta hydrolase, putative	N/A
Down	PF3D7_0713800	-0.97101	3.17E-11	9.4	9.2	9.3	8.3	8.2	8.5	negative elongation factor A, putative	NELFA
Down	PF3D7_0618100	-0.96773	1.46E-07	8.6	8.5	8.5	7.5	7.3	7.9	conserved Plasmodium protein, unknown function	N/A
Down	PF3D7_0605900	-0.96273	1.02E-07	9.5	9.3	9.5	8.8	8.4	8.1	elongation of fatty acids protein, putative	N/A
Down	PF3D7_0221700	-0.96183	3.75E-05	7.4	7.3	8	6.8	6.8	6.4	Plasmodium exported protein, unknown function	N/A
Down	PF3D7_1232600	-0.96137	3.43E-08	9	8.8	9.3	8.1	7.9	8.3	conserved Plasmodium protein, unknown function	N/A
Down	PF3D7_0206700	-0.96088	7.58E-11	9.4	9.2	9.2	8.3	8.1	8.5	adenylosuccinate lyase	ADSL
Down	PF3D7_0316000	-0.9586	2.41E-07	9.3	9	9.6	8.2	8.2	8.7	microneme associated antigen	MA
Down	PF3D7_1138700	-0.95692	3.05E-09	11	11	11	9.9	9.6	10	protein KIC5	KIC5
Down	PF3D7_1310400	-0.95622	2.09E-11	9.6	9.4	9.7	8.6	8.6	8.7	conserved Plasmodium protein, unknown function	N/A

Down	PF3D7_0210800	-0.95589	6.06E-03	5.8	5.6	6.2	5.1	4.7	5.2	conserved Plasmodium protein, unknown function	N/A
Down	PF3D7_0424300	-0.95505	4.48E-11	12	12	12	11	11	11	erythrocyte binding antigen-165, pseudogene	EBA165
Down	PF3D7_1037400	-0.95499	1.01E-06	8.1	8.3	8.7	7.3	7.4	7.7	conserved Plasmodium protein, unknown function	N/A
Down	PF3D7_1011500	-0.95462	5.31E-04	6.7	6.4	6.3	5.6	5.7	5.4	conserved protein, unknown function	N/A
Down	PF3D7_1431200	-0.95107	9.66E-07	8.3	8.1	8.4	7.6	7	7.4	OST-HTH associated domain protein, putative	N/A
Down	PF3D7_1457700	-0.95072	1.23E-05	7.7	7.3	7.9	6.9	6.5	6.7	large ribosomal subunit nuclear export factor, putative	N/A
Down	PF3D7_1462400	-0.94839	1.23E-06	9	8.5	9.2	8	7.7	8.2	conserved Plasmodium protein, unknown function	N/A
Down	PF3D7_1433500	-0.948	1.23E-09	13	13	13	12	12	12	DNA topoisomerase 2	TOP2
Down	PF3D7_0401800	-0.945	8.92E-12	9.9	10	10	9	9	9.3	Plasmodium exported protein (PHISTb), unknown function	PfD80
Down	PF3D7_0501400	-0.94286	1.05E-06	12	11	12	11	10	11	interspersed repeat antigen	FIRA
Down	PF3D7_0731500	-0.94125	5.35E-12	16	15	16	15	14	15	erythrocyte binding antigen-175	EBA175
Down	PF3D7_1418900	-0.94105	2.94E-04	7.5	7	7.8	6.4	6.3	6.9	ATP-dependent RNA helicase DBP4, putative	DBP4
Down	PF3D7_0530100	-0.93976	5.09E-09	9.4	9.4	9.9	8.7	8.5	8.7	SNARE protein, putative	SYN6
Down	PF3D7_0314100	-0.93883	2.25E-08	9	9	9.3	8.4	8.1	8.1	vesicle transport v-SNARE protein, putative	N/A
Down	PF3D7_0201700	-0.93775	7.08E-06	8.2	7.8	8.5	7.4	7.2	7.3	DnaJ protein, putative	N/A
Down	PF3D7_1428200	-0.93535	3.81E-11	11	11	11	9.9	9.7	10	major facilitator superfamily domain-containing protein, putative	MFS5
Down	PF3D7_0108900	-0.93286	1.32E-02	6.1	5.2	6.3	4.9	5.1	5.2	non-coding RNA	N/A
Down	PF3D7_0818600	-0.93239	6.77E-07	8.2	8	8.5	7.3	7.5	7.2	BEM46-like protein, putative	PBLP
Down	PF3D7_1243800	-0.93095	3.30E-08	10	10	10	9.1	8.9	9.5	WD repeat-containing protein 82, putative	WDR82
Down	PF3D7_0805600	-0.92941	6.96E-10	10	9.8	10	9.3	8.9	9	phosphatidic acid phosphatase 2	PAP2
Down	PF3D7_0628000	-0.92797	5.87E-02	4.7	4.9	5.2	4.2	4	4.2	6-pyruvoyltetrahydropterin synthase	PTPS
Down	PF3D7_1243700	-0.92552	1.12E-07	12	12	12	11	11	11	ubiquitin-conjugating enzyme E2, putative	N/A
Down	PF3D7_1017400	-0.92461	9.04E-07	8.3	8.2	8.4	7.4	7	7.7	phosphomannomutase	HAD5

Down	PF3D7_1206500	-0.92244	8.99E-03	5.9	5.6	5.6	4.9	4.9	5	Tat binding protein 1(TBP-1)-interacting protein, putative	N/A
Down	PF3D7_1104900	-0.92222	5.66E-10	12	12	12	11	11	12	calcium/calmodulin-dependent protein kinase, putative	N/A
Down	PF3D7_0408000	-0.92039	1.41E-08	10	10	11	9.6	9.5	9.8	conserved Plasmodium protein, unknown function	N/A
Down	PF3D7_1301500	-0.91795	8.08E-03	5.7	5.8	5.9	5.2	4.9	4.7	Plasmodium exported protein (PHISTa), unknown function	N/A
Down	PF3D7_1329700	-0.91477	1.57E-06	8.6	8.2	8.5	7.4	7.4	7.9	apicoplast calcium binding protein 1	ACBP1
Down	PF3D7_1444100	-0.91254	6.65E-08	9.4	9.3	9.6	8.4	8.3	8.8	conserved Plasmodium protein, unknown function	N/A
Down	PF3D7_1016200	-0.91104	2.90E-04	7	6.8	6.9	6.3	5.7	6.1	Rab3 GTPase-activating protein non-catalytic subunit, putative	N/A
Down	PF3D7_1345100	-0.91001	1.63E-04	7.9	7.7	8.2	7.5	7	6.6	thioredoxin 2	TRX2
Down	PF3D7_1001400	-0.90972	7.11E-04	7.5	6.9	7.4	6.1	6.1	6.9	exported lipase 1	XL1
Down	PF3D7_0310200	-0.90966	1.77E-09	12	12	12	11	11	11	phd finger protein, putative	N/A
Down	PF3D7_0927200	-0.90955	4.67E-07	8.6	8.5	9	7.9	7.8	7.6	zinc finger protein, putative	N/A
Down	PF3D7_1329500	-0.90894	4.62E-08	11	10	11	9.7	9.5	9.8	conserved protein, unknown function	N/A
Down	PF3D7_0423900	-0.90892	6.46E-05	7.6	7.6	8.3	7.2	6.8	7	probable protein, unknown function	N/A
Down	PF3D7_1317900	-0.90867	3.39E-02	5.3	5	5.6	4.7	4.3	4.5	nucleolar complex protein 4, putative	NOC4
Down	PF3D7_0308300	-0.90762	3.65E-08	10	10	11	9.4	9.4	9.8	PhIL1-interacting candidate PIC4	PIC4
Down	PF3D7_0523000	-0.90712	1.09E-07	11	11	11	11	10	10	multidrug resistance protein 1	MDR1
Down	PF3D7_1026600	-0.90533	1.78E-08	15	15	15	14	14	14	conserved Plasmodium protein, unknown function	N/A
Down	PF3D7_1121600	-0.90335	3.87E-11	12	12	13	12	11	11	exported protein 1	EXP1
Down	PF3D7_1001900	-0.90083	1.08E-04	7.3	7	7.7	6.4	6.5	6.6	Plasmodium exported protein (hyp16), unknown function	PfJ23
Down	PF3D7_0919200	-0.90001	1.66E-07	11	10	11	9.8	9.5	10	PPPDE peptidase, putative	N/A
Down	PF3D7_1023000	-0.89797	1.60E-08	9.6	9.3	9.6	8.6	8.4	8.8	conserved Plasmodium protein, unknown function	N/A
Down	PF3D7_1252800	-0.89787	9.68E-07	9.8	9.7	10	9.2	8.7	9.2	Plasmodium exported protein (PHISTb), unknown function	N/A
Down	PF3D7_0216700	-0.89694	1.74E-07	12	12	13	11	11	12	autophagy-related protein 11, putative	ATG11

Down	PF3D7_0524800	-0.89416	9.66E-07	9	8.6	8.8	7.9	7.6	8.2	ubiquitin fusion degradation protein 1, putative	UFD1
Down	PF3D7_1136600	-0.89381	3.18E-07	9	8.8	9.2	8.1	7.9	8.3	conserved Plasmodium protein, unknown function	N/A
Down	PF3D7_0830900	-0.89333	1.01E-06	8.2	8	8.4	7.3	7.4	7.3	Plasmodium exported protein, unknown function	N/A
Down	PF3D7_1208200	-0.89321	3.34E-02	5.4	5.3	5.9	5.2	4.1	4.9	cysteine repeat modular protein 3	CRMP3
Down	PF3D7_1125700	-0.89267	1.54E-09	12	12	12	11	11	11	kelch domain-containing protein, putative	N/A
Down	PF3D7_0420300	-0.89178	1.83E-09	14	14	15	13	13	14	AP2 domain transcription factor, putative	ApiAP2
Down	PF3D7_1016000	-0.89082	7.03E-07	10	10	10	9.2	9.1	9.6	conserved Plasmodium protein, unknown function	N/A
Down	PF3D7_1030300	-0.88879	2.71E-08	10	9.9	10	9.3	9.1	9.5	conserved Plasmodium protein, unknown function	N/A
Down	PF3D7_1112000	-0.88741	1.53E-06	7.9	8	8.3	7.3	7.1	7.3	conserved protein, unknown function	N/A
Down	PF3D7_0104200	-0.88681	5.31E-11	12	12	12	11	11	11	StAR-related lipid transfer protein	N/A
Down	PF3D7_1464600	-0.88592	1.41E-10	13	13	13	12	12	12	serine/threonine protein phosphatase UIS2, putative	UIS2
Down	PF3D7_1415200	-0.88591	3.05E-02	5.3	5.4	5.3	4.6	4.3	4.7	DNA-directed RNA polymerases I and III subunit RPAC2, putative	N/A
Down	PF3D7_1413700	-0.88553	1.51E-07	12	12	12	11	11	11	DET1 domain-containing protein, putative	N/A
Down	PF3D7_0301200	-0.88553	3.34E-04	6.9	6.6	7.1	6.1	5.9	6.1	serine/threonine protein kinase, FIKK family	FIKK3
Down	PF3D7_0828800	-0.88523	1.18E-07	12	12	12	11	11	12	GPI-anchored micronemal antigen	GAMA
Down	PF3D7_0729300	-0.88468	3.25E-03	6.5	6.2	6.9	6	5.5	5.5	60S ribosomal export protein NMD3, putative	NMD3
Down	PF3D7_0718300	-0.88466	3.00E-02	5.3	5.7	5.3	4.5	4.4	5.1	cysteine repeat modular protein 2	CRMP2
Down	PF3D7_0518900	-0.88461	3.20E-02	5.2	5.3	5.9	4.5	4.9	4.7	conserved protein, unknown function	N/A
Down	PF3D7_0931100	-0.88419	4.87E-05	8.4	8	8.9	7.7	7.6	7.6	nucleolar protein Nop52, putative	N/A
Down	PF3D7_1225500	-0.88037	2.55E-02	5.6	5.3	5.7	4.6	4.5	5.2	small subunit rRNA processing factor, putative	N/A
Down	PF3D7_0211100	-0.87979	9.71E-02	4.9	4.4	5	4.1	4	4.1	conserved Plasmodium protein, unknown function	N/A
Down	PF3D7_0931900	-0.87839	2.04E-05	7.9	7.8	8	7	6.7	7.3	adenylate kinase-like protein 2	AKLP2

Down	PF3D7_1411900	-0.87806	2.55E-03	6.9	6.2	6.7	6.1	5.6	5.6	p1/s1 nuclease, putative	N/A
Down	PF3D7_0822900	-0.87574	1.52E-08	14	14	14	13	13	13	PhIL1-interacting candidate PIC2	PIC2
Down	PF3D7_1326700	-0.87451	2.50E-05	7.8	7.7	8.2	7.2	7.2	6.8	conserved Apicomplexan protein, unknown function	N/A
Down	PF3D7_0515400	-0.87352	6.71E-08	11	11	12	11	10	11	conserved protein, unknown function	N/A
Down	PF3D7_1342600	-0.87291	2.08E-11	14	14	14	13	13	13	myosin A	MyoA
Down	PF3D7_1231400	-0.87144	1.35E-06	11	11	12	11	10	11	membrane protein ICM1	ICM1
Down	PF3D7_0807400	-0.8661	4.46E-04	6.7	6.7	6.9	5.8	6	6.1	coenzyme Q-binding protein COQ10 homolog, mitochondrial	COQ10
Down	PF3D7_1322100	-0.86576	2.69E-04	8.5	8	8.8	7.6	7.2	8	histone-lysine N-methyltransferase SET2	SET2
Down	PF3D7_0906500	-0.86444	4.74E-06	9	8.8	9.4	8.5	8.1	8.1	arginase	N/A
Down	PF3D7_1358400	-0.86412	1.40E-06	7.9	7.8	8	7	7.1	7.1	conserved Plasmodium protein, unknown function	N/A
Down	PF3D7_1471100	-0.86368	2.97E-10	11	11	11	10	9.9	10	exported protein 2	EXP2
Down	PF3D7_1231500	-0.86255	2.42E-04	6.9	7	7.3	6.3	6.1	6.3	mitosis protein dim1, putative	N/A
Down	PF3D7_0511500	-0.86161	1.37E-07	12	12	13	12	11	12	RNA pseudouridylate synthase, putative	N/A
Down	PF3D7_1122300	-0.86096	2.01E-03	6.6	6.5	6.9	5.9	5.5	6.2	conserved Plasmodium protein, unknown function	N/A
Down	PF3D7_0102700	-0.85802	2.03E-04	6.9	6.9	7.1	6.2	6.2	6	merozoite-associated tryptophan-rich antigen	MaTrA
Down	PF3D7_0618000	-0.8577	1.00E-10	13	13	13	12	12	12	conserved Plasmodium membrane protein, unknown function	N/A
Down	PF3D7_0621300	-0.85751	3.19E-02	5.9	5.2	6.1	5.2	5.4	4.4	mRNA-binding protein PUF3	PUF3
Down	PF3D7_0220700	-0.85629	7.49E-04	7.3	6.8	7.5	6.4	6.1	6.7	Plasmodium exported protein (hyp9), unknown function	N/A
Down	PF3D7_1335400	-0.85535	2.38E-07	13	13	13	12	12	13	reticulocyte binding protein 2 homologue a	RH2a
Down	PF3D7_0730300	-0.8517	1.60E-08	13	13	13	12	12	12	AP2 domain transcription factor AP2-L, putative	ApiAP2
Down	PF3D7_0622900	-0.85153	2.07E-08	11	11	11	10	10	10	AP2 domain transcription factor AP2Tel	AP2Tel
Down	PF3D7_1126700	-0.84992	4.66E-09	13	13	13	12	12	13	conserved Plasmodium protein, unknown function	ATG23
Down	PF3D7_1023800	-0.8494	8.87E-05	7.6	7.5	7.7	6.6	6.5	7.1	conserved Plasmodium protein, unknown function	N/A

Down	PF3D7_0612600	-0.8491	5.27E-09	9.4	9.3	9.4	8.4	8.4	8.7	cytoplasmic tRNA 2-thiolation protein 1, putative	NCS6
Down	PF3D7_0316100	-0.84862	5.48E-06	8.4	8.3	8.6	7.4	7.5	7.9	ribosomal protein L27, mitochondrial, putative	N/A
Down	PF3D7_1231300	-0.84827	3.38E-07	8.5	8.5	8.5	7.7	7.4	7.8	conserved Plasmodium protein, unknown function	N/A
Down	PF3D7_0809400	-0.84803	3.96E-08	8.8	8.8	8.9	7.8	8	8.1	conserved protein, unknown function	N/A
Down	PF3D7_0718600	-0.84657	9.47E-07	8.5	8.5	8.8	7.6	7.8	8	conserved Plasmodium protein, unknown function	N/A
Down	PF3D7_0927400	-0.84634	2.06E-02	5.7	5.5	6.4	5.2	5.4	4.9	conserved Plasmodium protein, unknown function	N/A
Down	PF3D7_1244100	-0.84621	1.77E-07	11	11	11	10	10	11	N-alpha-acetyltransferase 15, NatA auxiliary subunit, putative	N/A
Down	PF3D7_0323500	-0.84504	6.33E-08	9.4	9.3	9.7	8.6	8.5	8.8	survival motor neuron-like protein	SMN
Down	PF3D7_1407200	-0.84419	2.77E-06	8.5	8.4	8.8	7.9	7.6	7.7	conserved Plasmodium protein, unknown function	N/A
Down	PF3D7_0407900	-0.84242	1.24E-06	12	12	12	11	11	11	AAA family ATPase, putative	N/A
Down	PF3D7_1008800	-0.84197	8.79E-07	9	9	9.2	8.5	8.3	7.9	nucleolar protein 5, putative	NOP5
Down	PF3D7_1238900	-0.8415	6.44E-06	11	10	11	9.7	9.3	10	protein kinase 2	PK2
Down	PF3D7_1340300	-0.84025	3.37E-04	7.2	7.2	7.8	6.6	6.6	6.7	nucleolar complex protein 2, putative	N/A
Down	PF3D7_1344100	-0.83939	9.58E-08	9.7	9.6	9.8	8.8	8.7	9.2	TLD domain-containing protein, putative	N/A
Down	PF3D7_1342500	-0.83887	3.54E-03	6.3	6.2	6.5	5.4	5.5	5.7	sporozoite protein essential for cell traversal	SPECT1
Down	PF3D7_1204900	-0.83886	1.56E-05	9	9.1	9.6	8.3	8.2	8.7	conserved Plasmodium protein, unknown function	N/A
Down	PF3D7_0930400	-0.83814	7.59E-08	10	10	10	9.1	9	9.5	zinc finger protein, putative	N/A
Down	PF3D7_1221900	-0.8381	4.79E-06	8.5	8.1	8.6	7.7	7.4	7.7	conserved Plasmodium membrane protein, unknown function	N/A
Down	PF3D7_1237900	-0.83481	2.45E-08	12	12	13	12	11	12	conserved Plasmodium protein, unknown function	N/A
Down	PF3D7_0219800	-0.83455	2.35E-08	8.9	8.8	9	8	8.1	8.1	Plasmodium exported protein (PHISTc), unknown function	N/A
Down	PF3D7_0925900	-0.83448	1.13E-06	10	9.8	10	9.5	9.3	9.1	lipocalin	LCN
Down	PF3D7_0528300	-0.83356	2.10E-07	9.1	9.1	9.3	8.2	8.2	8.6	conserved protein, unknown function	N/A

Down	PF3D7_1026500	-0.83304	1.21E-04	7.1	7.3	7.4	6.6	6.4	6.4	conserved Plasmodium protein, unknown function	N/A
Down	PF3D7_0613600	-0.83006	8.44E-08	11	11	11	10	10	11	conserved Plasmodium protein, unknown function	N/A
Down	PF3D7_0419700	-0.82964	5.42E-10	10	9.9	10	9.2	9.1	9.3	apical merozoite protein	Pf34
Down	PF3D7_0504200	-0.82768	1.85E-05	8	8	8.4	7.2	7.1	7.5	ATP-dependent RNA helicase DDX27, putative	DDX27
Down	PF3D7_1454200	-0.8272	5.75E-02	5.6	5	5.7	5.1	4.8	4.1	conserved Plasmodium protein, unknown function	N/A
Down	PF3D7_1302300	-0.82285	2.51E-04	7.1	7	7.3	6.6	6.2	6.3	Plasmodium exported protein, unknown function	N/A
Down	PF3D7_1301600	-0.82256	1.02E-07	14	14	15	14	13	14	erythrocyte binding antigen-140	EBA140
Down	PF3D7_1361400	-0.82226	3.92E-07	8.6	8.4	8.4	7.8	7.6	7.6	actin-depolymerizing factor 2	ADF2
Down	PF3D7_1331500	-0.81946	1.72E-07	11	11	11	9.9	9.7	10	conserved Plasmodium protein, unknown function	N/A
Down	PF3D7_1127900	-0.81881	4.64E-06	9.2	9.1	9.6	8.4	8.3	8.8	conserved Plasmodium protein, unknown function	N/A
Down	PF3D7_0418600	-0.818	1.73E-07	13	13	13	12	12	12	regulator of chromosome condensation, putative	N/A
Down	PF3D7_1125800	-0.81689	1.05E-08	13	12	13	12	12	12	kelch domain-containing protein, putative	N/A
Down	PF3D7_1143800	-0.81679	1.50E-06	10	10	11	9.6	9.3	9.9	oocyst capsule protein Cap93, putative	CAP93
Down	PF3D7_1133500	-0.81456	9.48E-02	4.7	4.9	5.2	4.1	4.3	4.4	conserved protein, unknown function	N/A
Down	PF3D7_1356800	-0.81427	1.45E-07	14	14	14	13	13	13	serine/threonine protein kinase ARK3, putative	ARK3
Down	PF3D7_0628300	-0.81425	9.17E-11	13	13	13	12	12	12	choline/ethanolaminephosphotransferase, putative	CEPT
Down	PF3D7_1405700	-0.81379	2.11E-07	10	10	11	9.5	9.3	9.8	RING zinc finger protein, putative	N/A
Down	PF3D7_1307500	-0.81361	9.88E-06	8.5	8.6	9	7.9	7.7	8.1	conserved Plasmodium protein, unknown function	N/A
Down	PF3D7_0102500	-0.81219	1.03E-07	14	14	15	14	13	14	erythrocyte binding antigen-181	EBA181
Down	PF3D7_1035500	-0.81165	5.63E-08	14	14	14	13	13	13	merozoite surface protein 6	MSP6
Down	PF3D7_1327600	-0.81116	4.07E-05	8.5	8.3	8.5	7.6	7.2	8	nicotinamide/nicotinic acid mononucleotide adenylyltransferase	NMNAT

Down	PF3D7_0214800	-0.81088	1.02E-07	10	10	10	9.5	9.2	9.7	conserved Plasmodium membrane protein, unknown function	N/A
Down	PF3D7_0902400	-0.80946	6.92E-02	5.2	4.9	5.4	4.9	4.2	4.4	serine/threonine protein kinase, FIKK family	FIKK9.5
Down	PF3D7_1476300	-0.80619	1.53E-10	12	12	11	11	11	11	Plasmodium exported protein (PHISTb), unknown function	N/A
Down	PF3D7_0210600	-0.80589	8.79E-07	12	12	12	11	11	11	protein CERLI1	CERLI1
Down	PF3D7_1341500	-0.80352	1.24E-08	9.3	9.1	9.3	8.4	8.4	8.5	inner membrane complex suture component, putative	ISC1
Down	PF3D7_1445500	-0.80251	3.44E-05	7.8	7.7	7.9	7	6.8	7.2	conserved Plasmodium protein, unknown function	N/A
Down	PF3D7_0630700	-0.80169	1.77E-03	6.6	6.9	7.2	6.3	5.9	6.2	bifunctional methylenetetrahydrofolate dehydrogenase/cyclohydrolase, putative	N/A
Down	PF3D7_0318500	-0.8003	2.39E-08	13	13	13	12	12	12	conserved Plasmodium protein, unknown function	N/A
Down	PF3D7_1438800	-0.79946	7.71E-03	6	6.4	6.5	5.7	5.3	5.7	OST-HTH associated domain protein, putative	N/A
Down	PF3D7_0813000	-0.79854	3.66E-06	9.8	9.6	10	9.3	9	8.8	protein KIC7	KIC7
Down	PF3D7_1313800	-0.7983	1.28E-07	11	11	11	10	9.9	10	conserved Plasmodium membrane protein, unknown function	N/A
Down	PF3D7_0723200	-0.79804	1.21E-02	6	5.9	6.3	5.3	5.4	5.2	conserved Plasmodium protein, unknown function	N/A
Down	PF3D7_0214600	-0.79485	1.23E-06	13	13	13	12	12	12	serine/threonine protein kinase STK2, putative	STK2
Down	PF3D7_0316900	-0.79477	3.25E-10	10	10	10	9.5	9.4	9.5	E3 ubiquitin-protein ligase, putative	N/A
Down	PF3D7_0503600	-0.79244	6.92E-07	12	12	12	11	11	11	myosin B	MyoB
Down	PF3D7_1321900	-0.78932	8.13E-08	11	11	12	11	10	11	conserved protein, unknown function	N/A
Down	PF3D7_0911100	-0.78919	2.91E-08	13	13	13	12	12	13	START domain-containing protein, putative	N/A
Down	PF3D7_0204200	-0.78876	1.96E-06	10	10	11	9.6	9.6	10	translocation protein SEC66, putative	SEC66
Down	PF3D7_0210700	-0.78783	3.45E-05	8.6	8.6	9	7.9	7.7	8.2	syntaxin, Qa-SNARE family	SYN17
Down	PF3D7_0220500	-0.78698	1.01E-06	9.6	9.6	9.8	8.8	8.8	9.2	Plasmodium exported protein (hyp2), unknown function	N/A
Down	PF3D7_0920700	-0.78631	4.53E-07	11	11	11	10	10	11	CRAL/TRIO domain-containing protein, putative	N/A

Down	PF3D7_0302800	-0.78564	5.85E-04	7.1	7.2	7.3	6.3	6.2	6.7	RNA-binding protein, putative	N/A
Down	PF3D7_0203000	-0.7856	8.56E-07	12	12	12	11	11	12	repetitive organellar protein, putative	ROPE
Down	PF3D7_0724900	-0.78555	2.39E-06	10	10	11	9.4	9.5	9.9	kinesin-20, putative	N/A
Down	PF3D7_1460600	-0.785	1.70E-06	8.8	8.7	9	8	8	8.3	inner membrane complex sub-compartment protein 3	ISP3
Down	PF3D7_0934600	-0.78461	1.44E-04	8.4	8.3	8.9	7.8	7.6	8	conserved Plasmodium protein, unknown function	N/A
Down	PF3D7_1136400	-0.78432	4.15E-05	9.1	8.7	9.1	8.4	7.8	8.4	signal recognition particle subunit SRP72, putative	SRP72
Down	PF3D7_1347700	-0.78404	1.69E-07	11	10	10	9.9	9.6	9.5	ethanolamine-phosphate cytidylyltransferase	ECT
Down	PF3D7_1316200	-0.78362	2.54E-05	9.2	9.1	9.4	8.4	8.1	8.8	ADP-ribosylation factor, putative	N/A
Down	PF3D7_1035900	-0.78248	1.47E-07	13	13	13	12	12	13	merozoites-associated armadillo repeats protein	MAAP
Down	PF3D7_0913800	-0.78165	2.15E-05	10	10	11	9.4	9.4	9.8	conserved Plasmodium protein, unknown function	N/A
Down	PF3D7_1224200	-0.78154	1.72E-06	9.1	9	9.3	8.4	8.2	8.6	BRO1 domain-containing protein, putative	N/A
Down	PF3D7_1245800	-0.7787	7.39E-07	9.6	9.5	9.8	8.9	8.6	9.1	epsin-like protein, putative	EpsL
Down	PF3D7_0803500	-0.77725	2.77E-06	11	11	12	11	10	11	AAA family ATPase, putative	N/A
Down	PF3D7_1326900	-0.77719	4.27E-04	7.4	7.3	7.6	6.5	6.5	7	conserved Plasmodium membrane protein, unknown function	N/A
Down	PF3D7_1351700	-0.77688	3.19E-09	13	13	14	13	13	13	inner membrane complex protein 1f, putative	IMC1f
Down	PF3D7_1231200	-0.77331	7.46E-07	10	9.9	10	9.2	9.1	9.5	conserved Plasmodium protein, unknown function	N/A
Down	PF3D7_1473400	-0.77255	3.38E-07	11	11	11	9.9	9.8	10	conserved protein, unknown function	N/A
Down	PF3D7_0614700	-0.77224	2.07E-07	9.9	9.8	9.8	9	8.9	9.3	F-box protein FBXO6, putative	N/A
Down	PF3D7_1367400	-0.77079	3.51E-03	6.7	6.5	6.5	6	5.7	5.8	conserved Plasmodium protein, unknown function	N/A
Down	PF3D7_0220200	-0.77066	2.34E-06	9.9	9.6	10	9.1	8.9	9.3	Plasmodium exported protein, unknown function	N/A
Down	PF3D7_0323400	-0.77064	2.05E-07	13	13	13	12	12	12	Rh5 interacting protein	RIPR
Down	PF3D7_0507400	-0.77012	4.53E-07	9.3	9	9.3	8.5	8.3	8.5	subtilisin propeptide-like protein	SUB1-ProM

Down	PF3D7_0818100	-0.77008	1.55E-06	12	12	12	11	11	11	zinc finger protein, putative	N/A
Down	PF3D7_0613300	-0.76953	1.42E-08	13	12	12	12	12	12	rhoptry protein ROP14	ROP14
Down	PF3D7_1035800	-0.76725	1.73E-03	7.3	6.8	7.2	6.2	6.2	6.6	probable protein, unknown function	M712
Down	PF3D7_1366100	-0.76637	4.30E-05	8.2	8.1	8.5	7.2	7.6	7.6	DIP13 homolog, putative	DIP13
Down	PF3D7_0711100	-0.76395	1.54E-02	6.3	5.8	6.3	5.3	5.5	5.5	conserved protein, unknown function	N/A
Down	PF3D7_1210200	-0.76257	8.62E-02	5.3	5.2	5.3	4.2	4.3	5.2	zinc finger protein, putative	N/A
Down	PF3D7_1020700	-0.76162	1.13E-02	6.8	6.3	6.8	6.4	5.9	5.5	N-acetyltransferase, GNAT family, putative	N/A
Down	PF3D7_1310700	-0.76106	3.27E-07	13	13	13	12	12	12	PhIL1-interacting candidate PIC5	PIC5
Down	PF3D7_0620500	-0.75956	8.86E-06	8.9	8.8	8.8	8.2	7.7	8.3	cleavage stimulation factor subunit 1, putative	N/A
Down	PF3D7_0613900	-0.75933	9.61E-07	15	15	15	14	14	14	myosin E, putative	MyoE
Down	PF3D7_1414700	-0.75827	2.99E-02	6.1	5.9	7.1	5.8	5.7	5.7	ubiquitin carboxyl-terminal hydrolase, putative	N/A
Down	PF3D7_0206000	-0.75788	3.67E-09	10	10	10	9.6	9.5	9.7	DNA repair protein RAD2, putative	RAD2
Down	PF3D7_1146100	-0.7574	5.73E-02	5.3	5.3	6	5	4.7	5	PH-like domain-containing protein, putative	N/A
Down	PF3D7_1401900	-0.7573	1.24E-04	7.8	7.8	8.2	7.1	7.3	7.3	conserved protein, unknown function	N/A
Down	PF3D7_1235000	-0.75658	3.14E-02	5.7	6.1	5.5	5.1	5	5.2	PIH1 domain-containing protein, putative	PIH1
Down	PF3D7_1031000	-0.75645	4.01E-02	5.5	5.7	5.6	4.9	5.1	4.7	ookinete surface protein P25	P25
Down	PF3D7_1246400	-0.75614	1.24E-09	12	12	12	11	11	12	myosin A-tail interacting protein	MTIP
Down	PF3D7_0717300	-0.75466	1.77E-06	9.2	9	9.3	8.2	8.5	8.5	transcription initiation factor IIE subunit alpha, putative	N/A
Down	PF3D7_0620400	-0.75461	2.01E-08	9.8	9.8	9.9	9.1	8.9	9.2	merozoite surface protein 10	MSP10
Down	PF3D7_1417400	-0.75447	1.28E-07	12	12	12	11	11	11	rap guanine nucleotide exchange factor, putative, pseudogene	EPAC
Down	PF3D7_0722200	-0.75426	7.33E-10	14	14	14	13	13	13	rhoptry-associated leucine zipper-like protein 1	RALP1
Down	PF3D7_1005700	-0.75228	2.27E-03	6.9	6.7	7	6.3	5.9	6.3	peptidase, putative	N/A
Down	PF3D7_0706300	-0.75195	4.76E-05	9	8.8	8.9	8.1	7.8	8.5	conserved Plasmodium protein, unknown function	N/A
Down	PF3D7_1318700	-0.74984	3.53E-07	11	11	11	9.9	9.9	10	protein AAP4	AAP4

Down	PF3D7_1476500	-0.74747	1.75E-02	6.1	5.9	6.4	5.6	5.4	5.3	probable protein, unknown function	N/A
Down	PF3D7_1137200	-0.74649	2.73E-07	9.5	9.3	9.4	8.8	8.5	8.8	apical exonemal protein	AEP
Down	PF3D7_1472300	-0.74633	3.37E-06	9.3	9.2	9.4	8.7	8.3	8.7	conserved protein, unknown function	N/A
Down	PF3D7_1209400	-0.74551	1.96E-06	11	11	11	10	10	11	cytosolic iron-sulfur protein assembly protein 1, putative	CIA1
Down	PF3D7_0206100	-0.74259	1.46E-03	7.3	6.8	7.2	6.3	6.3	6.5	cysteine desulfuration protein SufE	SufE
Down	PF3D7_1143100	-0.74246	8.27E-06	13	13	13	12	12	13	AP2 domain transcription factor AP2-O	AP2-O
Down	PF3D7_0818500	-0.74028	2.05E-08	11	11	11	10	10	10	zinc finger protein, putative	N/A
Down	PF3D7_0418700	-0.73898	1.34E-04	10	10	10	9.4	9.1	9.9	RNA-binding protein NOB1, putative	N/A
Down	PF3D7_0614600	-0.73852	3.93E-03	6.8	6.7	6.5	6	5.8	6.2	conserved Plasmodium protein, unknown function	N/A
Down	PF3D7_1129100	-0.73833	1.84E-07	12	12	12	11	11	11	parasitophorous vacuolar protein 1	PV1
Down	PF3D7_1408500	-0.73811	3.06E-02	5.8	5.9	6	5.2	5.5	4.9	conserved Plasmodium protein, unknown function	N/A
Down	PF3D7_1425400	-0.73797	7.35E-07	11	11	11	9.7	9.8	10	DEAD/DEAH box helicase, putative	N/A
Down	PF3D7_1229800	-0.73672	2.04E-07	13	13	13	12	12	13	myosin J, putative	MyoJ
Down	PF3D7_0617400	-0.73608	8.44E-05	7.8	7.8	7.7	7.1	6.9	7.1	erythrocyte membrane protein 1, PfEMP1	VAR
Down	PF3D7_0802700	-0.7353	2.97E-02	6	5.7	5.8	5	5.4	5.2	conserved Plasmodium protein, unknown function	N/A
Down	PF3D7_1371600	-0.73521	9.66E-07	12	12	12	11	11	12	erythrocyte binding like protein 1, pseudogene	EBL1
Down	PF3D7_0220100	-0.73476	3.44E-03	7.8	7.2	7.9	7.1	6.5	7.1	DnaJ protein, putative	N/A
Down	PF3D7_1125900	-0.73463	4.46E-03	7.5	7.7	8.2	6.8	6.9	7.6	XTBD domain-containing protein, putative	N/A
Down	PF3D7_0725400	-0.73396	1.34E-05	12	12	13	12	11	12	conserved Plasmodium protein, unknown function	N/A
Down	PF3D7_1335300	-0.73384	1.28E-05	12	12	13	12	11	12	reticulocyte binding protein 2 homologue b	RH2b
Down	PF3D7_0408300	-0.73262	1.18E-04	7.8	8.2	7.9	7.2	7.2	7.3	zinc finger Ran-binding domain-containing protein 2, putative	ZRANB2
Down	PF3D7_1331000	-0.73178	9.57E-06	9.2	9.1	9.3	8.3	8.4	8.7	protein kinase, putative	N/A
Down	PF3D7_1353100	-0.73127	1.38E-02	7.1	6.2	7.2	6.2	6.2	6.2	Plasmodium exported protein, unknown function	N/A

Down	PF3D7_1359000	-0.73073	2.18E-03	7.3	7.1	7.8	6.8	6.5	6.8	conserved Plasmodium protein, unknown function	N/A
Down	PF3D7_1018800	-0.73043	9.47E-02	5	5.2	5.3	4.7	4.5	4.5	conserved protein, unknown function	N/A
Down	PF3D7_1126600	-0.72974	3.55E-03	7.4	7.1	7.7	6.5	6.5	7.1	steryl ester hydrolase, putative	N/A
Down	PF3D7_0207100	-0.72889	1.01E-06	13	12	13	12	12	12	conserved Plasmodium protein, unknown function	N/A
Down	PF3D7_0423800	-0.72875	4.69E-06	10	10	10	9.4	9.3	9.7	cysteine-rich protective antigen	CyRPA
Down	PF3D7_0311600	-0.72635	1.61E-02	6.2	6	6.4	5.7	5.4	5.5	dolichyl-diphosphooligosaccharide--protein glycosyltransferase subunit OST1, putative	OST1
Down	PF3D7_1405800	-0.72541	5.62E-05	8.5	8.3	8.6	7.9	7.5	7.8	ribosome biogenesis protein BOP1, putative	BOP1
Down	PF3D7_0919300	-0.72372	3.21E-04	8.7	8.9	8.6	7.8	7.7	8.4	thioredoxin-like protein 1, putative	TrxL1
Down	PF3D7_0304100	-0.72274	3.07E-07	11	11	11	10	10	11	inner membrane complex protein 1e, putative	IMC1e
Down	PF3D7_1468400	-0.72233	4.69E-06	11	10	11	9.8	9.7	10	zinc finger protein, putative	D13
Down	PF3D7_1017500	-0.72213	2.35E-06	12	11	12	11	11	11	myosin essential light chain ELC	ELC
Down	PF3D7_1030200	-0.72081	8.70E-08	12	12	12	11	11	11	claudin-like apicomplexan microneme protein, putative	CLAMP
Down	PF3D7_0507500	-0.71896	7.41E-06	13	13	13	12	12	12	subtilisin-like protease 1	SUB1
Down	PF3D7_1352100	-0.717	2.55E-08	10	9.9	9.9	9.2	9.1	9.3	ABC transporter B family member 6, putative	ABCB6
Down	PF3D7_1253300	-0.71614	9.06E-02	5.7	5	6.3	5.1	4.7	5.3	Plasmodium exported protein (PHISTa), unknown function, pseudogene	N/A
Down	PF3D7_0916700	-0.71593	2.98E-06	9.8	9.7	10	9.3	9.1	9.1	RNA-binding protein musashi, putative	HoMu
Down	PF3D7_0813300	-0.71521	6.10E-05	8.9	8.7	9.2	8.3	8.1	8.4	NPL domain-containing protein, putative	N/A
Down	PF3D7_1366000	-0.71255	9.51E-02	5.1	5.2	5.5	4.6	4.7	4.6	conserved Plasmodium protein, unknown function	N/A
Down	PF3D7_1028700	-0.71248	2.66E-06	13	13	13	12	12	13	merozoite TRAP-like protein	MTRAP
Down	PF3D7_1007300	-0.7122	3.89E-06	10	10	10	9.5	9.4	9.9	RING zinc finger protein, putative	N/A
Down	PF3D7_1469200	-0.71141	3.93E-07	10	10	10	9.4	9.4	9.7	shewanella-like protein phosphatase 1, putative	SHLP1
Down	PF3D7_1232400	-0.71081	2.45E-03	7.5	7.4	7.9	7.3	6.6	6.8	CWC16 domain-containing protein, putative	N/A

Down	PF3D7_1348600	-0.71053	1.76E-07	9.7	9.6	9.6	8.9	8.8	9.1	conserved Plasmodium protein, unknown function	N/A
Down	PF3D7_0914000	-0.71043	1.35E-03	7.5	7.2	7.6	6.6	6.6	7	pseudouridylate synthase, putative	N/A
Down	PF3D7_0731100	-0.70968	4.07E-05	10	9.6	10	9.3	9.1	9.4	EMP1-trafficking protein	PTP2
Down	PF3D7_1450000	-0.70936	5.31E-04	8.7	8.5	9.1	8	7.8	8.4	serine/threonine protein kinase, putative	N/A
Down	PF3D7_0722000	-0.70908	8.87E-05	8.2	8	8.4	7.7	7.4	7.6	conserved Plasmodium protein, unknown function	N/A
Down	PF3D7_1420300	-0.70829	9.83E-03	6.3	6.4	6.5	5.7	5.7	5.8	DNL-type zinc finger protein	HEP1
Down	PF3D7_1458000	-0.70827	5.09E-05	9.1	8.9	9.3	8.6	8.4	8.1	cysteine proteinase falcipain 1	FP1
Down	PF3D7_1218000	-0.708	1.54E-05	9.2	9	9.4	8.3	8.4	8.7	thrombospondin-related apical membrane protein	TRAMP
Down	PF3D7_1315100	-0.70783	9.06E-02	5.4	5.2	5.7	4.6	4.5	5.2	serine/threonine protein kinase PK9	PK9
Down	PF3D7_1118600	-0.7075	1.12E-06	9.2	9.2	9.3	8.7	8.5	8.4	histone acetyltransferase MYST	MYST
Down	PF3D7_0808200	-0.70734	5.10E-07	12	12	12	11	11	12	plasmepsin X	PMX
Down	PF3D7_1205500	-0.70683	1.95E-06	12	12	12	11	11	11	zinc finger protein, putative	N/A
Down	PF3D7_1036000	-0.70585	2.97E-05	12	11	12	11	11	11	merozoite surface protein 11	MSP11
Down	PF3D7_0936800	-0.70559	3.14E-02	7.3	6.5	6.1	6.2	5.9	5.9	Plasmodium exported protein (PHISTc), unknown function	N/A
Down	PF3D7_1251200	-0.70515	2.34E-06	14	14	14	13	13	14	coronin	N/A
Down	PF3D7_0109000	-0.70447	1.44E-05	12	12	12	11	11	12	photosensitized INA-labeled protein PHIL1	PHIL1
Down	PF3D7_0903600	-0.70345	4.15E-05	12	12	13	12	11	12	conserved protein, unknown function	N/A
Down	PF3D7_0828700	-0.70342	1.46E-04	8.4	8.3	8.6	7.6	7.5	8	conserved protein, unknown function	N/A
Down	PF3D7_0105500	-0.70333	4.94E-02	5.9	6	5.6	5.4	4.7	5.5	conserved protein, unknown function	N/A
Down	PF3D7_0924400	-0.70245	3.90E-07	12	12	12	11	11	12	conserved Plasmodium protein, unknown function	N/A
Down	PF3D7_1327500	-0.70223	2.04E-05	8.2	8.3	8.2	7.6	7.5	7.5	conserved protein, unknown function	N/A
Down	PF3D7_1113200	-0.69893	1.82E-02	6.5	6.1	6.4	5.9	5.5	5.6	conserved Plasmodium protein, unknown function	N/A
Down	PF3D7_0914100	-0.6971	1.04E-05	12	12	12	11	11	11	conserved Plasmodium protein, unknown function	N/A
Down	PF3D7_0717400	-0.69454	1.95E-05	9	8.8	9.2	8.2	8.3	8.5	queuine tRNA-ribosyltransferase, putative	N/A

Down	PF3D7_1321300	-0.69397	5.31E-05	9.6	9.5	9.9	8.9	8.8	9.2	conserved Plasmodium membrane protein, unknown function	N/A
Down	PF3D7_1425300	-0.69287	3.50E-06	9.8	9.6	10	9	9.1	9.3	conserved Plasmodium protein, unknown function	N/A
Down	PF3D7_1013200	-0.69184	4.77E-07	11	11	11	11	10	11	conserved Plasmodium protein, unknown function	N/A
Down	PF3D7_1230100	-0.69176	4.18E-04	7.6	7.6	7.5	6.7	6.9	7	thioredoxin-like associated protein 1, putative	TLAP1
Down	PF3D7_1003600	-0.69161	5.80E-06	14	14	14	13	13	13	inner membrane complex protein 1c, putative	IMC1c
Down	PF3D7_1216200	-0.69083	3.38E-06	9.3	9.1	9.3	8.5	8.4	8.7	glycerol-3-phosphate dehydrogenase [NAD(+)], putative	N/A
Down	PF3D7_1013000	-0.69071	9.73E-05	8	7.9	8	7.3	7.3	7.2	zinc finger protein, putative	N/A
Down	PF3D7_1415100	-0.69067	9.71E-04	8.5	8.2	8.6	7.6	7.4	8.2	conserved protein, unknown function	N/A
Down	PF3D7_1136900	-0.69003	5.53E-05	12	12	12	11	11	12	subtilisin-like protease 2	SUB2
Down	PF3D7_1035400	-0.68955	1.67E-06	14	14	14	13	13	13	merozoite surface protein 3	MSP3
Down	PF3D7_1450300	-0.68926	1.63E-02	6.5	6.4	6.3	5.8	6	5.4	NADPH--cytochrome P450 reductase, putative	CPR
Down	PF3D7_1028900	-0.68838	2.60E-05	9.4	9.3	9.5	8.5	8.6	9	inner membrane complex protein 1m, putative	IMC1m
Down	PF3D7_0102600	-0.68756	2.81E-02	6.6	5.9	6.5	5.6	5.5	6	serine/threonine protein kinase, FIKK family	FIKK1
Down	PF3D7_0505200	-0.68691	4.53E-05	8.9	8.8	9	8.2	7.9	8.4	actin-like protein, putative	ALP2b
Down	PF3D7_1332400	-0.68681	2.50E-04	8.2	8	8.3	7.7	7.3	7.4	nucleotidyltransferase, putative	N/A
Down	PF3D7_1217500	-0.68674	2.45E-04	8.5	8.2	8.8	7.9	7.9	7.7	conserved Plasmodium protein, unknown function	N/A
Down	PF3D7_0522100	-0.68625	1.63E-05	9.7	9.6	9.7	8.8	8.8	9.2	conserved Plasmodium protein, unknown function	N/A
Down	PF3D7_1419400	-0.68528	4.77E-05	9	8.9	9.3	8.5	8.5	8.2	conserved Plasmodium membrane protein, unknown function	N/A
Down	PF3D7_0410600	-0.6849	2.43E-05	9.3	9.2	9.2	8.5	8.3	8.8	armadillo-type repeat protein ATRP	ATRP
Down	PF3D7_1230900	-0.68347	2.12E-05	8.8	8.6	9	8.1	8.1	8.2	serine/threonine protein kinase RIO1, putative	RIO1
Down	PF3D7_0305300	-0.68321	6.61E-05	9.4	9.1	9.6	8.9	8.7	8.6	transporter, putative	N/A
Down	PF3D7_0407700	-0.68109	4.99E-07	12	12	12	11	11	11	conserved Plasmodium protein, unknown function	N/A

Down	PF3D7_1223000	-0.68035	2.57E-04	7.9	8.1	8.3	7.4	7.3	7.5	conserved Plasmodium protein, unknown function	N/A
Down	PF3D7_0517200	-0.67986	2.09E-02	6.4	6.2	6.8	5.7	5.7	6	conserved Plasmodium protein, unknown function	N/A
Down	PF3D7_1208100	-0.6794	1.13E-03	8.1	7.9	8.3	7.7	7.2	7.3	conserved Plasmodium protein, unknown function	N/A
Down	PF3D7_0909700	-0.67814	2.54E-06	10	10	10	9.6	9.4	9.8	FHA domain protein, putative	N/A
Down	PF3D7_0908100	-0.6764	2.96E-02	6.1	6.2	6.3	5.8	5.7	5.2	apicoplast integral membrane protein, putative	N/A
Down	PF3D7_0902100	-0.6747	5.83E-06	9.5	9.4	9.5	8.7	8.7	9	serine/threonine protein kinase, FIKK family	FIKK9.2
Down	PF3D7_0627100	-0.67444	6.06E-08	12	12	12	11	11	11	ankyrin-repeat protein, putative	N/A
Down	PF3D7_0913900	-0.67405	2.65E-02	6.3	6.2	6.5	6	5.3	5.7	arginine--tRNA ligase, putative	RRSapi
Down	PF3D7_0926300	-0.67395	1.57E-03	7.6	7.3	7.8	6.8	6.8	7.1	protein kinase, putative	N/A
Down	PF3D7_1202700	-0.67353	2.69E-02	6.2	6	6.2	5.5	5.5	5.5	AATF domain-containing protein, putative	N/A
Down	PF3D7_0314600	-0.67251	1.45E-03	7.3	7.4	7.6	6.9	6.6	6.8	conserved protein, unknown function	N/A
Down	PF3D7_1455300	-0.67204	6.65E-06	11	11	12	11	11	11	conserved protein, unknown function	N/A
Down	PF3D7_0520500	-0.6712	6.48E-04	7.6	7.8	7.5	7	6.9	7.2	phosphomethylpyrimidine kinase, putative	N/A
Down	PF3D7_0625000	-0.67095	3.59E-05	9.3	9.2	9.4	8.6	8.4	8.9	sphingomyelin synthase 1, putative	SMS1
Down	PF3D7_0621400	-0.66728	6.97E-04	8.1	8.2	8	7.2	7.3	7.8	Pf77 protein	ALV7
Down	PF3D7_0924000	-0.6657	8.15E-06	12	11	12	11	11	11	patatin-like phospholipase, putative	N/A
Down	PF3D7_0309800	-0.6654	5.12E-02	6	5.8	6.2	5.7	5.3	5	YTH domain-containing protein 2	YTH2
Down	PF3D7_1352200	-0.66496	2.34E-05	8.6	8.5	8.7	7.9	7.9	8	U3 small nucleolar RNA-associated protein 15, putative	UTP15
Down	PF3D7_0808300	-0.66472	5.80E-06	10	10	10	9.4	9.4	9.7	ubiquitin regulatory protein, putative	N/A
Down	PF3D7_1444800	-0.66367	1.14E-06	13	13	13	13	13	12	fructose-bisphosphate aldolase	FBPA
Down	PF3D7_1122200	-0.6635	1.09E-04	8.2	8.1	8.2	7.5	7.3	7.6	JmjC domain-containing protein 3	JmjC3
Down	PF3D7_1335100	-0.66224	8.63E-07	14	14	14	13	13	13	merozoite surface protein 7	MSP7
Down	PF3D7_1145000	-0.66161	1.33E-03	7.9	7.6	7.5	7	6.8	7.2	conserved Plasmodium protein, unknown function	N/A
Down	PF3D7_1018600	-0.66077	1.18E-06	9.7	9.7	9.9	9.1	9.1	9.1	tRNA wybutosine-synthesizing protein, putative	N/A

Down	PF3D7_0629300	-0.66015	2.14E-05	11	11	11	9.9	9.9	10	phospholipase, putative	PL
Down	PF3D7_1206000	-0.66013	1.25E-06	10	11	10	9.7	9.7	10	shewanella-like protein phosphatase 2	SHLP2
Down	PF3D7_1237200	-0.65961	2.00E-04	8.2	8.1	8.3	7.6	7.3	7.7	conserved Plasmodium protein, unknown function	N/A
Down	PF3D7_1405100	-0.65954	2.46E-05	8.9	8.9	9.1	8.3	8.2	8.5	GTPase-activating protein, putative	N/A
Down	PF3D7_1209600	-0.65941	9.29E-03	7	7.1	6.8	6.3	6	6.7	porphobilinogen deaminase	PBGD
Down	PF3D7_1030900	-0.65892	1.24E-02	6.6	6.7	6.7	6.1	5.7	6.3	ookinete surface protein P28	P28
Down	PF3D7_0404000	-0.65817	4.96E-05	9.2	9	9	8.3	8.3	8.7	conserved Plasmodium protein, unknown function	N/A
Down	PF3D7_0914200	-0.65804	1.62E-04	8.2	8.3	8.4	7.9	7.7	7.5	phospholipid or glycerol acyltransferase, putative	N/A
Down	PF3D7_0503400	-0.65801	1.20E-07	12	12	12	11	11	11	actin-depolymerizing factor 1	ADF1
Down	PF3D7_1313500	-0.65793	4.98E-05	9	9	9.4	8.5	8.4	8.6	conserved Plasmodium membrane protein, unknown function	N/A
Down	PF3D7_0203100	-0.65772	1.18E-04	11	11	12	11	11	11	protein kinase, putative	N/A
Down	PF3D7_1223400	-0.65738	1.71E-07	12	12	12	12	12	12	phospholipid-transporting ATPase, putative	N/A
Down	PF3D7_0801000	-0.657	5.24E-04	9.7	9.2	9.9	8.9	8.8	9.2	Plasmodium exported protein (PHISTc)	N/A
Down	PF3D7_1003300	-0.65635	8.69E-05	8.5	8.5	8.7	7.8	7.9	8.1	N-terminal acetyltransferase A complex catalytic subunit ARD1, putative	ARD1
Down	PF3D7_1435600	-0.65504	1.64E-06	13	13	13	12	12	12	conserved Plasmodium protein, unknown function	N/A
Down	PF3D7_1434400	-0.65275	3.78E-05	10	10	10	9.3	9.3	9.7	conserved protein, unknown function	N/A
Down	PF3D7_1018300	-0.64959	7.57E-06	10	10	10	9.5	9.3	9.5	peptidase, putative	N/A
Down	PF3D7_1013800	-0.64891	3.49E-05	11	11	11	10	10	11	conserved Plasmodium protein, unknown function	N/A
Down	PF3D7_1206300	-0.64872	9.68E-07	12	12	12	11	11	12	conserved Plasmodium protein, unknown function	N/A
Down	PF3D7_1432300	-0.64871	1.25E-03	7.5	7.7	7.7	7.2	6.8	7	conserved Plasmodium protein, unknown function	N/A
Down	PF3D7_1108100	-0.6484	8.22E-07	11	11	12	11	11	11	conserved Plasmodium protein, unknown function	N/A
Down	PF3D7_0409800	-0.64765	1.19E-06	11	11	11	10	10	10	zinc finger protein, putative	N/A

Down	PF3D7_1472400	-0.64723	1.02E-03	7.8	7.9	8.2	7.3	7.3	7.6	M1-family alanyl aminopeptidase, putative	N/A
Down	PF3D7_0400200	-0.64582	1.58E-06	11	11	11	10	10	10	erythrocyte membrane protein 1 (PfEMP1), exon 2, pseudogene	N/A
Down	PF3D7_1140600	-0.64535	1.50E-05	9.5	9.4	9.6	8.8	8.7	9.1	conserved Plasmodium protein, unknown function	N/A
Down	PF3D7_1411200	-0.64414	2.81E-06	12	11	12	11	11	11	rhomboid protease ROM8	ROM8
Down	PF3D7_0804300	-0.64381	1.19E-02	6.8	6.8	7	6.5	6.3	5.9	zinc finger protein, putative	N/A
Down	PF3D7_1035700	-0.64332	1.89E-06	13	13	13	13	13	13	duffy binding-like merozoite surface protein	DBLMSP
Down	PF3D7_0820300	-0.64274	1.83E-06	10	10	10	9.6	9.5	9.8	conserved Plasmodium protein, unknown function	N/A
Down	PF3D7_0414900	-0.64254	1.56E-05	12	12	12	11	11	12	armadillo-domain containing rhoptry protein	ARO
Down	PF3D7_0414700	-0.6422	5.58E-06	9.9	9.9	9.8	9.1	9.1	9.4	GTP-binding protein, putative	N/A
Down	PF3D7_1331200	-0.64096	1.15E-03	7.6	7.4	7.7	7	6.9	7	conserved Plasmodium protein, unknown function	N/A
Down	PF3D7_1233700	-0.64064	5.06E-03	7.1	7.2	6.9	6.6	6.3	6.5	homocysteine S-methyltransferase, putative	N/A
Down	PF3D7_0509800	-0.63999	6.50E-07	12	12	12	11	11	11	phosphatidylinositol 4-kinase beta	PI4KB
Down	PF3D7_0305500	-0.6399	3.50E-06	13	13	13	12	12	12	protein dopey homolog, putative	N/A
Down	PF3D7_1320000	-0.63888	1.09E-05	10	10	10	9.6	9.3	9.7	golgi protein 1	GP1
Down	PF3D7_0729700	-0.6382	2.76E-03	7.5	7.2	7.2	6.7	6.7	6.7	zinc finger protein, putative	N/A
Down	PF3D7_0930100	-0.63805	7.88E-05	9.3	9.1	9.4	8.5	8.5	8.8	heptatricopeptide repeat-containing protein, putative	N/A
Down	PF3D7_1237100	-0.63771	1.96E-05	9.7	9.7	9.5	8.9	8.9	9.2	conserved Plasmodium protein, unknown function	N/A
Down	PF3D7_0811500	-0.63586	7.22E-02	5.9	5.4	6.2	5.3	5.3	5.3	histone-arginine methyltransferase CARM1, putative	CARM1
Down	PF3D7_1364200	-0.63528	5.86E-06	9.5	9.4	9.5	9	8.9	8.7	nucleoporin NUP205, putative	NUP205
Down	PF3D7_1003000	-0.63526	7.08E-06	9.9	9.7	9.8	9.2	9	9.3	guanylate cyclase organizer UGO, putative	N/A
Down	PF3D7_1473900	-0.63393	5.94E-03	7.2	6.9	7.3	6.5	6.5	6.7	conserved Plasmodium protein, unknown function	N/A
Down	PF3D7_1315500	-0.63384	3.90E-04	8.8	8.7	8.6	8.1	7.8	8.4	conserved protein, unknown function	N/A

Down	PF3D7_1302100	-0.63377	3.24E-04	9.3	9.1	9.6	8.9	8.7	8.6	gamete antigen 27/25	G27/25
Down	PF3D7_0612700	-0.63158	1.14E-05	11	11	11	10	10	11	6-cysteine protein P12	P12
Down	PF3D7_0410000	-0.63121	3.12E-05	11	11	12	11	11	11	erythrocyte vesicle protein 1	EVP1
Down	PF3D7_0206200	-0.631	2.34E-05	11	10	11	9.8	9.7	10	pantothenate transporter	PAT
Down	PF3D7_0408100	-0.63026	4.01E-04	9	8.7	8.9	8	8.1	8.5	conserved Plasmodium protein, unknown function	N/A
Down	PF3D7_1244500	-0.6302	5.89E-02	5.9	5.7	6.1	5.3	5.5	5.3	PIMMS57 protein	PIMMS57
Down	PF3D7_1017300	-0.6302	3.98E-05	9.3	9.3	9.4	8.5	8.6	8.9	golgi re-assembly stacking protein 1	GRASP
Down	PF3D7_1334700	-0.62966	5.97E-02	6.3	5.6	6.5	5.5	5.5	5.7	MSP7-like protein	MSRP7
Down	PF3D7_0511600	-0.62956	3.75E-03	7.2	7.3	7.4	6.9	6.5	6.6	apical rhoptry neck protein	ARNP
Down	PF3D7_0930500	-0.62919	7.22E-06	11	11	11	10	10	11	diacylglycerol kinase, putative	DGK1
Down	PF3D7_1337400	-0.62769	3.61E-02	6.3	6.1	6.3	5.8	5.4	5.7	zinc finger protein, putative	N/A
Down	PF3D7_0802600	-0.62498	6.61E-07	13	13	13	12	12	12	adenylyl cyclase beta	ACbeta
Down	PF3D7_0907500	-0.62491	4.00E-06	9.3	9.3	9.4	8.7	8.7	8.8	conserved protein, unknown function	N/A
Down	PF3D7_0404700	-0.62471	2.27E-06	13	13	13	12	12	12	dipeptidyl aminopeptidase 3	DPAP3
Down	PF3D7_1307100	-0.62453	4.35E-03	7.1	7.4	7.2	6.6	6.6	6.7	U3 small nucleolar RNA-associated protein 6, putative	UTP6
Down	PF3D7_1439500	-0.62424	3.35E-05	9.4	9.1	9.4	8.6	8.7	8.8	oocyst rupture protein 2, putative	ORP2
Down	PF3D7_0214900	-0.62417	1.02E-06	13	13	13	12	12	13	rhoptry neck protein 6	RON6
Down	PF3D7_1217400	-0.6237	1.88E-05	10	10	9.9	9.4	9.2	9.6	conserved protein, unknown function	N/A
Down	PF3D7_1467900	-0.62312	2.35E-06	12	12	12	12	11	12	rab GTPase activator, putative	N/A
Down	PF3D7_0511700	-0.62198	9.61E-02	5.7	5.6	5.9	4.7	5.2	5.5	EKC/KEOPS complex subunit CGI121	CGI121
Down	PF3D7_0108400	-0.62131	7.65E-02	5.9	5.7	5.7	5.4	5.2	5	mitochondrial carrier protein, putative	N/A
Down	PF3D7_1017100	-0.62125	5.98E-07	11	11	11	10	10	10	rhoptry neck protein 12	RON12
Down	PF3D7_0525800	-0.62122	6.61E-05	13	13	13	12	13	13	inner membrane complex protein 1g, putative	IMC1g
Down	PF3D7_1035300	-0.62117	2.14E-05	13	13	13	12	12	13	glutamate-rich protein GLURP	GLURP
Down	PF3D7_1031400	-0.61981	2.92E-05	10	11	11	9.8	9.7	10	OTU-like cysteine protease	OTU
Down	PF3D7_0922400	-0.6191	7.17E-02	5.7	5.7	6.1	5.2	5.5	5.2	para-aminobenzoic acid synthetase	pBAS

Down	PF3D7_1403500	-0.61871	1.02E-02	7.2	7.1	7.8	6.7	6.9	6.8	dynein light chain, putative	N/A
Down	PF3D7_0730900	-0.61563	5.25E-04	8.7	8.3	8.8	8.1	7.9	8.1	EMP1-trafficking protein	PTP4
Down	PF3D7_1036400	-0.61505	2.09E-02	7.2	7.4	7.6	7.2	6.9	6.2	liver stage antigen 1	LSA1
Down	PF3D7_1327300	-0.61429	2.53E-04	13	13	14	13	13	13	conserved Plasmodium protein, unknown function	N/A
Down	PF3D7_0308700	-0.61407	8.61E-05	10	10	10	9.7	9.4	9.9	conserved protein, unknown function	N/A
Down	PF3D7_0504100	-0.61368	2.35E-03	7.9	7.8	8.1	7.3	7.1	7.7	AN1-type zinc finger protein, putative	N/A
Down	PF3D7_1336700	-0.61356	2.06E-03	7.9	8	8.4	7.7	7.5	7.4	parasitophorous vacuolar protein 3, putative	PV3
Down	PF3D7_1251600	-0.6117	1.06E-02	7.1	7.1	7.6	6.9	6.7	6.5	conserved Plasmodium protein, unknown function	N/A
Down	PF3D7_1012900	-0.61065	2.90E-04	9.6	9.4	9.8	9.2	9	8.8	autophagy-related protein 18	ATG18
Down	PF3D7_1212900	-0.61049	1.41E-05	11	11	11	10	10	10	bromodomain protein 2, putative	BDP2
Down	PF3D7_1420200	-0.61024	1.64E-05	10	10	10	9.6	9.5	9.9	myosin-specific chaperone UNC, putative	UNC
Down	PF3D7_1304200	-0.61005	1.83E-04	8.5	8.4	8.6	7.9	7.8	8	CorA-like Mg2+ transporter protein, putative	MIT2
Down	PF3D7_1209500	-0.60992	8.86E-06	9.5	9.3	9.5	8.8	8.7	8.9	cGMP-specific 3',5'-cyclic phosphodiesterase alpha	PDEalpha
Down	PF3D7_0507800	-0.60859	1.01E-06	12	12	12	11	11	11	conserved protein, unknown function	N/A
Down	PF3D7_1133400	-0.60738	1.95E-05	13	13	14	13	13	13	apical membrane antigen 1	AMA1
Down	PF3D7_0821400	-0.60726	1.25E-04	9.4	9.2	9.7	8.9	8.7	8.9	conserved Plasmodium protein, unknown function	N/A
Down	PF3D7_0110800	-0.60707	2.05E-04	9.3	9.4	9.7	8.9	8.7	9.1	transcription initiation factor TFIIB, putative	N/A
Down	PF3D7_0525700	-0.60559	2.76E-03	8.7	8.6	8.7	7.8	7.8	8.5	conserved protein, unknown function	N/A
Down	PF3D7_1445400	-0.60414	5.87E-05	10	10	10	9.3	9.3	9.6	protein serine/threonine kinase-1	CLK1
Down	PF3D7_0717500	-0.6039	1.05E-05	12	12	12	11	11	12	calcium-dependent protein kinase 4	CDPK4
Down	PF3D7_1146600	-0.60352	1.61E-04	12	12	13	12	12	12	oocyst rupture protein 1, putative	ORP1
Down	PF3D7_0910500	-0.60115	5.69E-05	10	10	10	9.4	9.4	9.7	DNA repair protein REV1, putative	N/A
Down	PF3D7_0730400	-0.60096	1.95E-04	11	11	11	9.9	9.9	10	IMP1-like protein, putative	IMP2
Down	PF3D7_1459900	-0.60079	1.72E-02	7.4	7.4	6.9	6.5	6.5	7	rhoptry protein, putative	N/A

Down	PF3D7_1013100	-0.59807	1.18E-03	7.8	7.7	7.7	7.2	7.2	7.1	U3 small nucleolar RNA-associated protein 13, putative	UTP13
Down	PF3D7_1449900	-0.5978	2.48E-02	6.6	6.5	6.9	6	6.1	6.2	conserved protein, unknown function	N/A
Down	PF3D7_0806500	-0.59718	7.52E-06	11	11	11	10	9.9	10	DnaJ protein, putative	N/A
Down	PF3D7_1439300	-0.59651	6.76E-05	9.6	9.6	9.9	9.1	9	9.3	Sad1/UNC domain-containing protein, putative	N/A
Down	PF3D7_0108300	-0.59643	1.52E-06	13	13	13	12	12	12	conserved Plasmodium protein, unknown function	ARP
Down	PF3D7_0516000	-0.59581	7.20E-02	5.7	6	6.1	5.5	5.2	5.5	RAP protein, putative	N/A
Down	PF3D7_1307900	-0.59548	2.34E-05	9.1	9.2	9.2	8.6	8.5	8.7	tripartite motif protein, putative	N/A
Down	PF3D7_1012200	-0.59481	2.24E-05	11	11	11	11	11	11	rhoptry associated adhesin	RA
Down	PF3D7_1436200	-0.59468	1.21E-05	13	13	13	12	12	12	basal complex protein BCP1	BCP1
Down	PF3D7_1332200	-0.5939	9.72E-05	13	13	13	12	12	13	conserved protein, unknown function	N/A
Down	PF3D7_1339300	-0.5931	1.75E-04	10	10	11	9.7	9.8	10	conserved protein, unknown function	N/A
Down	PF3D7_0206900	-0.59299	6.25E-04	11	11	11	10	10	11	merozoite surface protein 5	MSP5
Down	PF3D7_1232500	-0.58878	9.67E-05	12	12	12	11	11	12	CG2-related protein, putative	N/A
Down	PF3D7_1335200	-0.5873	9.41E-02	5.7	5.8	6.4	5.6	5.2	5.6	reticulocyte binding protein homologue 6, pseudogene	RH6
Down	PF3D7_1307700	-0.58704	1.47E-04	10	10	11	9.7	9.6	10	TOM1-like protein, putative	N/A
Down	PF3D7_0303100	-0.58703	1.35E-04	9.4	9.3	9.4	8.9	8.5	8.9	CLP1 P-loop domain-containing protein, putative	N/A
Down	PF3D7_1238800	-0.58702	6.08E-06	11	11	11	11	10	10	acyl-CoA synthetase	ACS11
Down	PF3D7_1012100	-0.58625	7.60E-04	8.3	8.3	8.5	7.8	7.6	8	conserved protein, unknown function	N/A
Down	PF3D7_1211900	-0.58621	7.73E-06	12	12	12	11	11	11	non-SERCA-type Ca2+ -transporting P-ATPase	ATP4
Down	PF3D7_1311600	-0.58605	2.40E-04	9.4	9.1	9.4	8.7	8.6	8.9	conserved Plasmodium protein, unknown function	N/A
Down	PF3D7_1469900	-0.58577	6.25E-03	7.6	7.8	7.5	6.9	6.9	7.3	protein MGET	MGET
Down	PF3D7_1321100	-0.58567	2.14E-06	13	13	13	12	12	13	protein kinase domain-containing protein, putative	N/A

Table S3J. Differential expression between PfDNMT2 disruption and complementation at the ring stage

R: replicate, KO: PfDNMT2 disruptant, CO: PfDNMT2 complement, Adj.Pval: adjusted P value

columns G-L is EdgeR log2 (counts per million + count)

Change pattern	Gene ID	log2 Fold Change	Adj.Pval	CO R1	CO R2	CO R3	KO R1	KO R2	KO R3	Product Description	Name or Symbol
Up	PF3D7_0413300	4.613847	2.68E-02	2	2	2	3.7	3.4	3.7	rifin	RIF
Up	PF3D7_1401000	4.144377	6.87E-02	2	2	2	3.32	3.1	3.5	GBPH protein	GBPH
Up	PF3D7_0413400	3.104655	9.47E-14	3.5	4.2	3.9	6.76	6.3	6.7	erythrocyte membrane protein 1 (PfEMP1), exon 1, pseudogene	VAR
Up	PF3D7_0223400	2.297899	6.02E-03	3.7	2.5	3.2	4.8	4.9	5.1	rifin	RIF
Up	PF3D7_1478700	2.054966	2.59E-02	3.5	2.5	3.7	4.88	4.5	5.2	Plasmodium exported protein, unknown function, pseudogene	N/A
Up	PF3D7_1221500	2.050265	8.85E-02	2	3.5	2.9	4.12	3.9	4.8	heptatricopeptide repeat-containing protein, putative	N/A
Up	PF3D7_0712900	1.93897	7.86E-24	7.6	7.9	7.1	9.38	9.3	9.7	erythrocyte membrane protein 1, PfEMP1	VAR
Up	PF3D7_1442100	1.90395	3.59E-02	3.5	3.2	2.9	4.55	4.5	4.7	replication factor A protein 3, putative	RPA3
Up	PF3D7_0302300	1.884163	4.16E-08	4.7	5.7	4.9	6.62	6.9	7.2	erythrocyte membrane protein 1 (PfEMP1), pseudogene	N/A
Up	PF3D7_1334500	1.761739	3.21E-02	3.5	4	4.8	5.32	5	6.5	MSP7-like protein	MSRP6
Up	PF3D7_0504400	1.67759	9.07E-02	2	2.9	4.9	4.64	4.4	5.9	ATP-dependent helicase, putative	N/A
Up	PF3D7_0712800	1.592538	1.41E-04	4.5	4.7	5	6.3	6	6.3	erythrocyte membrane protein 1, PfEMP1	VAR
Up	PF3D7_1323600	1.588688	6.06E-02	3.7	2	4.1	4.72	4.3	5.1	conserved protein, unknown function	N/A
Up	PF3D7_0218000	1.566022	8.84E-02	2.9	2.5	4.2	4.88	3.3	5.4	replication factor C subunit 2, putative	RFC2
Up	PF3D7_0808700	1.564526	8.15E-11	5.5	6.1	5.2	7.02	7.1	7.3	erythrocyte membrane protein 1, PfEMP1	VAR
Up	PF3D7_0917500	1.550629	8.84E-02	2.5	2.9	4.2	4.24	4.2	5.2	conserved protein, unknown function	N/A
Up	PF3D7_1101700	1.536557	8.97E-02	4	3.7	3.5	5.02	4.4	5.5	Pfmc-2TM Maurer's cleft two transmembrane protein	MC-2TM

Up	PF3D7_1148000	1.476399	1.16E-02	2.5	4	4.7	4.64	4.9	6.1	serine/threonine protein kinase, putative	N/A
Up	PF3D7_1034500	1.445265	3.94E-02	3.7	3.5	4.1	4.72	4.3	5.8	armadillo repeat protein, putative	N/A
Up	PF3D7_1330600	1.423228	3.09E-02	3.5	3.9	4.3	4.64	4.6	6	elongation factor Tu, putative	N/A
Up	PF3D7_0617400	1.412134	2.14E-14	6.5	6.5	6.3	7.74	7.9	8	erythrocyte membrane protein 1, PfEMP1	VAR
Up	PF3D7_0631900	1.3813	5.57E-02	4.3	4.5	5.6	6.03	5.5	6.8	stevor	N/A
Up	PF3D7_0713800	1.361064	7.70E-02	3.7	3.9	3.7	4.12	5	5.2	negative elongation factor A, putative	NELFA
Up	PF3D7_1240600	1.282282	1.80E-08	9.8	10	9.6	11.2	11	11	erythrocyte membrane protein 1, PfEMP1	VAR
Up	PF3D7_1111100	1.26402	3.72E-02	4	3.7	4.6	4.72	4.8	6	replication factor C subunit 5, putative	RFC5
Up	PF3D7_1017000	1.232449	3.94E-02	3.9	3.5	4.8	4.88	4.9	5.8	DNA polymerase delta catalytic subunit	N/A
Up	PF3D7_0808600	1.182108	2.82E-11	9.1	9.3	8.9	10.2	10	10	erythrocyte membrane protein 1, PfEMP1	VAR
Up	PF3D7_0221800	1.151241	7.03E-02	4.4	4.6	4.1	5.52	5.5	4.9	hypothetical protein	N/A
Up	PF3D7_0303700	1.133661	4.78E-02	3.7	4	5	5.02	4.8	6.2	lipoamide acyltransferase component of branched-chain alpha-keto acid dehydrogenase complex	BCKDH-E2
Up	PF3D7_1463200	1.131168	8.92E-02	3.7	3.5	5	5.14	4.5	5.8	replication factor C subunit 3, putative	RFC3
Up	PF3D7_1359600	1.129185	5.57E-02	6.1	6	7.2	7.41	7.3	8	conserved Plasmodium protein, unknown function	N/A
Up	PF3D7_1219300	1.119733	2.59E-03	5	5.8	5.1	6.35	6.1	6.7	erythrocyte membrane protein 1, PfEMP1	VAR
Up	PF3D7_0413100	1.062174	8.91E-02	4	5	4.2	5.52	5.2	5.5	erythrocyte membrane protein 1, PfEMP1	VAR
Up	PF3D7_1219400	1.039701	1.05E-05	7	7.5	6.5	7.85	8.1	8.3	erythrocyte membrane protein 1 (PfEMP1), pseudogene	VAR
Up	PF3D7_1001200	1.017805	9.92E-02	5.3	5.4	5.4	6.4	6.1	6.4	acyl-CoA binding protein, isoform 2, ACBP2	ACBP2
Up	PF3D7_0712600	0.992418	5.72E-06	7.8	7.8	7.3	8.51	8.5	8.9	erythrocyte membrane protein 1, PfEMP1	VAR

Up	PF3D7_0413700	0.985611	1.52E-02	4.5	5	5.7	5.89	5.8	6.5	lysine decarboxylase-like protein, putative	N/A
Up	PF3D7_0712300	0.956691	3.72E-02	7.3	7.2	6.9	7.89	7.9	8.4	erythrocyte membrane protein 1, PfEMP1	VAR
Up	PF3D7_1366000	0.954866	3.38E-02	5.1	5.1	5.4	6.43	5.7	6.1	conserved Plasmodium protein, unknown function	N/A
Up	PF3D7_0814100	0.89214	2.82E-02	5.8	5.2	5.6	6.59	6.4	6	conserved Plasmodium protein, unknown function	N/A
Up	PF3D7_0712000	0.885344	1.05E-05	8.2	8.1	7.9	9	8.8	9	erythrocyte membrane protein 1, PfEMP1	VAR
Up	PF3D7_0912200	0.884992	7.11E-02	5.3	5.4	5.4	5.99	6.3	6.2	conserved protein, unknown function	N/A
Up	PF3D7_1436900	0.879255	6.56E-02	5	5	5.2	6.09	5.8	5.7	histidine triad protein, putative	N/A
Up	PF3D7_0420700	0.872074	3.82E-03	6.1	6.6	6.2	6.88	7.1	7.4	erythrocyte membrane protein 1, PfEMP1	VAR
Up	PF3D7_1349700	0.851582	5.77E-02	5.2	5	5.3	6.12	5.7	6	peptidase, putative	N/A
Up	PF3D7_1312700	0.840493	3.72E-02	5.3	5.3	5.7	6.21	6.1	6.4	conserved Plasmodium protein, unknown function	N/A
Up	PF3D7_1324300	0.798818	4.16E-06	6.4	7	6.9	7.51	7.7	7.4	conserved Plasmodium membrane protein, unknown function	N/A
Up	PF3D7_1106300	0.7412	8.84E-02	5.2	5.3	5.6	5.78	5.9	6.5	5'-3' exoribonuclease 1, putative	XRN1
Up	PF3D7_0626100	0.720334	1.28E-02	6.2	5.7	6.4	6.68	6.9	6.9	oxidoreductase, short-chain dehydrogenase family, putative	N/A
Up	PF3D7_1419000	0.715612	5.52E-02	5.3	5.4	6	6.21	6.1	6.5	conserved Plasmodium protein, unknown function	N/A
Up	PF3D7_0204600	0.68932	6.87E-02	5.4	5.8	5.8	6.18	6.4	6.3	5'-3' exonuclease, putative	N/A
Up	PF3D7_0824400	0.682167	3.38E-02	6	5.8	6.3	6.48	6.5	7.2	nucleoside transporter 2	NT2
Up	PF3D7_0110700	0.671894	3.38E-02	6.5	6.4	6.7	7.26	6.9	7.5	chromatin assembly factor 1 subunit C, putative	CAF1C
Up	PF3D7_0704300	0.623377	7.70E-02	5.9	6.1	6.2	6.62	6.6	6.8	conserved Plasmodium membrane protein, unknown function	N/A
Up	PF3D7_0917000	0.592351	2.83E-02	6.6	6.5	6.9	7	7.3	7.4	merozoite organizing protein	MOP
Down	PF3D7_0809100	-4.367193	4.17E-297	13	13	13	8.92	8.7	9.1	erythrocyte membrane protein 1, PfEMP1	VAR

Down	PF3D7_1306000	-4.067973	7.03E-02	3.2	4	3.5	2.46	2	2	conserved Plasmodium protein, unknown function	N/A
Down	PF3D7_1147200	-4.029539	9.92E-02	2.5	3.5	2.9	2	2	2	tubulin--tyrosine ligase, putative	N/A
Down	PF3D7_1101300	-3.912658	2.01E-10	6.8	5.4	6.3	3.52	3	2.9	rifin	RIF
Down	PF3D7_0727300	-3.4894	3.27E-10	13	12	13	9.4	9.8	9.7	DNA (cytosine-5)-methyltransferase	DNMT
Down	PF3D7_0713300	-3.259626	3.86E-151	11	11	11	7.88	7.9	7.9	erythrocyte membrane protein 1 (PfEMP1), pseudogene	VAR
Down	PF3D7_0700800	-3.02324	4.08E-07	6.1	4.9	6.1	3.52	3	3.7	Pfmc-2TM Maurer's cleft two transmembrane protein	MC-2TM
Down	PF3D7_0713200	-2.986306	2.00E-38	8.7	9	9.1	6.24	6.1	5.7	exported protein family 1	EPF1
Down	PF3D7_1121300	-2.357823	3.94E-02	3.2	3.5	5	2	2.8	3.3	tyrosine kinase-like protein	TKL2
Down	PF3D7_0713100	-2.289724	6.32E-10	6.6	5.7	6.2	4.24	4.2	4.1	Pfmc-2TM Maurer's cleft two transmembrane protein	MC-2TM
Down	PF3D7_0937600	-1.775205	1.05E-02	4.6	5	4.8	2	3.5	4.2	erythrocyte membrane protein 1, PfEMP1	VAR
Down	PF3D7_1141100	-1.314557	1.14E-03	6	6.4	6	4.45	5.2	5	conserved Plasmodium protein, unknown function	N/A
Down	PF3D7_0632800	-1.254442	9.76E-06	6.9	7.3	6.6	5.47	5.9	5.9	erythrocyte membrane protein 1, PfEMP1	VAR
Down	PF3D7_0211900	-1.108293	7.03E-02	4.9	4.6	5.6	3.32	4.2	4.7	pseudouridine synthase, putative	N/A
Down	PF3D7_0833500	-0.840559	1.97E-02	6.5	6.2	6.4	5.81	5.5	5.5	erythrocyte membrane protein 1, PfEMP1	VAR
Down	PF3D7_0823600	-0.819031	3.59E-02	6.3	6.5	6.2	5.42	5.8	5.3	lipoate-protein ligase B	LipB
Down	PF3D7_0621100	-0.786874	5.10E-02	6.2	6	6.5	5.78	5.4	5.2	conserved Plasmodium protein, unknown function	N/A
Down	PF3D7_1350900	-0.756689	1.52E-02	6.9	7.3	7.1	6.59	6.3	6.3	AP2 domain transcription factor AP2-O4, putative	ApiAP2
Down	PF3D7_1125000	-0.694064	9.71E-02	6	6.2	6	5.42	5.1	5.8	conserved protein, unknown function	N/A
Down	PF3D7_0412700	-0.683654	4.89E-02	9.4	8.4	9.2	8.22	8.4	8.6	erythrocyte membrane protein 1, PfEMP1	VAR
Down	PF3D7_1428700	-0.670119	3.50E-02	6.6	7.1	6.9	6.5	6.1	6.1	heme/steroid binding domain containing protein, putative	N/A

Down	PF3D7_1031300	-0.631023	8.84E-02	6.3	6.3	6.3	5.52	5.7	5.9	SAE2 domain-containing protein, putative	N/A
Down	PF3D7_0401800	-0.611638	3.25E-02	8.6	8.2	7.8	7.73	7.4	7.7	Plasmodium exported protein (PHISTb), unknown function	PfD80
Down	PF3D7_0218200	-0.608405	2.77E-02	6.6	7.2	6.9	6.09	6.5	6.4	SUZ domain-containing protein, putative	N/A
Down	PF3D7_1131100	-0.5957	8.52E-02	6.9	7	6.7	6.15	6.4	6.3	serpentine receptor 1, putative	SR1
Down	PF3D7_1017300	-0.589191	9.10E-02	7.1	6.7	6.7	6.15	6.5	6.3	golgi re-assembly stacking protein 1	GRASP

Table S3K. Differential expression between PfDNMT2 disruption and complementation at the trophozoite stage

R: replicate, KO: PfDNMT2 disruptant, CO: PfDNMT2 complement, Adj.Pval: adjusted P value

columns G-L is EdgeR log2 (counts per million + count)

Change pattern	Gene ID	log2 Fold Change	Adj.Pval	CO R1	CO R2	CO R3	KO R1	KO R2	KO R3	Product Description	Name or Symbol
Up	PF3D7_1350600	3.946957	1.24E-02	2	2	2	2.6	2.2	2.6	conserved Plasmodium protein, unknown function	N/A
Up	PF3D7_0411600	3.48471	3.99E-02	2	2	2	2.5	2.2	2.5	tRNA Glutamic acid	N/A
Up	PF3D7_1003900	3.382659	6.79E-02	2	2	2	2.5	2.8	2.7	dynein attachment factor-domain containing protein, putative	N/A
Up	PF3D7_0532900	3.289615	5.09E-02	2	2	2	2.5	2.2	2.4	rifin	RIF
Up	PF3D7_1236500	3.161363	6.38E-02	2	2	2	2.1	2.4	2.4	conserved Plasmodium protein, unknown function	N/A
Up	PF3D7_1404700	3.154786	6.00E-02	2	2	2	2.2	2	2.5	cysteine-rich small secreted protein CSS, putative	N/A
Up	PF3D7_1104500	2.752839	5.33E-02	2.3	2	2	2.6	3	2.7	WD repeat-containing protein, putative	N/A
Up	PF3D7_1401000	2.605697	5.89E-10	2.9	3	3	5	4.8	4.9	GBPH protein	GBPH
Up	PF3D7_0511700	2.453931	2.31E-05	2.7	2	3	3.8	4.2	4.1	EKC/KEOPS complex subunit CGI121	CGI121
Up	PF3D7_1346200	2.189026	3.15E-02	2	2	3	3.5	2.8	3.3	nuclear import protein MOG1, putative	N/A
Up	PF3D7_0512900	2.07291	1.16E-04	3	3	3	4	5	4.7	AKAP-like protein	AKAL
Up	PF3D7_0601600	1.874437	1.64E-02	2.3	3	2	3.3	3.6	3.1	tetratricopeptide repeat protein, putative	N/A
Up	PF3D7_0926200	1.773912	3.87E-04	3.7	3	3	4.4	5.1	4.8	conserved Plasmodium protein, unknown function	N/A
Up	PF3D7_1462500	1.70726	3.92E-02	2.5	2	3	3.3	3.5	3.3	conserved Plasmodium protein, unknown function	N/A
Up	PF3D7_0916900	1.698713	8.11E-05	5.6	5	5	6.6	7.1	7.1	zinc finger protein, putative	N/A
Up	PF3D7_0506400	1.629771	8.74E-02	2	3	2	3.1	3	2.6	conserved Plasmodium protein, unknown function	N/A
Up	PF3D7_1242100	1.623816	8.13E-03	3.2	3	3	4.1	4.6	4.4	conserved Plasmodium protein, unknown function	N/A
Up	PF3D7_0107700	1.612466	2.05E-05	3.5	4	4	4.8	5.1	4.8	dolichyl-diphosphooligosaccharide--protein glycosyltransferase subunit OST3/OST6, putative	N/A
Up	PF3D7_1400100	1.611572	6.54E-02	2.5	3	2	3	2.4	3.2	erythrocyte membrane protein 1 (PfEMP1), pseudogene	N/A
Up	PF3D7_1364700	1.6115	6.87E-03	2.9	3	3	3.8	3.9	3.8	WD repeat-containing protein, putative, pseudogene	N/A

Up	PF3D7_1133000	1.604378	2.86E-02	2.9	2	2	3	3.3	3.5	conserved Plasmodium protein, unknown function	N/A
Up	PF3D7_0915500	1.578506	4.77E-02	2.7	3	3	3.5	3.8	3.3	conserved Plasmodium protein, unknown function	N/A
Up	PF3D7_1137700	1.57383	9.03E-02	3.2	2	2	3.5	3.3	3.1	calcium-binding protein, putative	N/A
Up	PF3D7_1315000	1.536659	1.37E-03	3.8	4	3	4.5	5	5	conserved protein, unknown function	N/A
Up	PF3D7_0620300	1.491318	2.85E-04	3.4	3	3	3.8	4.6	4.3	conserved Plasmodium protein, unknown function	N/A
Up	PF3D7_0626200	1.450239	4.42E-02	3.4	2	3	4.1	4.3	3.8	conserved Plasmodium protein, unknown function	N/A
Up	PF3D7_1252400	1.443125	8.77E-02	3.4	4	5	5	4.8	6.1	reticulocyte binding protein homologue 3, pseudogene	RH3
Up	PF3D7_1210300	1.431773	1.85E-04	3.8	3	4	4.5	4.7	5	conserved Plasmodium protein, unknown function	N/A
Up	PF3D7_0811800	1.419294	1.28E-05	4.5	4	4	5.4	5.7	5.5	conserved Plasmodium protein, unknown function	N/A
Up	PF3D7_1014100	1.410859	3.05E-02	3.4	4	4	4.7	4.1	5.5	merozoite surface protein MSA180	MSA180
Up	PF3D7_0906400	1.361092	5.10E-02	2.7	3	3	3	3.7	3.4	dynein intermediate light chain, putative	N/A
Up	PF3D7_1434900	1.35784	2.71E-02	3	3	2	3.4	3.3	3.4	conserved Plasmodium protein, unknown function	N/A
Up	PF3D7_0727600	1.353141	6.22E-08	4.1	4	4	5.4	5.5	5.5	conserved Plasmodium protein, unknown function	N/A
Up	PF3D7_1122300	1.338278	6.48E-04	3.6	3	3	3.8	4.6	4.4	conserved Plasmodium protein, unknown function	N/A
Up	PF3D7_0404000	1.309796	9.47E-02	2.5	2	3	3	3	3	conserved Plasmodium protein, unknown function	N/A
Up	PF3D7_1101700	1.281973	1.86E-02	3	3	3	4.4	3.6	3.8	Pfmc-2TM Maurer's cleft two transmembrane protein	MC-2TM
Up	PF3D7_0934200	1.273018	6.91E-04	4.1	4	4	4.5	5	4.9	conserved protein, unknown function	N/A
Up	PF3D7_1370800	1.26789	7.22E-15	5.7	5	6	6.6	6.6	6.5	non-coding RNA	N/A
Up	PF3D7_1416700	1.17048	9.38E-02	2.7	3	3	3.8	3.5	3.4	conserved protein, unknown function	N/A
Up	PF3D7_0507500	1.169357	3.20E-02	3.2	4	4	4.5	4.4	5.3	subtilisin-like protease 1	SUB1
Up	PF3D7_1446000	1.16737	5.65E-02	2.9	3	3	3.7	2.9	3.4	U5 spliceosomal RNA	N/A
Up	PF3D7_1205200	1.162549	8.82E-02	2.9	3	3	2.7	3.5	3.4	HAD domain ookinete protein, putative	HADO
Up	PF3D7_1001300	1.157605	3.60E-03	3.6	4	5	5.3	5.1	5.2	Plasmodium exported protein (PHISTa), unknown function	N/A
Up	PF3D7_1354100	1.151366	7.98E-03	3.7	3	3	4.2	4.5	4.1	conserved Plasmodium protein, unknown function	N/A
Up	PF3D7_1018400	1.126265	5.62E-02	2.7	3	3	3.7	3.1	3.6	conserved Plasmodium protein, unknown function	N/A
Up	PF3D7_1303300	1.121082	1.30E-04	4.5	4	4	5.1	5.3	5.4	conserved Plasmodium protein, unknown function	N/A

Up	PF3D7_0829700	1.116318	4.64E-02	3.2	4	3	3.8	4.2	4	non-coding RNA	N/A
Up	PF3D7_1135000	1.106282	4.78E-06	5.2	5	5	5.9	6.1	6.1	PQ-loop repeat-containing protein	N/A
Up	PF3D7_1037400	1.101043	5.44E-05	4.5	5	5	5.5	5.8	5.6	conserved Plasmodium protein, unknown function	N/A
Up	PF3D7_0308200	1.096911	2.31E-05	7.8	8	9	9	9.3	9.1	T-complex protein 1 subunit eta	CCT7
Up	PF3D7_0905500	1.089073	7.70E-04	4.2	4	3	4.3	4.8	4.9	conserved protein, unknown function	N/A
Up	PF3D7_1339800	1.086196	1.63E-02	3.4	4	3	4.2	4.3	4.1	mitotic-spindle organizing protein 1, putative	MZT1
Up	PF3D7_1456100	1.084587	4.45E-02	3	3	3	3.7	3.7	4	serine hydroxymethyltransferase	SHMT
Up	PF3D7_1422200	1.0779	1.32E-06	4.6	5	4	5.2	5.3	5.5	conserved Plasmodium protein, unknown function	N/A
Up	PF3D7_1438600	1.068526	1.14E-04	4.7	5	5	5.9	6	6.1	Golgi to ER traffic protein 4	GET4
Up	PF3D7_1428000	1.063958	5.59E-03	3.2	4	3	4.1	4.6	4.3	conserved Plasmodium membrane protein, unknown function	N/A
Up	PF3D7_0934600	1.062182	6.83E-02	2.7	3	3	3.9	3.1	3.7	conserved Plasmodium protein, unknown function	N/A
Up	PF3D7_0211000	1.058799	1.79E-02	3.7	4	4	4.3	4.5	4.5	non-coding RNA	N/A
Up	PF3D7_1328600	1.056171	4.70E-02	3.2	3	3	3.8	3.5	3.3	conserved Plasmodium protein, unknown function	N/A
Up	PF3D7_1310000	1.054616	2.87E-03	4.1	4	4	4.5	4.8	4.9	ATP synthase subunit O, mitochondrial, putative	OSCP
Up	PF3D7_1370500	1.05458	3.98E-07	6.7	6	6	7.3	7.3	7.2	non-coding RNA	N/A
Up	PF3D7_0506600	1.049735	5.31E-02	3	3	3	3.6	3.7	3.9	histone deacetylase, putative, pseudogene	N/A
Up	PF3D7_1476700	1.044574	1.31E-02	3.7	4	3	4.4	4.1	4.7	lysophospholipase LPL1	LPL1
Up	PF3D7_0525300	1.043629	3.16E-03	3.6	4	3	4.3	4.5	4.4	conserved protein, unknown function	N/A
Up	PF3D7_0301900	1.042807	9.33E-03	3.6	4	4	4.3	4.5	4.4	conserved protein, unknown function	N/A
Up	PF3D7_1349800	1.027678	1.00E-03	4.4	4	4	5.3	5.2	5.1	GPN-loop GTPase, putative	N/A
Up	PF3D7_1148200	1.0259	4.92E-07	6.9	6	7	7.6	7.5	7.5	non-coding RNA	N/A
Up	PF3D7_0412200	1.024011	2.32E-03	3.5	4	4	4.6	4.6	4.7	conserved Plasmodium protein, unknown function	N/A
Up	PF3D7_1217500	1.022563	3.88E-02	6.6	7	7	7.5	8	7.8	conserved Plasmodium protein, unknown function	N/A
Up	PF3D7_0829800	1.020646	4.49E-04	6.4	6	7	7.6	7.7	7.8	non-coding RNA	N/A
Up	PF3D7_1363900	1.019639	4.97E-02	5	5	4	5.2	6.1	5.7		
Up	PF3D7_0612300	1.008549	8.64E-02	2.9	3	3	3.7	3.4	3.7	transmembrane protein 234, putative	TMEM234
Up	PF3D7_0526100	1.007802	8.47E-02	4.1	3	4	4.3	4.6	4.9	conserved Plasmodium membrane protein, unknown function	N/A

Up	PF3D7_1022200	0.991987	2.12E-08	6.8	7	7	7.7	7.9	7.9	conserved Plasmodium membrane protein, unknown function	N/A
Up	PF3D7_0507400	0.991331	7.94E-02	2.9	3	3	3	3.4	3.6	subtilisin propeptide-like protein	SUB1-ProM
Up	PF3D7_1401100	0.977707	3.06E-03	4.4	4	5	5.5	5.2	5.5	DnaJ protein, putative	N/A
Up	PF3D7_0809800	0.963279	3.18E-02	3.3	3	3	3.8	4.1	3.8	conserved Plasmodium protein, unknown function	N/A
Up	PF3D7_1137000	0.962388	3.86E-02	3.3	3	3	3.9	3.6	3.7	U2 spliceosomal RNA	N/A
Up	PF3D7_0703100	0.949615	6.52E-03	3.6	4	4	4.5	4.4	4.7	cytosolic iron-sulfur assembly component 2, putative	CIA2
Up	PF3D7_1351800	0.93447	2.84E-03	4.4	4	5	5.1	5.3	5.2	conserved Plasmodium protein, unknown function	N/A
Up	PF3D7_0725600	0.932686	2.15E-02	5.9	6	7	7.2	7.2	7	18S ribosomal RNA	N/A
Up	PF3D7_0822200	0.917805	3.36E-02	3.2	4	3	3.9	4.2	4	phosphorylated CTD interacting factor 1 WW domain-containing protein, putative	N/A
Up	PF3D7_1016200	0.915249	4.11E-04	5.4	5	5	5.9	6.2	6.1	Rab3 GTPase-activating protein non-catalytic subunit, putative	N/A
Up	PF3D7_1469500	0.904821	6.10E-02	3.3	3	3	4	3.8	3.7	conserved Plasmodium protein, unknown function	N/A
Up	PF3D7_0207900	0.904031	2.51E-02	4.8	5	6	6.2	6.7	6.2	serine repeat antigen 2	SERA2
Up	PF3D7_1347300	0.902239	1.74E-04	4.9	5	4	5.4	5.2	5.4	conserved Plasmodium membrane protein, unknown function	N/A
Up	PF3D7_0823700	0.899085	1.98E-02	4.6	4	4	5.1	5	5.4	mitochondrial import receptor subunit TOM7, putative	TOM7
Up	PF3D7_1304300	0.89869	4.06E-02	3.5	3	3	4.1	3.9	3.9	conserved Plasmodium protein, unknown function	N/A
Up	PF3D7_0829900	0.897967	1.33E-04	6.5	7	7	7.6	7.8	7.6	non-coding RNA	N/A
Up	PF3D7_0814100	0.896742	7.03E-03	3.7	4	4	4.4	4.5	4.6	conserved Plasmodium protein, unknown function	N/A
Up	PF3D7_0515100	0.890213	1.70E-02	5	6	6	6.2	6.5	6.4	rhomboid protease ROM9	ROM9
Up	PF3D7_0621000	0.887866	1.19E-02	6.6	7	7	7.4	7.5	7.5	conserved Plasmodium protein, unknown function	N/A
Up	PF3D7_0218800	0.881735	7.59E-06	5.1	6	6	6.6	6.5	6.4	ribonuclease P	N/A
Up	PF3D7_0533100	0.880801	5.08E-03	5.5	6	6	6.6	6.8	6.5	erythrocyte membrane protein 1 (PfEMP1), pseudogene	VAR1CSA
Up	PF3D7_1117300	0.879961	4.92E-07	5.9	5	5	6.2	6.4	6.6	conserved protein, unknown function	N/A
Up	PF3D7_1324600	0.875478	9.96E-05	6.2	6	7	7.1	7.7	7.3	conserved Plasmodium protein, unknown function	N/A

Up	PF3D7_1005800	0.870254	7.79E-03	3.8	4	4	4.3	4.3	4.6	conserved Plasmodium membrane protein, unknown function	N/A
Up	PF3D7_0210700	0.869028	2.86E-02	3.5	3	4	4.1	4.4	4.4	syntaxin, Qa-SNARE family	SYN17
Up	PF3D7_1479000	0.868906	8.83E-02	3.3	2	4	3.6	3.6	4	acyl-CoA synthetase	ACS1a
Up	PF3D7_1313900	0.866435	2.63E-03	4.9	5	5	5.4	5.7	5.8	ankyrin-repeat protein, putative	N/A
Up	PF3D7_1129850	0.864986	1.95E-02	3.9	4	4	4.3	4.6	4.7	UNC-50 protein, putative, pseudogene	N/A
Up	PF3D7_0931200	0.862257	3.77E-03	4.9	5	5	5.1	5.9	5.6	selenoprotein	Sel2
Up	PF3D7_0935300	0.858511	9.96E-05	4.7	5	5	5.9	5.7	5.8	phosphatidylinositol N-acetylglucosaminyltransferase subunit P, putative	N/A
Up	PF3D7_1473400	0.857642	1.34E-02	3.8	4	4	4.4	4.5	4.8	conserved protein, unknown function	N/A
Up	PF3D7_1139900	0.855709	3.81E-04	6.1	6	6	7	6.9	6.7	ER membrane protein complex subunit 8, putative	EMC8
Up	PF3D7_1022300	0.847791	4.46E-03	4.6	5	5	5.7	5.4	5.3	ZIP domain-containing protein, putative	ZIPCO
Up	PF3D7_0916600	0.839826	9.90E-03	4.1	4	4	5	4.8	4.9	methyltransferase, putative	N/A
Up	PF3D7_0808400	0.8297	1.46E-02	5.5	6	5	6.2	6.7	6.3	coatomer subunit epsilon, putative	SEC28
Up	PF3D7_0608310	0.829357	2.81E-04	6.1	6	6	6.8	6.9	6.9	sorting assembly machinery 50 kDa subunit, putative	SAM50
Up	PF3D7_1313200	0.8225	5.76E-03	4.5	4	4	4.9	5.1	5.4	methionyl-tRNA formyltransferase, putative	MTFMT
Up	PF3D7_0109300	0.821915	1.17E-02	3.7	5	5	5.1	5.1	5.1	elongation of fatty acids protein, putative	N/A
Up	PF3D7_1245200	0.817139	5.63E-06	5.5	6	6	6.4	6.6	6.6	conserved Plasmodium membrane protein, unknown function	N/A
Up	PF3D7_0707500	0.815817	4.85E-02	5.1	5	5	5.9	6.3	6	conserved Plasmodium protein, unknown function	N/A
Up	PF3D7_0303600	0.812961	1.60E-02	4	4	4	4.6	4.7	4.8	plasmoredoxin	Plrx
Up	PF3D7_1370900	0.811292	9.86E-06	6.5	6	6	6.9	6.8	6.8	non-coding RNA	N/A
Up	PF3D7_1407100	0.805579	5.99E-04	6.7	7	7	7.5	7.8	7.5	rRNA 2'-O-methyltransferase fibrillarin, putative	NOP1
Up	PF3D7_1119900	0.804991	6.27E-08	7.6	8	8	8.5	8.8	8.6	protein transport protein SEC16, putative	SEC16
Up	PF3D7_0630500	0.801808	1.80E-03	5.3	5	6	6.2	6.2	6.1	ribosome biogenesis protein YTM1, putative	YTM1
Up	PF3D7_1037900	0.799955	4.85E-02	3.4	4	3	4.1	3.8	4.3	conserved Plasmodium protein, unknown function	N/A
Up	PF3D7_1111700	0.799328	5.54E-04	5.3	5	5	5.7	5.9	6	conserved Plasmodium protein, unknown function	N/A
Up	PF3D7_1368500	0.797744	4.51E-03	4.4	4	4	4.8	5	5	heptatricopeptide repeat-containing protein, putative	N/A

Up	PF3D7_1409000	0.79538	1.51E-08	6	6	6	6.7	6.9	6.8	WD repeat-containing protein, putative	N/A
Up	PF3D7_1111000	0.792285	8.09E-05	5.1	5	5	5.9	5.8	5.9	RNA cytosine C(5)-methyltransferase NSUN2	NSUN2
Up	PF3D7_0407000	0.786276	1.59E-03	4.5	5	5	4.9	5.4	5.3	conserved Plasmodium protein, unknown function	N/A
Up	PF3D7_1459300	0.786149	1.74E-02	3.6	4	4	4.5	4.8	4.6	OPA3-like protein, putative	N/A
Up	PF3D7_1007500	0.785447	2.71E-02	4.9	5	5	5.2	5.6	5.7	conserved Plasmodium protein, unknown function	N/A
Up	PF3D7_0419200	0.780844	1.53E-03	4.5	5	5	5.3	5.3	5.6	protein transport protein GOT1, putative	GOT1
Up	PF3D7_1335600	0.7808	6.52E-03	5.4	5	5	5.4	6	5.8	WD repeat-containing protein, putative	N/A
Up	PF3D7_0510400	0.780726	4.59E-03	4.8	4	5	5.3	5.3	5.4	PDCD2 domain-containing protein, putative	N/A
Up	PF3D7_0526400	0.779344	5.58E-04	5.5	5	5	5.8	6	6	conserved Plasmodium protein, unknown function	N/A
Up	PF3D7_0110900	0.778589	8.93E-03	4.7	5	5	5.4	5.3	5.1	adenylate kinase-like protein 1	AKLP1
Up	PF3D7_1208500	0.777561	6.21E-05	5.3	5	6	6	6	6.3	conserved Plasmodium protein, unknown function	N/A
Up	PF3D7_0921100	0.774111	9.34E-02	3.5	4	4	4.1	4.3	4.3	conserved Plasmodium protein, unknown function	N/A
Up	PF3D7_0618800	0.769135	5.88E-04	5.5	5	5	5.8	6.1	5.9	heptatricopeptide repeat-containing protein, putative	N/A
Up	PF3D7_0318200	0.76912	6.60E-04	9.6	10	10	10	11	11	DNA-directed RNA polymerase II subunit RPB1	RPB1
Up	PF3D7_1342200	0.767809	7.89E-02	3.4	4	4	4.6	4.3	4.6	conserved Plasmodium membrane protein, unknown function	N/A
Up	PF3D7_1422900	0.766774	9.63E-02	3.2	4	3	4	4.1	4.1	14-3-3 protein, putative	N/A
Up	PF3D7_1133500	0.765062	1.94E-02	4.4	5	4	4.8	5.1	5	conserved protein, unknown function	N/A
Up	PF3D7_0525400	0.76409	2.05E-05	5.9	5	6	6.7	6.4	6.5	7-helix-1 protein	N/A
Up	PF3D7_0109000	0.754446	7.41E-02	3.8	3	4	4.2	4.2	4.7	photosensitized INA-labeled protein PHIL1	PHIL1
Up	PF3D7_1369400	0.753432	8.18E-07	6.7	7	7	7.3	7.7	7.5	conserved Plasmodium protein, unknown function	N/A
Up	PF3D7_1122100	0.749368	1.10E-06	6.2	6	6	6.9	6.7	6.8	GPI transamidase component GPI16, putative	GPI16
Up	PF3D7_0626600	0.74791	2.86E-02	4.1	4	4	4.7	4.6	4.5	conserved Plasmodium protein, unknown function	N/A
Up	PF3D7_1469400	0.740834	1.10E-03	5.2	5	5	6.1	5.6	5.9	nucleoside transporter 3, putative	NT3
Up	PF3D7_1456300	0.736942	6.65E-02	3.9	4	4	4.6	4.5	4.2	conserved Plasmodium protein, unknown function	N/A
Up	PF3D7_0418600	0.735943	4.23E-02	4.6	5	5	5.4	5.1	6	regulator of chromosome condensation, putative	N/A
Up	PF3D7_1441700	0.735648	1.07E-02	4.6	4	4	4.8	5.2	5	mitochondrial inner membrane protease ATP23, putative	ATP23

Up	PF3D7_1236300	0.734645	1.16E-04	5.7	6	5	6.1	6.3	6.1	conserved protein, unknown function	N/A
Up	PF3D7_1016000	0.73285	2.71E-02	4.8	4	5	5	5.2	5.3	conserved Plasmodium protein, unknown function	N/A
Up	PF3D7_1459200	0.732232	3.31E-05	6.4	6	7	7	7.4	7.3	WD repeat-containing protein, putative	N/A
Up	PF3D7_0219300	0.729748	6.63E-03	4.6	5	5	5.1	5.5	5.4	conserved Plasmodium protein, unknown function	N/A
Up	PF3D7_0908100	0.72921	3.75E-02	3.5	4	4	4.3	4.4	4.1	apicoplast integral membrane protein, putative	N/A
Up	PF3D7_1447400	0.719598	3.63E-02	4.3	4	5	4.7	4.7	4.9	conserved Plasmodium protein, unknown function	N/A
Up	PF3D7_0930200	0.715766	6.82E-02	4.2	3	4	4.2	4.6	4.8	leucine-rich repeat protein	LRR8
Up	PF3D7_1139200	0.714069	3.05E-02	3.9	4	4	4.7	4.5	4.8	VPS9 domain-containing protein, putative	N/A
Up	PF3D7_0623700	0.713846	6.44E-06	6.6	6	7	7.6	7.3	7.6	ATP-dependent RNA helicase SUV3, putative	SUV3
Up	PF3D7_0806400	0.71317	2.69E-02	4.3	4	5	4.7	5.1	4.9	UDP-N-acetylglucosamine transferase subunit ALG13, putative	ALG13
Up	PF3D7_0826600	0.711814	1.32E-03	4.8	5	5	5.3	5.3	5.5	SNARE protein, putative	VAMP7
Up	PF3D7_0506000	0.706082	7.95E-03	4.5	5	4	4.8	5.1	5.1	conserved Plasmodium protein, unknown function	N/A
Up	PF3D7_1448100	0.704568	5.27E-02	3.8	4	4	4.4	4.5	4.6	conserved Plasmodium protein, unknown function	N/A
Up	PF3D7_0223500	0.703374	5.71E-02	4	4	3	4.1	3.9	4.4	erythrocyte membrane protein 1, PfEMP1	VAR
Up	PF3D7_0610700	0.703273	4.43E-05	5.7	6	6	6.4	6.3	6.4	conserved Plasmodium protein, unknown function	N/A
Up	PF3D7_1410000	0.699093	5.62E-02	6.1	6	6	6.9	7	7	ER membrane protein complex subunit 2, putative	EMC2
Up	PF3D7_0628700	0.697987	1.60E-04	6.1	6	6	6.7	6.6	6.6	conserved Plasmodium protein, unknown function	N/A
Up	PF3D7_1216100	0.697748	5.34E-03	5.1	5	5	5.3	5.4	5.4	conserved protein, unknown function	N/A
Up	PF3D7_1456200	0.696198	1.23E-03	5.2	5	5	5.9	5.9	5.9	conserved Plasmodium protein, unknown function	N/A
Up	PF3D7_0929700	0.695442	4.14E-02	4.6	4	4	4.9	4.9	5	conserved Plasmodium protein, unknown function	N/A
Up	PF3D7_1119700	0.69496	2.73E-02	3.7	4	4	4.2	4.3	4.5	conserved Plasmodium protein, unknown function	N/A
Up	PF3D7_0514800	0.692904	1.18E-05	6.7	7	7	7.4	7.5	7.5	inositol polyphosphate multikinase, putative	N/A
Up	PF3D7_1436900	0.69269	1.25E-02	4.2	4	4	5	4.6	4.9	histidine triad protein, putative	N/A
Up	PF3D7_0912600	0.689943	7.15E-02	3.5	4	4	4.4	4.6	4.5	conserved Plasmodium protein, unknown function	N/A
Up	PF3D7_1462900	0.689247	1.36E-02	4.8	5	5	5.7	5.6	5.4	AAA ATPase, putative	N/A
Up	PF3D7_1458800	0.687438	1.83E-04	5.8	5	6	6.3	6.3	6.5	DNA-directed RNA polymerase III subunit RPC5, putative	N/A
Up	PF3D7_1017800	0.687309	1.98E-06	6.5	6	6	7.1	7.1	7.1	conserved Plasmodium protein, unknown function	N/A

Up	PF3D7_1113200	0.687279	1.11E-02	4.5	4	4	4.9	5	5.1	conserved Plasmodium protein, unknown function	N/A
Up	PF3D7_0407500	0.687246	1.53E-03	5.8	5	5	6.1	6.2	6.1	mitochondrial carrier protein, putative	N/A
Up	PF3D7_0802000	0.685251	2.16E-03	11	11	12	12	12	12	glutamate dehydrogenase, putative	GDH3
Up	PF3D7_0604200	0.682299	1.32E-02	4.9	5	5	5.5	5.4	5.4	ribosomal protein L41, mitochondrial, putative	N/A
Up	PF3D7_1365600	0.68161	1.84E-03	4.2	5	5	5.2	5.2	5.4	DNA topoisomerase 6 subunit B, putative	TOP6B
Up	PF3D7_1302900	0.681441	6.95E-05	5.9	6	6	6.7	6.4	6.6	conserved protein, unknown function	N/A
Up	PF3D7_1354600	0.68022	1.03E-02	5.1	5	5	5.8	5.6	5.6	60S ribosomal protein L7-2, putative	N/A
Up	PF3D7_1229800	0.678977	5.78E-02	5	5	5	5.5	5.6	6	myosin J, putative	MyoJ
Up	PF3D7_1237800	0.67723	3.61E-04	5.6	5	5	5.9	6.2	5.9	ubiquitin-like modifier HUB1, putative	HUB1
Up	PF3D7_1235500	0.676419	4.57E-04	6.3	7	7	7.2	7.5	7.4	N6-adenosine-methyltransferase, putative	MT-A70.2
Up	PF3D7_0917800	0.674324	2.20E-02	4.3	4	4	4.7	4.7	4.7	conserved Plasmodium protein, unknown function	N/A
Up	PF3D7_0605400	0.673487	2.28E-02	4.5	5	4	5	4.7	5	calcium-binding protein, putative	N/A
Up	PF3D7_0320600	0.672498	1.70E-02	4.5	5	5	5.2	5.4	5.3	conserved protein, unknown function	N/A
Up	PF3D7_0214100	0.670541	1.70E-02	8.6	9	9	9.3	9.7	9.4	protein transport protein SEC31	SEC31
Up	PF3D7_1305500	0.669273	2.73E-02	4.4	4	4	5	4.9	4.9	mitogen-activated protein kinase phosphatase 1, putative	MKP1
Up	PF3D7_1237000	0.668517	4.31E-05	7.7	8	8	8.4	8.6	8.5	SUMO-activating enzyme subunit 2	UBA2
Up	PF3D7_0411100	0.668357	2.36E-03	5.1	5	5	5.7	5.6	5.6	mediator of RNA polymerase II transcription subunit 8, putative	MED8
Up	PF3D7_1315200	0.668095	9.96E-05	5.3	5	6	6.2	6	6.1	heptatricopeptide repeat-containing protein, putative	N/A
Up	PF3D7_0928600	0.663594	3.64E-03	5.2	5	5	5.8	5.4	5.6	conserved Plasmodium protein, unknown function	N/A
Up	PF3D7_0210800	0.66194	7.45E-02	4.3	4	4	4.8	4.8	5	conserved Plasmodium protein, unknown function	N/A
Up	PF3D7_0629600	0.654991	1.29E-02	4.5	5	5	5.3	5.1	5.1	ribosomal RNA-processing protein 7, putative	N/A
Up	PF3D7_1455400	0.645536	6.44E-05	6.5	6	6	7	6.9	7	hemolysin III	HlyIII
Up	PF3D7_0105500	0.644333	2.83E-02	4.4	4	4	4.9	4.8	4.8	conserved protein, unknown function	N/A
Up	PF3D7_1347000	0.640178	9.07E-02	3.7	4	4	4.1	4.5	4.1	WD repeat-containing protein 92, putative	WDR92
Up	PF3D7_0515900	0.637063	4.48E-05	6.4	6	6	6.9	6.8	7	NLI interacting factor-like phosphatase, putative	NIF2
Up	PF3D7_1032800	0.637011	1.93E-04	6.5	6	6	6.8	6.8	7	leucine-rich repeat protein	LRR1

Up	PF3D7_0922400	0.635505	1.42E-04	6.1	6	6	6.7	7	6.9	para-aminobenzoic acid synthetase	pBAS
Up	PF3D7_1030700	0.633258	6.53E-03	4.7	5	5	5.5	5.4	5.5	RNA methyltransferase, putative	N/A
Up	PF3D7_0623600	0.628771	1.62E-02	6.7	7	7	7.4	7.9	7.6	splicing factor-like protein 1, putative	N/A
Up	PF3D7_1213200	0.62782	2.73E-03	5.7	6	6	6.4	6.4	6.3	mediator of RNA polymerase II transcription subunit 18, putative	MED18
Up	PF3D7_1470000	0.627344	1.87E-02	5.3	5	5	5.6	6	5.8	autophagy-related protein 12, putative	ATG12
Up	PF3D7_0912000	0.626624	1.23E-03	8.7	8	9	9.2	9.3	9.3	conserved Plasmodium protein, unknown function	N/A
Up	PF3D7_1122000	0.622038	1.87E-02	5.5	5	5	5.8	5.8	5.8	conserved protein, unknown function	N/A
Up	PF3D7_0525500	0.621238	2.06E-04	6.2	6	7	7	7.1	7	WD repeat-containing protein, putative	N/A
Up	PF3D7_1005700	0.617861	1.39E-02	4.8	5	5	5.7	5.6	5.2	peptidase, putative	N/A
Up	PF3D7_0807400	0.614334	1.40E-02	4.4	4	5	4.9	5	5.1	coenzyme Q-binding protein COQ10 homolog, mitochondrial	COQ10
Up	PF3D7_1002600	0.612441	1.14E-04	6.4	6	6	6.7	6.8	6.7	conserved Plasmodium protein, unknown function	N/A
Up	PF3D7_1013300	0.610985	8.77E-04	6.5	6	6	6.7	7	6.8	conserved Plasmodium protein, unknown function	N/A
Up	PF3D7_0721500	0.610297	2.17E-02	5.6	5	5	5.8	6.1	6	conserved Plasmodium membrane protein, unknown function	N/A
Up	PF3D7_0409300	0.610178	3.38E-04	6.7	6	7	7.4	7.2	7.2	methyltransferase, putative	N/A
Up	PF3D7_1011100	0.609668	2.46E-04	5.9	6	6	6.5	6.4	6.5	conserved Plasmodium protein, unknown function	N/A
Up	PF3D7_0728400	0.609036	8.00E-03	5	5	5	5.5	5.7	5.7	SDH5 domain-containing protein, putative	N/A
Up	PF3D7_1344000	0.608669	4.63E-03	6.7	6	7	7.5	7.5	7.4	aminomethyltransferase, putative	N/A
Up	PF3D7_1108700	0.60728	1.35E-05	9.1	9	10	9.7	10	9.9	heat shock protein J2	Pfj2
Up	PF3D7_0817000	0.606737	4.59E-02	4.7	4	5	5	5	5.1	NEDD8-activating enzyme E1 catalytic subunit, putative	UBA3
Up	PF3D7_1212600	0.605777	1.87E-03	5.6	6	6	6.1	6.2	6.3	SND2 domain-containing protein, putative	N/A
Up	PF3D7_1346600	0.605179	1.96E-02	4.4	5	5	4.9	5.2	5.3	respiratory chain complex 2 associated protein 4	C2AP4
Up	PF3D7_0703200	0.60517	9.14E-04	8.9	9	9	9.6	9.9	9.8	conserved Plasmodium protein, unknown function	N/A
Up	PF3D7_1308600	0.6048	7.95E-03	5.7	5	6	6.1	6	6.1	conserved Plasmodium protein, unknown function	N/A
Up	PF3D7_1427400	0.602876	4.43E-05	6	6	6	6.8	6.6	6.6	conserved Plasmodium membrane protein, unknown function	N/A
Up	PF3D7_1355300	0.601328	1.47E-03	5.7	6	6	6.6	6.3	6.4	histone-lysine N-methyltransferase, putative	SET6

Up	PF3D7_1111300	0.601138	6.11E-03	5.2	5	5	5.5	6	5.9	protein transport protein BOS1, putative	BOS1
Up	PF3D7_0203100	0.600894	6.95E-03	6	6	6	6.6	6.7	6.7	protein kinase, putative	N/A
Up	PF3D7_0903100	0.600654	6.28E-03	5.5	5	5	5.7	6	5.8	protein RER1, putative	RER1
Up	PF3D7_1230500	0.59998	9.96E-02	4.2	5	5	5.1	5	5.1	WD repeat-containing protein, putative	N/A
Up	PF3D7_1110800	0.599344	4.67E-02	4.4	4	4	4.7	4.9	4.7	conserved Plasmodium protein, unknown function	N/A
Up	PF3D7_1338500	0.599186	1.16E-02	4.8	5	5	5.3	5.6	5.5	conserved Plasmodium protein, unknown function	N/A
Up	PF3D7_1449900	0.599132	4.19E-03	5.6	5	6	6.1	6	6	conserved protein, unknown function	N/A
Up	PF3D7_0302500	0.598468	2.86E-02	6.3	7	6	6.7	7	7.3	cytoadherence linked asexual protein 3.1	CLAG3.1
Up	PF3D7_1446500	0.597998	1.16E-03	9.4	10	10	10	10	10	nucleoporin NUP313, putative	NUP313
Up	PF3D7_0631000	0.597458	1.55E-02	4.9	4	5	5	5.1	5.1	tetratricopeptide repeat protein, putative	N/A
Up	PF3D7_1220200	0.597186	2.19E-02	4.6	5	5	5.1	5.5	5.5	conserved Plasmodium protein, unknown function, pseudogene	N/A
Up	PF3D7_0724500	0.597092	8.74E-02	3.7	4	4	4.6	4.1	4.4	conserved Plasmodium protein, unknown function	N/A
Up	PF3D7_0811200	0.596807	7.98E-08	7.7	8	8	8	8.4	8.2	ER membrane protein complex subunit 1, putative	EMC1
Up	PF3D7_1225900	0.596492	8.96E-04	7.1	7	7	7.2	7.5	7.6	conserved Plasmodium protein, unknown function	N/A
Up	PF3D7_0620700	0.596205	1.53E-03	5.3	5	5	5.7	5.7	5.7	DnaJ protein, putative	N/A
Up	PF3D7_0720500	0.594192	1.06E-03	5.8	6	5	6.3	6.1	6.2	conserved Plasmodium protein, unknown function	N/A
Up	PF3D7_1250800	0.590987	1.03E-03	8.8	9	9	9.6	9.8	9.7	DNA repair protein rhp16, putative	N/A
Up	PF3D7_0324100	0.590822	9.29E-02	4.8	5	5	5.7	5.6	5.5	Pfmc-2TM Maurer's cleft two transmembrane protein	MC-2TM
Up	PF3D7_1443700	0.590463	9.12E-04	6.9	6	6	6.9	7.1	7.1	dephospho-CoA kinase	DPCK
Up	PF3D7_1458200	0.589367	3.23E-02	4.5	5	5	5.3	5.2	5.4	conserved Plasmodium protein, unknown function	N/A
Up	PF3D7_0820400	0.587339	3.92E-02	4.9	5	5	5.5	5.6	5.2	conserved Plasmodium protein, unknown function	N/A
Up	PF3D7_1446300	0.586232	6.60E-03	6.2	6	7	7.1	6.9	7	conserved Plasmodium membrane protein, unknown function	N/A
Up	PF3D7_1365200	0.585291	2.37E-03	6	6	6	6.4	6.7	6.5	conserved protein, unknown function	N/A
Up	PF3D7_1433900	0.585138	1.20E-03	5.5	6	5	6	6.1	6.1	protein kinase, putative	N/A
Down	PF3D7_0727300	-5.200064	1.48E-23	12	10	13	7.1	7.1	7.1	DNA (cytosine-5)-methyltransferase	DNMT
Down	PF3D7_1101300	-5.190461	6.46E-05	3.7	3	3	2	2	2.1	rifin	RIF

Down	PF3D7_1149800	-5.035115	1.06E-03	3.2	3	2	2	2	2.1	rifin	RIF
Down	PF3D7_0617600	-4.099651	4.92E-07	4.9	4	5	2.4	2.2	2.4	stevor	N/A
Down	PF3D7_0713100	-3.934884	7.02E-28	5.2	5	5	2.4	2.7	2.9	Pfmc-2TM Maurer's cleft two transmembrane protein	MC-2TM
Down	PF3D7_1213100	-3.865552	3.99E-02	2	3	2	2	2	2	U1 spliceosomal RNA	N/A
Down	PF3D7_0713200	-3.537059	2.14E-03	3	3	3	2	2.2	2.1	exported protein family 1	EPF1
Down	PF3D7_0937500	-3.378049	8.83E-04	3.2	3	3	2.1	2	2.2	rifin	RIF
Down	PF3D7_0833500	-3.102284	1.48E-36	5.6	6	6	3.3	3.5	3.2	erythrocyte membrane protein 1, PfEMP1	VAR
Down	PF3D7_0700800	-3.098397	2.56E-12	4.5	5	5	3	2.7	2.9	Pfmc-2TM Maurer's cleft two transmembrane protein	MC-2TM
Down	PF3D7_1421150	-2.941239	1.73E-04	4	4	3	2.2	2	2.5	C/D small nucleolar RNA	N/A
Down	PF3D7_0713300	-2.623624	2.37E-03	3.4	3	3	2	2.3	2.3	erythrocyte membrane protein 1 (PfEMP1), pseudogene	VAR
Down	PF3D7_0114300	-2.055318	2.85E-10	5.4	5	5	3.6	3.4	3.4	exported protein family 4, pseudogene	EPF4
Down	PF3D7_0732900	-2.030022	1.17E-03	3.7	3	3	2.4	2.2	2.6	rifin	RIF
Down	PF3D7_1300500	-1.9781	1.75E-02	3.3	3	3	2.4	2	2.3	rifin	RIF
Down	PF3D7_1102300	-1.974535	4.50E-03	2.7	3	3	2.6	2.2	2.3	Plasmodium exported protein, unknown function	N/A
Down	PF3D7_0617500	-1.951141	4.62E-02	2.7	3	3	2.2	2.2	2.3	rifin, pseudogene	RIF
Down	PF3D7_1141100	-1.636493	5.33E-08	5.1	5	5	3.9	3.7	3.6	conserved Plasmodium protein, unknown function	N/A
Down	PF3D7_0423900	-1.626605	9.90E-02	2.3	2	3	2.2	2.2	2.3	probable protein, unknown function	N/A
Down	PF3D7_0114200	-1.609293	8.28E-02	3.4	3	3	2.4	2.7	2.5	exported protein family 3	EPF3
Down	PF3D7_0808900	-1.466012	5.30E-07	4.7	5	5	3.8	3.6	3.5	rifin	RIF
Down	PF3D7_0808800	-1.46485	2.37E-03	3.3	4	3	2.8	3	2.6	rifin	RIF
Down	PF3D7_1240300	-1.439107	2.29E-20	7.1	7	7	5.7	5.8	5.8	erythrocyte membrane protein 1, PfEMP1	VAR
Down	PF3D7_1322400	-1.420269	3.71E-02	3.4	3	3	2.5	2.7	2.4	conserved Plasmodium protein, unknown function	N/A
Down	PF3D7_0809100	-1.360196	6.95E-21	7.1	7	7	6	5.9	6.1	erythrocyte membrane protein 1, PfEMP1	VAR
Down	PF3D7_1477300	-1.306757	1.37E-05	5	5	4	3.8	3.5	3.8	Plasmodium exported protein (PHIST), unknown function	Pfg14-744
Down	PF3D7_1335000	-1.302849	7.37E-02	3.4	2	3	2.4	2.6	2.4	MSP7-like protein	MSRP1

Down	PF3D7_1372100	-1.289312	1.06E-03	4.9	5	5	4	3.7	3.6	Plasmodium exported protein (PHISTb), unknown function	GEXP04
Down	PF3D7_1477700	-1.251935	5.67E-04	4.7	4	4	3.6	3.6	3.5	Plasmodium exported protein (PHISTa), unknown function	Pfg14-748
Down	PF3D7_1006000	-1.211957	9.19E-02	2.7	3	3	2.4	2.3	2.5	IMP1-like protein, putative	IMP4
Down	PF3D7_1451600	-1.120348	8.16E-02	3	3	3	2.5	2.6	2.7	LCCL domain-containing protein	LAP5
Down	PF3D7_0929600	-1.089137	1.79E-02	3.7	4	3	2.6	2.4	3.2	G2 protein, putative	N/A
Down	PF3D7_1474000	-1.086319	1.76E-04	5.6	5	5	4.1	4.3	4.4	conserved Plasmodium protein, unknown function	N/A
Down	PF3D7_0301200	-1.01473	1.58E-02	3.4	4	4	3	3.1	3.1	serine/threonine protein kinase, FIKK family	FIKK3
Down	PF3D7_0108500	-0.98527	2.31E-04	6.2	6	6	5.4	5	5.4	ELM2 domain-containing protein, putative	N/A
Down	PF3D7_0206800	-0.983504	5.44E-05	9.4	9	9	8.2	8.4	8.6	merozoite surface protein 2	MSP2
Down	PF3D7_0936300	-0.965338	4.05E-02	4.4	4	5	3.6	3.1	4	ring-exported protein 3	REX3
Down	PF3D7_1372500	-0.9436	6.45E-02	3.3	4	3	2.9	3.1	2.9	stevor, pseudogene	N/A
Down	PF3D7_0823500	-0.933557	1.98E-02	4.2	4	4	2.9	3.1	3.5	inner membrane complex protein 1i, putative	IMC1i
Down	PF3D7_1473700	-0.922982	6.09E-03	6.6	7	7	5.7	5.6	5.7	nucleoporin NUP116/NSP116, putative	NUP116
Down	PF3D7_1209600	-0.915546	2.45E-03	4.6	4	4	3.8	3.5	3.8	porphobilinogen deaminase	PBGD
Down	PF3D7_0405300	-0.896877	3.87E-02	3.7	4	4	3.2	3.3	3.3	liver specific protein 2, putative	LISP2
Down	PF3D7_1141200	-0.841022	2.36E-04	6.5	6	6	5.3	5	5.4	ATP synthase-associated protein, putative	N/A
Down	PF3D7_1467600	-0.831313	2.31E-04	7.4	7	8	6.7	6.5	6.6	conserved Plasmodium protein, unknown function	N/A
Down	PF3D7_0501300	-0.802351	4.59E-03	6	6	7	5.7	5.5	5.6	skeleton-binding protein 1	SBP1
Down	PF3D7_0400300	-0.771579	3.16E-02	4.3	5	4	3.5	3.9	3.6	rifin	RIF
Down	PF3D7_0300100	-0.768534	5.43E-02	4.1	3	4	3.1	3.7	3.3	erythrocyte membrane protein 1, PfEMP1	VAR
Down	PF3D7_0723300	-0.759124	3.01E-07	7.6	8	7	6.7	6.9	6.9	conserved protein, unknown function	N/A
Down	PF3D7_0935900	-0.752362	9.22E-02	5.3	6	7	5.4	4.9	5.5	ring-exported protein 1	REX1
Down	PF3D7_0808700	-0.740807	2.65E-03	4.8	5	5	4.1	4.8	4.3	erythrocyte membrane protein 1, PfEMP1	VAR
Down	PF3D7_0933100	-0.729775	8.15E-05	5.8	5	6	5	4.7	5	conserved Plasmodium protein, unknown function	N/A
Down	PF3D7_0500800	-0.688018	3.26E-02	14	15	15	14	14	14	mature parasite-infected erythrocyte surface antigen	MESA
Down	PF3D7_1211800	-0.684909	1.18E-05	10	10	10	9.3	9.3	9.4	polyubiquitin	PfpUB
Down	PF3D7_1200200	-0.684063	5.88E-02	4.2	4	5	3.9	3.9	3.6	rifin	RIF

Down	PF3D7_0617300	-0.683824	6.52E-04	6.2	6	6	5.4	5.3	5.7	RNA-binding protein, putative	N/A
Down	PF3D7_0707300	-0.682412	9.40E-03	10	10	10	9	9.4	9.6	rhoptry-associated membrane antigen	RAMA
Down	PF3D7_0113000	-0.675077	1.35E-05	10	11	11	10	10	9.9	glutamic acid-rich protein GARP	GARP
Down	PF3D7_0931700	-0.672434	7.20E-03	5.8	6	5	5	4.9	4.9	PIH1 domain-containing protein, putative	N/A
Down	PF3D7_0400700	-0.667782	6.94E-02	4.3	4	4	3.8	3.8	3.6	rifin	RIF
Down	PF3D7_0503000	-0.666431	4.11E-11	8.2	8	8	7.6	7.5	7.6	50S ribosomal protein L28, apicoplast, putative	N/A
Down	PF3D7_1027300	-0.666189	4.58E-05	11	11	11	11	11	11	peroxiredoxin	nPrx
Down	PF3D7_1471700	-0.665863	9.81E-02	4.2	4	4	4	3.1	3.9	conserved Plasmodium protein, unknown function	N/A
Down	PF3D7_1247800	-0.656729	9.22E-02	4	4	4	3.3	3.1	3.5	dipeptidyl aminopeptidase 2	DPAP2
Down	PF3D7_1301700	-0.655366	5.57E-02	4.4	4	5	4.2	4.2	4.2	CX3CL1-binding protein 2	CBP2
Down	PF3D7_0937800	-0.6505	1.39E-04	7	7	7	6.6	6.4	6.5	erythrocyte membrane protein 1, PfEMP1	VAR
Down	PF3D7_1216500	-0.644819	1.72E-03	5.4	6	5	4.7	4.9	5	male development gene 1	MDV1
Down	PF3D7_0423300	-0.643772	9.02E-03	5.7	5	5	4.8	4.7	5.1	conserved Plasmodium protein, unknown function	N/A
Down	PF3D7_0324800	-0.643281	7.40E-02	4.7	5	4	4	3.8	4.2	rifin	RIF
Down	PF3D7_1426900	-0.634876	7.52E-06	7.5	8	7	6.8	6.9	6.8	cytochrome b-c1 complex subunit 6, putative	QCR6
Down	PF3D7_0520900	-0.62826	7.52E-06	12	12	12	11	11	11	adenosylhomocysteinase	SAHH
Down	PF3D7_1232300	-0.627818	9.56E-04	6.1	6	6	5.3	5.5	5.5	cytochrome b5, putative	N/A
Down	PF3D7_0921900	-0.625817	1.89E-10	8.7	9	9	8.1	8	8.1	conserved Plasmodium protein, unknown function	N/A
Down	PF3D7_0207000	-0.622903	3.72E-04	9.7	10	10	9	9.1	9.2	merozoite surface protein 4	MSP4
Down	PF3D7_0202400	-0.622514	5.88E-04	9.9	10	10	9.4	9.3	9.3	translation-enhancing factor	PTEF
Down	PF3D7_1413200	-0.622374	1.24E-02	5.6	6	6	5.1	4.8	5.2	conserved Plasmodium protein, unknown function	N/A
Down	PF3D7_1041300	-0.608559	3.47E-04	7.1	7	7	6.4	6.4	6.4	erythrocyte membrane protein 1, PfEMP1	VAR
Down	PF3D7_0733000	-0.604499	1.46E-02	5.1	5	5	4.7	4.7	4.4	erythrocyte membrane protein 1, PfEMP1	VAR
Down	PF3D7_0100100	-0.604214	2.88E-02	6.2	6	6	5.4	5.7	5.7	erythrocyte membrane protein 1, PfEMP1	VAR
Down	PF3D7_0922200	-0.600991	1.71E-04	13	13	13	12	12	12	S-adenosylmethionine synthetase	SAMS
Down	PF3D7_1476500	-0.600523	4.54E-04	6.2	6	6	5.7	5.5	5.5	probable protein, unknown function	N/A
Down	PF3D7_0107000	-0.599528	6.60E-02	9.2	9	9	8.6	8.7	8.6	centrin-1	CEN1
Down	PF3D7_0404300	-0.597423	4.37E-08	10	10	10	9.7	9.7	9.7	conserved Plasmodium protein, unknown function	N/A

Down	PF3D7_0214900	-0.590417	1.98E-02	7.7	8	8	6.6	7.2	7.2	rhoptry neck protein 6	RON6
Down	PF3D7_1353000	-0.587029	3.45E-10	11	11	11	10	10	10	tryptophan-rich protein, pseudogene	LysTrpA
Down	PF3D7_1105000	-0.585494	8.20E-04	11	11	10	10	9.9	10	histone H4	H4

Table S3L. Differential expression between PfDNMT2 disruption and complementation at the schizont stage

R: replicate, KO: PfDNMT2 disruptant, CO: PfDNMT2 complement, Adj.Pval: adjusted P value

columns G-L is EdgeR log2 (counts per million + count)

Change pattern	Gene ID	log2 Fold Change	Adj.Pval	CO R1	CO R2	CO R3	KO R1	KO R2	KO R3	Product Description	Name or Symbol
Up	PF3D7_1401000	2.6251291	2.75E-05	3.7	2.9	2.6	6	4.7	4.9	GBPH protein	GBPH
Up	PF3D7_0726000	2.6009214	5.51E-05	13	13	13	15	16	15	28S ribosomal RNA	N/A
Up	PF3D7_0532000	2.4980178	1.67E-04	12	12	13	14	16	15	28S ribosomal RNA	N/A
Up	PF3D7_0111200	2.2562384	1.35E-02	2.5	2.4	2.9	4	4	3.8	conserved protein, unknown function	N/A
Up	PF3D7_0223400	1.9772327	3.73E-02	2.9	2.7	2.4	4	3.4	3.8	rifin	RIF
Up	PF3D7_1319500	1.8142262	6.09E-02	3.5	2.4	2	4	3.7	3.4	conserved protein, unknown function	N/A
Up	PF3D7_1129850	1.4020127	3.98E-02	3.2	3.4	2.9	4	4.1	4.1	UNC-50 protein, putative, pseudogene	N/A
Up	PF3D7_0112700	1.3849145	3.08E-02	4.5	4.3	4.3	5	6.1	5.1	28S ribosomal RNA	N/A
Up	PF3D7_0223500	1.3729373	3.24E-07	5.2	5.2	5.5	7	6.3	6.6	erythrocyte membrane protein 1, PfEMP1	VAR
Up	PF3D7_0712000	1.2830011	2.11E-02	3.5	3.7	3.3	5	4.5	4.3	erythrocyte membrane protein 1, PfEMP1	VAR
Up	PF3D7_0929700	1.2700441	2.52E-02	3.7	3.5	3.4	4	4.7	4.3	conserved Plasmodium protein, unknown function	N/A
Up	PF3D7_1228000	1.2393289	6.37E-03	2.9	3.7	4.3	5	4.8	4.7	WPP motif-containing protein, putative	N/A
Up	PF3D7_0533100	1.1055905	3.16E-04	7.3	7.2	7.9	9	8.8	8.2	erythrocyte membrane protein 1 (PfEMP1), pseudogene	VAR1CSA
Up	PF3D7_1112500	1.0146919	3.81E-02	3.7	3.5	3.9	5	4.7	4.3	RNA transcription, translation and transport factor protein, putative	N/A
Up	PF3D7_1253400	0.9939353	3.68E-04	4.9	4.7	5.2	6	6	5.8	acyl-CoA synthetase	ACS3
Up	PF3D7_1038900	0.9019825	2.41E-02	4.1	4.3	4.5	5	5	5	esterase, putative	N/A
Up	PF3D7_0302500	0.8865881	2.65E-04	12	13	12	13	14	13	cytoadherence linked asexual protein 3.1	CLAG3.1
Up	PF3D7_1401200	0.8487667	2.61E-02	5.5	5.5	5.5	7	6.2	6.2	Plasmodium exported protein, unknown function	N/A
Up	PF3D7_0416600	0.8157175	2.08E-02	4.6	4.7	4.5	5	5.4	5.3	prohibitin-like protein PHBL, putative	PHBL
Up	PF3D7_0627200	0.786219	5.14E-02	4.1	4.7	4.4	5	5.3	5	myosin light chain, putative	N/A
Up	PF3D7_1204600	0.7532657	7.02E-02	5	3.5	4.1	5	5.3	4.7	cytidine deaminase, putative	N/A
Up	PF3D7_0727500	0.6923606	2.27E-03	5.9	5.8	5.7	6	6.6	6.3	mTERF domain-containing protein, putative	N/A

Up	PF3D7_0929900	0.6902403	3.41E-02	4.9	4.9	5	6	5.6	5.5	conserved Plasmodium protein, unknown function	N/A
Up	PF3D7_0417700	0.6881659	7.94E-02	5.3	4.8	5.2	6	5.8	5.4	conserved Plasmodium protein, unknown function	N/A
Up	PF3D7_0727400	0.6871775	5.55E-05	8	8.2	8.4	9	8.8	8.9	proteasome subunit alpha type-5, putative	N/A
Up	PF3D7_1429000	0.6857897	5.73E-02	4.2	4.6	4.7	5	5	5.2	protein archease, putative	N/A
Up	PF3D7_1024300	0.6822543	1.91E-02	5.4	5	4.9	6	5.7	5.6	ATP synthase-associated protein, putative	ATPTG10
Up	PF3D7_0414300	0.6611223	3.98E-02	5.1	4.9	5.2	6	5.5	5.8	Rab5-interacting protein, putative	N/A
Up	PF3D7_0805000	0.6556443	3.42E-02	5.9	5.5	5.5	7	5.9	5.9	alpha/beta hydrolase, putative	N/A
Up	PF3D7_1314300	0.6463859	7.38E-03	5.6	4.9	5.8	6	6.2	6	MACRO domain-containing protein, putative	N/A
Up	PF3D7_0310400	0.63948	2.01E-05	10	10	11	11	11	11	parasite-infected erythrocyte surface protein	PIESP1
Up	PF3D7_1416900	0.6390914	8.97E-02	4.9	4.3	4.9	6	5.5	5.1	prefoldin subunit 2, putative	N/A
Up	PF3D7_1455900	0.6248751	3.01E-02	5.2	5.5	5.2	6	5.8	5.9	polyprenol reductase	PPRD
Up	PF3D7_1430700	0.623206	1.88E-02	5.5	5.3	5.4	6	5.8	5.9	NADP-specific glutamate dehydrogenase	GDH2
Up	PF3D7_1342300	0.6221788	1.11E-02	5.7	5.4	5.7	6	6.3	6.1	tetratricopeptide repeat protein, putative	N/A
Up	PF3D7_0715400	0.6209559	4.60E-02	4.7	5	5.2	6	5.6	5.4	secreted ookinete protein, putative	PSOP20
Up	PF3D7_0919400	0.6198367	2.36E-06	8.3	8.4	8.6	9	9.1	9	protein disulfide-isomerase PDI-Trans	PDI-Trans
Up	PF3D7_0727100	0.6187661	6.74E-05	6.9	6.9	7.1	8	7.5	7.5	conserved protein, unknown function	N/A
Up	PF3D7_0623900	0.6142428	2.82E-02	5.4	5.1	5.6	6	6.1	5.7	ribonuclease H2 subunit A, putative	N/A
Up	PF3D7_1113700	0.5941474	3.58E-02	5.1	5.2	5.2	6	5.8	5.6	glyoxalase I	GLO1
Up	PF3D7_1134100	0.5937091	4.36E-07	7.5	7.8	7.7	8	8.1	8.3	protein disulfide-isomerase, putative	PDI-11
Up	PF3D7_1324800	0.5909959	3.68E-04	7.1	7.5	7.5	8	7.9	7.9	dihydrofolate synthase/folylpolyglutamate synthase	DHFS-FPGS
Up	PF3D7_1404400	0.5886902	6.80E-02	5.1	4.6	5.1	5	5.5	5.4	ribosomal protein L16, mitochondrial, putative	N/A
Down	PF3D7_0727300	-4.382629	9.54E-16	11	9.9	11	6	6.8	6.4	DNA (cytosine-5)-methyltransferase	DNMT
Down	PF3D7_0700800	-3.770339	1.04E-06	4.5	4.7	5.1	2	2.3	2.7	Pfmc-2TM Maurer's cleft two transmembrane protein	MC-2TM
Down	PF3D7_0713100	-3.626395	1.04E-06	5.3	4.4	3.9	3	2.3	2.5	Pfmc-2TM Maurer's cleft two transmembrane protein	MC-2TM
Down	PF3D7_0713200	-3.514096	1.04E-06	5	4.5	4.4	2	2.3	2.7	exported protein family 1	EPF1

Down	PF3D7_0713300	-2.867465	9.31E-10	5.1	5	5.1	3	2.6	3.1	erythrocyte membrane protein 1 (PfEMP1), pseudogene	VAR
Down	PF3D7_1101300	-2.505003	1.16E-03	4.1	3.9	4.3	3	3	2.5	rifin	RIF
Down	PF3D7_0833500	-2.38969	3.09E-39	7.6	7.6	7.9	6	5.2	5.5	erythrocyte membrane protein 1, PfEMP1	VAR
Down	PF3D7_1444900	-1.486619	3.15E-02	4.2	3.8	3.8	3	2.3	3.3	conserved protein, unknown function	N/A
Down	PF3D7_1447400	-1.344368	2.95E-03	4.9	4.8	4.7	4	3.6	3.8	conserved Plasmodium protein, unknown function	N/A
Down	PF3D7_1102900	-1.183571	7.17E-03	5.2	4.9	4.9	3	3.7	4.3	Plasmodium exported protein (hyp11), unknown function	N/A
Down	PF3D7_1128600	-1.179315	1.78E-02	4.2	4.7	4.9	3	3.9	3.8	CCR4-NOT transcription complex subunit 2, putative	NOT2
Down	PF3D7_0809100	-1.162211	8.80E-18	9.5	9.4	9.7	8	8.4	8.4	erythrocyte membrane protein 1, PfEMP1	VAR
Down	PF3D7_1200600	-1.158749	5.95E-03	8	7.2	7.7	6	6.8	6.7	erythrocyte membrane protein 1, PfEMP1	VAR2CSA
Down	PF3D7_0407300	-1.118236	9.13E-04	5	5.6	5.1	3	4.4	4.4	transcription factor, putative	N/A
Down	PF3D7_0937500	-1.117617	1.20E-02	4.9	4.9	5.3	4	4.5	3.8	rifin	RIF
Down	PF3D7_0528900	-1.078111	4.70E-02	5	5.5	4.5	3	4.6	4.3	conserved protein, unknown function	N/A
Down	PF3D7_0525900	-1.062263	8.73E-03	5.4	5.1	5.2	4	4.6	4.3	NIMA related kinase 2	NEK2
Down	PF3D7_0905600	-1.038726	6.72E-02	4.8	5.2	4.5	3	3.8	4.2	WD repeat-containing protein 66, putative	WDR66
Down	PF3D7_0724500	-1.013882	6.20E-03	5.6	5.1	4.4	4	4.3	4.1	conserved Plasmodium protein, unknown function	N/A
Down	PF3D7_0707500	-1.012082	6.49E-03	11	11	9.7	9	9	9.7	conserved Plasmodium protein, unknown function	N/A
Down	PF3D7_1110800	-1.009356	1.84E-03	5	5.7	5.5	4	4.4	4.6	conserved Plasmodium protein, unknown function	N/A
Down	PF3D7_0731000	-0.996944	1.62E-03	9.2	9.4	8.3	8	7.9	8.4	non-coding RNA	N/A
Down	PF3D7_1477700	-0.995763	2.12E-03	6.5	6.3	6.2	5	5.2	5.5	Plasmodium exported protein (PHISTa), unknown function	Pfg14-748
Down	PF3D7_1315000	-0.987087	2.53E-02	7	7.1	6.1	6	6	5.9	conserved protein, unknown function	N/A
Down	PF3D7_0922300	-0.964667	1.41E-03	6.2	5.6	5.7	5	4.6	5	conserved protein, unknown function	N/A
Down	PF3D7_0808900	-0.950491	5.78E-06	7.8	7.5	7.9	7	6.8	7	rifin	RIF
Down	PF3D7_0318800	-0.938567	4.20E-03	6	6.1	5.9	4	5.3	5.3	triosephosphate isomerase, putative	N/A
Down	PF3D7_0604900	-0.93675	9.38E-02	3.7	3.9	4.7	4	3.2	3.5	conserved Plasmodium protein, unknown function	N/A
Down	PF3D7_1477800	-0.919668	1.63E-03	8.4	8.8	8	7	7.4	7.7	acyl-CoA binding protein	ACBP

Down	PF3D7_1477300	-0.911002	6.01E-03	5.7	5.6	5.6	5	4.6	4.9	Plasmodium exported protein (PHIST), unknown function	Pfg14-744
Down	PF3D7_1363000	-0.905106	1.34E-02	5	5.1	5.4	4	4.6	4.3	conserved Plasmodium protein, unknown function	N/A
Down	PF3D7_1002000	-0.895639	2.83E-03	6.9	6.9	6.6	5	6.2	6	Plasmodium exported protein (hyp2), unknown function	N/A
Down	PF3D7_0630100	-0.887912	8.76E-04	7.7	7.9	7.4	6	7.1	6.8	alpha/beta hydrolase, putative	N/A
Down	PF3D7_0114000	-0.885257	2.50E-02	7.1	6.8	6.8	5	6.3	6.2	exported protein family 1	EPF1
Down	PF3D7_1220500	-0.878798	1.27E-02	5.3	5.5	5.3	5	4.1	4.8	ribosome biogenesis protein TSR3, putative	TSR3
Down	PF3D7_1252800	-0.878564	2.45E-05	11	11	10	9	9.7	9.7	Plasmodium exported protein (PHISTb), unknown function	N/A
Down	PF3D7_1133500	-0.872229	1.33E-02	5.8	5.7	5.3	4	5.4	4.6	conserved protein, unknown function	N/A
Down	PF3D7_0301800	-0.872042	1.27E-02	9.5	9.8	9	8	8.5	8.9	Plasmodium exported protein, unknown function	N/A
Down	PF3D7_0525300	-0.864039	1.13E-05	7.9	7.8	7.6	7	7.2	6.7	conserved protein, unknown function	N/A
Down	PF3D7_0800800	-0.85778	2.28E-03	6.4	6	6.1	6	5.5	5.2	Plasmodium exported protein (hyp7), unknown function	N/A
Down	PF3D7_1149300	-0.849999	1.05E-03	7.5	7.8	7.3	6	7	6.8	serine/threonine protein kinase, FIKK family	FIKK11
Down	PF3D7_1039100	-0.83571	1.78E-03	6.8	7.2	6.5	6	5.7	6.2	DnaJ protein, putative, pseudogene	N/A
Down	PF3D7_1114200	-0.835601	2.35E-06	9.2	9.4	8.8	8	8.1	8.5	GTPase-activating protein, putative	N/A
Down	PF3D7_0105000	-0.834904	3.27E-02	6.3	5.6	5.3	5	4.8	5.1	non-coding RNA	N/A
Down	PF3D7_1351800	-0.83317	9.13E-04	8.2	8.4	7.4	7	7.1	7.3	conserved Plasmodium protein, unknown function	N/A
Down	PF3D7_1302200	-0.826949	3.15E-02	5.7	6	5.4	4	5.3	5	protein UIS3	UIS3
Down	PF3D7_0500600	-0.826248	2.44E-02	6.4	5.5	6.3	5	5.4	5.4	stevor, pseudogene	N/A
Down	PF3D7_0424500	-0.822366	1.41E-03	9.8	9.8	9.3	9	8.7	9.1	serine/threonine protein kinase, FIKK family	FIKK4.1
Down	PF3D7_0702000	-0.809185	8.53E-04	9.1	9.5	8.9	8	8.4	8.7	Plasmodium exported protein (hyp12), unknown function	N/A
Down	PF3D7_1474000	-0.800998	1.01E-03	8.6	7.9	8.2	7	7.4	7.5	conserved Plasmodium protein, unknown function	N/A
Down	PF3D7_0906500	-0.800755	4.87E-06	9.7	9.9	9.3	9	8.9	9.1	arginase	N/A
Down	PF3D7_0502400	-0.797328	6.80E-04	8.8	8.9	8.2	7	7.9	8.1	merozoite surface protein 8	MSP8
Down	PF3D7_1401100	-0.796337	2.23E-03	11	11	11	10	10	10	DnaJ protein, putative	N/A
Down	PF3D7_1126800	-0.795893	8.70E-03	5.5	5.7	5.5	4	5	4.9	alternative splicing factor SR-MG, putative	SR-MG

Down	PF3D7_0324300	-0.794859	5.41E-02	4.8	4.7	4.5	3	4	4.1	exported protein family 4	EPF4
Down	PF3D7_1478000	-0.791673	4.53E-03	9.3	9.3	9	8	8.6	8.7	Plasmodium exported protein (PHISTa), unknown function	GEXP17
Down	PF3D7_0113700	-0.790882	7.38E-04	6.2	6.8	6.3	6	5.6	5.8	heat shock protein 40, type II	HSP40
Down	PF3D7_0304900	-0.790036	4.28E-02	5.1	4.9	4.3	5	4.1	4	conserved protein, unknown function	N/A
Down	PF3D7_1225000	-0.789185	8.78E-02	4.2	4.3	4.5	4	3	4	conserved Plasmodium protein, unknown function	N/A
Down	PF3D7_0814700	-0.788953	8.27E-02	5	5.2	5.2	4	4.5	4.6	conserved Plasmodium protein, unknown function	N/A
Down	PF3D7_1477400	-0.781709	9.96E-02	5.1	4.9	4.5	4	3.9	4.3	Plasmodium exported protein (PHIST), unknown function	N/A
Down	PF3D7_0702600	-0.780461	3.85E-02	5.8	5.4	5.4	4	5	4.9	chitinase, fragment	N/A
Down	PF3D7_0831500	-0.776162	5.55E-05	9.8	10	9.5	9	8.9	9.3	Plasmodium exported protein (PHIST), unknown function	N/A
Down	PF3D7_1337800	-0.773608	2.05E-05	11	12	11	10	11	11	calcium-dependent protein kinase 5	CDPK5
Down	PF3D7_1252500	-0.771448	6.46E-02	5.7	5.6	5.3	4	5	4.9	Plasmodium exported protein, unknown function	N/A
Down	PF3D7_1001900	-0.771389	1.67E-04	7.6	7.9	7.3	7	6.9	7	Plasmodium exported protein (hyp16), unknown function	PfJ23
Down	PF3D7_0315900	-0.768408	8.26E-03	6.4	6.7	6.1	6	5.7	5.7	conserved Plasmodium protein, unknown function	N/A
Down	PF3D7_0726100	-0.768184	5.66E-04	8.2	8.3	8.2	7	7.7	7.5	Plasmodium exported protein, unknown function	N/A
Down	PF3D7_1149600	-0.756881	6.99E-04	11	11	10	10	9.7	10	DnaJ protein, putative	N/A
Down	PF3D7_1026500	-0.749106	3.17E-04	8	8.2	7.7	7	7.4	7.4	conserved Plasmodium protein, unknown function	N/A
Down	PF3D7_1302000	-0.748313	6.55E-04	6.9	7.3	7.4	6	6.6	6.5	EMP1-trafficking protein	PTP6
Down	PF3D7_0926200	-0.745757	9.78E-02	7.9	7.9	6.3	6	7.3	6.8	conserved Plasmodium protein, unknown function	N/A
Down	PF3D7_1353100	-0.745404	1.88E-03	7.3	6.9	7	6	6.4	6.5	Plasmodium exported protein, unknown function	N/A
Down	PF3D7_0936300	-0.743782	6.10E-02	8.1	8.5	8.2	7	7.8	7.8	ring-exported protein 3	REX3
Down	PF3D7_1201200	-0.742283	4.20E-03	10	10	10	9	9.5	9.9	Plasmodium exported protein (PHISTa-like), unknown function	N/A
Down	PF3D7_1466800	-0.7395	9.10E-04	7.2	6.4	6.5	6	6	5.9	NOC3 domain-containing protein, putative	N/A
Down	PF3D7_0220700	-0.736787	7.61E-04	7.6	7.5	7.2	7	6.6	6.7	Plasmodium exported protein (hyp9), unknown function	N/A
Down	PF3D7_0401900	-0.736707	4.42E-04	11	11	11	10	10	10	acyl-CoA synthetase	ACS6

Down	PF3D7_1037400	-0.736513	7.11E-04	9.1	9.3	8.6	8	8.4	8.3	conserved Plasmodium protein, unknown function	N/A
Down	PF3D7_1252900	-0.734977	5.04E-02	6.2	6.5	5.9	6	5.5	5.6	Plasmodium exported protein, unknown function	N/A
Down	PF3D7_0402100	-0.732982	7.68E-04	12	12	12	11	11	12	Plasmodium exported protein (PHISTb), unknown function	N/A
Down	PF3D7_0814500	-0.732062	7.19E-03	8.5	8.5	7.8	7	7.5	7.9	conserved protein, unknown function	N/A
Down	PF3D7_0702300	-0.728218	1.62E-03	7.9	8	7.7	7	7.2	7.5	sporozoite threonine and asparagine-rich protein	STARP
Down	PF3D7_0220400	-0.728001	2.76E-02	5.8	6.2	5.3	5	5	5.2	DnaJ protein, putative, pseudogene	N/A
Down	PF3D7_0201700	-0.727552	5.11E-04	8.5	8.5	8.4	8	7.7	8	DnaJ protein, putative	N/A
Down	PF3D7_1404800	-0.726806	2.15E-03	11	11	11	10	10	11	conserved Plasmodium protein, unknown function	N/A
Down	PF3D7_0935900	-0.723167	9.06E-02	11	11	11	9	10	10	ring-exported protein 1	REX1
Down	PF3D7_1416400	-0.723147	1.90E-02	6.3	6.2	5.5	5	5	5.5	conserved protein, unknown function	N/A
Down	PF3D7_0202200	-0.720282	7.61E-04	8.6	8.9	8.6	8	8	8.1	EMP1-trafficking protein	PTP1
Down	PF3D7_0927400	-0.720254	1.29E-02	6.4	6.7	6.4	6	5.8	5.7	conserved Plasmodium protein, unknown function	N/A
Down	PF3D7_0702200	-0.72004	2.02E-04	11	11	10	10	9.7	10	lysophospholipase LPL20	LPL20
Down	PF3D7_1016800	-0.719472	3.35E-04	11	11	11	10	10	11	Plasmodium exported protein (PHISTc), unknown function	N/A
Down	PF3D7_1148900	-0.716957	1.91E-02	6	5.9	5.6	5	5	5.2	Plasmodium exported protein, unknown function	N/A
Down	PF3D7_0202500	-0.716124	9.59E-02	14	14	13	13	13	13	early transcribed membrane protein 2	ETRAMP2
Down	PF3D7_1416500	-0.71545	9.13E-04	10	10	10	10	9.5	9.7	NADP-specific glutamate dehydrogenase	GDH1
Down	PF3D7_1370300	-0.7144	5.28E-02	6.7	6.2	6.8	5	5.5	6.3	membrane associated histidine-rich protein 1	MAHRP1
Down	PF3D7_1352900	-0.714094	4.05E-02	9.3	9.6	8.9	8	8.8	8.7	Plasmodium exported protein, unknown function	N/A
Down	PF3D7_0423900	-0.712635	7.61E-04	8.4	8.8	8.4	8	7.8	8	probable protein, unknown function	N/A
Down	PF3D7_1423400	-0.711549	1.23E-02	6.4	6.7	6.3	5	6.1	5.8	conserved Plasmodium membrane protein, unknown function	N/A
Down	PF3D7_0830600	-0.711007	6.28E-03	8.5	8.5	7.9	7	7.8	7.8	Plasmodium exported protein (PHISTc), unknown function	N/A
Down	PF3D7_1108200	-0.710438	2.36E-06	8.3	8.3	8	7	7.7	7.4	D-tyrosyl-tRNA(Tyr) deacylase	DTD
Down	PF3D7_1146800	-0.710317	2.29E-02	5.5	5.5	5.4	4	4.9	4.9	conserved Plasmodium protein, unknown function	N/A
Down	PF3D7_1236200	-0.710228	9.54E-04	9.4	9	9.1	8	8.5	8.7	conserved Plasmodium protein, unknown function	N/A

Down	PF3D7_1450000	-0.709587	4.87E-06	9.2	9.1	9.1	8	8.5	8.5	serine/threonine protein kinase, putative	N/A
Down	PF3D7_0221700	-0.708812	1.39E-03	8.2	8.5	7.8	7	7.4	7.7	Plasmodium exported protein, unknown function	N/A
Down	PF3D7_1335200	-0.705669	5.00E-02	6.4	6.6	5.9	5	5.9	5.6	reticulocyte binding protein homologue 6, pseudogene	RH6
Down	PF3D7_1240300	-0.705372	1.56E-04	7.7	7.8	8.2	7	7.2	7.2	erythrocyte membrane protein 1, PfEMP1	VAR
Down	PF3D7_1407700	-0.701906	4.14E-02	5.9	6	5	5	5.1	5.1	conserved protein, unknown function	N/A
Down	PF3D7_1367400	-0.700804	3.05E-04	7.1	7.4	6.8	6	6.2	6.7	conserved Plasmodium protein, unknown function	N/A
Down	PF3D7_1102800	-0.700798	7.26E-02	14	15	14	13	14	14	early transcribed membrane protein 11.2	ETRAMP1 1.2
Down	PF3D7_0415800	-0.69826	1.05E-03	10	10	10	9	9.8	9.7	PhIL1-interacting candidate PIC3	N/A
Down	PF3D7_1102700	-0.697642	1.15E-02	13	13	12	12	12	12	early transcribed membrane protein 11.1	ETRAMP1 1.1
Down	PF3D7_1335500	-0.69729	6.49E-02	5.3	5.9	6	5	5.3	5.2	DNA replication complex GINS protein, putative	N/A
Down	PF3D7_1149500	-0.696726	3.87E-02	7.5	7.5	7.1	6	7.1	6.8	ring-infected erythrocyte surface antigen 2, pseudogene	RESA2
Down	PF3D7_1455100	-0.696306	5.29E-07	8.7	8.9	8.6	8	8	8	protein tyrosine phosphatase, putative	PTP1
Down	PF3D7_0202100	-0.696037	1.71E-03	12	12	12	11	11	12	liver stage associated protein 2	LSAP2
Down	PF3D7_0407900	-0.695057	3.64E-05	12	12	12	11	12	12	AAA family ATPase, putative	N/A
Down	PF3D7_0930200	-0.693009	6.89E-03	11	11	11	10	11	11	leucine-rich repeat protein	LRR8
Down	PF3D7_1001400	-0.692653	4.75E-03	7.4	7.7	7.1	7	6.5	6.9	exported lipase 1	XL1
Down	PF3D7_0424800	-0.692097	4.48E-02	7.9	8.1	7.5	6	7.5	7.3	Plasmodium exported protein (PHISTb), unknown function	N/A
Down	PF3D7_1016000	-0.691968	4.64E-03	11	11	10	10	10	10	conserved Plasmodium protein, unknown function	N/A
Down	PF3D7_1420100	-0.691798	1.81E-04	7	7	7.1	6	6.5	6.3	conserved Plasmodium protein, unknown function	N/A
Down	PF3D7_0805300	-0.690375	7.12E-03	12	12	12	11	12	11	zinc finger protein, putative	N/A
Down	PF3D7_1413800	-0.688919	3.82E-05	8.8	8.6	8.5	8	8.1	7.9	diphthamide biosynthesis protein 1, putative	DPH1
Down	PF3D7_0217200	-0.687502	6.99E-04	7.2	7.5	6.8	7	6.5	6.5	conserved Plasmodium protein, unknown function	N/A
Down	PF3D7_0402300	-0.687217	8.15E-02	14	14	14	13	14	14	reticulocyte binding protein homologue 1	RH1
Down	PF3D7_0108500	-0.686619	4.73E-03	12	12	11	10	11	11	ELM2 domain-containing protein, putative	N/A
Down	PF3D7_1321400	-0.685773	5.20E-06	8.5	8.6	8.5	8	8	7.8	palmitoyltransferase DHHC8, putative	DHHC8
Down	PF3D7_0910700	-0.685485	1.39E-03	6.7	6.9	6.9	6	6.4	6	actin-like protein, putative	ALP5a

Down	PF3D7_0713500	-0.685318	4.99E-03	7.5	7.3	7.2	6	6.6	6.9	conserved Plasmodium protein, unknown function	N/A
Down	PF3D7_0406200	-0.685106	1.46E-04	11	10	10	10	9.7	9.8	parasitophorous vacuole membrane protein S16	Pfs16
Down	PF3D7_1115800	-0.681187	4.59E-04	12	12	11	11	11	11	conserved Plasmodium protein, unknown function	N/A
Down	PF3D7_0702100	-0.680008	1.36E-02	11	11	10	10	9.5	10	Plasmodium exported protein (PHISTb), unknown function, pseudogene	N/A
Down	PF3D7_0622900	-0.679549	3.80E-06	12	12	11	11	11	11	AP2 domain transcription factor AP2Tel	AP2Tel
Down	PF3D7_0902500	-0.678092	5.43E-03	9.5	9.7	9.1	8	8.6	9.1	serine/threonine protein kinase, FIKK family	FIKK9.6
Down	PF3D7_1446300	-0.677844	4.31E-04	11	11	10	10	10	10	conserved Plasmodium membrane protein, unknown function	N/A
Down	PF3D7_0216700	-0.675247	5.04E-03	13	13	13	12	12	12	autophagy-related protein 11, putative	ATG11
Down	PF3D7_0934600	-0.674446	4.62E-02	9.1	9.2	9	8	8.7	8.6	conserved Plasmodium protein, unknown function	N/A
Down	PF3D7_1218100	-0.673451	2.50E-02	6.3	6.3	6.5	6	5.6	5.8	conserved Plasmodium protein, unknown function	N/A
Down	PF3D7_1462400	-0.67094	7.46E-05	9.4	9.4	8.9	8	8.7	8.6	conserved Plasmodium protein, unknown function	N/A
Down	PF3D7_0623800	-0.666282	9.13E-04	13	13	12	12	12	12	tyrosine kinase-like protein, putative	TKL4
Down	PF3D7_1116800	-0.665397	6.53E-06	13	13	13	12	13	13	heat shock protein 101	HSP101
Down	PF3D7_1401400	-0.664702	1.29E-02	12	12	12	11	11	12	early transcribed membrane protein 14.1	ETRAMP14
Down	PF3D7_0830500	-0.660988	4.84E-02	12	12	12	11	11	11	sporozoite and liver stage tryptophan-rich protein, putative	TryThrA
Down	PF3D7_0401600	-0.658679	1.43E-03	7.3	7.3	7.3	6	6.7	6.8	rifin	RIF
Down	PF3D7_0210800	-0.658095	6.74E-02	6	6.5	5.9	6	5.2	5.7	conserved Plasmodium protein, unknown function	N/A
Down	PF3D7_0204200	-0.657998	2.78E-06	11	11	11	10	11	10	translocation protein SEC66, putative	SEC66
Down	PF3D7_1419900	-0.654615	6.07E-03	6.5	5.9	6.4	6	5.7	5.6	YTH domain-containing protein 1, putative	YTH1
Down	PF3D7_0209500	-0.654195	5.81E-03	7.7	8.1	7.6	7	7.3	7.3	conserved Plasmodium protein, unknown function	N/A
Down	PF3D7_1001100	-0.653467	6.60E-03	8.5	8.6	8.1	8	7.8	7.8	acyl-CoA binding protein, isoform 1, ACBP1	ACBP1
Down	PF3D7_1355300	-0.653425	1.94E-02	5.7	6	5.9	5	5	5.4	histone-lysine N-methyltransferase, putative	SET6
Down	PF3D7_0936700	-0.65231	4.48E-02	5.5	5.3	5.3	5	4.8	4.8	lysophospholipase, putative	N/A
Down	PF3D7_1253000	-0.649155	8.55E-03	7	7.7	7	7	6.6	6.6	gametocyte erythrocyte cytosolic protein	GECO
Down	PF3D7_0625400	-0.649101	5.44E-04	10	10	9.6	9	9.5	9.4	conserved Plasmodium protein, unknown function	N/A
Down	PF3D7_1108300	-0.648259	2.52E-02	6	6.4	5.7	5	5.4	5.6	pterin-4a-carbinolamine dehydratase	PCD

Down	PF3D7_1105600	-0.647727	2.57E-04	12	12	11	11	11	11	translocon component PTEX88	PTEX88
Down	PF3D7_1138700	-0.647483	6.12E-05	11	12	11	10	11	11	protein KIC5	KIC5
Down	PF3D7_1125900	-0.645385	2.29E-03	8.6	8.7	8.4	8	8.1	8	XTBD domain-containing protein, putative	N/A
Down	PF3D7_1308500	-0.645269	1.16E-03	7.3	7.6	7	7	6.6	6.7	conserved Plasmodium protein, unknown function	N/A
Down	PF3D7_0724900	-0.644327	5.80E-03	11	11	11	10	10	10	kinesin-20, putative	N/A
Down	PF3D7_1439500	-0.643426	2.52E-11	9.9	9.9	9.7	9	9.2	9.1	oocyst rupture protein 2, putative	ORP2
Down	PF3D7_0808700	-0.64049	2.00E-04	8	7.8	8.1	7	7.4	7.4	erythrocyte membrane protein 1, PfEMP1	VAR
Down	PF3D7_0424600	-0.638841	6.31E-03	12	12	12	11	11	12	Plasmodium exported protein (PHISTb)	N/A
Down	PF3D7_0935700	-0.635184	3.43E-04	9	9.1	8.9	8	8.5	8.5	Plasmodium exported protein, unknown function	N/A
Down	PF3D7_1479000	-0.63492	1.94E-02	9.7	9.8	9.1	9	8.7	9.3	acyl-CoA synthetase	ACS1a
Down	PF3D7_0916700	-0.634366	4.43E-06	10	10	10	10	9.6	9.7	RNA-binding protein musashi, putative	HoMu
Down	PF3D7_1243400	-0.63421	3.16E-03	10	10	9.6	9	9.5	9.4	conserved Plasmodium protein, unknown function	N/A
Down	PF3D7_1218000	-0.631972	3.94E-04	9.7	9.8	9.4	9	9.2	9.1	thrombospondin-related apical membrane protein	TRAMP
Down	PF3D7_1436300	-0.63108	8.59E-04	13	14	13	13	13	13	translocon component PTEX150	PTEX150
Down	PF3D7_0104300	-0.630476	1.39E-04	14	14	14	13	13	14	ubiquitin carboxyl-terminal hydrolase 1, putative	UBP1
Down	PF3D7_0629600	-0.630455	4.68E-03	7.2	7.2	6.2	6	6.5	6.3	ribosomal RNA-processing protein 7, putative	N/A
Down	PF3D7_0323500	-0.630335	2.47E-08	9.9	10	9.8	9	9.4	9.3	survival motor neuron-like protein	SMN
Down	PF3D7_1001500	-0.629473	7.35E-02	9.2	9.5	9.1	8	8.7	8.9	early transcribed membrane protein 10.1	ETRAMP10
Down	PF3D7_1143800	-0.629096	6.71E-05	11	11	11	10	10	10	oocyst capsule protein Cap93, putative	CAP93
Down	PF3D7_0305300	-0.628131	6.04E-06	9.9	10	9.6	9	9.2	9.3	transporter, putative	N/A
Down	PF3D7_0310200	-0.627856	2.45E-05	13	13	12	12	12	12	phd finger protein, putative	N/A
Down	PF3D7_0906600	-0.62779	1.10E-04	9.4	9.4	9	9	8.4	8.9	zinc finger protein, putative	N/A
Down	PF3D7_0731200	-0.626589	4.62E-03	8.5	8.7	8.1	8	7.6	8.1	Plasmodium exported protein, unknown function	N/A
Down	PF3D7_0408000	-0.625065	2.20E-03	11	11	11	10	11	11	conserved Plasmodium protein, unknown function	N/A
Down	PF3D7_1232400	-0.622219	4.64E-03	8.1	8.3	7.8	7	7.5	7.7	CWC16 domain-containing protein, putative	N/A
Down	PF3D7_1039000	-0.621731	1.10E-02	7.1	6.8	7	6	6.4	6.4	serine/threonine protein kinase, FIKK family	FIKK10.2
Down	PF3D7_0210700	-0.620165	7.42E-03	9.6	9.4	8.9	8	8.9	8.7	syntaxin, Qa-SNARE family	SYN17

Down	PF3D7_0503600	-0.619088	5.04E-03	12	12	12	11	12	12	myosin B	MyoB
Down	PF3D7_0220000	-0.617016	5.44E-04	15	15	14	14	14	14	liver stage antigen 3	LSA3
Down	PF3D7_1002100	-0.616642	2.04E-03	11	11	11	10	11	11	EMP1-trafficking protein	PTP5
Down	PF3D7_1357700	-0.615385	9.98E-03	6.7	6.5	6.4	6	5.9	6.1	U3 small nucleolar RNA-associated protein 21, putative	UTP21
Down	PF3D7_0804700	-0.613868	2.32E-02	6.6	6.6	6.4	6	5.7	6.1	conserved Plasmodium protein, unknown function	N/A
Down	PF3D7_1016700	-0.611316	2.24E-03	8.5	8.5	8.2	8	7.8	7.9	Plasmodium exported protein (PHISTc), unknown function	N/A
Down	PF3D7_1127900	-0.611023	7.74E-05	9.8	9.8	9.8	9	9.4	9.2	conserved Plasmodium protein, unknown function	N/A
Down	PF3D7_0618800	-0.610405	5.80E-02	5.5	5.8	5.4	5	5.2	4.9	heptatricopeptide repeat-containing protein, putative	N/A
Down	PF3D7_0219900	-0.610393	2.83E-02	8.6	8.6	7.9	8	7.6	8	Plasmodium exported protein, unknown function	N/A
Down	PF3D7_1026400	-0.609882	1.60E-02	6.7	7	6.5	6	6	6.3	cell division cycle protein 20 homolog, putative	CDC20
Down	PF3D7_1431200	-0.608207	5.01E-04	8.9	9	8.5	8	8.3	8.4	OST-HTH associated domain protein, putative	N/A
Down	PF3D7_0831300	-0.606655	9.76E-04	7.4	7.4	7.2	7	6.8	6.8	Plasmodium exported protein, unknown function	GEXP13
Down	PF3D7_0730300	-0.605189	4.01E-04	14	14	13	13	13	13	AP2 domain transcription factor AP2-L, putative	ApiAP2
Down	PF3D7_1457700	-0.604206	5.43E-04	8.2	8.4	7.9	7	7.8	7.7	large ribosomal subunit nuclear export factor, putative	N/A
Down	PF3D7_1030300	-0.603453	2.61E-03	11	11	10	10	10	10	conserved Plasmodium protein, unknown function	N/A
Down	PF3D7_1336700	-0.602626	2.99E-04	8.9	8.6	8.4	8	8	8.1	parasitophorous vacuolar protein 3, putative	PV3
Down	PF3D7_0920600	-0.601588	2.16E-05	9.6	9.8	9.5	9	9.3	9	conserved Plasmodium protein, unknown function	N/A
Down	PF3D7_0425400	-0.601433	1.70E-02	7.9	7.8	7.5	7	7.2	7.3	Plasmodium exported protein (PHISTa), unknown function	N/A
Down	PF3D7_0808800	-0.601059	1.55E-02	6.8	6.4	6.8	6	6.3	6.1	rifin	RIF
Down	PF3D7_1031100	-0.596909	5.11E-02	5.9	5.9	5.8	5	5.3	5.4	conserved Plasmodium protein, unknown function	N/A
Down	PF3D7_1331500	-0.596053	1.73E-03	11	11	11	10	11	11	conserved Plasmodium protein, unknown function	N/A
Down	PF3D7_0425200	-0.595671	5.97E-02	6	6.3	5.6	5	5.5	5.5	Plasmodium exported protein (hyp15), unknown function	N/A
Down	PF3D7_0911400	-0.595475	1.45E-02	6.4	6.6	6	5	5.5	6	conserved Plasmodium protein, unknown function	N/A
Down	PF3D7_1253300	-0.595246	7.44E-02	5.9	5.4	5.7	5	5.3	5	Plasmodium exported protein (PHISTa), unknown function, pseudogene	N/A

Down	PF3D7_0113300	-0.595125	5.46E-03	6.8	6.3	6.4	6	6	6	Plasmodium exported protein (hyp1), unknown function	N/A
Down	PF3D7_1307500	-0.593282	7.67E-02	9.3	9.5	9.2	8	9	8.8	conserved Plasmodium protein, unknown function	N/A
Down	PF3D7_1224200	-0.593022	5.12E-04	9.8	10	9.5	9	9.3	9.2	BRO1 domain-containing protein, putative	N/A
Down	PF3D7_0214300	-0.592542	1.32E-03	7.7	7.8	7.5	7	7	7.1	conserved Plasmodium protein, unknown function	N/A
Down	PF3D7_0507300	-0.592282	1.31E-02	10	10	10	9	9.7	9.5	subtilisin-like ookinete protein SOPT	SOPT
Down	PF3D7_1313800	-0.587662	7.38E-04	11	12	11	10	11	11	conserved Plasmodium membrane protein, unknown function	N/A
Down	PF3D7_1146200	-0.587591	3.80E-02	6.4	6.9	6.9	6	6.2	6.2	conserved Plasmodium protein, unknown function	N/A
Down	PF3D7_0318600	-0.586665	1.78E-03	9.2	9.1	9.1	8	8.7	8.7	cleavage and polyadenylation specificity factor, putative	N/A
Down	PF3D7_0215300	-0.586385	7.49E-03	9.1	9	8.6	8	8.2	8.6	acyl-CoA synthetase	ACS8
Down	PF3D7_0903600	-0.586049	2.32E-03	13	13	13	12	13	12	conserved protein, unknown function	N/A
